# Unlocking Chromium Decarboxylative Ligand-to-Metal Charge Transfer: Efficient and Redox-Neutral Allylation of Aldehydes Using Carboxylic Acids

**DOI:** 10.1021/jacs.5c04691

**Published:** 2025-06-16

**Authors:** Supeng Wu, Ziqi Jiao, Alex T. Sung, Abigail B. Faulhaber, Nathan D. Schley, Alexander W. Schuppe

**Affiliations:** Department of Chemistry, 5718 Vanderbilt University, Nashville, Tennessee 37235, United States

## Abstract

Here, we report the light-induced decarboxylative ligand-to-metal charge transfer (LMCT) of Cr­(III) carboxylate complexes and demonstrate its applicability toward stereoselective Nozaki-Hiyama-Kishi (NHK) allylation reactions. The critical design element of our reaction was identifying a bipyridyl ligand scaffold that enables a single Cr catalyst to facilitate both photolytic dissociation and aldehyde addition. This approach allows for the direct utilization of carboxylic acids and eliminates the need for external redox reagents. The broad utility of this protocol was demonstrated by the preparation of a variety of homoallylic alcohols in good yields and diastereoselectivities as well as the identification of advantageous retrosynthetic disconnections. Extensive studies supported the LMCT mechanism of this transformation, including the characterization of the catalytically active Cr-carboxylate species.

## Graphical abstract

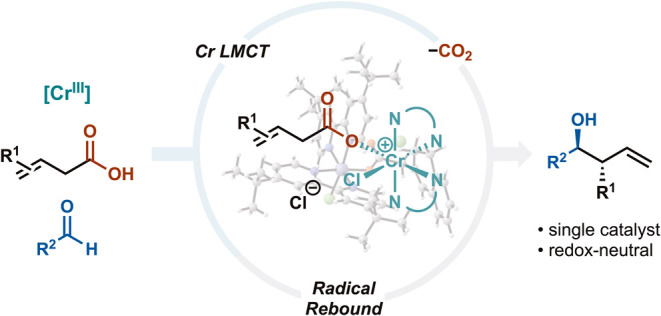

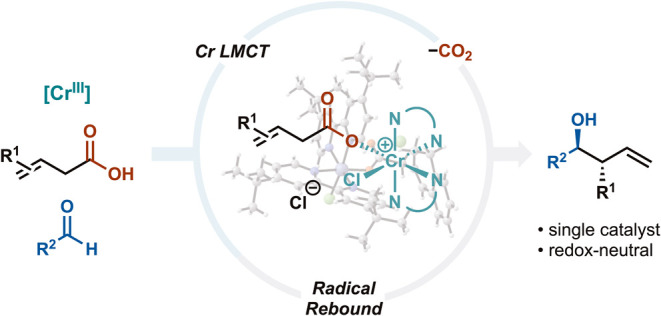

## Introduction

Decarboxylative coupling reactions represent an enabling strategy to construct new C–C or C-heteroatom bonds and convert abundant, bench-stable carboxylic acids into value-added products.^1^ Unlike thermally demanding two-electron decarboxylation,^2^ radical decarboxylative processes are generally spontaneous at room temperature due to stabilization of the resultant carbon-centered radical.^3,4^ Accordingly, various radical decarboxylative methods, including the Hunsdiecker-Borodin,^5^ Kochi,^6^ and Barton decarboxylation reactions,^7^ have found widespread applications in organic synthesis; nonetheless, their requirement for reactive halogen or metalloid reagents remains a significant drawback.^8^ More recently, milder decarboxylative conditions have been realized through the development of photo- and electrochemical methods that oxidize carboxylates through outer-sphere single-electron transfer (SET) (Scheme 1A).^1e,9,10^ However, these strategies necessitate matching the redox potential of the carboxylic acid (*E* ^0^_1/2_ = ca. 1.2–1.5 V vs SCE),^11^ which can pose considerable challenges in selectivity and tolerance for oxidatively labile functional groups.

**Scheme 1:**
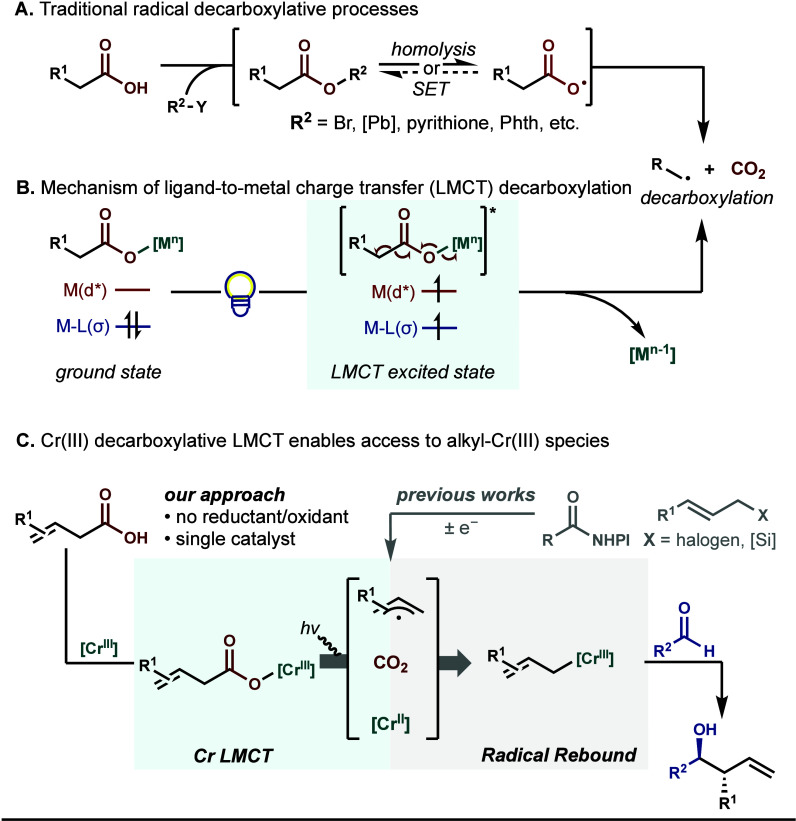
Rapid Access to Alkyl-Cr­(III) Species through Cr­(III) Decarboxylative LMCT

In contrast with outer-sphere electron transfer approaches, decarboxylative ligand-to-metal charge transfer (LMCT) is an inner-sphere process that is less dependent on the redox properties of the metal center and allows the utilization of complexes with short excited-state lifetimes.^12^ Mechanistically, light excitation of a metal-carboxylate complex results in the population of a metal-centered orbital with electron density from a ligand-centered orbital, triggering homolysis of the metal-carboxylate bond and formation of a carboxyl radical primed for decarboxylation (Scheme 1B).^13^ The resulting alkyl radical can engage in further downstream coupling to access a diverse array of functional groups (e.g., halides, azides, (hetero)­arenes).^14^ Despite growing applications of decarboxylative LMCT with transition metals (e.g., Fe­(III),^15^ Cu­(II),^16^ Ce­(IV),^17^ Ag­(II)^18^), their corresponding reduced states following LMCT are often unavailable to facilitate further reactivity, thus requiring stoichiometric amounts of metal complexes or oxidants.^16a^

To address this, we sought to utilize the unique reactivity of earth-abundant metal Cr, which exhibits a reactive Cr­(II) oxidation state that can readily engage carbon radicals to form versatile Cr­(III) reagents without external oxidants. Thus, harnessing the decarboxylative LMCT reactivity of trivalent Cr complexes could grant expedient access to organo-Cr­(III) nucleophiles from free carboxylic acids, obviating the need for cooperative catalysis to mediate subsequent bond formation steps. Specifically, we hypothesized that photolytic dissociation of a Cr­(III) carboxylate followed by decarboxylation could concomitantly produce a Cr­(II) complex and an alkyl radical, which could undergo facile recombination to yield an alkyl-Cr­(III) intermediate capable of constructing various C–C bonds (Scheme 1C).^19^ Specific reports supported the feasibility of this proposal,^20^ including the observation of a transient signal assigned to an alkyl-Cr species upon UV irradiation of polypyridyl Cr­(III) complexes in the presence of poly­(acrylic acid).^21^ Additionally, Wang recently demonstrated that photolysis of CrBr_3_ generated Br radicals that underwent hydrogen-atom transfer (HAT) with alkene pronucleophiles to form allyl-Cr­(III) reagents.^22^ However, the application of Cr decarboxylative LMCT to synthetic transformations remains elusive. We speculated that its implementation would be complicated by Cr­(III)’s slow rate of ligand substitution and deleterious pathways (e.g., protodemetalation or back electron transfer (BET)) resulting from reagent incompatibilities.^23^ To address these challenges, we reasoned that the ancillary ligand and additives must be carefully tuned to enable both photolytic dissociation and C–C bond-forming steps with a single Cr catalyst.

A prominent application of organochromium reagents is the Nozaki-Hiyama-Kishi (NHK) reaction. While prototypical NHK reaction conditions require highly reactive precursors (i.e., vinyl or allyl halides) and superstoichiometric heterogeneous reductants,^24,25^ recent investigations have exploited novel activation strategies (e.g., photocatalysis and HAT) to overcome specific limitations.^26^ Pioneering work by Baran, Reisman, and Blackmond demonstrated the decarboxylative coupling of *N*-hydroxyphthalimide (NHPI) esters with aldehydes through electrochemical catalysis, reimagining the use of abundant carboxylic acids as organochromium precursors.^27^ While these contemporary strategies have revolutionized catalytic generation of alkyl-Cr species, they often necessitate prefunctionalization and high loadings of pronucleophile reagents or redox mediators. Through our proposed decarboxylative LMCT paradigm, we envisioned that unmodified carboxylic acids could be directly utilized for the allylation of aldehydes, allowing us to construct homoallylic alcohol products from widely accessible feedstocks in a redox-neutral fashion.

## Results and Discussion

We commenced our investigation of the light-induced decarboxylative NHK reaction employing (*E*)-4-cyclohexylbut-3-enoic acid (**1a**) and 3-phenylpropanal (**2a**) as the model pronucleophile and electrophile, respectively (Table 1). Using the bench-stable Cr precatalyst [Cr­(dtbbpy)_2_Cl_2_]Cl (**Cr-1**) in conjunction with K_2_CO_3_, Et_3_SiCl, 4 Å molecular sieves, and 370 nm LED irradiation, we were able to form alcohol **3a** in 78% yield with high *anti* stereoselectivity (15:1 dr) without detection of the corresponding silylated analogue (entry 1). We discovered a marked dependence on the identity of the base, chlorosilane, and solvent, as alternative reaction conditions led to a precipitous decrease in the yield of **3a** (entries 2–5, see Supporting Information for additional optimization details). Furthermore, we observed that a delicate 2:1 molar ratio between Et_3_SiCl and K_2_CO_3_ was crucial for reaction success (see Table 2.4 in the Supporting Information for additional details). When the Cr precatalyst loading was lowered to 5 mol %, **3a** was formed in 40% yield but with a higher turnover number (entry 6), underscoring the potential efficiency of the transformation. Employing CrCl_3_ and dtbbpy instead of **Cr-1** resulted in a decreased yield of **3a**, as the prerequisite ligand associations are likely hindered by the coordinative inertness of Cr­(III) (entry 7).^23^ Evaluation of alternative bipyridyl ligand scaffolds, including 2,2’-bipyridyl (bpy, **Cr-2**), phenanthroline (phen, **Cr-3**), or terpyridine (terpy, **Cr-4**), resulted in minimal or no yield of **3a** (entries 8–10). Inferior results were obtained with 340 or 390 nm LEDs (entries 11–12). Control experiments demonstrated that light irradiation (entry 13) as well as each component of the reaction were indispensable for the formation of **3a** (entries 14–16).

**Table 1:**
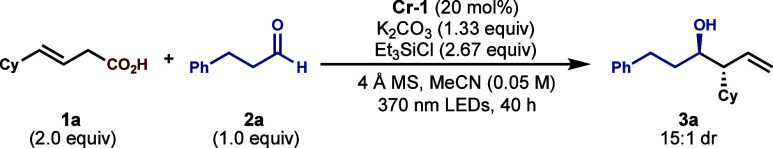

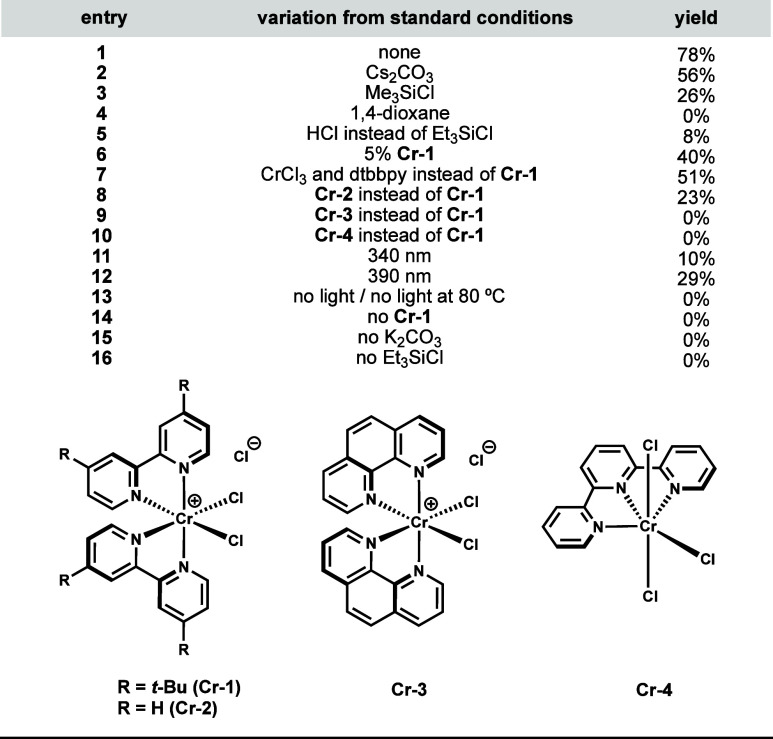
Optimization of Reaction Conditions^a^

a All reactions were conducted on a 0.10 mmol scale with respect to **2a**. Yields and diastereomeric ratios (dr) were determined by ^1^H NMR spectroscopy of the crude product mixtures using 1,1,2,2-tetrachloroethane (TCE) as an internal standard.

Having established the optimal set of reaction conditions, we investigated the scope of applicable aldehydes (Scheme 2). When **1a** was employed as the pronucleophile, a variety of products were formed with excellent yields and diastereoselectivities. Both aliphatic (**3b**–**3d**, **3f**, **3h**, and **3k**) and aromatic (**3e**, **3g**, **3i**, and **3j**) aldehydes as well as various pharmaceutically relevant substructures were well-tolerated, including aza-heterocycles (**3b**, **3d**, **3e**, and **3g**), an alkyl chloride (**3f**), an ester (**3h**), a furan (**3i**), and an amide (**3j**). Notably, the highly labile allylic C–H bonds present in the aldehyde component (**3k**) were accommodated under our protocol, which may prove incompatible with complementary allylation methods that leverage HAT to generate the allyl-Cr species.^22,26d,26e,26k^

**Scheme 2:**
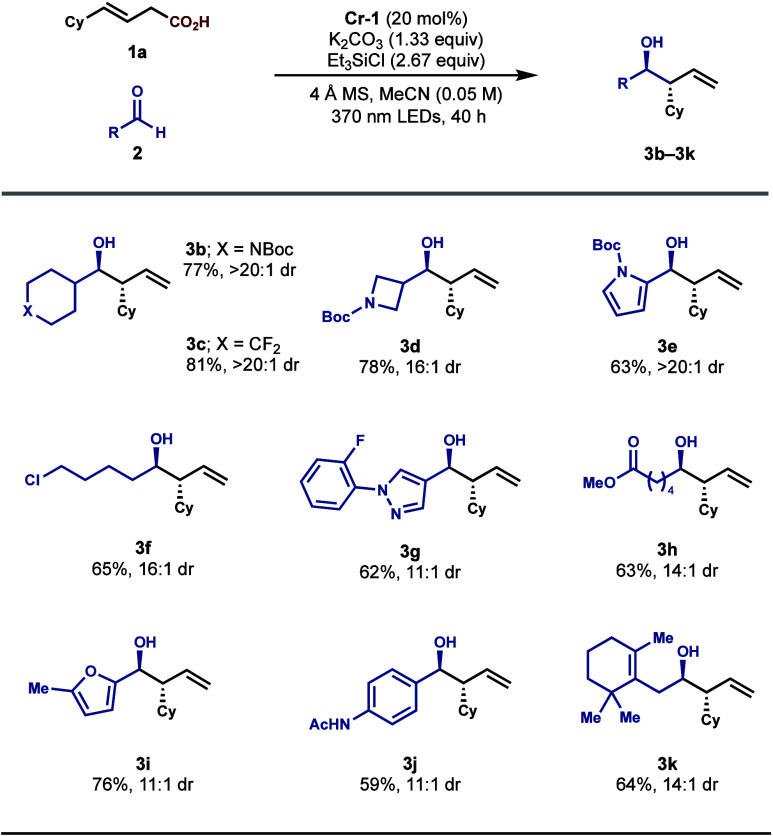
Aldehyde Scope^a^

a All reactions were conducted on a 0.30 mmol scale with respect to the aldehyde starting material (**2**) with 2 equiv of carboxylic acid (**1a**) unless otherwise noted. All yields were following flash column chromatography on silica gel; diastereomeric ratios (dr) were determined via ^1^H NMR.

We then shifted our focus to subjecting differentially substituted carboxylic acids to our decarboxylative NHK reaction (Scheme 3). We envisioned that a rapid decarboxylation event could be promoted by the stabilization of the resultant allyl radical through hyperconjugative effects with α- or γ-substituents. Indeed, carboxylic acids containing two such substituents provided, in general, the highest yields of the allylation adducts (**3l**–**3m**, **3v**, **3x**, **3y–3aa**, **3af**, **3ah–3ai**), while those bearing one α- or γ-substituent also furnished products in good yields and diastereoselectivities (**3n**–**3q**, **3w**, **3ab**–**3ac**, **3ae**, **3ag**). Moreover, employing vinyl acetic acid, which lacks stabilizing groups at these positions, provided **3ad** in 16% yield. Of note, products featuring methylidene motifs (**3r**–**3u**, **3aj**) were produced exclusively as a single isomer from carboxylic acids substituted at vinylic positions. Carboxylic acids bearing an α-heteroatom substituent, such as ethers (**3p**, **3u**, **3aj**), a benzoate (**3q**), and a carbamate (**3ab**), smoothly produced the corresponding products with selectivities comparable to prior studies involving allyl-Cr species.^26e,26f^

**Scheme 3:**
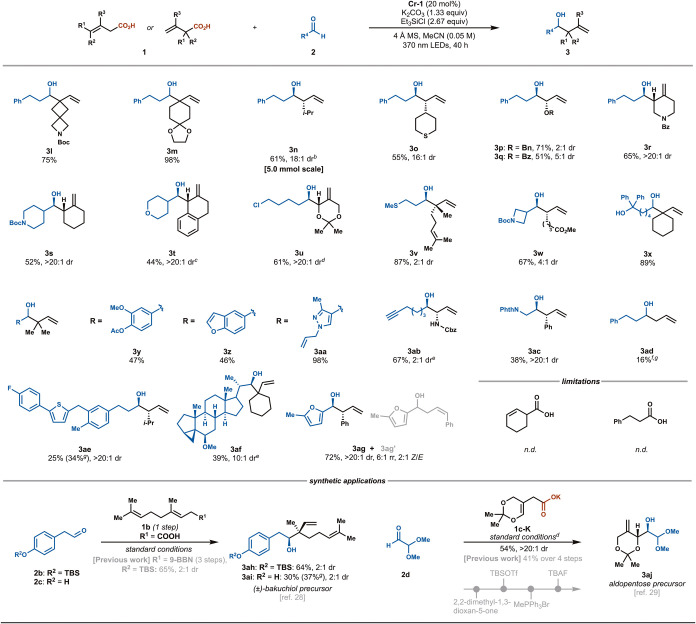
Scope of Differentially Substituted Carboxylic Acids^a^

a All reactions were conducted on a 0.30 mmol scale with respect to the aldehyde starting material (**2**) with 2 equiv of carboxylic acid (**1**) unless otherwise noted. All yields were following flash column chromatography on silica gel unless otherwise noted; diastereomeric ratios (dr) were determined via ^1^H NMR.
b Reaction performed for 88 h.
c Reaction conditions: carboxylic acid (**1**, 2.5 equiv), MeCN (0.025 M), 40 h.
d Reaction conditions: the corresponding potassium carboxylate (1 equiv), KHCO_3_ (1 equiv), Et_3_SiCl (2 equiv), MeCN (0.05 M), 18 h.
e Reaction conditions: carboxylic acid (1.5 equiv), K_2_CO_3_ (1 equiv), Et_3_SiCl (2 equiv), 40 h.
f
**Cr-1** (30 mol %) was used.
g Yield was determined by ^1^H NMR spectroscopy of the crude product mixture using 1,1,2,2-tetrachloroethane (TCE) as an internal standard.

Our transformation tolerates a variety of additional structural elements, including ketals (**3m**, **3u**, **3aj**), sulfides (**3o**, **3v**), a benzamide (**3r**), a free alcohol (**3x**), a terminal alkyne (**3ab**), and a free N–H bond (**3ab**). a phthalimide (**3ac**), and a phenol (**3ai**). A diverse array of heteroarenes could also be incorporated, including benzofuran (**3z**), pyrazole (**3aa**), thiophene (**3ae**), and furan (**3ag**). A canagliflozin derivative and a steroid derived from stigmasterol were successfully modified to give **3ae** and **3af** in moderate yields with high diastereoselectivities. While this transformation generally provided exquisite selectivity for the branched regioisomer (>20:1 rr), the coupling of *trans*-styrylacetic acid and 5-methylfurfural to synthesize **3ag** also yielded the linear addition isomer **3ag’** (6:1 rr, 2:1 *Z*/*E*). Notably, utilizing potassium carboxylate **1c-K** circumvented the instability of the corresponding carboxylic acid, producing **3u** and **3aj,** while the requisite protons for catalyst turnover were supplemented through substitution of K_2_CO_3_ to KHCO_3_. Performing the Cr-catalyzed process on a 5 mmol scale provided **3n** in a comparable yield; however, a prolonged reaction time was required (88 h), likely due to poor light penetration of the reaction mixture.

We further envisioned that our decarboxylative NHK protocol could complement the established allylation strategies toward the synthesis of natural products. For example, synthesis of (±)-bakuchiol precursor **3ah** via Brown allylation required the use of the highly sensitive borane reagent, geranyl 9-BBN, prepared in situ via hydroboration of the corresponding allene.^28^ Conversely, employing air- and moisture-stable carboxylic acid **1b**, synthesized in one step from commercially available material, and silyl-protected aldehyde **2b** furnished **3ah** in a comparable yield. Our protocol also tolerated the corresponding unprotected phenol **2c** to provide **3ai**, omitting the need for an inefficient protection–deprotection sequence. Moreover, although the homoallylic alcohol moiety could be a desirable synthon from β-hydroxyketones, its preparation often entails consecutive nonstrategic manipulations to account for functional group incompatibilities. While aldopentose precursor **3aj** had previously necessitated four synthetic steps,^29^ our decarboxylative NHK protocol provided an advantageous retrosynthetic disconnection, allowing us to access **3aj** directly from pronucleophile **1c-K** and aldehyde **2d** with 54% yield and >20:1 diastereoselectivity.

Although our Cr-catalyzed allylation process worked well on a range of substrate classes, several limitations remained. For example, we were unable to extend our substrate scope to 3-cyclohexenecarboxylic acid, likely due to the steric encumbrance of the resultant secondary allylic radical. Additionally, aliphatic carboxylic acids, such as hydrocinnamic acid, resulted in recovery of the starting material, possibly due to unproductive pathways (e.g., BET or HAT) dominating over decarboxylation to form a primary alkyl radical.

To gain insight into the mechanism of our light-induced decarboxylative NHK reaction, we conducted a series of mechanistic experiments (Scheme 4). While Cr­(III) species are known to be coordinatively inert,^23^ we initially suspected that differentially ligated chromium complexes responsible for different reactivities could be formed in situ*.* Thus, we independently synthesized [Cr­(dtbbpy)_3_]­(PF_6_)_3_ (**Cr-5**) and Cr­(dtbbpy)­Cl_3_ (**Cr-6**) to understand the likely speciation of the Cr precatalyst. When we performed the reaction with **Cr-5**, which has been shown to exhibit reductive quenching upon photoexcitation (*E*_1/2_(*Cr^III^/Cr^II^) = ca. 1.2 V vs SCE),^30^ **3a** was not observed, and 60% of acid **1a** was recovered (Scheme 4A). Subjecting **Cr-6** to the reaction conditions resulted in a diminished 22% yield. Noting an increase in yield upon the addition of 40 mol % dtbbpy, we hypothesized that **Cr-1** could be generated from **Cr-6** in situ via ligand association (see Supporting Information for additional spectroscopic details). Thus, we speculated that two bipyridyl ligands were necessary to facilitate photolysis of the Cr­(III) carboxylate adduct and generate the allyl-Cr intermediate.

**Scheme 4:**
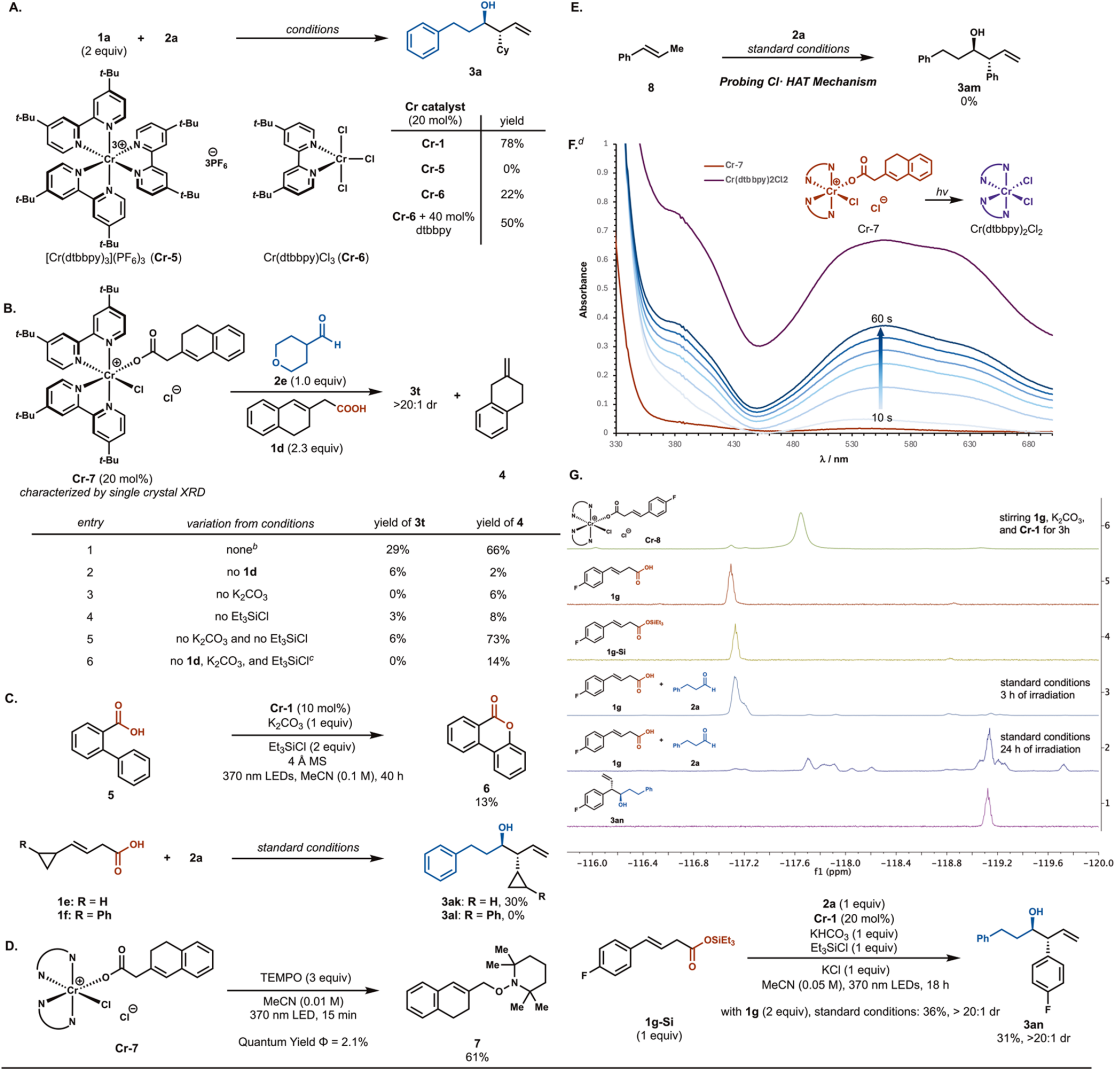
Mechanistic Investigations^a^

a Yields and dr determined by ^1^H NMR spectroscopy of the crude reaction mixture using 1,1,2,2-tetrachloroethane (TCE) as the internal standard, see Supporting Information for experimental details. (A) Investigating the in situ speciation of Cr precatalyst **Cr-1**. Standard conditions: **2a** (0.10 mmol), **1a** (2 equiv), Cr complex (20 mol %), K_2_CO_3_ (1.33 equiv), Et_3_SiCl (2.67 equiv) in MeCN (0.05 M) and irradiating for 40 h. (B) Decarboxylative NHK reactions with monocarboxylate complex **Cr-7**. Conditions: **2e** (0.10 mmol), **1d** (2.3 equiv), **Cr-7** (20 mol %), K_2_CO_3_ (1.33 equiv), Et_3_SiCl (2.67 equiv) in MeCN (0.025 M) and irradiating for 40 h. For entry 6, the reaction was performed with **Cr-7** (1 equiv) and **2e** (2 equiv), and the yield was calculated with respect to **Cr-7**. (C) Radical trap and radical probe experiments. Standard conditions for radical probe experiments: **2a** (0.10 mmol), carboxylic acid (2 equiv), **Cr-1** (20 mol %), K_2_CO_3_ (1.33 equiv), Et_3_SiCl (2.67 equiv) in MeCN (0.05 M) and irradiating for 40 h. (D) Quantum yield experiment. (E) Probing the chlorine radical HAT pathway. (F) UV–vis monitoring of **Cr-7** photolysis. All UV–vis spectra were acquired at 0.3 mM concentration. (G) ^19^F NMR monitoring of reaction progress and decarboxylative LMCT experiment employing **1g-Si** as pronucleophile.

With this knowledge, we proceeded to ascertain the identity of the catalytically active species in our reaction (Scheme 4B). Treating **Cr-1** with excess **1d** and K_2_CO_3_ afforded a Cr carboxylate complex (**Cr-7**), which was characterized by single-crystal X-ray diffraction and further supported by UV–vis spectroscopic studies (see Supporting Information for additional details). Employing **Cr-7** in lieu of **Cr-1** in conjunction with acid **1d** and aldehyde **2e** led to the formation of **3t** along with protodemetalated product **4** in 29 and 66% yield, respectively, which confirmed the catalytic competency of **Cr-7** (entry 1). Utilizing **Cr-7** in the absence of any carboxylic acid produced **3t** in 30% yield with respect to the Cr complex (entry 2). Intriguingly, we discovered that while neither K_2_CO_3_ nor Et_3_SiCl were essential for **Cr-7** to undergo LMCT, they proved crucial in the formation of the allylation product (entries 3–5). Additionally, a stoichiometric experiment using only **Cr-7** and **2e** did not produce **3t** (entry 6), suggesting that the presence of a Bro̷nsted acid is critical for aldehyde addition.

We then proceeded to examine the radical intermediates present in our reaction (Scheme 4C). When a benzoic acid derivative bearing an *ortho*-phenyl substituent (**5**) was subjected to the reaction conditions, lactone **6** was detected, presumably arising from intramolecular 6-*endo*-*trig* cyclization of a carboxyl radical intermediate.^10c,14a^ Radical probe experiments utilizing **1e** afforded the allylation product in 30% yield, while **1f**, which has a higher propensity to undergo ring-opening fragmentation (*k* ≈ 2 × 10^8^ s^–1^),^31^ only provided products derived from cyclopropyl ring-opening (see Supporting Information for additional details). Together, these experiments support the generation of an allyl radical upon decarboxylation and suggest a rate of Cr-radical rebound comparable to the rate of α-cyclopropylallyl radical ring-opening (*k* ≈ 6 × 10^4^ s^–1^).^31^

The photochemical efficiency of the decarboxylative LMCT process was also assessed. When **Cr-7** was irradiated in the presence of (2,2,6,6-tetramethylpiperidin-1-yl)­oxyl (TEMPO), adduct **7** was formed in 61% yield (Scheme 4D). We thereby determined the quantum yield for this radical trapping process to be 2.1%, which is comparable to Fe- or Cu-decarboxylative functionalization reactions triggered by LMCT.^32,33^

To further support the decarboxylative LMCT nature of our transformation, we considered the possibility of an alternative pathway promoted by Cr-halide LMCT (Scheme 4E).^22^ To test this hypothesis, we utilized *trans*-β-methylstyrene **8** instead of *trans*-styrylacetic acid, which would likely undergo facile HAT to form an allyl radical if any Cl radicals were generated. The absence of any allylation product (**3am**) suggested that this mechanism was not operative, compounded by our analysis that a direct HAT between the Cl radical and the acid substrate to form the carboxyl radical would not be polarity matched (see Supporting Information for additional details). Next, monitoring the photolytic dissociation of **Cr-7** by UV–vis spectroscopy revealed the development of significant absorption bands at 380 and 580 nm (Scheme 4F), which progressively converged to that of independently synthesized Cr­(dtbbpy)_2_Cl_2_.^34^ This observation was consistent with the dissociation of a carboxylate ligand and the generation of a Cr­(II) species upon LMCT. Moreover, the intermediacy of an allyl-Cr complex was established through the addition of MeOD to form the deuterated protodemetalation product, agreeing with previous studies by Knowles (see Supporting Information for additional details).^35^

Finally, by employing a fluorinated substrate (**1g**), we followed the speciation of the carboxylic acid through ^19^F NMR spectroscopy. Reacting **1g**, **Cr-1**, and K_2_CO_3_ resulted in a distinct signal that diminished upon irradiation, which was confirmed to be Cr carboxylate complex **Cr-8** by single-crystal X-ray diffraction (Scheme 4G, see Supporting Information for additional details). To study the role of Et_3_SiCl in the reaction, we synthesized the silylated derivative **1g-Si**. Monitoring the reaction between **1g** and **2a** revealed that **1g** gradually converted to **1g-Si** while the amount of allylation product **3an** increased over time. The plausible intermediacy of the silylated acid was further supported by the utilization of **1g-Si** as a pronucleophile to construct **3an** in comparable yield to **1g** (Scheme 4G, see Supporting Information for additional details).

Based on our preliminary mechanistic investigations, we propose a plausible catalytic cycle in Figure 1. Precatalyst **Cr-1** first undergoes ligand exchange with deprotonated carboxylic acid **1-K** to generate Cr-carboxylate species **I**. Upon light irradiation, **I** undergoes LMCT excitation to furnish a carboxyl radical and a Cr­(II) complex. An ensuing decarboxylation forms a stabilized allyl radical (**II**), which can engage the Cr­(II) complex to produce an allyl-Cr­(III) intermediate (**III**). The aldehyde electrophile then undergoes a nucleophilic addition through a Zimmerman-Traxler transition state to yield **IV**, which is protonated by a carbonic or carboxylic acid equivalent (i.e., triethylsilyl carbonic acid or pronucleophile **1**) to furnish homoallylic alcohol product **3**.

**Figure 1:**
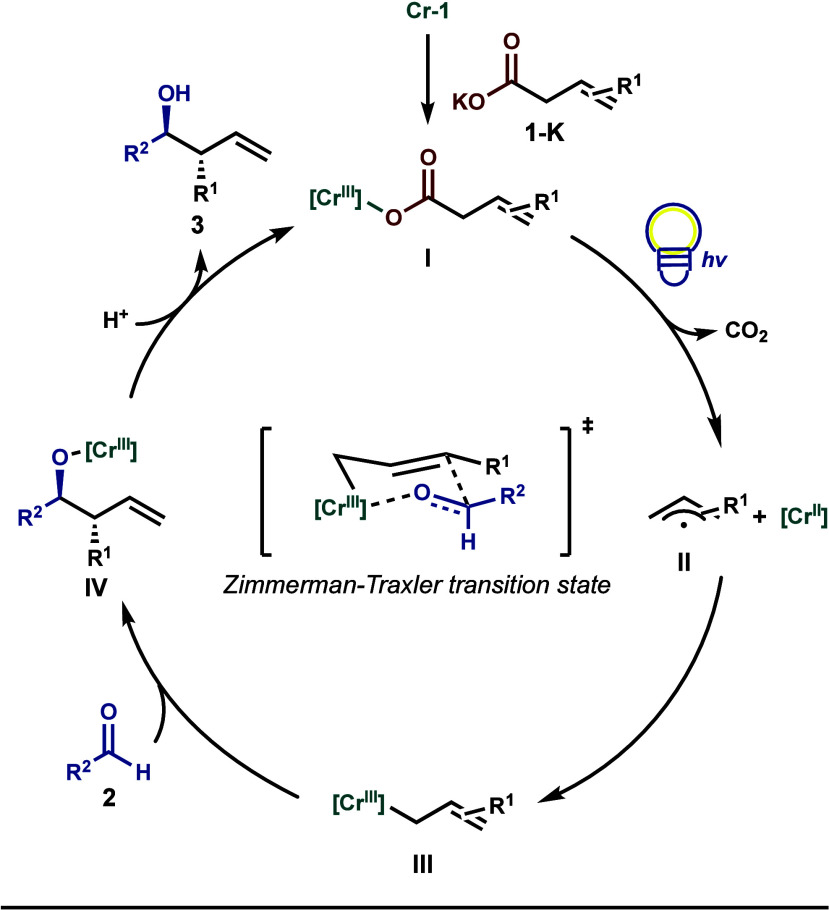
Proposed catalytic cycle.

## Conclusions

In summary, we demonstrated the propensity of Cr­(III)-carboxylate complexes to undergo LMCT and facilitate a redox-neutral decarboxylative NHK reaction utilizing abundant carboxylic acid starting materials. Our approach leverages careful ligand substitution and delicate additive loadings to enable a single Cr catalyst to promote both photolytic dissociation and C–C bond-formation steps. Our strategy allows for the formation of a diverse range of homoallylic alcohols with high yields and diastereoselectivities while also offering novel retrosynthetic disconnections to complement existing allylation and aldol reaction paradigms. By probing the elementary steps of our transformation, we provide further understanding of the nature of the LMCT process and offer insight for future investigations.

## Supplementary Material



## Notes

Materials and methods, computational data, experimental procedures, mechanistic studies, optimization studies, NMR spectra, and other characterization data (PDF)
