# Supplementary material for: Unlocking Chromium Decarboxylative Ligand-to-Metal Charge Transfer: Efficient and Redox-Neutral Allylation of Aldehydes Using Carboxylic Acids
Source: J Am Chem Soc. 2025 Jun 16;147(26):22759–67. doi: 10.1021/jacs.5c04691 (PMC12232319; doi:10.1021/jacs.5c04691)
Supplement: Supplementary file 1 [file ja5c04691_si_001.pdf]

Supplementary Information

Unlocking Chromium Decarboxylative Ligand-to-Metal Charge Transfer: Efficient and Redox-Neutral Allylation of Aldehydes Using Carboxylic Acids

Supupeng Wu†, Ziqi Jiao†, Alex T. Sung, Abigail B. Faulhaber, Nathan D. Schley, and Alexander W. Schuppe.

Department of Chemistry, Vanderbilt University, Nashville, Tennessee, 37235, United States

Corresponding author: *alexander.w.schuppe@vanderbilt.edu*

Table of Contents

1. General Experimental Details **4**

2. Optimization of the Reaction Conditions **6**

2.1. **General Procedure A**: Optimization Studies 6

3. Synthesis and Characterization of Starting Materials **14**

3.1. **General Procedure B**: Synthesis of Bis-Bipyridyl Chromium Complexes 16

3.2. Synthesis and Characterization of Chromium Complexes **Cr-5**–**Cr-7** 20

3.3. Synthesis and Characterization of Carboxylic Acid Starting Materials 22

3.3.1. **General Procedure C**: Purification by Reversed-Phase Chromatography 22

3.3.2. **General Procedure D**: Synthesis of β,γ-unsaturated Carboxylic Acids **SI-2**–**SI-3**, **1b**, **1e**, **1f** 22

3.3.3. **General Procedure E**: Synthesis and Characterization of Carboxylic Acids **SI-6** and **1c-K** 26

3.3.4. Synthesis and Characterization of Carboxylic Acids **SI-7**–**SI-10**, and **1d** 29

3.4. Synthesis and Characterization of Aldehydes **SI-11**–**SI-12**, and **2d** 34

4. General Procedures for Decarboxylative Nozaki-Hiyama-Kishi Coupling **37**

4.1. **General Procedure F**: Decarboxylative Nozaki-Hiyama-Kishi Coupling 37

4.1.1. **Graphical General Procedure F**: Decarboxylative Nozaki-Hiyama-Kishi Coupling 38

5. Synthesis and Characterization of Decarboxylative Nozaki-Hiyama-Kishi Coupling Products **40**

5.1. **General Procedure G**: Synthesis of 3n through Gram-Scale Decarboxylative Nozaki-Hiyama-Kishi Coupling 65

5.1.1 **Graphical General Procedure G**: Gram-Scale Decarboxylative Nozaki-Hiyama-Kishi Coupling 66

6. Mechanistic Experiments **68**

6.1. **General Procedure H**: Mechanistic Experiments 68

6.2. Experiments with $[Cr(dtbbpy)_3](PF_6)_3$ (**Cr-5**) and $Cr(dtbbpy)Cl_3$ (**Cr-6**) 69

6.3. Decarboxylative LMCT-NHK Experiments with **Cr-7** 72

6.4. Radical Cyclization Experiments 76

6.5. Radical Trap and Radical Probe Experiments 77

6.6. Quantum Yield Measurement using Ferrioxalate Actinometry 80

6.7. Probing Chlorine Radical HAT Pathway 83

6.8. UV-Vis Spectroscopy 84

6.9 ^{19}F NMR Experiments 93

6.10. Cyclic Voltammetry (CV) Experiments 102

6.11. Fluorescence Measurement ... 107
6.12. Deuteration Experiment ... 108
6.13. *in situ* IR Monitoring of **1g-Si** Desilylation ... 110
6.14. Demonstrating Regioselectivity of Using Carboxylic Acids as Pronucleophiles over Olefins ... 113
6.15. Investigating NHK Allylation Efficiency of **Cr-1**–**Cr-3** ... 115
7. Associated Analytical Data ... 118
7.1. Crystallographic Data ... 118
7.2. Associated NMR Spectra ... 129
7.2.1. NMR Spectra of Starting Materials ... 129
7.2.2. NMR Spectra of Products ... 145
8. References ... 203

1. General Experimental Details

General Experimental Procedures: All reactions were performed in flame-dried or oven-dried (at 140 °C) glassware fitted with rubber or PTFE/silicone septa under a positive pressure of N_2, unless otherwise noted. Standard reactions were performed in glass reaction tubes with crimp-tops (Thermo Scientific, catalog no. CHCV20-14; oven-dried at 140 °C) equipped with a magnetic stir bar (Chemglass Life Sciences, catalog no. CG-2003-17, 12.7 x 3 mm) and sealed with a DWK Life Sciences PTFE/Silicone lined aluminum crimp vial seal (Thermo Scientific, catalog no. 15-111-703). Air- and moisture-sensitive liquids were transferred via syringe through rubber or PTFE/silicone septa. Solids were added under inert gas counter flow or were dissolved in the appropriate solvent. Reactions carried out at temperatures above room temperature (r.t.) were conducted in a pre-heated oil bath.

All reactions were magnetically stirred and monitored by ^{1}H NMR spectroscopy, gas chromatography/mass spectrometry (GC/MS), or analytical thin-layer chromatography (TLC), using glass-backed plates precoated with silica gel (250 µm, 60-Å pore size, Extra Hard Layer, SilicaPlate) impregnated with a fluorescent indicator (254 nm). TLC plates were visualized by exposure to ultraviolet light (UV) or were stained by submersion in iodine dispersed in SiO_2 (I_2), an acidic solution of *p*-anisaldehyde (PAA), an acidic solution of cerium ammonium molybdate (CAM), or an aqueous potassium permanganate solution ($KMnO_4$) and were developed by heating with a heat gun. Flash column chromatography was performed using SiliCycle SilicaFlash® P60 silica gel (40–63 µm, 230–400 mesh, 60-Å pore diameter). Automated column chromatography was performed using a BUCHI C-810 Pure Chromatography System using prepacked SNAP silica cartridges (10–100 g). The yields refer to chromatographically and spectroscopically (^{1}H and ^{13}C NMR) pure material. All ^{1}H NMR yields are corrected. For light irradiation, Kessil PR160L-Blue LED lamps (λ_{max}= 370 Gen 2, 390; max 44, 52 W, respectively) and a Lucent360 side light module (340 nm, HCK1021-01-043, max 54 W) at 100% intensity were placed 3 cm away from the reaction vials in a custom-made temperature-controlled LED photoreactor setup or a Lucent360 advanced photoreactor.

Materials: Unless noted otherwise, all reagents and starting materials were purchased from commercial sources and used as received (Millipore Sigma, Thermo Fisher Scientific, Strem, TCIAmerica, Combi-Blocks, Ambeed, Oakwood Chemical, or Matrix Scientific). $CDCl_3$ was purchased from Milipore Sigma. Tetrahydrofuran (THF), acetonitrile (MeCN), toluene (PhMe), dimethylformamide (DMF), and dichloromethane (CH_2Cl_2) were obtained from Fisher Scientific and purified by successive filtrations though packed columns of neutral alumina or 4 Å molecular sieves under N_2 pressure. Solvents for extraction, crystallization, and flash column chromatography were purchased in ACS grade from Fisher Scientific.

Instrumentation: NMR spectra were measured on Bruker Avance III HD 400, 500, or 600 MHz spectrometers. Proton chemical shifts are expressed in parts per million (ppm, δ scale) and are referenced to the residual proton in the NMR solvent ($CDCl_3$: δ 7.26). ^{1}H NMR spectroscopic data are reported as follows: Chemical shift in ppm (multiplicity, coupling constants J (Hz), integration intensity). The multiplicities are abbreviated with s

(singlet), br s (broad singlet), d (doublet), t (triplet), q (quartet), p (pentet), and m (multiplet). All ^{13}C spectra recorded are proton-decoupled. The carbon chemical shifts are expressed in parts per million (ppm, δ scale) and are referenced to the carbon resonance of the NMR solvent ($CDCl_3$: δ 77.16). ^{13}C NMR spectroscopic data are reported as follows: Chemical shift in ppm (multiplicity, coupling constants J (Hz)). All ^{19}F spectra were acquired without proton-decoupling. The ^{19}F chemical shifts are expressed in parts per million (ppm, δ scale). All raw ".fid" files were processed and analyzed using MestReNOVA 14.3 from Mestrelab Research S. L. High-resolution mass spectra were obtained on a LTQ Orbitrap XL™ Hybrid FT MassSpectrometer and an Agilent Technologies 6550 Q-TOF LC/MS system using an Agilent Zorbax 300 SB-C3 (2.1 × 150 mm, 5-μm particle size). XRD structures were visualized with Cylview: Legault, C. Y. Cylview20; University of De Sherbrooke, 2020.[1] Syringe pumps (BS-300) were purchased from Braintree Scientific, Inc. Gas chromatograms were collected on an Agilent Technologies 6890N GC with attached 5977 MSD tuned to an ionization energy of 70 eV. UV-Vis data was collected using a Hitachi U-3000 spectrophotometer utilizing the UV solutions software (program no.: 1344331-15). All samples were measured in absorbance mode with a wavelength range from 250–700 nm and a slit width of 2.0 mm. Fluorescence data was obtained using a Varian Cary Eclipse Fluorescence Spectrophotometer. FTIR spectra were obtained on a Nicolet iS5 spectrometer equipped with an iD5 diamond laminate ATR accessory from Thermo Scientific. FTIR spectra were acquired from thin-film, neat samples. If required, substances were dissolved in CH_2Cl_2 prior to direct application on the ATR unit. Data are reported as follows: frequency of absorption (cm^{-1}). Melting points were determined on a Mel-Temp® 3.0 capillary system. The reported values are uncorrected. Cyclic voltammograms were collected on a Pine Research WaveDriver 40 DC Bipotentiostat against an $Ag/AgNO_3$ reference electrode in MeCN and calibrated against ferrocenium (Fc^+). Specific optical rotations were recorded for chloroform solutions using a Rudolph Research Autopol IV automatic polarimeter operating at 589 nm and 23 °C and a 0.5 dm polarimeter cell.

2. Optimization of the Reaction Conditions

2.1. General Procedure A: Optimization Studies

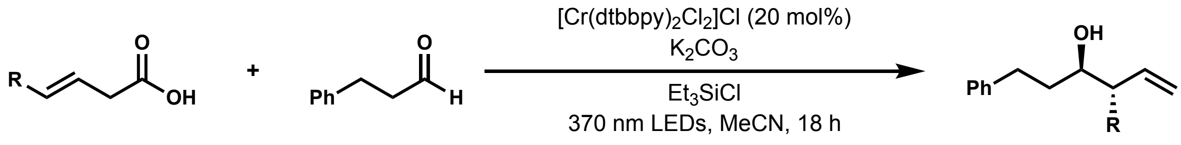

1 2a 3

Inside a N_2-filled glovebox, to an oven-dried reaction vial (VWR, catalog no. 66011-041) equipped with a magnetic stir bar (Chemglass Life Sciences, catalog no. CG-2003-160, 10 x 3 mm) was charged the corresponding carboxylic acid (if solid), K_2CO_3, $[Cr(dtbbpy)_2Cl_2]Cl$ (14 mg, 0.02 mmol, 20 mol%), and MeCN. The reaction mixture was allowed to stir for 5 min. To the stirred reaction mixture was added the corresponding carboxylic acid (if liquid), aldehyde (**2a**) (13 μL, 0.10 mmol, 1.0 equiv), and Et_3SiCl. The reaction vessel was sealed with a PTFE-lined phenolic vial screw cap (Thermo Scientific, catalog no. 03-375-25A with 03-340-10G), removed from the glovebox, and placed in a custom-made photoreactor 3 cm away from two 44 W Kessil PR-160L 370 nm LEDs and one 75 mm fan. The reaction mixture was subjected to LED irradiation at 100% intensity with vigorous stirring (*ca.* 50 °C). After completion, the reaction mixture was allowed to cool to room temperature, then filtered through a short silica plug eluting with EtOAc (3 x 2 mL) and concentrated *in vacuo* with the aid of a rotary evaporator. Yield was evaluated by ^1H NMR of the crude reaction mixture using 1,1,2,2-tetrachloroethane (TCE) (*ca.* 6 μL) as the internal standard.

<u>Note</u>: vigorous stirring of the reaction mixture was crucial to ensure reaction mixture homogeneity and maintain a reproducible yield.

Table 2.1. General Optimization Experiments and Control Reactions

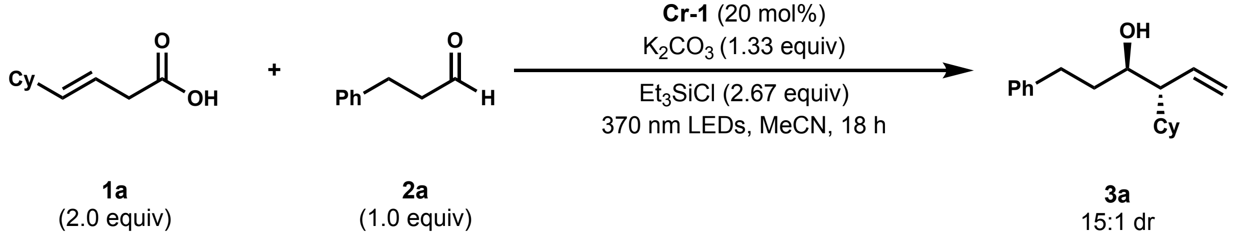

entry	variation from standard conditions	yield[a]
1	none	78%
2	Cs_2CO_3	56%
3	Me_3SiCl	26%
4	1,4-dioxane	0%
5	HCl instead of Et_3SiCl	8%
6	5% **Cr-1**	40%
7	$CrCl_3$ and dtbbpy instead of **Cr-1**	51%
8	**Cr-2** instead of **Cr-1**	23%
9	**Cr-3** instead of **Cr-1**	0%
10	**Cr-4** instead of **Cr-1**	0%
11	340 nm	10%
12	390 nm	29%
13	no light / no light at 80 ºC	0%
14	no **Cr-1**	0%
15	no K_2CO_3	0%
16	no Et_3SiCl	0%

[a]All reactions performed on 0.10 mmol scale with respect to **2a**. Yields and diastereomeric ratios (dr) determined by ^{1}H NMR spectroscopy of the crude reaction mixtures utilizing 1,1,2,2-tetrachloroethane (TCE) as the internal standard.

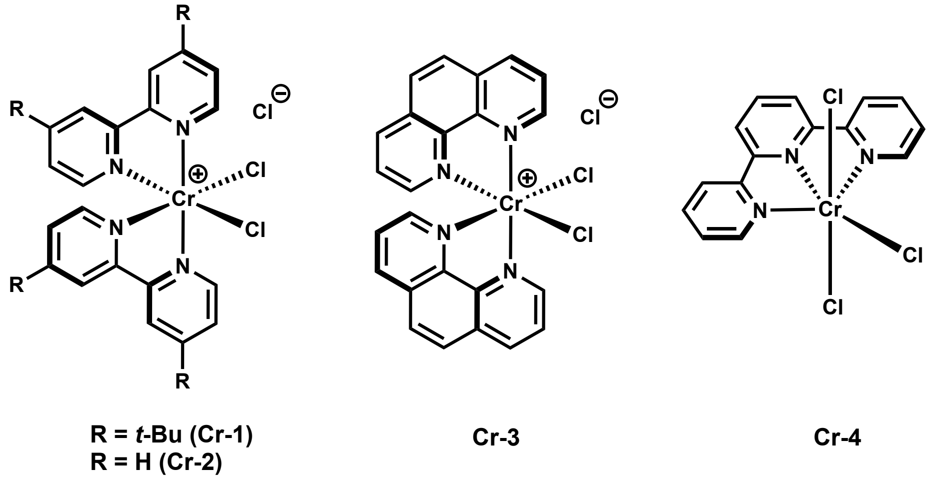

Table 2.2. Base Screen

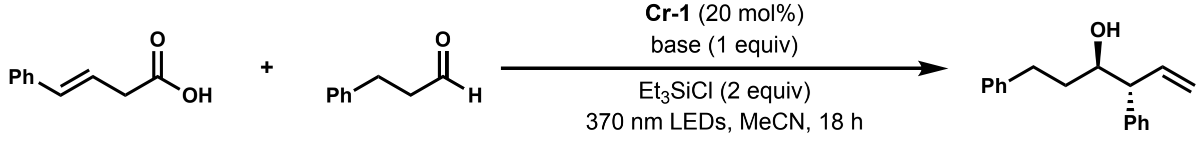

(1.5 equiv)

2a
(1.0 equiv)

3am
>20:1 dr

entry	variation from standard conditions	yield[a]
1	K_2CO_3	50%
2	Li_2CO_3	0%
3	Na_2CO_3	10%
4	Cs_2CO_3	20%
5	$[(n\text{-Bu})_4N]_2CO_3$[b]	7%
6	$KHCO_3$ (2 equiv)[b]	20%
7	KO_2COMe[b]	0%
8	KO_2COMe[b,d]	40%
9	KOTMS	0%
10	K_2HPO_4	0%
11	K_3PO_4	0%
12	KOAc[c]	trace
13	KOBz[c]	0%
14	$K_2C_2O_4$[c]	0%
15	NaOMe	0%
16	NaO*t*-Bu[c]	0%
17	Na_3PO_4[c]	0%
18	MTBD	0%

[a]All reactions performed on 0.10 mmol scale with respect to **2a**. Yields and diastereomeric ratios (dr) determined by ^1H NMR spectroscopy of the crude reaction mixtures utilizing 1,1,2,2-tetrachloroethane (TCE) as the internal standard.

[b]Reaction performed with (*E*)-hex-3-enoic acid.

[c]Reaction performed with 1 equiv of (*E*)-4-phenylbut-3-enoic acid.

[d]Reaction performed with 1 equiv of Et_3SiCl.

Table 2.3. Chlorosilane Screen

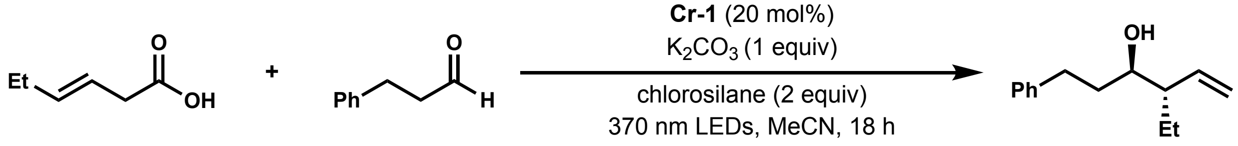

(1.5 equiv) **2a** (1.0 equiv) **SI-1** 5:1 dr

entry	variation from standard conditions	yield[a]
**1**	Et_3SiCl	61%
**2**	Me_3SiCl[b]	30%
**3**	$(i\text{-}Pr)_3SiCl$[b]	16%
**4**	$PhMe_2SiCl$	58%
**5**	$Ph_2MeSiCl$	63%
**6**	Ph_3SiCl	49%
**7**	Me_2SiCl_2 (1 equiv)	53%
**8**	Me_2SiCl_2 (2 equiv)	0%
**9**	Ph_2SiCl_2 (2 equiv)	55%
**10**	$(EtO)_2MeSiCl$	30%
**11**	HCl (4 M in dioxane)	17%
**12**	addition of Et_3SiCl over 1h[b,c]	3%

[a]All reactions performed on 0.10 mmol scale with respect to **2a**. Yields and diastereomeric ratios (dr) determined by ^1H NMR spectroscopy of the crude reaction mixtures utilizing 1,1,2,2-tetrachloroethane (TCE) as the internal standard.

[b]Reaction performed with (*E*)-4-phenylbut-3-enoic acid.

[c]Reaction performed with 1 equiv of acid substrate.

Table 2.4. Chlorosilane and Base Equivalence Screen

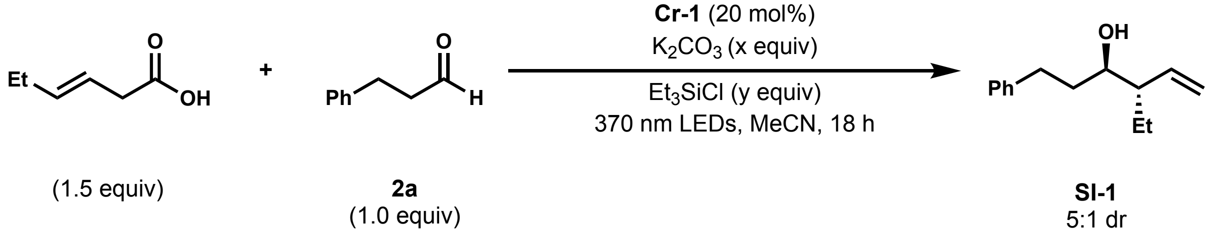

entry	K_2CO_3 equivalence	Et_3SiCl equivalence	yield[a]
**1**	0.25	2	0%
**2**	0.5	2	0%
**3**	0.75	2	43%
**4**	1	2	61%
**5**[b]	1.25	2	0%
**6**[b]	1.5	2	0%
**7**	1	1	7%
**8**[b]	1	1.5	20%
**9**[b]	1	1.75	47%
**10**	1	2.5	17%
**11**	1	3	0%
**12**[c]	1.33	2.66	75%

[a]All reactions performed on 0.10 mmol scale with respect to **2a**. Yields and diastereomeric ratios (dr) determined by ^{1}H NMR spectroscopy of the crude reaction mixtures utilizing 1,1,2,2-tetrachloroethane (TCE) as the internal standard.

[b]Reaction performed with (*E*)-4-phenylbut-3-enoic acid.

[c]Reaction performed for 40 h.

Table 2.5. Solvent Screen

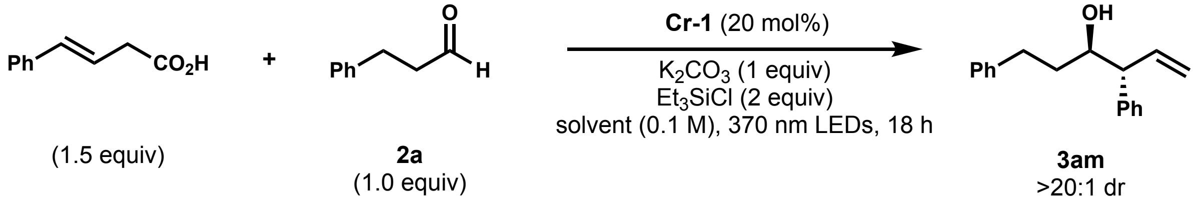

entry	variation from standard conditions	yield[a]
1	MeCN	50%
2	THF	0%
3	MTBE	0%
4	1,4-dioxane	0%
5	DMA	0%
6	DMF	0%
7	DME	0%
8	DCE	0%
9	EtOAc[b]	0%
10	MeCN/DMA (1:1)	0%
11	MeCN/THF (1:1)	0%
12	MeCN/DMF (3:1)[b]	29%
13	MeCN/THF (3:1)[b]	44%
14	MeCN/1,4-dioxane (3:1)	31%
15	MeCN/MTBD (3:1)	10%
16	MeCN/EtOAc (3:1)[b]	34%
17	MeCN/$PhCF_3$ (3:1)[b]	17%
18	MeCN/DME (9:1)[b]	54%
19	CyCN[b]	0%

[a]All reactions performed on 0.10 mmol scale with respect to **2a**. Yields and diastereomeric ratios (dr) determined by ^{1}H NMR spectroscopy of the crude reaction mixtures utilizing 1,1,2,2-tetrachloroethane (TCE) as the internal standard.

[b]Reaction performed with (*E*)-hex-3-enoic acid.

Table 2.6. Additives Screen

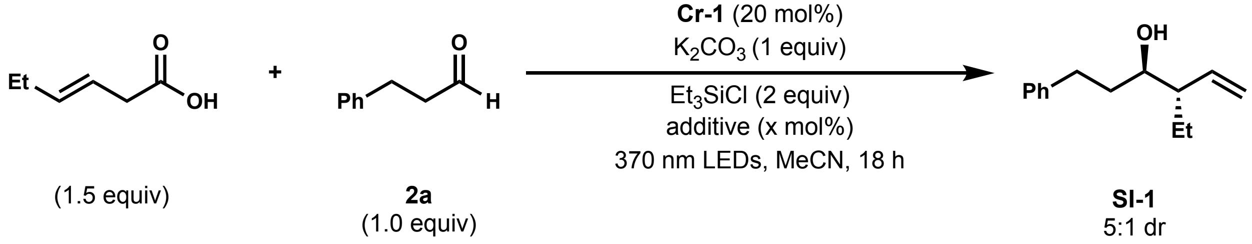

entry	variation from standard conditions	yield[a]
1	none	60%
2	$(n\text{-Bu})_4NCl$ (10 mol%)	53%
3	$(n\text{-Bu})_4NCl$ (50 mol%)	59%
4	$(n\text{-Bu})_4NCl$ (1 equiv)	54%
5	$(n\text{-Bu})_4NCl$ (2 equiv)	39%
6	dtbbpy (40 mol%)[b]	10%
7	$(n\text{-Bu})_4NF$ (1 equiv)	0%
8	$(n\text{-Bu})_4NI$ (50 mol%)	35%
9	$[(n\text{-Bu})_4N]PF_6$ (50 mol%)	29%
10	$MgCl_2$ (50 mol%)	0%
11	$ZnCl_2$ (50 mol%)	0%
12	$AlCl_3$ (50 mol%)	0%
13	KCl (1 equiv)	48%
14	$Bn(Et)_3NCl$ (50 mol%)	12%
15	H_2O (1 equiv)[b]	0%

[a]All reactions performed on 0.10 mmol scale with respect to **2a**. Yields and diastereomeric ratios (dr) determined by ^1H NMR spectroscopy of the crude reaction mixtures utilizing 1,1,2,2-tetrachloroethane (TCE) as the internal standard.

[b]Reaction performed with (*E*)-4-phenylbut-3-enoic acid.

Table 2.7. Other Optimization Experiments

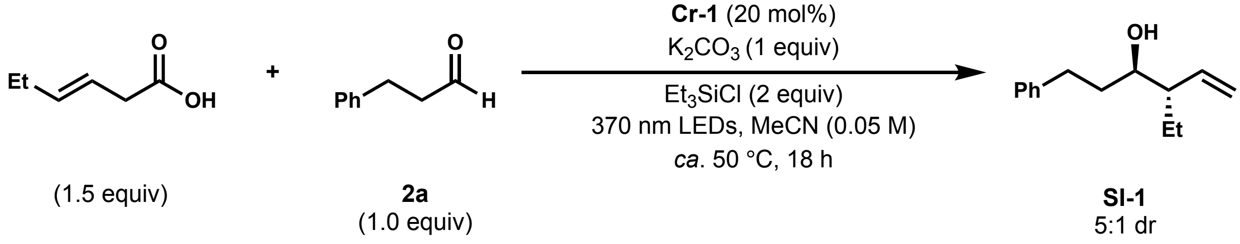

entry	variation from standard conditions	yield[a]
**1**	none	60%
**2**	1 equiv of acid	32%
**3**	low temp. (*ca.* 10 ºC)[b]	0%
**4**	high temp. (*ca.* 80 ºC)	57%
**5**	7 h[b]	3%
**6**	25 h	63%
**7**	0.033 M	54%
**8**	0.1 M	55%
**9**	0.2 M	50%

[a]All reactions performed on 0.10 mmol scale with respect to **2a**. Yields and diastereomeric ratios (dr) determined by ^{1}H NMR spectroscopy of the crude reaction mixtures utilizing 1,1,2,2-tetrachloroethane (TCE) as the internal standard.

[b]Reaction performed with (*E*)-4-phenylbut-3-enoic acid.

3. Synthesis and Characterization of Starting Materials

The following starting materials used in this study were purchased or prepared according to the listed reference:[2-9]

Purchased from Ambeed

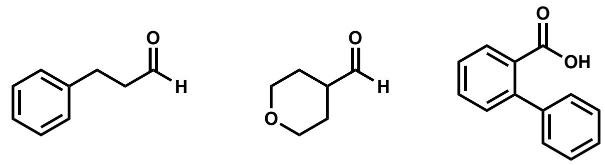

2a 2e 4

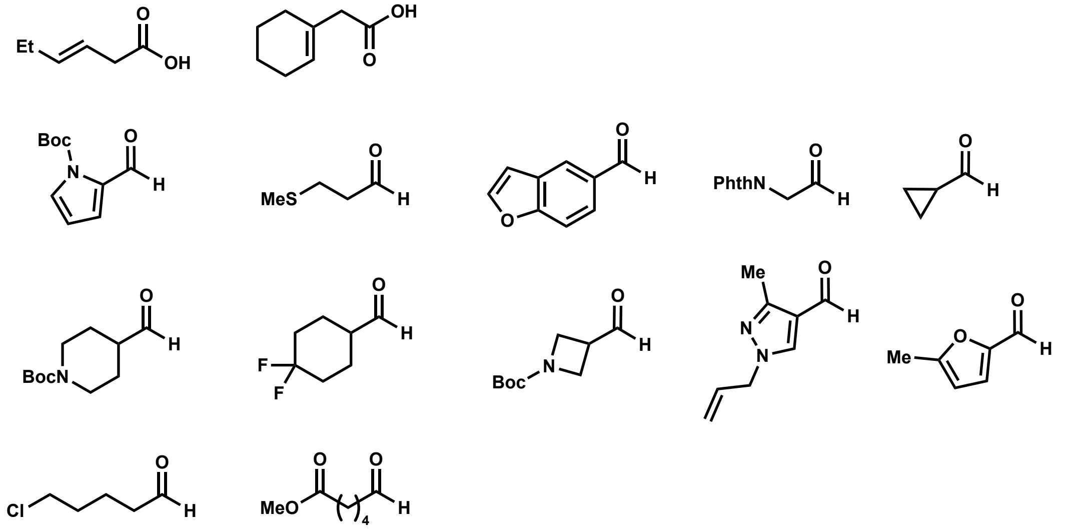

Purchased from Acros Organics

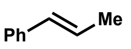

8

Purchased from Millipore Sigma

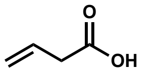

Purchased from Biosynth

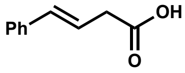

Purchased from Oakwood Chemical

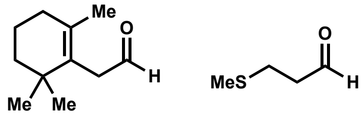

Purchased from Combi Blocks

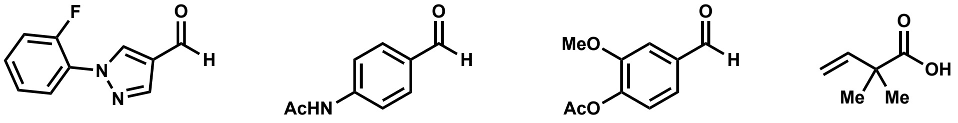

Figure SI-1a: Commercial and previously synthesized starting materials.

Synthesized according to prior literature procedure

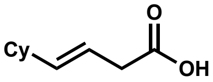

1a
ref.2

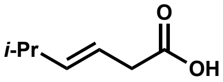

ref. 2

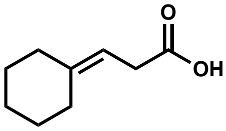

ref. 3

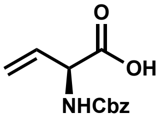

ref. 4

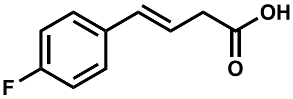

1g
ref. 5

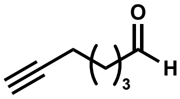

ref. 6

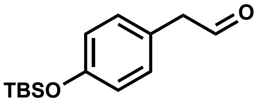

2b
ref. 7

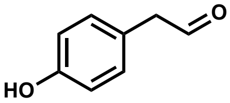

2c
ref. 8

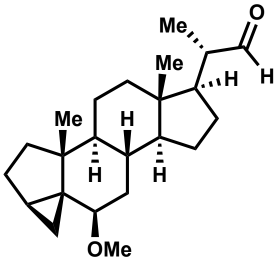

ref. 9

Figure SI-1b: Commercial and previously synthesized starting materials (cont'd.).

3.1. General Procedure B: Synthesis of Bis-Bipyridyl Chromium Complexes

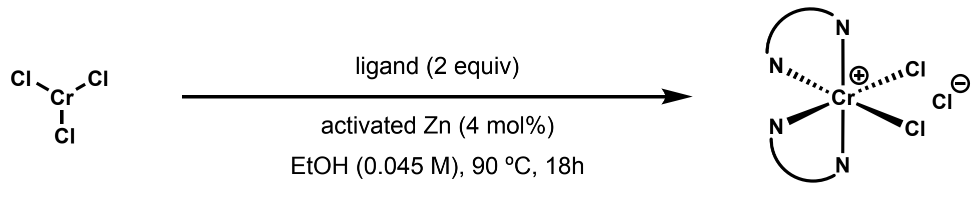

Cr-1–Cr-4

Activation of zinc:

To a 500 mL round-bottom flask equipped with a magnetic stir bar was added Zn dust (9.81 g, 150 mmol, 1.0 equiv) and aq. HCl (1.0 M, 30.0 mL). The reaction mixture was stirred at room temperature for 30 min, then filtered through a Buchner funnel. The solid residue was washed with H_2O (50 mL), acetone (50 mL), MeOH (50 mL), and hexanes (50 mL). The resulting solid was collected into a 500 mL round-bottom flask equipped with a magnetic stir bar and was allowed to stir under high vacuum utilizing a dual-manifold Schlenk line for 48 h to yield activated Zn.

To a flame-dried 100 mL round-bottom flask equipped with a magnetic stir bar was added anhydrous $CrCl_3$ (1.0 equiv), ligand (2.0 equiv), and activated Zn (4 mol%). The flask was equipped with a reflux condenser, sealed with a rubber septum, and evacuated then backfilled with N_2 utilizing a dual manifold Schlenk line three times. To the reaction vessel was added ethanol (0.045 M), and the reaction vessel was placed in a preheated oil bath at 90 °C and allowed to stir. After 18 h, the reaction vessel was removed from the oil bath and allowed to cool to room temperature. Once at room temperature, the reaction mixture was filtered through a 30 mL coarse fritted funnel to remove unreacted $CrCl_3$, and the filtrate was concentrated *in vacuo* with the aid of a rotary evaporator. The solid residue was collected on a 30 mL medium fritted funnel and washed with Et_2O (3 x 20 mL) and dried under vacuum for 24 h. The dried solid was recrystallized via vapor diffusion crystallization to yield the corresponding chromium complex.

[Cr(dtbbpy)$_2$Cl$_2$]Cl (Cr-1)

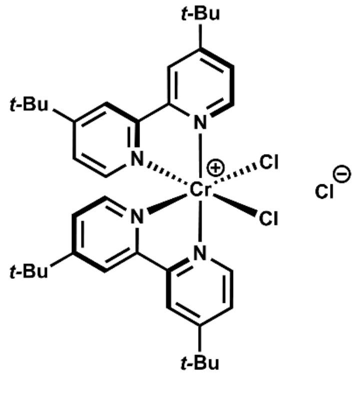

Cr-1

Prepared according to **General Procedure B** from anhydrous $CrCl_3$ (250 mg, 1.58 mmol,

1.0 equiv) and 4,4'-di-*tert*-butyl-2,2'-bipyridine (848 mg, 3.16 mmol, 2.0 equiv). The dried solid was dissolved in a minimal amount of MeCN, and vapor diffusion of Et_2O into this concentrated solution at room temperature led to dark-red crystalline plates suitable for single crystal X-ray diffraction (with one molecule of H_2O in each unit cell) after approximately 24 h. The supernatant was carefully decanted, and the crystals were collected onto a 30 mL medium fritted funnel, washed with Et_2O (3 x 20 mL), and dried under vacuum for 24 h to yield **Cr-1** as a brown solid (810 mg, 74%).

Melting Point: 350 ºC (decomp.).

IR (Diamond-ATR, neat) $\tilde{v}$ (cm^{-1}): 3047, 2969, 2869, 1611, 1541, 1484, 1414, 1367, 1208, 1032, 901, 843.

HRMS (ESI): m/z: $[M-Cl]^+$ calc'd for $C_{36}H_{48}Cl_2CrN_4^+$: 658.2646. Found: 658.2661.

[Cr(bpy)$_2$Cl$_2$]Cl (Cr-2)

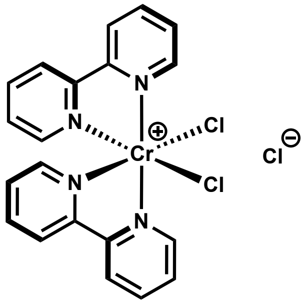

Cr-2

Prepared according to **General Procedure B** from anhydrous $CrCl_3$ (494 mg, 1.58 mmol, 1.0 equiv) and 2,2'-bipyridine (494 mg, 3.16 mmol, 2.0 equiv). The dried solid was dissolved in a minimal amount of MeCN, and vapor diffusion of Et_2O into this concentrated solution at room temperature led to dark-red crystals of **Cr-2** after approximately 24 h. The supernatant was carefully decanted, and the crystals were collected onto a 30 mL medium fritted funnel, washed with Et_2O (3 x 20 mL), and dried under vacuum for 24 h to yield **Cr-2** as a brown solid (56 mg, 8%). Single crystals of **Cr-2** suitable for X-ray diffraction were grown by dissolving **Cr-2** (*ca.* 2 mg) in H_2O (*ca. 50* μL) inside a recrystallization tube (1 mL, Fisherbrand™ Disposable Flint Glass Tubes with Plain End, Catalog No.14-958A). Vapor diffusion of Et_2O into this concentrated solution at room temperature gave dark red crystals of **Cr-2** after approximately 48 h. The unit cell matches that reported for the compound $[(bipy)_2CrCl_2]Cl\cdot 2H_2O$.[10] Analogous compounds of Co(III) and Rh(III) have been reported which crystalize with the same unit cell.[11,12]

[Cr(phen)$_2$Cl$_2$]Cl (Cr-3)

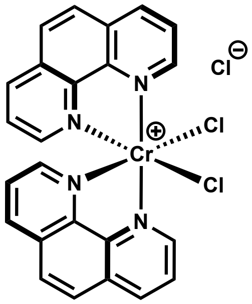

Cr-3

Prepared according to **General Procedure B** from anhydrous $CrCl_3$ (250 mg, 1.58 mmol, 1.0 equiv) and 1,10-phenanthroline (569 mg, 3.16 mmol, 2.0 equiv) to yield **Cr-3** as a dark green solid (78 mg, 10%). Single crystals suitable for X-ray diffraction were grown by dissolving **Cr-3** (*ca.* 2 mg) in a 1:1 solution of EtOH and H_2O (*ca. 50* μL) inside a recrystallization tube (1 mL, Fisherbrand™ Disposable Flint Glass Tubes with Plain End, Catalog No.14-958A). Vapor diffusion of Et_2O into this concentrated solution at −30 ºC gave dark-green crystals of **Cr-3** (with two molecules of H_2O in each unit cell) after approximately 48 h. The crystal structure was found to be analogous to that of [(phen)$_2$CrCl$_2$]Cl·DMF.[13]

Melting Point: 259–261 ºC.

IR (Diamond-ATR, neat) ṽ (cm^{-1}): 3143, 2887, 2756, 1634, 1609, 1585, 1520, 1431, 1374, 1281, 1259, 1215, 1171, 1126, 1096, 892, 850, 838.

HRMS (ESI): m/z: $[M-Cl]^+$ calc'd for $C_{24}H_{16}Cl_2CrN_4^+$: 482.0152. Found: 482.0152.

Cr(terpy)Cl$_3$ (Cr-4)

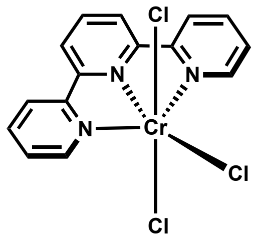

Cr-4

Prepared according to a modified **General Procedure B** from anhydrous $CrCl_3$ (125 mg, 0.79 mmol, 1.0 equiv) and 2,2:6,2-terpyridine (184 mg, 0.79 mmol, 1.0 equiv). The dried solid was dissolved in a minimal amount of 1,1,1,3,3,3-hexafluoro-2-propanol (HFIP), and vapor diffusion of Et_2O into this concentrated solution at room temperature led to dark-

green crystals of **Cr-4** after approximately 24 h. The supernatant was carefully decanted, and the crystals were collected onto a 30 mL medium fritted funnel, washed with Et_2O (3 x 20 mL), and dried under vacuum for 24 h to yield **Cr-4** as a dark-green solid (810 mg, 74%). Single crystals of **Cr-4** suitable for X-ray diffraction were grown by dissolving **Cr-4** (*ca.* 2 mg) in a solution of HFIP (*ca. 50* μL) inside a recrystallization tube (1 mL, Fisherbrand™ Disposable Flint Glass Tubes with Plain End, Catalog No.14-958A). Vapor diffusion of Et_2O into this concentrated solution at room temperature gave dark green crystals of **Cr-4** after approximately 24 h.

Melting Point: > 400 ºC.

IR (Diamond-ATR, neat) $\tilde{\nu}$ (cm^{-1}): 3389, 3072, 1602, 1573, 1477, 1449, 1379, 1285, 1253, 1215, 1175, 1126, 1093, 1023, 890, 840.

HRMS (ESI): m/z: $[M-Cl]^+$ calc'd for $C_{15}H_{11}Cl_2CrN_3^+$: 354.9730. Found: 354.9728.

3.2. Synthesis and Characterization of Chromium Complexes Cr-5–Cr-7

Cr(dtbbpy)$_3$(PF$_6$)$_3$ (Cr-5)

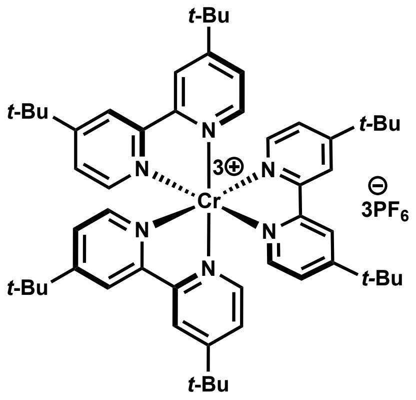

Cr-5

Prepared according to a modified procedure for the preparation of Cr(bpy)$_3$(PF$_6$)$_3$ using 4,4’-di-*tert*-butyl 2,2’-bipyridine instead of 2,2’-bipyridine.[14]

Cr(dtbbpy)Cl$_3$ (Cr-6)

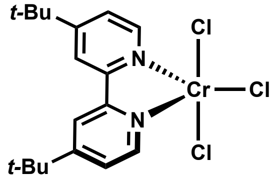

Cr-6

Prepared according to an unmodified literature procedure.[15]

[Cr(dtbbpy)$_2$(2-(3,4-dihydronaphthalen-2-yl)acetic acid)Cl]Cl (Cr-7)

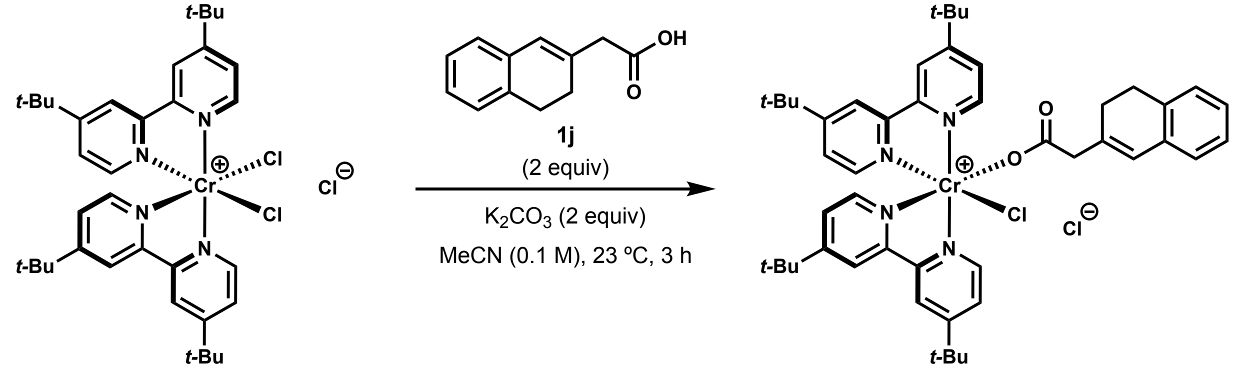

Cr-1 **Cr-7**

Inside a N_2-filled glovebox, to an oven-dried reaction vial (VWR, catalog no. 66011-041) equipped with a magnetic stir bar was added [Cr(dtbbpy)$_2$Cl$_2$]Cl (**Cr-1**) (70 mg, 0.10 mmol, 1.0 equiv), 3,4-dihydronaphthalene-2-carboxylic acid (38 mg, 0.20 mmol, 2.0 equiv), K_2CO_3 (28 mg, 0.20 mmol, 2.0 equiv), and MeCN (1.0 mL, 0.1 M). The reaction mixture was stirred for 3 h at room temperature. The resulting red mixture was filtered through a packed pad of oven-dried Celite (*ca.* 20 mg). Vapor diffusion of Et_2O into this solution gave a microcrystalline red solid, which after decanting the supernatant, washing with Et_2O (3 x 1 mL), and drying under vacuum yielded **Cr-7** (18 mg, 21%). Single crystals of **Cr-7** suitable for X-ray diffraction were grown by transferring *ca.* 50 μL of the filtered red solution into a recrystallization tube (1 mL, Fisherbrand™ Disposable Flint Glass Tubes with Plain End, Catalog No.14-958A). Vapor diffusion of Et_2O into this concentrated solution at −17 °C under N_2 atmosphere gave light red crystals of **Cr-7** (with one molecule of MeCN and 0.5 molecules of Et_2O in each unit cell) after approximately 48 h.

Melting Point: 178 ºC (decomp.).

IR (Diamond-ATR, neat) ṽ (cm^{-1}): 2965, 2876, 2824, 1634, 1616, 1545, 1482, 1410, 1316, 1248, 1201, 1037, 899, 845.

HRMS (ESI): m/z: [M−Cl]$^+$ calc'd for $C_{48}H_{59}ClCrN_4O_2^+$: 810.3726. Found: 810.3733.

3.3. Synthesis and Characterization of Carboxylic Acid Starting Materials

3.3.1. General Procedure C: Purification by Reversed-Phase Chromatography

The crude residue was dry-loaded onto a C18 column (prepacked Biotage® Sfär C18 D - Duo 100 Å 30 µm cartridge (30 g)) and purified using an automated Biotage® Selekt Flash Chromatography System. After the chromatography was complete, the test tubes containing the product were combined into a 500 mL round-bottom flask and the test tubes were each washed with EtOAc (*ca*. 2 x 2 mL). The mixture was concentrated *in vacuo* with the aid of a rotary evaporator to remove the MeCN and EtOAc. Once only the aqueous phase remained, the mixture was transferred to a separatory funnel and diluted with EtOAc (50 mL). The layers were separated, and the aqueous layer was extracted with EtOAc (2 x 50 mL). The combined organic extracts were washed with brine (25 mL), dried over anhydrous Na_2SO_4, filtered and concentrated *in vacuo* with the aid of a rotary evaporator to yield the corresponding product.

3.3.2. General Procedure D: Synthesis of β,γ-unsaturated Carboxylic Acids SI-2–SI-3, 1b, 1e, 1f

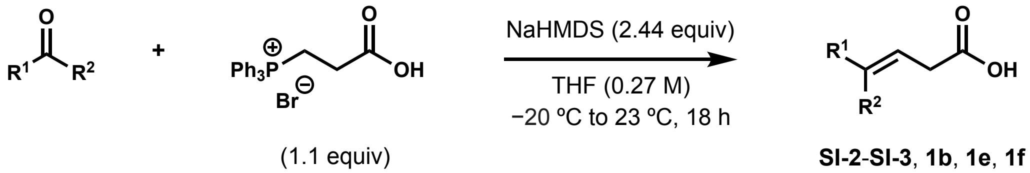

To a flame-dried 100 mL Schlenk flask equipped with a magnetic stir bar was added (2-carboxyethyl)triphenylphosphonium bromide (1.1 equiv). The reaction vessel was sealed with a rubber septum and evacuated then backfilled with N_2 utilizing a dual manifold Schlenk line. This process was repeated three times. Then, anhydrous THF (0.45 M) was added via syringe and the reaction mixture was cooled to −20 ºC. To the colorless suspension was added NaHMDS (2.5 M in THF, 2.44 equiv) dropwise via syringe over 5 min. The resulting orange solution was allowed to stir at −20 ºC. After 30 min, a solution of the ketone (2.0 M in THF, 1.0 equiv) was added dropwise via syringe at −20 ºC (final concentration: 0.27 M). The resulting mixture was allowed to warm up to room temperature and stirred for 18 h. After this time, the reaction mixture was quenched with H_2O (20 mL), transferred to a separatory funnel, and washed with CH_2Cl_2 (2 x 20 mL). The aqueous layer was acidified to a pH = *ca.* 1 with aq. HCl (1 M) and extracted with EtOAc (3 x 20 mL). The combined organic extracts were washed with brine, dried over anhydrous Na_2SO_4, and concentrated *in vacuo* with the aid of a rotary evaporator. The residue was purified according to **General Procedure C** to yield the desired acid product.

3-(2-(*tert*-Butoxycarbonyl)-2-azaspiro[3.3]heptan-6-ylidene)propanoic acid (SI-2)

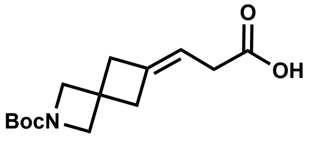

SI-2

Prepared according to **General Procedure D** from *tert*-butyl 6-oxo-2-azaspiro[3.3]heptane-2-carboxylate (1.06 g, 5.00 mmol). The residue was purified according to **General Procedure C** (gradient elution: 15% MeCN in H_2O to 25% MeCN in H_2O) to yield **SI-2** as a colorless solid (255 mg, 19%).

^{1}H NMR (600 MHz, $CDCl_3$): δ 5.34–5.29 (m, 1H), 3.92 (s, 4H), 2.94 (d, *J* = 7.1 Hz, 2H), 2.85 (s, 2H), 2.82 (s, 2H), 1.42 (s, 9H).

^{13}C NMR (151 MHz, $CDCl_3$): δ 177.4, 156.5, 137.5, 113.7, 79.7, 61.4, 42.1, 40.4, 33.7, 33.4, 28.5.

IR (Diamond-ATR, neat) ṽ (cm^{-1}): 3081, 3001, 2976, 2948, 2906, 2873, 1730, 1646, 1477, 1430, 1369, 1294, 1252, 1166, 1102, 850.

HRMS (ESI): m/z: [M+H]$^+$ calc'd for $C_{14}H_{22}NO_4^{+}$: 268.1543. Found: 268.1541.

4-(1,4-Dioxaspiro[4.5]decan-8-yl)but-3-enoic acid (SI-3)

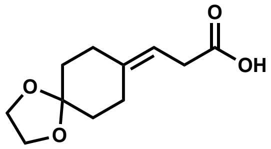

SI-3

Prepared according to **General Procedure D** from 1,4-dioxaspiro[4.5]decan-8-one (469 mg, 3.00 mmol). The residue was purified according to **General Procedure C** (gradient elution: 10% MeCN in H_2O to 40% MeCN in H_2O) to yield **SI-3** as a colorless oil (284 mg, 45%).

^{1}H NMR (600 MHz, $CDCl_3$): δ 5.32 (t, *J* = 7.1 Hz, 1H), 3.96 (s, 4H), 3.10 (d, *J* = 7.2 Hz, 2H), 2.28 (dt, *J* = 6.5, 5.7 Hz, 4H), 1.68 (dt, *J* = 15.7, 6.7 Hz, 4H).

^{13}C NMR (151 MHz, $CDCl_3$): δ 178.4, 141.4, 113.5, 108.9, 64.5, 36.1, 35.3, 33.5, 33.1, 25.5.

IR (Diamond-ATR, neat) ṽ (cm^{-1}): 3029, 2956, 2931, 2904, 2884, 2845, 1699, 1427, 1399, 1330, 1299, 1277, 1216, 1127, 1091, 1069, 1033, 961, 941, 908, 816.

HRMS (ESI): m/z: [M+H]$^+$ calc'd for $C_{11}H_{17}O_4^{+}$: 213.1121. Found: 213.1118.

(*E*)-4,8-Dimethylnona-3,7-dienoic acid (1b)

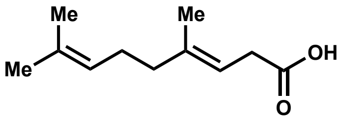

1b

Prepared according to **General Procedure D** from 6-methylhept-5-en-2-one (469 mg, 3.00 mmol). The residue was purified according to **General Procedure C** (gradient elution: 0% MeCN in H_2O to 100% MeCN in H_2O) to yield **1b** as a colorless oil (105 mg, 22%).

All spectroscopic data for **1l** was consistent with that which was previously reported.[16]

(*E*)-4-Cyclopropylbut-3-enoic acid (1e)

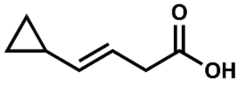

1e

Prepared according to **General Procedure D** from cyclopropanecarbaldehyde (224 µL, 3.00 mmol). The residue was purified according to **General Procedure C** (gradient elution: 0% MeCN in H_2O to 25% MeCN in H_2O) to yield **1e** as a colorless oil and 4:1 mixture of *E/Z* isomers (78 mg, 21%). The configuration of the major olefin isomer was assigned to be *E* based on the major *J* coupling constant of the vinyl protons (*ca.* 15 Hz).

^{1}H NMR (600 MHz, $CDCl_3$, *E* isomer): δ 5.58 (dt, *J* = 15.3, 7.1 Hz, 1H), 5.12 (ddt, *J* = 15.2, 8.7, 1.4 Hz, 1H), 3.06 (dd, *J* = 7.2, 1.3 Hz, 2H), 1.44–1.37 (m, 1H), 0.72–0.68 (m, 2H), 0.38–0.35 (m, 2H).

^{13}C NMR (151 MHz, $CDCl_3$, *E* isomer): δ 178.8, 139.1, 118.4, 37.8, 13.8, 6.7.

IR (Diamond-ATR, neat) ṽ (cm^{-1}): 3079, 3006, 2920, 1704, 1407, 1287, 1220, 1188, 1138, 964, 856, 812.

HRMS (ESI): m/z: $[M+H]^+$ calc'd for $C_7H_{11}O_2^+$: 127.0754. Found: 127.0751.

(*E*)-4-(2-Phenylcyclopropyl)but-3-enoic acid (1f)

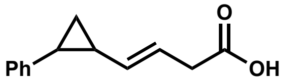

1f

Prepared according to **General Procedure D** from 2-phenylcyclopropane-1-carbaldehyde (520 mg, 3.56 mmol). The residue was purified according to **General Procedure C** (gradient elution: 20% MeCN in H_2O to 60% MeCN in H_2O) to yield **1f** as a colorless oil and 1:1 mixture of *E/Z* isomers (70 mg, 10%). The configuration of the olefin isomers were assigned based on the major *J* coupling constant of the vinyl protons (*ca.* 15 Hz for *E* isomer, *ca.* 11 Hz for *Z* isomer).

^{1}H NMR (600 MHz, $CDCl_3$, mixture of *E/Z* isomers): δ 7.30–7.25 (m, 2H), 7.20–7.14 (m, 1H), 7.11–7.05 (m, 2H), 5.64 (dt, *J* = 15.3, 7.1 Hz, 1H, *E* isomer), 5.56 (dt, *J* = 10.7, 7.2z Hz, 1H, *Z* isomer), 5.36–5.16 (m, 1H), 3.33–3.09 (m, 2H), 1.99–1.89 (m, 1H), 1.79–1.68 (m, 1H), 1.32–1.18 (m, 1H), 1.13–1.06 (m, 1H).

^{13}C NMR (151 MHz, $CDCl_3$, mixture of *E/Z* isomers): δ 178.5, 178.4, 142.3, 142.0, 137.2, 136.3, 128.5, 128.5, 125.9, 125.9, 125.8, 119.5, 119.3, 37.8, 33.2, 26.4, 25.4, 25.2, 22.7, 17.3, 16.8.

IR (Diamond-ATR, neat) ṽ (cm^{-1}): 3066, 3025, 2923, 1704, 1407, 1293, 1217, 1179, 964, 938.

HRMS (ESI): m/z: $[M+H]^+$ calc'd for $C_{13}H_{15}O_2^+$: 203.1067. Found: 203.1062.

3.3.3. General Procedure E: Synthesis and Characterization of Carboxylic Acids SI-6 and 1c-K

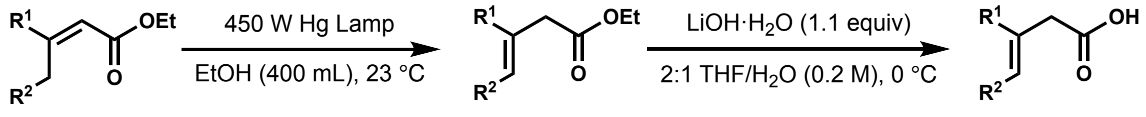

To a 500 mL reaction vessel fitted with a quartz water-cooled immersion well and equipped with a magnetic stir bar was added the corresponding α,β-unsaturated esters (1 equiv). The reaction vessel was sealed with a rubber septum and evacuated then backfilled with N_2 utilizing a dual manifold Schlenk line. This process was repeated three times. Then EtOH (400 mL) was added via syringe, and the solution was sonication degassed while bubbling N_2 for 30 min. Then a mercury lamp (Hanovia, 450 W medium pressure) was placed inside the immersion well, and the reaction mixture was irradiated while cooled with circulating room temperature water. After the reaction was determined complete (*ca.* 1-5 h), the lamp was switched off and the reaction mixture was concentrated *in vacuo* with the aid of a rotary evaporator. The resulting crude residue was purified by silica gel column chromatography to yield the β,γ-unsaturated esters **SI-4**–**SI-5**.

To a 50 mL round-bottom flask equipped with a magnetic stir bar was added **SI-4**–**SI-5** (1.0 equiv). To the flask was then added THF and H_2O (2:1 v/v, 0.2 M total), and the flask was placed in a 0 °C ice-water bath. Then $LiOH\cdot H_2O$ (1.1 equiv) was added in one portion, and the flask was lightly capped with a plastic cap and left to stir in the ice-water bath. After the reaction was determined complete through TLC analysis, the reaction mixture was diluted with sat. aq. $NaHCO_3$ (25 mL) and transferred to a separatory funnel with EtOAc (10 mL) and H_2O (10 mL). The aqueous layer was extracted with EtOAc (3 x 5 mL) and the organic extracts were discarded. The aqueous layer was acidified to a pH = *ca.* 1 with aq. HCl (1 M) and extracted with EtOAc (3 x 20 mL). The combined organic extracts were washed with brine (10 mL), dried over anhydrous Na_2SO_4, and concentrated *in vacuo* with the aid of a rotary evaporator to give the β-γ unsaturated acids **1h** and **1k**. The crude materials were used without further purification.

Ethyl 2-(1-benzoyl-1,2,3,6-tetrahydropyridin-4-yl)acetate (SI-4)

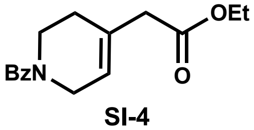

SI-4

Prepared according to **General Procedure E** using ethyl 2-(1-benzoylpiperidin-4-ylidene)acetate (2.00 g, 7.33 mmol) and irradiating for 3.5 hours. The resulting crude residue was purified by silica gel column chromatography (gradient elution: hexanes to

50% EtOAc in hexanes) to give ethyl 2-(1-benzoyl-1,2,3,6-tetrahydropyridin-4-yl)acetate (**SI-4**) as a yellow oil (981 mg, 49%).

^{1}H NMR (600 MHz, $CDCl_3$, mixture of rotamers): δ 7.43–7.32 (m, 5H), 5.76–5.30 (m, 1H), 4.22 (br s, 1H), 4.14 (q, *J* = 7.2 Hz, 2H), 3.91 (br s, 1H), 3.87 (br s, 1H), 3.49 (br s, 1H), 3.09–2.96 (m, 2H), 2.32–2.08 (m, 2H), 1.27 (t, *J* = 7.2 Hz, 3H).

^{13}C NMR (151 MHz, $CDCl_3$, mixture of rotamers): δ 171.2, 170.9, 170.5, 136.3, 129.8, 128.6, 127.2, 126.9, 122.7, 121.8, 60.9, 47.5, 44.6, 42.6, 42.4, 39.4, 29.2, 28.2, 14.4.

All spectroscopic data for **SI-4** was consistent with that which was previously reported.[17]

Ethyl 2-(2,2-dimethyl-4*H*-1,3-dioxin-5-yl)acetate (SI-5)

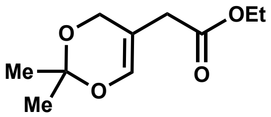

SI-5

Prepared according to **General Procedure E** using ethyl 2-(2,2-dimethyl-1,3-dioxan-5-ylidene)acetate (2.00 g, 10.00 mmol) and irradiating for 1 hour. The resulting crude residue was purified by flash column chromatography on silica gel (gradient elution: hexanes to 25% EtOAc in hexane) to give ethyl 2-(2,2-dimethyl-4*H*-1,3-dioxin-5-yl)acetate (**SI-5**) as a colorless oil (1.23 g, 61%).

^{1}H NMR (600 MHz, $CDCl_3$): δ 6.33 (s, 1H), 4.21 (s, 2H), 4.14 (q, *J* = 7.1 Hz, 2H), 2.89 (s, 2H), 1.47 (s, 6H), 1.26 (t, *J* = 7.2 Hz, 3H).

^{13}C NMR (151 MHz, $CDCl_3$): δ 171.5, 139.0, 105.0, 98.7, 61.5, 61.0, 35.5, 24.3, 14.3.

HRMS (ESI): m/z: $[M+H]^+$ calc'd for $C_{10}H_{17}O_4^+$: 201.1121. Found: 201.1116.

2-(1-Benzoyl-1,2,3,6-tetrahydropyridin-4-yl)acetic acid (SI-6)

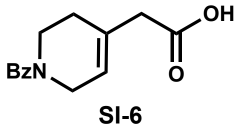

SI-6

Prepared according to **General Procedure E** using ethyl 2-(1-benzoyl-1,2,3,6-tetrahydropyridin-4-yl)acetate (**SI-4**) (880 mg, 3.22 mmol) to give 2-(1-benzoyl-1,2,3,6-tetrahydropyridin-4-yl)acetic acid (**SI-6**) as a colorless solid (684 mg, 87%).

^{1}H NMR (600 MHz, $CDCl_3$, mixture of rotamers): δ 7.45–7.33 (m, 5H), 5.73–5.34 (m, 1H), 4.22 (br s, 1H), 4.00–3.76 (m, 2H), 3.48 (br s, 1H), 3.14–2.95 (m, 2H), 2.34–2.13 (m, 2H).

^{13}**C NMR** (151 MHz, $CDCl_3$, mixture of rotamers): δ 175.9, 171.2, 170.8, 135.8, 131.0, 130.0, 129.5, 128.6, 127.3, 127.0, 122.9, 122.1, 47.5, 44.6, 42.5, 42.3, 39.6, 29.1, 28.1.

IR (Diamond-ATR, neat) ṽ (cm^{-1}): 3063, 3012, 2971, 2892, 2683, 1723, 1591, 1559, 1473, 1458, 1321, 1255, 1192, 1173, 1154, 977, 926.

HRMS (ESI): m/z: $[M+H]^+$ calc'd for $C_{14}H_{16}NO_3^+$: 246.1125. Found: 246.1124.

Potassium 2-(2,2-dimethyl-4*H*-1,3-dioxin-5-yl)acetate (1c-K)

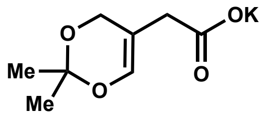

1c-K

Prepared according to a modified **General Procedure E** using ethyl 2-(2,2-dimethyl-4*H*-1,3-dioxin-5-yl)acetate (**SI-5**) (1.23 g, 6.13 mmol, 1.0 equiv) and potassium hydroxide (344 mg, 6.13 mmol, 1.0 equiv). The crude reaction mixture was concentrated *in vacuo* with the aid of a rotary evaporator, washed with EtOAc (3 x 5 mL), and lyophilized to give potassium 2-(2,2-dimethyl-4*H*-1,3-dioxin-5-yl)acetate (**1c-K**) as a colorless solid (1.00 g, 78%).

^{1}**H NMR** (600 MHz, D_2O): δ 6.35 (br s, 1H), 4.21 (s, 2H), 2.79 (s, 2H), 1.49 (s, 6H).

^{13}**C NMR** (151 MHz, D_2O): δ 180.0, 136.6, 108.7, 99.2, 61.2, 37.8, 23.2.

IR (Diamond-ATR, neat) ṽ (cm^{-1}): 3426, 3349, 3007, 2988, 2937, 2848, 1702, 1673, 1558, 1421, 1385, 1376, 1365, 1232, 1121, 829.

HRMS (ESI): m/z: $[M-K+2H]^+$ calc'd for $C_8H_{13}O_4^+$: 173.0808. Found: 173.0806.

3.3.4. Synthesis and Characterization of Carboxylic Acids SI-7–SI-10, and 1d

(*E*)-4-(Tetrahydro-2*H*-thiopyran-4-yl)but-3-enoic acid (SI-7)

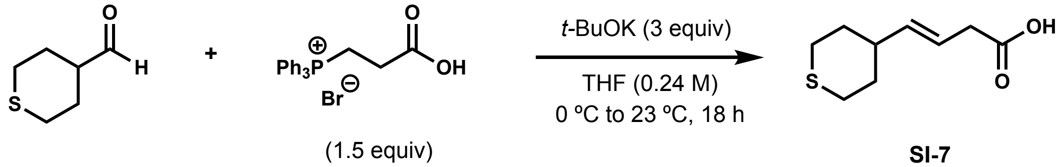

SI-7

To a flame-dried round-bottom flask equipped with a magnetic stir bar was added (2-carboxyethyl)-triphenylphosphonium bromide (981 mg, 2.36 mmol, 1.5 equiv). The reaction vessel was sealed with a rubber septum and evacuated then backfilled with N_2 utilizing a dual manifold Schlenk line. This process was repeated three times. To the flask was added THF (3.8 mL) and tetrahydro-2*H*-thiopyran-4-carbaldehyde (205 mg, 1.57 mmol, 1.0 equiv). The reaction flask was placed in an ice-water bath and allowed to stir at 0 °C. To a separate flame-dried 25 mL round-bottom flask was added KO*t*-Bu (530 mg, 4.72 mmol, 3 equiv). The flask was sealed with a rubber septum and evacuated then backfilled with N_2 utilizing a dual manifold Schlenk line. This process was repeated three times. To this flask was added THF (2.8 mL, 1.69 M), and the flask was sonicated until the complete dissolution of KO*t*-Bu was observed *(ca.* 30 s). The resulting KO*t*-Bu solution was added dropwise over 5 min to the stirred reaction mixture at 0 °C (final concentration: 0.24 M). The ice-water bath was removed, and the reaction mixture was allowed to warm to room temperature. After the reaction had stirred for 18 h, the reaction mixture was diluted with aq. NaOH (2.0 M) to a pH = *ca.* 10–12. Then, the reaction mixture was transferred to a separatory funnel and extracted with EtOAc (2 x 20 mL). The aqueous layer was acidified to a pH = *ca.* 1 with aq. HCl (1 M) and extracted with EtOAc (3 x 20 mL). The combined organic extracts were washed with brine, dried over anhydrous Na_2SO_4, and concentrated *in vacuo* with the aid of a rotary evaporator. The residue was purified according to **General Procedure C** (gradient elution: 20% MeCN in H_2O to 35% MeCN in H_2O) to yield **SI-7** as a colorless oil and 5:1 mixture of *E*/*Z* isomers (97 mg, 33%). The configuration of the major olefin isomer was assigned to be *E* based on analogy with other β,γ-unsaturated carboxylic acids synthesized from wittig olefination (e.g., **1e** and **1f**).

^{1}H NMR (600 MHz, $CDCl_3$, *E* isomer): δ 5.52–5.44 (m, 2H), 3.14–3.12 (m, 2H), 2.71 (t, *J* = 12.7 Hz, 2H), 2.60 (dt, *J* = 13.9, 3.9 Hz, 2H), 2.31–2.24 (m, 1H), 1.90 (dq, *J* = 13.7, 2.5 Hz, 2H), 1.52 (dtd, *J* = 13.0, 12.0, 3.1 Hz, 2H).

^{13}C NMR (151 MHz, $CDCl_3$, *E* isomer): δ 177.7, 138.5, 119.2, 40.2, 37.8, 36.2, 33.5, 32.9, 28.2.

IR (Diamond-ATR, neat) ṽ (cm^{-1}): 3076, 3023, 2920, 2904, 2840, 1707, 1427, 1297, 1219, 1188, 1161, 969, 938, 908.
HRMS (ESI): m/z: $[M+H]^+$ calc'd for $C_9H_{15}O_2S^+$: 187.0787. Found: 187.0784.

(±)-2-(Benzyloxy)but-3-enoic acid (SI-8)

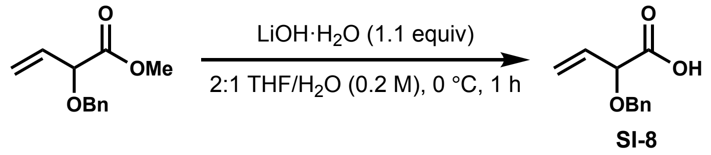

To a 25 mL round-bottom flask equipped with a magnetic stir bar was added methyl 2-(benzyloxy)but-3-enoate (417 mg, 2.02 mmol, 1.0 equiv). To the flask was then added THF (7 mL) and H_2O (3.5 mL, 0.2 M total), and the flask was placed in a 0 °C ice-water bath. Then LiOH·H_2O (93 mg, 2.22 mmol, 1.1 equiv) was added in one portion, and the flask was lightly capped with a plastic cap and left to stir in the ice-water bath. After 1 h, the reaction mixture was diluted with sat. aq. $NaHCO_3$ (10 mL) and transferred to a separatory funnel with EtOAc (10 mL) and H_2O (10 mL). The aqueous layer was extracted with EtOAc (3 x 5 mL) and the organic extracts were discarded. The aqueous layer was acidified to a pH = *ca.* 1 with aq. HCl (1 M) and extracted with EtOAc (3 x 10 mL). The combined organic extracts were washed with brine (10 mL), dried over anhydrous Na_2SO_4, and concentrated *in vacuo* with the aid of a rotary evaporator to give (±)-2-(benzyloxy)but-3-enoic acid (**SI-8**) as a colorless solid (321 mg, 83%). The crude material was used without further purification.

^{1}H NMR (600 MHz, $CDCl_3$): δ 7.39–7.31 (m, 5H), 5.93 (ddd, *J* = 17.1, 10.3, 6.4 Hz, 1H), 5.55 (d, *J* = 17.4 Hz, 1H), 5.43 (d, *J* = 10.3 Hz, 1H), 4.65 (s, 2H), 4.48 (d, *J* = 6.3 Hz, 1H).

^{13}C NMR (151 MHz, $CDCl_3$): δ 174.9, 136.7, 131.9, 128.8, 128.4, 128.2, 120.4, 78.3, 71.7.

IR (Diamond-ATR, neat) ṽ (cm^{-1}): 3066, 3028, 2965, 2879, 1701, 1420, 1274, 1255, 1217, 1144, 1059, 1027, 998, 929, 903.

HRMS (ESI): m/z: $[M+H]^+$ calc'd for $C_{11}H_{13}O_3^+$: 193.0859. Found: 193.0856.

2-(Benzoyloxy)but-3-enoic acid (SI-9)

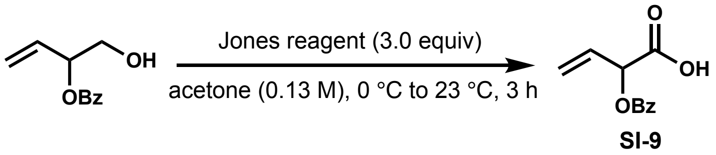

Preparation of Jones reagent solution:

CrO_3 (1.50 g, 15.0 mmol, 3.0 equiv) was added to a test tube and dissolved with H_2O (5 mL). The test tube was placed in a 0 °C ice-water bath, and concentrated H_2SO_4 (1.60 mL, 30.0 mmol, 6.0 equiv) was added dropwise over 2 min. The solution was thoroughly mixed by shaking to give Jones reagent (*ca.* 2.0 M).

To a 100 mL round-bottom flask equipped with a magnetic stir bar was added 1-hydroxybut-3-en-2-yl benzoate (961 mg, 5.00 mmol, 1.0 equiv) and diluted with acetone (38 mL, 0.13 M). The flask was placed in a 0 °C ice-water bath, and all the Jones reagent solution was added dropwise over 5 min. The flask was then sealed with a rubber septum and allowed to warm to room temperature. After 3 h, the reaction was quenched with *i*-PrOH (5 mL), filtered through a pad of packed Celite (30 g) packed in a glass fritted funnel, and concentrated *in vacuo* with the aid of a rotary evaporator. The residue was then diluted with H_2O (20 mL) and EtOAc (20 mL), transferred to a separatory funnel and extracted with EtOAc (3 x 20 mL). The combined organic extracts were washed with brine, dried over anhydrous Na_2SO_4, and concentrated *in vacuo* with the aid of a rotary evaporator. The residue was purified according to **General Procedure C** (gradient elution: 10% MeCN in H_2O to 50% MeCN in H_2O) to yield 2-(benzoyloxy)but-3-enoic acid (**SI-9**) as a colorless solid (454 mg, 44%).

^{1}H NMR (600 MHz, $CDCl_3$): δ 8.12–8.10 (m, 2H), 7.61–7.58 (m, 1H), 7.47 (t, *J* = 7.8 Hz, 2H), 6.11 (ddd, *J* = 16.7, 10.5, 5.9 Hz, 1H), 5.77 (dt, *J* = 5.9, 1.5 Hz, 1H), 5.67 (dd, *J* = 17.2, 1.5 Hz, 1H), 5.48 (dd, *J* = 10.5, 1.2 Hz, 1H).

^{13}C NMR (151 MHz, $CDCl_3$): δ 173.3, 165.7, 133.7, 130.1, 129.7, 129.2, 128.7, 120.5, 73.0.

IR (Diamond-ATR, neat) ṽ (cm^{-1}): 2984, 2844, 2563, 1733, 1704, 1302, 1280, 1258, 1233, 1109, 1097, 1071, 1027, 989, 932, 900.

HRMS (ESI): m/z: $[M+H]^+$ calc'd for $C_{11}H_{11}O_4^+$: 207.0652. Found: 207.0650.

(*E*)-8-Methoxy-8-oxooct-3-enoic acid (SI-10)

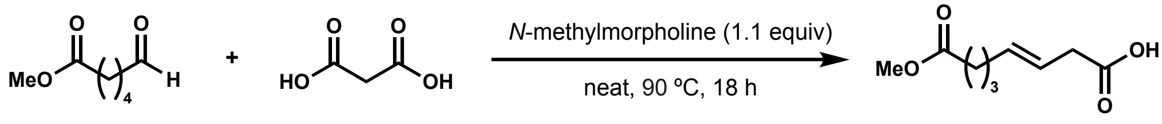

(1.1 equiv)

SI-10

To an oven-dried 20 mL microwave vial equipped with a magnetic stir bar was added malonic acid (1.15 g, 11.0 mmol, 1.1 equiv). The vial was sealed with a crimp cap (Chemglass Life Sciences, catalog no. CG-4920-10) and evacuated then backfilled with N_2 utilizing a dual manifold Schlenk line. This process was repeated three times. Then, *N*-methylmorpholine (1.2 mL, 11.0 mmol, 1.1 equiv) and methyl-6-oxohexanoate (1.44 mL, 10.0 mmol, 1.0 equiv) were added. The reaction vial was placed in an oil bath pre-heated to 90 ºC and allowed to stir. After 18 h, the reaction mixture was diluted with aq. NaOH (2.0 M) to a pH = *ca.* 10–12. Then, the reaction mixture was transferred to a separatory funnel, extracted with EtOAc (2 x 20 mL), and the organic extracts were

discarded. The aqueous extracts were acidified to a pH = *ca.* 1 with aq. HCl (1 M) and extracted with EtOAc (3 x 20 mL). The combined organic extracts were washed with brine, dried over anhydrous Na_2SO_4, and concentrated *in vacuo* with the aid of a rotary evaporator to yield **SI-10** as a yellow oil (1.35 g, 73%). The crude product was utilized without further purification.

^{1}H NMR (600 MHz, $CDCl_3$): δ 5.58–5.51 (m, 2H), 3.65 (s, 3H), 3.07 (d, *J* = 5.3 Hz, 2H), 2.30 (t, *J* = 7.4 Hz, 2H), 2.09–2.06 (m, 2H),1.71 (p, *J* = 7.4 Hz, 2H).

^{13}C NMR (151 MHz, $CDCl_3$): δ 178.3, 174.2, 134.1, 122.1, 51.7, 37.8, 33.4, 31.9, 24.3.

IR (Diamond-ATR, neat) ṽ (cm^{-1}): 3170, 3047, 2952, 2844, 1736, 1708, 1439, 1416, 1290, 1220, 1201, 1173, 1147, 973.

HRMS (ESI): m/z: $[M+H]^+$ calc'd for $C_9H_{15}O_4^+$: 187.0965. Found: 187.0962.

2-(3,4-Dihydronaphthalen-2-yl)acetic acid (1d)

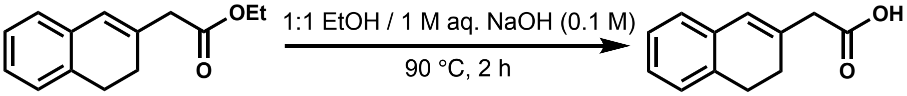

1d

To a 250 mL round-bottom flask equipped with a magnetic stir bar was added ethyl 2-(3,4-dihydronaphthalen-2-yl)acetate (1.94 g, 8.97 mmol, 1.0 equiv) and diluted with EtOH (45 mL) and aq. NaOH (1 M, 45 mL, 0.1 M total). Then, a water-cooled reflux condenser was attached to the flask, and the reaction apparatus was placed in an oil bath preheated to 90 °C and allowed to stir. After 2 h, the reaction apparatus was removed from the oil bath and allowed to cool to room temperature. Then, the volatiles were removed *in vacuo* with the aid of a rotary evaporator, and the resulting residue was transferred to a separatory funnel with H_2O (20 mL) and EtOAc (20 mL). The aqueous layer was extracted with EtOAc (3 x 20 mL) and the organic extracts were discarded. The aqueous extracts were acidified to a pH = *ca.* 1 with aq. HCl (1 M), and extracted with EtOAc (3 x 25 mL). The combined organic extracts were washed with brine, dried over anhydrous Na_2SO_4, and concentrated *in vacuo* with the aid of a rotary evaporator to give 2-(3,4-dihydronaphthalen-2-yl)acetic acid (**1d**) as an off-white solid (1.50 g, 89%). The crude material was used without further purification.

^{1}H NMR (600 MHz, $CDCl_3$): δ 7.15–7.09 (m, 3H), 7.02–7.00 (m, 1H), 6.38 (s, 1H), 3.26 (s, 2H), 2.86 (t, *J* = 8.1 Hz, 2H), 2.38 (t, *J* = 8.0 Hz, 2H).

^{13}C NMR (151 MHz, $CDCl_3$): δ 176.8, 134.6, 134.1, 133.1, 127.4, 127.1, 126.9, 126.6, 126.1, 42.6, 28.1, 27.3.

All spectroscopic data for **1d** was consistent with that which was previously reported.[18]

3.4. Synthesis and Characterization of Aldehydes SI-11–SI-12, and 2d

6-Hydroxy-6,6-diphenylhexanal (SI-11)

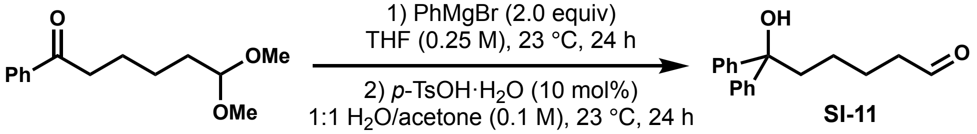

To a flame-dried 25 mL round-bottom flask equipped with a magnetic stir bar was added 6,6-dimethoxy-1-phenylhexan-1-one (473 mg, 2.00 mmol, 1.0 equiv). The reaction vessel was sealed with a rubber septum and evacuated then backfilled with N_2 utilizing a dual manifold Schlenk line. This process was repeated three times. Then, THF (8 mL, 0.25 M) was added to the reaction flask followed by PhMgBr (4.0 mL, 4.00 mmol, 2.0 equiv, 1.0 M in THF) dropwise over 2 min at room temperature. Then, the reaction mixture was allowed to stir at room temperature. After 24 h, the rubber septum was removed and the mixture was diluted with sat. aq. NH_4Cl (10 mL) and transferred to a separatory funnel with H_2O (10 mL) and EtOAc (10 mL). The aqueous layer was extracted with EtOAc (3 x 10 mL), and the combined organic extracts were washed with brine (10 mL), dried over anhydrous Na_2SO_4, and concentrated *in vacuo* with the aid of a rotary evaporator.

To the resulting crude residue in a 100 mL round-bottom flask was added a magnetic stir bar, and the residue was dissolved in acetone (10 mL) and H_2O (10 mL, 0.1 M total). Then *p*-TsOH·H_2O (38 mg, 0.20 mmol, 10 mol%) was added and the reaction mixture was allowed to stir at room temperature. After 24 h, all volatiles were removed *in vacuo* with the aid of a rotary evaporator. The resulting residue was neutralized with sat. aq. $NaHCO_3$ (2 mL) and transferred to a separatory funnel with H_2O (10 mL) and EtOAc (10 mL). The aqueous layer was extracted with EtOAc (3 x 10 mL), and the combined organic extracts were washed with brine (10 mL), dried over anhydrous Na_2SO_4, and concentrated *in vacuo* with the aid of a rotary evaporator. The residue was purified by flash column chromatography on silica gel (gradient elution: CH_2Cl_2 to 5% EtOAc in CH_2Cl_2) to afford 6-hydroxy-6,6-diphenylhexanal (**SI-11**) as a colorless solid (240 mg, 45%).

^{1}H NMR (600 MHz, $CDCl_3$): δ 9.72 (t, *J* = 1.7 Hz, 1H), 7.41–7.38 (m, 4H), 7.33–7.29 (m, 4H), 7.24–7.21 (m, 2H), 2.40 (td, *J* = 7.5, 1.7 Hz, 2H), 2.31–2.29 (m, 2H), 2.10 (s, 1H), 1.66 (p, *J* = 7.6 Hz, 2H), 1.35–1.30 (m, 2H).

^{13}C NMR (151 MHz, $CDCl_3$): δ 202.7, 147.0, 128.4, 127.0, 126.1, 78.3, 44.0, 41.8, 23.6, 22.5.

IR (Diamond-ATR, neat) ṽ (cm^{-1}): 3464, 3082, 3051, 3023, 2934, 2864, 2827, 2726, 1716, 1595, 1491, 1447, 1388, 1358, 1175, 1058, 1030, 997, 983, 913, 875.

HRMS (ESI): m/z: $[M+H]^+$ calc'd for $C_{18}H_{21}O_2^+$: 269.1536. Found: 269.1533.

3-(3-((5-(4-Fluorophenyl)thiophen-2-yl)methyl)-4-methylphenyl)propanal (SI-12)

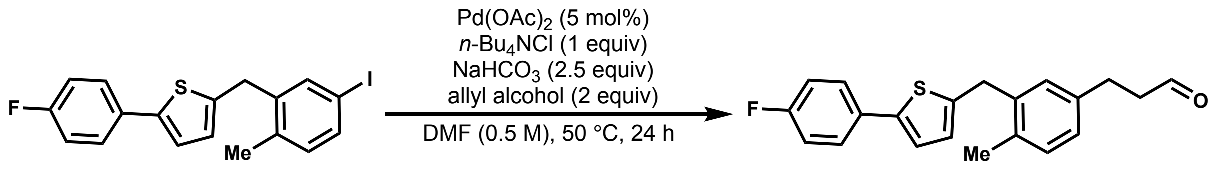

SI-12

To a flame-dried 20 mL reaction tube equipped with a magnetic stir bar was added 2-(4-fluorophenyl)-5-(5-iodo-2-methylbenzyl)thiophene (1.63 g, 4.00 mmol, 1.0 equiv), $Pd(OAc)_2$ (45 mg, 0.20 mmol, 5 mol%), $NaHCO_3$ (840 mg, 10.0 mmol, 2.5 equiv), and *n*-Bu_4NCl (1.11 g, 4.00 mmol, 1.0 equiv). The tube was then sealed with an aluminum crimp cap and evacuated then backfilled with N_2 utilizing a dual manifold Schlenk line. This process was repeated three times. Then the reaction mixture was diluted with DMF (8 mL, 0.5 M) and allyl alcohol (544 µL, 8.00 mmol, 2 equiv), and the tube was placed in an oil bath preheated to 50 °C and allowed to stir. After 24 h, the tube was removed from the oil bath and allowed to cool to room temperature. The mixture was diluted with sat. aq. NH_4Cl (25 mL) and transferred to a separatory funnel with H_2O (10 mL) and Et_2O (20 mL). The aqueous layer was extracted with Et_2O (3 x 20 mL), and the combined organic extracts were washed with brine (3 x 20 mL), dried over anhydrous Na_2SO_4, and concentrated *in vacuo* with the aid of a rotary evaporator. The crude residue was purified by flash column chromatography on silica gel (gradient elution: hexanes to 20% EtOAc in hexanes) to give 3-(3-((5-(4-fluorophenyl)thiophen-2-yl)methyl)-4-methylphenyl)propanal (**SI-12**) as a colorless solid (650 mg, 48%).

^{1}H NMR (600 MHz, $CDCl_3$): δ 9.82 (t, *J* = 1.5 Hz, 1H), 7.50–7.46 (m, 2H), 7.11 (d, *J* = 7.7 Hz, 1H), 7.06–7.00 (m, 5H), 6.66 (dt, *J* = 3.6, 1.0 Hz, 1H), 4.10 (s, 2H), 2.93 (t, *J* = 7.6 Hz, 2H), 2.76 (td, *J* = 7.9, 1.5 Hz, 2H), 2.29 (s, 3H).

^{13}C NMR (151 MHz, $CDCl_3$): δ 201.9, 162.2 (d, *J* = 246.6 Hz), 143.5, 141.7, 138.5, 138.3, 134.4, 131.0 (d, *J* = 3.0 Hz), 130.9, 129.6, 127.3 (d, *J* = 8.0 Hz), 126.9, 126.1, 122.8, 115.8 (d, *J* = 21.9 Hz), 45.5, 34.2, 27.9, 19.2.

^{19}F NMR (470 MHz, $CDCl_3$): δ −115.2.

IR (Diamond-ATR, neat) ṽ (cm^{-1}): 3068, 3035, 3002, 2920, 2857, 2822, 2721, 1723, 1684, 1602, 1548, 1507, 1468, 1440, 1414, 1386, 1346, 1299, 1230, 1159, 1096, 1049, 1014, 955, 894, 833, 802.

HRMS (ESI): m/z: $[M+H]^+$ calc'd for $C_{21}H_{20}FOS^+$: 339.1213. Found: 339.1211.

Dimethoxyacetaldehyde, 22 wt% in MTBE (2d)

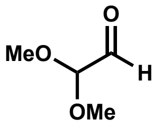

2d

To a flame-dried 25 mL round-bottom flask equipped with a magnetic stir bar was added activated powdered 4 Å molecular sieves (4.6 g). The flask was then sealed with a rubber septum and evacuated then backfilled with N_2 utilizing a dual manifold Schlenk line. This process was repeated three times. Then, MTBE (10 mL) was added via syringe, and the mixture was allowed to stir vigorously at room temperature for 1 min before dimethoxyacetaldehyde (1.9 mL, 60 wt% in H_2O) was added via syringe over 1 minute. The mixture was stirred vigorously for 24 hours, then filtered through a plug of packed Celite (*ca*. 10 g) in a glass fritted funnel, washed with MTBE (10 mL), and concentrated *in vacuo* with the aid of a rotary evaporator until a volume of *ca.* 2 mL. The remaining colorless solution was stored over beaded 4 Å molecular sieves under N_2 at −20 °C. An aliquot was analyzed with ^{1}H NMR spectroscopy to determine the concentration of dimethoxyacetaldehyde to be 22 wt%.

4. General Procedures for Decarboxylative Nozaki-Hiyama-Kishi Coupling

4.1. General Procedure F: Decarboxylative Nozaki-Hiyama-Kishi Coupling

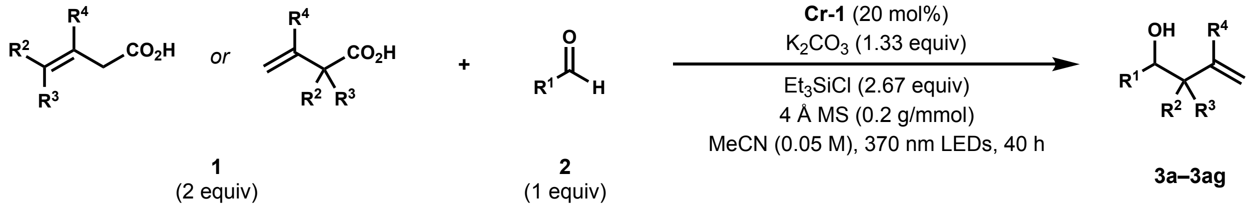

Inside a N_2-filled glovebox, an oven-dried reaction tube (Thermo Scientific, catalog no. CHCV20-14) equipped with a magnetic stir bar (Chemglass Life Sciences, catalog no. CG-2003-17, 12.7 x 3 mm) was charged with K_2CO_3 (55 mg, 0.40 mmol, 1.33 equiv), 4 Å molecular sieves (60 mg, 0.2 g/mmol), and MeCN (6.0 mL, 0.05 M). Et_3SiCl (134 µL, 0.80 mmol, 2.67 equiv) was added dropwise via syringe under vigorous stirring, and the resulting mixture was allowed to stir at room temperature for 5 min (Figure **SI-2a**–**SI-2b**). To the stirred reaction mixture was added $[Cr(dtbbpy)_2Cl_2]Cl$ (**Cr-1**) (42 mg, 0.06 mmol, 20 mol%), the carboxylic acid (0.60 mmol, 2 equiv), and the aldehyde (0.30 mmol, 1 equiv) (Figure **SI-2b**). The reaction vessel was sealed with a DWK Life Sciences PTFE/Silicone lined aluminum crimp vial seal (Thermo Scientific, catalog no. 15-111-703), removed from the glovebox, and placed in a custom-made photoreactor 3 cm away from two 44 W Kessil PR-160L 370 nm LEDs and one 75 mm fan (Figure **SI-2c**). The reaction mixture was subjected to LED irradiation at 100% intensity with vigorous stirring (*ca.* 50 °C). After 40 h, the reaction mixture was allowed to cool to room temperature and concentrated *in vacuo* with the aid of a rotary evaporator (**SI-2d**). The residue was purified by flash column chromatography on silica gel to yield the corresponding product.

Note: vigorous stirring was crucial to ensure reaction mixture homogeneity and maintain a reproducible yield.

4.1.1. Graphical General Procedure F: Decarboxylative Nozaki-Hiyama-Kishi Coupling

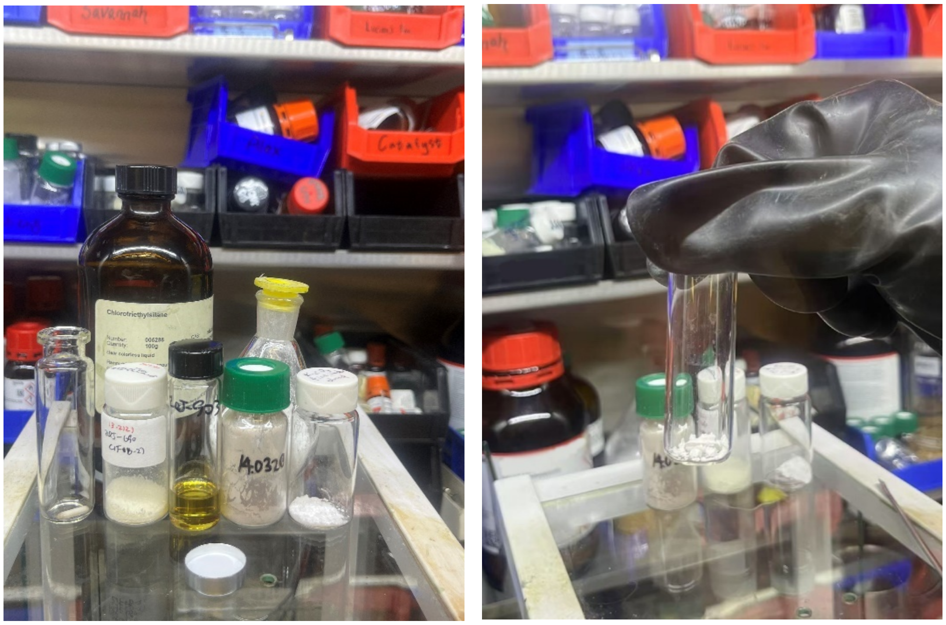

Figure SI-2a: Left: All materials needed, front left to right: oven-dried reaction tube equipped with stir bar, aldehyde, carboxylic acid, [Cr(dtbbpy)$_2$Cl$_2$]Cl (**Cr-1**), K_2CO_3. Back left to right: Et_3SiCl, 4 Å molecular sieves. Right: K_2CO_3 and 4 Å molecular sieves were added to the reaction vessel.

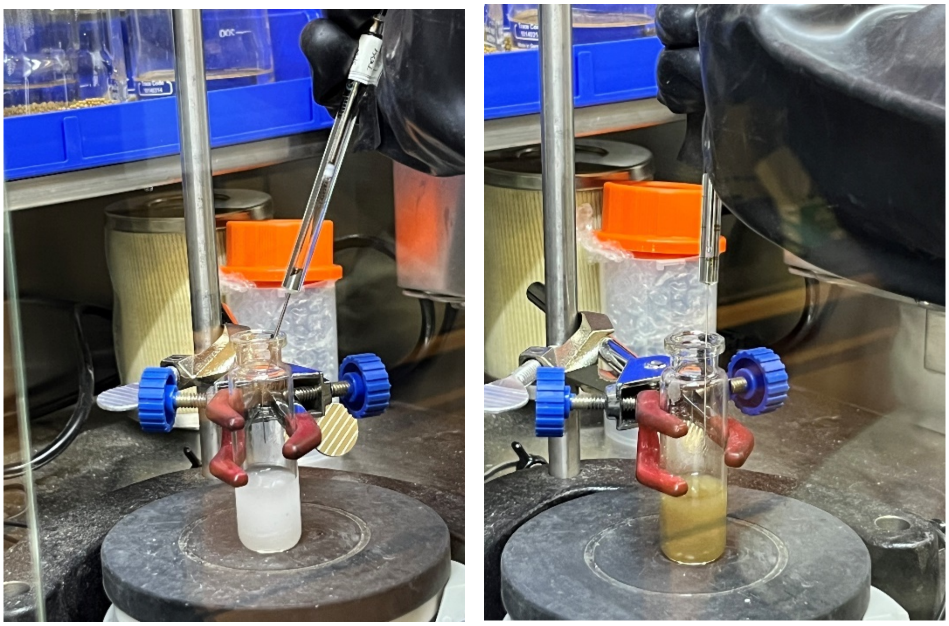

Figure SI-2b: Left: MeCN (6 mL) and Et_3SiCl (134 μL) were added via syringe and the mixture was stirred for 5 min. Right: **Cr-1**, the carboxylic acid, and the aldehyde were added.

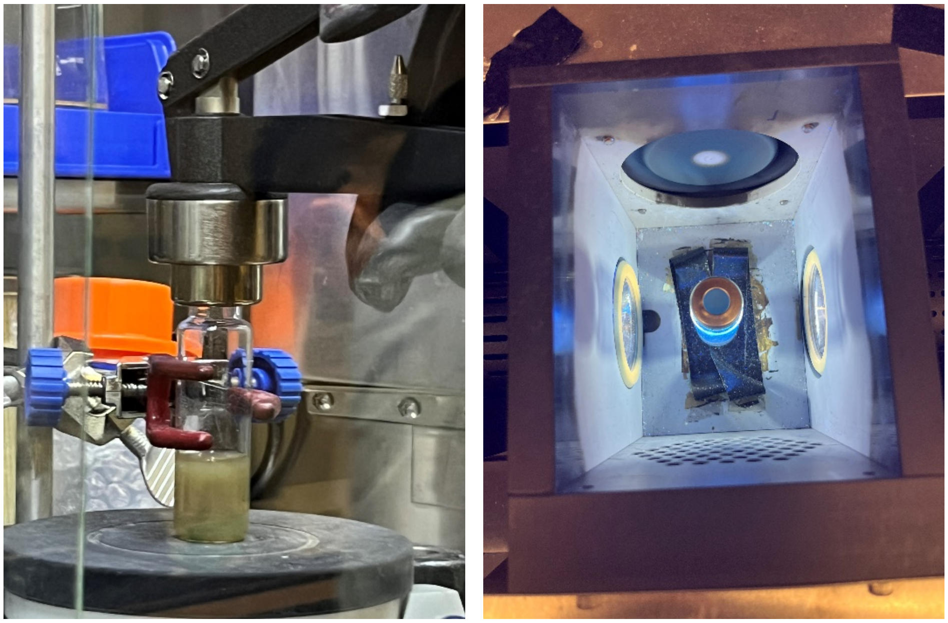

Figure SI-2c: Left: Reaction tube is sealed with an aluminum crimp cap. Right: The tube is brought out of the glovebox and placed in a custom made photoreactor 3 cm away from two 44 W 370 nm Kessil lamps. The two lamps were set to 100% intensity and the 75 mm fan was turned on (*ca.* 50 ºC).

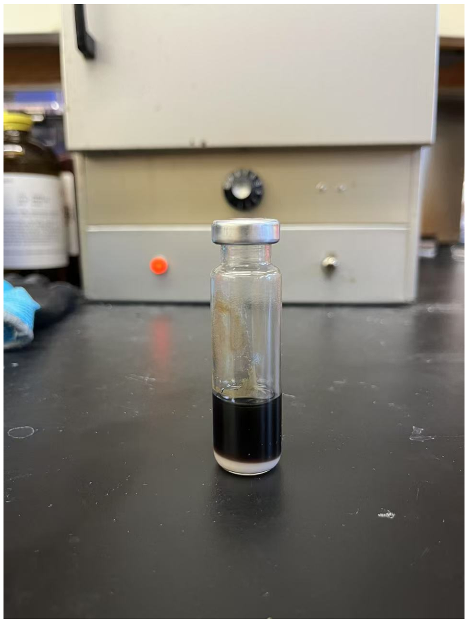

Figure SI-2d: Appearance of reaction mixture after 40 h of irradiation.

5. Synthesis and Characterization of Decarboxylative Nozaki-Hiyama-Kishi Coupling Products

(±)-(3*R,4R*)-4-Cyclohexyl-1-phenylhex-5-en-3-ol (3a)

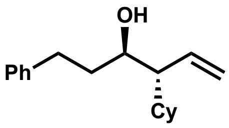

3a

Prepared according to a modified **General Procedure F** from (*E*)-4-cyclohexylbut-3-enoic acid (**1a**) (34 µL, 0.20 mmol) and 3-phenylpropanal (**2a**) (13 µL, 0.10 mmol). A NMR yield was determined by ^{1}H NMR spectroscopy of the crude reaction mixture using 1,1,2,2-tetrachloroethane (TCE) as the internal standard (78% ^{1}H NMR yield). An analytically pure sample of **3a** was purified by preparatory thin-layer chromatography (70% CH_2Cl_2 in hexanes) and isolated as a colorless oil and 14:1 mixture of diastereomers.

^{1}H NMR (600 MHz, $CDCl_3$, major diastereomer): δ 7.28 (t, *J* = 7.5 Hz, 2H), 7.21 (d, *J* = 7.3 Hz, 2H), 7.18 (t, *J* = 7.4 Hz, 1H), 5.71 (dt, *J* = 17.2, 10.1 Hz, 1H), 5.20 (dd, *J* = 10.3, 2.1 Hz, 1H), 5.05 (dd, *J* = 17.2, 2.1 Hz, 1H), 3.74 (dt, *J* = 8.8, 4.4 Hz, 1H), 2.80 (ddd, *J* = 13.9, 10.3, 5.5 Hz, 1H), 2.66 (ddd, *J* = 13.8, 10.0, 6.5 Hz, 1H), 1.83–1.63 (m, 7H), 1.51–1.44 (m, 1H), 1.32–1.07 (m, 4H), 0.99 (qd, *J* = 12.0, 3.0 Hz, 1H), 0.90 (qd, *J* = 12.0, 3.0 Hz, 1H).

^{13}C NMR (151 MHz, $CDCl_3$, major diastereomer): δ 142.5, 137.0, 128.6, 128.5, 125.9, 118.8, 70.1, 56.4, 37.9, 37.2, 32.3, 31.9, 30.0, 26.7, 26.7, 26.6.

IR (Diamond-ATR, neat) ṽ (cm^{-1}): 3383, 2923, 2851, 1277, 1258, 1059, 1043, 1002, 910.

HRMS (ESI): m/z: $[M+H]^+$ calc'd for $C_{18}H_{27}O^+$: 259.2056. Found: 259.2052.

(±)-*tert*-Butyl 4-((1*R*,2*R*)-2-cyclohexyl-1-hydroxybut-3-en-1-yl)piperidine-1-carboxylate (3b)

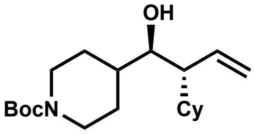

3b

Prepared according to **General Procedure F** from (*E*)-4-cyclohexylbut-3-enoic acid (**1a**) (101 µL, 0.60 mmol) and *tert*-butyl 4-formylpiperidine-1-carboxylate (64 mg, 0.30 mmol). The residue was purified by flash column chromatography on silica gel (gradient elution: hexanes to 50% CH_2Cl_2 in hexanes) to yield **3b** as a colorless oil and >20:1 mixture of diastereomers (78 mg, 77%).

^{1}H NMR (600 MHz, $CDCl_3$): δ 5.71 (dt, *J* = 17.2, 10.1 Hz, 1H), 5.19 (dd, *J* = 10.4, 2.1 Hz, 1H), 5.03 (dd, *J* = 17.3, 2.0 Hz, 1H), 4.12 (br s, 2H), 3.44 (dd, *J* = 6.7, 4.7 Hz, 1H), 2.65

(br s, 2H), 1.90 (ddd, J = 9.9, 7.3, 4.5 Hz, 1H), 1.82 (dt, J = 13.1, 2.5 Hz, 1H), 1.76–1.68 (m, 4H), 1.65 (d, J = 12.7 Hz, 1H), 1.55–1.49 (m, 1H), 1.45 (s, 9H), 1.44–1.32 (m, 2H), 1.31–1.07 (m, 6H), 1.01–0.85 (m, 2H).

^{13}C NMR (151 MHz, $CDCl_3$): δ 155.0, 136.8, 118.5, 79.4, 73.9, 52.5, 39.1, 37.5, 31.6, 30.6, 28.6, 26.7, 26.6, 26.6.

IR (Diamond-ATR, neat) ṽ (cm^{-1}): 3452, 2972, 2965, 2921, 2848, 1668, 1477, 1468, 1448, 1426, 1392, 1366, 1281, 1252, 1222, 1166, 1136, 1114, 1067, 1005, 976, 939, 912, 869, 817.

HRMS (ESI): m/z: $[M+H]^+$ calc'd for $C_{20}H_{36}NO_3^+$: 338.2723. Found: 338.2720.

(±)-(1*R*,2*R*)-2-Cyclohexyl-1-(4,4-difluorocyclohexyl)but-3-en-1-ol (3c)

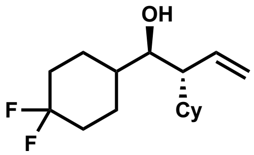

3c

Prepared according to **General Procedure F** from (*E*)-4-cyclohexylbut-3-enoic acid (**1a**) (101 µL, 0.60 mmol) and 4,4-difluorocyclohexane-1-carbaldehyde (40 µL, 0.30 mmol). The residue was purified by flash column chromatography on silica gel (gradient elution: 15% CH_2Cl_2 in hexanes to CH_2Cl_2) to yield **3c** as a red-brown oil and >20:1 mixture of diastereomers (66 mg, 81%).

^{1}H NMR (600 MHz, $CDCl_3$): δ 5.71 (dt, J = 17.2, 10.1 Hz, 1H), 5.20 (dd, J = 10.2, 2.1 Hz, 1H), 5.04 (dd, J = 17.2, 2.1 Hz, 1H), 3.48 (dd, J = 6.7, 4.7 Hz, 1H), 2.16–2.05 (m, 2H), 1.98–1.93 (m, 1H), 1.90 (ddd, J = 10.0, 7.4, 4.6 Hz, 1H), 1.78–1.62 (m, 8H), 1.50–1.34 (m, 5H), 1.31–1.08 (m, 3H), 1.01–0.94 (m, 1H), 0.93–0.87 (m, 1H).

^{13}C NMR (151 MHz, $CDCl_3$): δ 136.7, 123.8 (dd, J = 241.9, 239.3 Hz), 118.6, 73.5 (d, J = 2.2 Hz), 53.0, 38.8, 37.5, 33.5 (t, J = 22.8 Hz), 33.4 (t, J = 22.8 Hz), 31.6, 30.5, 26.7, 26.6, 26.6, 25.5 (d, J = 9.8 Hz), 24.4 (d, J = 9.8 Hz).

^{19}F NMR (470 MHz, $CDCl_3$) δ −91.5 (d, J = 235.2 Hz), −102.6 (dt, J = 234.4, 34.1 Hz).

IR (Diamond-ATR, neat) ṽ (cm^{-1}): 3322, 2961, 2930, 2847, 1448, 1388, 1363, 1277, 1195, 1122, 1097, 1074, 1043, 979, 960, 926, 922.

HRMS (ESI): m/z: $[M{-}OH]^+$ calc'd for $C_{16}H_{25}F_2^+$: 255.1919. Found: 255.1921.

(±)-*tert*-Butyl 3-((1*S*,2*R*)-2-cyclohexyl-1-hydroxybut-3-en-1-yl)azetidine-1-carboxylate (3d)

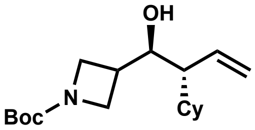

3d

Prepared according to **General Procedure F** from (*E*)-4-cyclohexylbut-3-enoic acid (**1a**) (101 µL, 0.60 mmol) and *tert*-butyl 3-formylazetidine-1-carboxylate (56 mg, 0.30 mmol). The residue was purified by flash column chromatography on silica gel (gradient elution: hexanes to 50% CH_2Cl_2 in hexanes) to yield **3d** as a colorless oil and 16:1 mixture of diastereomers (73 mg, 78%).

^{1}H NMR (600 MHz, $CDCl_3$, major diastereomer): δ 5.68 (dt, *J* = 17.2, 10.1 Hz, 1H), 5.16 (dd, *J* = 10.2, 2.0 Hz, 1H), 4.96 (dd, *J* = 17.1, 1.6 Hz, 1H), 3.93–3.88 (m, 3H), 3.83 (dd, *J* = 8.5, 5.8 Hz, 1H), 3.64 (dd, *J* = 8.5, 6.0 Hz, 1H), 2.64 (qt, *J* = 8.0, 6.0 Hz, 1H), 1.78–1.63 (m, 6H), 1.43 (s, 9H), 1.34–1.08 (m, 4H), 1.02–0.94 (m, 1H), 0.92–0.85 (m, 1H).

^{13}C NMR (151 MHz, $CDCl_3$, major diastereomer): δ 156.5, 136.5, 118.8, 79.4, 72.4, 54.2, 37.6, 32.8, 31.6, 30.8, 28.6, 26.6, 26.5, 26.5.

IR (Diamond-ATR, neat) ṽ (cm^{-1}): 3407, 2979, 2922, 2853, 1678, 1477, 1419, 1393, 1367, 1253, 1144, 1065, 1002, 965, 914, 857.

HRMS (ESI): m/z: $[M+H]^+$ calc'd for $C_{18}H_{32}NO_3^+$: 310.2377. Found: 310.2372.

(±)-*tert*-Butyl-2-((1*S*,2*R*)-2-cyclohexyl-1-hydroxybut-3-en-1-yl)-1*H*-pyrrole-1-carboxylate (3e)

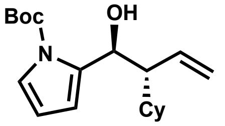

3e

Prepared according to **General Procedure F** from (*E*)-4-cyclohexylbut-3-enoic acid (**1a**) (101 µL, 0.60 mmol) and *tert*-butyl 2-formyl-1*H*-pyrrole-1-carboxylate (59 mg, 0.30 mmol). The residue was purified by automated flash column chromatography (40 g SiO_2, gradient elution: hexanes to 5% Et_2O in hexanes) to yield **3e** as a red-brown oil and >20:1 mixture of diastereomers (60 mg, 63%).

^{1}H NMR (600 MHz, $CDCl_3$): δ 7.15 (dd, *J* = 3.3, 1.7 Hz, 1H), 6.23 (dd, *J* = 3.2, 1.6 Hz, 1H), 6.09 (t, *J* = 3.3 Hz, 1H), 5.87 (dt, *J* = 17.1, 10.1 Hz, 1H), 5.18 (dd, *J* = 10.2, 2.2 Hz, 1H), 5.05–5.01 (m, 2H), 3.84 (d, *J* = 7.4 Hz, 1H), 2.38 (ddd, *J* = 9.8, 7.4, 4.7 Hz, 1H), 1.76–1.66 (m, 3H), 1.60 (s, 9H), 1.29–1.12 (m, 5H), 1.10–1.06 (m, 2H), 0.97–0.90 (m, 1H).

^{13}**C NMR** (151 MHz, $CDCl_3$): δ 150.4, 137.8, 137.2, 122.2, 117.6, 113.4, 110.4, 84.6, 67.7, 54.8, 39.2, 32.1, 29.1, 28.1, 26.8, 26.7, 26.7.

IR (Diamond-ATR, neat) ṽ (cm^{-1}): 3459, 2977, 2923, 2845, 1739, 1722, 1447, 1407, 1371, 1329, 1259, 1227, 1165, 1123, 1055, 998, 910, 847.

HRMS (ESI): m/z: $[M+H]^+$ calc'd for $C_{19}H_{30}NO_3^+$: 320.2220. Found: 320.2234.

(±)-(3*R*,4*R*)-8-Chloro-3-cyclohexyloct-1-en-4-ol (3f)

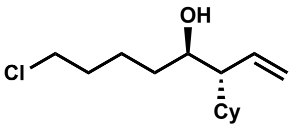

3f

Prepared according to **General Procedure F** from (*E*)-4-cyclohexylbut-3-enoic acid (**1a**) (101 µL, 0.60 mmol) and 5-chloropentanal (36 µL, 0.30 mmol). The residue was purified by flash column chromatography on silica gel (gradient elution: 30% CH_2Cl_2 in hexanes to CH_2Cl_2) to yield **3f** as a colorless oil and 16:1 mixture of diastereomers (48 mg, 65%).

^{1}**H NMR** (600 MHz, $CDCl_3$, major diastereomer): δ 5.69 (dt, *J* = 17.2, 10.1 Hz, 1H), 5.20 (dd, *J* = 10.3, 2.2 Hz, 1H), 5.04 (dd, *J* = 17.2, 2.2 Hz, 1H), 3.73–3.70 (m, 1H), 3.55 (t, *J* = 6.6 Hz, 2H), 1.85–1.68 (m, 6H), 1.66–1.59 (m, 1H), 1.53–1.40 (m, 4H), 1.38 (br s, 1H), 1.30–1.08 (m, 4H), 1.04–0.97 (m, 1H), 0.95–0.88 (m, 1H).

^{13}**C NMR** (151 MHz, $CDCl_3$, major diastereomer): δ 136.9, 118.8, 70.3, 56.2, 45.2, 37.8, 34.5, 32.8, 31.9, 30.0, 26.7, 26.7, 26.6, 23.2.

IR (Diamond-ATR, neat) ṽ (cm^{-1}): 3379, 2922, 2853, 1634, 1449, 1035, 1002, 912.

HRMS (ESI): m/z: $[M-OH]^+$ calc'd for $C_{14}H_{24}Cl^+$: 227.1561. Found: 227.1561.

(±)-(1*S*,2*R*)-2-Cyclohexyl-1-(1-(2-fluorophenyl)-1*H*-pyrazol-4-yl)but-3-en-1-ol (3g)

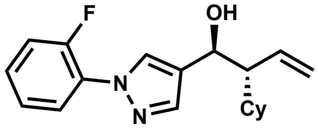

3g

Prepared according to **General Procedure F** from (*E*)-4-cyclohexylbut-3-enoic acid (**1a**) (101 µL, 0.60 mmol) and 1-(2-fluorophenyl)-1*H*-pyrazole-4-carboxaldehyde (57 mg, 0.30 mmol). The residue was purified by flash column chromatography on silica gel (gradient elution: hexanes to 75% CH_2Cl_2 in hexanes) to yield **3g** as a yellow solid and 11:1 mixture of diastereomers (58 mg, 62%).

^{1}**H NMR** (600 MHz, $CDCl_3$, major diastereomer): δ 7.97 (d, *J* = 2.9 Hz, 1H), 7.92–7.89 (m, 1H), 7.71 (s, 1H), 7.30–7.26 (m, 1H), 7.26–7.20 (m, 2H), 5.84 (dt, *J* = 17.1, 10.1 Hz, 1H),

5.30 (dd, J = 10.2, 2.0 Hz, 1H), 5.16 (dd, J = 17.2, 1.9 Hz, 1H), 4.83 (d, J = 7.8 Hz, 1H), 2.13 (ddd, J = 9.8, 7.9, 4.8 Hz, 1H), 1.97 (br s, 1H), 1.76–1.67 (m, 3H), 1.63–1.58 (m, 1H), 1.41–1.36 (m, 1H), 1.25–0.90 (m, 6H).

^{13}C NMR (151 MHz, $CDCl_3$, major diastereomer): δ 153.5 (d, J = 248.8 Hz), 139.5, 136.6, 129.1 (d, J = 10.5 Hz), 128.5 (d, J = 9.1 Hz), 127.7 (d, J = 7.8 Hz), 125.9, 125.1 (d, J = 3.8 Hz), 124.2, 120.2, 117.0 (d, J = 20.5 Hz), 66.6, 58.4, 38.2, 32.3, 28.7, 26.6, 26.6, 26.5.

^{19}F NMR (470 MHz, $CDCl_3$, major diastereomer): δ −125.1.

IR (Diamond-ATR, neat) ṽ (cm^{-1}): 3393, 3068, 2922, 2851, 1620, 1597, 1559, 1506, 1470, 1452, 1401, 1229, 1201, 1111, 1041, 1020, 952, 913, 861, 818.

HRMS (ESI): m/z: $[M+H]^+$ calc'd for $C_{19}H_{24}FN_2O^+$: 315.1867. Found: 315.1864.

(±)-Methyl (6*R*,7*R*)-7-cyclohexyl-6-hydroxynon-8-enoate (3h)

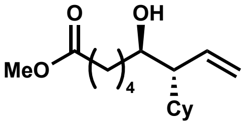

3h

Prepared according to **General Procedure F** from (*E*)-4-cyclohexylbut-3-enoic acid (**1a**) (101 µL, 0.60 mmol) and methyl 6-oxohexanoate (43 µL, 0.30 mmol). The residue was purified by flash column chromatography on silica gel (gradient elution: 1% acetone in CH_2Cl_2 to 3.5% acetone in CH_2Cl_2) to yield **3h** as a red-brown oil and 14:1 mixture of diastereomers (51 mg, 63%).

^{1}H NMR (600 MHz, $CDCl_3$, major diastereomer): δ 5.69 (dt, J = 17.1, 10.1 Hz, 1H), 5.19 (dd, J = 10.3, 2.2 Hz, 1H), 5.02 (dd, J = 17.1, 2.1 Hz, 1H), 3.71 (dt, J = 7.8, 4.4 Hz, 1H), 3.67 (s, 3H), 2.33 (t, J = 7.5 Hz, 2H), 1.74–1.61 (m, 8H), 1.50–1.34 (m, 6H), 1.30–1.08 (m, 3H), 1.03–0.96 (m, 1H), 0.94–0.87 (m, 1H).

^{13}C NMR (151 MHz, $CDCl_3$, major diastereomer): δ 174.4, 137.0, 118.7, 70.3, 56.2, 51.6, 37.8, 35.0, 34.2, 31.9, 30.1, 26.7, 26.7, 26.6, 25.4, 25.1.

IR (Diamond-ATR, neat) ṽ (cm^{-1}): 3482, 2922, 2852, 1738, 1723, 1637, 1448, 1437, 1419, 1365, 1257, 1194, 1171, 1157, 1110, 1098, 1003, 910, 854.

HRMS (ESI): m/z: $[M+H]^+$ calc'd for $C_{16}H_{29}O_3^+$: 269.2111. Found: 269.2106.

(±)-(1*S*,2*R*)-2-Cyclohexyl-1-(5-methylfuran-2-yl)but-3-en-1-ol (3i)

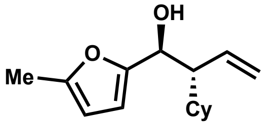

3i

Prepared according to **General Procedure F** from (*E*)-4-cyclohexylbut-3-enoic acid (**1a**) (101 µL, 0.60 mmol) and 5-methylfuran-2-carbaldehyde (30 µL, 0.30 mmol). The residue was purified by flash column chromatography on silica gel (gradient elution: 1% Et_2O in hexanes to 6% Et_2O in hexanes) to yield **3i** as a yellow oil and 11:1 mixture of diastereomers (54 mg, 76%).

^{1}H NMR (600 MHz, $CDCl_3$, major diastereomer): δ 6.14 (d, *J* = 3.0 Hz, 1H), 5.90 (dd, *J* = 3.0, 0.9 Hz, 1H), 5.79 (dt, *J* = 17.1, 10.1 Hz, 1H), 5.26 (dd, *J* = 10.2, 2.1 Hz, 1H), 5.16 (dd, *J* = 17.1, 2.0 Hz, 1H), 4.64 (dd, *J* = 8.3, 3.5 Hz, 1H), 2.37 (td, *J* = 9.1, 4.5 Hz, 1H), 2.29 (s, 3H), 1.94 (d, *J* = 3.7 Hz, 1H), 1.70–1.65 (m, 3H), 1.62–1.57 (m, 1H), 1.55–1.52 (m, 1H), 1.24–1.15 (m, 2H), 1.12–1.04 (m, 3H), 0.99–0.92 (m, 1H).

^{13}C NMR (151 MHz, $CDCl_3$, major diastereomer): δ 153.3, 151.8, 136.7, 119.7, 108.3, 106.1, 68.0, 55.7, 38.4, 32.1, 28.6, 26.7, 26.6, 26.6, 13.8.

IR (Diamond-ATR, neat) ṽ (cm^{-1}): 3433, 2922, 2852, 1641, 1562, 1449, 1225, 1021, 1000, 908.

HRMS (ESI): m/z: $[M+H]^+$ calc'd for $C_{15}H_{23}O_2^+$: 235.1693. Found: 235.1698.

(±)-*N*-(4-((1*S*,2*R*)-2-Cyclohexyl-1-hydroxybut-3-en-1-yl)phenyl)acetamide (3j)

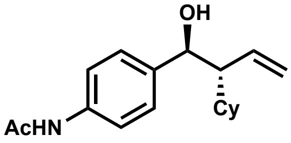

3j

Prepared according to **General Procedure F** from (*E*)-4-cyclohexylbut-3-enoic acid (**1a**) (101 µL, 0.60 mmol) and 4-acetamidobenzaldehyde (49 mg, 0.30 mmol). The residue was purified by flash column chromatography on silica gel (gradient elution: CH_2Cl_2 to 10% EtOAc in CH_2Cl_2) to yield **3j** as a yellow solid and 11:1 mixture of diastereomers (51 mg, 59%).

^{1}H NMR (600 MHz, $CDCl_3$, major diastereomer): δ 7.48 (d, *J* = 8.3 Hz, 2H), 7.28 (d, *J* = 8.4 Hz, 2H), 7.18 (br s, 1H), 5.80 (dt, *J* = 17.1, 10.0 Hz, 1H), 5.25 (dd, *J* = 10.2, 1.9 Hz, 1H), 5.09 (dd, *J* = 17.2, 1.9 Hz, 1H), 4.65 (d, *J* = 8.2 Hz, 1H), 2.18 (s, 3H), 2.10 (td, *J* = 9.2, 4.0 Hz, 1H), 2.01 (br s, 1H), 1.76–1.60 (m, 4H), 1.47–1.41 (m, 1H), 1.17–0.95 (m, 6H).

^{13}C NMR (151 MHz, $CDCl_3$, major diastereomer): δ 168.3, 139.0, 137.3, 136.7, 127.6, 119.9, 119.7, 73.7, 58.9, 38.0, 32.3, 28.5, 26.6, 26.5, 26.5, 24.8.

IR (Diamond-ATR, neat) ṽ (cm^{-1}): 3295, 3063, 2923, 2850, 1667, 1604, 1539, 1516, 1449, 1413, 1371, 1318, 1180, 1044, 998, 967, 913, 840.

HRMS (ESI): m/z: $[M+H]^+$ calc'd for $C_{18}H_{26}NO_2^+$: 288.1958. Found: 288.1953.

(±)-(2*R*,3*R*)-3-Cyclohexyl-1-(2,6,6-trimethylcyclohex-1-en-1-yl)pent-4-en-2-ol (3k)

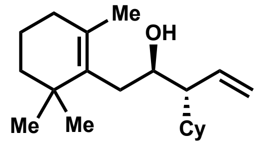

3k

Prepared according to **General Procedure F** from (*E*)-4-cyclohexylbut-3-enoic acid (**1a**) (101 µL, 0.60 mmol) and 2-(2,6,6-trimethylcyclohex-1-en-1-yl)acetaldehyde (50 mg, 0.30 mmol). The residue was purified by flash column chromatography on silica gel (gradient elution: hexanes to 50% CH_2Cl_2 in hexanes) to yield **3k** as a colorless oil and 14:1 mixture of diastereomers (56 mg, 64%).

^{1}H NMR (600 MHz, $CDCl_3$, major diastereomer): δ 5.81 (dt, *J* = 17.2, 10.1 Hz, 1H), 5.17 (dd, *J* = 10.2, 2.4 Hz, 1H), 5.00 (dd, *J* = 17.3, 2.4 Hz, 1H), 3.95 (dt, *J* = 10.9, 3.3 Hz, 1H), 2.41 (dd, *J* = 14.3, 11.0 Hz, 1H), 2.05–1.99 (m, 2H), 1.98–1.91 (m, 1H), 1.83–1.76 (m, 2H), 1.75–1.68 (m, 3H), 1.64 (s, 3H), 1.63–1.60 (m, 2H), 1.56–1.48 (m, 2H), 1.46–1.42 (m, 2H), 1.31–1.08 (m, 4H), 1.02 (s, 3H), 1.01–0.98 (m, 4H), 0.89 (qd, *J* = 12.5, 3.3 Hz, 1H).

^{13}C NMR (151 MHz, $CDCl_3$, major diastereomer): δ 137.7, 134.0, 131.6, 117.6, 69.7, 56.9, 40.2, 38.5, 35.0, 34.6, 33.2, 31.6, 31.1, 29.6, 28.8, 26.8, 26.7, 26.7, 21.3, 19.5.

IR (Diamond-ATR, neat) ṽ (cm^{-1}): 3528, 1473, 1448, 1382, 1359, 1106, 1074, 1005, 907.

HRMS (ESI): m/z: $[M+H]^+$ calc'd for $C_{20}H_{35}O^+$: 291.2682. Found: 291.2679.

(±)-*tert*-Butyl-6-(1-hydroxy-3-phenylpropyl)-6-vinyl-2-azaspiro[3.3]heptane-2-carboxylate (3l)

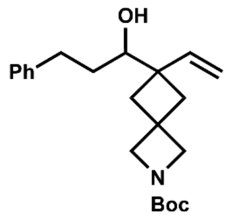

3l

Prepared according to a modified **General Procedure F** from 3-(2-(*tert*-butoxycarbonyl)-2-azaspiro[3.3]heptan-6-ylidene)propanoic acid (**SI-2**) (160 mg, 0.60 mmol) and 3-phenylpropanal (**2a**) (40 µL 0.30 mmol). The residue was purified by flash column

chromatography on silica gel (gradient elution: 2% EtOAc in hexanes to 20% EtOAc in hexanes) to produce **3l** as a mixture with impurities, which was subsequently purified by flash column chromatography on silica gel (25% EtOAc in hexanes) to yield **3l** as a colorless oil (80 mg, 75%).

^{1}H NMR (600 MHz, $CDCl_3$): δ 7.31–7.27 (m, 2H) 7.21–7.16 (m, 3H), 5.87 (dd, *J* = 17.4, 10.7 Hz, 1H), 5.21 (dd, *J* = 10.6, 0.9 Hz, 1H), 5.10 (dd, *J* = 17.4, 0.7 Hz, 1H), 3.83–3.79 (m, 3H), 3.74 (d, *J* = 8.8 Hz, 1H), 3.41–3.35 (m, 1H), 2.85 (ddd, *J* = 14.2, 9.7, 5.0 Hz, 1H), 2.60 (ddd, *J* = 13.7, 9.3, 7.3 Hz, 1H), 2.24–2.17 (m, 2H), 2.13–2.06 (m, 2H), 1.72–1.66 (m, 1H), 1.49–1.43 (m, 1H), 1.42 (s, 9H).

^{13}C NMR (151 MHz, $CDCl_3$): δ 156.2, 142.0, 140.3, 128.6, 128.6, 126.1, 115.3, 79.4, 76.8, 44.9, 40.1, 39.7, 33.8, 32.8, 32.3, 28.5.

IR (Diamond-ATR, neat) ṽ (cm^{-1}): 3420, 2980, 2929, 2866, 1675, 1456, 1411, 1366, 1315, 1153, 1104, 914, 855.

HRMS (ESI): m/z: $[M+H]^+$ calc'd for $C_{22}H_{32}NO_3^+$: 358.2377. Found: 358.2375.

(±)-(3-Phenyl-1-(8-vinyl-1,4-dioxaspiro[4.5]decan-8-yl)propan-1-ol (3m)

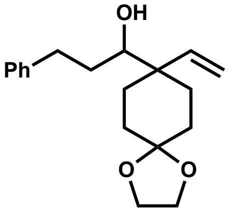

3m

Prepared according to **General Procedure F** from 3-(1,4-dioxaspiro[4.5]decan-8-ylidene)propanoic acid (**SI-3**) (127 mg, 0.60 mmol) and 3-phenylpropanal (**2a**) (40 µL, 0.30 mmol). The residue was purified by flash column chromatography on silica gel (gradient elution: 20% EtOAc in hexanes to 28% EtOAc in hexanes) to yield **3m** as a colorless oil (89 mg, 98%).

^{1}H NMR (600 MHz, $CDCl_3$): δ 7.29–7.26 (m, 2H), 7.19–7.16 (m, 3H), 5.61 (dd, *J* = 17.8, 11.0 Hz, 1H), 5.37 (dd, *J* = 11.0, 1.2 Hz, 1H), 5.14 (dd, *J* = 17.9, 1.2 Hz, 1H), 3.91 (s, 4H), 3.25 (dd, *J* = 10.8, 1.6 Hz, 1H), 2.90 (ddd, *J* = 13.8, 10.0, 4.9 Hz, 1H), 2.59 (ddd, *J* = 13.7, 9.5, 7.1 Hz, 1H), 1.90–1.84 (m, 2H), 1.71–1.68 (m, 1H), 1.64–1.49 (m, 8H).

^{13}C NMR (151 MHz, $CDCl_3$): δ 142.4, 140.7, 128.6, 128.5, 125.9, 117.8, 109.2, 77.6, 64.3, 64.3, 44.6, 33.6, 33.2, 31.1, 31.0, 29.4, 29.1.

IR (Diamond-ATR, neat) ṽ (cm^{-1}): 3639, 3454, 2951, 2923, 2880, 1641, 1602, 1496, 1445, 1381, 1161, 1108, 1087, 1065, 1034, 1007, 949, 935, 914, 876.

HRMS (ESI): m/z: $[M+H]^+$ calc'd for $C_{19}H_{27}O_3^+$: 303.1955. Found: 303.1956.

(±)-(3*R*,4*R*)-1-Phenyl-4-(tetrahydro-2*H*-thiopyran-4-yl)hex-5-en-3-ol (3o)

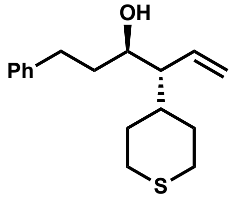

3o

Prepared according to **General Procedure F** from (*E*)-4-(tetrahydro-2*H*-thiopyran-4-yl)but-3-enoic acid (**SI-7**) (112 mg, 0.60 mmol) and 3-phenylpropanal (**2a**) (40 µL, 0.30 mmol). The residue was purified by flash column chromatography on silica gel (gradient elution: 5% EtOAc in hexanes to 10% EtOAc in hexanes) to yield **3o** as a red oil and 16:1 mixture of diastereomers (46 mg, 55%).

^{1}H NMR (600 MHz, $CDCl_3$, major diastereomer): δ 7.31–7.27 (m, 2H), 7.22–7.17 (m, 3H), 5.69 (dt, *J* = 17.2, 10.1 Hz, 1H), 5.24 (dd, *J* = 10.3, 2.0 Hz, 1H), 5.08 (dd, *J* = 17.2, 1.9 Hz, 1H), 3.74 (dt, *J* = 8.5, 4.3 Hz, 1H), 2.79 (ddd, *J* = 13.8, 9.8, 5.7 Hz, 1H), 2.72–2.64 (m, 2H), 2.62–2.56 (m, 3H), 1.98 (dd, *J* = 13.2, 2.4 Hz, 2H), 1.83–1.70 (m, 3H), 1.58–1.50 (m, 2H), 1.48–1.42 (m, 1H), 1.39–1.32 (m, 1H).

^{13}C NMR (151 MHz, $CDCl_3$, major diastereomer): δ 142.2, 136.0, 128.6, 128.6, 126.0, 119.6, 69.7, 56.1, 37.7, 37.3, 32.9, 32.3, 31.2, 29.1, 29.0.

IR (Diamond-ATR, neat) ṽ (cm^{-1}): 3630, 2930, 1499, 1454, 1424, 1257, 1063, 1007, 915.

HRMS (ESI): m/z: $[M+H]^+$ calc'd for $C_{17}H_{25}OS^+$: 277.1626. Found: 277.1619.

(±)-(3*R*,4*S*)-4-(Benzyloxy)-1-phenylhex-5-en-3-ol (3p)

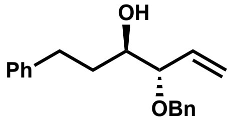

3p

Prepared according to **General Procedure F** from 2-(benzyloxy)but-3-enoic acid (**SI-8**) (115 mg, 0.60 mmol) and 3-phenylpropanal (**2a**) (40 µL, 0.30 mmol). The residue was purified by flash column chromatography on silica gel (gradient elution: 1% EtOAc in CH_2Cl_2 to 5% EtOAc in CH_2Cl_2) to yield **3p** as a yellow oil and 2:1 mixture of diastereomers (60 mg, 71%).

^{1}H NMR (600 MHz, $CDCl_3$, mixture of diastereomers): δ 7.37–7.26 (m, 7H), 7.20–7.16 (m, 3H), 5.88–5.67 (m, 1H), 5.41–5.29 (m, 2H), 4.65–4.62 (m, 1H), 4.38–4.34 (m, 1H), 3.78–3.55 (m, 2H), 2.88–2.82 (m, 1H), 2.72 (br s, 1H, major diastereomer), 2.70–2.62 (m, 1H), 2.18 (br s, 1H, minor diastereomer), 1.83–1.67 (m, 2H).

^{13}C NMR (151 MHz, $CDCl_3$, mixture of diastereomers): δ 142.3, 142.3, 138.3, 138.1, 135.3, 134.5, 128.7, 128.6, 128.6, 128.5, 128.5, 128.1, 127.9, 127.8, 125.9, 125.9, 120.6, 120.6, 84.7, 83.7, 72.8, 72.6, 70.5, 70.4, 34.5, 34.1, 32.2, 31.9.

IR (Diamond-ATR, neat) ṽ (cm^{-1}): 3462, 3060, 3028, 2923, 2857, 1499, 1458, 1394, 1204, 1109, 1065, 1030, 998, 932.

HRMS (ESI): m/z: $[M-OH]^+$ calc'd for $C_{19}H_{21}O^+$: 265.1587. Found: 265.1587.

(±)-(3*S*,4*R*)-4-Hydroxy-6-phenylhex-1-en-3-yl benzoate (3q)

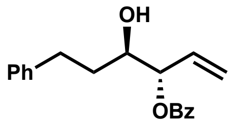

3q

Prepared according to **General Procedure F** from 2-(benzoyloxy)but-3-enoic acid (**SI-9**) (124 mg, 0.60 mmol) and 3-phenylpropanal (**2a**) (40 µL, 0.30 mmol). The residue was purified by flash column chromatography on silica gel (gradient elution: hexanes to 25% EtOAc in hexanes) to yield **3q** as a brown oil and 5:1 mixture of diastereomers (45 mg, 51%).

^{1}H NMR (600 MHz, $CDCl_3$, major diastereomer): δ 8.08–8.05 (m, 2H), 7.60–7.57 (m, 1H), 7.48–7.44 (m, 2H), 7.30–7.26 (m, 2H), 7.22–7.19 (m, 3H), 5.92 (ddd, *J* = 17.1, 10.7, 6.3 Hz, 1H), 5.48–5.46 (m, 1H), 5.43 (dt, *J* = 17.2, 1.2 Hz, 1H), 5.34 (dt, *J* = 10.6, 1.0 Hz, 1H), 3.83 (dtd, *J* = 9.0, 5.4, 3.5 Hz, 1H), 2.90 (ddd, *J* = 13.8, 9.5, 5.3 Hz, 1H), 2.77–2.72 (m, 1H), 1.96 (d, *J* = 5.4 Hz, 1H), 1.94–1.89 (m, 1H), 1.87–1.81 (m, 1H).

^{13}C NMR (151 MHz, $CDCl_3$, major diastereomer): δ 165.9, 141.8, 133.4, 133.2, 130.1, 129.8, 128.7, 128.6, 128.6, 126.1, 119.4, 78.2, 72.2, 34.7, 31.9.

IR (Diamond-ATR, neat) ṽ (cm^{-1}): 3464, 3058, 3023, 2920, 2852, 1717, 1602, 1583, 1501, 1447, 1316, 1175, 1112, 1070, 1026, 990, 927.

HRMS (ESI): m/z: $[M+H]^+$ calc'd for $C_{19}H_{21}O_3^+$: 297.1485. Found: 297.1489.

(±)-((*R*)-3-((*R*)-1-Hydroxy-3-phenylpropyl)-4-methylenepiperidin-1-yl)(phenyl)methanone (3r)

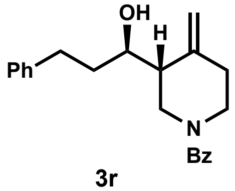

3r

Prepared according to **General Procedure F** from 2-(1-benzoyl-1,2,3,6-tetrahydropyridin-4-yl)acetic acid (**SI-6**) (147 mg, 0.60 mmol) and 3-phenylpropanal (**2a**) (40 µL, 0.30 mmol). The residue was purified according to **General Procedure C** (gradient elution: 40% MeCN in H_2O to 50% MeCN in H_2O) to yield **3r** as a colorless solid and >20:1 mixture of diastereomers (65 mg, 65%).

^{1}H NMR (500 MHz, DMSO-d$_6$, 80 °C): δ 7.43–7.41 (m, 3H), 7.36–7.34 (m, 2H), 7.25 (t, *J*

= 7.6 Hz, 2H), 7.15 (t, *J* = 6.7 Hz, 3H), 4.88 (s, 1H), 4.78 (s, 1H), 4.14 (d, *J* = 4.5 Hz, 1H), 3.91–3.60 (m, 3H), 3.37 (dd, *J* = 13.1, 4.3 Hz, 1H), 3.25–3.20 (m, 1H), 2.80–2.74 (m, 1H), 2.60–2.54 (m, 1H), 2.39 (ddd, *J* = 13.2, 9.5, 5.0 Hz, 1H), 2.27 (q, *J* = 5.2 Hz, 1H), 2.20–2.10 (m, 1H), 1.74 (br s, 1H), 1.54 (br s, 1H).

^{13}C NMR (126 MHz, DMSO-d$_6$, 80 °C): δ 169.0, 145.0, 142.0, 136.1, 128.8, 127.8, 127.8, 127.7, 126.2, 125.0, 110.5, 68.3, 48.7, 35.8, 32.3, 31.2.

IR (Diamond-ATR, neat) ṽ (cm^{-1}): 3410, 3058, 3023, 2913, 2851, 1612, 1576, 1496, 1435, 1270, 1248, 1149, 1074, 1041, 1024, 895.

HRMS (ESI): m/z: [M+H]$^+$ calc'd for $C_{22}H_{26}NO_2^+$: 336.1955. Found: 336.1963.

(±)-*tert*-Butyl 4-((*R*)-hydroxy((*S*)-2-methylenecyclohexyl)methyl)piperidine-1-carboxylate (3s)

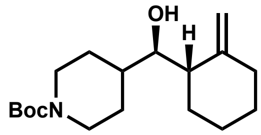

3s

Prepared according to a modified **General Procedure F** from 2-(cyclohex-1-en-1-yl) acetic acid (84 mg, 0.60 mmol) and *tert*-butyl 4-formylpiperidine-1-carboxylate (64 mg, 0.30 mmol). The residue was purified by flash column chromatography on silica gel (gradient elution: CH_2Cl_2 to 12% acetone in CH_2Cl_2) followed by aqueous extraction with $NaHSO_3$ to yield **3s** as a red-brown solid and >20:1 mixture of diastereomers (48 mg, 52%).

^{1}H NMR (600 MHz, CDCl$_3$): δ 4.87 (s, 1H), 4.77 (s, 1H), 3.65 (dd, *J* = 10.0, 1.9 Hz, 1H), 2.25 (dt, *J* = 9.9, 4.4 Hz, 1H), 2.21–2.14 (m, 2H), 1.75–1.65 (m, 6H), 1.60–1.54 (m, 2H), 1.53–1.40 (m, 8H), 1.45 (s, 9H).

^{13}C NMR (151 MHz, CDCl$_3$): δ 154.9, 149.8, 110.7, 79.4, 71.7, 46.6, 37.6, 32.8, 30.0, 28.9, 28.6, 28.1, 22.4.

IR (Diamond-ATR, neat) ṽ (cm^{-1}): 3506, 2927, 2854, 1676, 1644, 1439, 1366, 1315, 1283, 1249, 1173, 1128, 1074, 1014, 983, 948, 881.

HRMS (ESI): m/z: [M+H]$^+$ $C_{18}H_{32}NO_3^+$: 310.2377. Found: 310.2372.

(±)-(*R*)-((*R*)-2-Methylene-1,2,3,4-tetrahydronaphthalen-1-yl)(tetrahydro-2*H*-pyran-4-yl)methanol (3t)

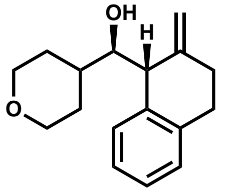

3t

Prepared according to a modified **General Procedure F** from 2-(3,4-dihydronaphthalen-2-yl)acetic acid (**1d**) (141 mg, 0.75 mmol, 2.5 equiv), 4-formyltetrahydropyran (**2e**) (34 mg, 0.30 mmol) and using MeCN (12 mL, 0.025 M) as solvent. The residue was purified by flash column chromatography on silica gel (gradient elution: hexanes to 75% CH_2Cl_2 in hexanes) to yield **3t** as a colorless solid and >20:1 mixture of diastereomers (34 mg, 44%).

^{1}H NMR (600 MHz, $CDCl_3$): δ 7.21–7.16 (m, 2H), 7.15–7.12 (m, 1H), 7.12–7.09 (m, 1H), 5.07 (s, 1H), 4.90 (s, 1H), 4.04 (dd, *J* = 11.4, 4.1 Hz, 1H), 3.98 (dd, *J* = 11.3, 4.0 Hz, 1H), 3.54–3.49 (m, 2H), 3.41 (td, *J* = 12.0, 2.1 Hz, 1H), 3.37 (td, *J* = 11.8, 2.4 Hz, 1H), 3.03–2.98 (m, 1H), 2.87–2.78 (m, 2H), 2.49–2.44 (m, 1H), 1.88–1.84 (m, 1H), 1.81–1.75 (m, 1H), 1.65–1.57 (m, 3H).

^{13}C NMR (151 MHz, $CDCl_3$): δ 146.0, 138.2, 137.2, 129.1, 128.7, 126.8, 126.4, 112.1, 78.1, 68.1, 67.9, 49.9, 36.9, 30.6, 30.5, 30.2, 28.1.

IR (Diamond-ATR, neat) ṽ (cm^{-1}): 3422, 2944, 2916, 2845, 1648, 1494, 1442, 1381, 1239, 1145, 1090, 1057, 1014, 983, 885.

HRMS (ESI): m/z: $[M-OH]^+$ calc'd for $C_{17}H_{21}O^+$: 241.1589. Found: 241.1587.

(±)-(*R*)-5-Chloro-1-((*S*)-2,2-dimethyl-5-methylene-1,3-dioxan-4-yl)pentan-1-ol (3u)

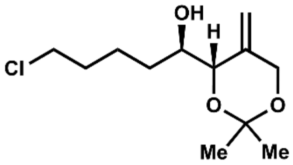

3u

Prepared according to a modified **General Procedure F** from potassium 2-(2,2-dimethyl-4*H*-1,3-dioxin-5-yl)acetate (**1c-K**) (63 mg, 0.30 mmol, 1.0 equiv), 5-chloropentanal (36 mg, 0.30 mmol, 1.0 equiv), $KHCO_3$ (30 mg, 0.30 mmol, 1.0 equiv), and Et_3SiCl (101 µL, 0.60 mmol, 2.0 equiv). The residue was purified by flash column chromatography on silica gel (gradient elution: CH_2Cl_2 to 10% EtOAc in CH_2Cl_2) to yield **3u** as a yellow oil and >20:1 mixture of diastereomers (46 mg, 61%).

^{1}H NMR (600 MHz, C_6D_6): δ 4.61 (s, 2H), 4.25 (dq, *J* = 4.3, 1.6 Hz, 1H), 4.15 (dq, *J* = 13.3, 1.5 Hz, 1H), 3.96 (dt, *J* = 13.2, 1.1 Hz, 1H), 3.61 (dddd, *J* = 9.6, 7.2, 4.2, 2.5 Hz,

1H), 3.13 (t, *J* = 6.7 Hz, 2H), 1.60–1.37 (m, 5H), 1.36–1.30 (m, 1H), 1.36 (s, 3H), 1.31 (s, 3H).

^{13}C NMR (151 MHz, C_6D_6): δ 144.1, 108.1, 99.7, 75.5, 72.6, 64.5, 44.9, 32.8, 31.1, 27.5, 23.6, 23.4.

IR (Diamond-ATR, neat) ṽ (cm^{-1}): 3431, 2988, 2944, 2869, 1452, 1381, 1370, 1221, 1199, 1154, 1079, 904.

HRMS (ESI): m/z: $[M+H]^+$ calc'd for $C_{12}H_{22}ClO_3^+$: 249.1252. Found: 249.1243.

(±)-(3*R*,4*S*)-4,8-Dimethyl-1-(methylthio)-4-vinylnon-7-en-3-ol (3v)

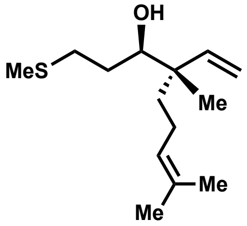

3v

Prepared according to **General Procedure F** from (*E*)-4,8-dimethylnona-3,7-dienoic acid (**1b**) (109 mg, 0.60 mmol) and 3-(methylthio)propanal (30 µL, 0.30 mmol). The residue was purified by flash column chromatography on silica gel (gradient elution: 70% CH_2Cl_2 in hexanes to 84% CH_2Cl_2 in hexanes) to yield **3v** as a yellow oil and 2:1 mixture of diastereomers (63 mg, 87%).

^{1}H NMR (600 MHz, $CDCl_3$, mixture of diastereomers): δ 5.78–5.69 (m, 1H), 5.23–5.15 (m, 1H), 5.09–5.02 (m, 2H), 3.48–3.43 (m, 1H), 2.74–2.67 (m, 1H), 2.61–2.56 (m, 1H), 2.13–2.07 (m, 3H), 1.94–1.75 (m, 3H), 1.67 (s, 3H), 1.61–1.55 (m, 1H), 1.58 (s, 3H), 1.42–1.30 (m, 2H), 1.01–0.97 (m, 3H).

^{13}C NMR (151 MHz, $CDCl_3$, mixture of diastereomers): δ 144.0, 143.4, 131.6, 131.6, 124.9, 124.8, 115.5, 114.9, 76.0, 45.1, 44.9, 37.5, 37.4, 32.2, 32.1, 30.9, 30.3, 25.8, 22.9, 22.8, 17.7, 16.9, 15.7, 15.6.

IR (Diamond-ATR, neat) ṽ (cm^{-1}): 3452, 2913, 2852, 1634, 1440, 1412, 1374, 1065, 1007, 960, 908, 833.

HRMS (ESI): m/z: $[M+H]^+$ calc'd for $C_{14}H_{27}OS^+$: 243.1777. Found: 243.1779.

(±)-*tert*-Butyl 3-((1*S*, 2*S*)-1-hydroxy-6-methoxy-6-oxo-2-vinylhexyl)azetidine-1-carboxylate (3w)

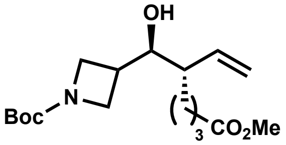

3w

Prepared according to a modified **General Procedure F** from (*E*)-8-methoxy-8-oxooct-3-enoic acid (**SI-10**) (112 mg, 0.60 mmol) and *tert*-butyl 3-formylazetidine-1-carboxylate (56 mg, 0.30 mmol). The residue was purified by flash column chromatography on silica gel (gradient elution: CH_2Cl_2 to 20% acetone in CH_2Cl_2) followed by aqueous extraction with $NaHSO_3$ to yield **3w** as a yellow oil and 4:1 mixture of diastereomers (65 mg, 67%).

^{1}H NMR (600 MHz, $CDCl_3$, mixture of diastereomers): δ 5.65–5.46 (m, 1H), 5.18–5.04 (m, 2H), 3.92–3.83 (m, 3H), 3.73–3.64 (m, 5H), 2.72–2.61 (m, 1H), 2.35–2.29 (m, 2H), 1.99–1.95 (m, 1H), 1.68–1.60 (m, 1H), 1.57–1.52 (m, 1H), 1.46–1.39 (m, 11H).

^{13}C NMR (151 MHz, $CDCl_3$, mixture of diastereomers): δ 174.1, 156.5, 138.0, 137.4, 118.7, 118.0, 79.5, 75.0, 74.7, 51.7, 50.9, 50.0, 48.5, 33.9, 33.9, 32.6, 32.5, 30.7, 30.4, 29.8, 29.6, 28.5, 22.7, 22.5.

IR (Diamond-ATR, neat) ṽ (cm^{-1}): 3443, 2974, 2955, 2933, 2885, 1736, 1701, 1673, 1416, 1394, 1366, 1249, 1166, 1141, 995, 919.

HRMS (ESI): m/z: $[M+H]^+$ calc'd for $C_{17}H_{30}NO_5^+$: 328.2118. Found: 328.2119.

(±)-1,1-Diphenyl-6-(1-vinylcyclohexyl)hexane-1,6-diol (3x)

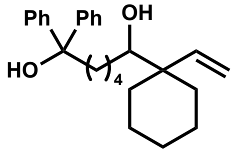

3x

Prepared according to **General Procedure F** from 3-cyclohexylidenepropanoic acid (93 mg, 0.60 mmol) and 6-hydroxy-6,6-diphenylhexanal (**SI-11**) (81 mg, 0.30 mmol). The residue was purified by flash column chromatography on silica gel (gradient elution: CH_2Cl_2 to 10% EtOAc in CH_2Cl_2) to yield **3x** as a colorless oil (101 mg, 89%).

^{1}H NMR (600 MHz, $CDCl_3$): δ 7.40 (d, *J* = 7.9 Hz, 4H), 7.30 (t, *J* = 7.6 Hz, 4H), 7.22 (t, *J* = 7.3 Hz, 2H), 5.56 (dd, *J* = 18.0, 11.1 Hz, 1H), 5.29 (dd, *J* = 11.0, 1.1 Hz, 1H), 5.05 (dd, *J* = 18.0, 1.1 Hz, 1H), 3.13 (dd, *J* = 10.5, 6.6 Hz, 1H), 2.33–2.23 (m, 2H), 2.14 (s, 1H), 1.77 (d, *J* = 12.5 Hz, 1H), 1.63–1.58 (m, 2H), 1.53–1.47 (m, 3H), 1.40–1.22 (m, 9H), 1.20–1.10 (m, 2H).

^{13}C NMR (151 MHz, $CDCl_3$): δ 147.4, 147.2, 142.5, 128.3, 126.9, 126.9, 126.2, 126.2, 117.0, 78.7, 78.4, 45.3, 42.1, 32.1, 32.0, 31.2, 27.5, 26.6, 24.0, 22.3, 22.2.

IR (Diamond-ATR, neat) ṽ (cm^{-1}): 3431, 3051, 3023, 2931, 2855, 1630, 1599, 1496, 1447, 1379, 1168, 1059, 1031, 1002, 913.

HRMS (ESI): m/z: $[M+H]^+$ calc'd for $C_{26}H_{35}O_2^+$: 379.2632. Found: 379.2636.

(±)-4-(1-Hydroxy-2,2-dimethylbut-3-en-1-yl)-2-methoxyphenyl acetate (3y)

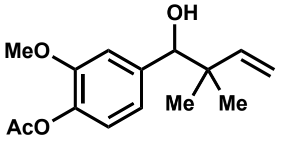

3y

Prepared according to **General Procedure F** from 2,2-dimethylbut-3-enoic acid (72 µL, 0.60 mmol) and 4-formyl-2-methoxyphenyl acetate (58 mg, 0.30 mmol). The residue was purified by flash column chromatography on silica gel (gradient elution: CH_2Cl_2 to 4% EtOAc in CH_2Cl_2) to yield **3y** as a yellow oil (37 mg, 47%).

^{1}H NMR (600 MHz, $CDCl_3$): δ 6.97–6.95 (m, 2H), 6.85 (dd, J = 8.1, 1.8 Hz, 1H), 5.92 (dd, J = 17.6, 10.8 Hz, 1H), 5.14 (dd, J = 10.8, 1.1 Hz, 1H), 5.08 (dd, J = 17.5, 1.1 Hz, 1H), 4.42 (s, 1H), 3.83 (s, 3H), 2.31 (s, 3H), 2.02 (br s, 1H), 1.03 (s, 3H), 0.98 (s, 3H).

^{13}C NMR (151 MHz, $CDCl_3$): δ 169.2, 150.5, 145.1, 139.9, 139.1, 121.8, 120.4, 114.1, 112.1, 80.6, 56.1, 42.4, 24.7, 21.2, 20.9.

IR (Diamond-ATR, neat) ṽ (cm^{-1}): 3522, 2967, 2918, 2864, 1764, 1604, 1508, 1465, 1418, 1369, 1215, 1195, 1146, 1120, 1032, 1010, 908, 862, 833.

HRMS (ESI): m/z: $[M+H]^+$ calc'd $C_{15}H_{21}O_4^+$: 265.1434. Found: 265.1435.

(±)-1-(Benzofuran-5-yl)-2,2-dimethylbut-3-en-1-ol (3z)

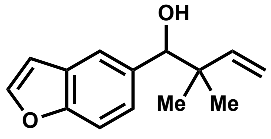

3z

Prepared according to **General Procedure F** from 2,2-dimethylbut-3-enoic acid (71 µL, 0.60 mmol) and benzofuran-5-carbaldehyde (44 mg, 0.30 mmol). The residue was purified by flash column chromatography on silica gel (5% EtOAc in hexanes) to yield **3z** as a yellow oil (30 mg, 46%).

^{1}H NMR (600 MHz, $CDCl_3$): δ 7.61 (d, J = 2.2 Hz, 1H), 7.54 (d, J = 1.7 Hz, 1H), 7.43 (d, J = 8.5 Hz, 1H), 7.24 (dd, J = 8.6, 1.8 Hz, 1H), 6.76 (dd, J = 2.2, 0.9 Hz, 1H), 5.96 (dd, J = 17.6, 10.8 Hz, 1H), 5.16 (dd, J = 10.8, 1.3 Hz, 1H), 5.10 (dd, J = 17.5, 1.3 Hz, 1H), 4.55 (s, 1H), 1.03 (s, 3H), 0.98 (s, 3H).

^{13}C NMR (151 MHz, $CDCl_3$): δ 154.6, 145.4, 145.3, 135.5, 126.9, 124.5, 120.4, 114.0,

110.4, 106.8, 80.9, 42.6, 24.8, 21.2.

IR (Diamond-ATR, neat) ṽ (cm^{-1}): 3433, 3077, 2965, 2927, 2862, 1639, 1538, 1463, 1435, 1189, 1126, 1103, 1030, 911, 887, 822.

HRMS (ESI): m/z: $[M+H]^+$ calc'd for $C_{14}H_{17}O_2^+$: 217.1223. Found: 217.1226.

(±)-1-(1-Allyl-3-methyl-1*H*-pyrazol-4-yl)-2,2-dimethylbut-3-en-1-ol (3aa)

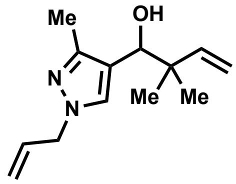

3aa

Prepared according to **General Procedure F** from 2,2-dimethylbut-3-enoic acid (72 µL, 0.60 mmol) and 1-allyl-3-methyl-1*H*-pyrazole-4-carbaldehyde (45 mg, 0.30 mmol). The residue was purified according to **General Procedure C** (gradient elution: 30% MeCN in H_2O to 40% MeCN in H_2O) to yield **3aa** as a colorless solid (65 mg, 98%).

^{1}H NMR (600 MHz, $CDCl_3$): δ 7.30 (s, 1H), 6.00 (ddt, *J* = 17.0, 10.2, 6.0 Hz, 1H), 5.94 (dd, *J* = 17.6, 10.8 Hz, 1H), 5.25 (dq, *J* = 10.2, 1.2, 1H), 5.19 (dq, *J* = 17.1, 1.3, 1H), 5.14 (dd, *J* = 10.8, 1.2 Hz, 1H), 5.10 (dd, *J* = 17.5, 1.1 Hz, 1H), 4.65 (d, *J* = 5.9 Hz, 2H), 4.41 (s, 1H), 2.23 (s, 3H), 1.79 (br s, 1H), 1.01 (s, 3H), 0.99 (s, 3H).

^{13}C NMR (151 MHz, $CDCl_3$): δ 147.0, 145.1, 133.2, 128.4, 119.7, 118.6, 114.1, 73.4, 54.7, 43.0, 24.5, 20.9, 12.7.

IR (Diamond-ATR, neat) ṽ (cm^{-1}): 3309, 3080, 2958, 2923, 2864, 1644, 1557, 1475, 1445, 1419, 1358, 1157, 1133, 993, 911, 822.

HRMS (ESI): m/z: $[M+H]^+$ calc'd for $C_{13}H_{21}N_2O^+$: 221.1648. Found: 221.1649.

(±)-Benzyl ((3*S*,4*R*)-4-hydroxydec-1-en-9-yn-3-yl)carbamate (3ab)

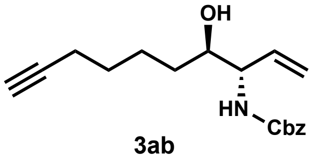

3ab

Prepared according to a modified **General Procedure F** from 2-(((benzyloxy)carbonyl)amino)but-3-enoic acid (106 mg, 0.45 mmol, 1.5 equiv), hept-6-ynal (33 mg, 0.30 mmol, 1.0 equiv), potassium carbonate (42 mg, 0.30 mmol, 1.0 equiv), and Et_3SiCl (101 µL, 0.60 mmol, 2.0 equiv). The residue was purified by flash column chromatography on silica gel (gradient elution: hexanes to 40% EtOAc in hexanes) to yield **3ab** as a yellow oil (60 mg, 67%). The d.r. was assessed by GC-MS of the purified product, showing a 2:1 mixture of diastereomers.

^{1}H NMR (600 MHz, $CDCl_3$, mixture of diastereomers): δ 7.38–7.31 (m, 5H), 5.88–5.74 (m, 1H), 5.31–5.22 (m, 2H), 5.17–5.09 (m, 3H), 4.23 (br s, 1H), 3.71 (br s, 1H), 2.21–2.17 (m, 2H), 1.94 (q, *J* = 2.5 Hz, 1H), 1.63–1.44 (m, 6H).

^{13}C NMR (151 MHz, $CDCl_3$, mixture of diastereomers): δ 156.5, 156.1, 136.6, 136.5, 128.7, 128.4, 128.3, 128.2, 118.4, 116.7, 84.4, 84.4, 73.7, 73.0, 68.7, 68.6, 67.1, 57.9, 57.2, 33.5, 33.1, 28.4, 28.4, 25.0, 25.0, 18.5, 18.4.

IR (Diamond-ATR, neat) ṽ (cm^{-1}): 3426, 3300, 2944, 2862, 1695, 1510, 1454, 1410, 1330, 1217, 1070, 1028, 990, 922.

HRMS (ESI): m/z: $[M+H]^+$ calc'd for $C_{18}H_{24}NO_3^+$: 302.1751. Found: 302.1750.

(±)-2-((2*S*,3*R*)-2-Hydroxy-3-phenylpent-4-en-1-yl)isoindoline-1,3-dione (3ac)

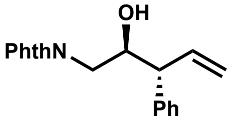

3ac

Prepared according to a modified **General Procedure F** from (*E*)-4-phenylbut-3-enoic acid (97 mg, 0.60 mmol) and 1,3-dioxo-2-isoindolineacetaldehyde (57 mg, 0.30 mmol). The residue was purified by flash column chromatography on silica gel (gradient elution: 20% EtOAc in hexanes to 30% EtOAc in hexanes) followed by aqueous extraction with $NaHSO_3$ to yield **3ac** as a colorless oil and >20:1 mixture of diastereomers (35 mg, 38%).

^{1}H NMR (600 MHz, $CDCl_3$): δ 7.83–7.79 (m, 2H), 7.72–7.68 (m, 2H), 7.35–7.27 (m, 4H), 7.23–7.19 (m, 1H), 6.16 (ddd, *J* = 17.0, 10.3, 8.7, 1H), 5.27–5.20 (m, 2H), 4.29–4.24 (m, 1H), 3.76–3.71 (m, 1H), 3.68–3.64 (m, 1H), 3.40 (t, *J* = 8.0 Hz, 1H), 2.34 (br s, 1H).

^{13}C NMR (151 MHz, $CDCl_3$): δ 168.8, 140.5, 137.6, 134.1, 132.1, 129.0, 128.1, 127.2, 123.4, 118.4, 72.0, 55.7, 42.9.

IR (Diamond-ATR, neat) ṽ (cm^{-1}): 3459, 3054, 3019, 2930, 1770, 1704, 1634, 1597, 1466, 1435, 1393, 1189, 1084, 1070, 1030, 1003, 911, 887.

HRMS (ESI): m/z: $[M+H]^+$ calc'd for $C_{19}H_{18}NO_3^+$: 308.1281. Found: 308.1282.

(±)-1-Phenylhex-5-en-3-ol (3ad)

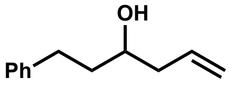

3ad

Prepared according to a modified **General Procedure F** from but-3-enoic acid (51 µL, 0.60 mmol), 3-phenylpropanal (**2a**) (40 µL, 0.30 mmol), and **Cr-1** (63 mg, 0.09 mmol, 30 mol%). A NMR yield was determined by ^{1}H NMR spectroscopy of the crude reaction mixture using CH_2Br_2 as the internal standard (16% ^{1}H NMR yield). An analytically pure

sample of **3ad** was purified by preparatory thin-layer chromatography (CH_2Cl_2) to yield **3ad** as a colorless oil.

^{1}H NMR (600 MHz, $CDCl_3$): δ 7.29 (t, *J* = 7.6 Hz, 2H), 7.22–7.17 (m, 3H), 5.86–5.78 (m, 1H), 5.17–5.15 (m, 1H), 5.14–5.12 (m, 1H), 3.68 (tt, *J* = 7.7, 4.6 Hz, 1H), 2.85–2.78 (m, 1H), 2.73–2.66 (m, 1H), 2.37–2.30 (m, 1H), 2.23–2.15 (m, 1H), 1.84–1.74 (m, 2H).

^{13}C NMR (151 MHz, $CDCl_3$): δ 142.2, 134.7, 128.6, 128.5, 126.0, 118.5, 70.1, 42.2, 38.6, 32.2.

All spectroscopic data for **3ad** was consistent with that which was previously reported.[19]

(±)-(3*R*,4*S*)-1-(3-((5-(4-Fluorophenyl)thiophen-2-yl)methyl)-4-methylphenyl)-4-isopropylhex-5-en-3-ol (3ae)

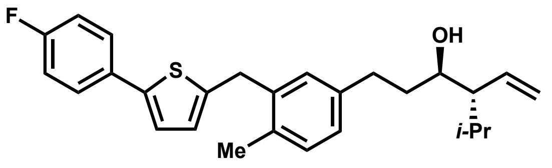

3ae

Prepared according to **General Procedure F** from (*E*)-5-methylhex-3-enoic acid (77 μL, 0.60 mmol) and 3-(3-((5-(4-fluorophenyl)thiophen-2-yl)methyl)-4-methylphenyl)propanal (**SI-12**) (102 mg, 0.30 mmol). A NMR yield was determined by ^{1}H NMR spectroscopy of the crude reaction mixture using 1,1,2,2-tetrachloroethane (TCE) as the internal standard (34% ^{1}H NMR yield). The residue was purified by flash column chromatography on silica gel (gradient elution: CH_2Cl_2 to 10% acetone in CH_2Cl_2) to yield **3ae** as a yellow oil and >20:1 mixture of diastereomers (31 mg, 25%).

^{1}H NMR (600 MHz, $CDCl_3$): δ 7.49–7.45 (m, 2H), 7.09 (d, *J* = 7.7 Hz, 1H), 7.07 (d, *J* = 1.3 Hz, 1H), 7.04–7.00 (m, 4H), 6.66 (d, *J* = 3.6 Hz, 1H), 5.69 (dt, *J* = 17.2, 10.1 Hz, 1H), 5.21 (dd, *J* = 10.3, 2.1 Hz, 1H), 5.07 (dd, *J* = 17.2, 2.1 Hz, 1H), 4.10 (s, 2H), 3.71–3.65 (m, 1H), 2.76 (ddd, *J* = 13.6, 10.0, 5.4 Hz, 1H), 2.65 (ddd, *J* = 13.8, 9.7, 6.6 Hz, 1H), 2.29 (s, 3H), 1.85–1.67 (m, 4H), 0.90 (d, *J* = 6.7 Hz, 3H), 0.81 (d, *J* = 6.7 Hz, 3H).

^{13}C NMR (151 MHz, $CDCl_3$): δ 162.2 (d, *J* = 247.2 Hz), 143.9, 141.6, 140.3, 138.2, 136.4, 133.8, 131.1 (d, *J* = 3.1 Hz), 130.6, 129.9, 127.2 (d, *J* = 8.1 Hz), 127.1, 126.0, 122.8, 119.2, 115.8 (d, *J* = 21.7 Hz), 70.6, 57.0, 37.2, 34.3, 31.7, 27.9, 21.6, 19.2, 19.1.

^{19}F NMR (470 MHz, $CDCl_3$): δ −115.3.

IR (Diamond-ATR, neat) ṽ (cm^{-1}): 3400, 3068, 2958, 2929, 2866, 1602, 1543, 1509, 1468, 1384, 1299, 1232, 1159, 1096, 1044, 1004, 962, 913, 833, 800.

HRMS (ESI): m/z: $[M-OH]^+$ calc'd for $C_{27}H_{30}FS^+$: 405.2047. Found: 405.2045.

(1*R*,2*S*)-2-((1a*R*,3a*R*,3b*S*,5a*S*,6*R*,8a*S*,8b*S*,10*R*,10a*R*)-10-Methoxy-3a,5a-dimethylhexadecahydrocyclopenta[a]cyclopropa[2,3]cyclopenta[1,2-f]naphthalen-6-yl)-1-(1-vinylcyclohexyl)propan-1-ol (3af)

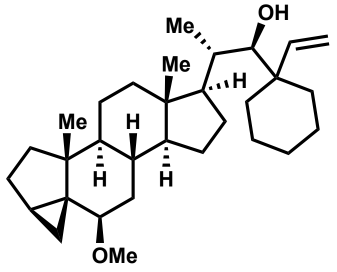

3af

Prepared according to a modified **General Procedure F** from 3-cyclohexylidenepropanoic acid (69 mg, 0.45 mmol, 1.5 equiv), (20*S*)-6β-methoxy-3α,5-cyclo-5α-pregnane-20-carbaldehyde (103 mg, 0.30 mmol, 1.0 equiv), K_2CO_3 (42 mg, 0.30 mmol, 1.0 equiv), and Et_3SiCl (101 µL, 0.60 mmol, 2.0 equiv). The residue was purified by flash chromatography on silica gel (gradient elution: 50% CH_2Cl_2 in hexanes to 75% CH_2Cl_2 in hexanes) to afford **3af** as a colorless oil and 10:1 mixture of diastereomers at C22 (53 mg, 39%).

^{1}H NMR (600 MHz, $CDCl_3$, major diastereomer): δ 5.67 (dd, *J* = 18.0, 11.1 Hz, 1H), 5.29 (dd, *J* = 11.0, 1.2 Hz, 1H), 5.06 (dd, *J* = 18.0, 1.2 Hz, 1H), 3.35–3.29 (m, 4H), 2.77 (t, *J* = 2.8 Hz, 1H), 1.98–1.66 (m, 7H), 1.63–1.54 (m, 3H), 1.53–1.45 (m, 4H), 1.42–1.33 (m, 5H), 1.31–1.05 (m, 8H), 1.02 (s, 3H), 0.90–0.88 (m, 1H), 0.86 (d, *J* = 6.6 Hz, 3H), 0.84–0.78 (m, 2H), 0.73 (s, 3H), 0.64 (t, *J* = 4.4 Hz, 1H), 0.43 (dd, *J* = 7.9, 5.1 Hz, 1H).

^{13}C NMR (151 MHz, $CDCl_3$, major diastereomer): δ 143.2, 116.4, 82.6, 56.7, 56.7, 54.2, 48.0, 45.9, 43.5, 42.9, 40.5, 36.4, 35.4, 35.2, 34.0, 33.7, 33.5, 30.7, 29.0, 26.6, 25.1, 24.3, 22.9, 22.6, 21.9, 21.6, 19.4, 14.4, 13.2, 12.2.

IR (Diamond-ATR, neat) ṽ (cm^{-1}): 2929, 2865, 2854, 1460, 1383, 1322, 1202, 1099, 1086, 1016, 986, 969, 922, 861, 814.

HRMS (ESI): m/z: $[M-OMe]^+$ calc'd for $C_{30}H_{47}O^+$: 423.3621. Found: 423.3619.

Specific Rotation $[\alpha]^{23}_D$: +22.0 (*c* = 1.0, $CHCl_3$).

To determine the relative configuration at C22, efforts to prepare single crystals suitable for X-ray diffraction analysis through derivatization using ferrocenoyl chloride or 3,5-dinitrobenzoyl chloride were unsuccessful. Thus, a Mosher ester analysis was performed according to a modified literature procedure.[20]

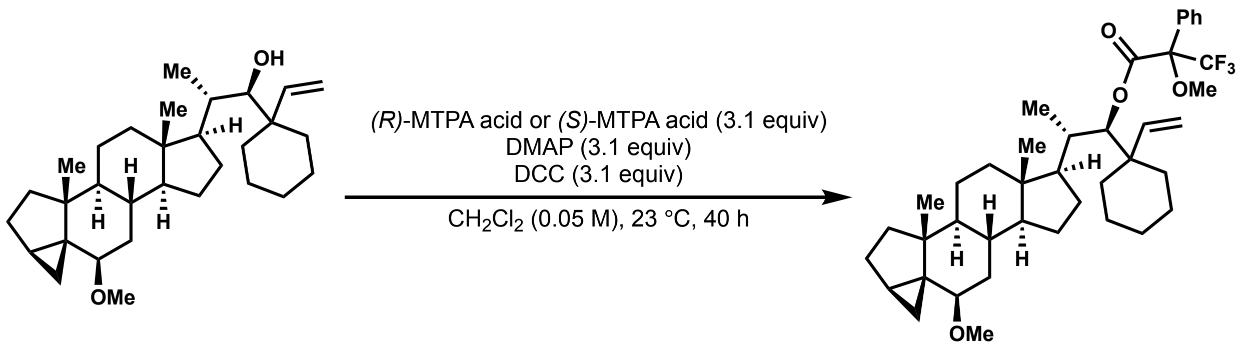

3af **SI-13-R** or **SI-13-S**

To an oven-dried vial equipped with a magnetic stir bar was added DMAP (8.3 mg, 68 μmol, 3.1 equiv) and DCC (14.1 mg, 68 μmol, 3.1 equiv). The vial was sealed with a septum-lined screw cap and evacuated then backfilled with N_2 utilizing a dual manifold Schlenk line. This process was repeated three times. Then a solution of **3af** in CH_2Cl_2 (50 mg/mL, 200 μL, 22 μmol, 1.0 equiv) was added to the mixture followed by a solution of (*R*)- or (*S*)-α-methoxy-α-trifluoromethylphenylacetic acid in CH_2Cl_2 (80 mg/mL, 200 μL, 68 μmol, 3.1 equiv), and the reaction was allowed to stir at room temperature. After 40 hours, the screw cap was removed and the reaction mixture was diluted with CH_2Cl_2 (1 mL), filtered through cotton, and concentrated *in vacuo* with the aid of a rotary evaporator. The residue was purified by preparatory thin-layer chromatography (10% EtOAc in hexanes) to afford the Mosher esters **SI-13-R** and **SI-13-S**, respectively.

(*R*)-MTPA ester derivative (SI-13-R)

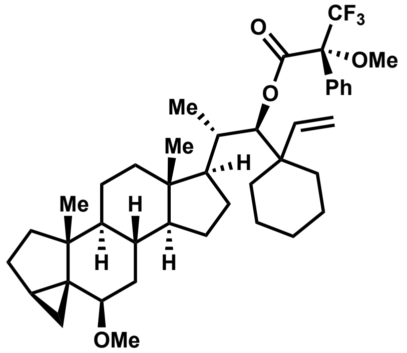

SI-13-R

^{1}H NMR (600 MHz, $CDCl_3$): δ 7.63–7.58 (m, 2H), 7.43–7.39 (m, 3H), 5.59 (dd, *J* = 18.0, 11.1 Hz, 1H), 5.22 (d, *J* = 11.1 Hz, 1H), 5.06 (s, 1H), 4.99 (d, *J* = 17.9 Hz, 1H), 3.54 (s, 3H), 3.32 (s, 3H), 2.77 (t, *J* = 2.6 Hz, 1H), 2.13 (dtd, *J* = 12.7, 9.4, 6.1 Hz, 1H), 1.94–1.90 (m, 1H, 20-CH), 1.88 (dt, *J* = 13.5, 2.9 Hz, 1H), 1.82 (dt, *J* = 12.8, 3.5 Hz, 1H), 1.79–1.75 (m, 1H), 1.73–1.59 (m, 3H), 1.57–1.45 (m, 4H), 1.42–1.21 (m, 8H), 1.18–0.96 (m, 6H), 1.00 (s, 3H), 0.93–0.80 (m, 3H), 0.81 (d, *J* = 6.7 Hz, 3H, 21-CH_3), 0.76 (td, *J* = 10.8, 5.0 Hz, 1H), 0.68 (s, 3H, 18-CH_3), 0.65 (t, *J* = 4.4 Hz, 1H), 0.44 (dd, *J* = 8.0, 5.1 Hz, 1H).

^{13}C NMR (151 MHz, $CDCl_3$): δ 166.9, 142.0, 132.1, 129.7, 128.5, 128.5, 128.0, 123.7 (q,

J = 288.6 Hz), 116.4, 85.3, 84.9 (q, J = 27.6 Hz), 82.5, 56.7, 56.7, 55.7, 54.3, 48.1, 44.8, 43.5, 42.9, 40.3, 36.5, 35.4, 35.1, 33.5, 33.0, 32.7, 30.6, 28.8, 26.2, 25.8, 25.1, 24.2, 22.9, 22.0, 21.7, 21.6, 19.4, 15.4, 13.2, 11.8.

^{19}F NMR (470 MHz, $CDCl_3$): δ −70.7.

IR (Diamond-ATR, neat) ṽ (cm^{-1}): 2927, 2857, 1737, 1454, 1215, 1182, 1171, 1119, 1098, 1077, 1014, 964, 892.

HRMS (ESI): m/z: $[M-OMe]^+$ calc'd for $C_{40}H_{54}F_3O_3^+$: 639.4020. Found: 639.4017.

Specific Rotation $[\alpha]^{23}_D$: +18.6 (c = 1.0, $CHCl_3$).

(*S*)-MTPA ester derivative (SI-13-S)

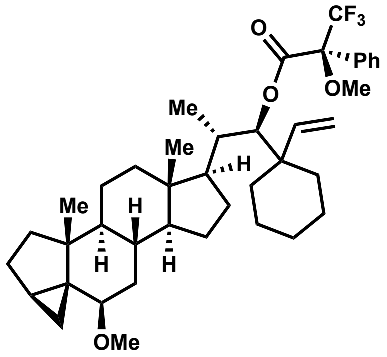

SI-13-S

^{1}H NMR (600 MHz, $CDCl_3$): δ 7.63–7.59 (m, 2H), 7.43–7.39 (m, 3H), 5.58 (dd, J = 17.9, 11.1 Hz, 1H), 5.21 (d, J = 11.1 Hz, 1H), 5.08 (s, 1H), 4.98 (d, J = 17.9 Hz, 1H), 3.54 (s, 3H), 3.32 (s, 3H), 2.77 (t, J = 2.6 Hz, 1H), 2.12 (dtd, J = 12.6, 9.4, 6.2 Hz, 1H), 1.94 (dq, J = 10.6, 6.7 Hz, 1H, 20-CH), 1.87 (dt, J = 13.3, 3.2 Hz, 2H), 1.77 (dtd, J = 11.9, 7.8, 3.9 Hz, 1H), 1.74–1.66 (m, 2H), 1.61 (dddd, J = 12.2, 9.8, 7.0, 2.6 Hz, 1H), 1.54–1.47 (m, 3H), 1.46–1.23 (m, 10H), 1.20–1.05 (m, 4H), 1.04–0.96 (m, 2H), 1.01 (s, 3H), 0.93–0.83 (m, 2H), 0.87 (d, J = 6.7 Hz, 3H, 21-CH_3), 0.79 (td, J = 11.1, 4.6 Hz, 1H), 0.70 (s, 3H, 18-CH_3), 0.65 (t, J = 4.4 Hz, 1H), 0.44 (dd, J = 8.1, 5.1 Hz, 1H).

^{13}C NMR (151 MHz, $CDCl_3$): δ 166.9, 141.9, 132.4, 129.7, 128.5, 127.9, 123.8 (q, J = 288.6 Hz), 116.5, 85.2, 84.8 (q, J = 27.6 Hz), 82.5, 56.7, 56.6, 55.7, 54.4, 48.1, 44.9, 43.5, 42.9, 40.4, 36.5, 35.4, 35.1, 33.5, 32.8, 32.5, 30.7, 28.9, 26.2, 25.1, 24.2, 22.9, 22.0, 21.7, 21.6, 19.4, 15.4, 13.2, 11.9.

^{19}F NMR (470 MHz, $CDCl_3$): δ −70.5.

IR (Diamond-ATR, neat) ṽ (cm^{-1}): 2923, 2848, 1733, 1454, 1374, 1241, 1182, 1166, 1117, 1093, 1077, 1051, 1014, 920, 894, 805.

HRMS (ESI): m/z: $[M-OMe]^+$ calc'd for $C_{40}H_{54}F_3O_3^+$: 639.4020. Found: 639.4020.

Specific Rotation $[\alpha]^{23}_{D}$: +43.7 (*c* = 1.0, $CHCl_3$).

Assignment of the relative configuration at C22 is illustrated in Figure **SI-3**. δ_{SR} values were calculated as $\delta_S - \delta_R$, each representing the chemical shift of analogous pairs of protons in the *S*-MTPA derivative and the *R*-MTPA derivative. Negative δ_{SR} values are observed for the 3 olefinic protons, while positive δ_{SR} values are observed for 18-CH_3, 20-CH, and 21-CH_3.

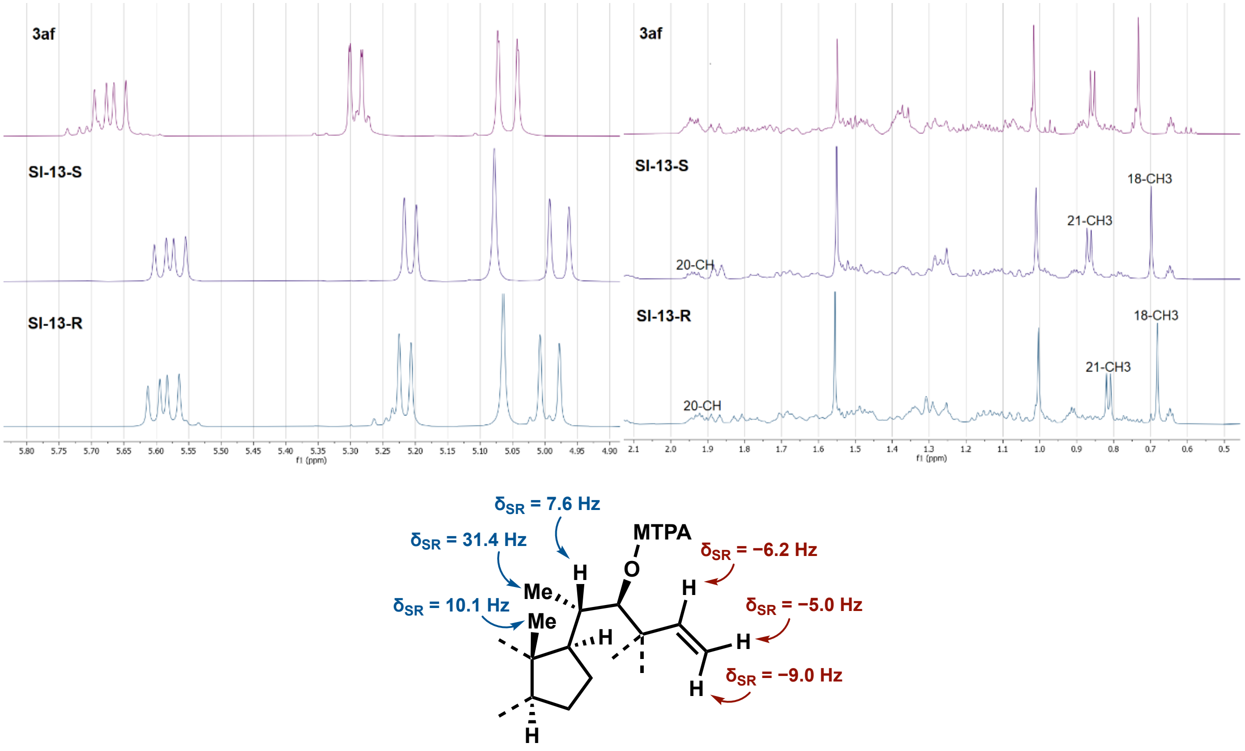

Figure SI-3: δ_{SR} values measured using 600 MHz instrument.

(±)-(1*S*,2*R*)-1-(5-Methylfuran-2-yl)-2-phenylbut-3-en-1-ol (3ag)

(±)-1-(5-Methylfuran-2-yl)-4-phenylbut-3-en-1-ol (3ag')

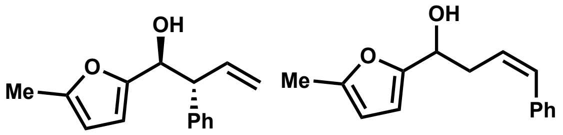

3ag **3ag'**

Prepared according to a modified **General Procedure F** from (*E*)-4-phenylbut-3-enoic acid (97 mg, 0.60 mmol) and 5-methylfuran-2-carbaldehyde (30 μL 0.30 mmol). The residue was purified by flash column chromatography on silica gel (gradient elution: 10% Et_2O in hexanes to 20% Et_2O in hexanes) followed by an aqueous extraction with $NaHSO_3$ to yield a 6:1 mixture of **3ag** and **3ag'** (49 mg, 72%). Analytically pure samples of **3ag**

and **3ag'** were purified by preparatory thin-layer chromatography (50% Et_2O in hexanes) to yield **3ag** as a colorless oil and >20:1 mixture of diastereomers (42 mg, 62%), and **3ag'** as a colorless oil and 2:1 mixture of *Z*/*E* isomers (7 mg, 10%). The configuration of the major olefin isomer of **3ag'** was assigned to be *Z* based on the major *J* coupling constant of the vinyl protons (*ca.* 12 Hz).

Data for 3ag:

^{1}H NMR (600 MHz, $CDCl_3$): δ 7.24 (t, *J* = 7.6 Hz, 2H), 7.18–7.13 (m, 3H), 6.22 (ddd, *J* = 17.1, 10.2, 8.6 Hz, 1H), 5.91 (d, *J* = 3.1 Hz, 1H), 5.77–5.76 (m, 1H), 5.30–5.23 (m, 2H), 4.82 (dd, *J* = 8.1, 3.9 Hz, 1H), 3.82 (t, *J* = 8.4 Hz, 1H), 2.24 (s, 3H), 2.21 (d, *J* = 4.0 Hz, 1H).

^{13}C NMR (151 MHz, $CDCl_3$): δ 152.3, 151.7, 140.7, 138.0, 128.5, 128.2, 126.8, 118.5, 108.6, 106.1, 71.0, 55.9, 13.7.

IR (Diamond-ATR, neat) ṽ (cm^{-1}): 3426, 3028, 2955, 2927, 1789, 1557, 1496, 1449, 1217, 1077, 1016, 922.

HRMS (ESI): m/z: $[M+H]^+$ calc'd for $C_{15}H_{17}O_2^+$: 229.1223. Found: 229.1220.

Data for 3ag':

^{1}H NMR (600 MHz, $CDCl_3$, mixture of *E/Z* isomers): δ 7.36–7.32 (m, 2H), 7.31–7.28 (m, 2H), 7.25–7.20 (m, 1H), 6.60–6.50 (m, 1H), 6.21 (dt, *J* = 15.8, 7.3 Hz, 1H, *E* isomer), 6.16–6.08 (m, 1H), 5.94–5.87 (m, 1H), 5.71 (dt, *J* = 11.8, 7.2 Hz, 1H, *Z* isomer), 4.77–4.72 (m, 1H), 2.95–2.73 (m, 2H), 2.32–2.26 (m, 3H).

^{13}C NMR (151 MHz, $CDCl_3$, mixture of *E/Z* isomers): δ 154.2, 152.1, 137.3, 133.6, 131.7, 128.9, 128.7, 128.4, 127.5, 127.0, 126.3, 125.6, 107.3, 107.2, 106.2, 106.2, 67.8, 67.5, 39.5, 34.8, 32.9, 29.9, 26.3, 26.1, 13.7, 13.7.

IR (Diamond-ATR, neat) ṽ (cm^{-1}): 3389, 3082, 3061, 3021, 2955, 2916, 2850, 1599, 1552, 1491, 1445, 1220, 1077, 1018, 969, 918.

HRMS (ESI): m/z: $[M+H]^+$ calc'd for $C_{15}H_{17}O_2^+$: 229.1223. Found: 229.1223.

(±)-(2*S*,3*R*)-1-(4-((*tert*-Butyldimethylsilyl)oxy)phenyl)-3,7-dimethyl-3-vinyloct-6-en-2-ol (3ah)

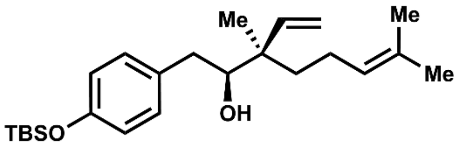

3ah

Prepared according to **General Procedure F** from (*E*)-4,8-dimethylnona-3,7-dienoic acid (**1b**) (109 mg, 0.60 mmol) and 2-(4-((*tert*-butyldimethylsilyl)oxy)phenyl)acetaldehyde (**2b**) (75 mg, 0.30 mmol). The residue was purified by flash column chromatography on silica gel (gradient elution: 25% CH_2Cl_2 in hexanes to 60% CH_2Cl_2 in hexanes) to yield **3ah** as a yellow oil and 2:1 mixture of diastereomers (75 mg, 64%).

^{1}H NMR (600 MHz, $CDCl_3$, mixture of diastereomers): δ 7.08–7.04 (m, 2H), 6.78–6.76 (m, 2H), 5.89–5.82 (m, 1H), 5.22–5.18 (m, 1H), 5.13–5.06 (m, 2H), 3.53–3.48 (m, 1H), 2.84–2.80 (m, 1H), 2.43–2.36 (m, 1H), 1.99–1.87 (m, 2H), 1.68 (s, 3H), 1.60 (s, 3H), 1.53–1.42 (m, 3H), 1.09–1.08 (m, 3H), 0.97 (s, 9H), 0.18 (s, 6H).

^{13}C NMR (151 MHz, $CDCl_3$, mixture of diastereomers): δ 154.3, 144.0, 143.8, 132.4, 132.4, 131.5, 131.5, 130.3, 130.3, 125.0, 124.9, 120.3, 120.2, 114.8, 114.4, 79.0, 78.4, 44.8, 44.6, 37.8, 37.7, 37.6, 37.2, 25.9, 25.8, 22.9, 22.8, 18.3, 17.8, 17.7, 17.6, -4.3.

All spectroscopic data for **3ah** was consistent with that which was previously reported.[21]

(±)-4-((2*S*,3*R*)-2-Hydroxy-3,7-dimethyl-3-vinyloct-6-en-1-yl)phenol (3ai)

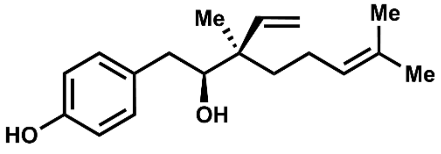

3ai

Prepared according to **General Procedure F** from (*E*)-4,8-dimethylnona-3,7-dienoic acid (**1b**) (109 mg, 0.60 mmol) and 2-(4-hydroxyphenyl)acetaldehyde (**2c**) (41 mg, 0.30 mmol). A NMR yield was determined by ^{1}H NMR spectroscopy of the crude reaction mixture using 1,1,2,2-tetrachloroethane (TCE) as the internal standard (37% ^{1}H NMR yield). The residue was purified by flash column chromatography on silica gel (gradient elution: CH_2Cl_2 to 10% EtOAc in CH_2Cl_2) to yield **3ai** as a yellow solid and 2:1 mixture of diastereomers (25 mg, 30%).

^{1}H NMR (600 MHz, $CDCl_3$, mixture of diastereomers): δ 7.11–7.07 (m, 2H), 6.76 (d, J = 8.4 Hz, 2H), 5.87–5.81 (m, 1H), 5.23–5.19 (m, 1H), 5.13–5.07 (m, 2H), 4.71 (br s, 1H), 3.51–3.45 (m, 1H), 2.84–2.80 (m, 1H), 2.43–2.37 (m, 1H), 1.99–1.87 (m, 2H), 1.68 (s, 3H), 1.60 (s, 3H), 1.55–1.42 (m, 3H), 1.11–1.06 (m, 3H).

^{13}C NMR (151 MHz, $CDCl_3$, mixture of diastereomers): δ 154.2, 143.9, 143.7, 132.1, 132.0, 131.6, 131.5, 130.6, 130.5, 125.0, 124.9, 115.6, 115.5, 115.0, 114.5, 79.1, 78.5, 44.9, 44.6, 37.7, 37.6, 37.0, 25.9, 22.9, 22.8, 17.8, 17.7, 17.5.

IR (Diamond-ATR, neat) ṽ (cm^{-1}): 3345, 2965, 2927, 2876, 2857, 1613, 1594, 1515, 1448, 1372, 1230, 1173, 1103, 1008, 919, 827.

HRMS (ESI): m/z: $[M+H]^+$ calc'd for $C_{18}H_{27}O_2^+$: 275.2006. Found: 275.2002.

(±)-(*R*)-1-((*R*)-2,2-Dimethyl-5-methylene-1,3-dioxan-4-yl)-2,2-dimethoxyethan-1-ol (3aj)

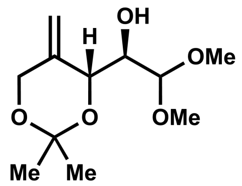

3aj

Prepared according to a modified **General Procedure F** from potassium 2-(2,2-dimethyl-4*H*-1,3-dioxin-5-yl)acetate (**1c-K**) (63 mg, 0.30 mmol, 1.0 equiv), dimethoxyacetaldehyde (**2d**) (142 mg, 0.30 mmol, 1.0 equiv, 22 wt% in MTBE), $KHCO_3$ (30 mg, 0.30 mmol, 1.0 equiv), and Et_3SiCl (101 µL, 0.60 mmol, 2.0 equiv). The residue was purified by flash column chromatography on silica gel (gradient elution: hexanes to 50% EtOAc in hexanes) to yield **3aj** as a yellow oil and >20:1 mixture of diastereomers (38 mg, 54%).

^{1}H NMR (600 MHz, $CDCl_3$): δ 5.15 (s, 1H), 5.02 (q, *J* = 1.3 Hz, 1H), 4.52 (d, *J* = 3.6 Hz, 1H), 4.45 (dd, *J* = 6.0, 1.1 Hz, 1H), 4.34–4.26 (m, 2H), 3.90–3.87 (m, 1H), 3.52 (s, 3H), 3.45 (s, 3H), 2.51 (d, *J* = 4.9 Hz, 1H), 1.47 (s, 3H), 1.39 (s, 3H).

^{13}C NMR (151 MHz, $CDCl_3$, major diastereomer): δ 142.3, 109.6, 103.5, 99.6, 72.9, 72.4, 65.0, 56.3, 55.2, 28.0, 22.7.

All spectroscopic data for **3aj** was consistent with that which was previously reported.[22]

5.1. General Procedure G: Synthesis of 3n through Gram-Scale Decarboxylative Nozaki-Hiyama-Kishi Coupling

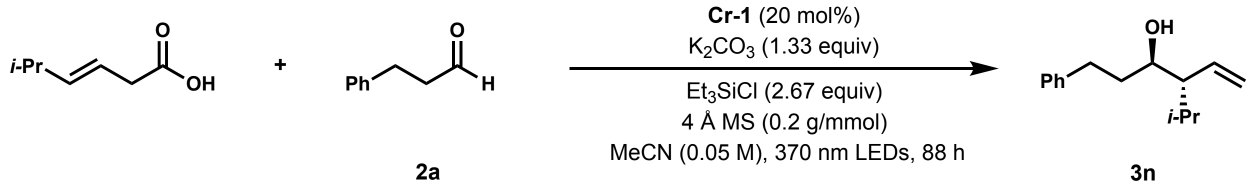

2 equiv | **2a** 5.00 mmol | **3n** 61%, 18:1 dr

Inside a N_2-filled glovebox, a 350 mL pressure vial (Synthware™ round bottom pressure vessel with PTFE bushing) equipped with a magnetic stir bar (SP Bel-Art spin bar, egg-shaped, catalog no. F37130-0112, 38.1 x 15.9 mm) (Figure **SI-4a** left) was charged with K_2CO_3 (919 mg, 6.65 mmol, 1.33 equiv), 4 Å molecular sieves (1.0 g, 0.2 g/mmol), (Figure **SI-4a** right) and MeCN (100 mL, 0.05 M). Et_3SiCl (2.24 mL, 13.4 mmol, 2.67 equiv) was added dropwise via syringe over 1 min under vigorous stirring, and the resulting mixture was allowed to stir at room temperature for 5 min (Figure **SI-4b** left). To the stirred reaction mixture was added [Cr(dtbbpy)$_2$Cl$_2$]Cl (**Cr-1**) (695 mg, 1.00 mmol, 20 mol%), (*E*)-5-methylhex-3-enoic acid (1.28 mL, 10.0 mmol, 2 equiv), and 3-phenyl-propanal (**2a**) (660 µL, 5.00 mmol, 1 equiv) (Figure **SI-4b** right). The reaction vessel was sealed, removed from the glovebox, and placed 4 cm away from four 44 W Kessil PR-160L 370 nm LEDs (Figure **SI-3c**). The reaction mixture was subjected to LED irradiation at 100% intensity with vigorous stirring (Figure **SI-4d**). After 88 h, the reaction mixture was allowed to cool to room temperature, filtered through a plug of packed silica gel (*ca.* 30 g) in a glass fritted funnel eluting with EtOAc (3 x 25 mL) and concentrated *in vacuo* with the aid of a rotary evaporator. The residue was purified by flash column chromatography on silica gel (gradient elution: 15% CH_2Cl_2 in hexanes to CH_2Cl_2) followed by aqueous extraction with $NaHSO_3$ to yield **3n** as a yellow oil and 18:1 mixture of diastereomers (670 mg, 61%).

^{1}H NMR (600 MHz, $CDCl_3$, major diastereomer): δ 7.28 (t, *J* = 7.5 Hz, 2H), 7.22–7.20 (m, 2H), 7.18 (tt, *J* = 7.3, 1.2 Hz, 1H), 5.70 (dt, *J* = 17.1, 10.1 Hz, 1H), 5.23 (dd, *J* = 10.3, 2.2 Hz, 1H), 5.09 (dd, *J* = 17.1, 2.1 Hz, 1H), 3.69 (ddd, *J* = 8.5, 5.3, 3.6 Hz, 1H), 2.81 (ddd, *J* = 13.8, 10.3, 5.3 Hz, 1H), 2.68 (ddd, *J* = 13.7, 10.0, 6.5 Hz, 1H), 1.86–1.69 (m, 4H), 0.91 (d, *J* = 6.7 Hz, 3H), 0.83 (d, *J* = 6.8 Hz, 3H).

^{13}C NMR (151 MHz, $CDCl_3$, major diastereomer): δ 142.4, 136.4, 128.5, 128.4, 125.8, 118.9, 70.6, 56.9, 37.2, 32.2, 27.8, 21.5, 19.2.

IR (Diamond-ATR, neat) ṽ (cm^{-1}): 3411, 3069, 3025, 2955, 2930, 2873, 1496, 1458, 1049, 1005, 916.

HRMS (ESI): m/z: $[M+H]^+$ calc'd for $C_{15}H_{23}O^+$: 219.1743. Found: 219.1739.

5.1.1 Graphical General Procedure G: Gram-Scale Decarboxylative Nozaki-Hiyama-Kishi Coupling

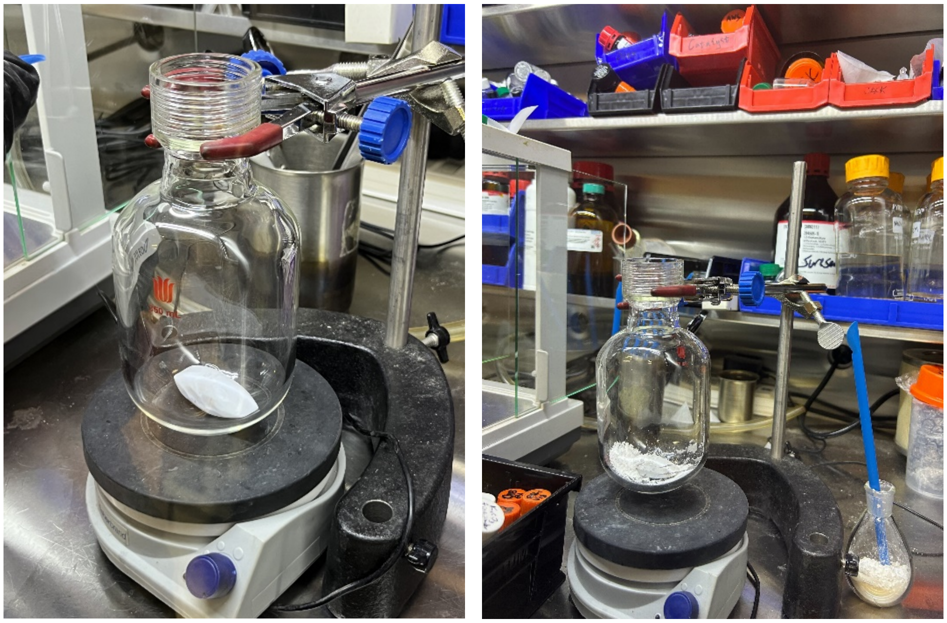

Figure SI-4a: Left: A 350 mL pressure flask equipped with a magnetic stir bar was brought into the glovebox. Right: K_2CO_3 and 4 Å molecular sieves were added.

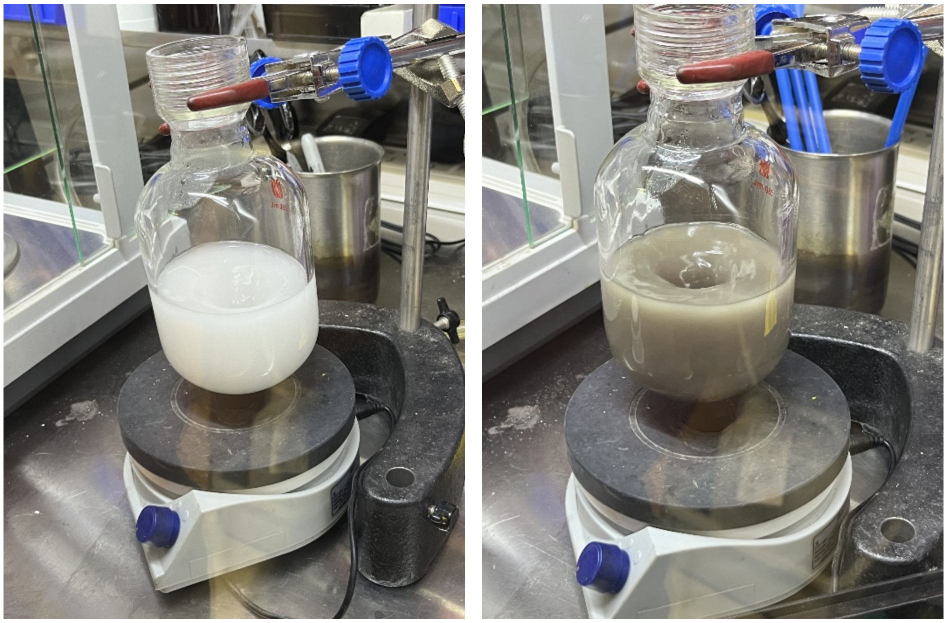

Figure SI-4b: Left: MeCN was added, the reaction mixture was set to vigorous stirring, and Et_3SiCl was added. The reaction mixture was allowed to stir for 5 min. Right: **Cr-1**, (*E*)-5-methylhex-3-enoic acid, and **2a** were added to the reaction mixture under vigorous stirring.

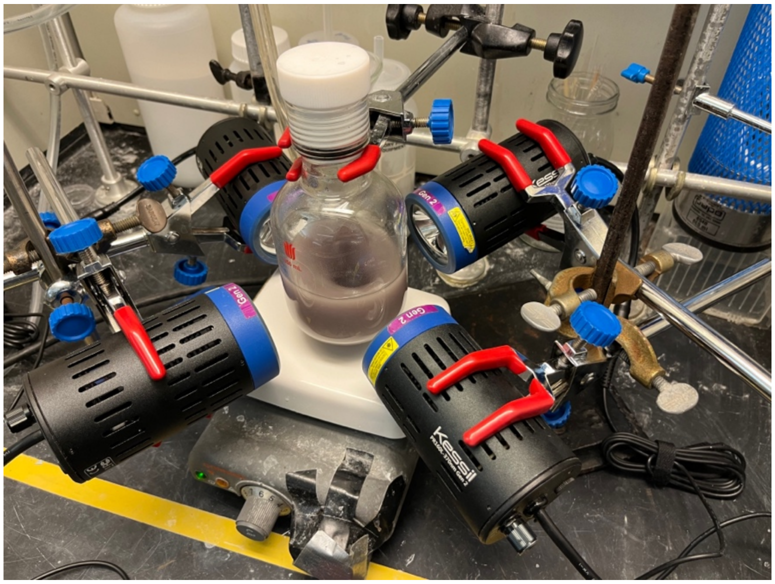

Figure SI-4c: The reaction vessel was sealed, removed from the glovebox, and placed 4 cm away from four 44 W Kessil PR-160L 370 nm LEDs.

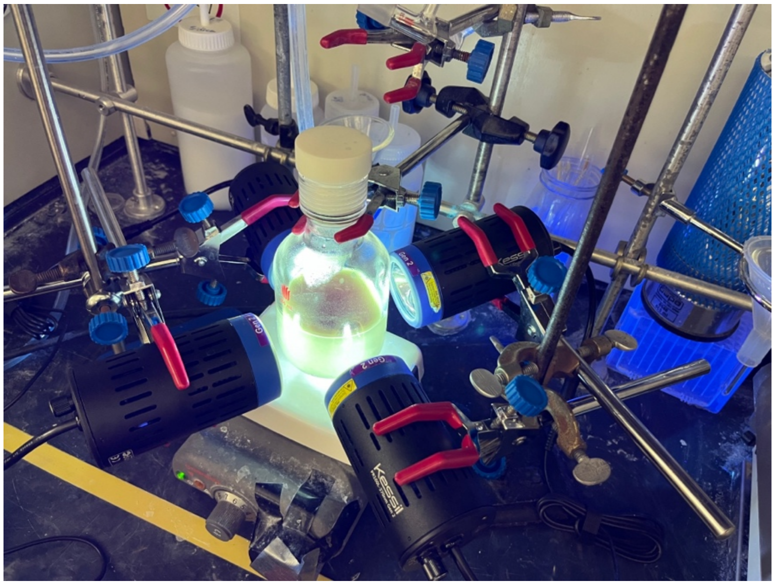

Figure SI-4d: The reaction mixture was subjected to LED irradiation at 100% intensity with vigorous stirring for 88 h.

6. Mechanistic Experiments

6.1. General Procedure H: Mechanistic Experiments

Inside a N_2-filled glovebox, to an oven-dried reaction vial (VWR, catalog no. 66011-041) equipped with a magnetic stir bar (Chemglass Life Sciences, catalog no. CG-2003-160, 10 x 3 mm) was charged K_2CO_3 (18 mg, 0.13 mmol, 1.33 equiv), 4 Å molecular sieves (20 mg, 0.2 g/mmol), and MeCN (2.0 mL, 0.05 M). Et_3SiCl (45 µL, 0.27 mmol, 2.67 equiv) was added via syringe under vigorous stirring, and the resulting mixture was allowed to stir at room temperature for 5 min. To the stirred reaction mixture was added $[Cr(dtbbpy)_2Cl_2]Cl$ (**Cr-1**) (14 mg, 0.02 mmol, 20 mol%), the carboxylic acid (0.20 mmol, 2 equiv), and the aldehyde (0.10 mmol, 1 equiv). The reaction vessel was sealed with a PTFE-lined phenolic vial screw cap (Thermo Scientific, catalog no. 03-375-25A with 03-340-10G), removed from the glovebox, and placed in a custom-made photoreactor 3 cm away from two 44 W Kessil PR-160L 370 nm LEDs and one 75 mm fan. The reaction mixture was subjected to LED irradiation at 100% intensity with vigorous stirring (*ca.* 50 °C). After 40 h, the reaction mixture was allowed to cool to room temperature, then filtered through a short silica plug (*ca.* 1 g) eluting with EtOAc (3 x 2 mL) and concentrated *in vacuo* with the aid of a rotary evaporator. Yield was evaluated by ^{1}H NMR of the crude reaction mixture using 1,1,2,2-tetrachloroethane (TCE) (*ca.* 6 µL) as the internal standard.

Note: vigorous stirring was crucial to ensure reaction mixture homogeneity and maintain a reproducible yield.

6.2. Experiments with [Cr(dtbbpy)$_3$](PF$_6$)$_3$ (Cr-5) and Cr(dtbbpy)Cl$_3$ (Cr-6)

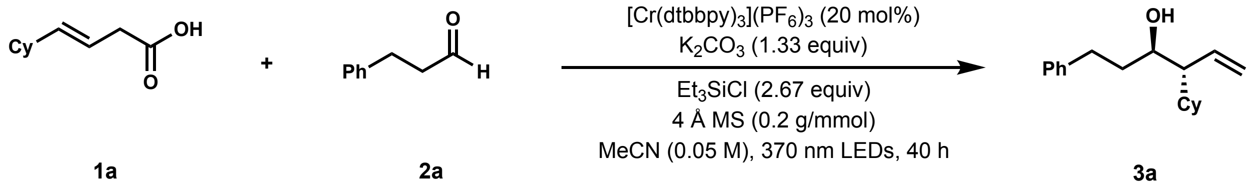

1a 2 equiv; **2a** 1 equiv; **3a** 0%

Prepared according to a modified **General Procedure H** from (*E*)-4-cyclohexylbut-3-enoic acid (**1a**) (34 μL, 0.20 mmol) and 3-phenyl-propanal (**2a**) (13 μL, 0.10 mmol), and using [Cr(dtbbpy)$_3$](PF$_6$)$_3$ (**Cr-5**) (26 mg, 0.02 mmol, 20 mol%) as the catalyst. A NMR yield was determined by ^{1}H NMR spectroscopy of the crude reaction mixture using 1,1,2,2-tetrachloroethane as the internal standard (0% ^{1}H NMR yield).

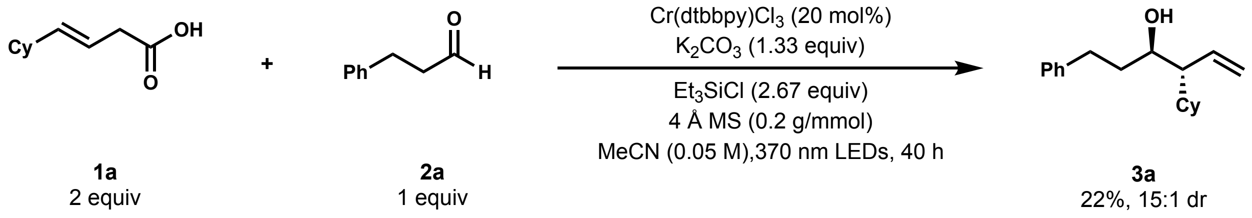

Prepared according to a modified **General Procedure H** from (*E*)-4-cyclohexylbut-3-enoic acid (**1a**) (34 μL, 0.20 mmol) and 3-phenyl-propanal (**2a**) (13 μL, 0.10 mmol), and using Cr(dtbbpy)Cl_3 (**Cr-6**) (9 mg, 0.02 mmol, 20 mol%) as the catalyst. A NMR yield was determined by ^{1}H NMR spectroscopy of the crude reaction mixture using 1,1,2,2-tetrachloroethane (TCE) as the internal standard (22% ^{1}H NMR yield).

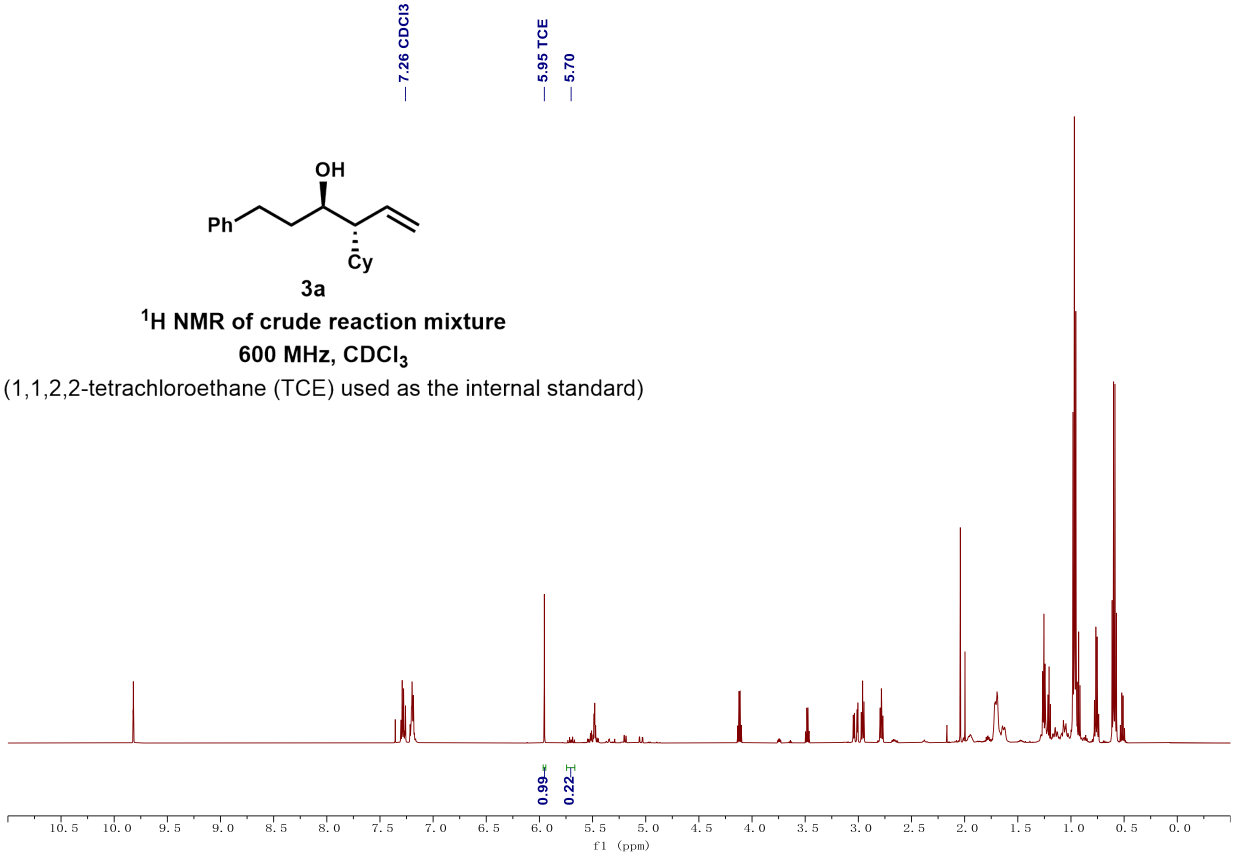

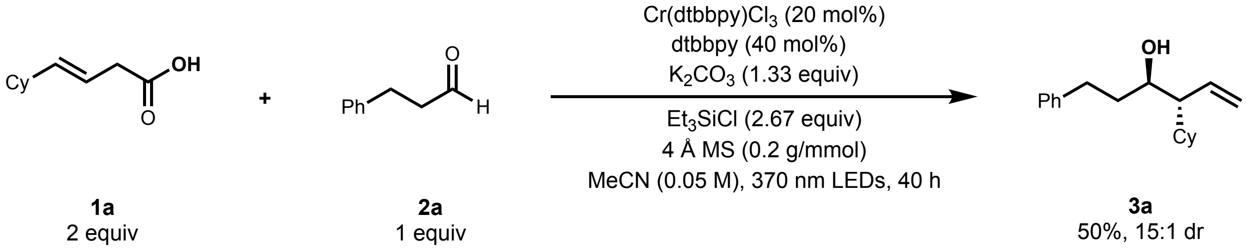

Prepared according to a modified **General Procedure H** from (*E*)-4-cyclohexylbut-3-enoic acid (**1a**) (34 μL, 0.20 mmol) and 3-phenyl-propanal (**2a**) (13 μL, 0.10 mmol), and using Cr(dtbbpy)Cl_3 (**Cr-6**) (9 mg, 0.02 mmol, 20 mol%) as the catalyst and 4,4'-di-*tert*-butyl-2,2'-bipyridine (11 mg, 40 mol%) as the additive. A NMR yield was determined by ^{1}H NMR spectroscopy of the crude reaction mixture using 1,1,2,2-tetrachloroethane (TCE) as the internal standard (50% ^{1}H NMR yield).

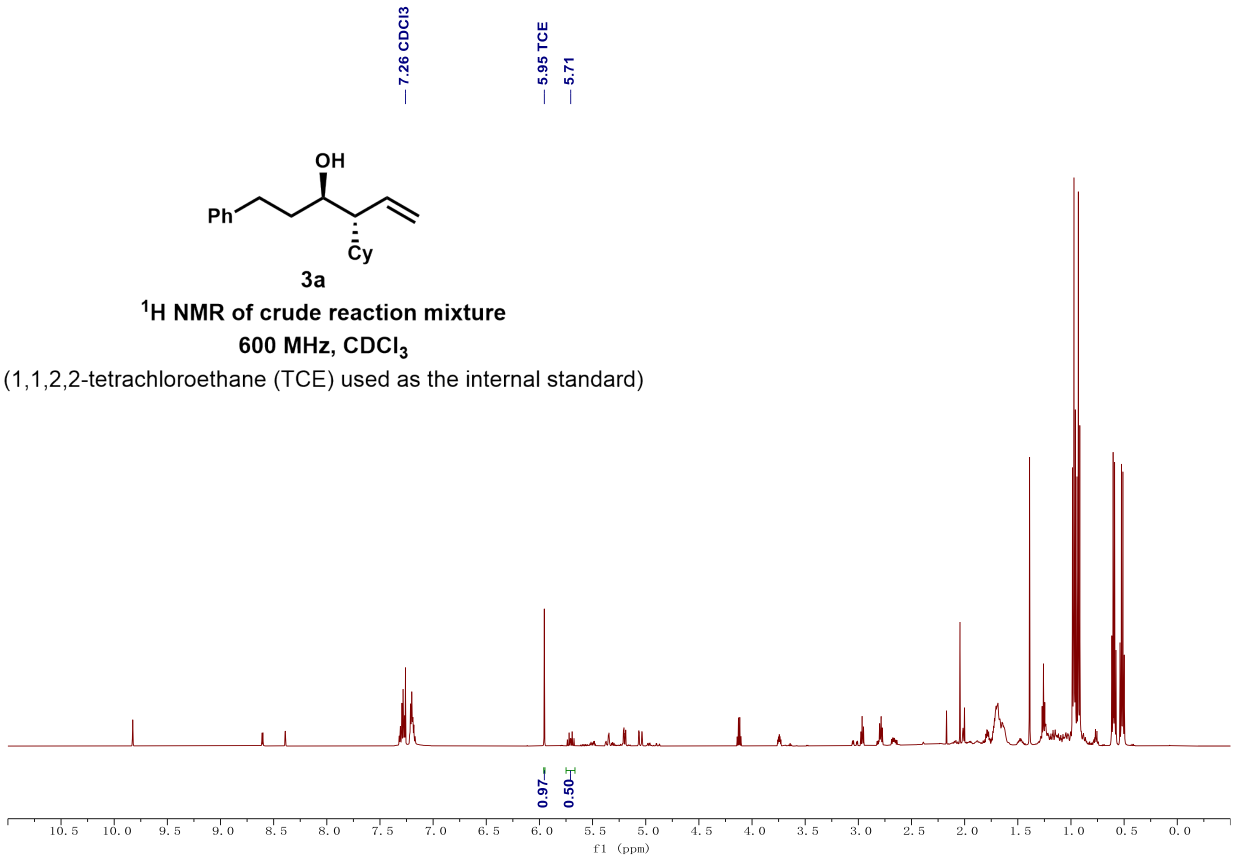

6.3. Decarboxylative LMCT-NHK Experiments with Cr-7

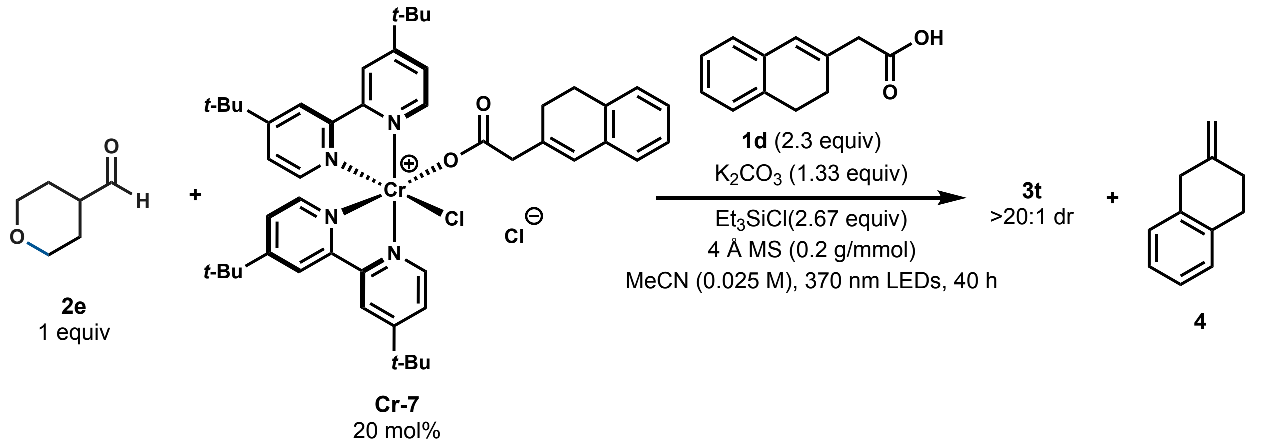

Standard Conditions:

Reactions were performed according to a modified **General Procedure H** using 3,4-dihydronaphthalene-2-carboxylic acid (**1d**) (43 mg, 0.23 mmol, 2.3 equiv) and tetrahydro-2*H*-pyran-4-carbaldehyde (**2e**) (11 mg, 0.10 mmol), with **Cr-7** as the catalyst and MeCN (4 mL, 0.025 M) as the solvent. NMR yields of **3t** and **4** were determined by ^{1}H NMR spectroscopy of the crude reaction mixture using 1,1,2,2-tetrachloroethane (TCE) as the internal standard (entry 1).

The following modifications were made to the standard conditions: without carboxylic acid **1d** (entry 2); without K_2CO_3 (entry 3); without Et_3SiCl (entry 4); without K_2CO_3 and Et_3SiCl (entry 5); with **Cr-7** (17 mg, 0.02 mmol, 1 equiv) and **2e** (5 mg, 0.04 mmol, 2 equiv), no **1d**, K_2CO_3, and Et_3SiCl, and with an irradiation time of 20 h (entry 6).

All spectroscopic data for **3t** was consistent with that which was reported above.

All spectroscopic data for **4** was consistent with that which was previously reported.[23]

entry	*variation from standard conditions*	yield of **3t** (%)	yield of **4** (%)
1	none	29	66
2	no **1d**	6	2
3	no K_2CO_3	0	6
4	no Et_3SiCl	3	8
5	no K_2CO_3 and no Et_3SiCl	6	73
6	no **1d**, K_2CO_3, and Et_3SiCl^a	0	14

Figure SI-5: Decarboxylative LMCT-NHK experiments with monocarboxylate complex **Cr-7**. [a]Reaction performed with **Cr-7** (1 equiv) and **2e** (2 equiv) and with an irradiation time of 20 h. Yield was calculated with respect to **Cr-7**.

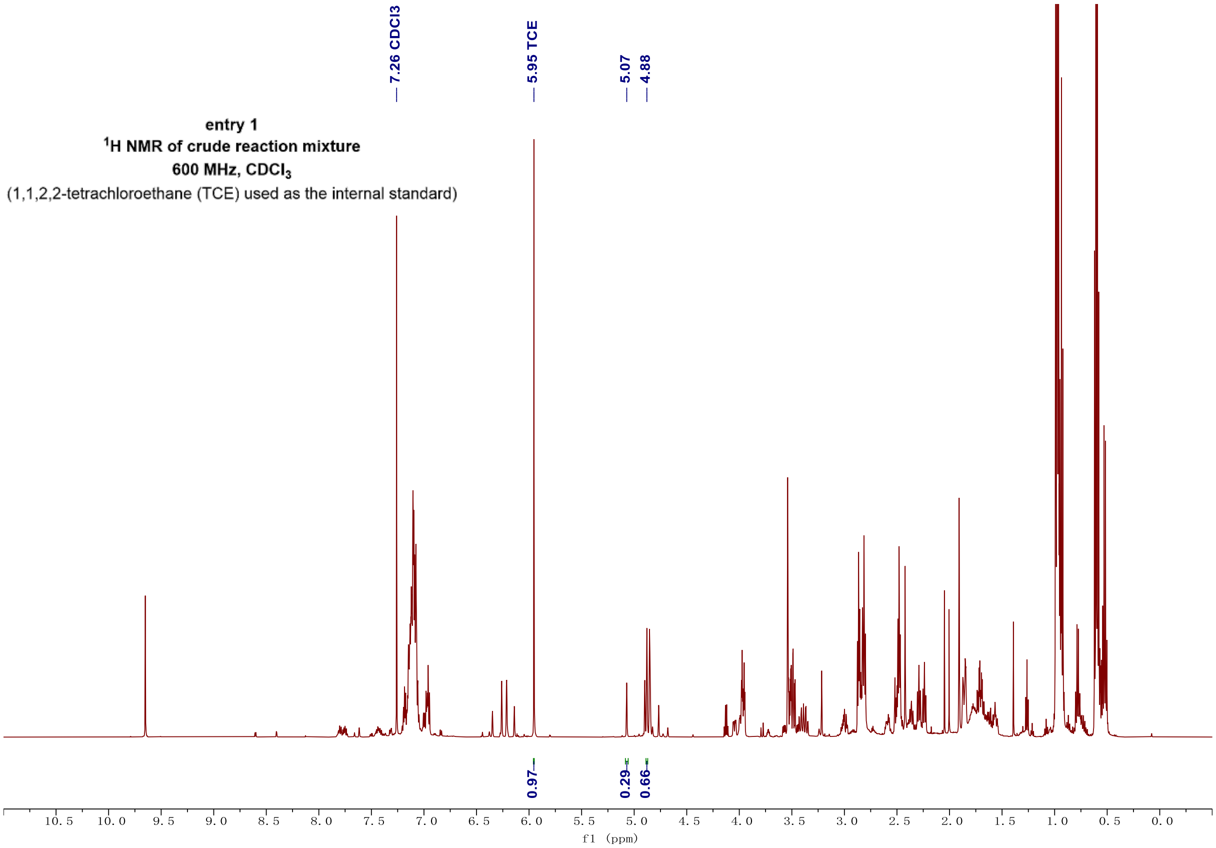
7.26 CDCl3
5.95 TCE
5.07
4.88
entry 1
^{1}H NMR of crude reaction mixture
600 MHz, $CDCl_3$
(1,1,2,2-tetrachloroethane (TCE) used as the internal standard)
0.97
0.29
0.66
10.5 10.0 9.5 9.0 8.5 8.0 7.5 7.0 6.5 6.0 5.5 5.0 4.5 4.0 3.5 3.0 2.5 2.0 1.5 1.0 0.5 0.0
f1 (ppm)

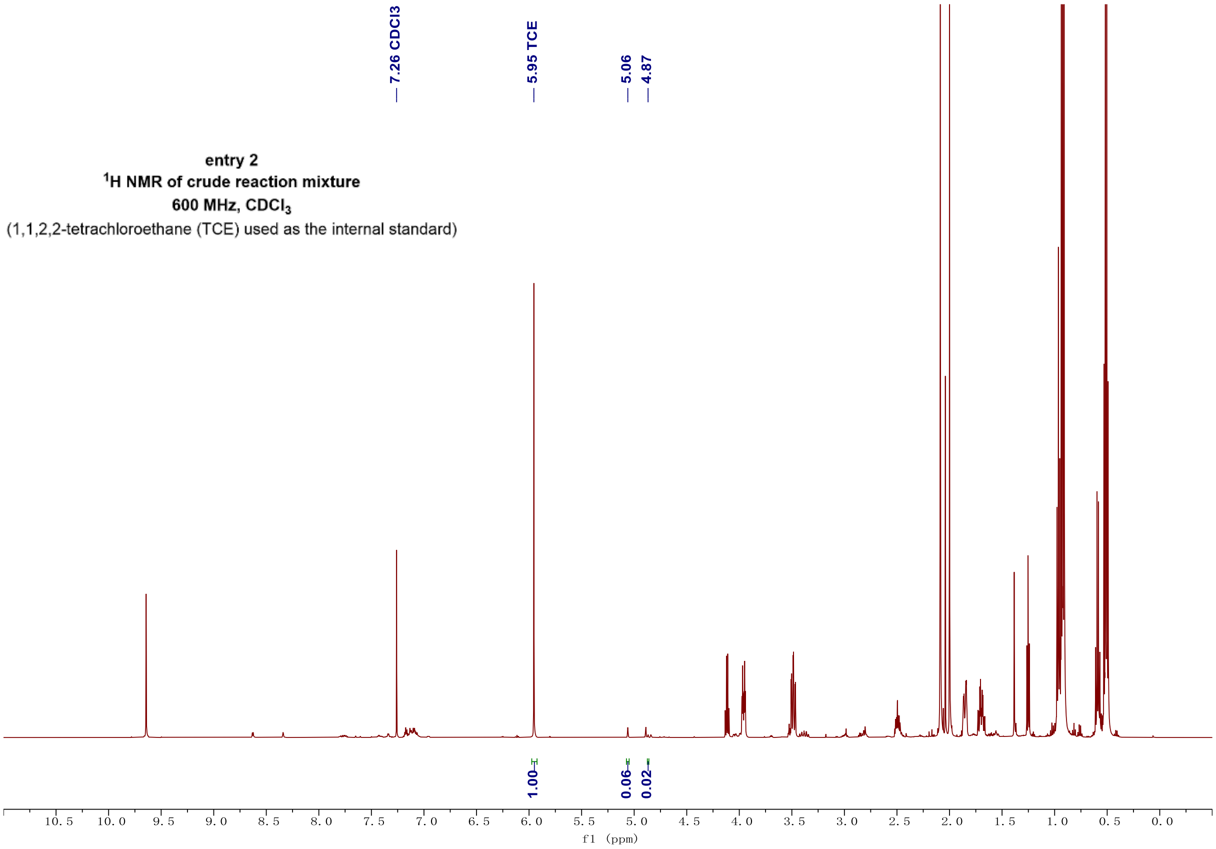
7.26 CDCl3
5.95 TCE
5.06
4.87
entry 2
^{1}H NMR of crude reaction mixture
600 MHz, $CDCl_3$
(1,1,2,2-tetrachloroethane (TCE) used as the internal standard)
1.00
0.06
0.02
10.5 10.0 9.5 9.0 8.5 8.0 7.5 7.0 6.5 6.0 5.5 5.0 4.5 4.0 3.5 3.0 2.5 2.0 1.5 1.0 0.5 0.0
f1 (ppm)

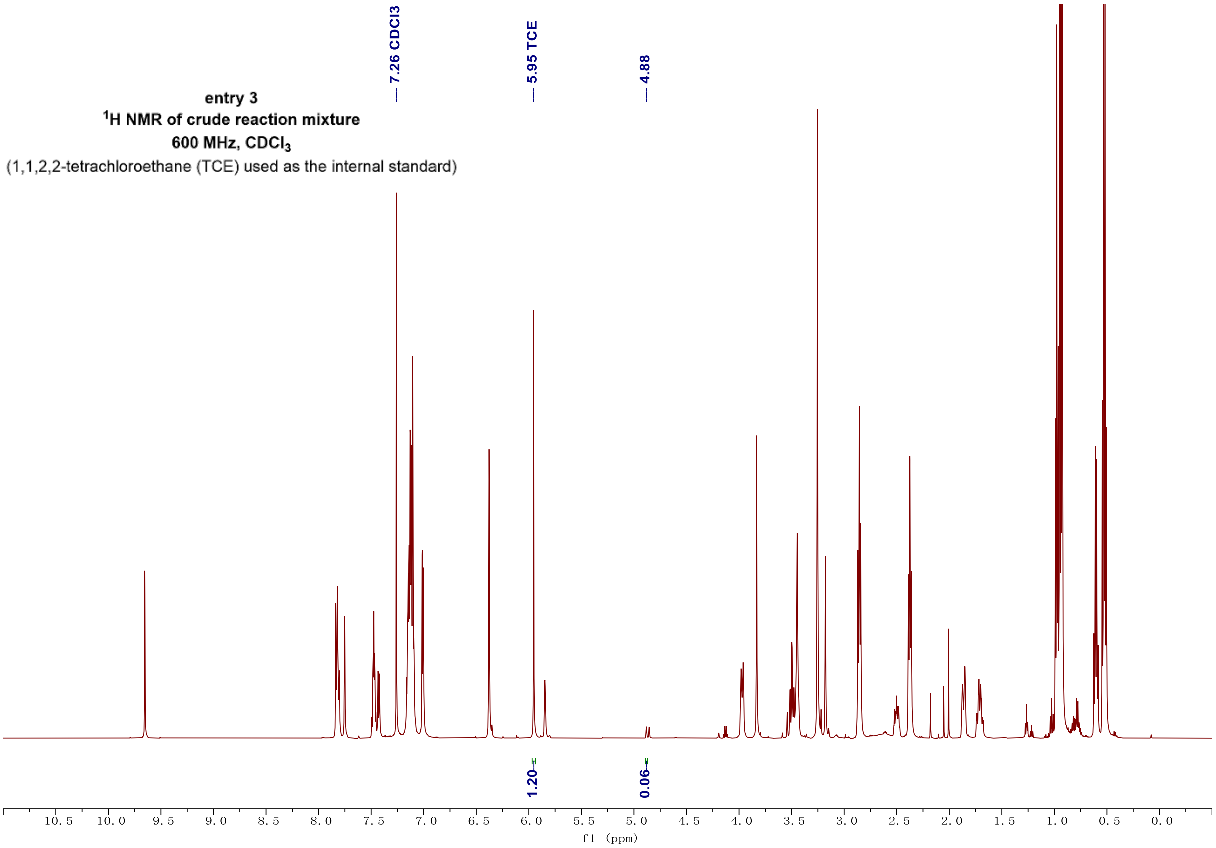
7.26 CDCl3
5.95 TCE
4.88
entry 3
1H NMR of crude reaction mixture
600 MHz, CDCl3
(1,1,2,2-tetrachloroethane (TCE) used as the internal standard)
1.20
0.06
10.5 10.0 9.5 9.0 8.5 8.0 7.5 7.0 6.5 6.0 5.5 5.0 4.5 4.0 3.5 3.0 2.5 2.0 1.5 1.0 0.5 0.0
f1 (ppm)

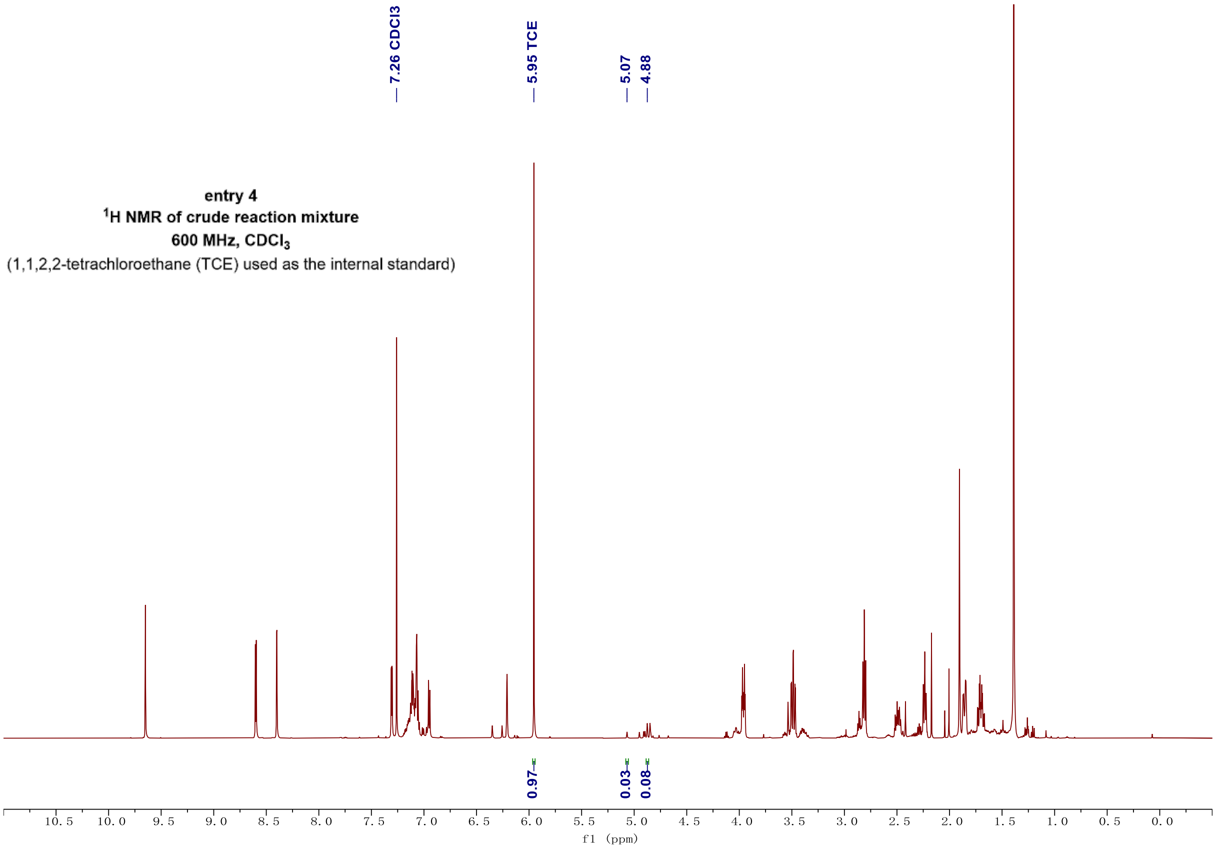
7.26 CDCl3
5.95 TCE
5.07
4.88
entry 4
1H NMR of crude reaction mixture
600 MHz, CDCl3
(1,1,2,2-tetrachloroethane (TCE) used as the internal standard)
0.97
0.03
0.08
10.5 10.0 9.5 9.0 8.5 8.0 7.5 7.0 6.5 6.0 5.5 5.0 4.5 4.0 3.5 3.0 2.5 2.0 1.5 1.0 0.5 0.0
f1 (ppm)

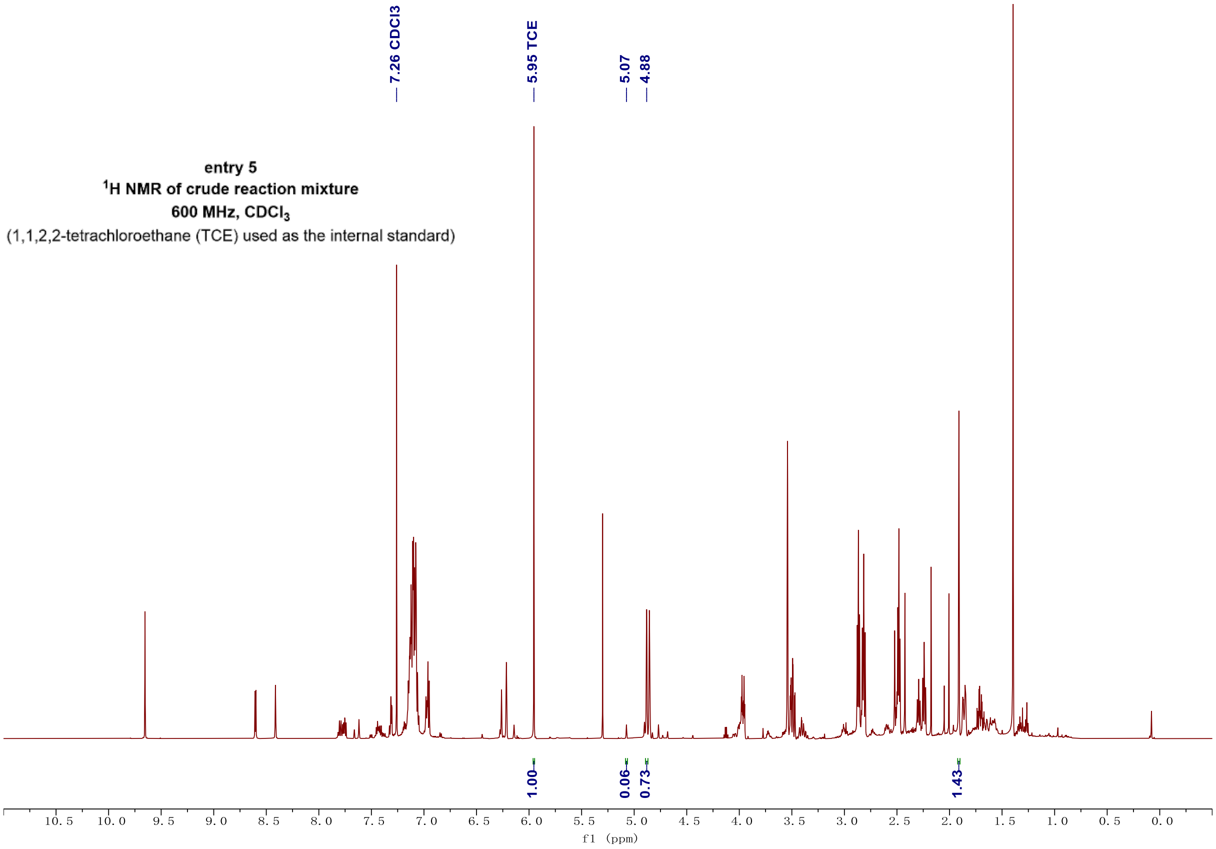

7.26 CDCl3
5.95 TCE
5.07
4.88
entry 5
1H NMR of crude reaction mixture
600 MHz, CDCl3
(1,1,2,2-tetrachloroethane (TCE) used as the internal standard)
1.00
0.06
0.73
1.43
10.5 10.0 9.5 9.0 8.5 8.0 7.5 7.0 6.5 6.0 5.5 5.0 4.5 4.0 3.5 3.0 2.5 2.0 1.5 1.0 0.5 0.0
f1 (ppm)

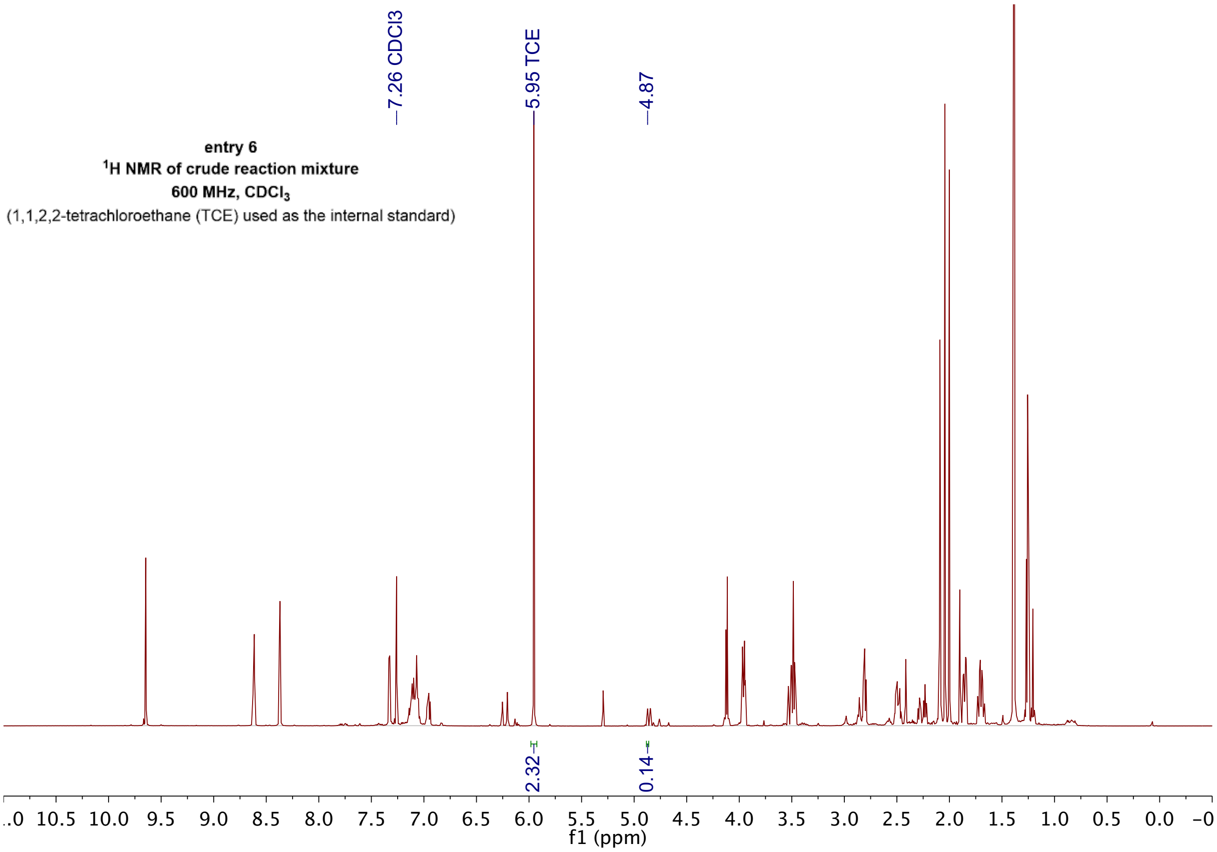

7.26 CDCl3
5.95 TCE
4.87
entry 6
1H NMR of crude reaction mixture
600 MHz, CDCl3
(1,1,2,2-tetrachloroethane (TCE) used as the internal standard)
2.32
0.14
.0 10.5 10.0 9.5 9.0 8.5 8.0 7.5 7.0 6.5 6.0 5.5 5.0 4.5 4.0 3.5 3.0 2.5 2.0 1.5 1.0 0.5 0.0 −0
f1 (ppm)

6.4. Radical Cyclization Experiments

6*H*-Benzo[c]chromen-6-one (5)

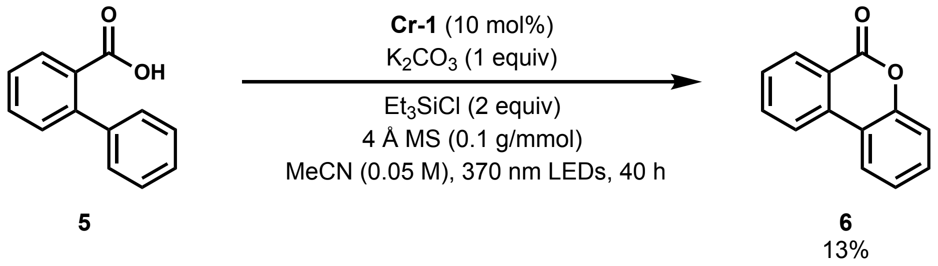

Prepared according to a modified **General Procedure H** from [1,1'-biphenyl]-2-carboxylic acid (**5**) (20 mg, 0.10 mmol, 1.0 equiv), **Cr-1** (7 mg, 0.01 mmol, 10 mol%), K_2CO_3 (14 mg, 0.10 mmol, 1.0 equiv), and Et_3SiCl (34 µL, 0.20 mmol, 2.0 equiv). A NMR yield was determined by ^{1}H NMR spectroscopy of the crude reaction mixture using 1,1,2,2-tetrachloroethane (TCE) as the internal standard (13% ^{1}H NMR yield).

All spectroscopic data for **6** was consistent with that which was previously reported.[24]

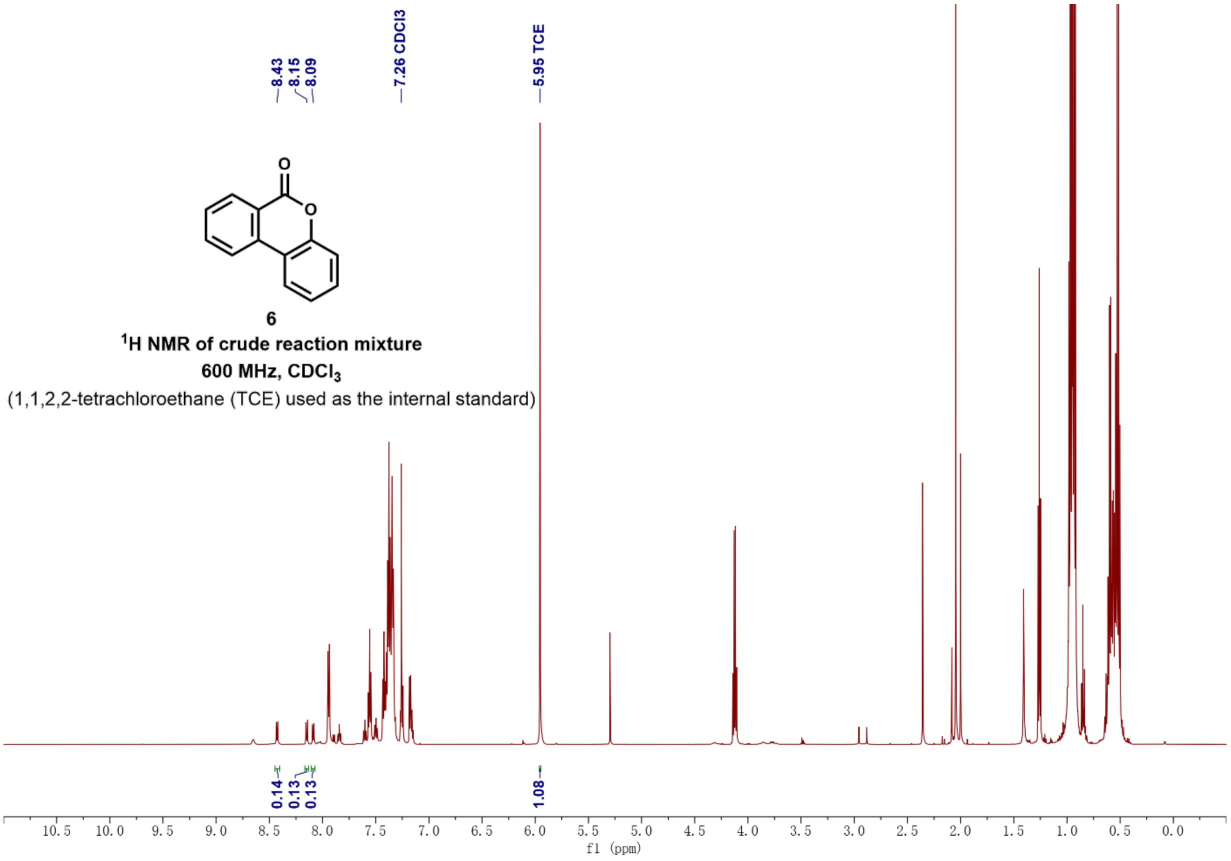

6.5. Radical Trap and Radical Probe Experiments

(±)-(3*R*,4*R*)-4-Cyclopropyl-1-phenylhex-5-en-3-ol (3ak)

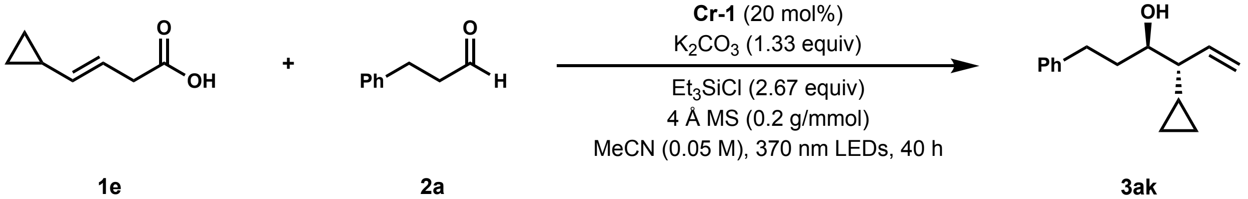

1e 2 equiv **2a** 1 equiv **3ak** 30%, 4:1 dr

Prepared according to **General Procedure H** from (*E*)-4-cyclopropylbut-3-enoic acid (**1e**) (25 mg, 0.20 mmol) and 3-phenyl-propanal (**2a**) (13 µL, 0.10 mmol). A NMR yield was determined by ^{1}H NMR spectroscopy of the crude reaction mixture using 1,1,2,2-tetrachloroethane (TCE) as the internal standard (30% ^{1}H NMR yield). An analytically pure sample of **3ak** was purified by preparatory thin-layer chromatography (50% CH_2Cl_2 in hexanes) and isolated as a colorless oil and 4:1 mixture of diastereomers.

^{1}H NMR (600 MHz, $CDCl_3$, mixture of diastereomers): δ 7.30–7.27 (m, 2H), 7.23–7.20 (m, 2H), 7.20–7.17 (m, 1H), 5.82–5.70 (m, 1H), 5.18–5.08 (m, 2H), 3.70–3.62 (m, 1H), 2.92–2.81 (m, 1H), 2.72–2.64 (m, 1H), 1.99–1.91 (m, 1H), 1.81–1.73 (m, 1H), 1.69 (br s, 1H), 1.42–1.36 (m, 1H), 0.82–0.73 (m, 1H), 0.60–0.45 (m, 2H), 0.31–0.18 (m, 1H), 0.12–0.03 (m, 1H).

^{13}C NMR (151 MHz, $CDCl_3$, mixture of diastereomers): δ 142.6, 142.5, 138.4, 137.9, 128.6, 128.5, 125.9, 125.9, 117.6, 116.9, 74.3, 73.9, 55.0, 36.7, 36.2, 32.6, 32.5, 12.4, 11.5, 5.0, 4.9, 3.2, 2.5.

IR (Diamond-ATR, neat) ṽ (cm^{-1}): 3342, 2925, 2860, 1491, 1454, 1023, 915.

HRMS (ESI): m/z: $[M+H]^+$ calc'd for $C_{15}H_{21}O^+$: 217.1587. Found: 217.1594.

(±)-(3*R*,4*R*)-1-Phenyl-4-(2-phenylcyclopropyl)hex-5-en-3-ol (3al)

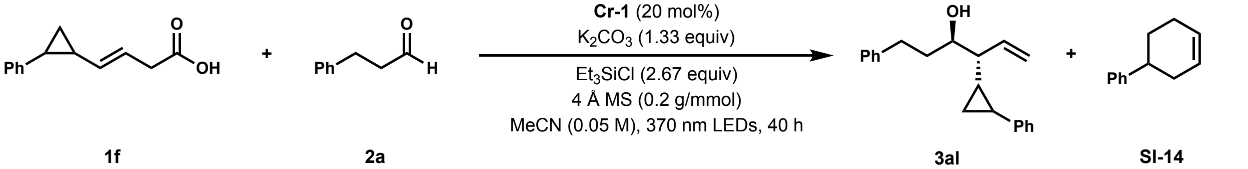

1f 2 equiv **2a** 1 equiv **3al** 0% **SI-14** detected by GC-MS

Prepared according to **General Procedure H** from (*E*)-4-(2-phenylcyclopropyl)but-3-enoic acid (**1f**) (41 mg, 0.20 mmol) and 3-phenyl-propanal (**2a**) (13 µL, 0.10 mmol). A NMR yield was determined by ^{1}H NMR spectroscopy of the crude reaction mixture using 1,1,2,2-tetrachloroethane (TCE) as the internal standard (0% ^{1}H NMR yield). Instead, **SI-**

14, presumably arising from 6-*endo*-trig cyclization of the radical generated from ring-opening (Figure **SI-6**), was detected by GC-MS (through comparison with an independently synthesized sample), suggesting the intermediacy of an allyl radical following decarboxylation.

Note**:** other products with the same m/z ratio as **SI-14** were detected, likely corresponding to constitutional isomers of **SI-14** or products of alternative cyclization modes.

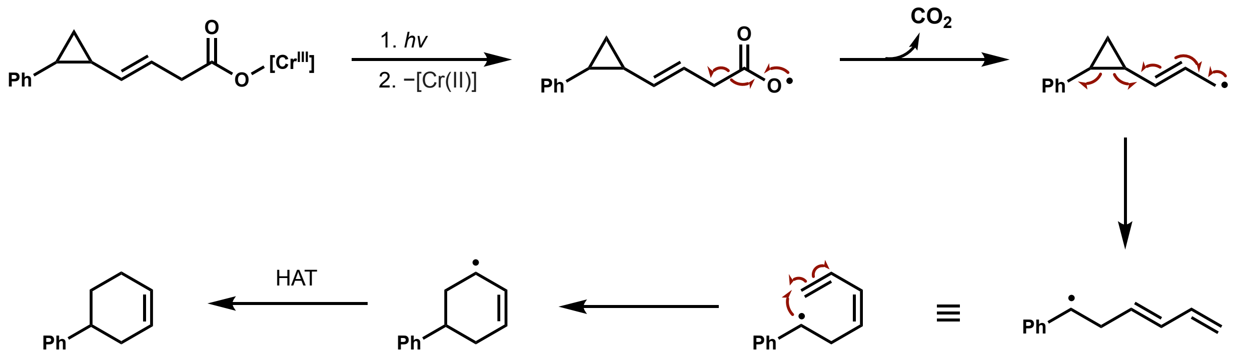

SI-14

Figure SI-6: Plausible mechanism for the formation of **SI-14** from decarboxylation of **1f**.

(±)-(1*R*,2*R*)-2-Cyclohexyl-1-cyclopropylbut-3-en-1-ol (SI-15)

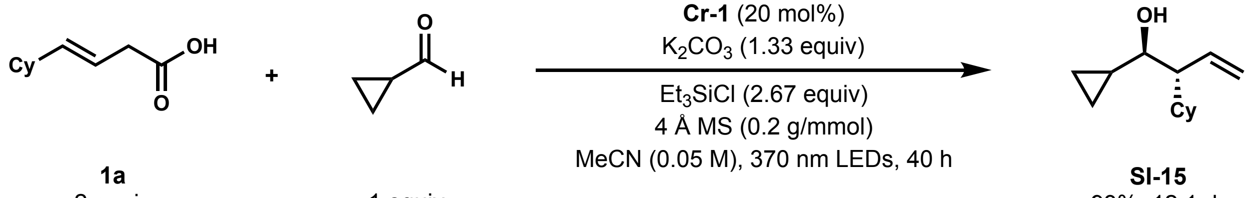

1a 2 equiv; 1 equiv; **SI-15** 93%, 12:1 dr

Prepared according to **General Procedure H** from (*E*)-4-cyclohexylbut-3-enoic acid (**1a**) (33 μL, 0.20 mmol) and cyclopropanecarbaldehyde (7 μL, 0.10 mmol). A NMR yield was determined by ^{1}H NMR spectroscopy of the crude reaction mixture using 1,1,2,2-tetrachloroethane (TCE) as the internal standard (93% ^{1}H NMR yield). An analytically pure sample of **SI-15** was purified by preparatory thin-layer chromatography (10% EtOAc in hexanes) and isolated as a colorless oil and 12:1 mixture of diastereomers.

^{1}H NMR (600 MHz, $CDCl_3$, major diastereomer): δ 5.79 (dt, *J* = 17.2, 10.2 Hz, 1H), 5.19 (dd, *J* = 10.1, 2.4 Hz, 1H), 5.07 (dd, *J* = 17.1, 2.2 Hz, 1H), 2.94 (dd, *J* = 9.0, 4.9 Hz, 1H), 1.91 (ddd, *J* = 9.9, 6.8, 5.0 Hz, 1H), 1.72–1.58 (m, 8H), 1.21–1.08 (m, 2H), 1.02–0.87 (m, 3H), 0.55–0.49 (m, 2H), 0.33–0.30 (m, 1H), 0.22–0.19 (m, 1H).

^{13}C NMR (151 MHz, $CDCl_3$, major diastereomer): δ 137.6, 118.2, 76.2, 56.9, 37.9, 31.8, 30.5, 26.8, 26.7, 26.6, 3.2, 2.9.

IR (Diamond-ATR, neat) ṽ (cm^{-1}): 3405, 3075, 2995, 2918, 2850, 1702, 1630, 1454, 1410, 1058, 1039, 1018, 997, 911, 824.

HRMS (ESI): m/z: $[M+H]^+$ calc'd for $C_{13}H_{23}O^+$: 195.1743. Found: 195.1745.

6.6. Quantum Yield Measurement using Ferrioxalate Actinometry

Preparation of Potassium Ferrioxalate:

In a dark room, to a 125 mL Erlenmeyer flask equipped with a magnetic stir bar was added $K_2C_2O_4 \cdot H_2O$ (13.8 g) and H_2O (70 mL). The flask was wrapped with aluminum foil and anhydrous $FeCl_3$ (4.0 g) was added. The solution was allowed to stir in the dark room for 30 min, then heated to reflux and allowed to slowly cool to room temperature. The flask was then placed in a 0 °C ice-water bath for 30 min to allow the product to crystallize. Then the solution was filtered through a glass fritted funnel and the solids were washed with H_2O (20 mL) and MeOH (10 mL), then dried *in vacuo* overnight to afford potassium ferrioxalate as a light green solid (5.5 g), which was used without further purification.

Preparation of the Actinometric Solution:

In a dark room, to a 125 mL amber bottle wrapped with aluminum foil was added 1 N H_2SO_4 (4 mL) and H_2O (37 mL). Then potassium ferrioxalate (3.0 g) was added, and the bottle was capped and shaken vigorously for 5 min. We performed the quantum yield measurement immediately after preparation of the solution.

Preparation of Buffer Solution:

2 mL aliquots of the buffer solution were prepared by mixing 5 µL of a NaOAc buffer solution (prepared by mixing 4.1 g NaOAc and 3.6 mL 1 N H_2SO_4 in 46 mL H_2O), 200 µL of phenanthroline solution (prepared by mixing 25 mg phenanthroline and 25 mL H_2O), and 1.8 mL H_2O.

Determination of Photon Flux in the Model Setup:

In a dark room, 1.0 mL of the actinometric solution was pipetted into a 4 mL vial equipped with a magnetic stir bar. A 10 µL aliquot was extracted using a gas-tight microliter syringe and dispensed into a 2 mL aliquot of buffer solution, and the solution was wrapped in aluminum foil and allowed to stand for 1 hour. A 500 µL aliquot of this solution was diluted to 2.0 mL with H_2O and the absorbance of this solution at 510 nm was measured to be 0.051.

The vial containing the actinometric solution was then placed in a photoreactor and irradiated with one 44 W Kessil PR-160L 370 nm LED at 25% intensity at distances of 5 cm for exactly 30 seconds with stirring. After this time, the vial was immediately wrapped in aluminum foil, a 10 µL aliquot was extracted using a gas-tight microliter syringe and dispensed into a 2 mL aliquot of buffer solution. This solution was thoroughly mixed, wrapped in aluminum foil and allowed to stand for 1 hour. A 500 µL aliquot of this solution was diluted to 2.0 mL with H_2O and the absorbance of this solution at 510 nm was measured to be 0.213.

The photon flux of the model setup was determined using the formulas below:

$$mol\ (Fe^{2+}) = \frac{\frac{2\ mL}{500\ \mu L} \times \frac{1\ mL}{10\ \mu L} \times 2\ mL \times \Delta A}{l \times \epsilon}$$

$$Photon\ flux = \frac{mol\ (Fe^{2+})}{\phi(Fe^{2+}) \times t \times f}$$

Where ΔA is the difference in absorbance between the irradiated and unirradiated sample, *l* is the path length (1 cm), and ε is the molar absorptivity of $[Fe(phen)_3]^{2+}$ at 510 nm (11100 L/mol·cm), Φ is the quantum yield for the ferrioxalate actinometer (1.210 at λ = 364 nm), *t* is the time of irradiation (s), and *f* ≈ 1.[25] Using this formula, the photon flux in the model setup was calculated to be 3.22×10^{-7} einstein/s.

<u>Determination of LMCT Quantum Yield with Monocarboxylate Complex **Cr-7**:</u>

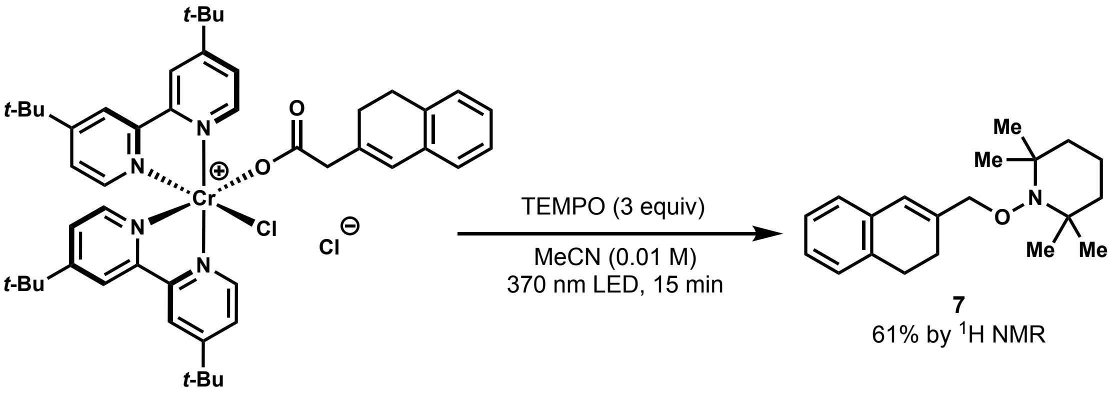

Cr-7

7
61% by ^{1}H NMR

In a N_2-filled glovebox, to an identical, oven-dried 4 mL vial equipped with a magnetic stir bar was added TEMPO (4.7 mg, 30 μmol, 3.0 equiv) and a solution of monocarboxylate complex **Cr-7** in MeCN (0.01 M, 1.0 mL, 10 μmol, 1.0 equiv). The solution was then irradiated with one 44 W Kessil PR-160L 370 nm LED at 25% intensity at distances of 5 cm. After 15 min, the vial was removed from the photoreactor, quenched with 3 drops of AcOH, then the mixture was filtered through a short plug of silica gel (*ca.* 1 g) eluting with EtOAc (3 x 1 mL), and concentrated *in vacuo* with the aid of a rotary evaporator. A NMR yield was determined by ^{1}H NMR spectroscopy of the crude reaction mixture using 1,1,2,2-tetrachloroethane as the internal standard (61% ^{1}H NMR yield) The absorbance of the reaction mixture at 370 nm before subjection to irradiation was determined to be 2.504.

Quantum yield of the reaction was determined using the formula below:

$$Quantum\ yield = \frac{yield \times 0.01\ mmol}{3.22 \times 10^{-7}\ mol/s\ \times t \times (1 - 10^{-2.504})}$$

Where yield is in % and *t* is the time of irradiation (s). Using this formula, the quantum yield of the LMCT reaction of **7** was calculated to be Φ = 0.021.

An analytically pure sample of **7** was purified by preparatory thin-layer chromatography (10% EtOAc in hexanes) to yield **7** as a colorless solid.

1-((3,4-Dihydronaphthalen-2-yl)methoxy)-2,2,6,6-tetramethylpiperidine (7)

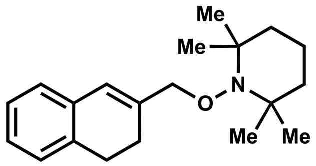

7

^{1}H NMR (600 MHz, $CDCl_3$): δ 7.15–7.08 (m, 3H), 7.04 (d, *J* = 7.3 Hz, 1H), 6.45 (s, 1H), 4.38 (s, 2H), 2.83 (t, *J* = 8.1 Hz, 2H), 2.30 (t, *J* = 8.1 Hz, 2H), 1.62–1.57 (m, 1H), 1.50–1.44 (m, 4H), 1.37–1.31 (m, 1H), 1.21 (s, 6H), 1.15 (s, 6H).

^{13}C NMR (151 MHz, $CDCl_3$): δ 138.1, 135.1, 134.5, 127.4, 126.7, 126.6, 126.1, 122.9, 80.0, 60.1, 39.9, 33.2, 27.9, 25.1, 20.4, 17.3.

IR (Diamond-ATR, neat) ṽ (cm^{-1}): 3002, 2974, 2925, 2866, 1484, 1470, 1454, 1372, 1356, 1246, 1133, 1030, 960, 878, 852, 801.

HRMS (ESI): m/z: $[M+H]^+$ calc'd for $C_{20}H_{30}NO^+$: 300.2322. Found: 300.2323.

6.7. Probing Chlorine Radical HAT Pathway

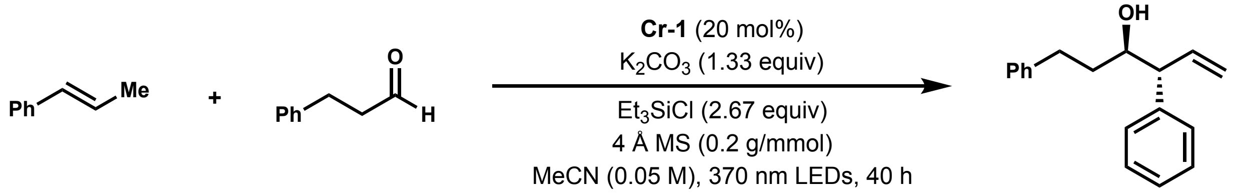

8 (2 equiv) **2a** (1 equiv) **3am** 0%

To rule out alternative mechanisms such as chlorine radical mediated hydrogen atom abstraction pathways,[26] an experiment was set up according to a modified **General Procedure H** utilizing *trans*-β-methyl styrene (**8**) (26 μL, 0.20 mmol, 2.0 equiv) and 3-phenylpropanal (**2a**) (13 μL, 0.10 mmol, 1.0 equiv). A NMR yield was determined by ^{1}H NMR spectroscopy of the crude reaction mixture using 1,1,2,2-tetrachloroethane (TCE) as the internal standard (0% ^{1}H NMR yield).

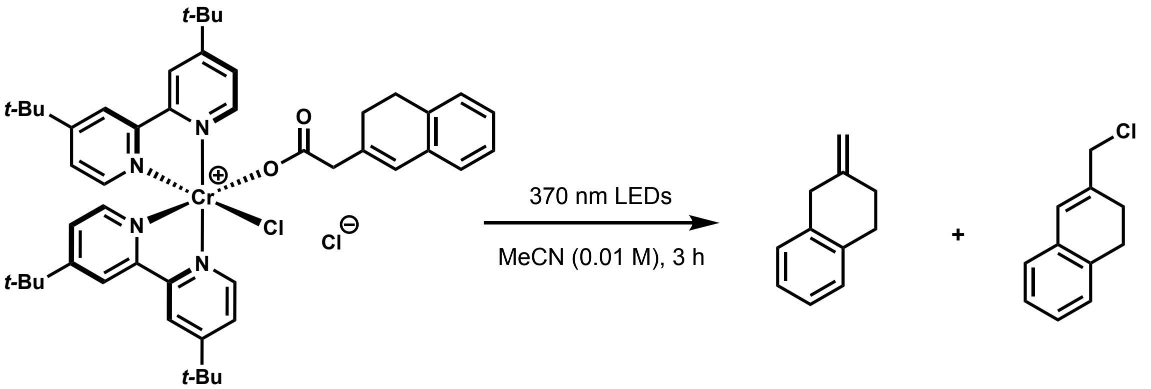

Cr-7 **4** detected by GC-MS **SI-16** not detected

Additionally, monitoring the photolysis of **Cr-7** by GC-MS led to the observation of **4**, while the chlorinated product **SI-16** was not detected. Together with the observation that substrates with labile benzylic C–H bonds (e.g., **3aa** and **3ae**) were well-tolerated under the reaction conditions, these experiments suggest that the generation of chlorine radicals via Cr–Cl LMCT is not a likely mechanistic pathway in this reaction.

6.8. UV-Vis Spectroscopy

<u>UV-Vis Spectra of **1d**, **1d-K**, 4,4'-di-*tert*-butyl-2,2'-bipyridine (dtbbpy), **Cr-1–Cr-4, Cr-6,** and **Cr-7**:</u>

Inside a N_2 filled glovebox, to a quartz cuvette (Starna Cells, Inc., Cat. No.: 3-Q-10-GL14-S, 10 mm path) was added the corresponding compound (5.0 µmol). Then MeCN (3 mL) was added to generate a 1.7 mM solution. The cuvette was sealed with a septum screw cap and taken out of the glovebox. The solution was homogenized with sonication (*ca.* 30 s) and subjected to UV-Vis spectral analysis. The UV-Vis absorption spectra of the compounds are displayed in **Figure SI-7**.

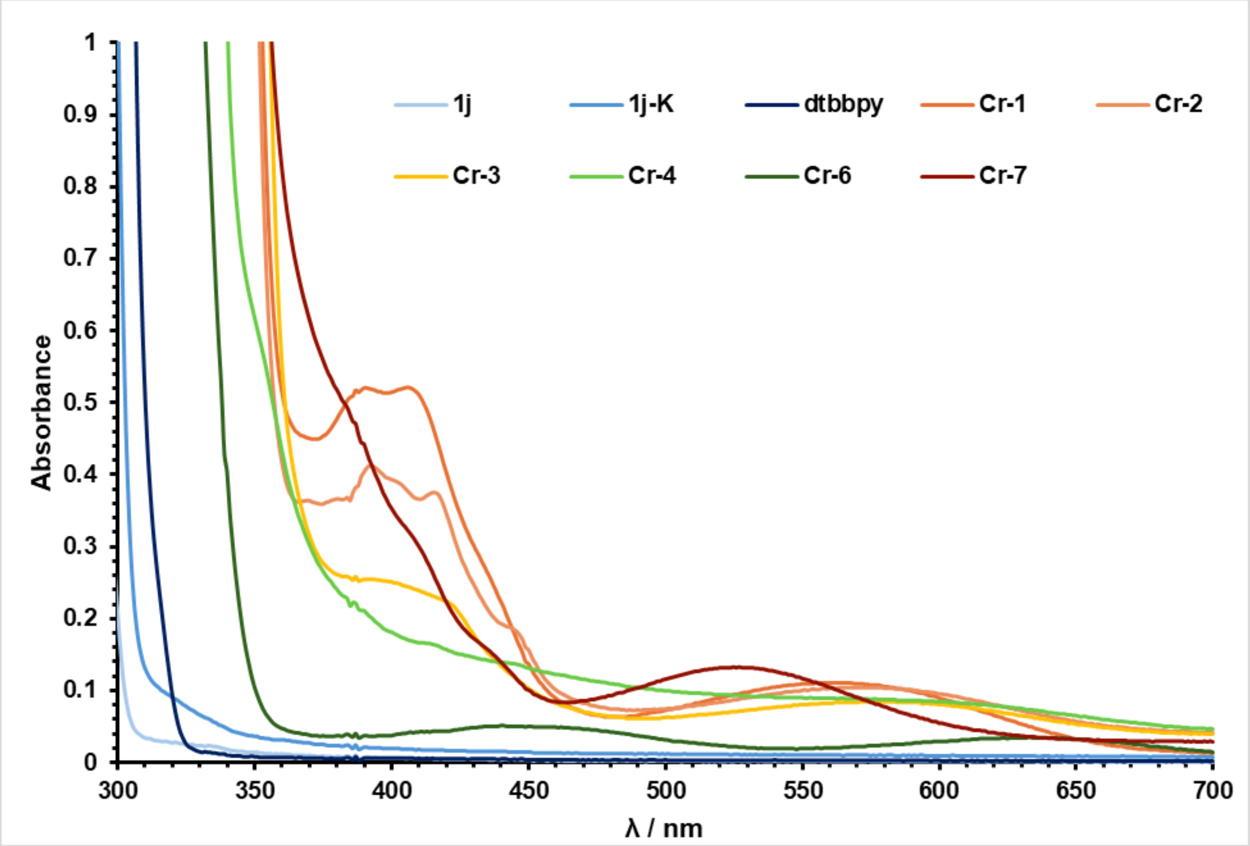

Figure SI-7: UV-Vis spectra of **1d**, **1d-K**, dtbbpy, **Cr-1–Cr-4, Cr-6, and Cr-7**. The UV-Vis spectrum for **Cr-5** has been previously reported.[27] All UV-Vis spectra were acquired at 1.7 mM concentration.

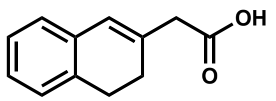

1j

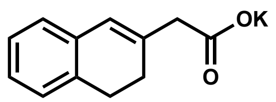

1j-K

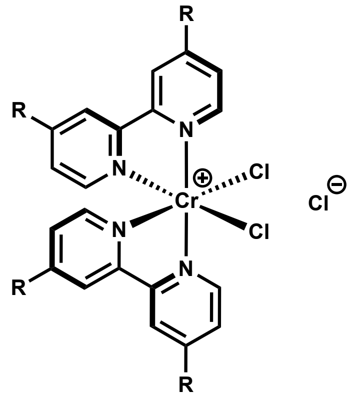

Cr-1: R = *t*-Bu
Cr-2: R = H

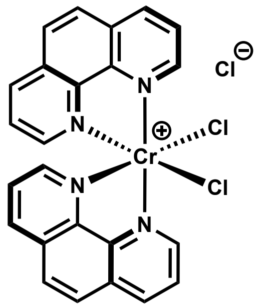

Cr-3

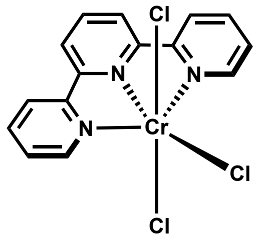

Cr-4

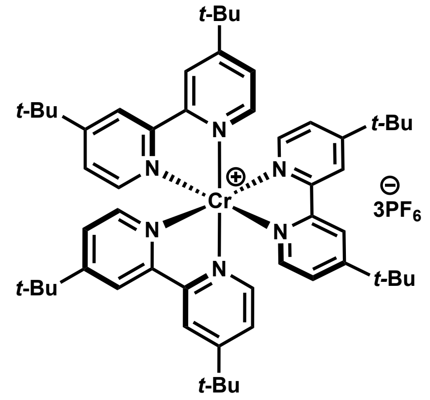

Cr-5

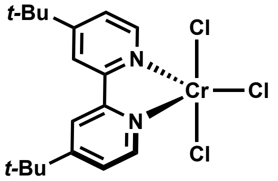

Cr-6

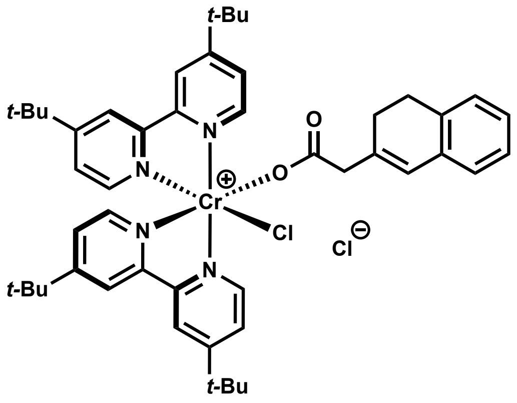

Cr-7

<u>Formation of Monocarboxylate Complex (**Cr-7**):</u>

Inside a N_2 filled glovebox, to an oven-dried reaction vial (VWR, catalog no. 66011-041) equipped with a magnetic stir bar was added **Cr-1** (14 mg, 0.02 mmol, 1.0 equiv), K_2CO_3 (6 mg, 0.04 mmol, 2.0 equiv), and **1d** (8 mg, 0.04 mmol, 2.0 equiv). Then, MeCN (2 mL, 0.01 M) was added, and the reaction mixture was allowed to stir at room

temperature for 3 h under N_2. Then the reaction mixture was filtered through a short pad of anhydrous Celite (*ca.* 20 mg), and 500 µL of the resulting solution was syringed into a cuvette and diluted with 2.5 mL of MeCN to make a 1.7 mM solution. The cuvette was sealed and taken out of the glovebox for UV-Vis spectral analysis. This procedure was then repeated for alternative amounts of **1d** and K_2CO_3 (0.5 equiv, 1 equiv, 5 equiv), with the ratio between **1d** and K_2CO_3 fixed at 1:1. The UV-Vis spectra are displayed in **Figure SI-8**.

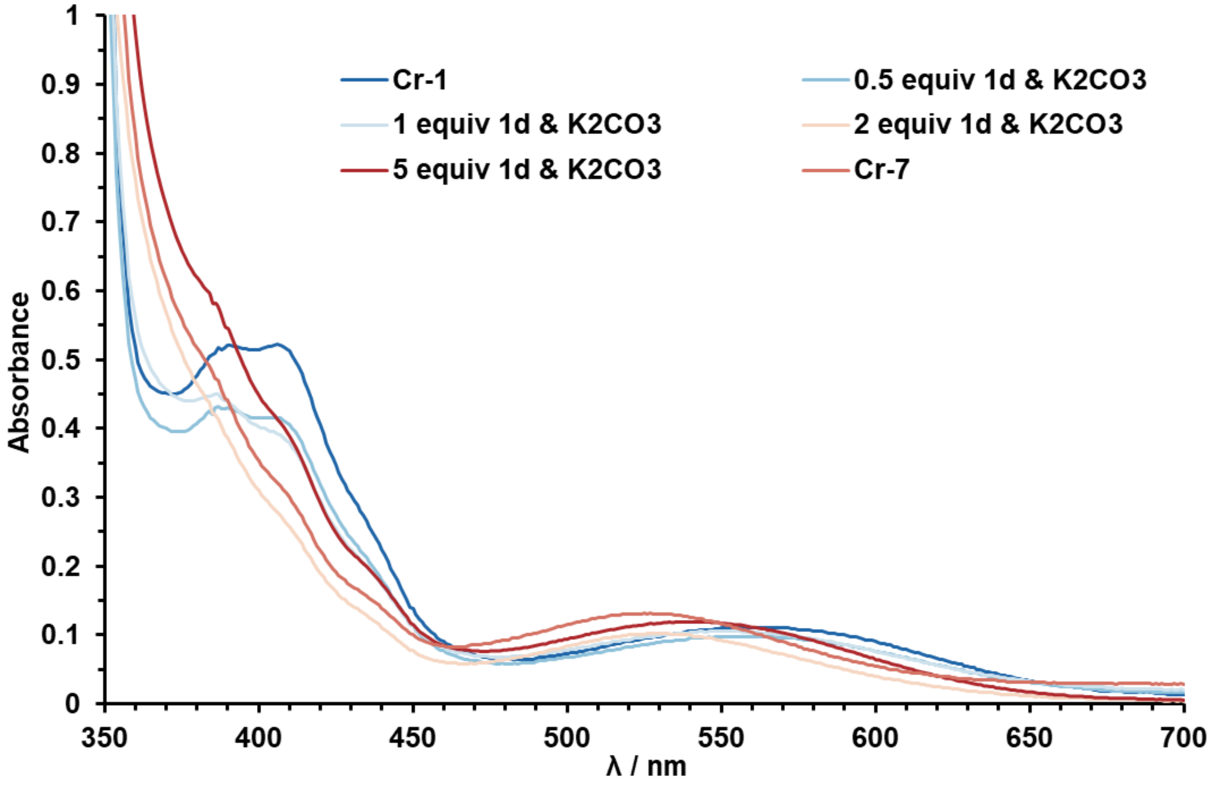

Figure SI-8: Formation of **Cr-7** from **Cr-1** with different equivalences of **1d** and K_2CO_3.

<u>UV-Vis Measurement of $Cr(dtbbpy)_2Cl_2$:</u>

Inside a N_2 filled glovebox, to an oven-dried 1-dram vial (VWR, catalog no. 66011-041) equipped with a magnetic stir bar was added $CrCl_2$ (3 mg, 0.02 mmol), 4,4'-di-*tert*-butyl-2,2'-bipyridine (11 mg, 0.04 mmol), and MeCN (2 mL, 0.01 M). The solution was allowed to stir for 3 h at room temperature under N_2. After this, the stirring was stopped, the solution was diluted to generate a 0.3 mM solution in MeCN, and 3 mL of the 0.3 mM solution was transferred to a cuvette. The cuvette was sealed and brought outside of the glovebox for UV-Vis spectral analysis. The UV-Vis spectrum is displayed in **Figure SI-9**.

<u>Photolysis of Cr-7:</u>

Inside a N_2 filled glovebox, to a cuvette was added **Cr-7** (0.7 mg, 0.8 µmol). Then, MeCN (3 mL) was added to generate a 0.3 mM solution. The cuvette was sealed, brought

outside of the glovebox, and subjected to 370 nm LED irradiation at 100% intensity. Irradiation was stopped at 10 s intervals (10 s, 20 s, 30 s, 40 s, 50 s, and 60 s after the onset of irradiation) for UV-Vis spectral analysis. The UV-Vis spectra are displayed in the **Figure SI-9** along with the UV-vis spectrum of a 0.3 mM solution of **Cr-7**.

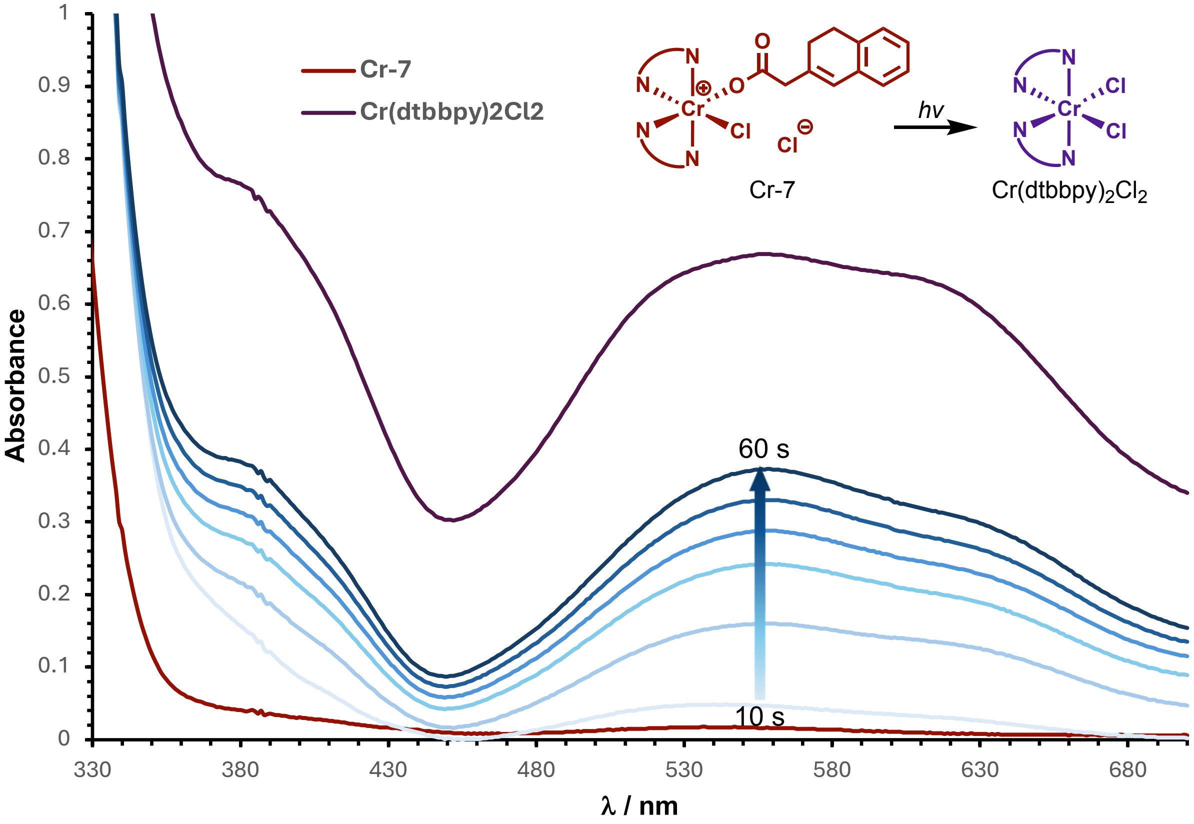

Figure SI-9: Photolysis of **Cr-7**.

Irradiation of **Cr-1**:

Inside a N_2 filled glovebox, to an oven-dried 1-dram vial (VWR, catalog no. 66011-041) equipped with a magnetic stir bar was added **Cr-1** (14 mg, 0.02 mmol) and MeCN (2 mL, 0.01 M). The vial was sealed with a PTFE-lined phenolic vial screw cap (Thermo Scientific, catalog no. 03-375-25A with 03-340-10G), removed from the glovebox, and subjected to 370 nm LED irradiation at 100% intensity with vigorous stirring for 3 h. Then, the vial was brought into the glovebox, and 500 μL of the solution was syringed into a cuvette and diluted to generate a 1.7 mM solution in 3 mL in MeCN. The cuvette was sealed and taken out of the glovebox for UV-Vis spectral analysis. The UV-Vis spectrum is displayed in **Figure SI-10**.

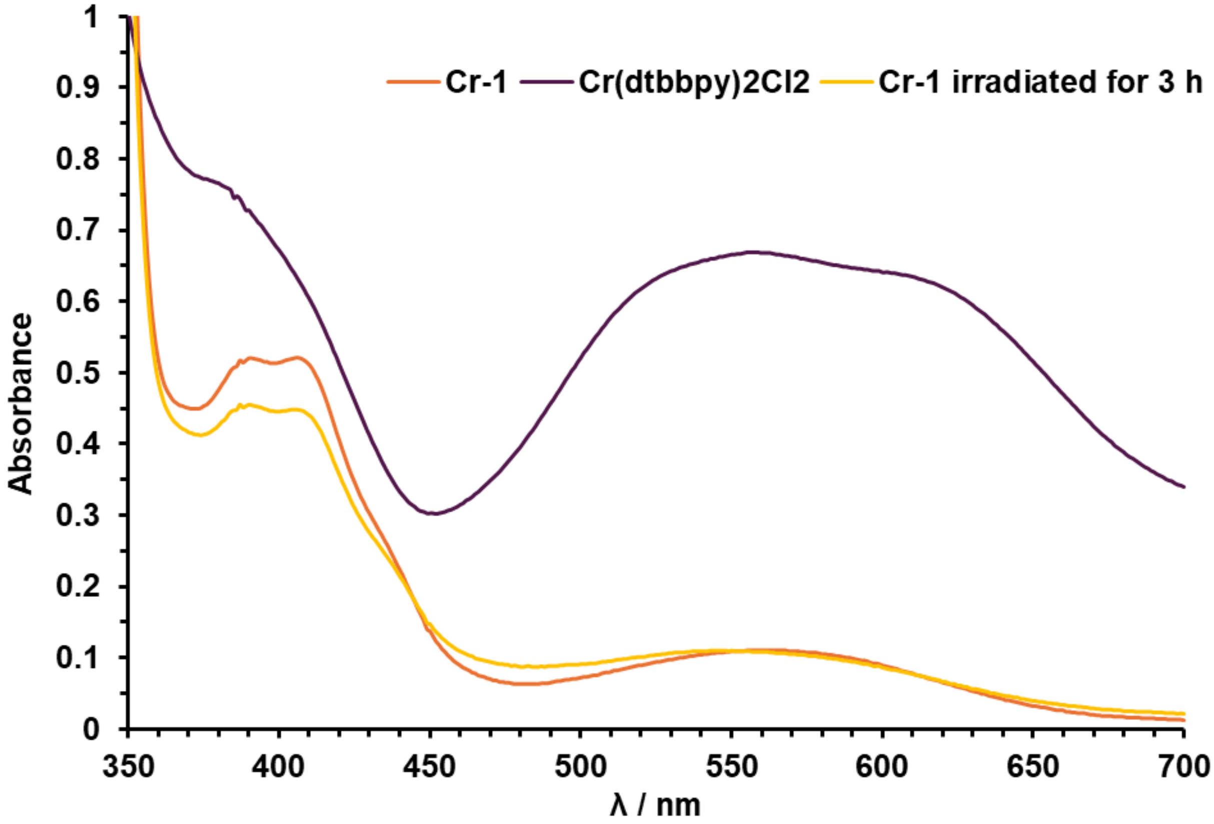

Figure SI-10: Irradiation of **Cr-1**.

Speciation of **Cr-5** in the Presence of dtbbpy:

Inside a N_2 filled glovebox, to an oven-dried 1-dram vial (VWR, catalog no. 66011-041) equipped with a magnetic stir bar was added **Cr-5** (9 mg, 0.02 mmol, 1 equiv), dtbbpy (11 mg, 0.04 mmol, 2 equiv), and MeCN (2 mL, 0.01 M). The reaction mixture was allowed to stir for 3 h under N_2. After this, stirring was stopped, and 500 μL of the solution was transferred to a cuvette and diluted with MeCN to generate a 1.7 mM solution. The cuvette was sealed with a septum screw cap and brought outside of the glovebox for UV-Vis spectral analysis. The UV-Vis spectrum is displayed in **Figure SI-11.**

Inside a N_2 filled glovebox, to an oven-dried scintillation vial was added **Cr-5** (9 mg, 0.02 mmol, 1 equiv), dtbbpy (11 mg, 0.04 mmol, 2 equiv), and MeCN (5 mL, 3.3 mM). 1 mL of this solution was transferred to a cuvette equipped with a magnetic stir bar and diluted with MeCN to generate a 1.7 mM solution. The cuvette was sealed with a septum screw cap, removed from the glovebox, and subjected to 370 nm LED irradiation at 100% intensity with vigorous stirring. Irradiation was stopped after 1 min and 1.5 h for UV-Vis spectral analysis. The UV-Vis spectra are displayed in **Figure SI-11**.

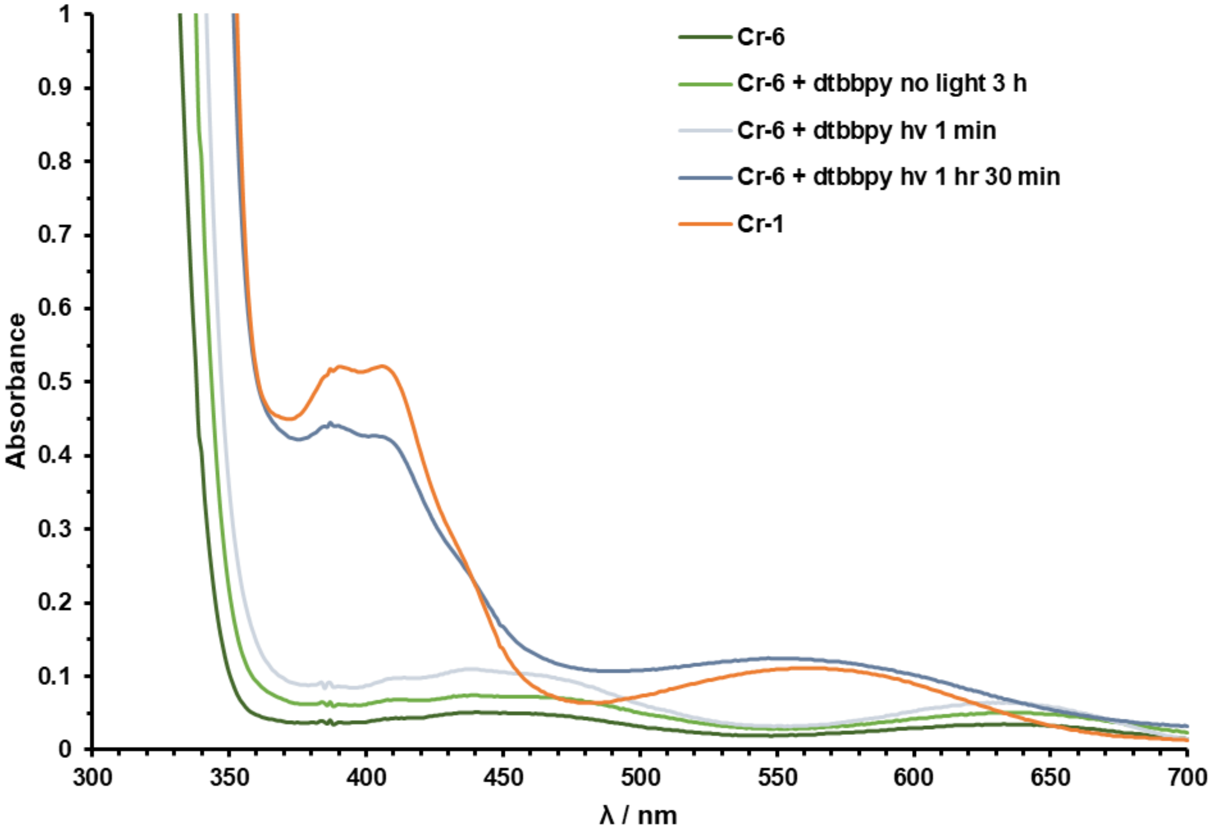

Figure SI-11: Speciation of **Cr-6** in the presence of dtbbpy.

Investigating LMCT efficiency of **Cr-1**–**Cr-3** through *in situ* monocarboxylate complex formation:

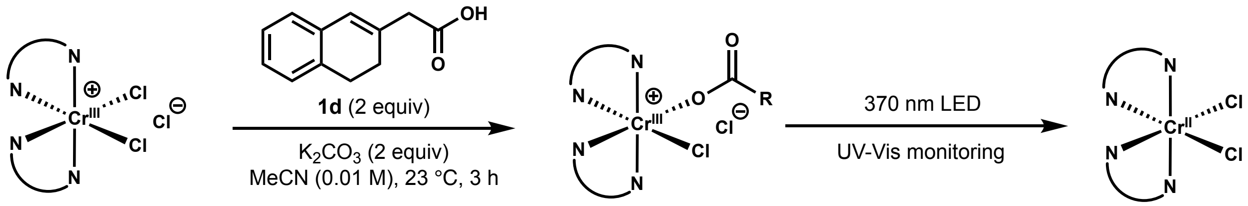

Cr-1–Cr-3

The monocarboxylate complexes for **Cr-1**–**Cr-3** were prepared analogously to **Cr-7** with 2 equiv of **1d** and K_2CO_3 in MeCN (0.01 M). Upon reaction completion, the reaction mixture was filtered through a short pad of anhydrous Celite (*ca.* 20 mg), and 500 µL of the resulting solution was syringed into a cuvette and diluted with 2.5 mL of MeCN to generate a 1.7 mM solution. The cuvette was sealed, removed from the glovebox, and an UV-Vis spectrum was acquired. Next, the solution was subjected to 370 nm LED irradiation at 100% intensity, with irradiation in 20 s intervals (i.e., 20 s, 40 s, and 60 s) for UV-Vis spectral analysis. The UV-Vis spectra are displayed in **Figure SI-12–14**. The UV-Vis spectra for the corresponding Cr(II) complexes were obtained analogously to $Cr(dtbbpy)_2Cl_2$.

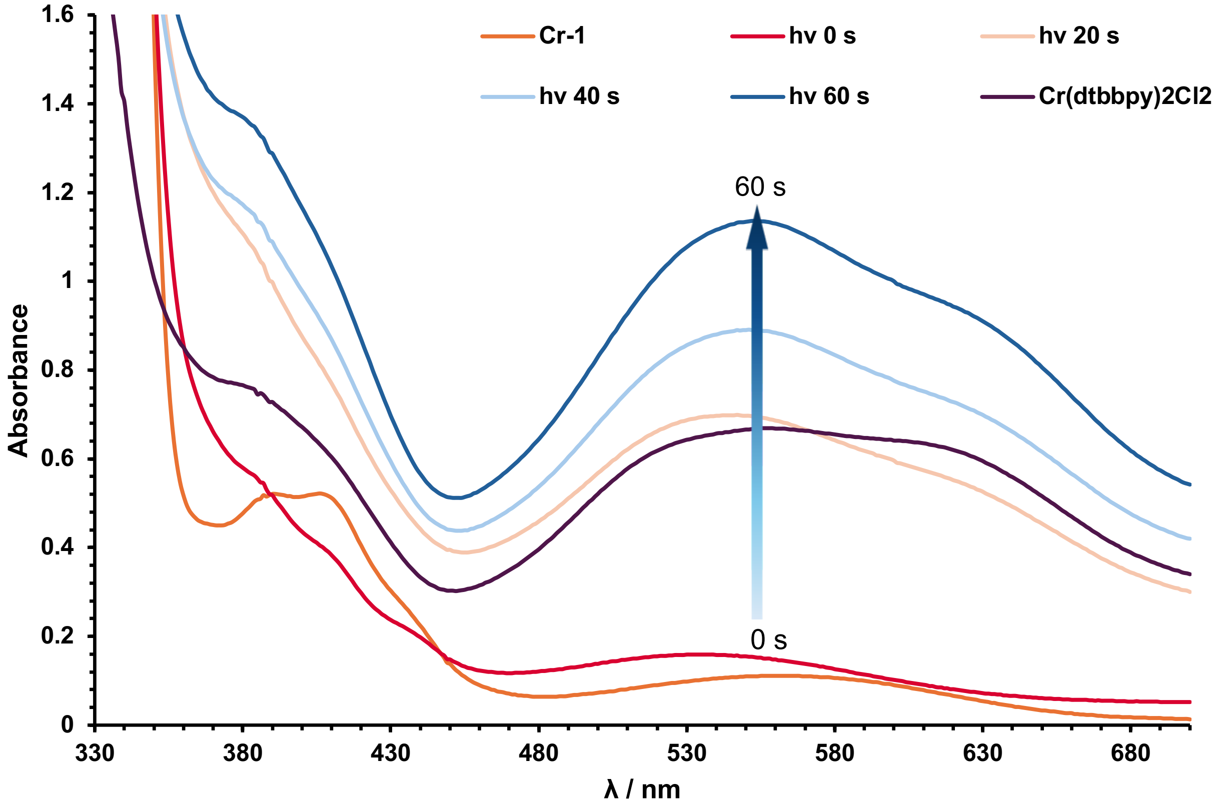

Figure SI-12: Photolysis of **Cr-7**. **Cr-7** was prepared *in situ* from **Cr-1** and diluted to *ca.* 1.7 mM for photolysis experiment.

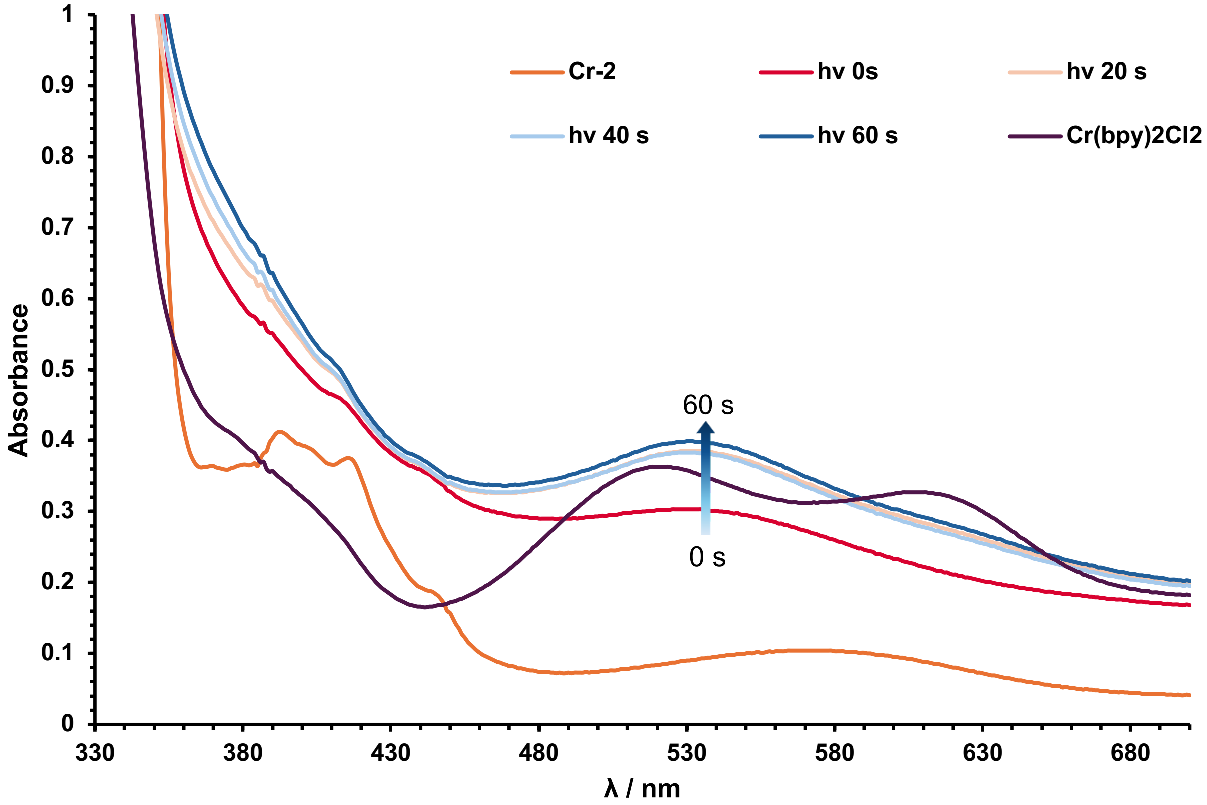

Figure SI-13: Photolysis of monocarboxylate complex of **Cr-2**. The monocarboxylate complex of **Cr-2** was prepared *in situ* and diluted to *ca.* 1.7 mM for photolysis experiment.

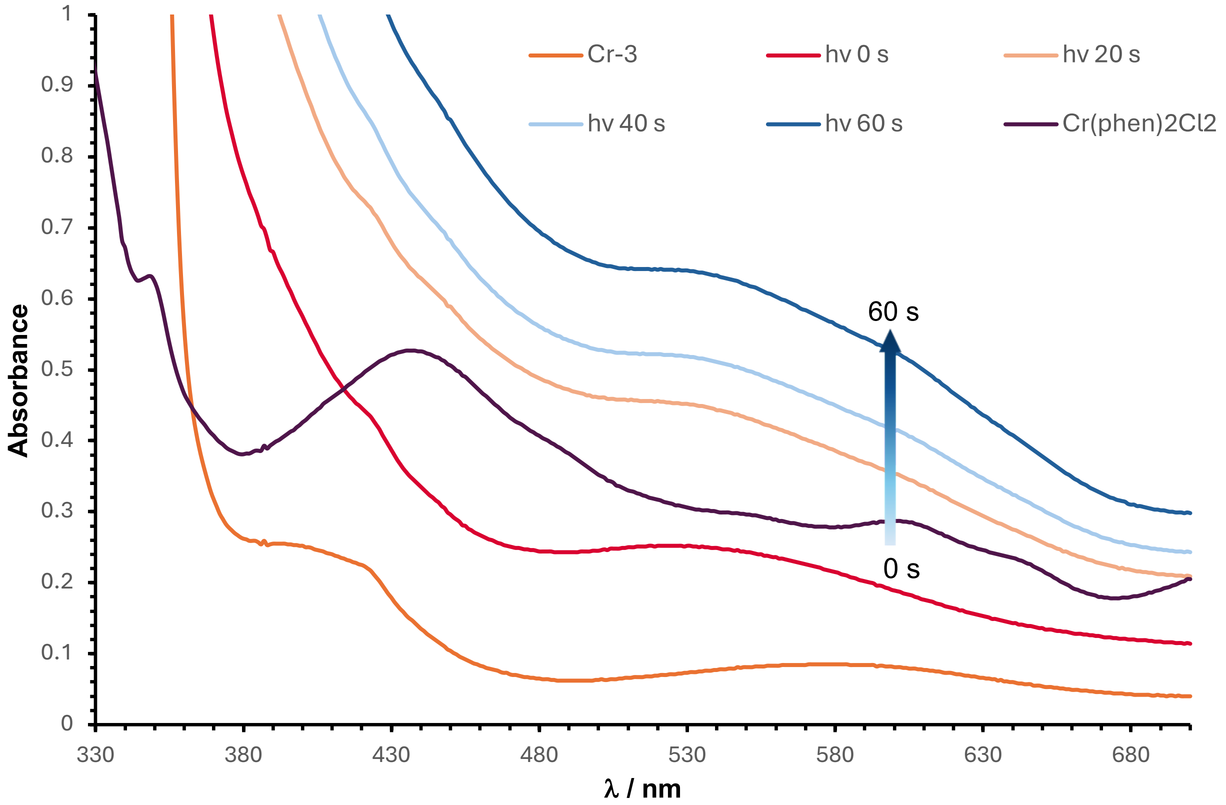

Figure SI-14: Photolysis of monocarboxylate complex of **Cr-3**. The monocarboxylate complex of **Cr-3** was prepared *in situ* and diluted to *ca.* 1.7 mM for photolysis experiment.

Although each monocarboxylate complex of **Cr-1**–**Cr-3** exhibited changes in absorption upon irradiation, only the UV-Vis trace for **Cr-7** (derived from **Cr-1**) showed convergence to the corresponding Cr(II) species. This result may suggest that the dtbbpy ligated Cr complex facilitates more efficient ligand-to-metal charge transfer compared to the analogous bpy and phen complexes.

Note: Due to the *in situ* formation protocol, the concentration of the resulting Cr-monocarboxylate complexes could not be precisely calibrated.

6.9 ^{19}F NMR Experiments

Preparation of triethylsilyl (*E*)-4-(4-fluorophenyl)but-3-enoate (**1g-Si**):

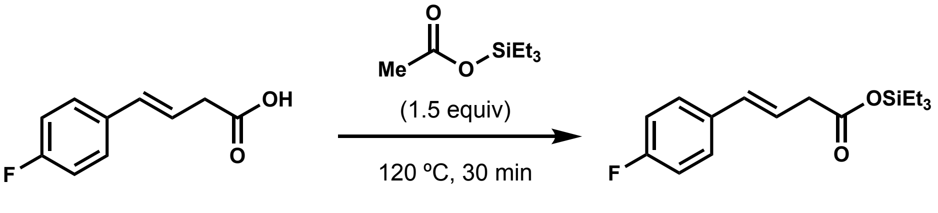

1g **1g-Si**

Prepared according to a modified literature procedure from Denmark and Edwards employing (*E*)-4-(4-fluorophenyl)but-3-noic acid instead of (*E*)-4-phenylbut-3-enoic acid.[28]

^{1}H NMR (600 MHz, MeCN-d$_3$): δ 7.44–7.39 (m, 2H), 7.06 (tt, *J* = 8.9, 2.3 Hz, 2H), 6.49 (d, *J* = 16.0 Hz, 1H), 6.27 (dt, *J* = 15.9, 7.2 Hz, 1H), 3.23 (dd, *J* = 7.1, 1.2 Hz, 2H), 0.97 (t, *J* = 8.1 Hz, 9H), 0.77 (q, *J* = 7.8 Hz, 6H).

^{13}C NMR (151 MHz, MeCN-d$_3$): δ 172.7, 163.7 (d, *J* = 245.1 Hz), 134.6 (d, *J* = 3.3 Hz), 132.4, 128.9 (d, *J* = 7.9 Hz), 123.9 (d, *J* = 2.2 Hz), 116.3 (d, *J* = 21.7 Hz), 40.2, 6.8, 5.2.

^{19}F NMR (470 MHz, MeCN-d$_3$): δ −117.1.

IR (Diamond-ATR, neat) ṽ (cm^{-1}): 2957, 2882, 1714, 1510, 1296, 1276, 1228, 1012, 973, 852.

HRMS (ESI): m/z: $[M+H]^+$ calc'd for $C_{16}H_{24}FO_2Si^+$: 295.1524. Found: 295.1512.

^{19}F Monitoring of **Cr-8** Formation:

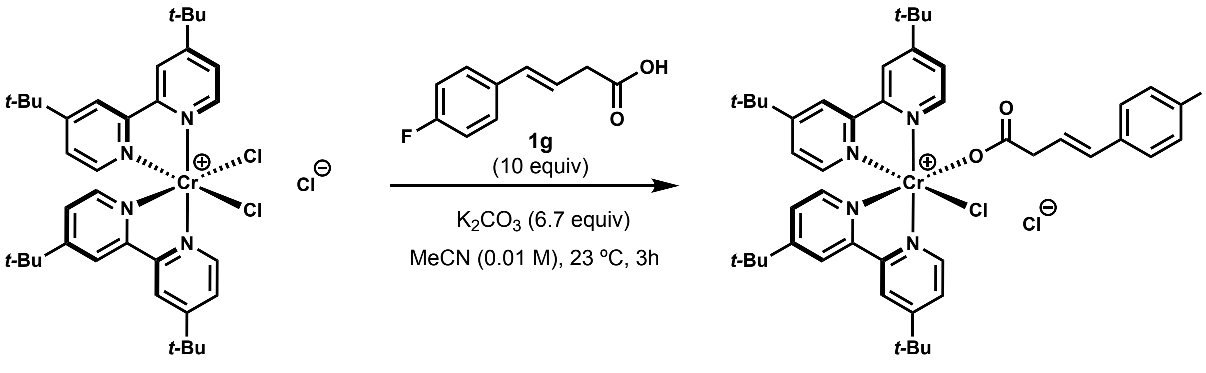

Cr-1 **Cr-8**

Inside a N_2 filled glovebox, to an oven-dried reaction vial (Thermo Scientific, catalog no. CHCV20-14) equipped with a magnetic stir bar was added **Cr-1** (42 mg, 0.060 mmol, 1.0 equiv), (*E*)-4-(4-fluorophenyl)but-3-enoic acid (**1g**) (108 mg, 0.60 mmol, 10.0 equiv), K_2CO_3 (55 mg, 0.40 mmol, 6.7 equiv), and MeCN (6 mL, 0.01 M). The reaction mixture was allowed to stir for 3 h at room temperature under N_2 atmosphere, and $PhCF_3$ (4 μL, 0.030 mmol, 0.5 equiv) was added as an internal standard. Next, 100 μL of the solution was transferred via syringe into an NMR tube, diluted with 300 μL of MeCN-d_3, sealed, removed from the glovebox, and subjected to ^{19}F NMR analysis to generate the ^{19}F spectrum for the Cr monocarboxylate complex (**Cr-8**) (**Figure SI-15**). Single crystals of **Cr-8** suitable for X-ray diffraction were grown by transferring *ca*. 50 μL of a solution of **Cr-8** in MeCN (0.04 M) into a recrystallization tube (Fisherbrand™ Disposable Flint Glass Tubes with Plain End, Catalog No.14-958A). Vapor diffusion of Et_2O into this solution at room temperature gave light red crystals of **Cr-8** (with one molecule of Et_2O per unit cell) after approximately 48 h.

^{19}F NMR (470 MHz, MeCN-d_3): δ −117.7.

HRMS (ESI): m/z: $[M-Cl]^+$ calc'd for $C_{46}H_{56}ClCrFN_4O_2^+$: 802.3475. Found: 802.3456.

The reaction vessel containing the remaining reaction mixture was sealed, removed from the glovebox, and irradiated at 370 nm for 1 h. After that, the reaction mixture was brought back into the glovebox, and 100 μL of the solution was transferred via syringe into an NMR tube, diluted with 300 μL of MeCN-d_3, sealed, removed from the glovebox, and subjected to ^{19}F NMR analysis. The ^{19}F signal for **Cr-8** disappeared upon 1 h of 370 nm LED irradiation, suggesting its photolysis upon ligand-to-metal charge transfer excitation (**Figure SI-15**).

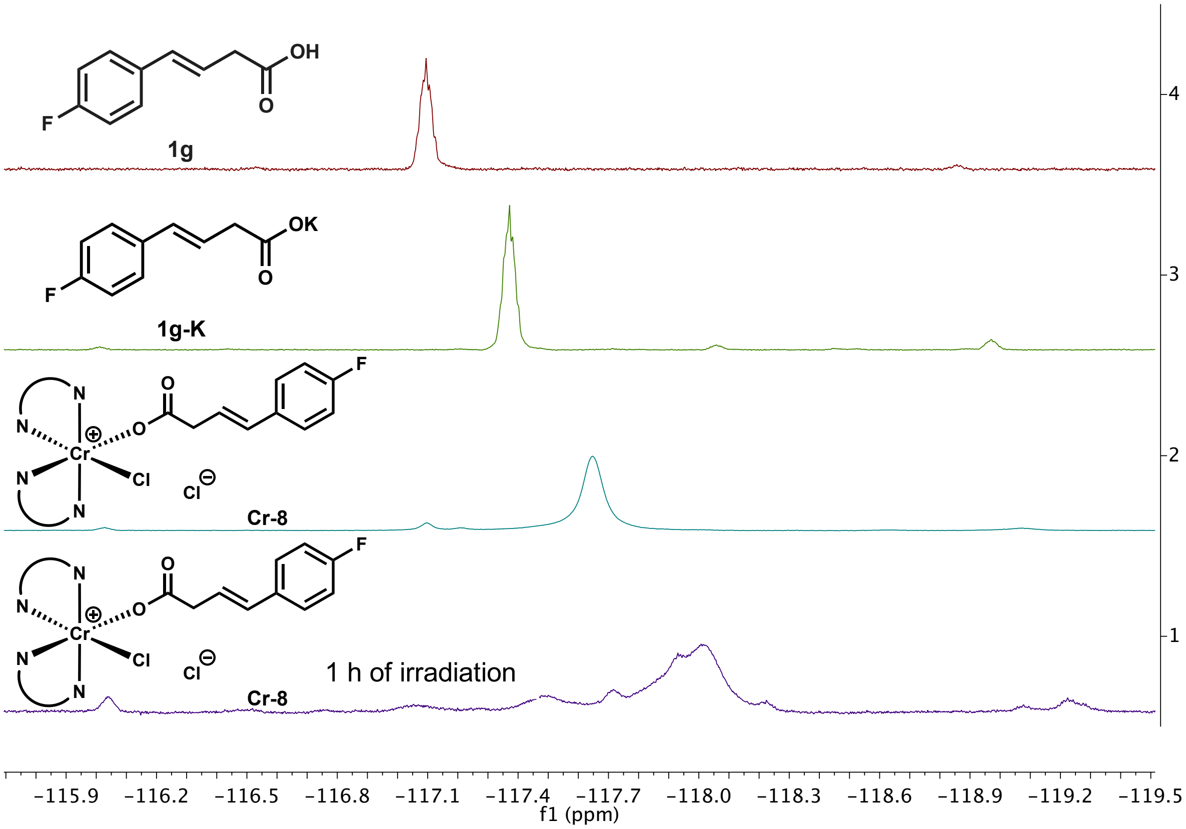

Figure SI-15: Formation of **Cr-8** from **Cr-1** and **1g.**

<u>[19]F Monitoring of Homoallylic Alcohol Product Formation:</u>

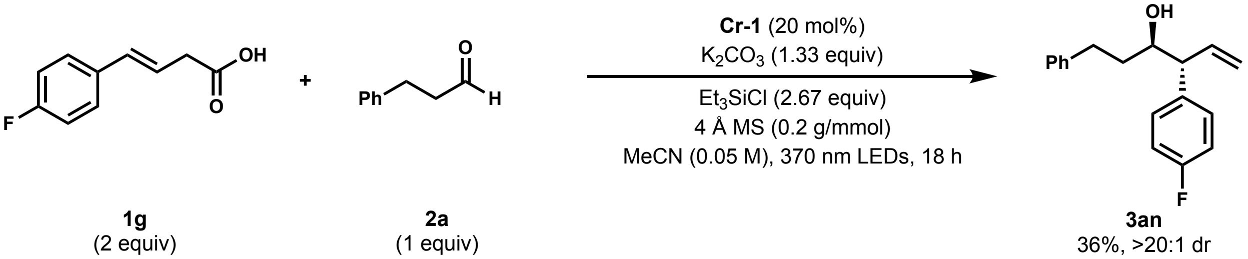

Prepared according to a modified **General Procedure H** from 3-phenyl-propanal (**2a**) (13 μL, 0.10 mmol) and (*E*)-4-(4-fluorophenyl)but-3-enoic acid (**1g**) (36 mg, 0.20 mmol) with a reaction time of 18 h. A NMR yield was determined by ^{1}H NMR spectroscopy of the crude reaction mixture using 1,1,2,2-tetrachloroethane (TCE) as the internal standard (36% ^{1}H NMR yield). An analytically pure sample of **3an** was purified by preparatory thin-layer chromatography (CH_2Cl_2) and isolated as a colorless oil and >20:1 mixture of diastereomers.

Product formation was analyzed with ^{19}F NMR spectroscopy via a modified **General Procedure H**. After 24 h of irradiation, the reaction mixture was brought into the glovebox.

100 μL of the solution was transferred via syringe into a NMR tube, diluted with 300 μL of MeCN-d_3, sealed, removed from the glovebox, and subjected to ^{19}F NMR analysis. This procedure was repeated for alternative irradiation times: 0 h, 1 h, 3 h, and 6 h. ^{19}F NMR shows the formation of the addition product over time (**Figure SI-16**).

^{1}H NMR (600 MHz, $CDCl_3$): δ 7.24 (d, *J* = 7.6 Hz, 2H), 7.16 (tt, *J* = 7.4, 1.3 Hz, 1H), 7.13–7.10 (m, 4H), 6.99 (t, *J* = 8.7 Hz, 2H), 6.06 (ddd, *J* = 17.1, 10.1, 9.1 Hz, 1H), 5.23 (dd, *J* = 10.3, 1.3 Hz, 1H), 5.20 (d, *J* = 17.2 Hz, 1H), 3.76 (tt, *J* = 7.9, 3.0 Hz, 1H), 3.26 (t, *J* = 8.2 Hz, 1H), 2.82 (ddd, *J* = 13.9, 9.6, 5.4 Hz, 1H), 2.63 (ddd, *J* = 13.8, 9.4, 7.2 Hz, 1H), 1.81 (d, *J* = 2.7 Hz, 1H), 1.70–1.60 (m, 2H).

^{13}C NMR (151 MHz, $CDCl_3$): δ 161.8 (d, *J* = 245.0 Hz), 142.1, 138.2, 137.2 (d, *J* = 3.4 Hz), 129.5 (d, *J* = 7.7 Hz), 128.6, 128.5, 126.0, 118.3, 115.7 (d, *J* = 21.2 Hz), 73.3, 56.8, 36.2, 32.2.

^{19}F NMR (470 MHz, MeCN-d_3): δ −119.1.

IR (Diamond-ATR, neat) ṽ (cm^{-1}): 3433, 2923, 2857, 1742, 1600, 1508, 1458, 1378, 1226, 1160, 1097, 922, 834.

HRMS (ESI): m/z: $[M-OH]^+$ calc'd for $C_{18}H_{18}F^+$: 253.1387. Found: 253.1368.

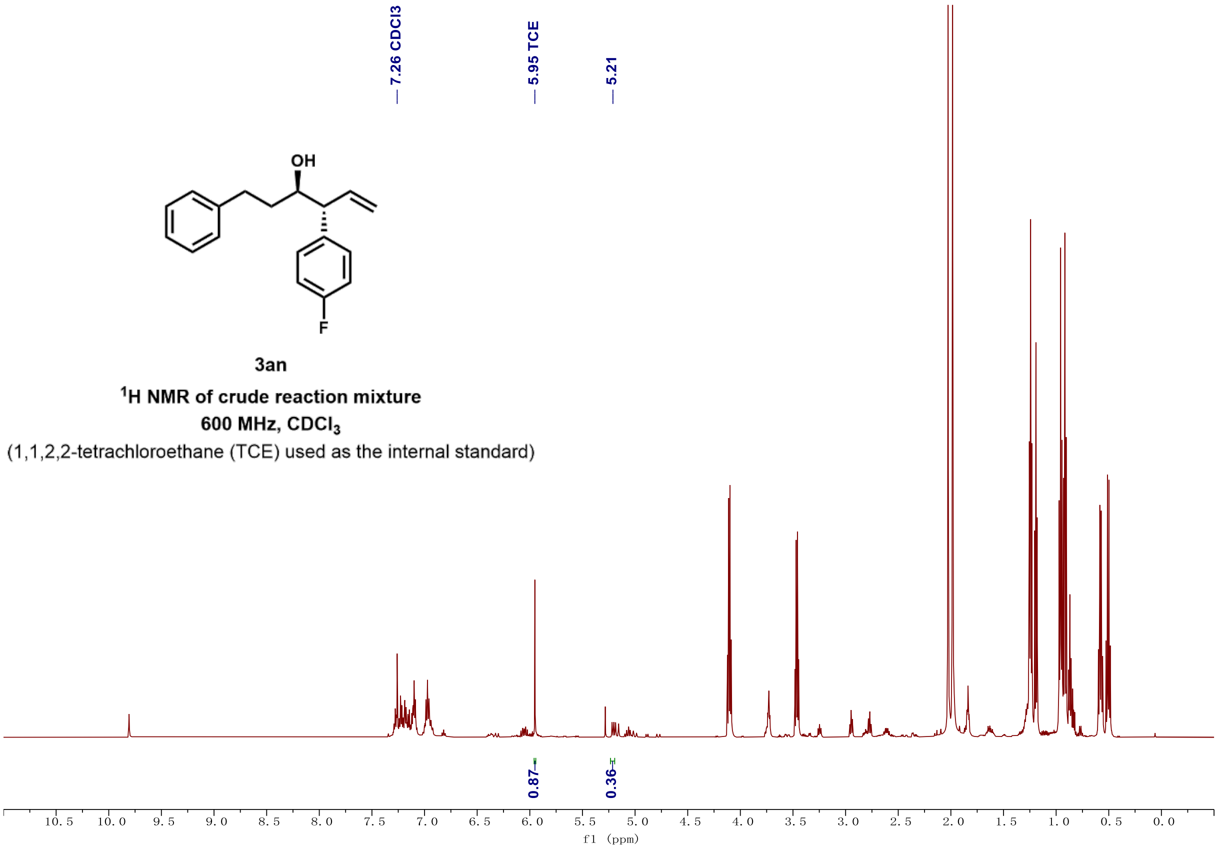

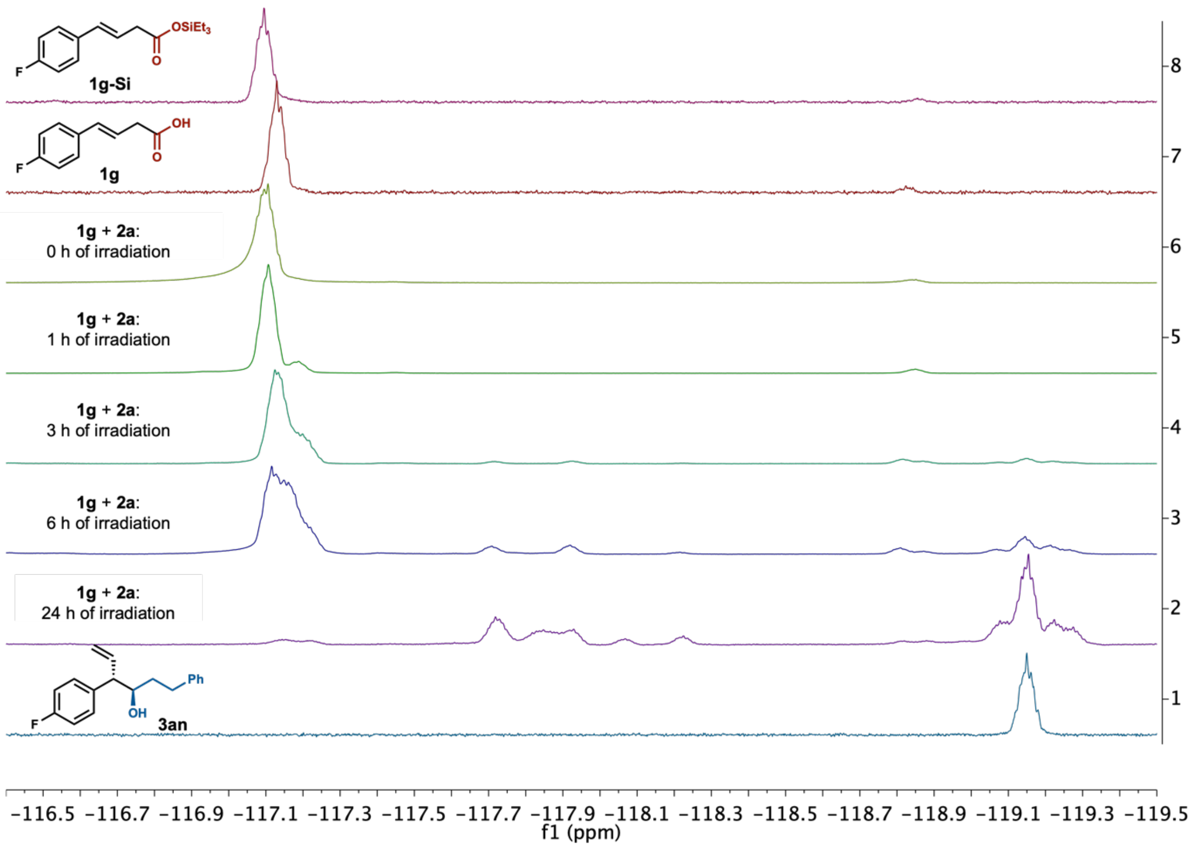

Figure SI-16: ^{19}F monitoring of the formation of allylation product **3an** from **1g** and **2a**.

Decarboxylative NHK experiments with **1g-Si**:

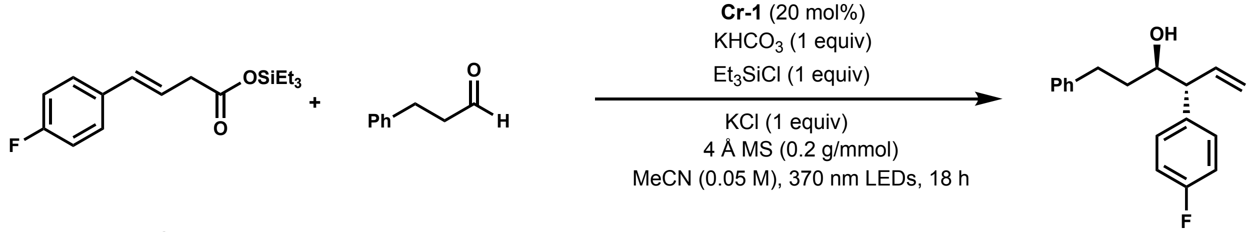

1g-Si (1 equiv) **2a** (1 equiv) **3an** 31%, >20:1 dr

Inside a N_2-filled glovebox, to an oven-dried reaction vial (VWR, catalog no. 66011-041) equipped with a magnetic stir bar (Chemglass Life Sciences, catalog no. CG-2003-160, 10 x 3 mm) was charged **Cr-1** (14 mg, 0.02 mmol, 20 mol%), KHCO3 (10 mg, 0.10 mmol, 1.0 equiv), KCl (7 mg, 0.10 mmol, 1.0 equiv), 4 Å molecular sieves (20 mg, 0.2 g/mmol), and MeCN (2.0 mL, 0.05 M). Then **1g-Si** (29 mg, 0.10 mmol, 1.0 equiv), **2a** (13 µL, 0.10 mmol, 1.0 equiv), and Et_3SiCl (17 µL, 0.10 mmol, 1.0 equiv) were added via syringe under vigorous stirring. The reaction vessel was sealed with a PTFE-lined phenolic vial screw cap (Thermo Scientific, catalog no. 03-375-25A with 03-340-10G), removed from the glovebox, and placed in a custom-made photoreactor 3 cm away from two 44 W Kessil PR-160L 370 nm LEDs and one 75 mm fan. The reaction mixture was subjected to LED irradiation at 100% intensity with vigorous stirring (*ca.* 50 °C). After 18 h, the reaction mixture was allowed to cool to room temperature, then filtered through a short silica plug (*ca.* 1 g) eluting with EtOAc (3 x 2 mL) and concentrated *in vacuo* with the aid of a rotary evaporator. Yield and diastereomeric ratio were evaluated by ^1H NMR of the crude reaction mixture using 1,1,2,2-tetrachloroethane (TCE) (*ca.* 6 µL) as the internal standard (31% ^1H NMR yield, >20:1 dr).

All spectroscopic data for **3an** was consistent with that which was reported above.

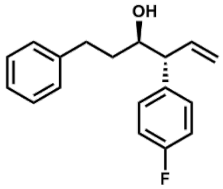

3an

^{1}H NMR of crude reaction mixture

600 MHz, $CDCl_3$

(1,1,2,2-tetrachloroethane (TCE) used as the internal standard)

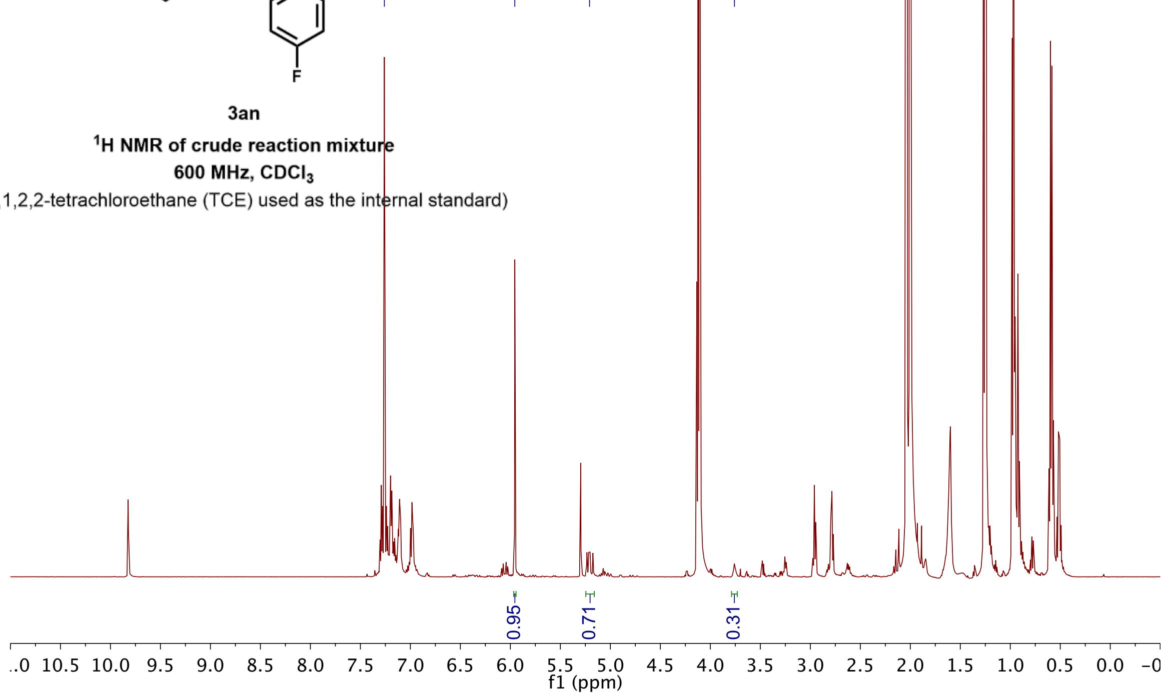

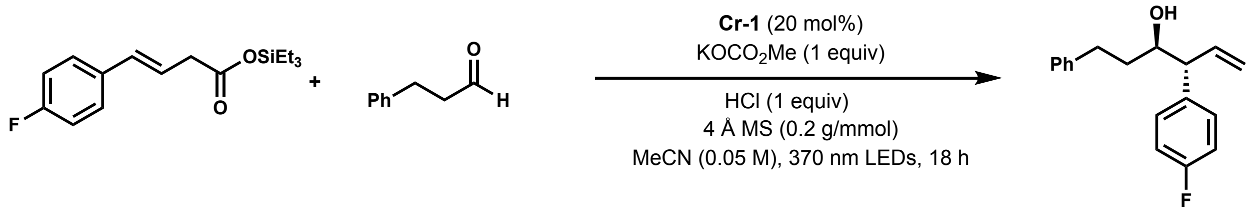

1g-Si (1 equiv) **2a** (1 equiv) **3an** 11%, >20:1 dr

In order to understand if the excess chlorosilane was interacting with the generated $KHCO_3$ to form triethylsilylcarbonic acid, we protonated potassium methyl carbonate ($KOCO_2Me$) *in situ* with HCl to act as a surrogate.

Inside a N_2-filled glovebox, to an oven-dried reaction vial (VWR, catalog no. 66011-041) equipped with a magnetic stir bar (Chemglass Life Sciences, catalog no. CG-2003-160, 10 x 3 mm) was charged $KOCO_2Me$ (11 mg, 0.10 mmol, 1.0 equiv), 4 Å molecular sieves (20 mg, 0.2 g/mmol), and MeCN (2.0 mL, 0.05 M). Then HCl (25 μL, 0.10 mmol, 1.0 equiv, 4.0 M in dioxane) was added via syringe, and the resulting mixture was allowed to stir at room temperature for 5 min. To the stirred reaction mixture was added **Cr-1** (14 mg, 0.02 mmol, 20 mol%), **1g-Si** (29 mg, 0.10 mmol, 1.0 equiv), and **2a** (13 μL, 0.10 mmol, 1.0 equiv). The reaction vessel was sealed with a PTFE-lined phenolic vial screw cap (Thermo Scientific, catalog no. 03-375-25A with 03-340-10G), removed from the glovebox, and placed in a custom-made photoreactor 3 cm away from two 44 W Kessil PR-160L 370 nm LEDs and one 75 mm fan. The reaction mixture was subjected to LED irradiation at 100% intensity with vigorous stirring (*ca.* 50 °C). After 18 h, the reaction mixture was allowed to cool to room temperature, then filtered through a short silica plug (*ca.* 1 g) eluting with EtOAc (3 x 2 mL) and concentrated *in vacuo* with the aid of a rotary evaporator. Yield and diastereomeric ratio were evaluated by ^1H NMR of the crude reaction mixture using 1,1,2,2-tetrachloroethane (TCE) (*ca.* 6 μL) as the internal standard (11% ^1H NMR yield, >20:1 dr).

All spectroscopic data for **3an** was consistent with that which was reported above.

The formation of **3an** catalyzed by a triethylsilylcarbonic acid analog supports the hypothesis that protonation of a carbonate species by Et_3SiCl is a plausible pathway in the reaction.

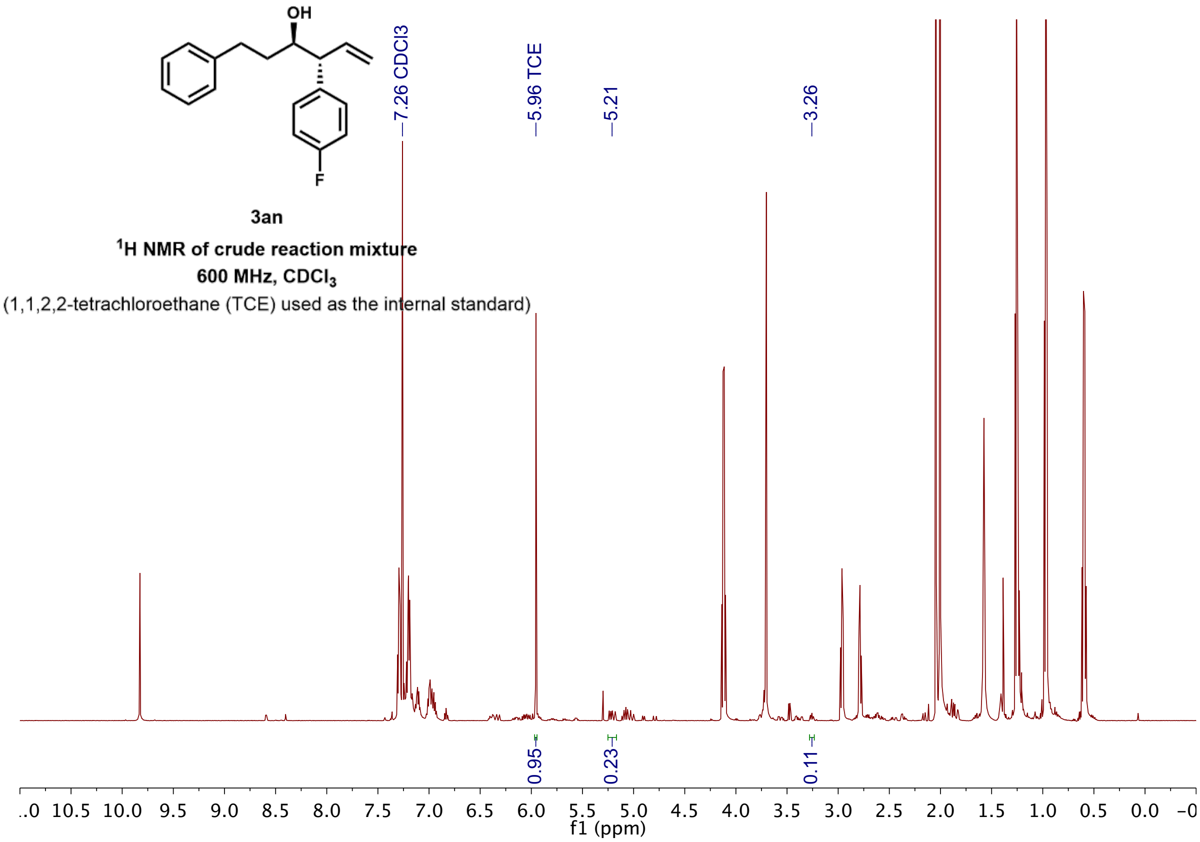
OH
F
3an
¹H NMR of crude reaction mixture
600 MHz, CDCl3
(1,1,2,2-tetrachloroethane (TCE) used as the internal standard)
7.26 CDCl3
5.96 TCE
5.21
3.26
0.95
0.23
0.11
f1 (ppm)

6.10. Cyclic Voltammetry (CV) Experiments

General Procedure for Acquiring Cyclic Voltammograms:

To a flame-dried 25 mL three-neck round-bottom flask equipped with a magnetic stir bar was added tetra-*n*-butylammonium hexafluorophosphate (194 mg, 0.50 mmol, 20.0 equiv). The flask was sealed with three rubber septa, evacuated, and backfilled with N_2 using a dual-manifold Schlenk line once. Then MeCN (5 mL, 0.005 M) and **Cr-1** (17 mg, 0.025 mmol, 1.0 equiv) were added. The resulting mixture was stirred vigorously for 5 min before the septa were removed. A glassy carbon working electrode, a platinum wire counter electrode, and an $Ag/AgNO_3$ reference electrode were attached through the three necks of the flask. The electrodes were connected to a Pine Research WaveDriver 40 DC Bipotentiostat through steel alligator clips, the stirring was stopped and the acquisition of the cyclic voltammogram was initiated. Cyclic voltammogram acquisition was performed at a rate of 75 mV/s, starting at 0 mV, with an initial rising segment to +2000 mV, followed by a falling segment to −2000 mV, and finally a rising segment to 0 mV. The resulting cyclic voltammograms were calibrated to ferrocene/ferrocenium by adding a small portion (*ca.* 2 mg) of ferrocene to the mixture, followed by stirring for 1 min before acquiring the cyclic voltammogram in the same manner. Cyclic voltammograms for potassium 2-(3,4-dihydronaphthalen-2-yl)acetate (**1d-K**) was also acquired in the same manner.

Results show that **Cr-1** is oxidized at $E_{p,c}$ = +0.75 V and **1d-K** is oxidized at $E_{p,c}$ = +1.20 V. The oxidation of **Cr-1** appears irreversible with no discernable corresponding reduction potential.

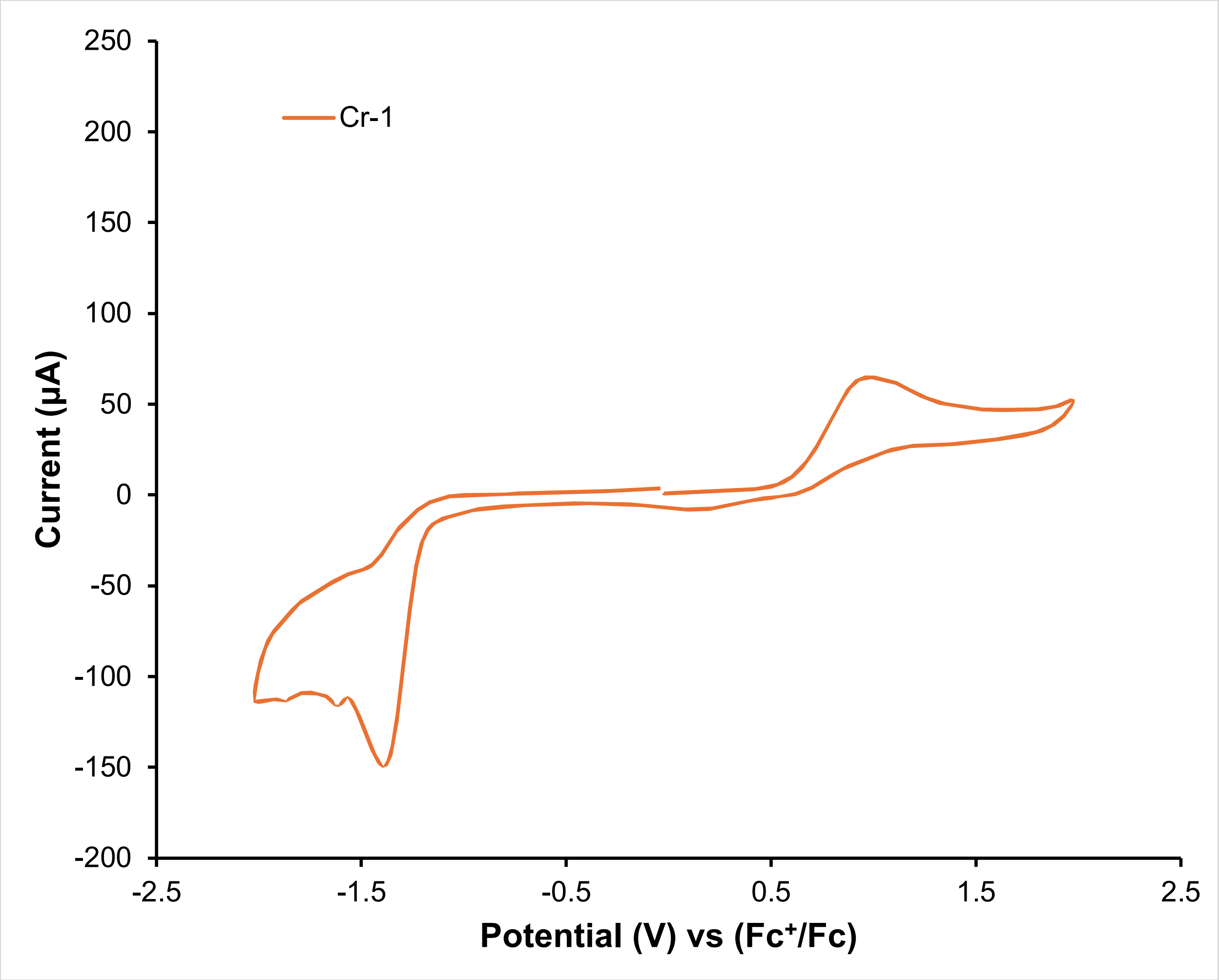

Figure SI-17: Cyclic voltammogram of **Cr-1**

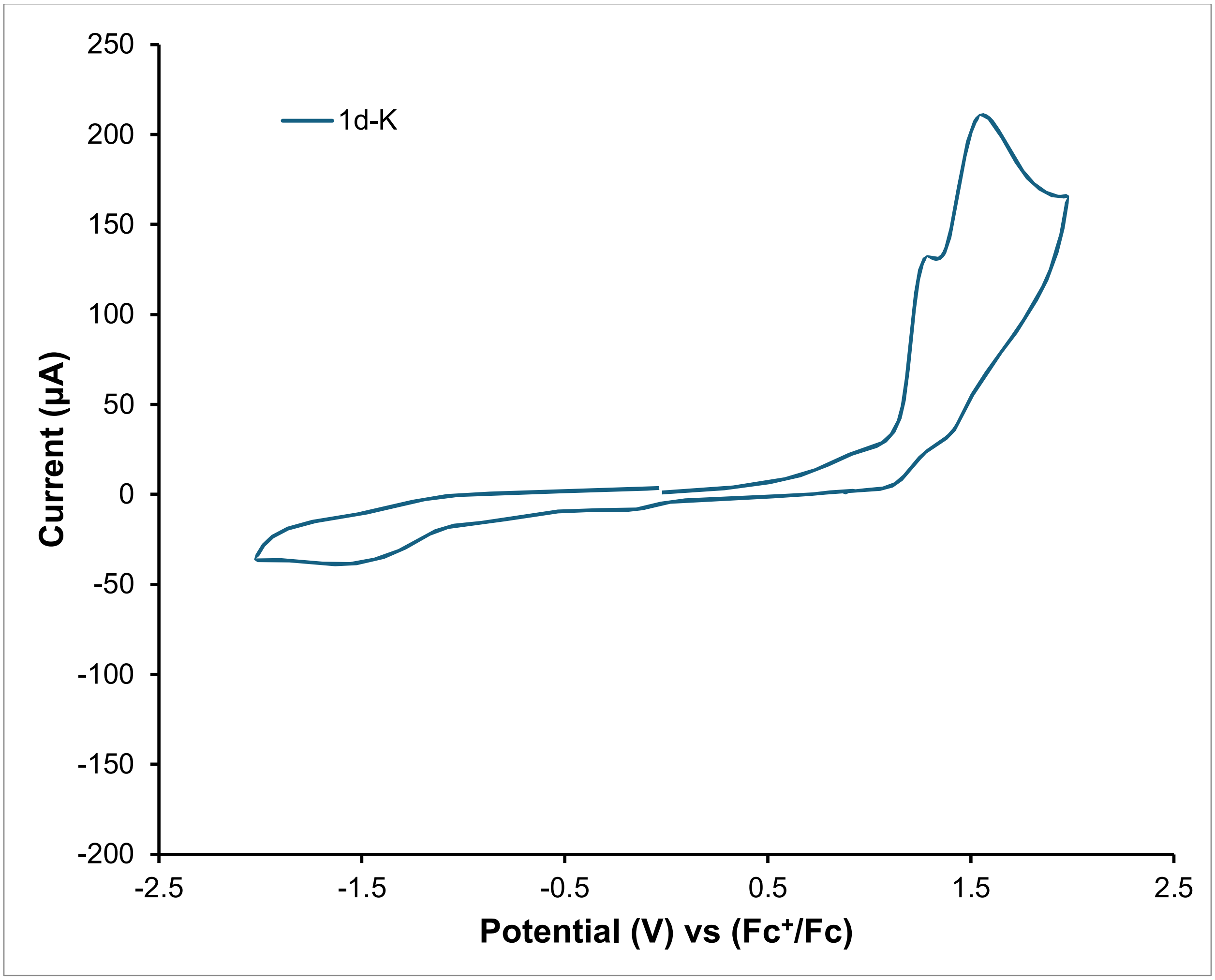

Figure SI-18: Cyclic voltammogram of **1d-K**

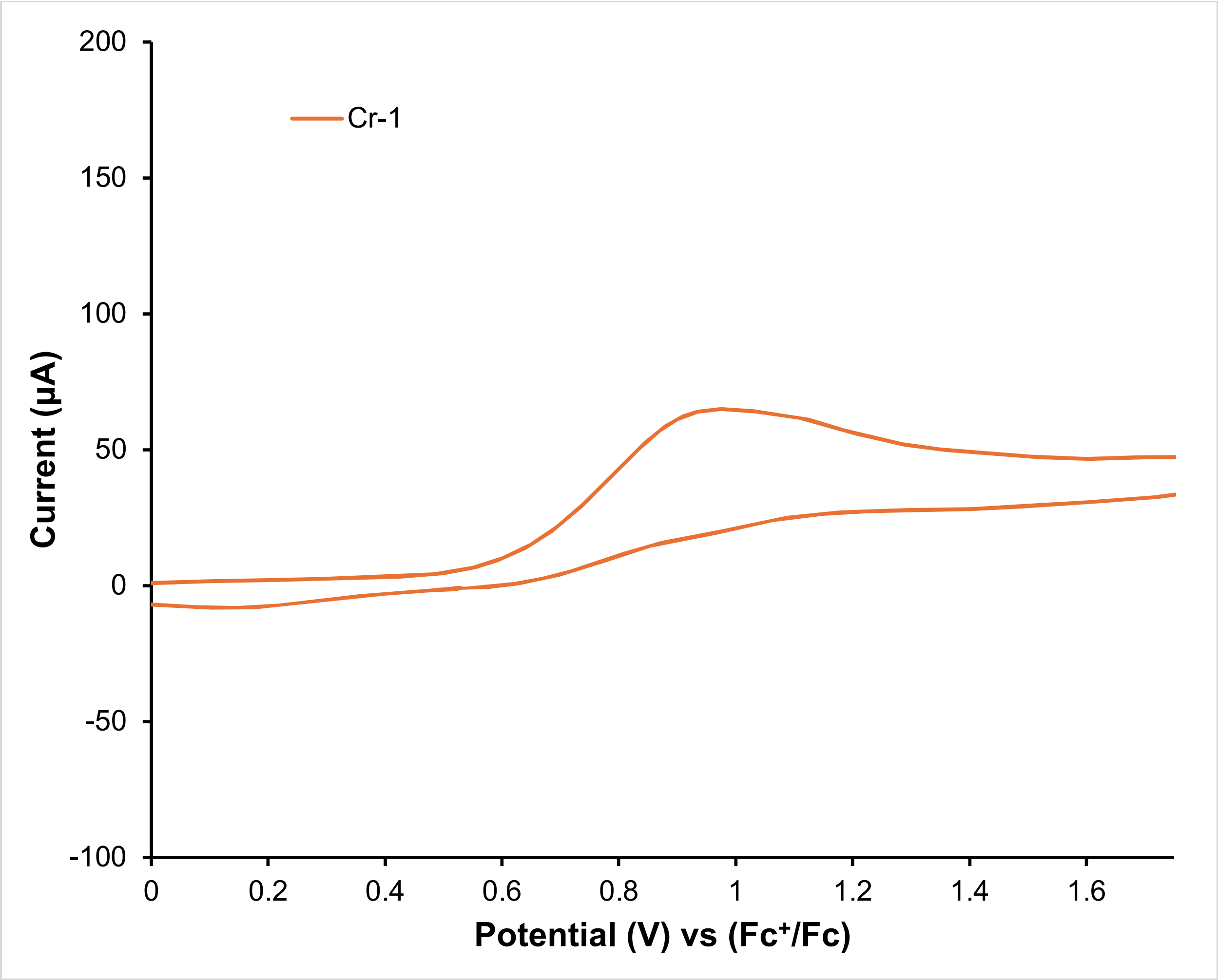

Figure SI-19: Expanded cyclic voltammogram region of **Cr-1** between 0.0 V and 1.75 V

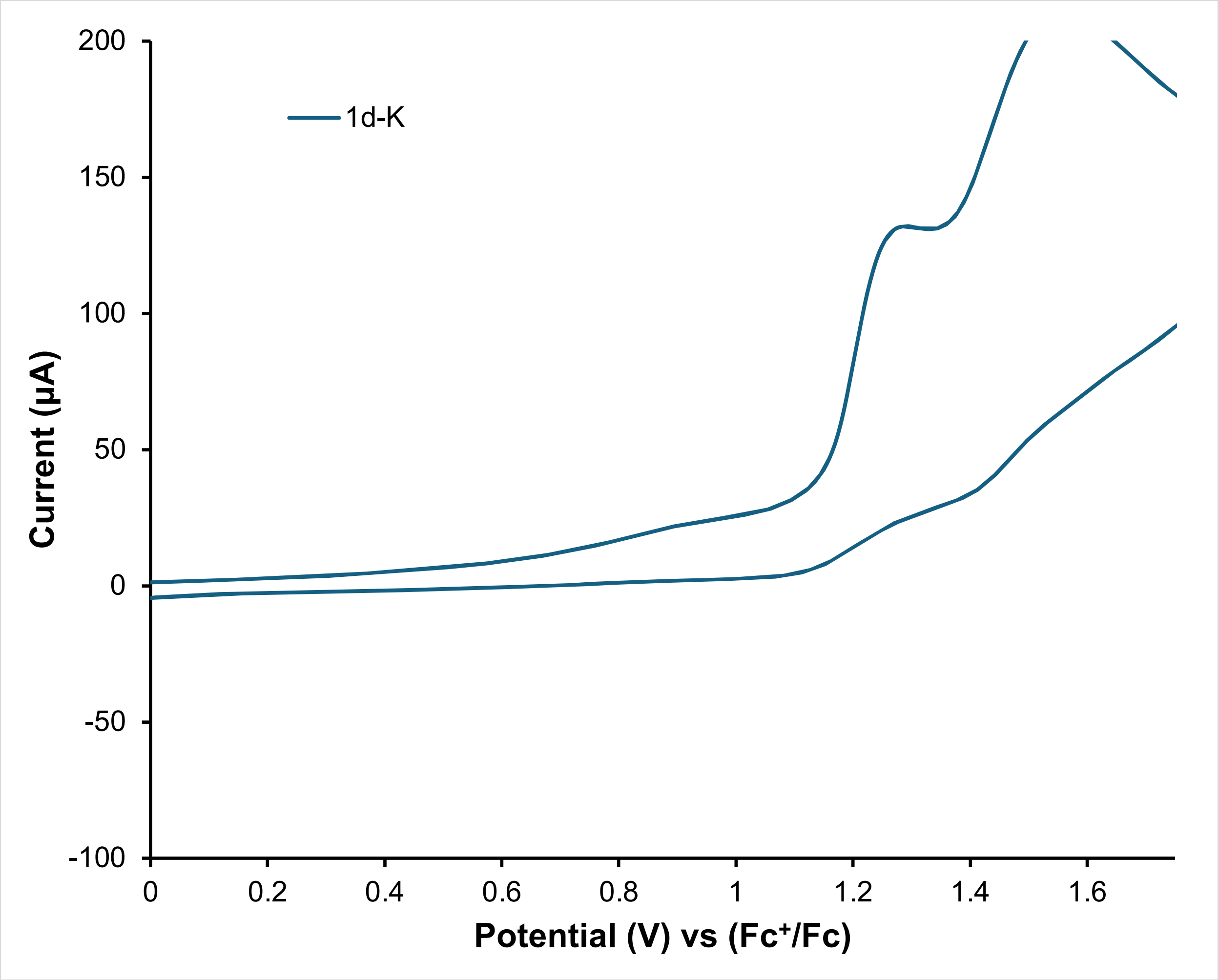

Figure SI-20: Expanded cyclic voltammogram region of **1d-K** between 0.0 V and 1.75 V

6.11. Fluorescence Measurement

Preparation of **Cr-1** stock solutions:

A sample of **Cr-1** (42 mg, 60 μmol) was dissolved in MeCN (6 mL), and the mixture was sonicated for 15 minutes to yield a homogeneous solution of **Cr-1** (0.01 M).

Measurement of fluorescence of **Cr-1**:

To a quartz cuvette was added 2.5 mL of **Cr-1** stock solution. Then, the cuvette was placed into a Varian Cary Eclipse Fluorescence Spectrophotometer. The fluorescence was measured utilizing fluorescence mode (Scan program provided by Varian Cary) with an excitation wavelength at 370 nm, 2.5 nm excitation slit width, and 2.5 nm emission slit width. Alternatively, the fluorescence was measured with an excitation wavelength at 350 nm. No fluorescence emission was detected in either experiment.

6.12. Deuteration Experiment

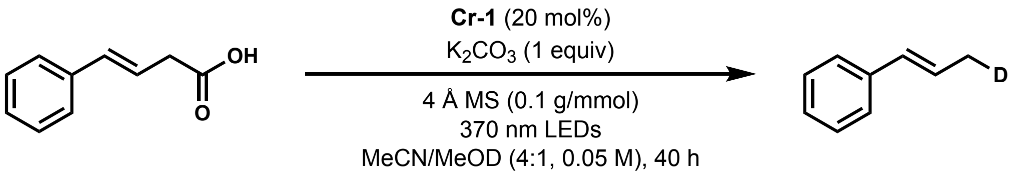

8-d
13%, 50% D incorporation

Inside a N_2-filled glovebox, to an oven-dried reaction vial (VWR, catalog no. 66011-041) equipped with a magnetic stir bar was charged (*E*)-4-phenylbut-3-enoic acid (16 mg, 0.10 mmol, 1.0 equiv), K_2CO_3 (14 mg, 0.10 mmol, 1.0 equiv), 4 Å molecular sieves (10 mg, 0.1 g/mmol), and **Cr-1** (14 mg, 0.020 mmol, 20 mol%). Then MeCN (0.8 mL) and MeOD (0.2 mL) were added via syringe (final concentration: 0.05 M). The reaction vessel was sealed with a PTFE-lined phenolic vial screw cap (Thermo Scientific, catalog no. 03-375-25A with 03-340-10G), removed from the glovebox, and placed in a custom-made photoreactor 3 cm away from two 44 W Kessil PR-160L 370 nm LEDs and one 75 mm fan. The reaction mixture was subjected to LED irradiation at 100% intensity with vigorous stirring (*ca.* 50 °C). After 40 h, the reaction mixture was allowed to cool to room temperature, then filtered through a short silica plug eluting with EtOAc (3 x 2 mL). The crude reaction mixture was subjected to GC-MS analysis with dodecane (8.2 mg) added as an internal standard. Yield of **8-d** was determined by a GC calibration curve between dodecane and *trans*-β-methyl styrene (**8**).

Analysis for Determining Deuterium Incorporation of **8-d**

To determine the percent deuterium incorporation of **8-d**, GC-MS was used (**Figure SI-21**). The ratio of the relative intensities of **8**:**8-d** was considered along with the 9.8% isotope contribution of **8** to the parent mass of **8-d**. The raw data was analyzed using Agilent GCMS Data Analysis Software. The analysis for determining deuterium incorporation in **8-d** is as follows:

8 m/z $[M]^+$ Calcd. for C_9H_{10}: 118.08. Found: 118.1. Relative intensity: 520000. Isotope contribution (9.8%): 50960

8-d m/z $[M+H]^+$ Calcd. for C_9H_9D: 119.08. Found: 119.1. Relative intensity: 580000.

Total contribution of **8-d** to parent mass peak: (580000−50960) = 529040.

Deuterium incorporation: 529040/(529040+520000) = 0.504; therefore 50% deuterium incorporation was detected in **8-d**.

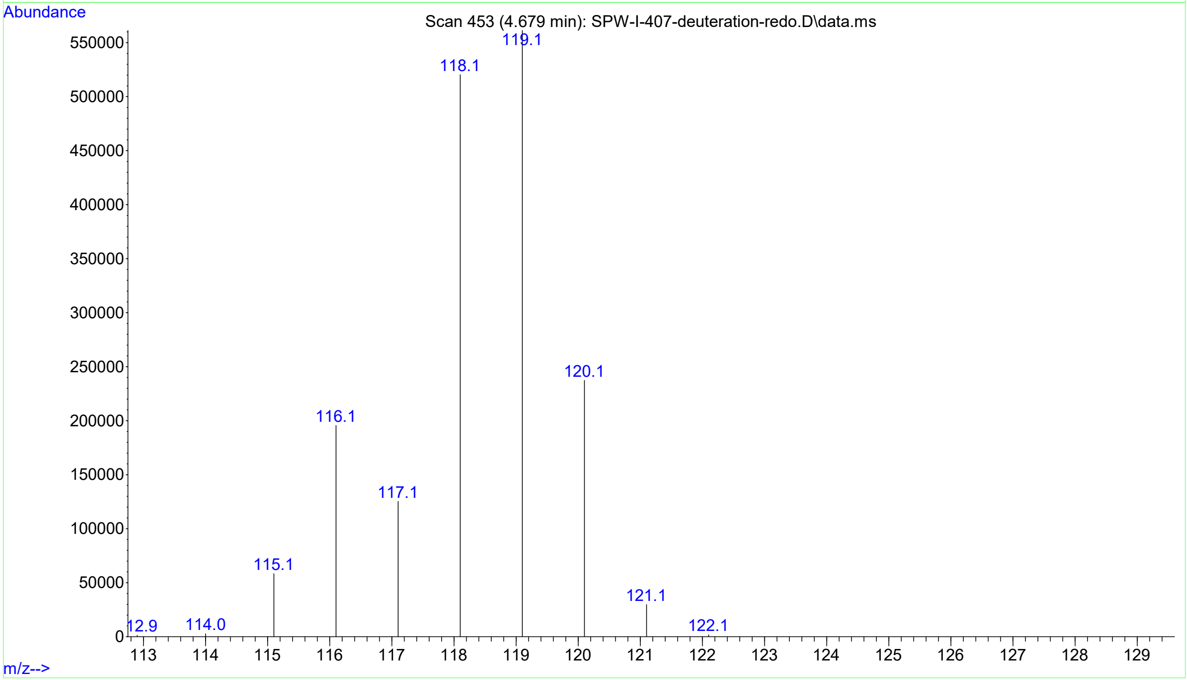

Figure SI-21: Mass spectrum of mixture of **8** and **8-d**.

6.13. *in situ* IR Monitoring of 1g-Si Desilylation

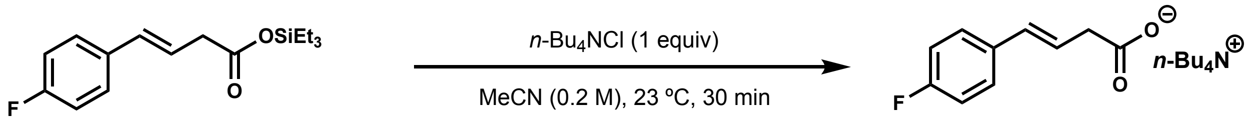

1g-Si
(1 equiv)

SI-17

Inside a N_2-filled glovebox, to an oven-dried 25 mL three-neck round-bottom flask equipped with a magnetic stir bar was added **1g-Si** (177 mg, 0.60 mmol, 1.0 equiv) and MeCN (3.0 mL, 0.2 M). The reaction vessel was sealed with three rubber septa, removed from the glovebox, and placed under a positive pressure of N_2. The central septum was removed and the probe of the *in situ* IR instrument (ReactIR 700 with SiComp probe, AgX 6 mm x 1.5 m Fiber, and Liquid N_2 MCT detector) was attached. IR spectra were collected at 30 s intervals and sampling from 3500 cm^{-1} to 650 cm^{-1}. Once a stable spectrum for **1g-Si** has been acquired (characteristic peak at 1714 cm^{-1}), *n*-BuN_4Cl (167 mg, 0.60 mmol, 1.0 equiv) was added to the reaction mixture under N_2. A new peak at 1724 cm^{-1} (presumably that of **SI-17**) developed while the intensity for the peak corresponding to **1g-Si** diminished. The IR spectra are displayed in Figure **SI-22** and Figure **SI-23**. The IR spectrum for **1g** was collected with the same instrument at 0.2 M in MeCN.

Note: Due to the close proximity of the IR peaks for **1g-K** and **1g-Si**, *n*-Bu_4NCl was utilized instead of KCl for this experiment.

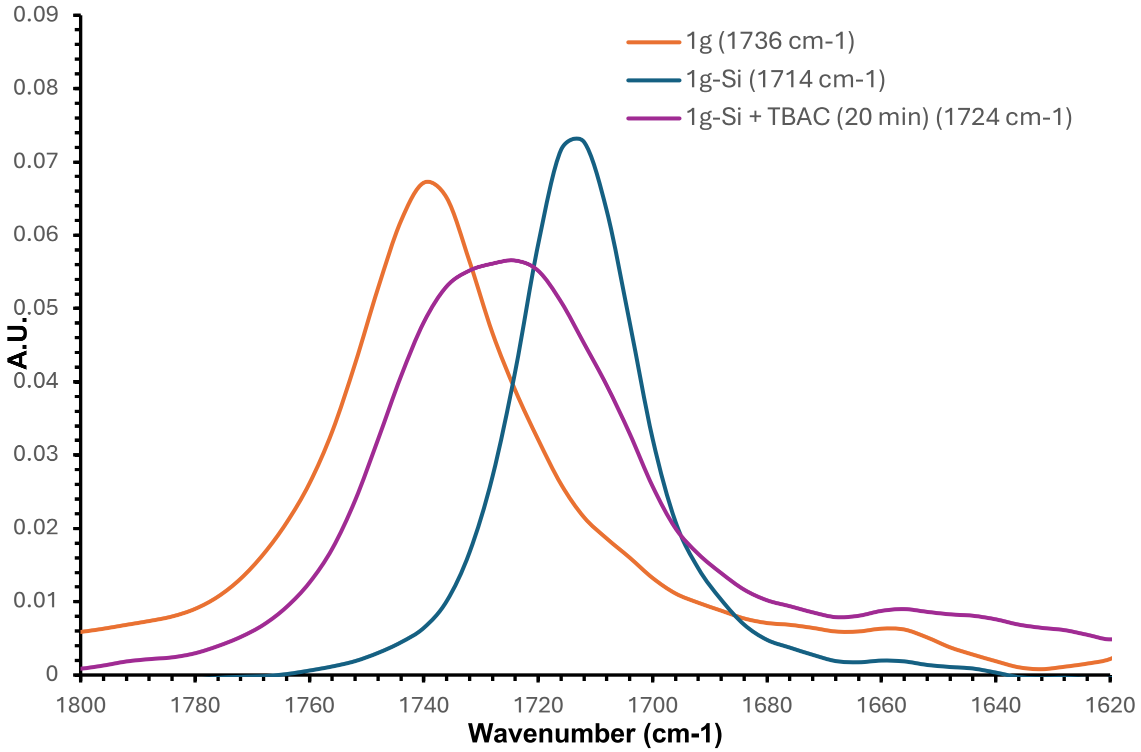

Figure SI-22: IR spectra for **1g**, **1g-Si**, and reaction of **1g-Si** with *n*-Bu_4NCl (TBAC) at 20 min.

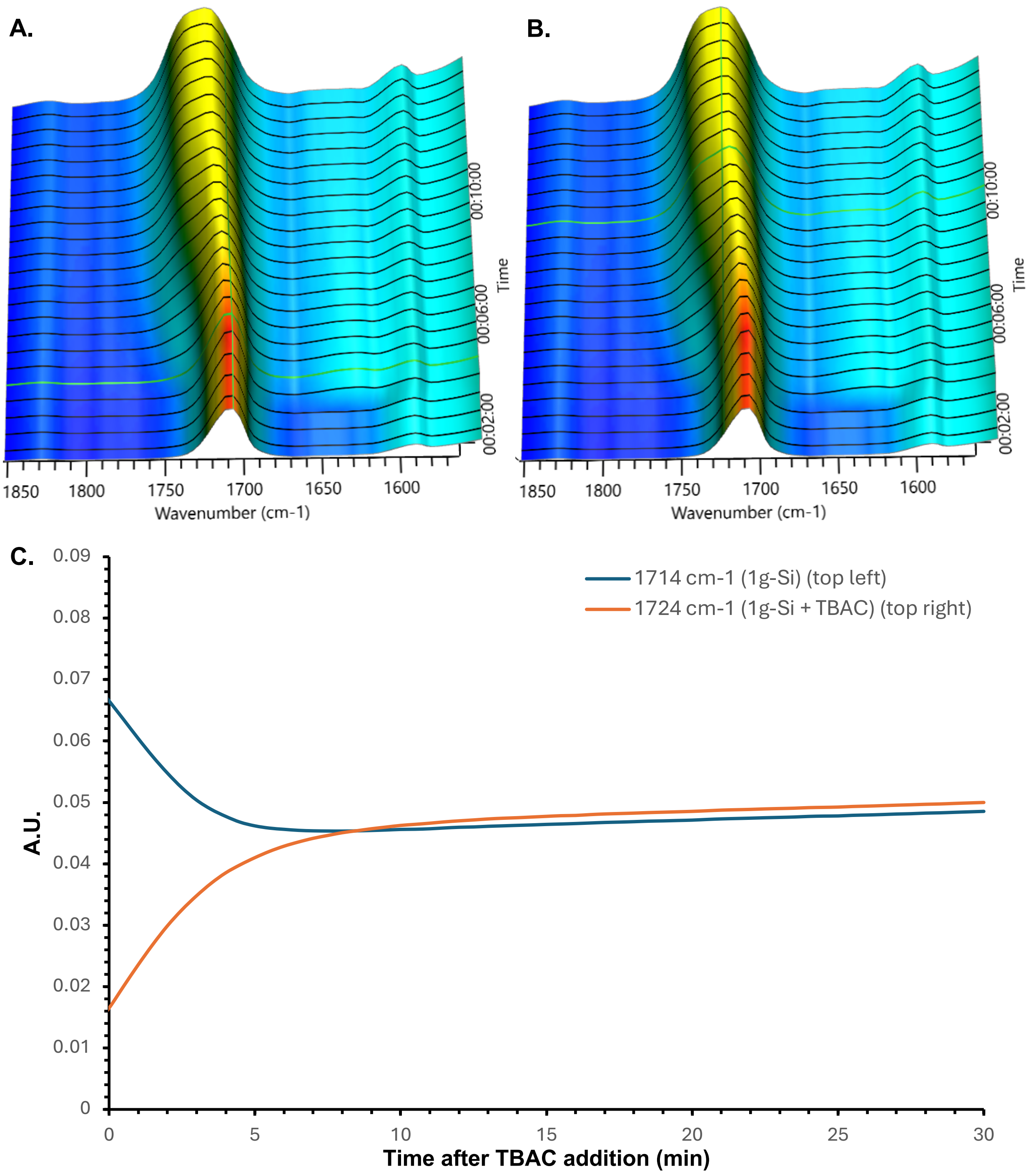

Figure SI-23: *in situ* IR monitoring of **1g-Si** desilylation with *n*-Bu_4NCl. **A**: IR peak at 1714 cm^{-1} that corresponds to **1g-Si**. **B**: IR peak at 1724 cm^{-1} that forms upon the addition of *n*-Bu_4NCl. **C**: Conversion of **1g-Si** to **SI-17** upon the addition of *n*-Bu_4NCl.

The consumption of **1g-Si** concomitant with formation of **SI-17** upon the addition of *n*-Bu_4NCl indicates that Cl^- present in the reaction is capable of desilylating the silylated acid. This provides further support for the silylated acid's role as a plausible intermediate and competent pronucleophile in the reaction (*cf*. Section **6.9**). Together, these experiments indicate that modulation of reaction kinetics through silylation of both the carboxylic acid and potassium carbonate is a possible explanation for the requirement for precise amounts of Et_3SiCl.

Based on these results, an alternative plausible catalytic cycle is proposed in **Figure SI-24**.

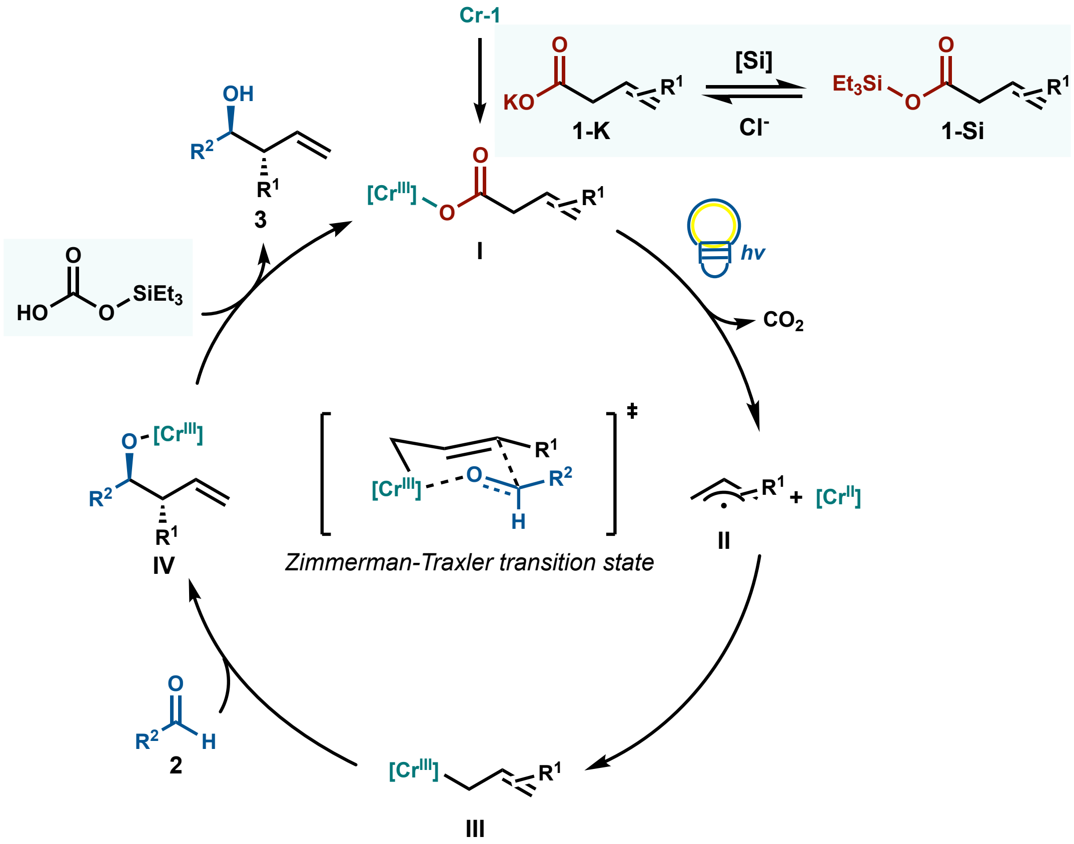

Figure SI-24: Alternative plausible catalytic cycle.

6.14. Demonstrating Regioselectivity of Using Carboxylic Acids as Pronucleophiles over Olefins

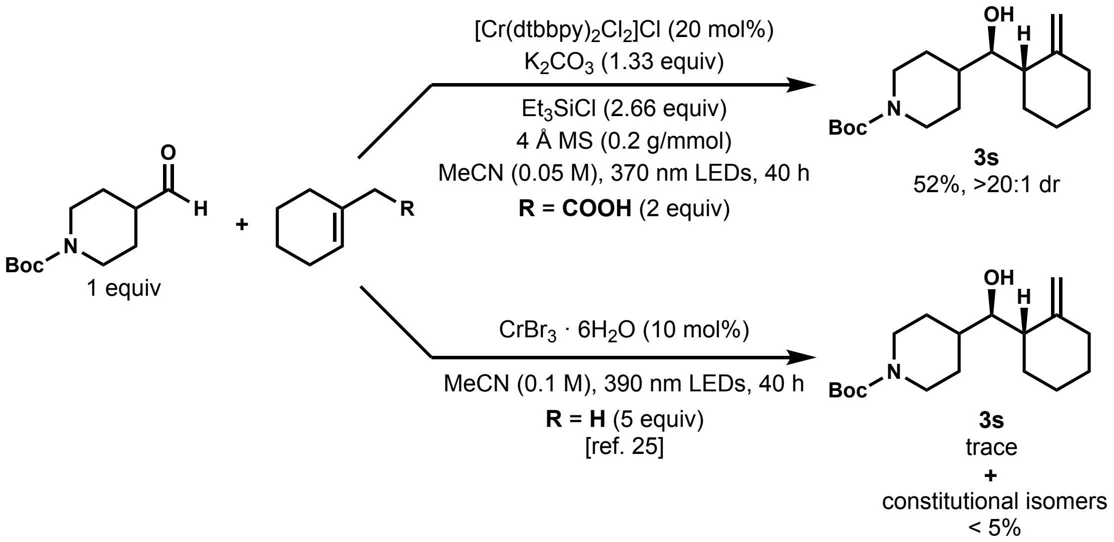

Figure SI-25: Comparison of our decarboxylative allylation method with alternative allylation protocols employing olefin precursors.

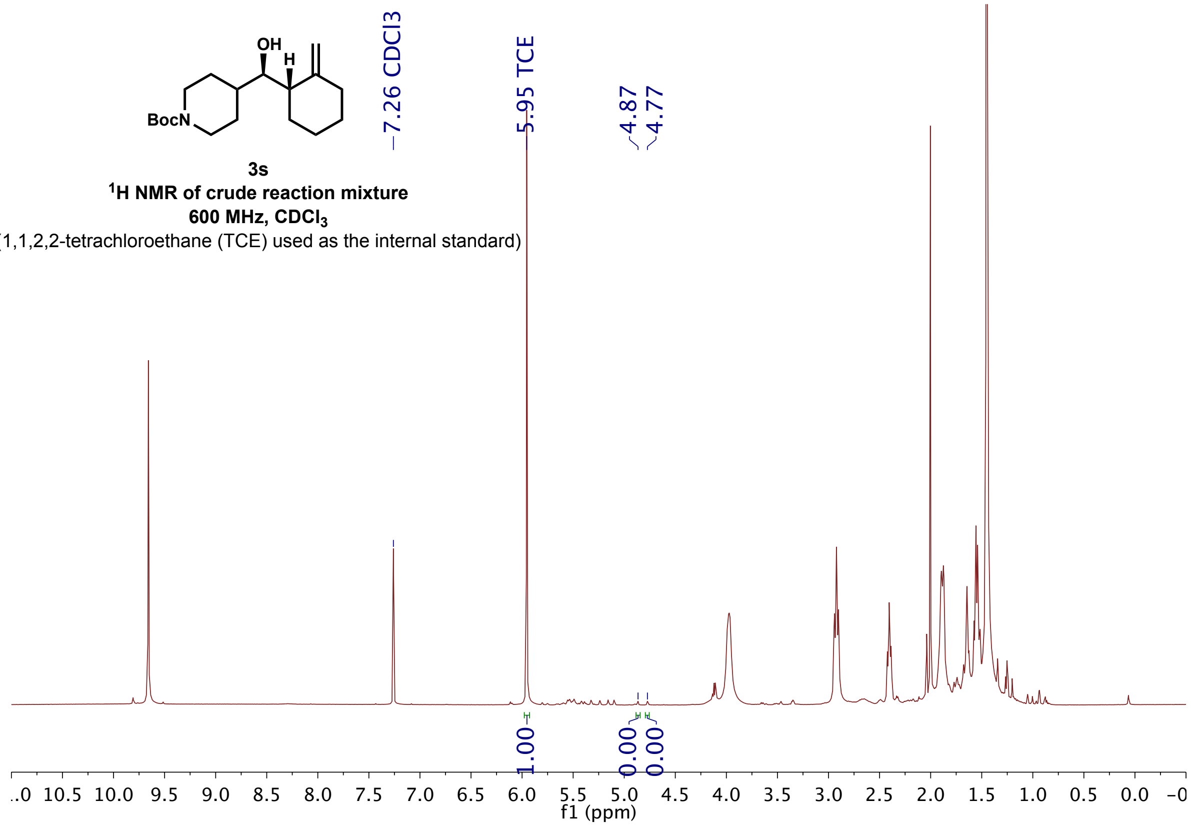

3s

^{1}H NMR of crude reaction mixture

600 MHz, $CDCl_3$

(1,1,2,2-tetrachloroethane (TCE) used as the internal standard)

6.15. Investigating NHK Allylation Efficiency of Cr-1–Cr-3

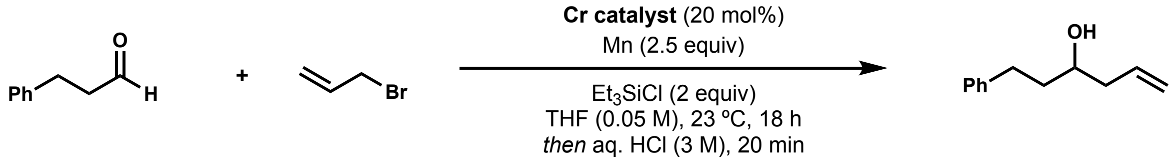

2a (1 equiv)

(2 equiv)

3ad

Cr catalyst	yield
**Cr-1**	43%
**Cr-2**	40%
**Cr-3**	42%

Inside a N_2-filled glovebox, to an oven-dried reaction vial (VWR, catalog no. 66011-041) equipped with a magnetic stir bar was charged **Cr-1** (14 mg, 0.02 mmol, 20 mol%), Mn powder (13.7 mg, 0.25 mmol, 2 equiv), and THF (2 mL, 0.05 M). The reaction mixture was allowed to stir for 15 min, then allyl bromide (17 µL, 0.20 mmol, 2 equiv) was added. The reaction mixture was allowed to stir for another 15 min and 3-phenylpropanal (**2a**) (13 µL, 0.10 mmol, 1 equiv) and Et_3SiCl (34 µL, 0.20 mmol, 2 equiv) were added via syringe. After 18 h, the reaction vessel was removed from the glovebox and the reaction mixture diluted with sat. *aq*. $NaHCO_3$ solution (1 mL), filtered through a short Celite plug eluting with EtOAc (3 x 2 mL), and concentrated *in vacuo* with the aid of a rotary evaporator. To the residue was added THF (3 mL) and 3 M HCl (1 mL), and the reaction mixture was allowed to stir for 20 min at room temperature. After that, the reaction mixture was diluted with H_2O (5 mL), transferred to a separatory funnel, and extracted with EtOAc (3 x 5 mL). The combined organic extracts were washed with brine (5 mL), dried over anhydrous Na_2SO_4, and concentrated *in vacuo* with the aid of a rotary evaporator. A NMR yield was determined by ^1H NMR spectroscopy of the crude reaction mixture using 1,1,2,2-tetrachloroethane (TCE) as the internal standard (43% ^1H NMR yield).

The above procedure was also performed utilizing **Cr-2** or **Cr-3** instead of **Cr-1** to give **3ad** in 40% and 42% yield, respectively. This result suggests that the different bipyridyl ligands on Cr are comparable with respect to NHK allylation efficiency, and thus the difference in performance between **Cr-1** to **Cr-3** in the decarboxylative NHK reaction is likely due to their different competencies in facilitating light-induced photolysis.

All spectroscopic data for **3ad** was consistent with that which was reported above.

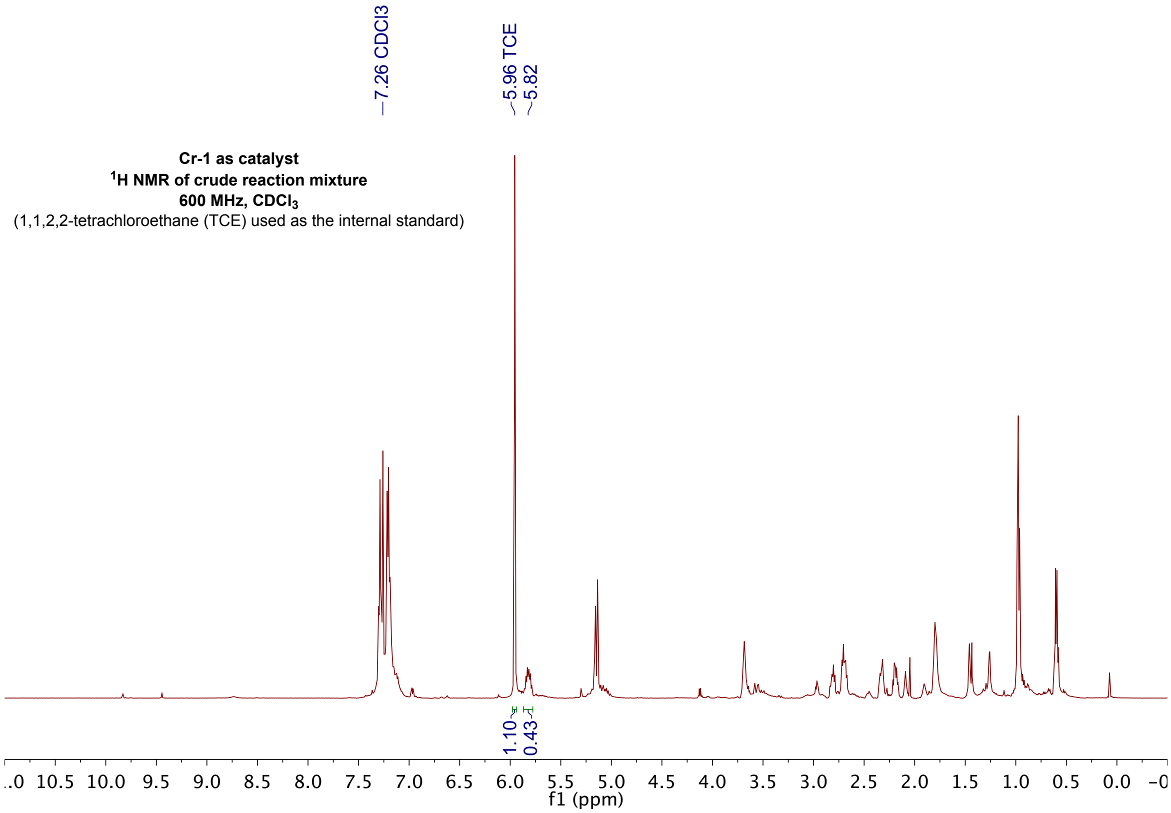
7.26 CDCl3
5.96 TCE
5.82
Cr-1 as catalyst
1H NMR of crude reaction mixture
600 MHz, CDCl3
(1,1,2,2-tetrachloroethane (TCE) used as the internal standard)
1.10
0.43
f1 (ppm)

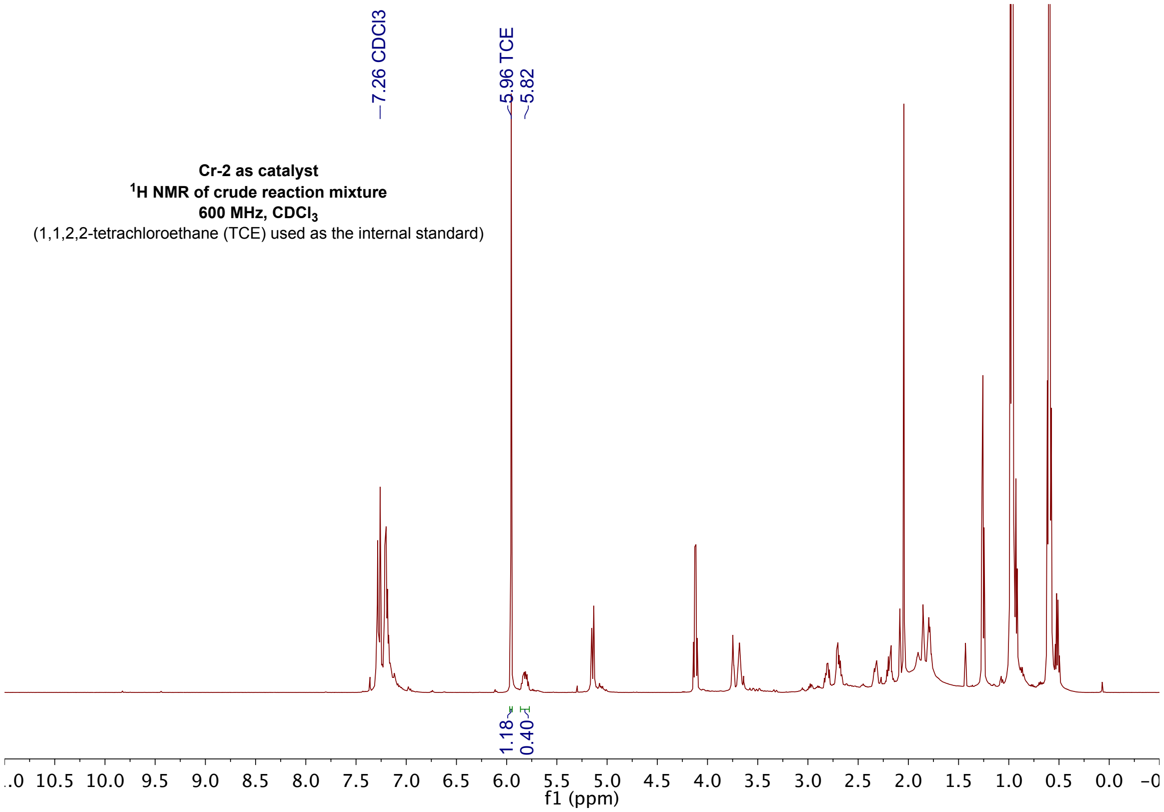
7.26 CDCl3
5.96 TCE
5.82
Cr-2 as catalyst
1H NMR of crude reaction mixture
600 MHz, CDCl3
(1,1,2,2-tetrachloroethane (TCE) used as the internal standard)
1.18
0.40
f1 (ppm)

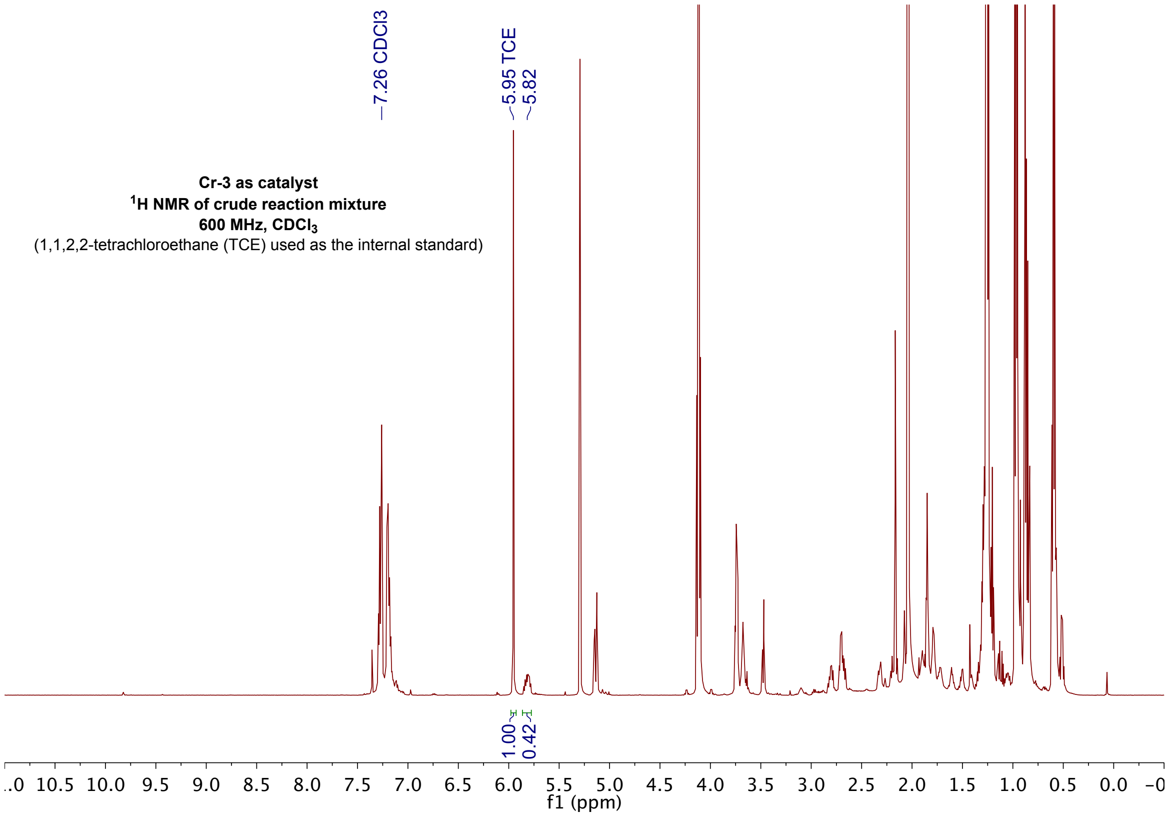
7.26 CDCl3
5.95 TCE
5.82
Cr-3 as catalyst
1H NMR of crude reaction mixture
600 MHz, CDCl3
(1,1,2,2-tetrachloroethane (TCE) used as the internal standard)
1.00
0.42
f1 (ppm)

7. Associated Analytical Data

7.1. Crystallographic Data

Details of crystallographic refinement

General Methods. A suitable crystal of each sample was selected for analysis and mounted in a polyimide loop. Crystal samples were handled under immersion oil and quickly transferred to a cold nitrogen stream. All measurements were made on a Rigaku Oxford Diffraction Supernova Eos CCD with filtered Cu-Kα radiation at a temperature of 100 K. Using Olex2,[29] the structure was solved with the ShelXT structure solution program using Direct Methods and refined with the ShelXL refinement package using Least Squares minimization.[30]

Compound **Cr-1**

Disorder in a *tert*-butyl group was modeled over two orientations with similarity restraints placed on atomic thermal parameters and C–C bond distances. Positional disorder in the outer sphere chloride was modeled over two positions with a similarity restraint placed on their atomic thermal parameters. Hydrogen atoms on the water molecule were added at idealized positions and the orientation of the water molecule was refined as a rigid group.

Compound **Cr-3**

Hydrogen atoms on water were added at fixed positions and refined as part of a rigid group.

Compound **Cr-4**

The structure was refined without additional restraints.

Compound **Cr-7**

The highly disordered diethyl ether was modeled over two symmetry-independent positions using a fragment approach according to the method of Guzei.[31] Their occupancies were refined to sum to 0.5.

Compound **Cr-8**

Positional disorder in the carboxylate ligand was modeled over four positions with similarity restraints placed on atomic bond distances and thermal parameters. Positional disorder in the chloride ligands was modeled over two positions with similarity restraints placed on atomic bond distances and thermal parameters. The occupancies of the partially occupied carboxylates were constrained using three SUMP commands to restrain the free variables: The sum of the occupancies of the two carboxylate positions at each site plus the occupancy of the chloride at that site were constrained to be equal to 1, and the sum of the two chloride occupancies was constrained to be equal to 1.

Table **SI-1**. Crystal data and structure refinement for **Cr-1**.

Identification code	CCDC 2431980	
Empirical formula	$C_{36}H_{50}Cl_3CrN_4O$	
Formula weight	713.15	
Temperature	100.00(10) K	
Wavelength	1.54184 Å	
Crystal system	Orthorhombic	
Space group	Pbca	
Unit cell dimensions	a = 20.1128(2) Å	α = 90°
	b = 14.11500(10) Å	β = 90°
	c = 26.2860(3) Å	γ = 90°
Volume	7462.39(12) Å^3	
Z	8	
Density (calculated)	1.270 Mg/m^3	
Absorption coefficient	4.748 mm^{-1}	
F(000)	3016	
Crystal size	0.26 x 0.07 x 0.03 mm^3	
Theta range for data collection	3.363 to 71.766°.	
Index ranges	-22<=h<=24, -16<=k<=17, -31<=l<=31	
Reflections collected	40570	
Independent reflections	7259 [R(int) = 0.0448]	
Completeness to theta = 67.684°	100.0 %	
Absorption correction	Gaussian	
Max. and min. transmission	1.000 and 0.436	
Refinement method	Full-matrix least-squares on F^2	
Data / restraints / parameters	7259 / 75 / 462	
Goodness-of-fit on F^2	1.018	
Final R indices [I>2sigma(I)]	R1 = 0.0506, wR2 = 0.1301	
R indices (all data)	R1 = 0.0582, wR2 = 0.1368	
Extinction coefficient	n/a	
Largest diff. peak and hole	0.502 and -0.683 e/Å^{-3}	

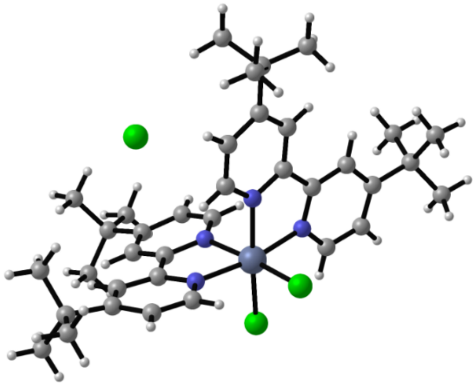

Figure SI-26. Crystal data and structure refinement for **Cr-1** (CCDC 2431980), one molecule of H_2O in unit cell omitted for clarity.

Table **SI-2**. Crystal data and structure refinement for **Cr-3**.

Identification code	CCDC 2431981	
Empirical formula	$C_{24}H_{20}Cl_3CrN_4O_2$	
Formula weight	554.79	
Temperature	99.9(2) K	
Wavelength	1.54184 Å	
Crystal system	Orthorhombic	
Space group	Pbcn	
Unit cell dimensions	a = 12.58910(10) Å	α = 90°
	b = 23.2531(2) Å	β = 90°
	c = 16.3545(2) Å	γ = 90°
Volume	4787.54(8) $Å^3$	
Z	8	
Density (calculated)	1.539 Mg/m^3	
Absorption coefficient	7.267 mm^{-1}	
F(000)	2264	
Crystal size	0.1 x 0.07 x 0.03 mm^3	
Theta range for data collection	3.802 to 71.548°.	
Index ranges	-15<=h<=15, -28<=k<=26, -19<=l<=17	
Reflections collected	24715	
Independent reflections	4631 [R(int) = 0.0473]	
Completeness to theta = 67.684°	100.0 %	
Absorption correction	Gaussian	
Max. and min. transmission	0.858 and 0.645	
Refinement method	Full-matrix least-squares on F^2	
Data / restraints / parameters	4631 / 0 / 309	
Goodness-of-fit on F^2	1.050	
Final R indices [I>2sigma(I)]	R1 = 0.0436, wR2 = 0.1098	
R indices (all data)	R1 = 0.0507, wR2 = 0.1148	
Extinction coefficient	n/a	
Largest diff. peak and hole	1.197 and -0.974 $e/Å^{-3}$	

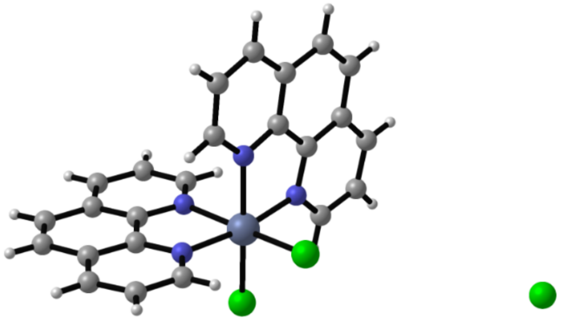

Figure SI-27. Crystal data and structure refinement for **Cr-3** (CCDC 2431981), two molecules of H_2O in unit cell omitted for clarity.

Table **SI-3**. Crystal data and structure refinement for **Cr-4**.

Identification code	CCDC 2431982	
Empirical formula	$C_{15}H_{11}Cl_3CrN_3$	
Formula weight	391.62	
Temperature	100.3(6) K	
Wavelength	1.54184 Å	
Crystal system	Monoclinic	
Space group	P 1 21/n 1	
Unit cell dimensions	a = 8.3058(6) Å	α = 90°
	b = 14.0484(12) Å	β = 104.661(7)°
	c = 13.6529(10) Å	γ = 90°
Volume	1541.2(2) Å^3	
Z	4	
Density (calculated)	1.688 Mg/m^3	
Absorption coefficient	10.873 mm^{-1}	
F(000)	788	
Crystal size	0.18 x 0.12 x 0.05 mm^3	
Theta range for data collection	4.595 to 66.586°.	
Index ranges	-9<=h<=9, -16<=k<=13, -15<=l<=16	
Reflections collected	11239	
Independent reflections	2707 [R(int) = 0.0779]	
Completeness to theta = 66.586°	99.5 %	
Absorption correction	Gaussian	
Max. and min. transmission	0.759 and 0.291	
Refinement method	Full-matrix least-squares on F^2	
Data / restraints / parameters	2707 / 0 / 199	
Goodness-of-fit on F^2	1.031	
Final R indices [I>2sigma(I)]	R1 = 0.0733, wR2 = 0.2006	
R indices (all data)	R1 = 0.0792, wR2 = 0.2125	
Extinction coefficient	n/a	
Largest diff. peak and hole	0.936 and -0.813 e/Å^{-3}	

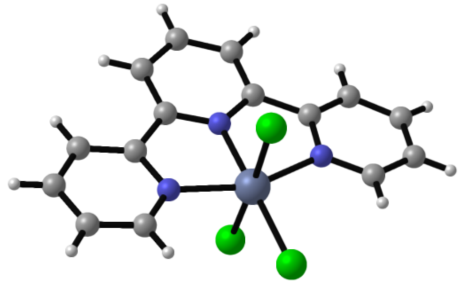

Figure SI-28. Crystal data and structure refinement for **Cr-4** (CCDC 2431982).

Table **SI-4**. Crystal data and structure refinement for **Cr-7**.

Identification code	CCDC 2431983	
Empirical formula	$C_{52}H_{67}Cl_2CrN_5O_{2.5}$	
Formula weight	925.00	
Temperature	100.01(10) K	
Wavelength	1.54184 Å	
Crystal system	Monoclinic	
Space group	P 1 21/c 1	
Unit cell dimensions	a = 14.9503(3) Å	α = 90°
	b = 11.3824(2) Å	β = 99.636(2)°
	c = 29.4762(6) Å	γ = 90°
Volume	4945.20(17) Å^3	
Z	4	
Density (calculated)	1.242 Mg/m^3	
Absorption coefficient	3.245 mm^{-1}	
F(000)	1968	
Crystal size	0.11 x 0.06 x 0.03 mm^3	
Theta range for data collection	2.998 to 71.601°.	
Index ranges	-15<=h<=18, -14<=k<=13, -33<=l<=36	
Reflections collected	53893	
Independent reflections	9480 [R(int) = 0.0728]	
Completeness to theta = 67.684°	99.4 %	
Absorption correction	Semi-empirical from equivalents	
Max. and min. transmission	1.00000 and 0.61932	
Refinement method	Full-matrix least-squares on F^2	
Data / restraints / parameters	9480 / 275 / 674	
Goodness-of-fit on F^2	1.033	
Final R indices [I>2sigma(I)]	R1 = 0.0603, wR2 = 0.1596	
R indices (all data)	R1 = 0.0878, wR2 = 0.1796	
Extinction coefficient	n/a	
Largest diff. peak and hole	0.813 and -0.531 e/Å^{-3}	

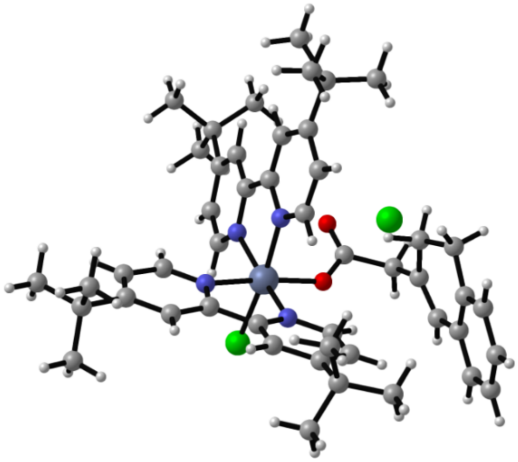

Figure SI-29. Crystal data and structure refinement for **Cr-7** (CCDC 2431983), one molecule of MeCN and 0.5 molecules of Et_2O omitted for clarity.

Table **SI-5**. Crystal data and structure refinement for **Cr-8**.

Identification code	CCDC 2448375	
Empirical formula	$C_{50}H_{66}Cl_2CrFN_4O_3$	
Formula weight	912.96	
Temperature	100.01(10) K	
Wavelength	1.54184 Å	
Crystal system	Monoclinic	
Space group	P 1 21/c 1	
Unit cell dimensions	a = 18.8317(8) Å	α = 90°
	b = 12.6880(4) Å	β = 107.029(3)°
	c = 23.5637(5) Å	γ = 90°
Volume	5383.4(3) $Å^3$	
Z	4	
Density (calculated)	1.126 Mg/m^3	
Absorption coefficient	3.003 mm^{-1}	
F(000)	1940	
Crystal size	0.23 x 0.15 x 0.02 mm^3	
Theta range for data collection	3.924 to 67.078°.	
Index ranges	-22<=h<=22, -15<=k<=9, -28<=l<=28	
Reflections collected	39159	
Independent reflections	9579 [R(int) = 0.0636]	
Completeness to theta = 67.078°	99.8 %	
Absorption correction	Semi-empirical from equivalents	
Max. and min. transmission	1.00000 and 0.67710	
Refinement method	Full-matrix least-squares on F^2	
Data / restraints / parameters	9579 / 858 / 840	
Goodness-of-fit on F^2	1.030	
Final R indices [I>2sigma(I)]	R1 = 0.0844, wR2 = 0.2318	
R indices (all data)	R1 = 0.1245, wR2 = 0.2677	
Extinction coefficient	n/a	
Largest diff. peak and hole	0.576 and -0.388 e/$Å^{-3}$	

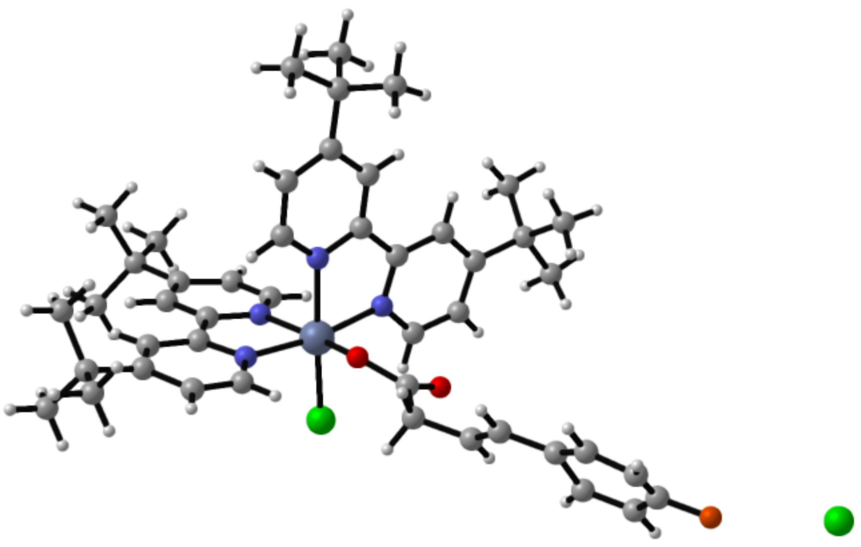

Figure SI-30. Crystal data and structure refinement for **Cr-8** (CCDC 2448375), one molecule of Et_2O omitted for clarity.

7.2. Associated NMR Spectra

7.2.1. NMR Spectra of Starting Materials

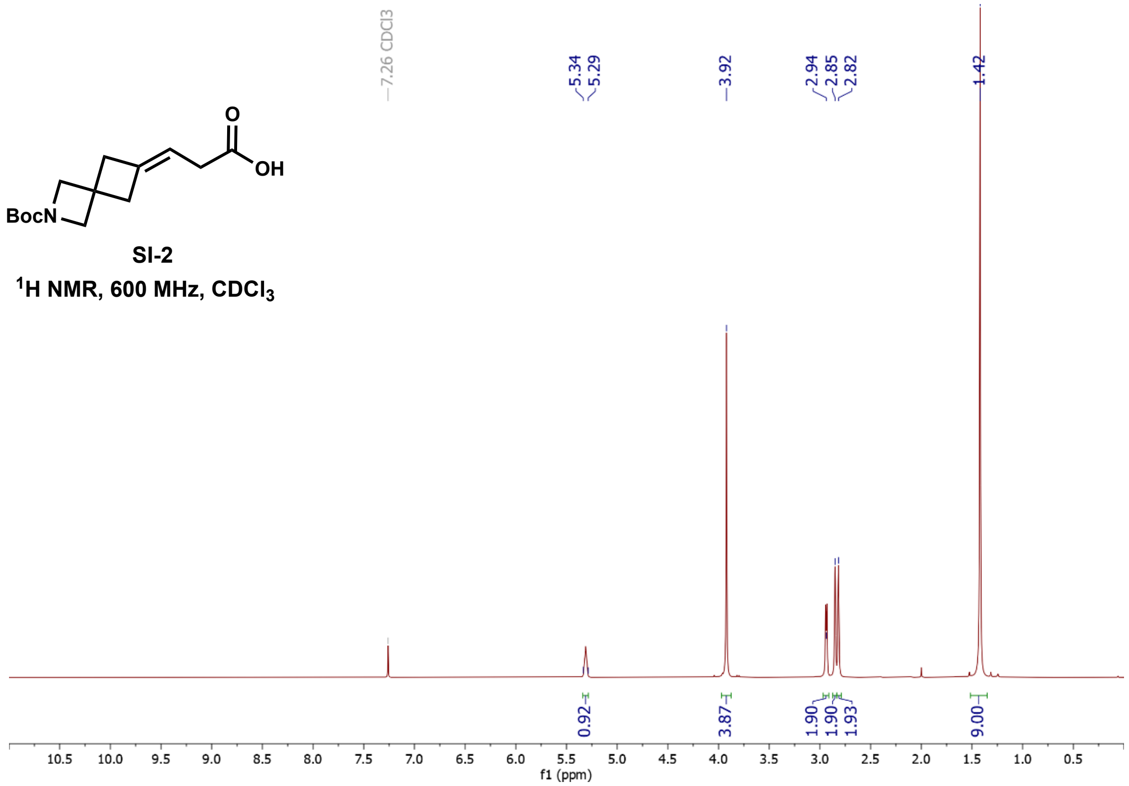

SI-2
^{1}H NMR, 600 MHz, $CDCl_3$

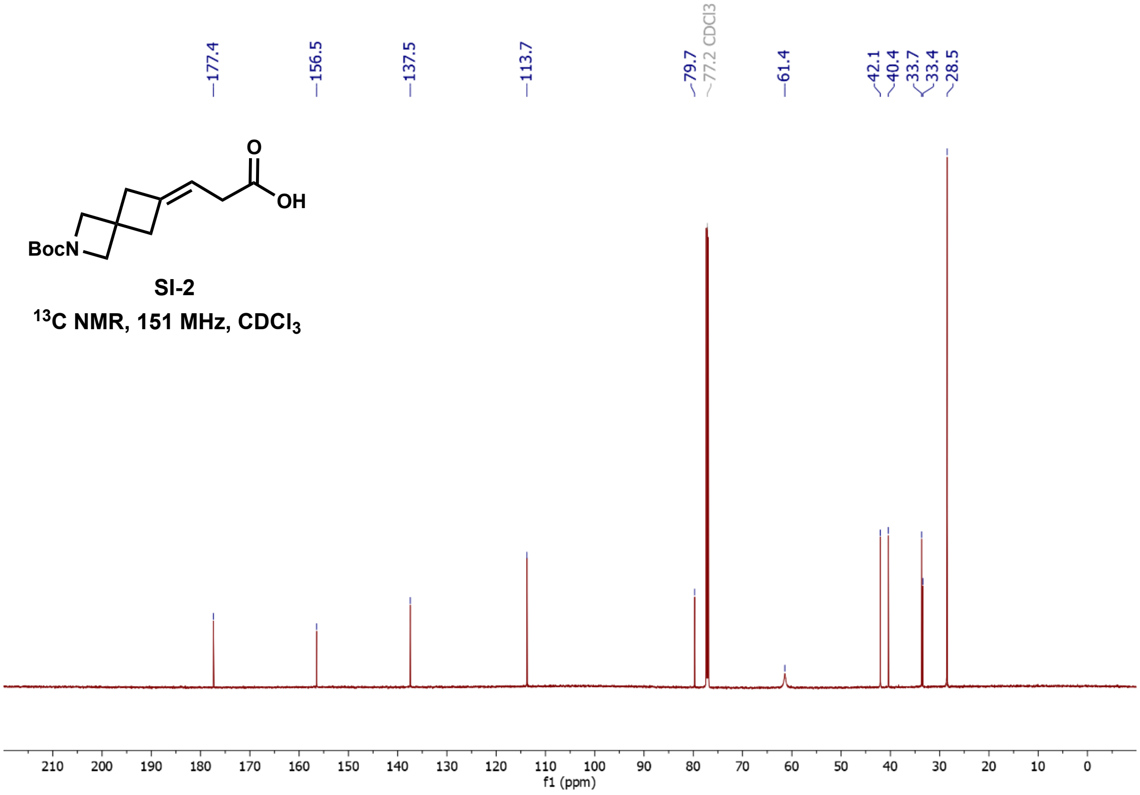

SI-2
^{13}C NMR, 151 MHz, $CDCl_3$

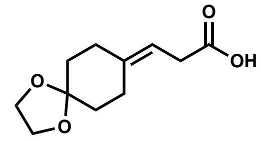

SI-3

^{1}H NMR, 600 MHz, $CDCl_3$

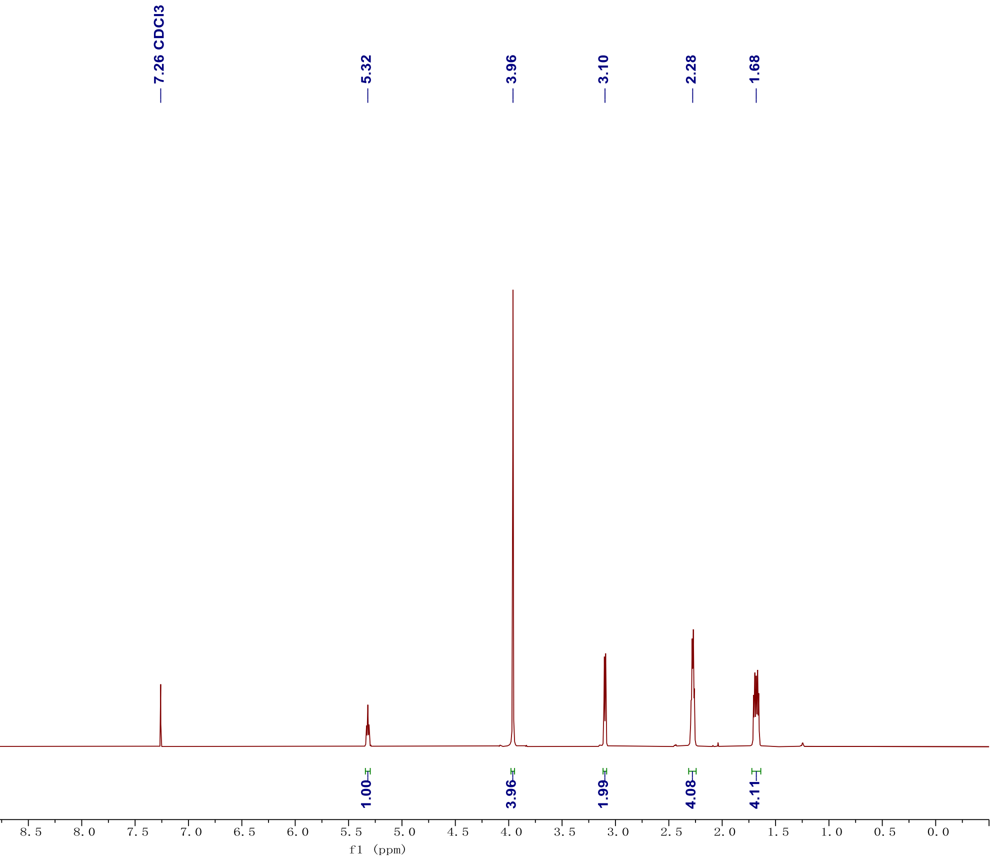

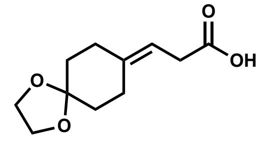

SI-3

^{13}C NMR, 151 MHz, $CDCl_3$

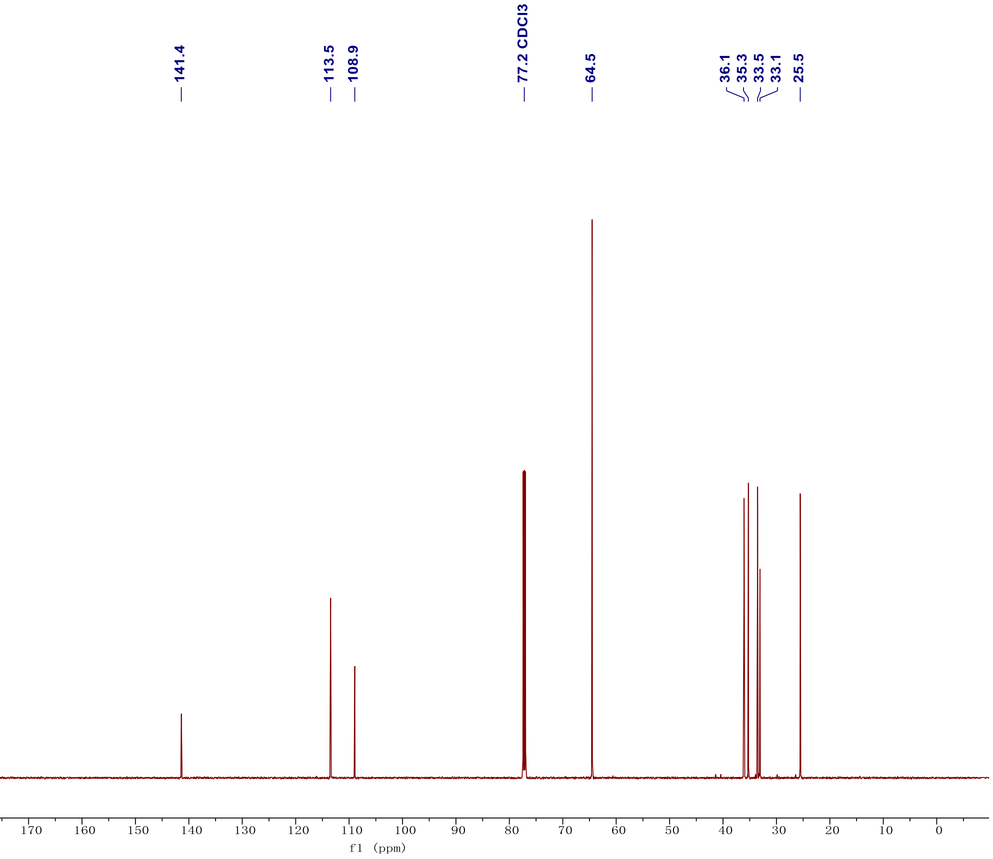

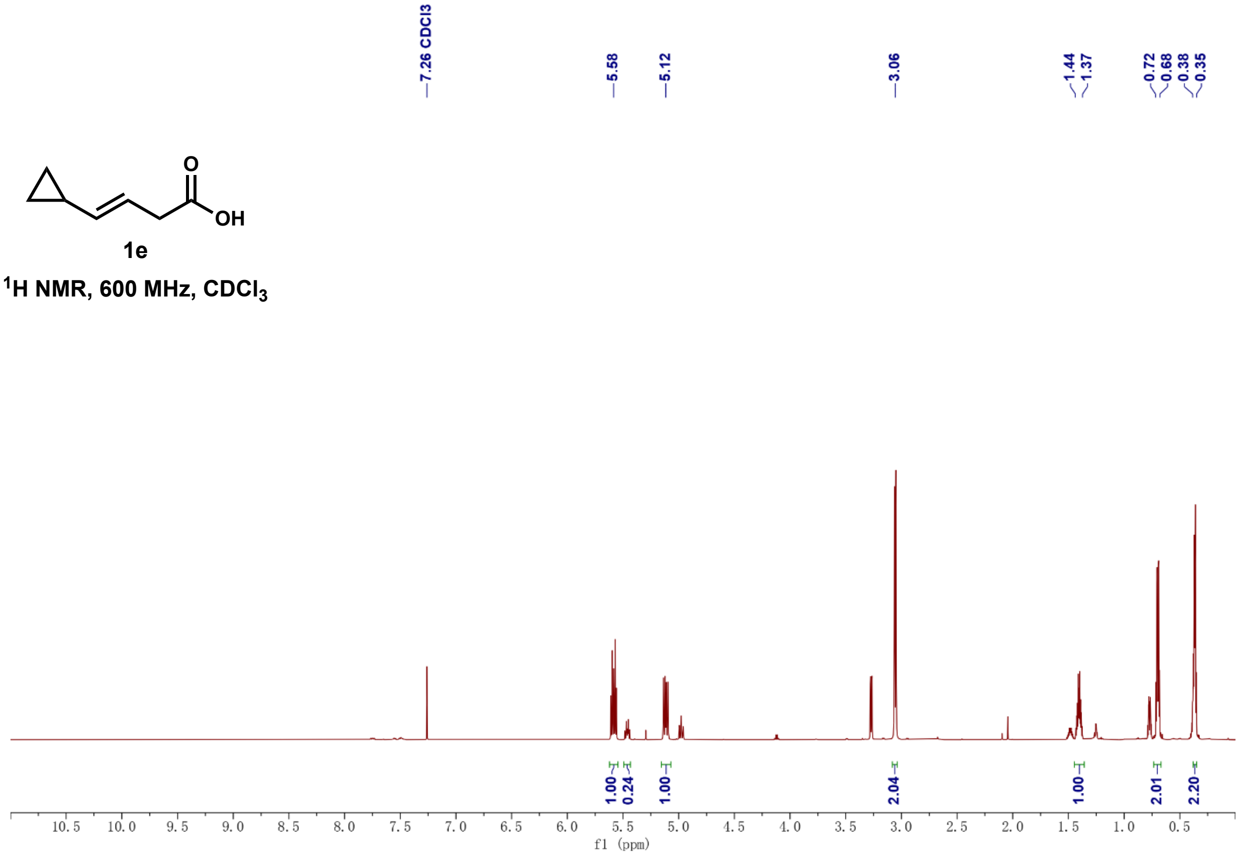
1e
^{1}H NMR, 600 MHz, $CDCl_3$
7.26 CDCl3
5.58
5.12
3.06
1.44
1.37
0.72
0.68
0.38
0.35
1.00
0.24
1.00
2.04
1.00
2.01
2.20
f1 (ppm)

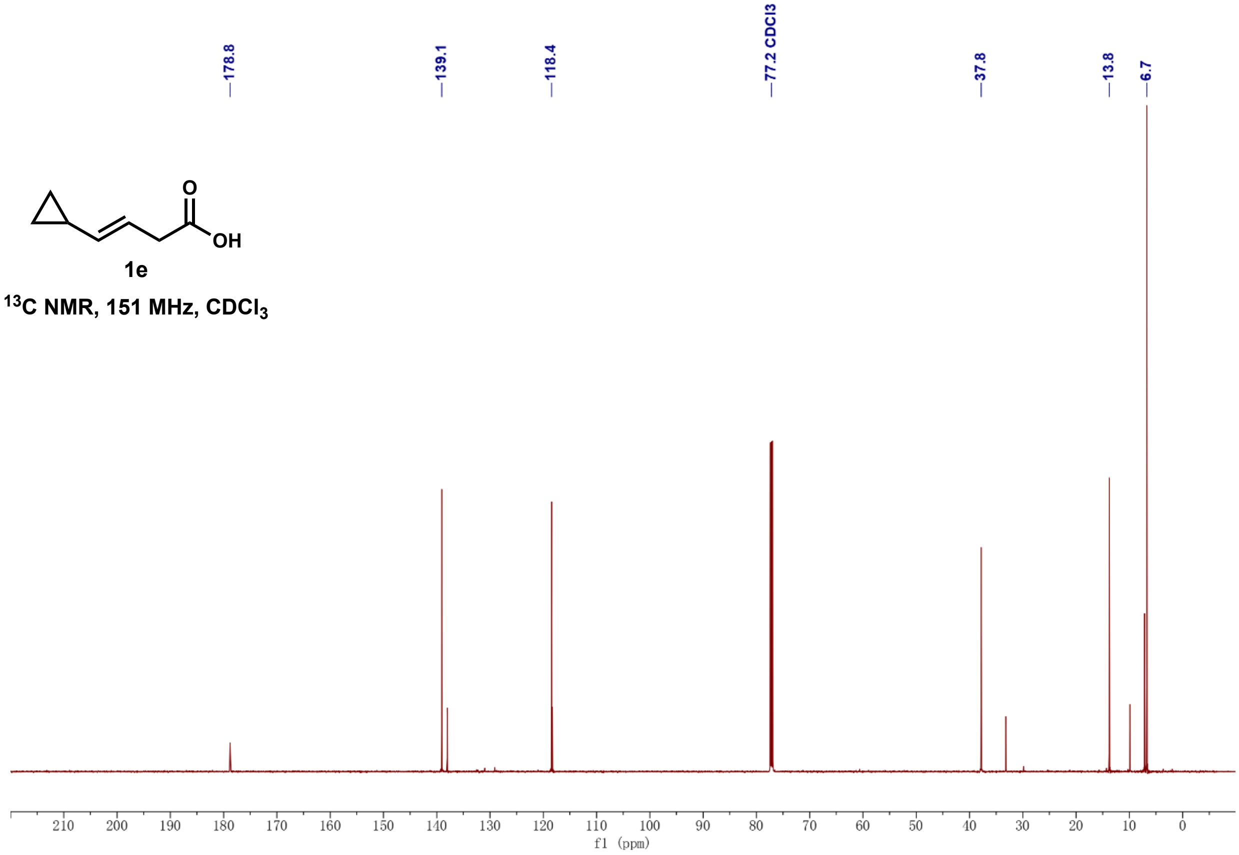
1e
^{13}C NMR, 151 MHz, $CDCl_3$
178.8
139.1
118.4
77.2 CDCl3
37.8
13.8
6.7
f1 (ppm)

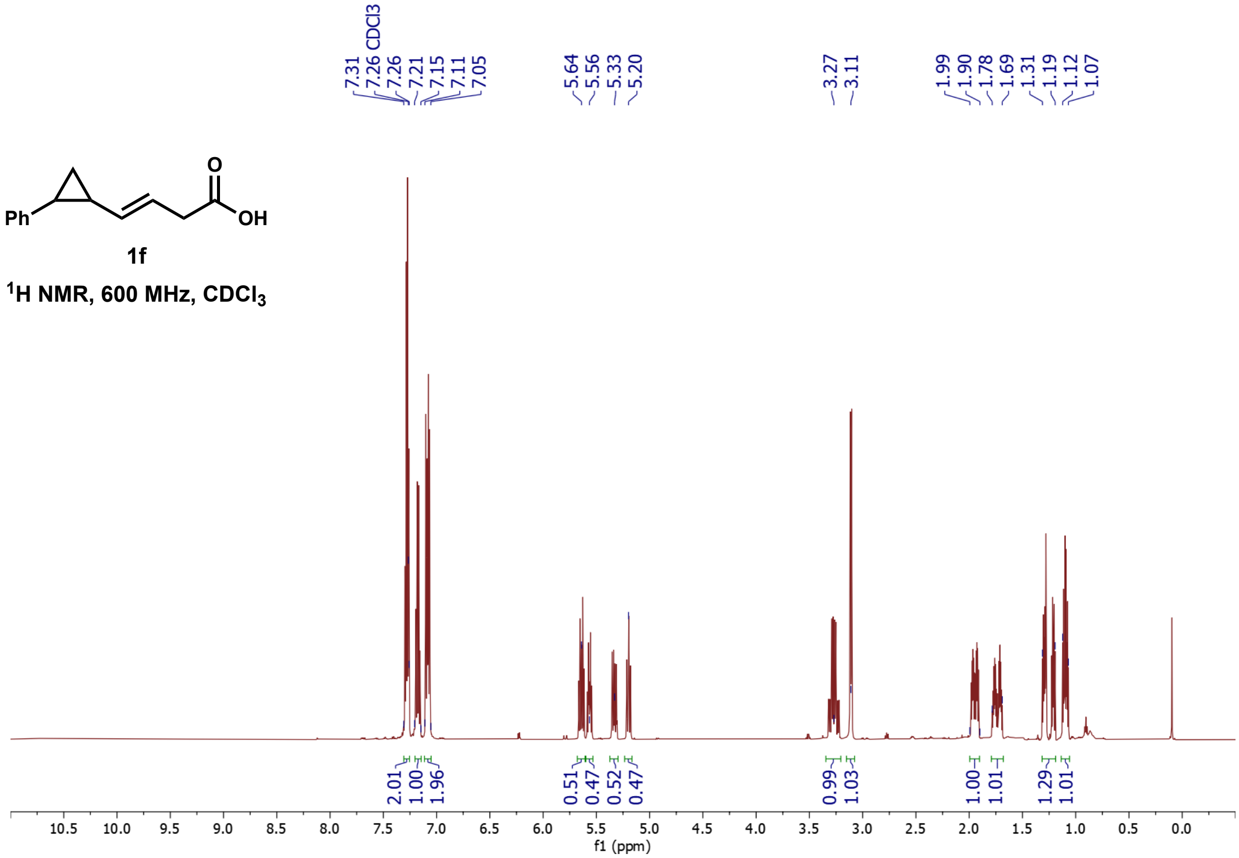
7.31 7.26 CDCl3 7.26 7.21 7.15 7.11 7.05
5.64 5.56 5.33 5.20
3.27 3.11
1.99 1.90 1.78 1.69 1.31 1.19 1.12 1.07
O
Ph
OH
1f
1H NMR, 600 MHz, CDCl3
2.01 1.00 1.96
0.51 0.47 0.52 0.47
0.99 1.03
1.00 1.01
1.29 1.01
10.5 10.0 9.5 9.0 8.5 8.0 7.5 7.0 6.5 6.0 5.5 5.0 4.5 4.0 3.5 3.0 2.5 2.0 1.5 1.0 0.5 0.0
f1 (ppm)

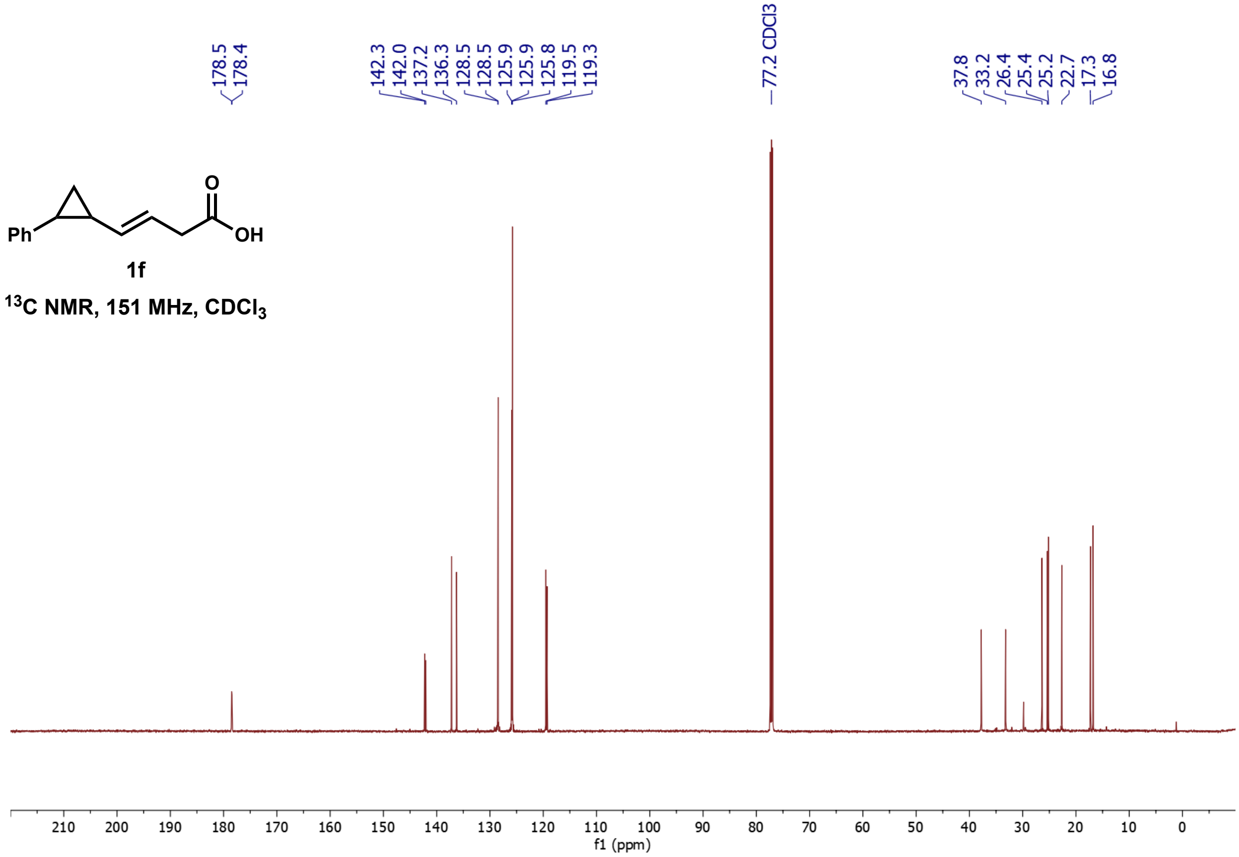
178.5 178.4
142.3 142.0 137.2 136.3 128.5 128.5 125.9 125.9 125.8 119.5 119.3
77.2 CDCl3
37.8 33.2 26.4 25.4 25.2 22.7 17.3 16.8
O
Ph
OH
1f
13C NMR, 151 MHz, CDCl3
210 200 190 180 170 160 150 140 130 120 110 100 90 80 70 60 50 40 30 20 10 0
f1 (ppm)

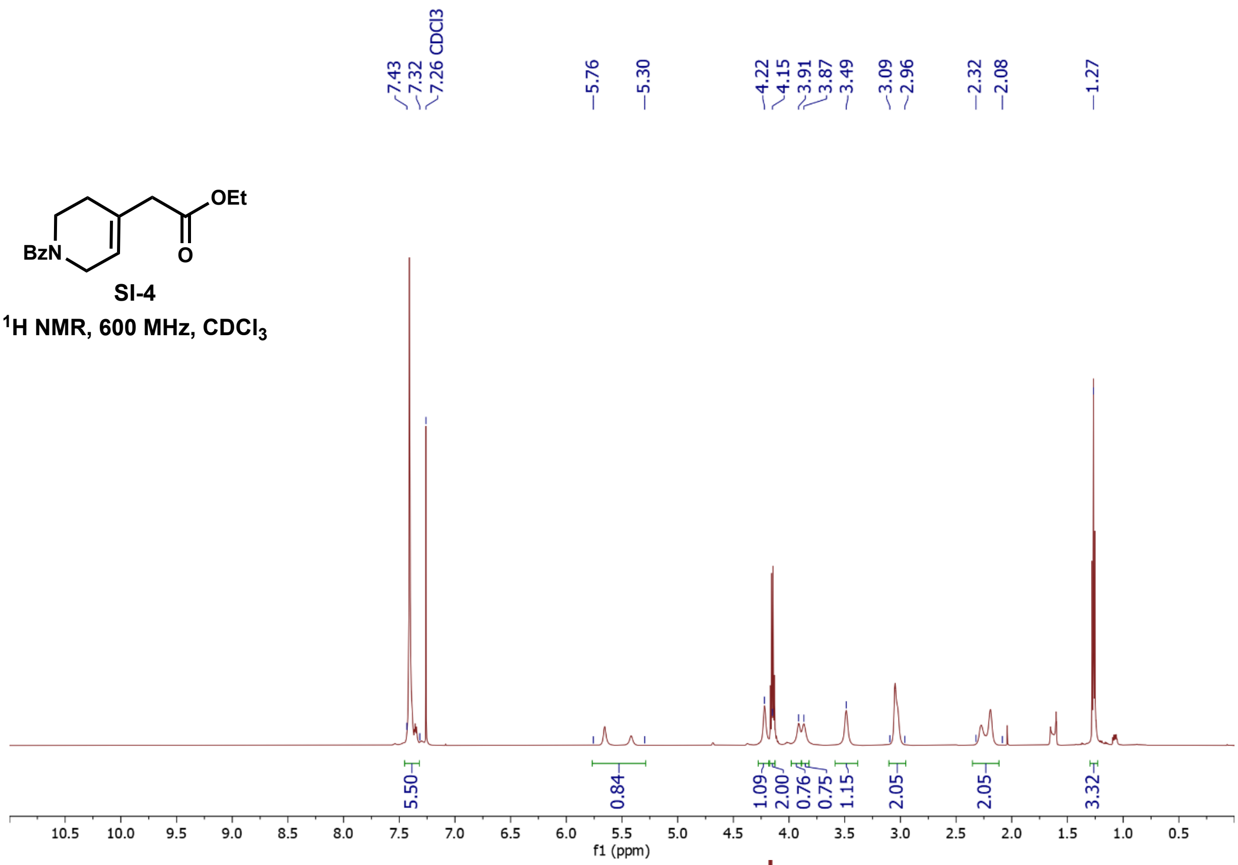

SI-4

^{1}H NMR, 600 MHz, CDCl$_3$

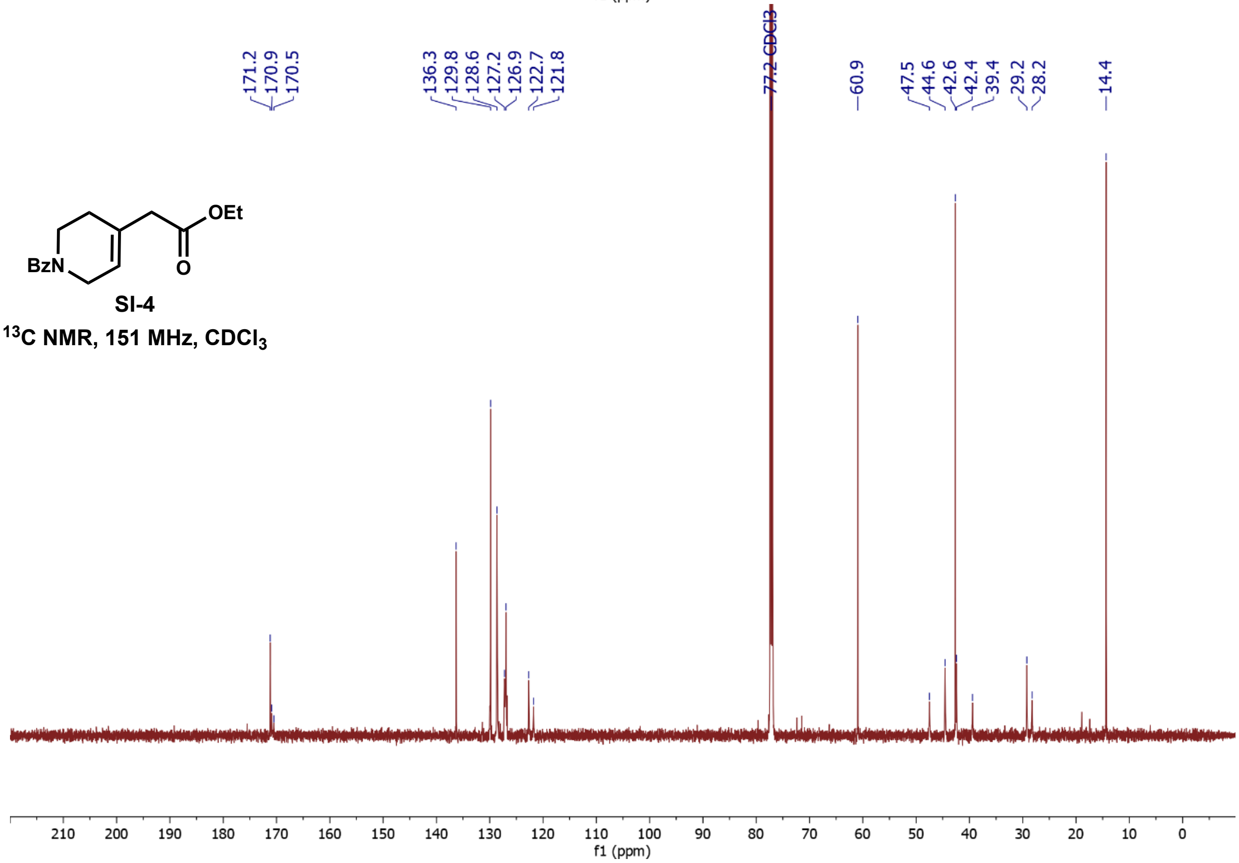

SI-4

^{13}C NMR, 151 MHz, CDCl$_3$

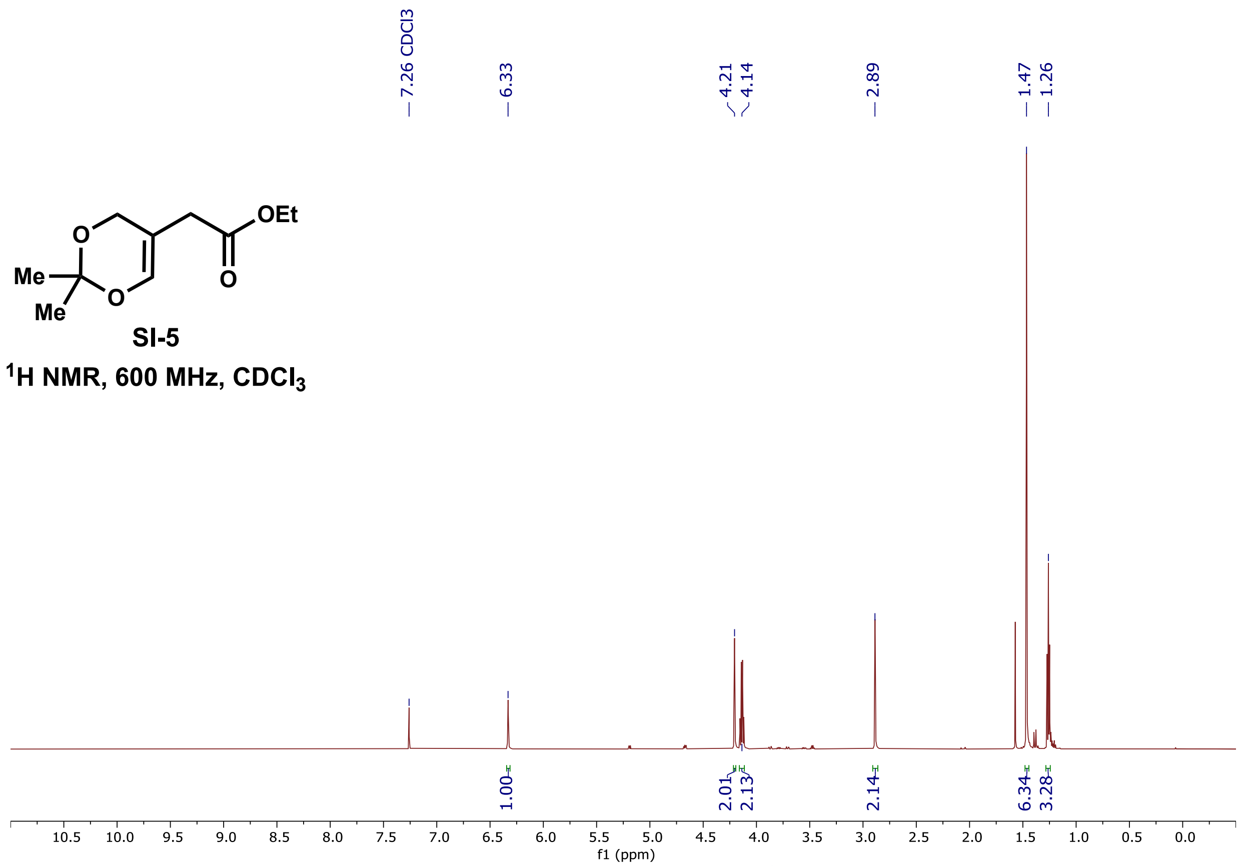
7.26 CDCl3
6.33
4.21
4.14
2.89
1.47
1.26
OEt
O
Me
Me
SI-5
^{1}H NMR, 600 MHz, $CDCl_3$
1.00
2.01
2.13
2.14
6.34
3.28
10.5 10.0 9.5 9.0 8.5 8.0 7.5 7.0 6.5 6.0 5.5 5.0 4.5 4.0 3.5 3.0 2.5 2.0 1.5 1.0 0.5 0.0
f1 (ppm)

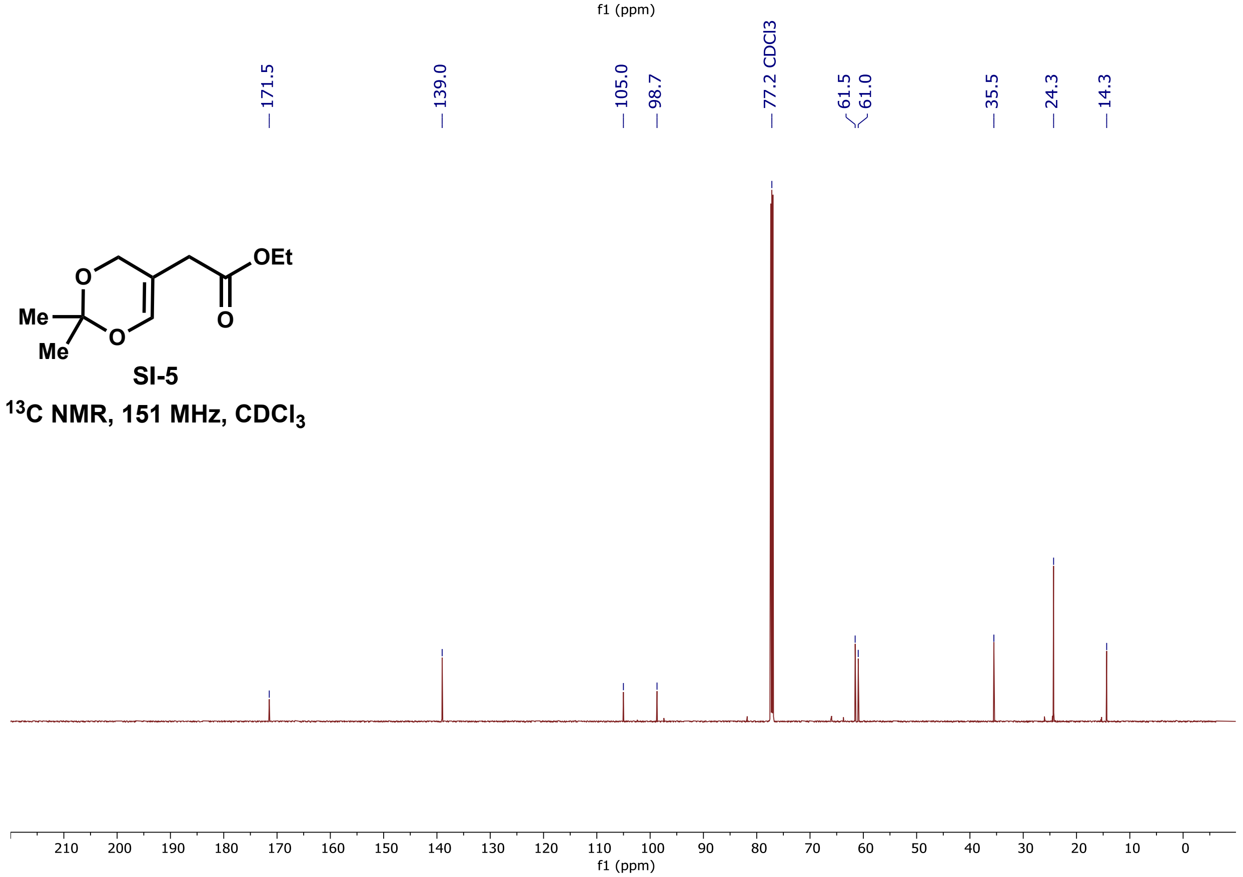
171.5
139.0
105.0
98.7
77.2 CDCl3
61.5
61.0
35.5
24.3
14.3
OEt
O
Me
Me
SI-5
^{13}C NMR, 151 MHz, $CDCl_3$
210 200 190 180 170 160 150 140 130 120 110 100 90 80 70 60 50 40 30 20 10 0
f1 (ppm)

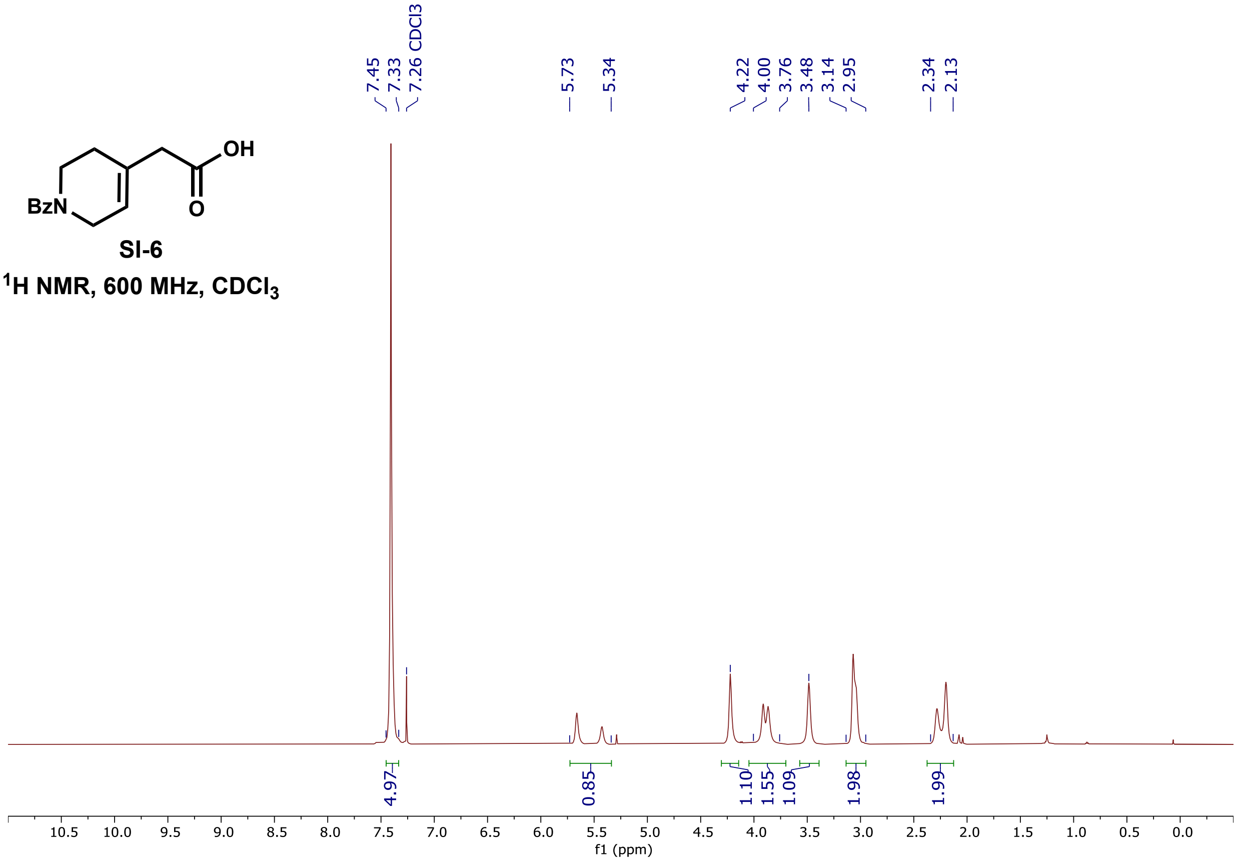

SI-6

^{1}H NMR, 600 MHz, $CDCl_3$

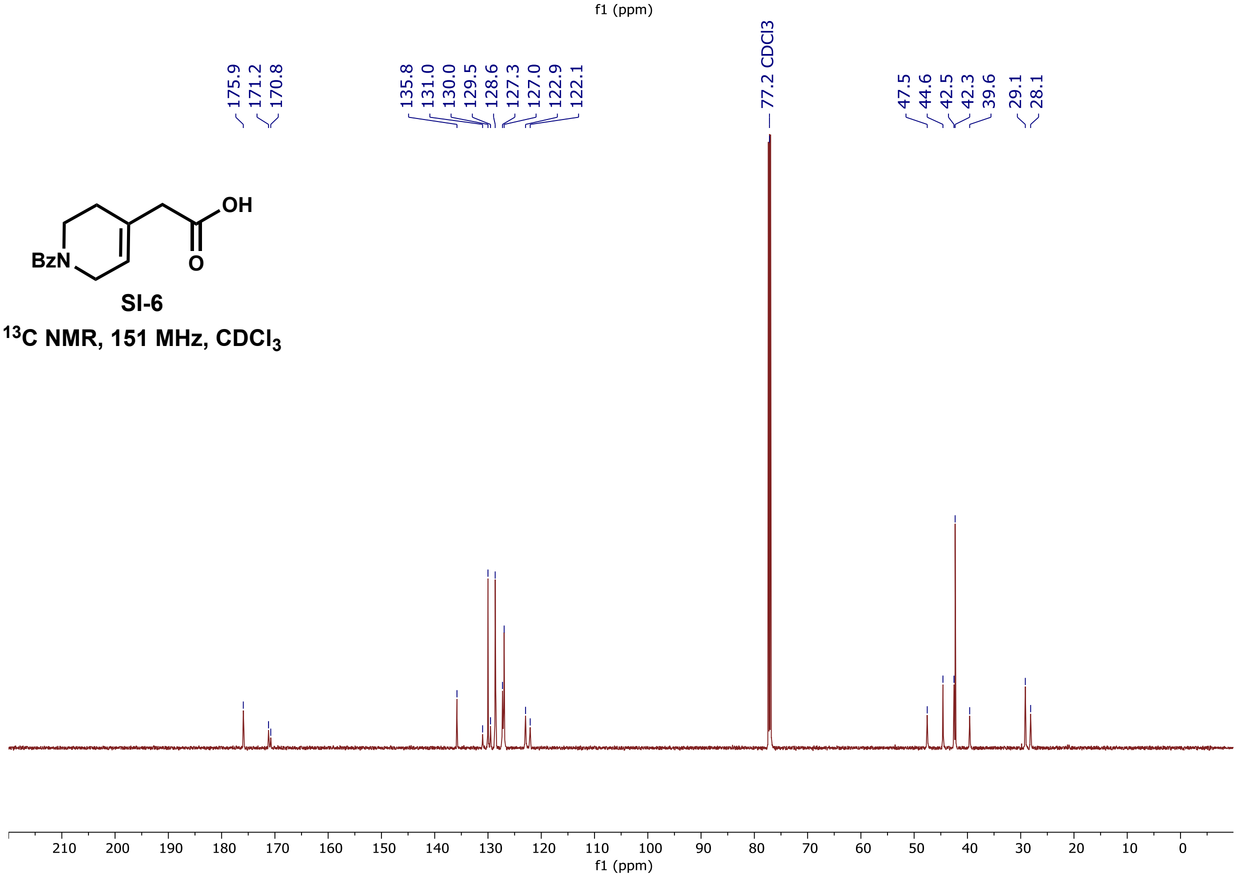

SI-6

^{13}C NMR, 151 MHz, $CDCl_3$

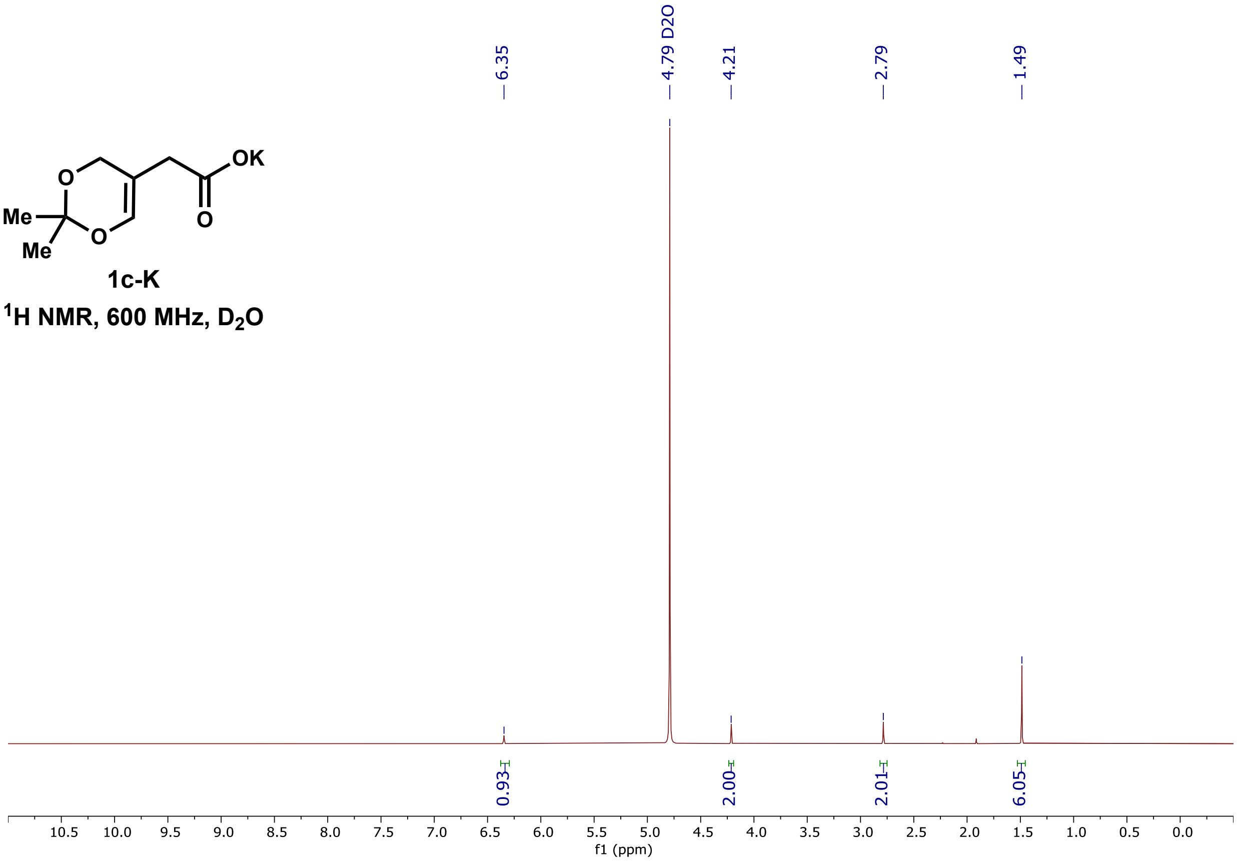

1c-K

^{1}H NMR, 600 MHz, D_2O

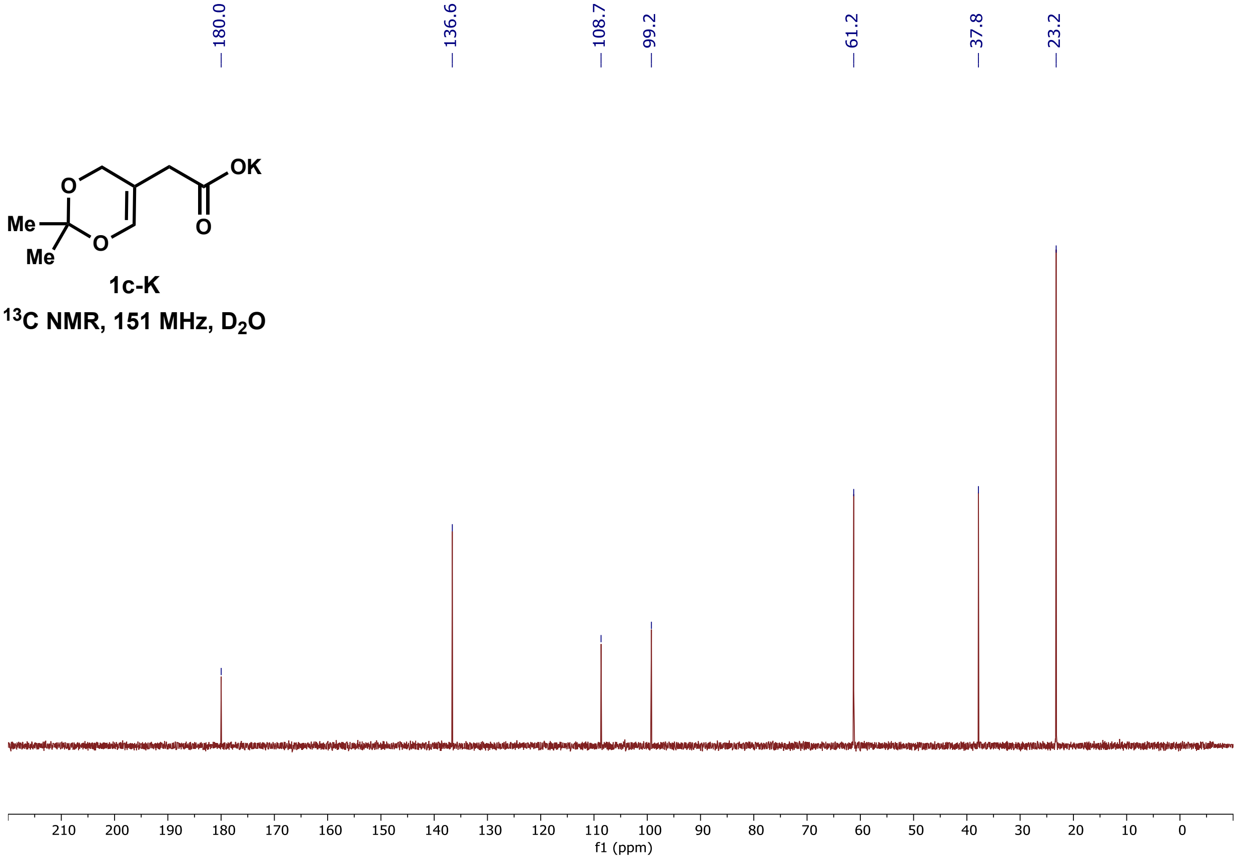

1c-K

^{13}C NMR, 151 MHz, D_2O

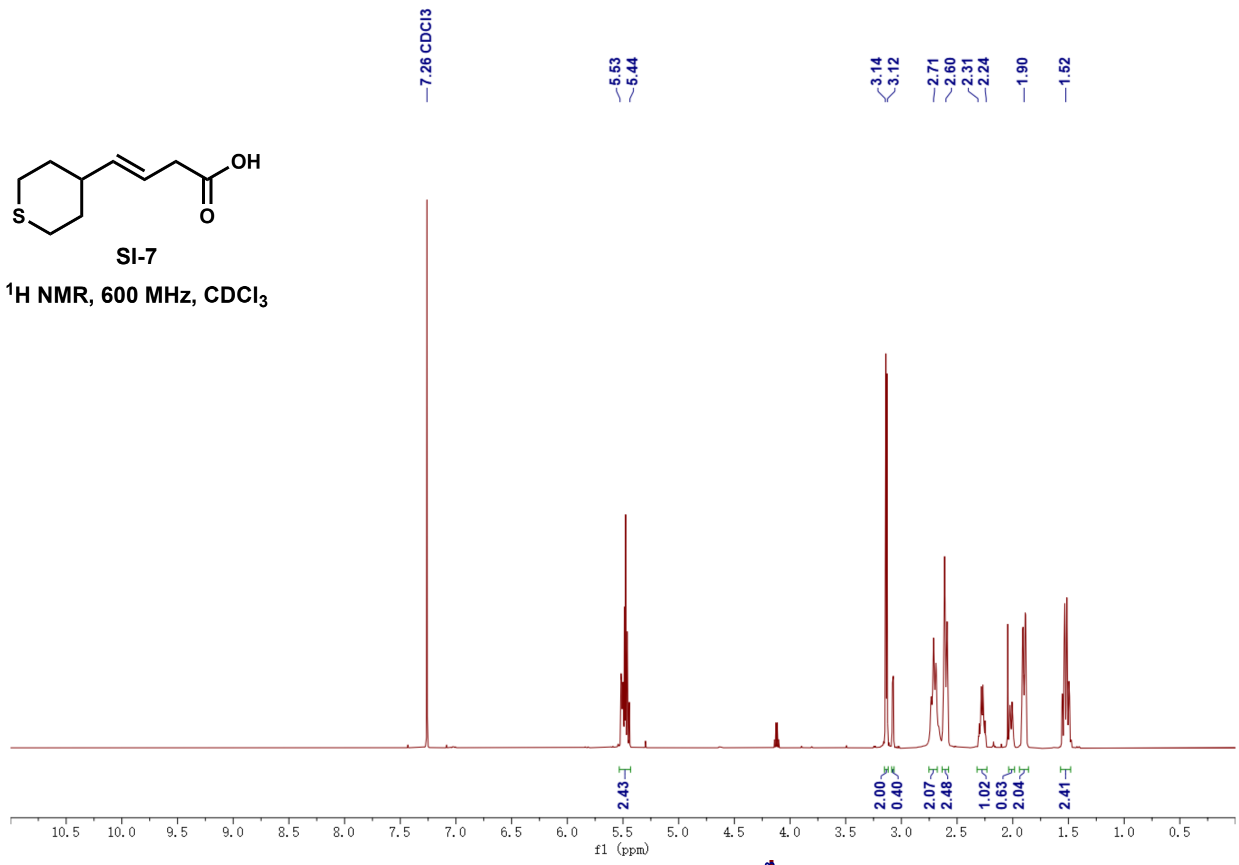
7.26 CDCl3
5.53
5.44
3.14
3.12
2.71
2.60
2.31
2.24
1.90
1.52
OH
O
S
SI-7
^{1}H NMR, 600 MHz, $CDCl_3$
2.43
2.00
0.40
2.07
2.48
1.02
0.63
2.04
2.41
10.5 10.0 9.5 9.0 8.5 8.0 7.5 7.0 6.5 6.0 5.5 5.0 4.5 4.0 3.5 3.0 2.5 2.0 1.5 1.0 0.5
f1 (ppm)

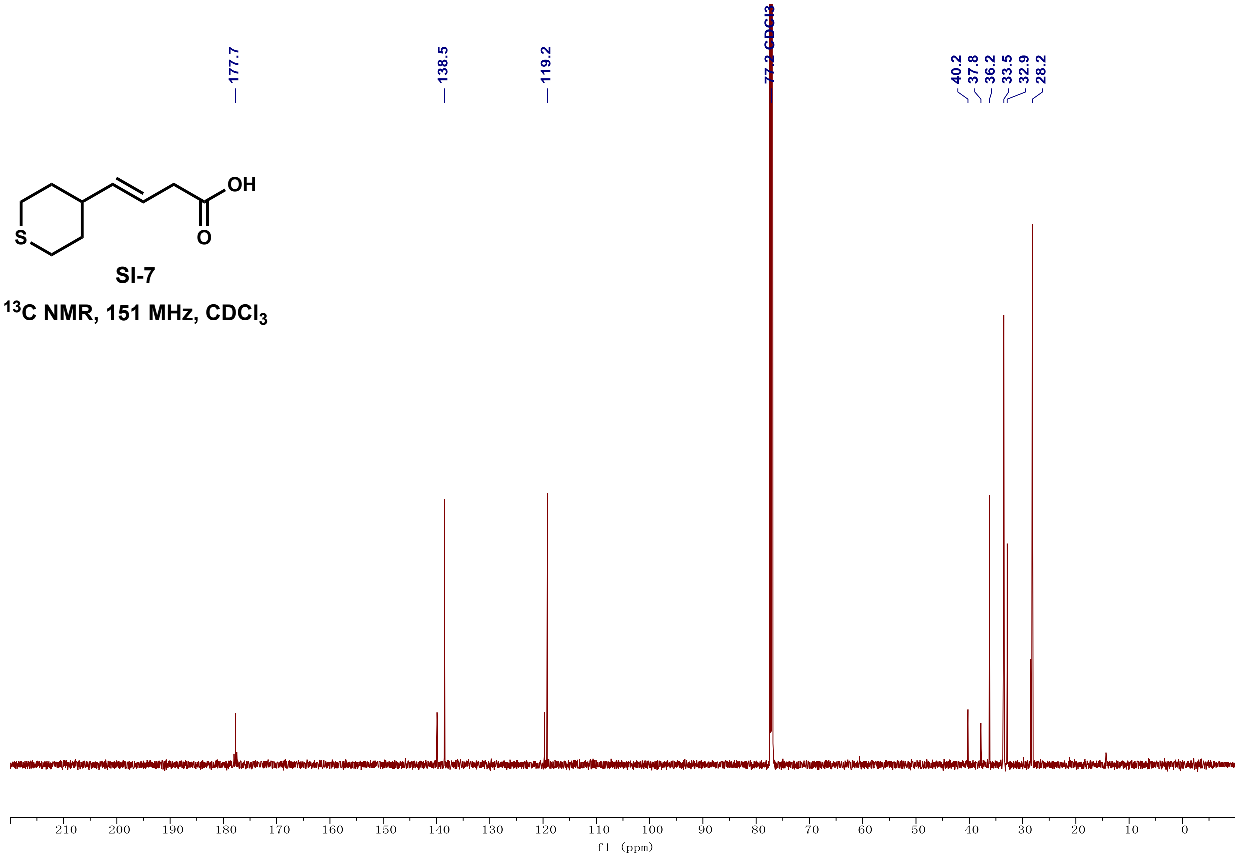
177.7
138.5
119.2
77.2 CDCl3
40.2
37.8
36.2
33.5
32.9
28.2
OH
O
S
SI-7
^{13}C NMR, 151 MHz, $CDCl_3$
210 200 190 180 170 160 150 140 130 120 110 100 90 80 70 60 50 40 30 20 10 0
f1 (ppm)

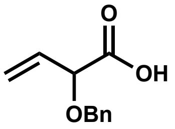

SI-8
^{1}H NMR, 600 MHz, $CDCl_3$

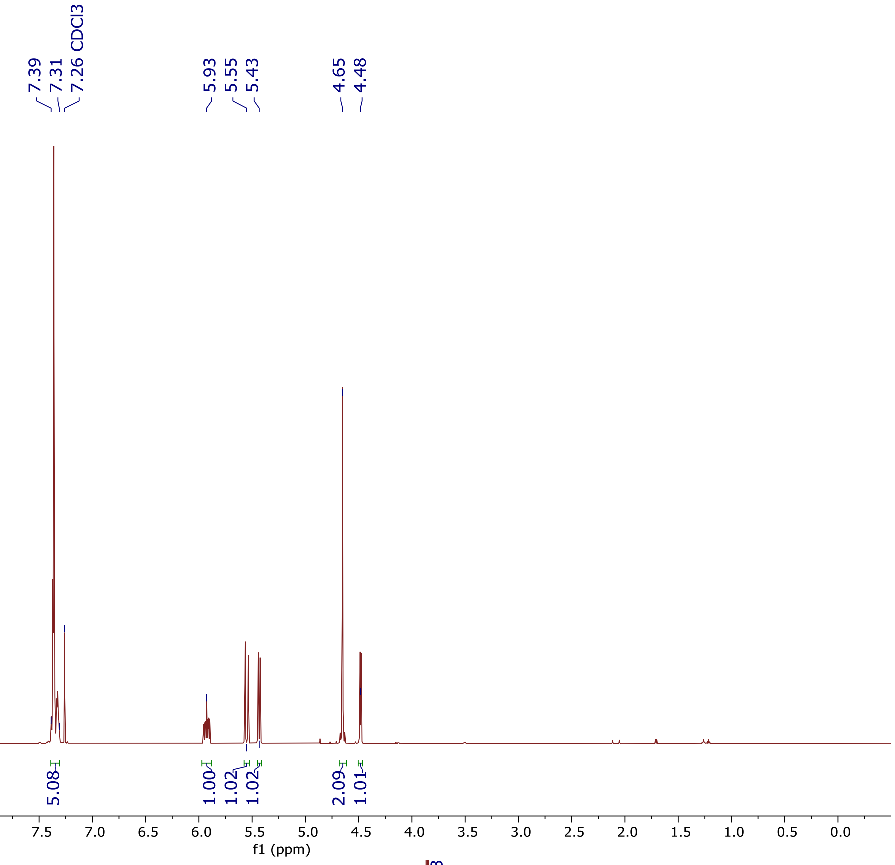

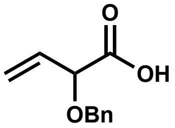

SI-8
^{13}C NMR, 151 MHz, $CDCl_3$

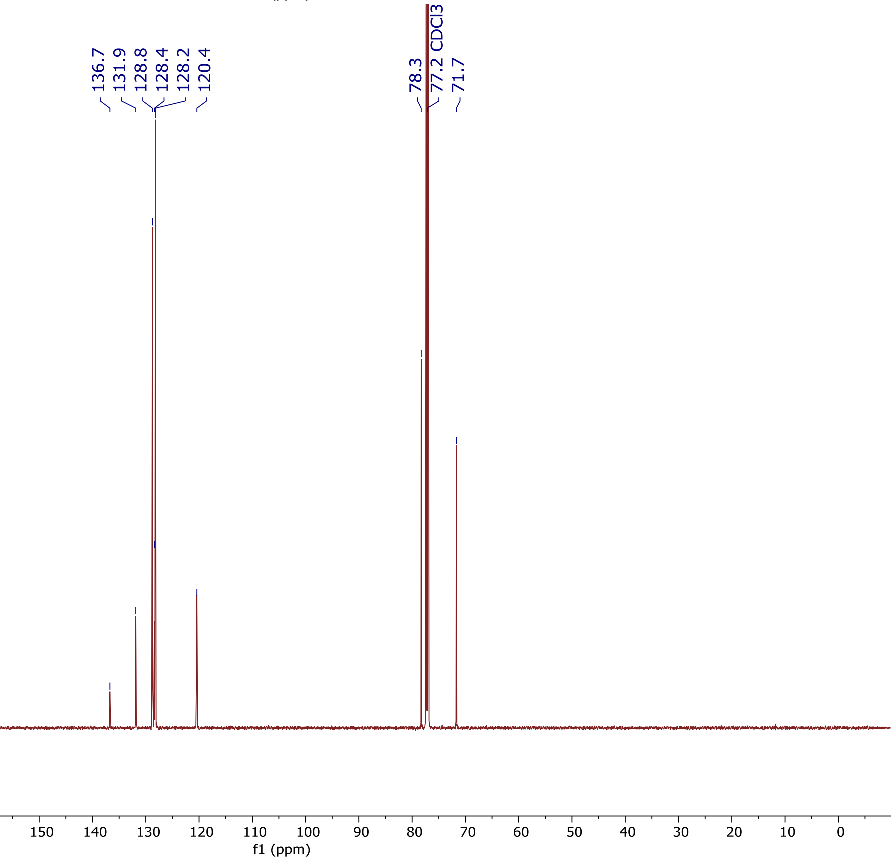

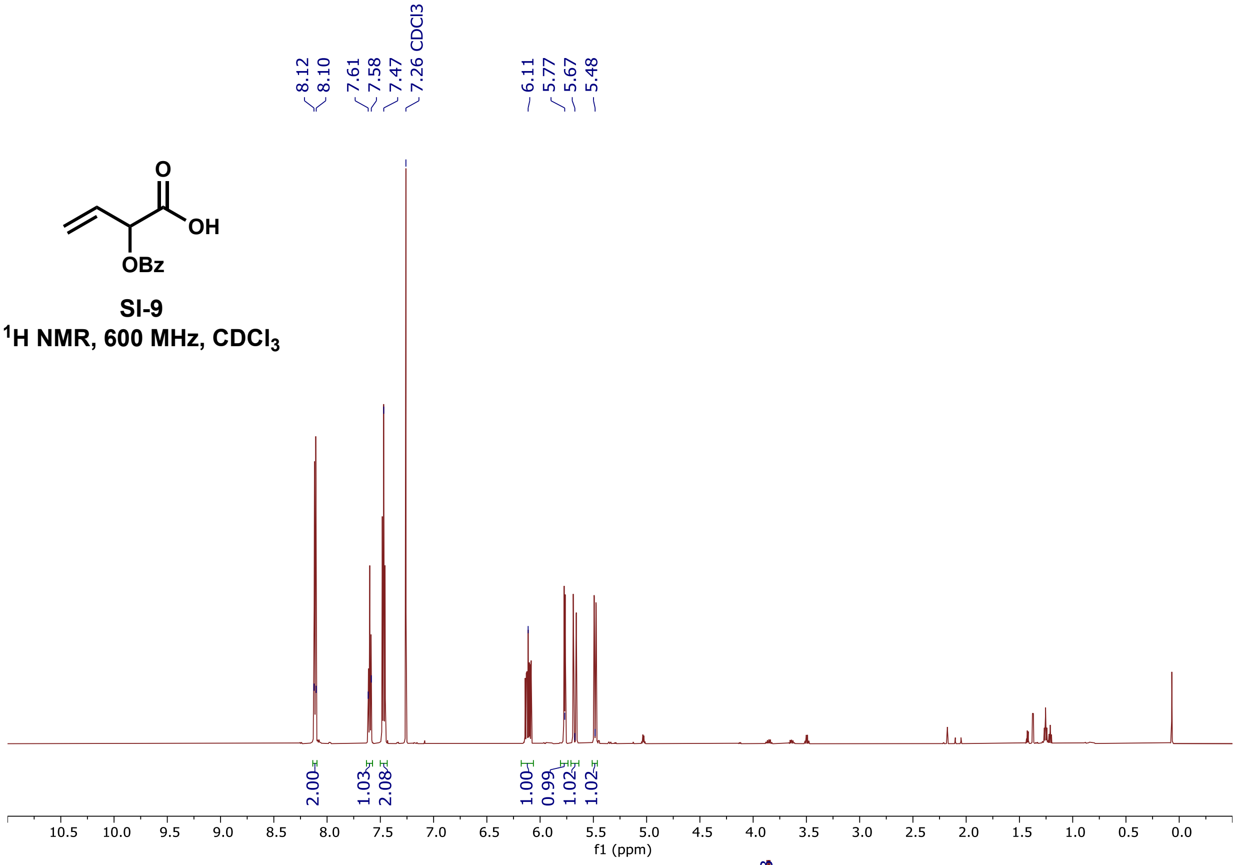
8.12
8.10
7.61
7.58
7.47
7.26 CDCl3
6.11
5.77
5.67
5.48
O
OH
OBz
SI-9
^{1}H NMR, 600 MHz, $CDCl_3$
2.00
1.03
2.08
1.00
0.99
1.02
1.02
10.5 10.0 9.5 9.0 8.5 8.0 7.5 7.0 6.5 6.0 5.5 5.0 4.5 4.0 3.5 3.0 2.5 2.0 1.5 1.0 0.5 0.0
f1 (ppm)

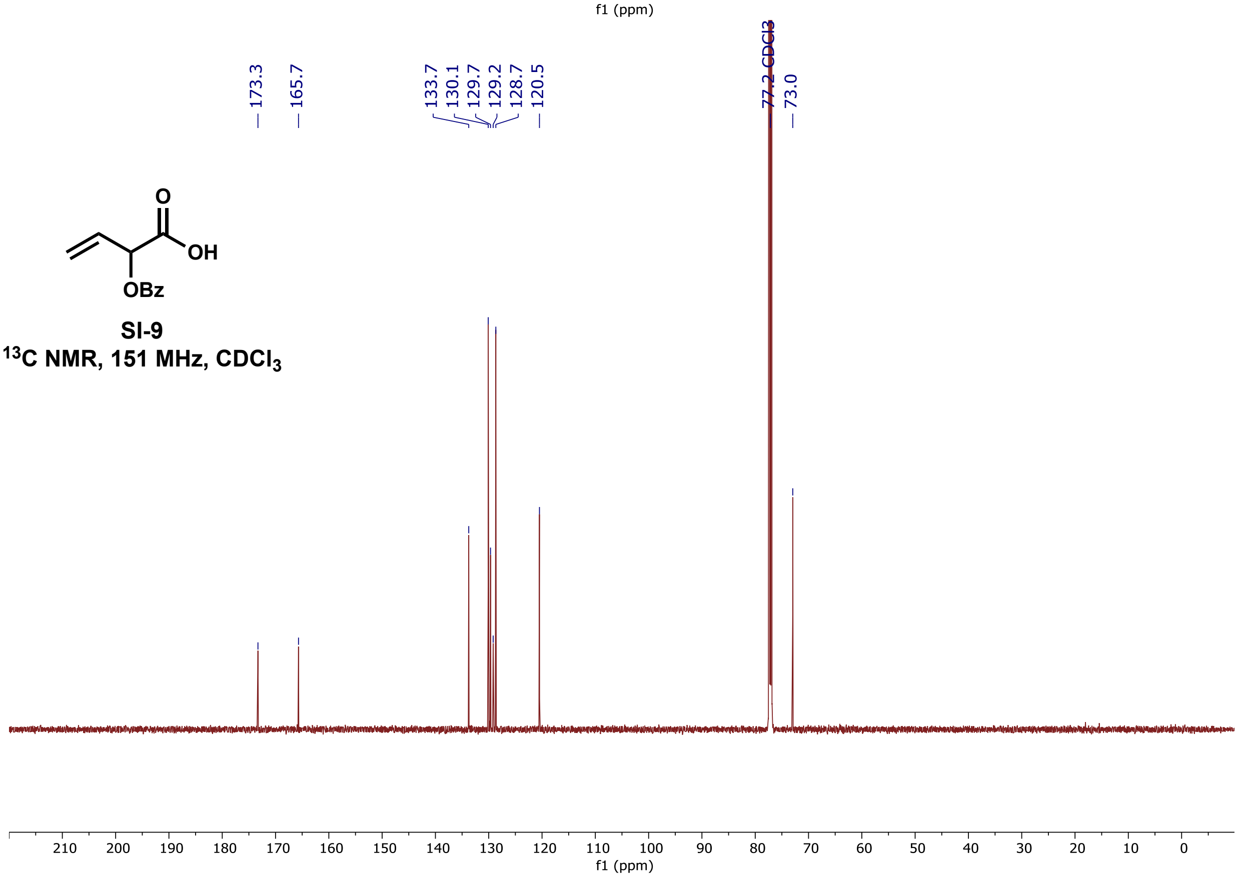
173.3
165.7
133.7
130.1
129.7
129.2
128.7
120.5
77.2 CDCl3
73.0
O
OH
OBz
SI-9
^{13}C NMR, 151 MHz, $CDCl_3$
210 200 190 180 170 160 150 140 130 120 110 100 90 80 70 60 50 40 30 20 10 0
f1 (ppm)

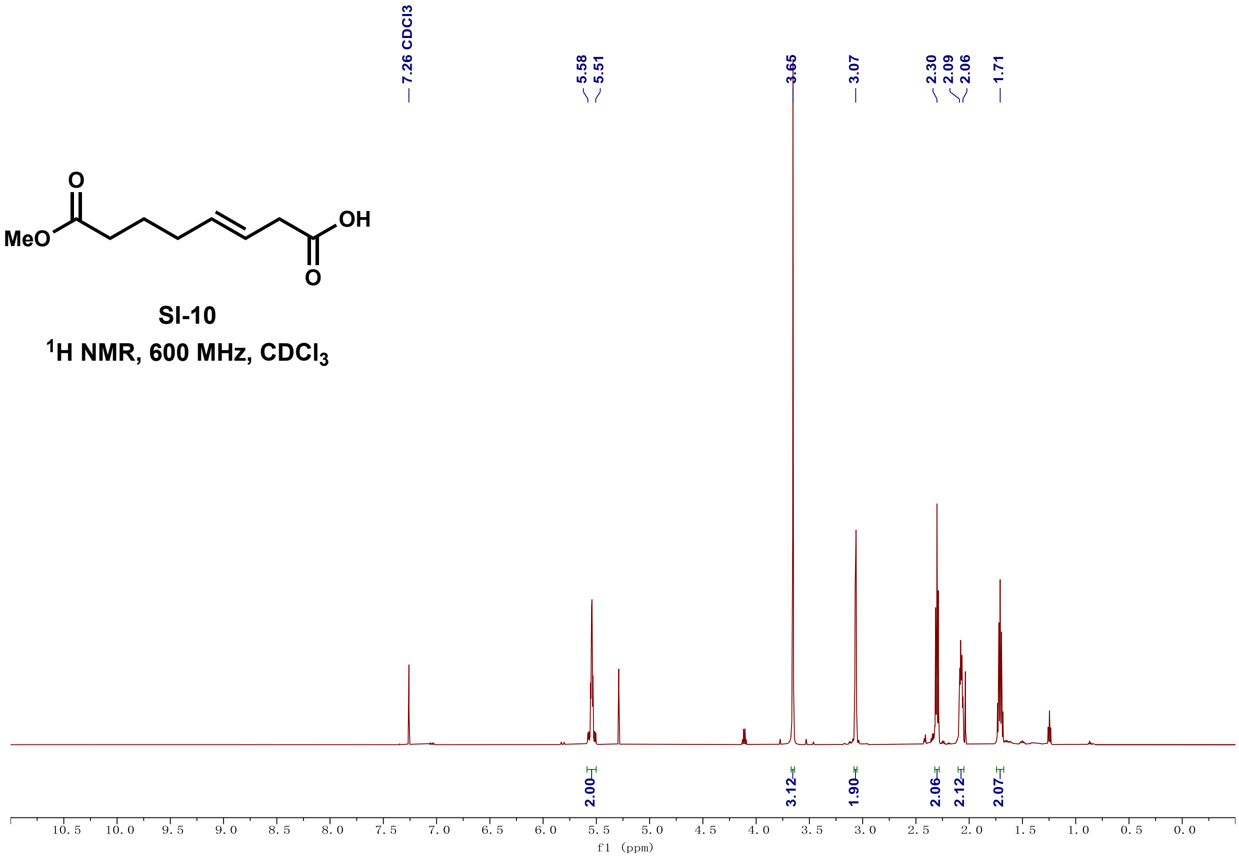

SI-10

^{1}H NMR, 600 MHz, $CDCl_3$

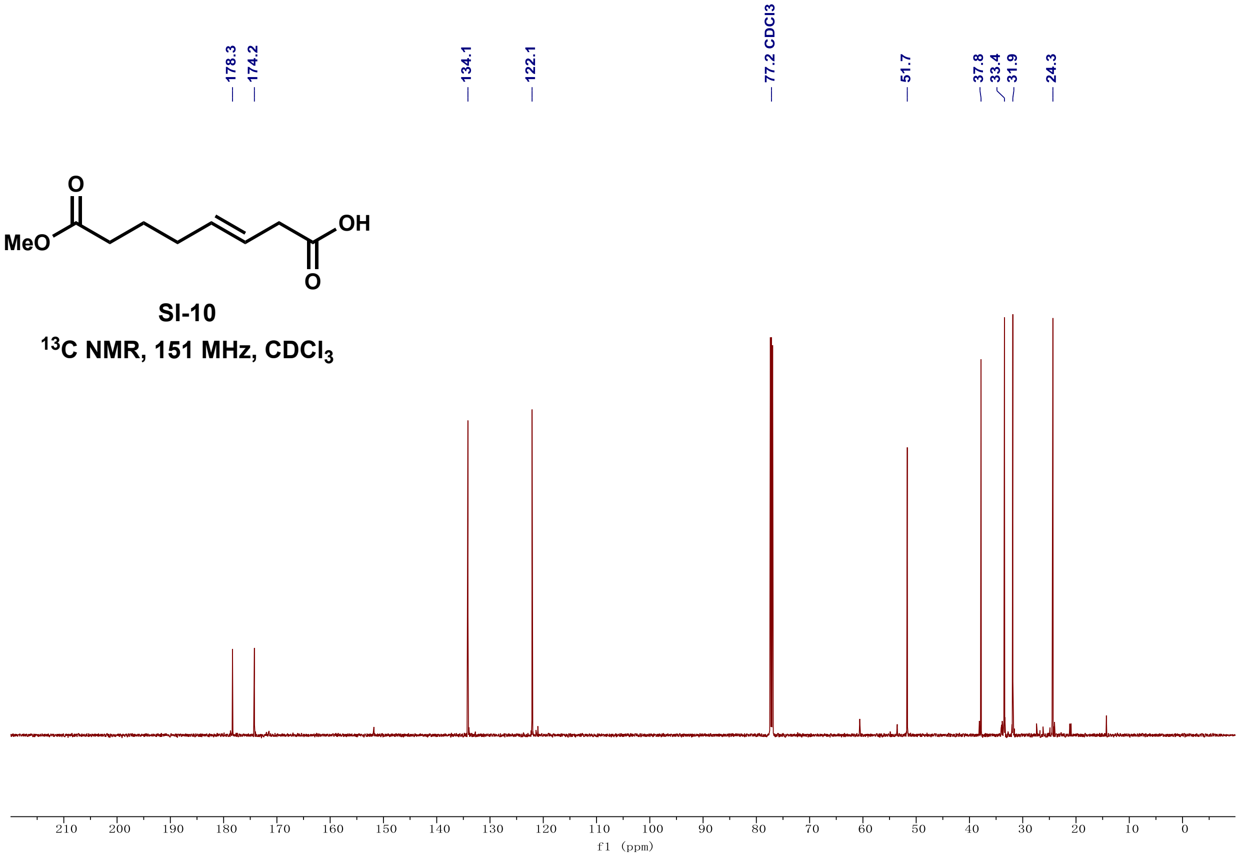

SI-10

^{13}C NMR, 151 MHz, $CDCl_3$

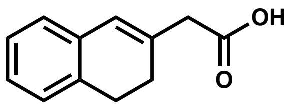

1d

^{1}H NMR, 600 MHz, $CDCl_3$

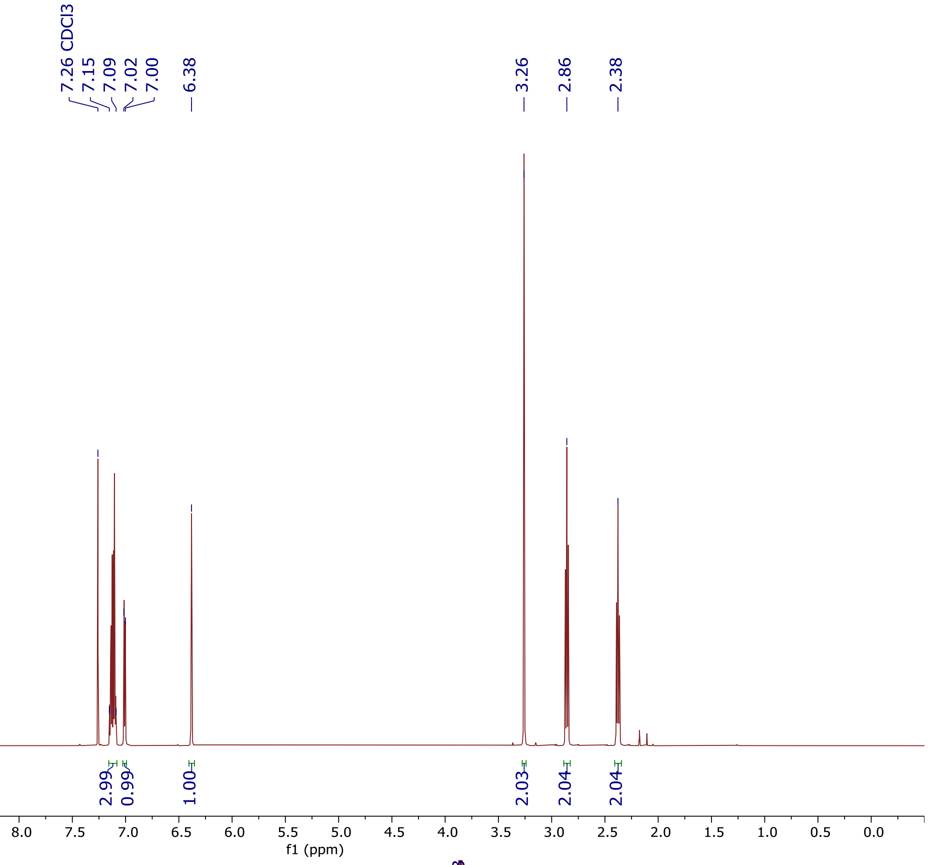

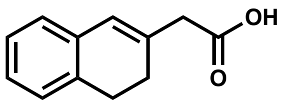

1d

^{13}C NMR, 151 MHz, $CDCl_3$

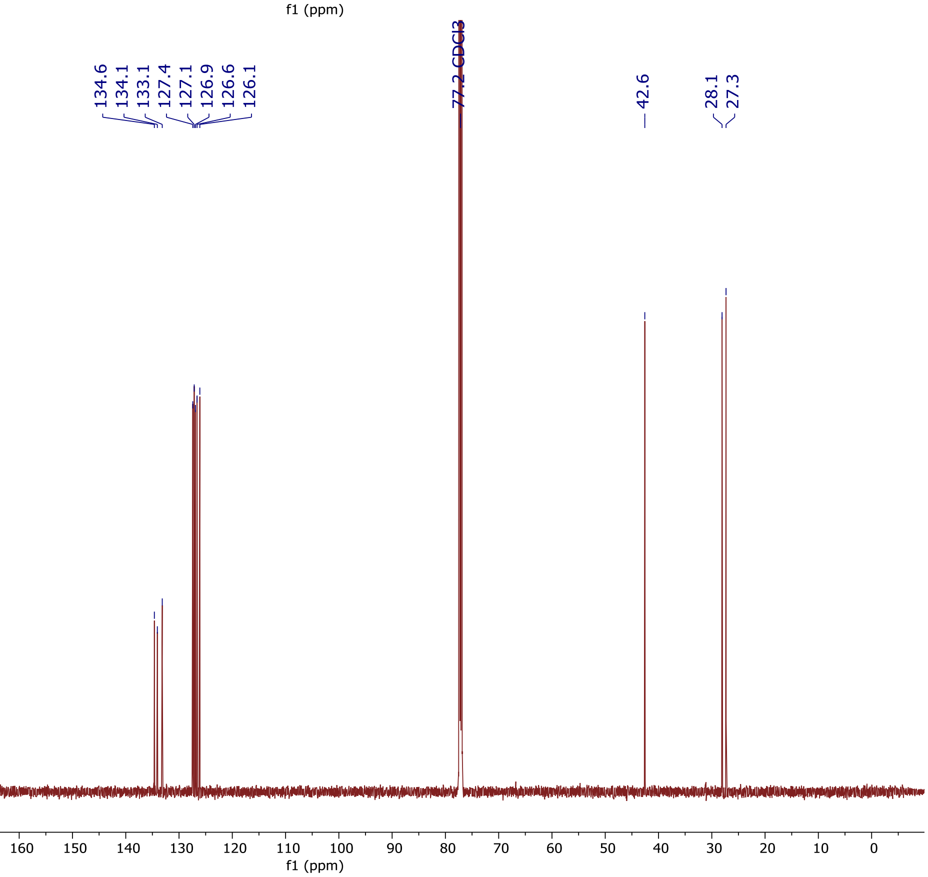

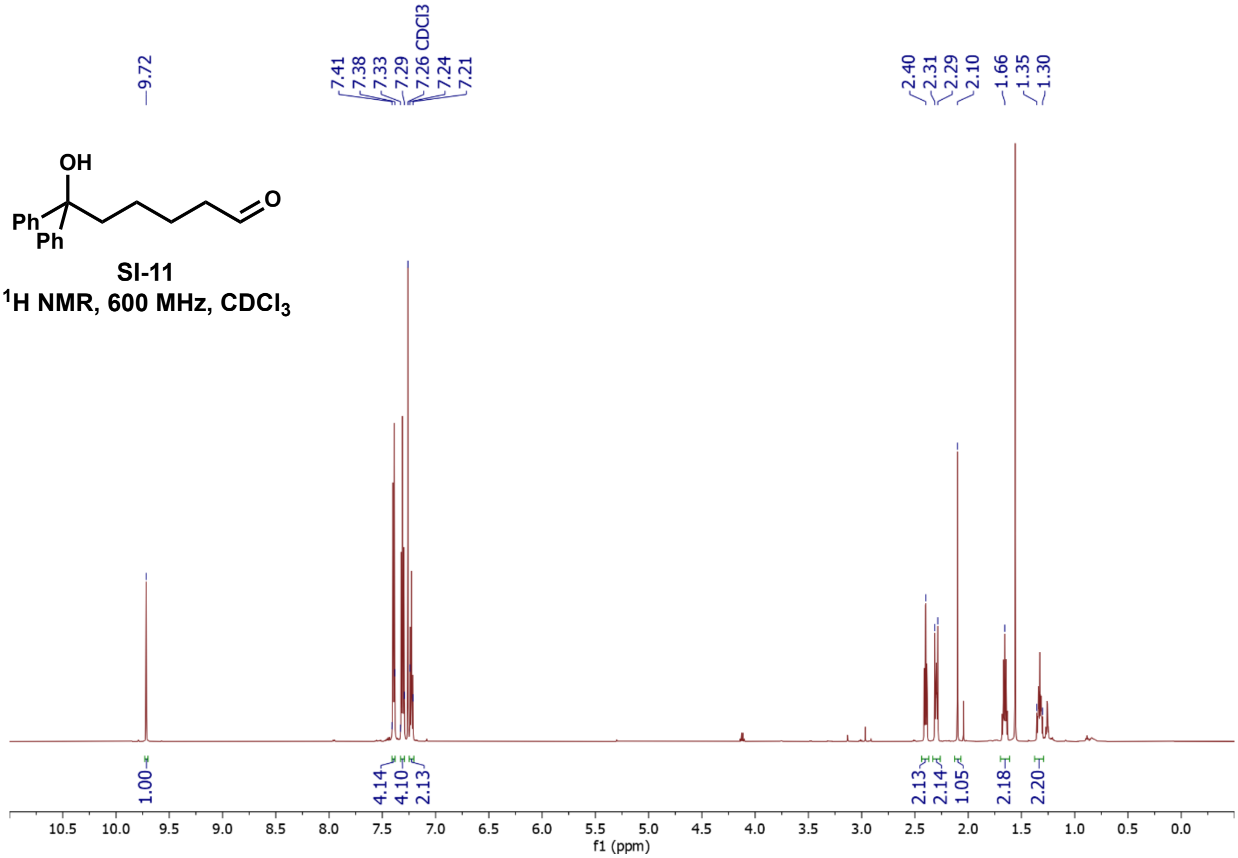
OH
Ph
Ph
O
SI-11
1H NMR, 600 MHz, CDCl3
9.72
7.41
7.38
7.33
7.29
7.26 CDCl3
7.24
7.21
2.40
2.31
2.29
2.10
1.66
1.35
1.30
1.00
4.14
4.10
2.13
2.13
2.14
1.05
2.18
2.20
f1 (ppm)

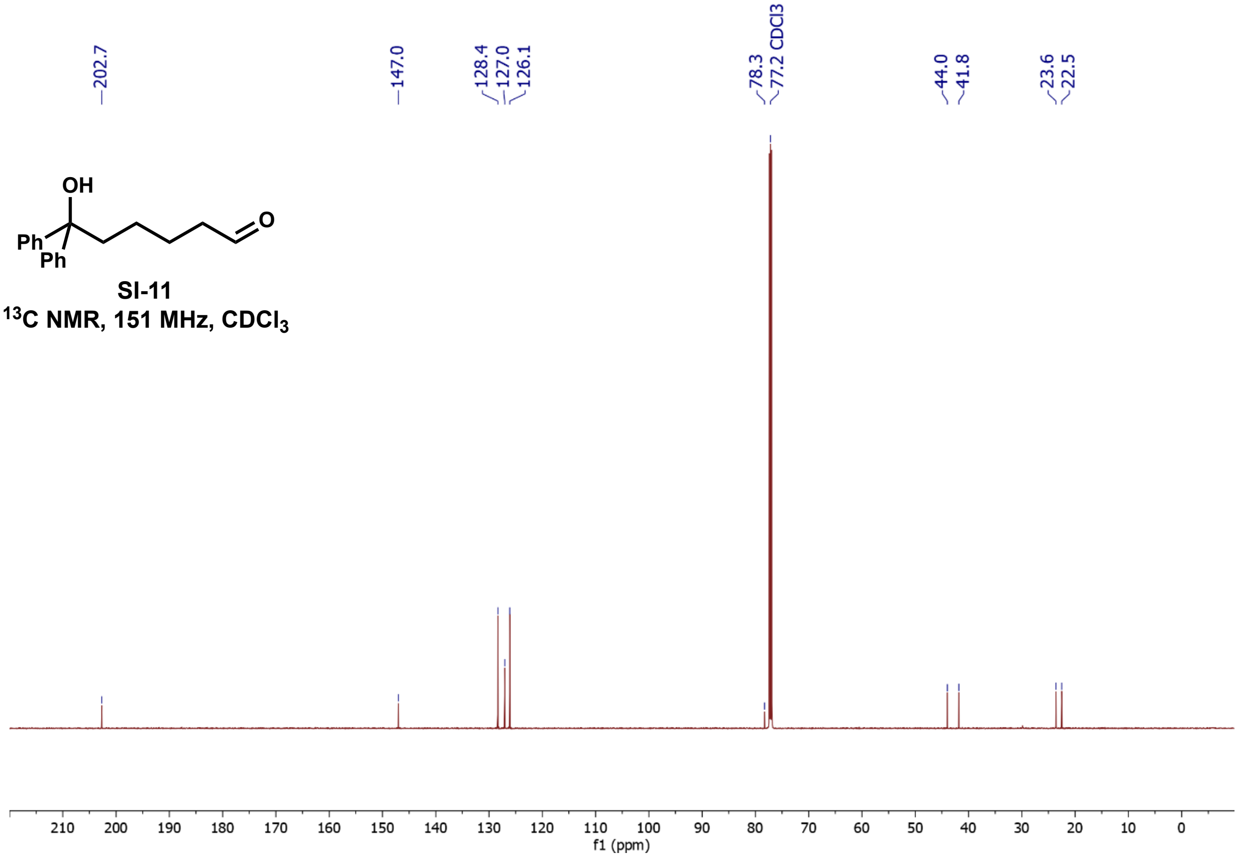
OH
Ph
Ph
O
SI-11
13C NMR, 151 MHz, CDCl3
202.7
147.0
128.4
127.0
126.1
78.3
77.2 CDCl3
44.0
41.8
23.6
22.5
f1 (ppm)

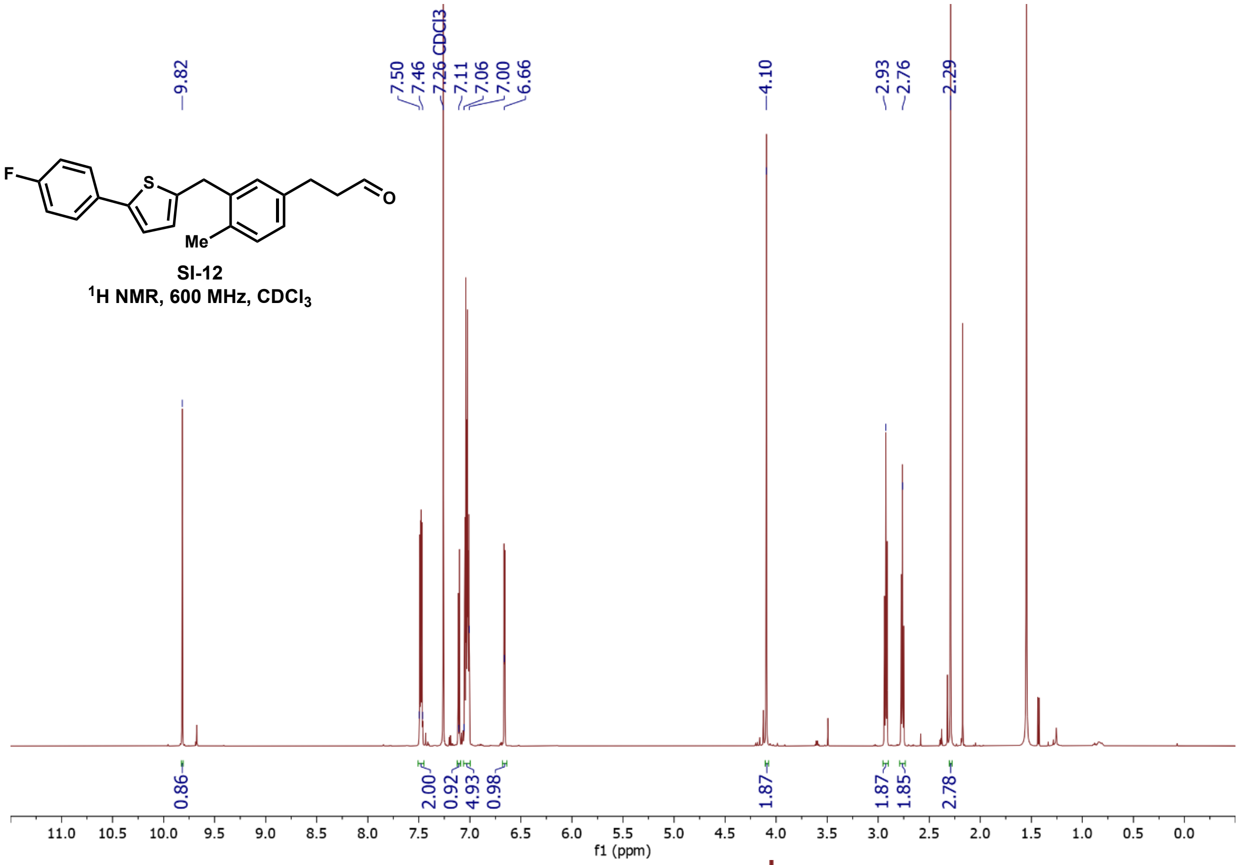

SI-12
^{1}H NMR, 600 MHz, CDCl$_3$
9.82
7.50
7.46
7.26 CDCl3
7.11
7.06
7.00
6.66
4.10
2.93
2.76
2.29
0.86
2.00
0.92
4.93
0.98
1.87
1.87
1.85
2.78
f1 (ppm)

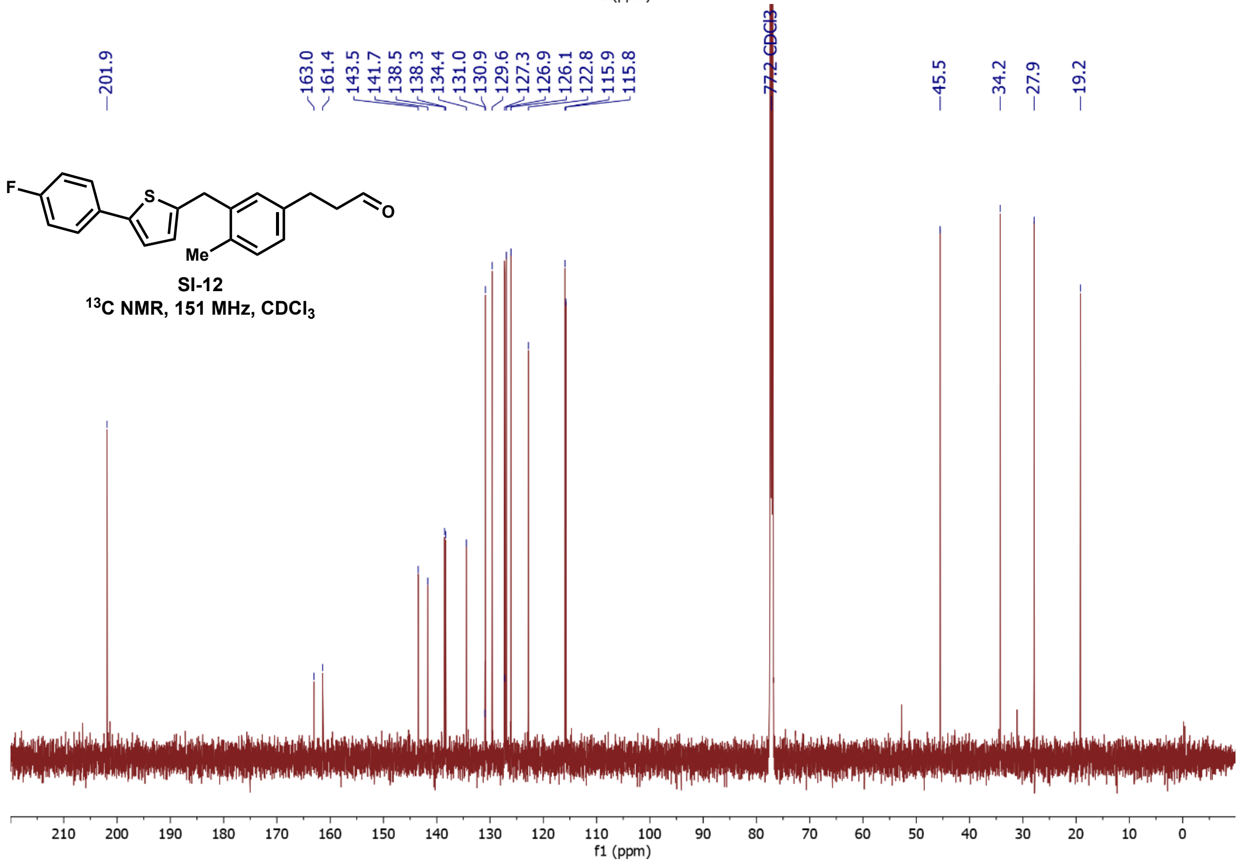

SI-12
^{13}C NMR, 151 MHz, CDCl$_3$
201.9
163.0
161.4
143.5
141.7
138.5
138.3
134.4
131.0
130.9
129.6
127.3
126.9
126.1
122.8
115.9
115.8
77.2 CDCl3
45.5
34.2
27.9
19.2
f1 (ppm)

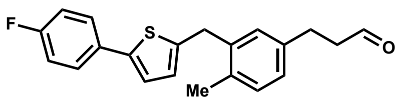

SI-12
^{19}F NMR, 470 MHz, $CDCl_3$

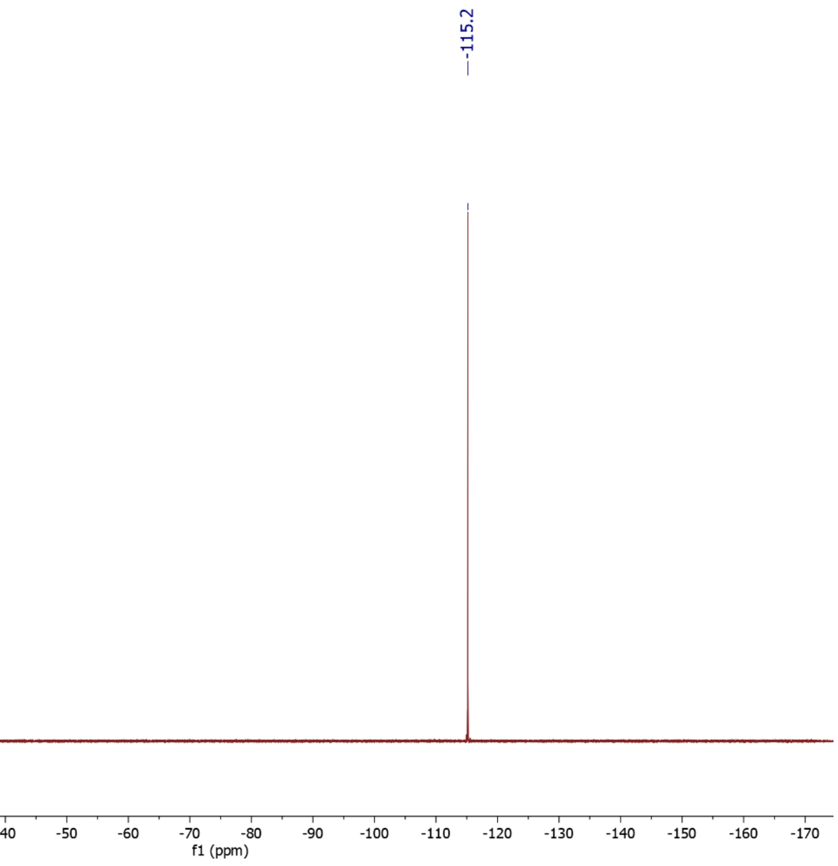

7.2.2. NMR Spectra of Products

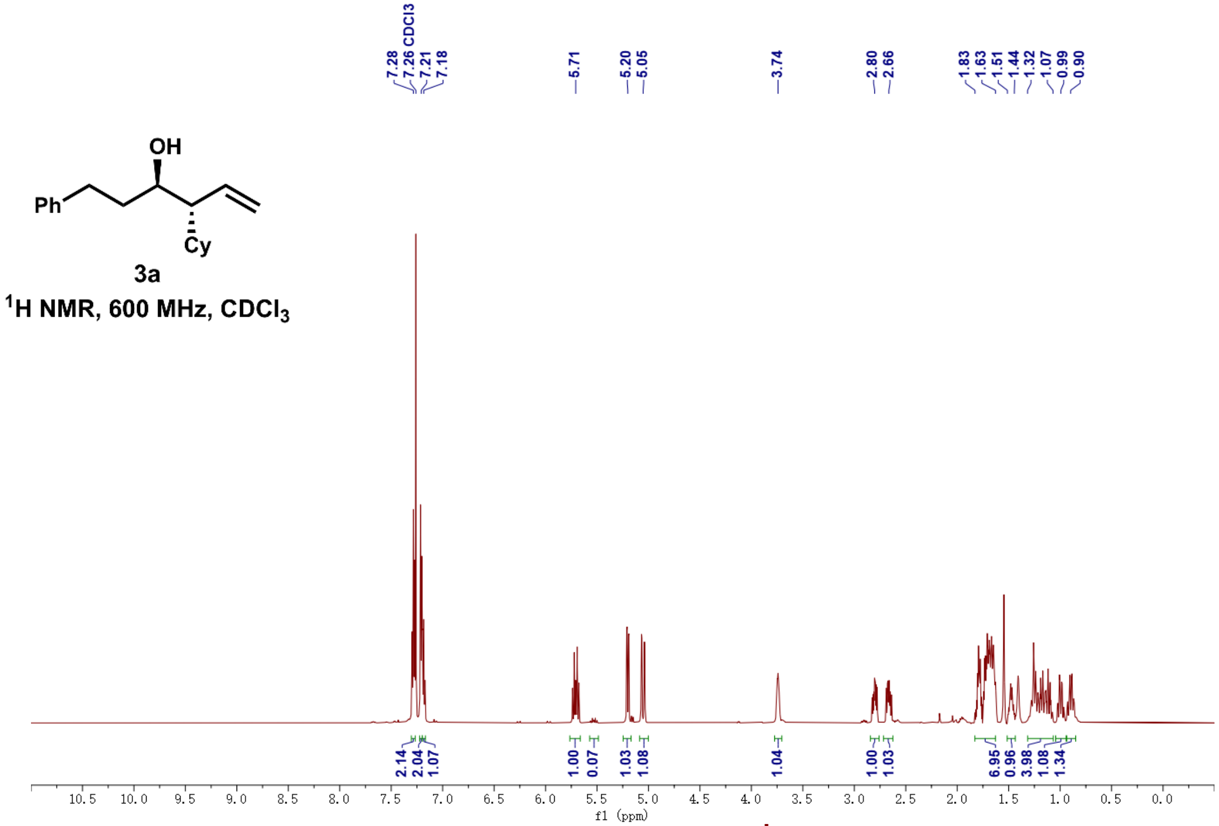

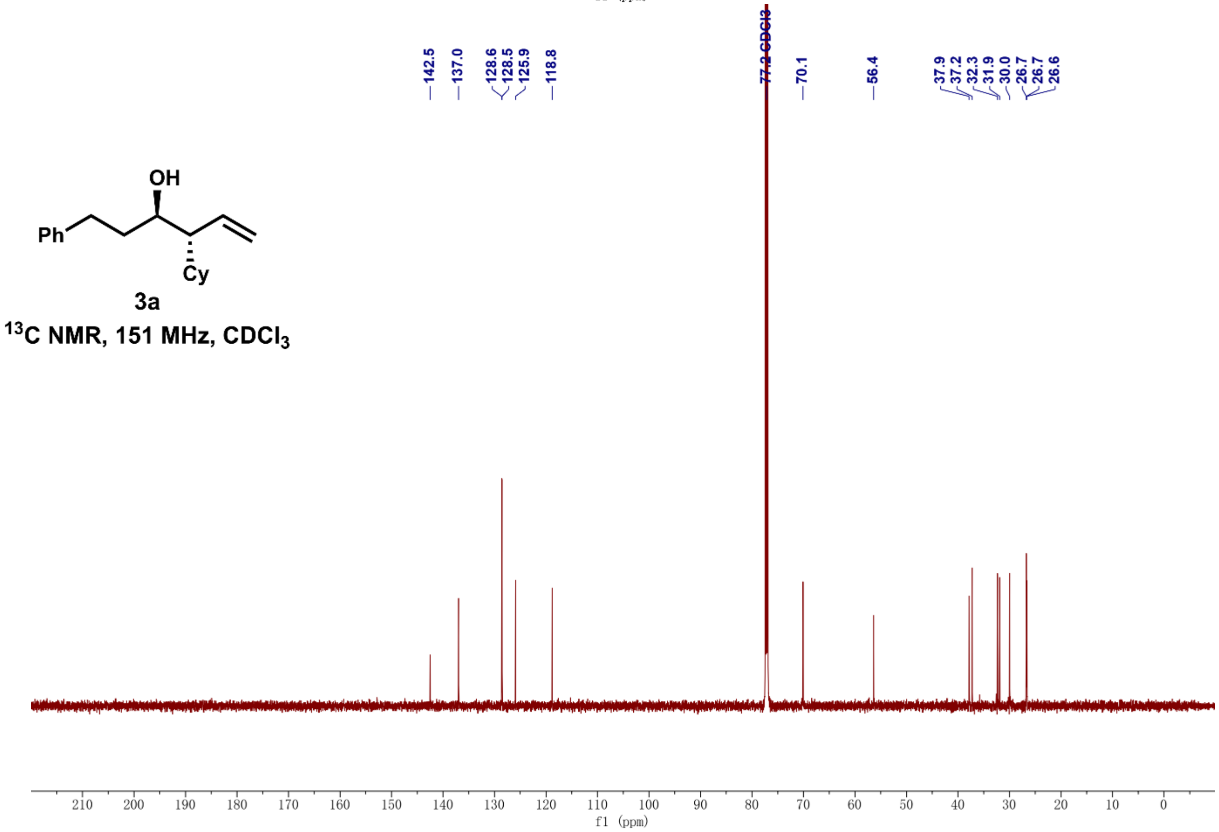

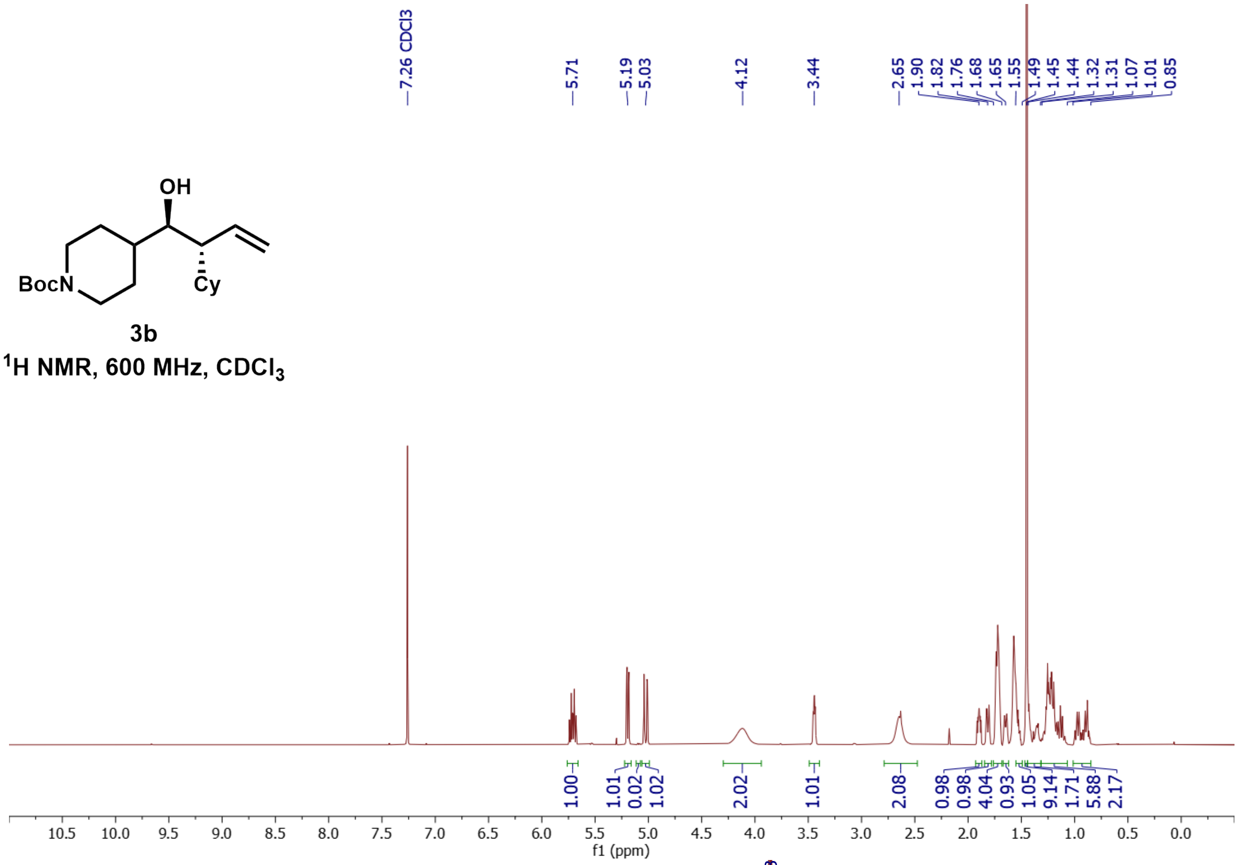

3b

^{1}H NMR, 600 MHz, $CDCl_3$

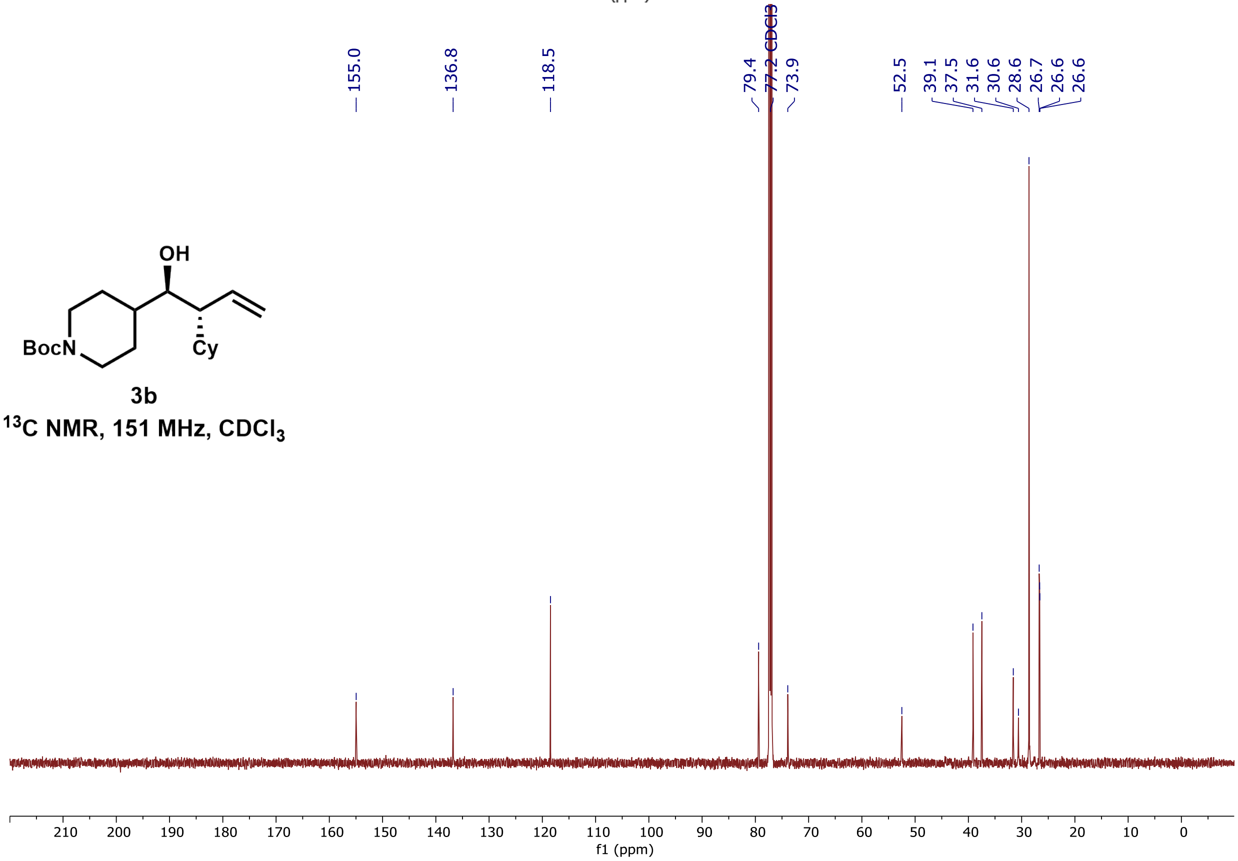

3b

^{13}C NMR, 151 MHz, $CDCl_3$

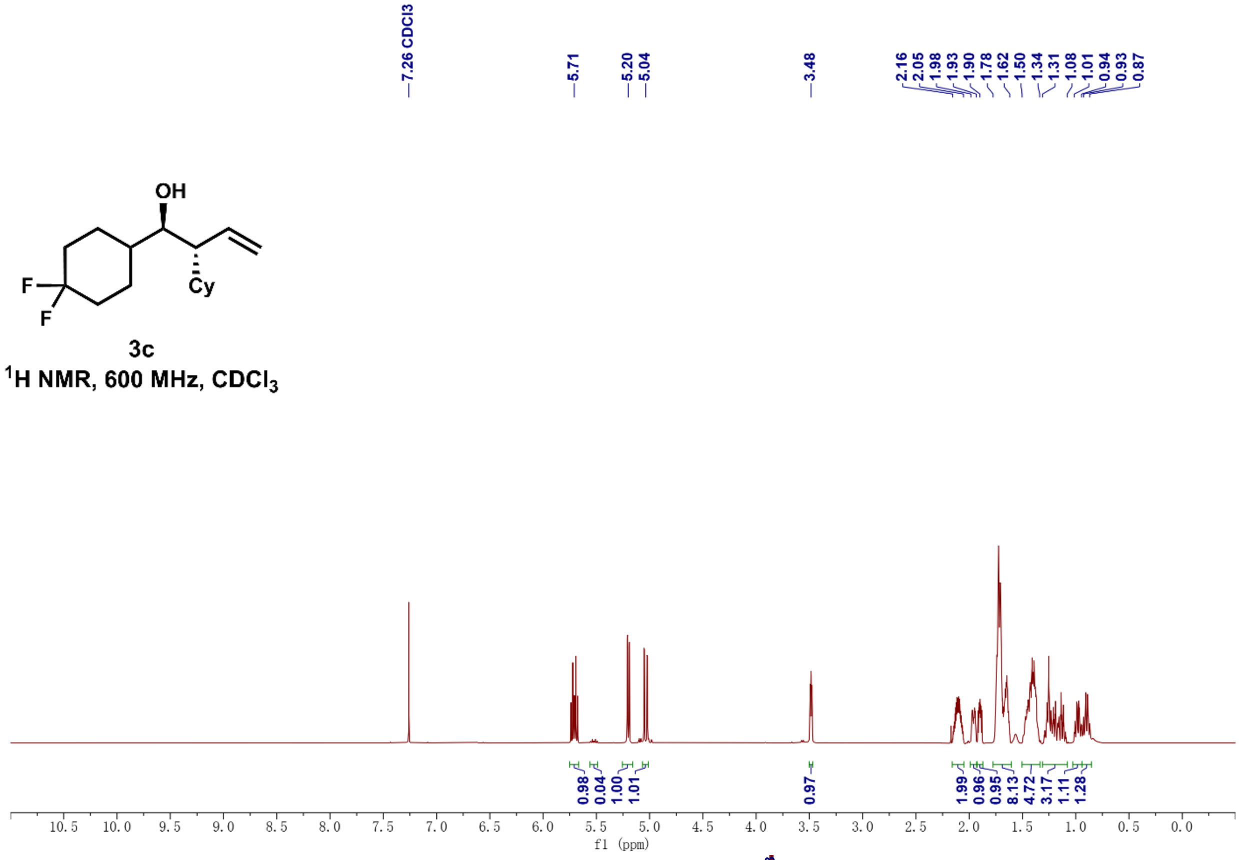
7.26 CDCl3
5.71
5.20
5.04
3.48
2.16
2.05
1.98
1.93
1.90
1.78
1.62
1.50
1.34
1.31
1.08
1.01
0.94
0.93
0.87
OH
F
F
Cy
3c
^{1}H NMR, 600 MHz, $CDCl_3$
0.98
0.04
1.00
1.01
0.97
1.99
0.96
0.95
8.13
4.72
3.17
1.11
1.28
f1 (ppm)

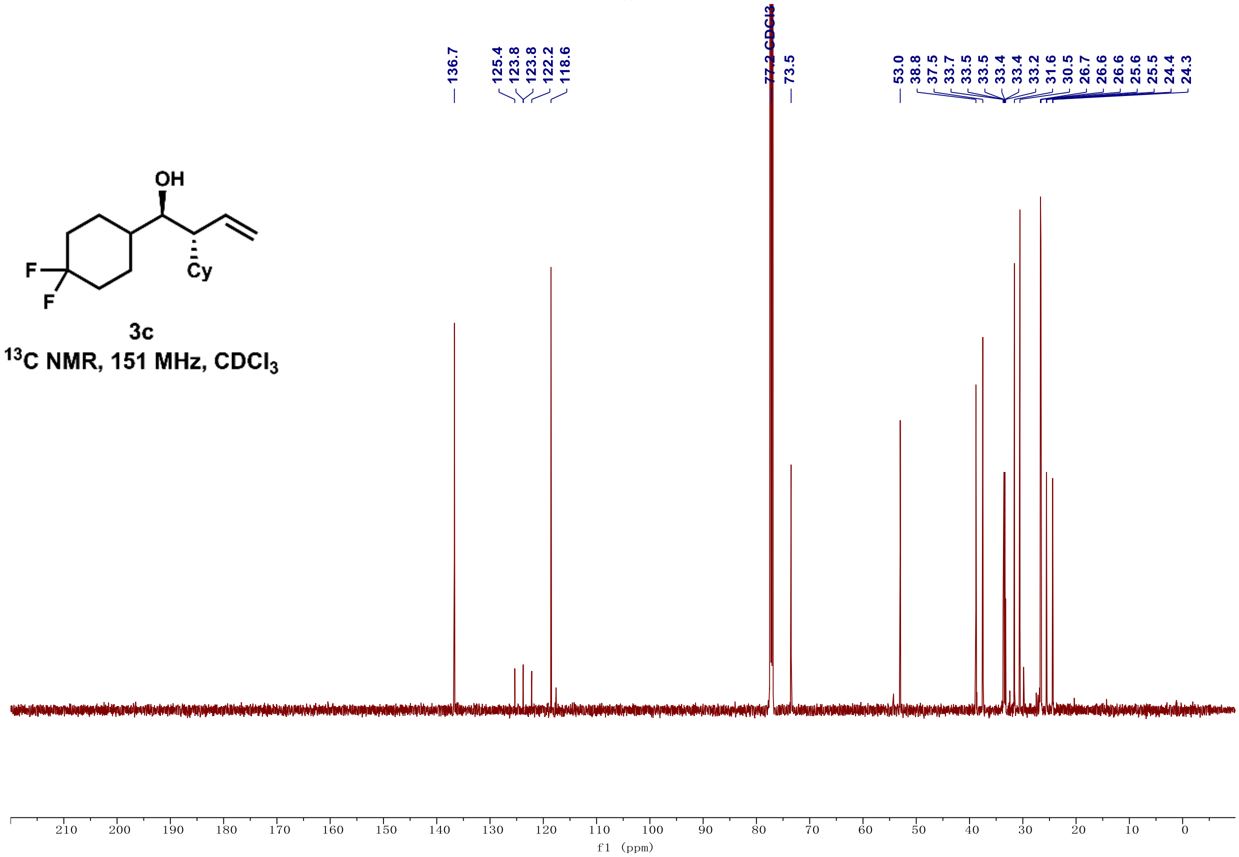
136.7
125.4
123.8
123.8
122.2
118.6
77.2 CDCl3
73.5
53.0
38.8
37.5
33.7
33.5
33.5
33.4
33.4
33.2
31.6
30.5
26.7
26.6
26.6
25.6
25.5
24.4
24.3
OH
F
F
Cy
3c
^{13}C NMR, 151 MHz, $CDCl_3$
f1 (ppm)

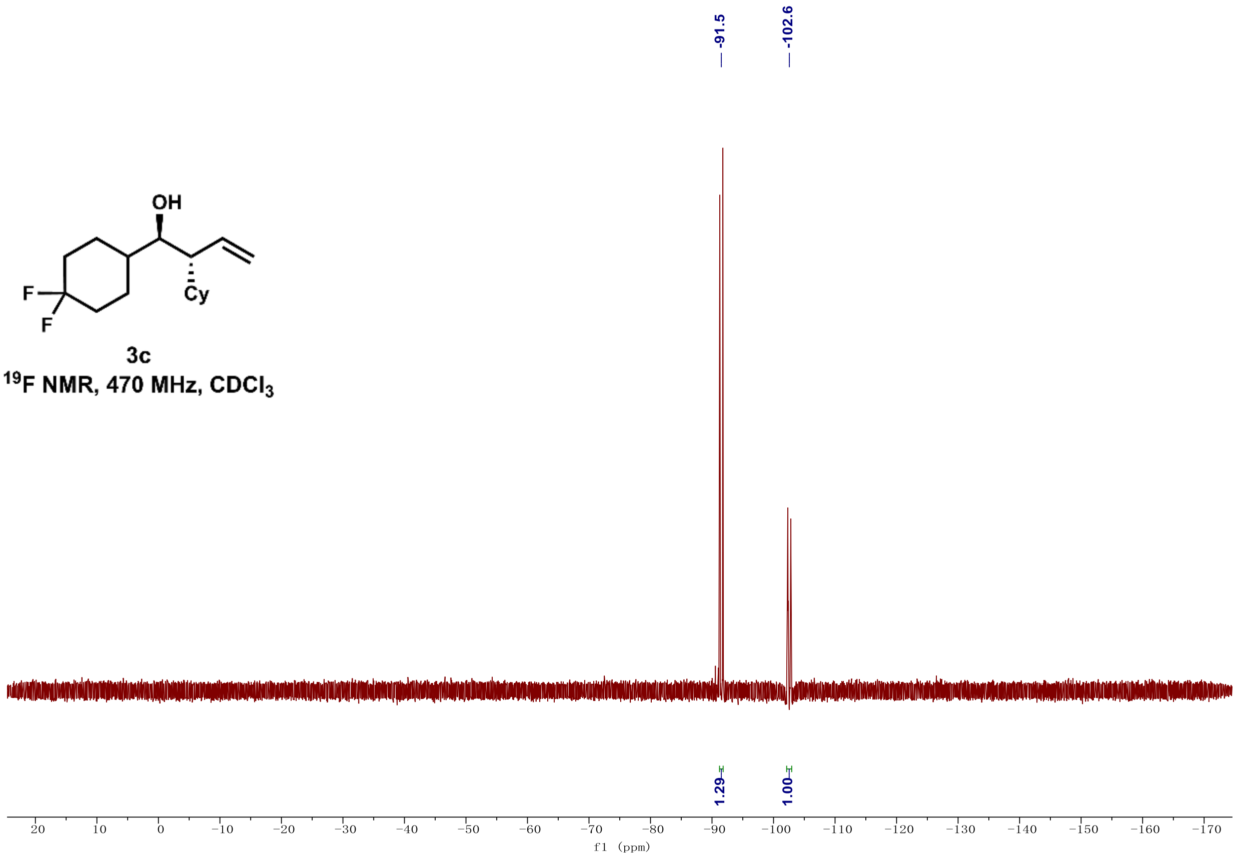

3c

^{19}F NMR, 470 MHz, $CDCl_3$

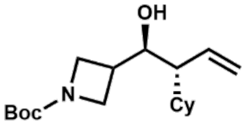

3d

1H NMR, 600 MHz, CDCl3

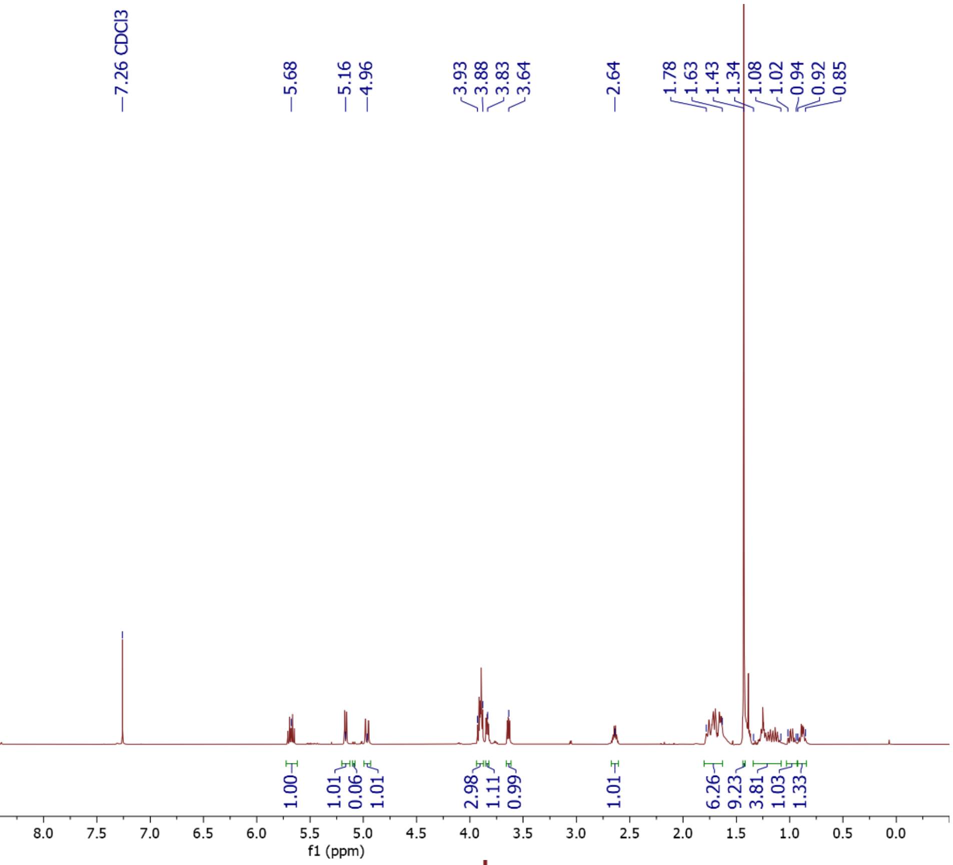

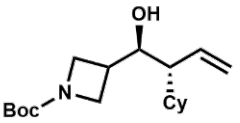

3d

13C NMR, 151 MHz, CDCl3

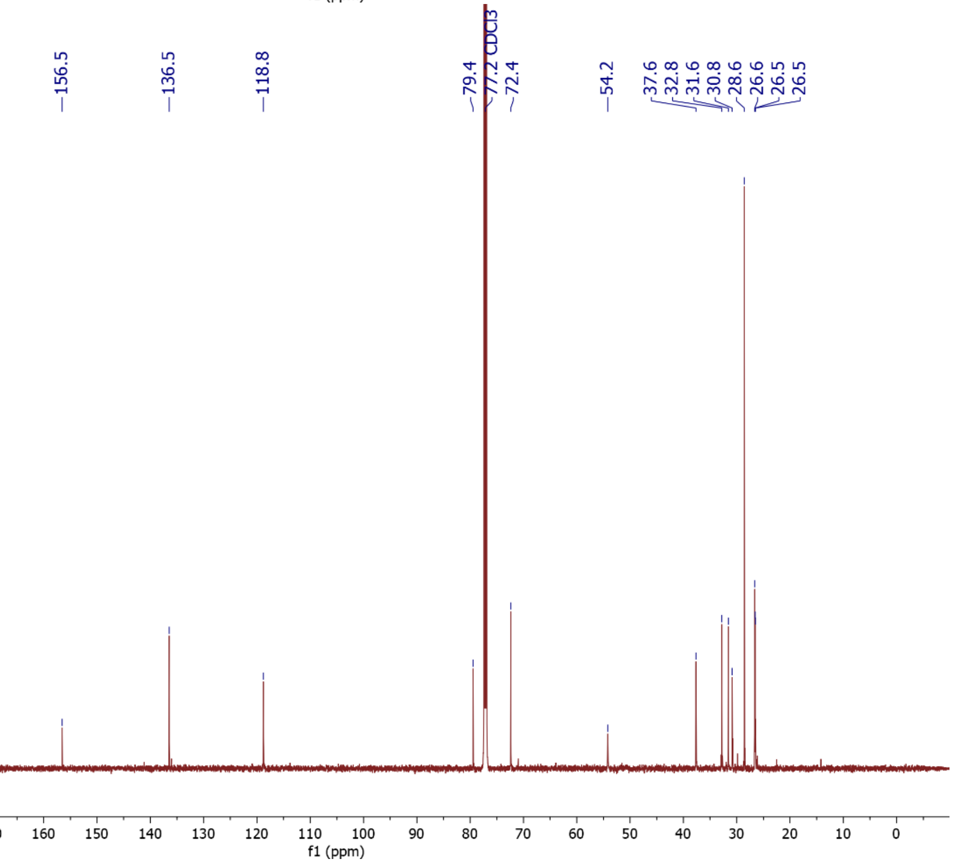

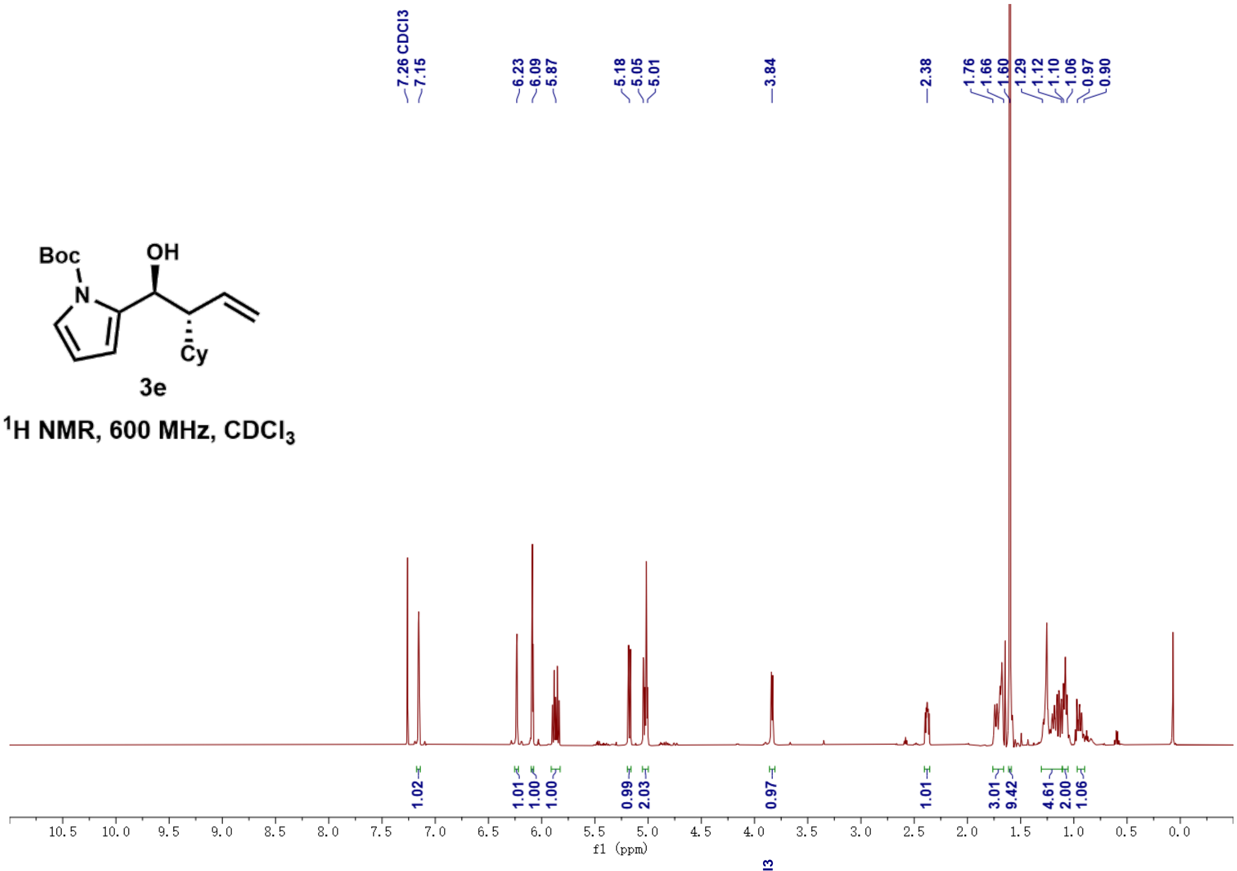
7.26 CDCl3
7.15
6.23
6.09
5.87
5.18
5.05
5.01
3.84
2.38
1.76
1.66
1.60
1.29
1.12
1.10
1.06
0.97
0.90
Boc
OH
N
Cy
3e
^{1}H NMR, 600 MHz, $CDCl_3$
1.02
1.01
1.00
1.00
0.99
2.03
0.97
1.01
3.01
9.42
4.61
2.00
1.06
f1 (ppm)

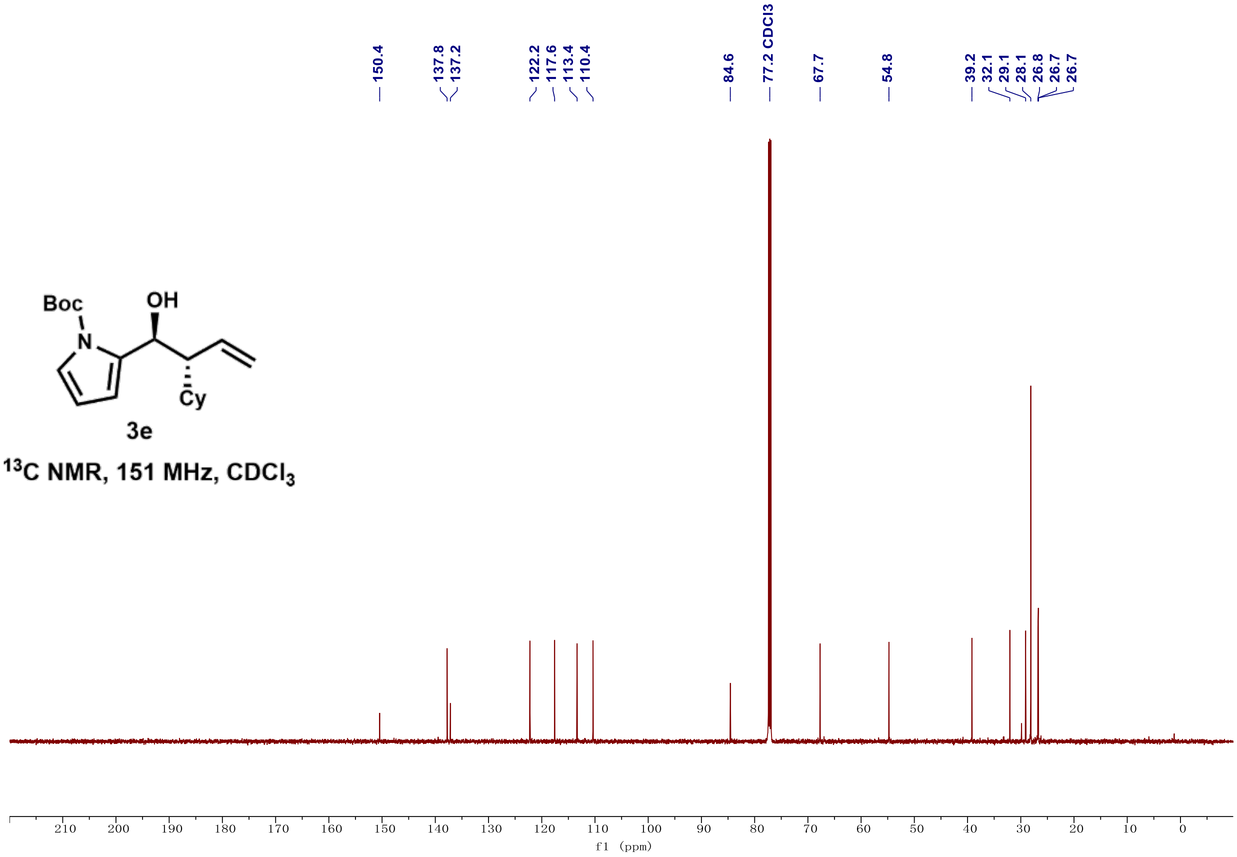
150.4
137.8
137.2
122.2
117.6
113.4
110.4
84.6
77.2 CDCl3
67.7
54.8
39.2
32.1
29.1
28.1
26.8
26.7
26.7
Boc
OH
N
Cy
3e
^{13}C NMR, 151 MHz, $CDCl_3$
f1 (ppm)

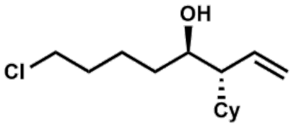

3f

^{1}H NMR, 600 MHz, $CDCl_3$

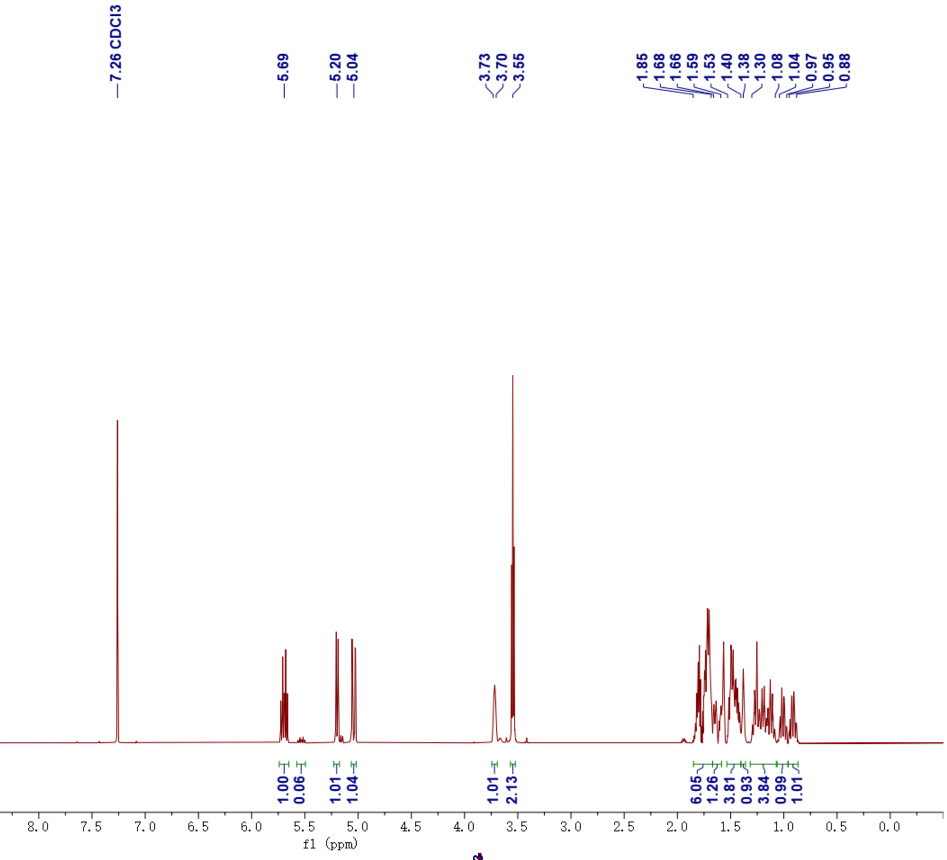

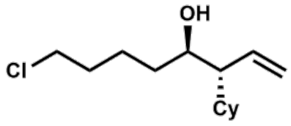

3f

^{13}C NMR, 151 MHz, $CDCl_3$

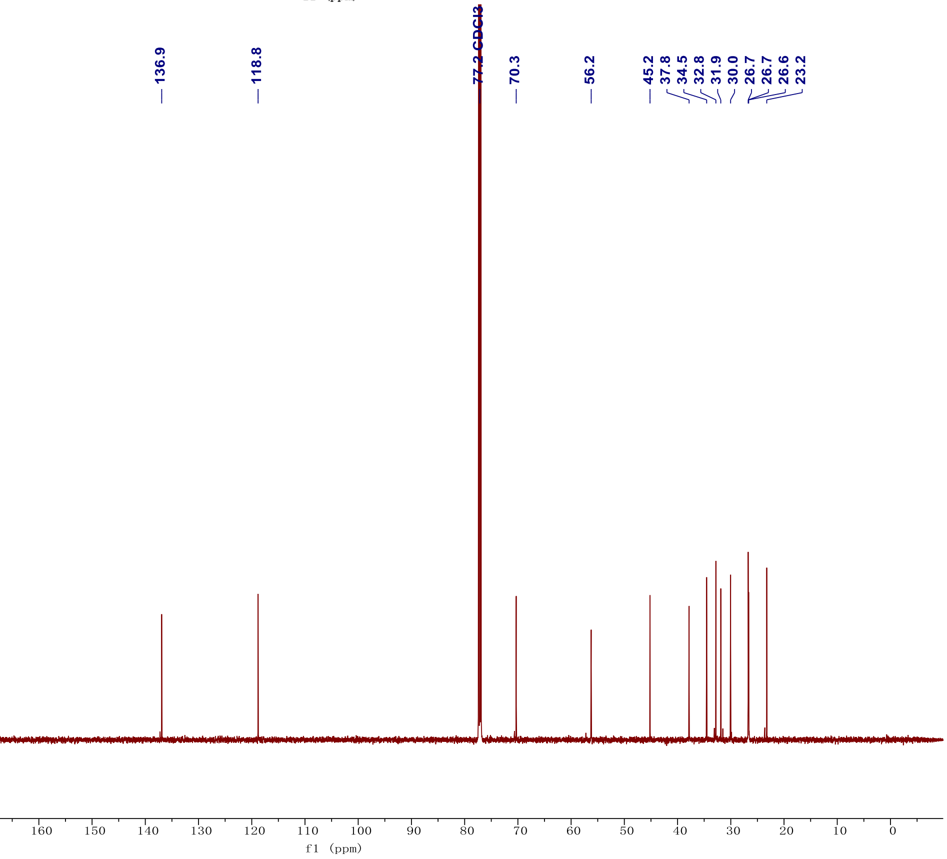

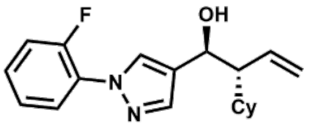

3g

^{1}H NMR, 600 MHz, $CDCl_3$

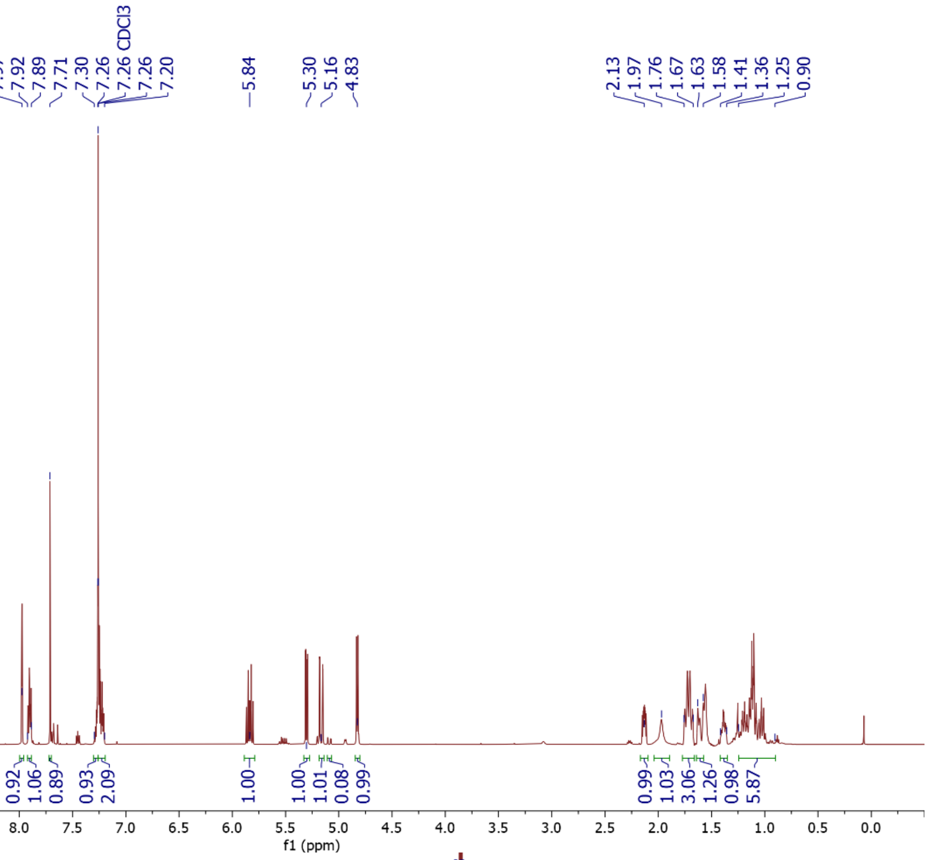

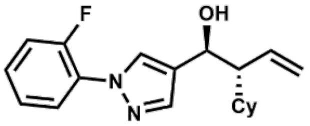

3g

^{13}C NMR, 151 MHz, $CDCl_3$

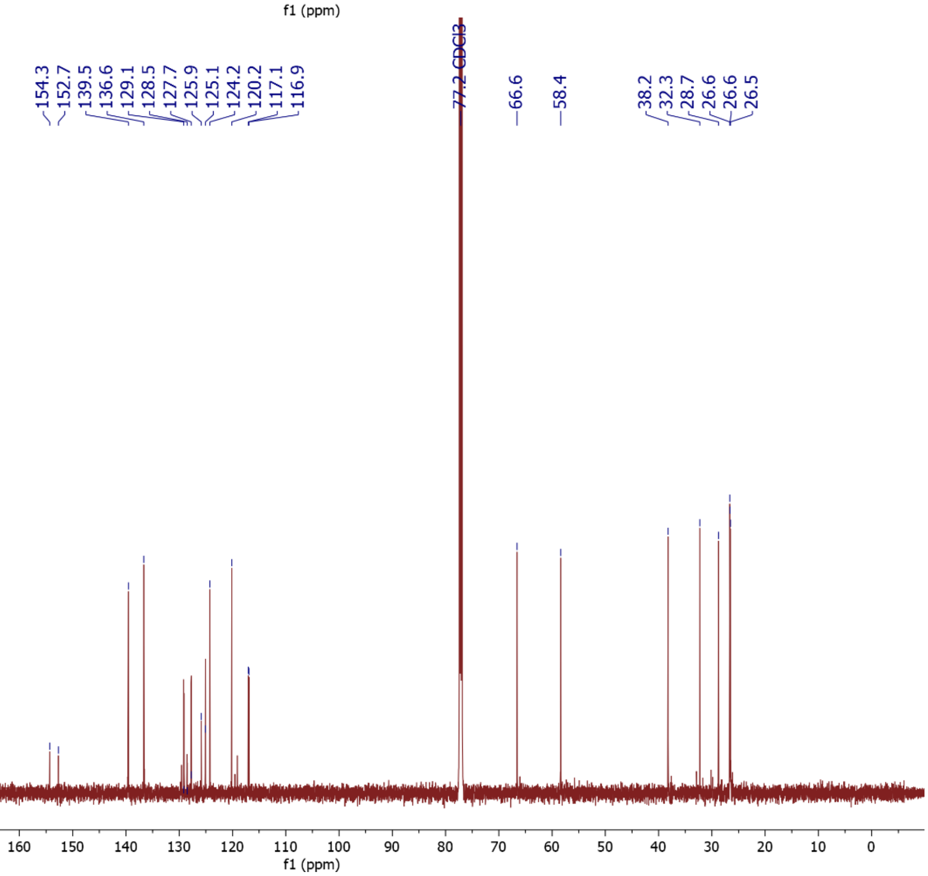

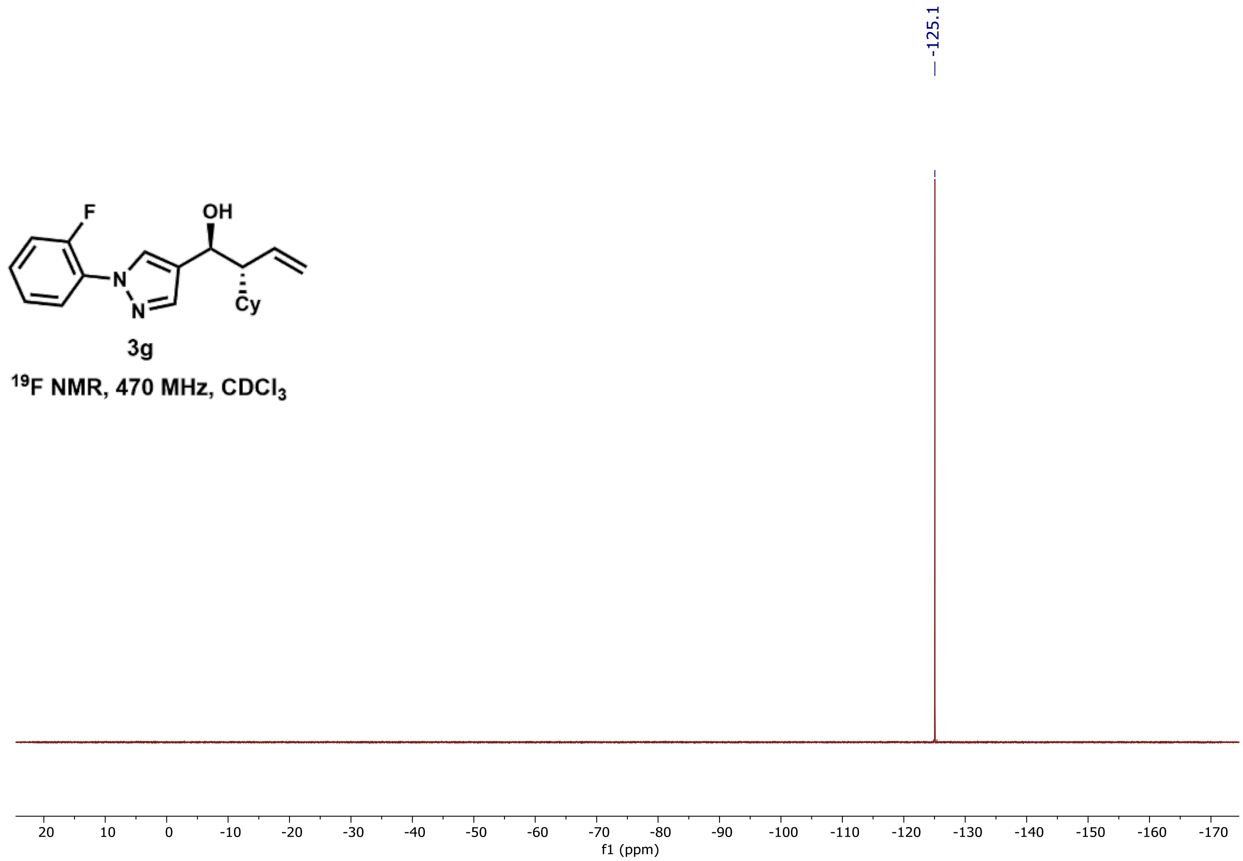

F
OH
N
N
Cy
3g
^{19}F NMR, 470 MHz, CDCl$_3$
-125.1
20 10 0 -10 -20 -30 -40 -50 -60 -70 -80 -90 -100 -110 -120 -130 -140 -150 -160 -170
f1 (ppm)

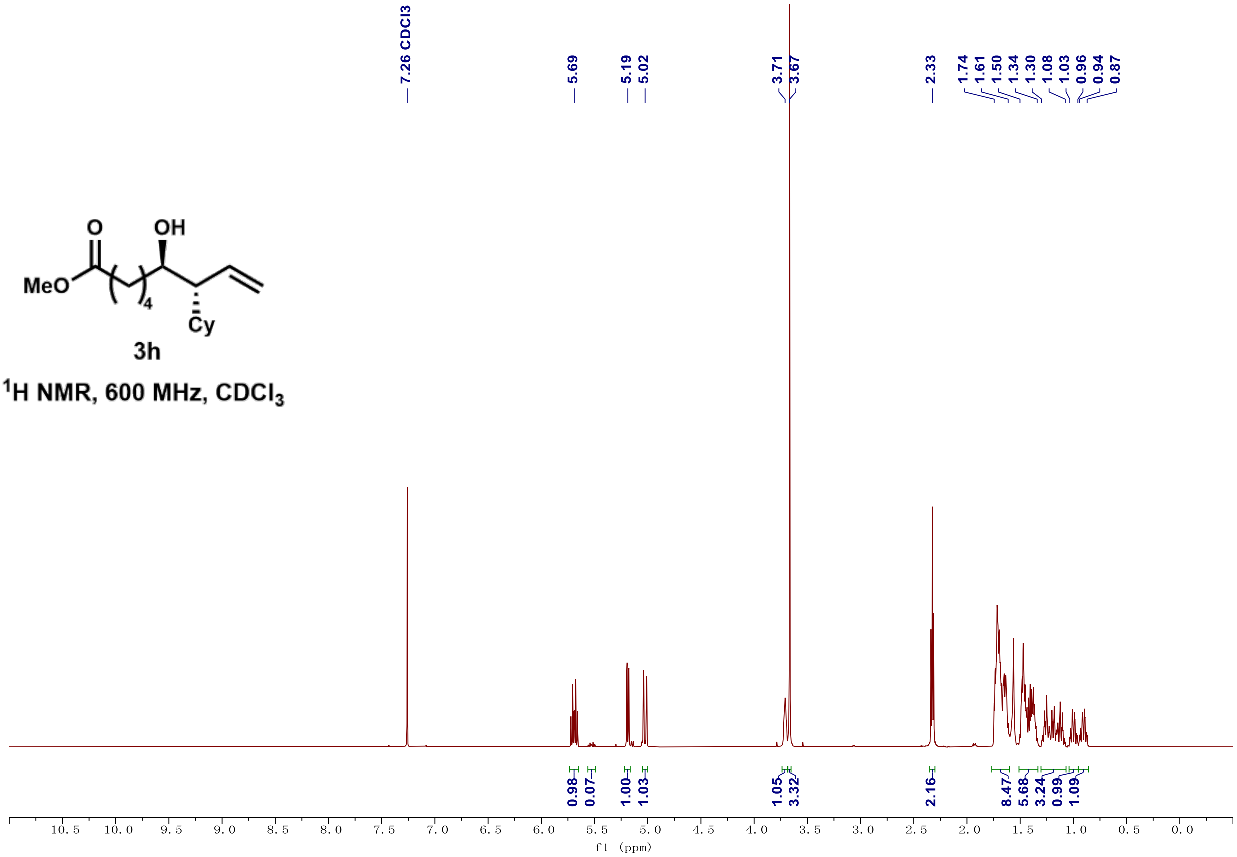

3h

^{1}H NMR, 600 MHz, $CDCl_3$

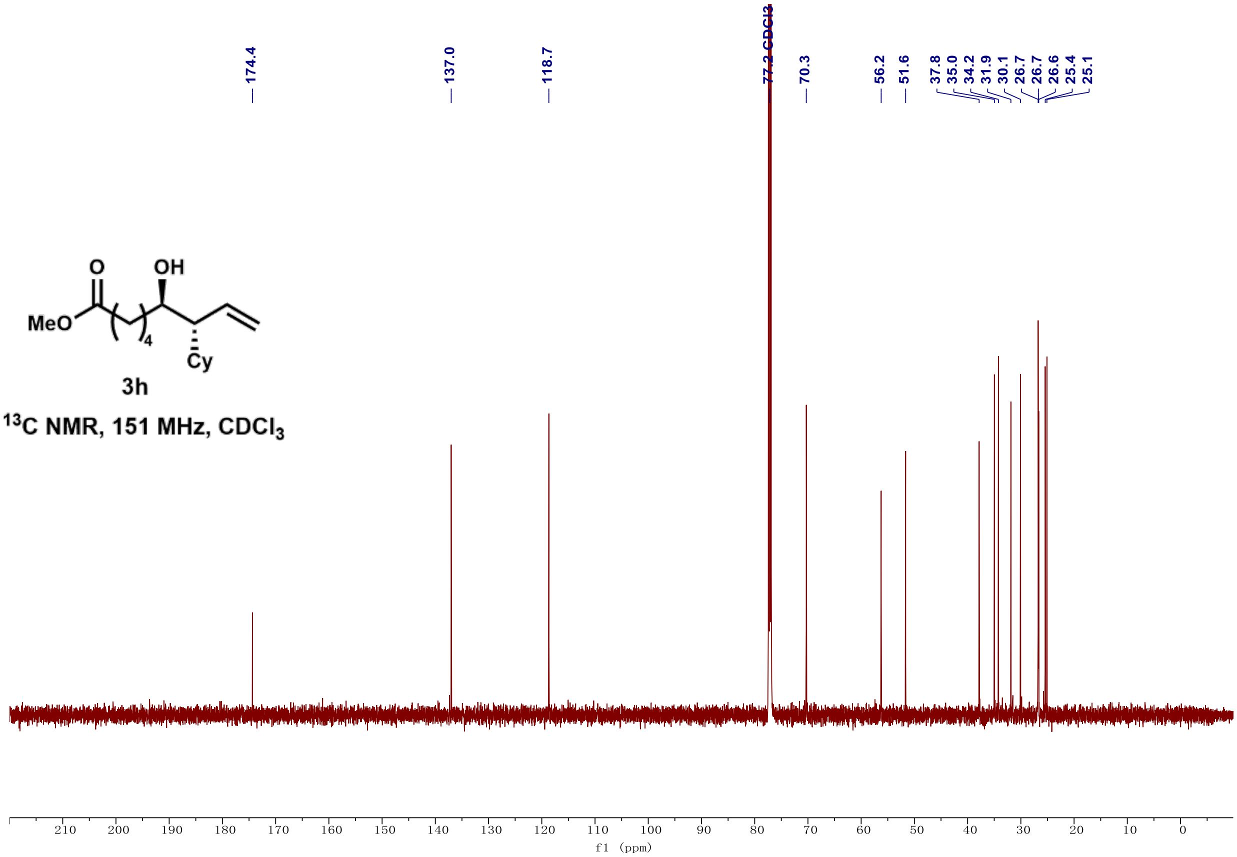

3h

^{13}C NMR, 151 MHz, $CDCl_3$

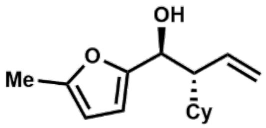

3i

^{1}H NMR, 600 MHz, $CDCl_3$

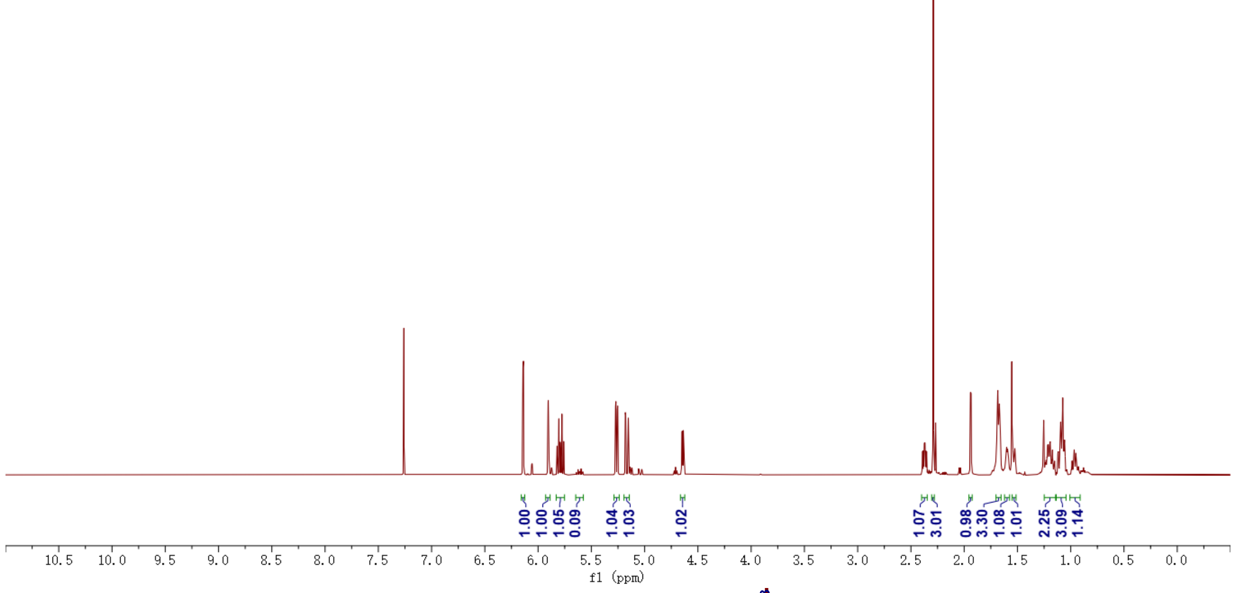

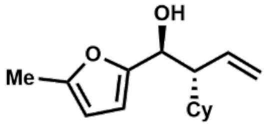

3i

^{13}C NMR, 151 MHz, $CDCl_3$

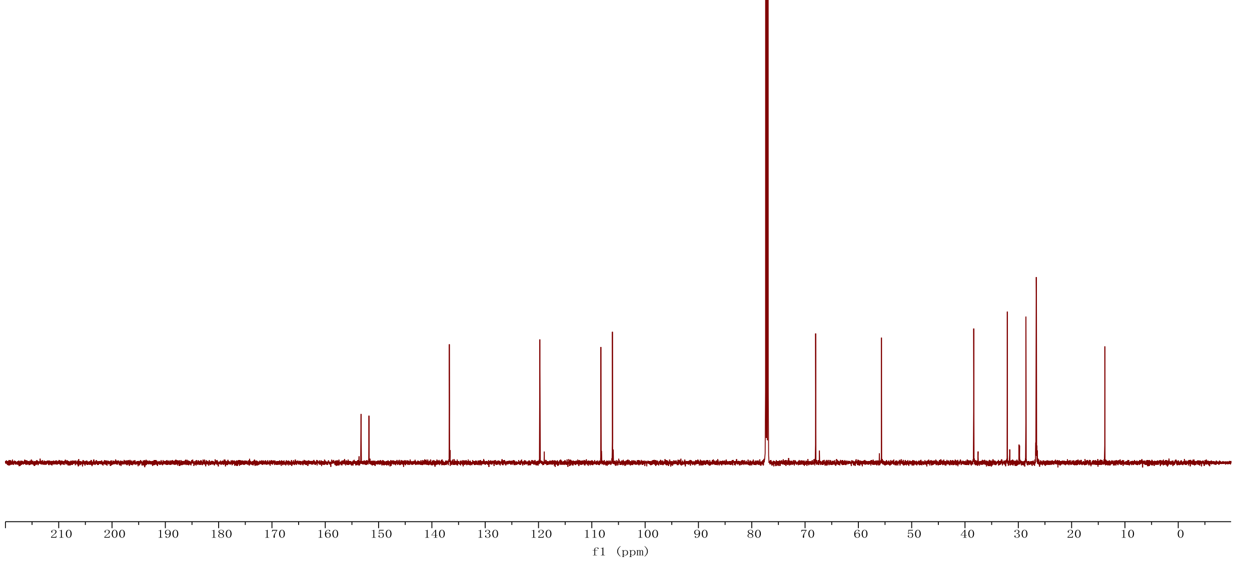

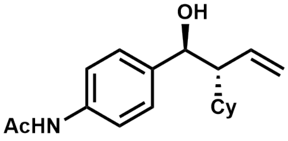

3j

^{1}H NMR, 600 MHz, $CDCl_3$

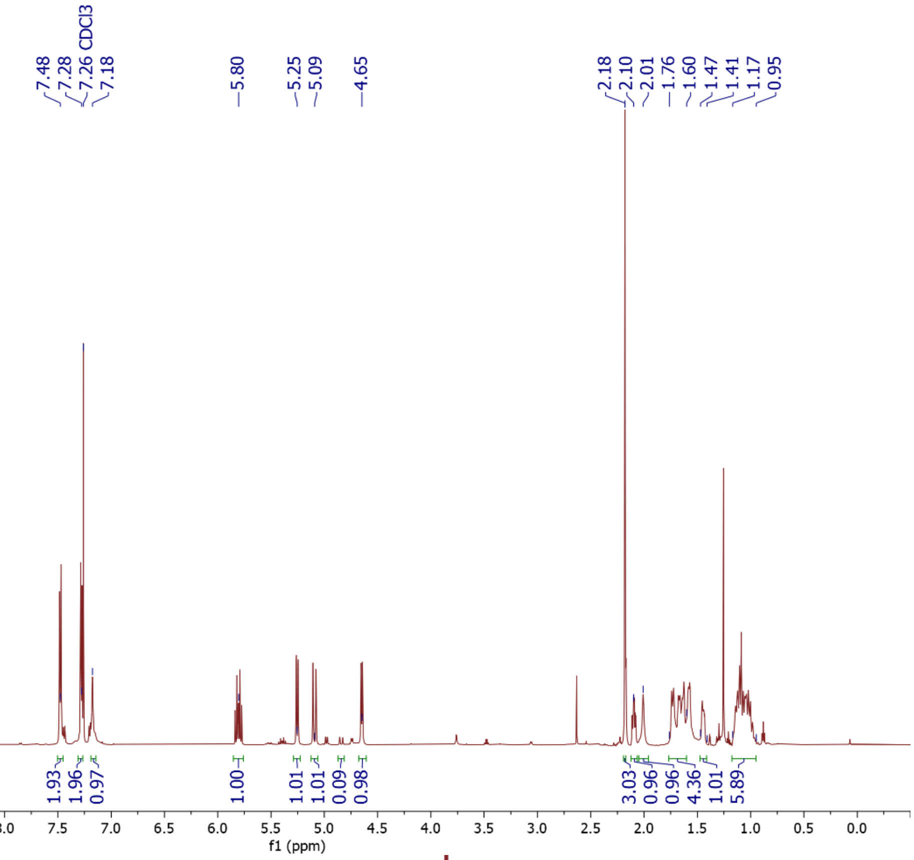

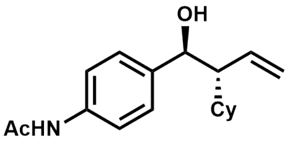

3j

^{13}C NMR, 151 MHz, $CDCl_3$

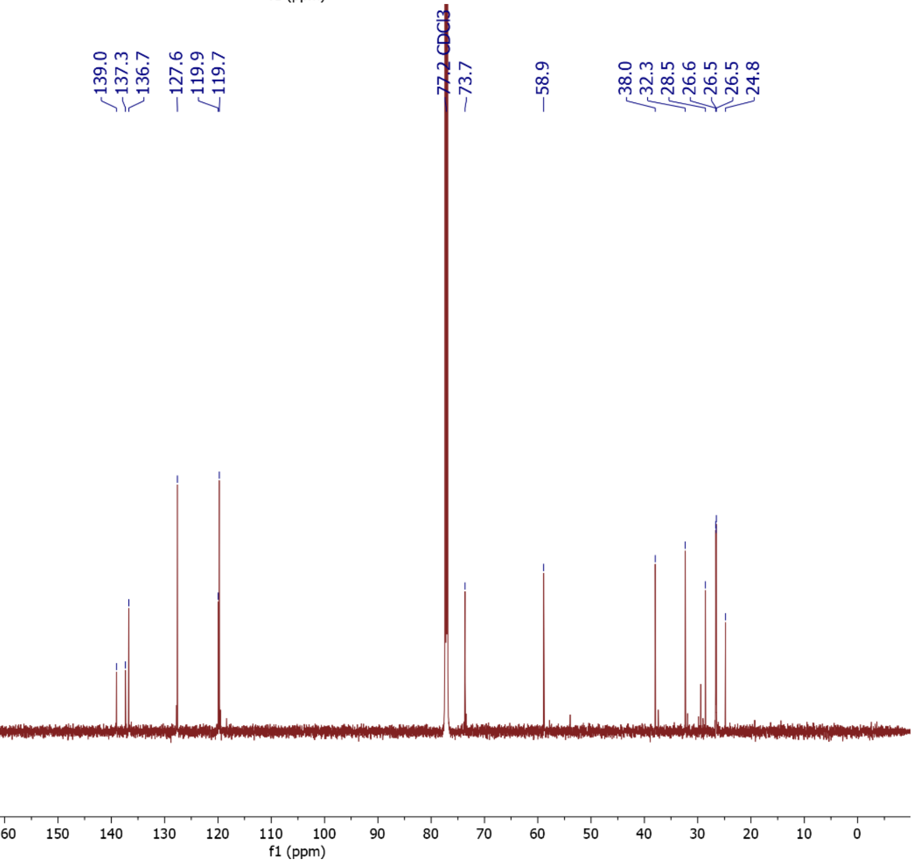

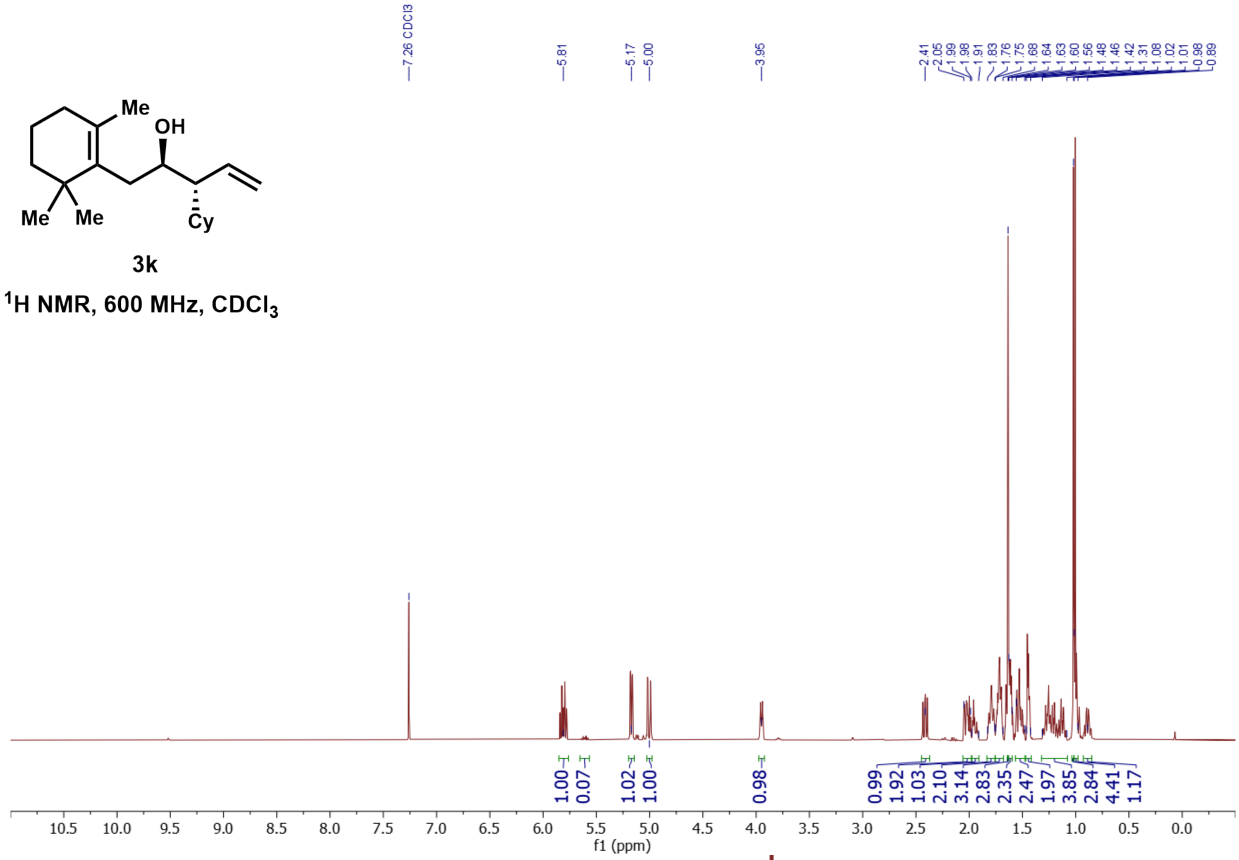
3k
^{1}H NMR, 600 MHz, $CDCl_3$
7.26 CDCl3
5.81
5.17
5.00
3.95
f1 (ppm)

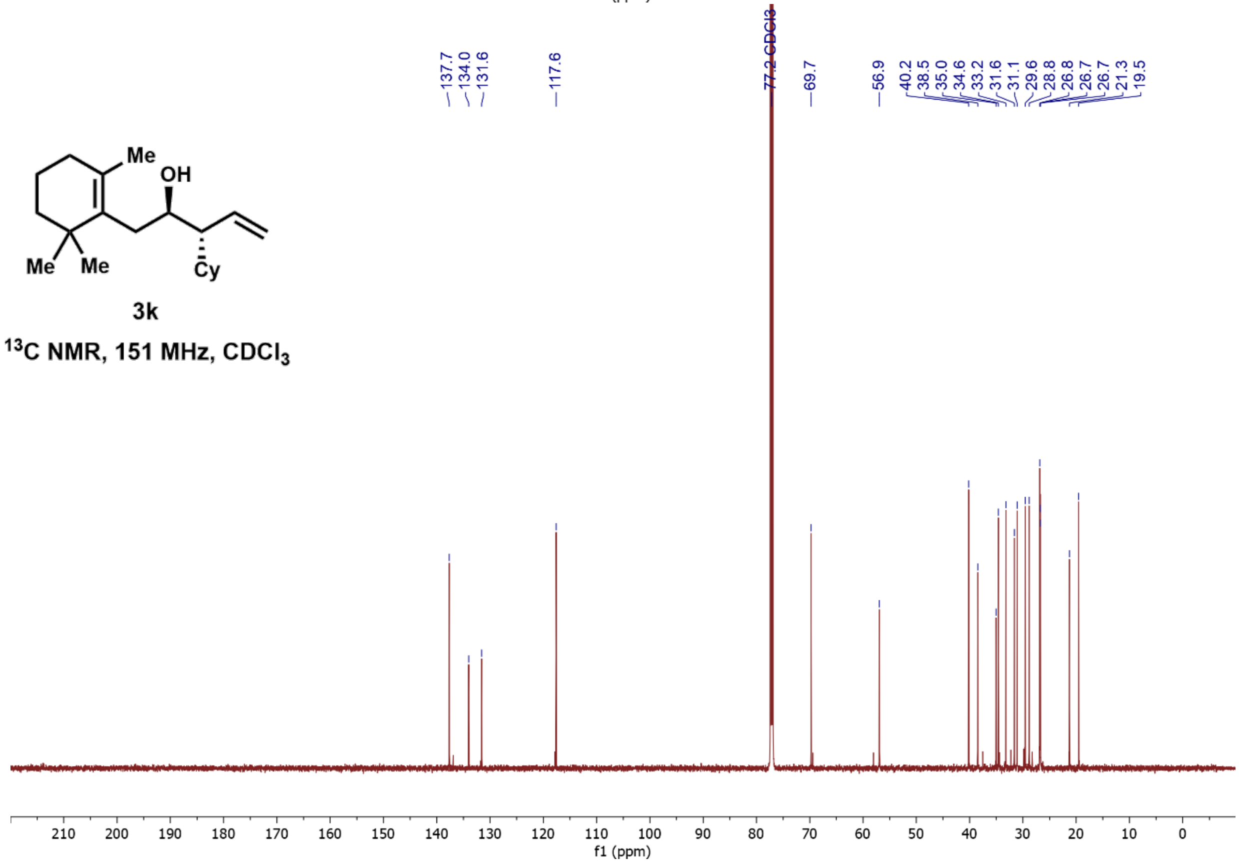
3k
^{13}C NMR, 151 MHz, $CDCl_3$
137.7
134.0
131.6
117.6
77.2 CDCl3
69.7
56.9
f1 (ppm)

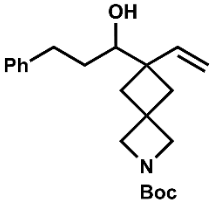

3l

^{1}H NMR, 600 MHz, $CDCl_3$

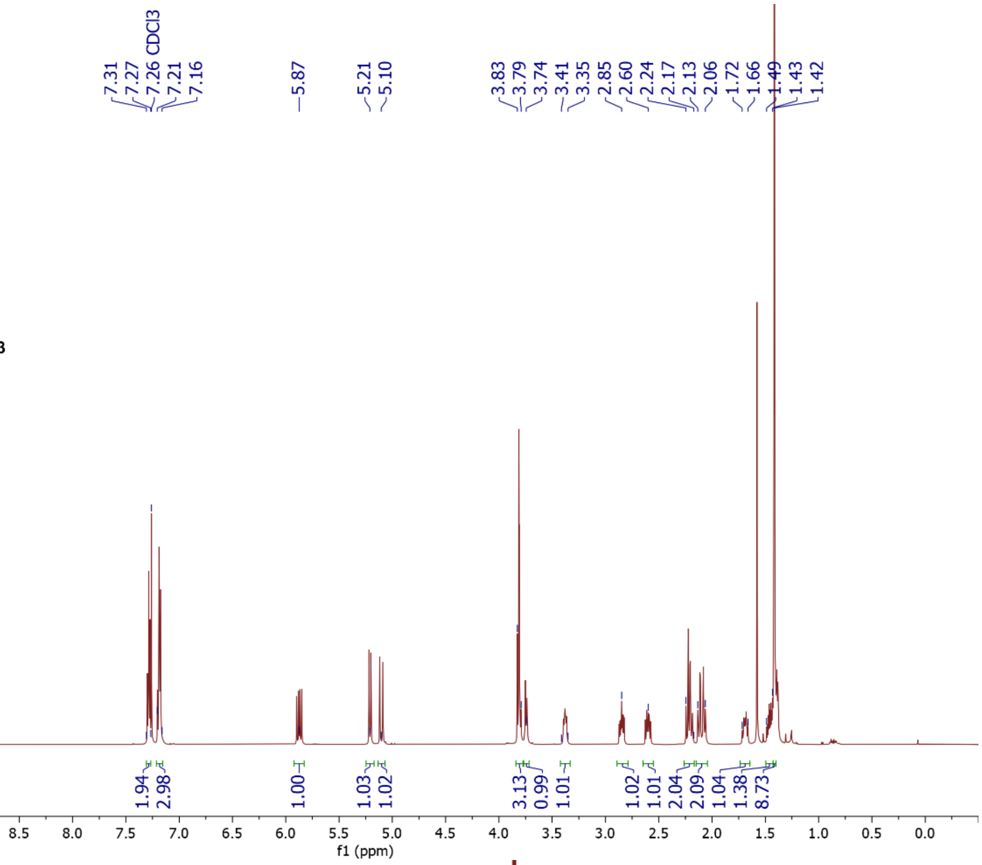

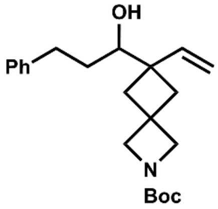

3l

^{13}C NMR, 151 MHz, $CDCl_3$

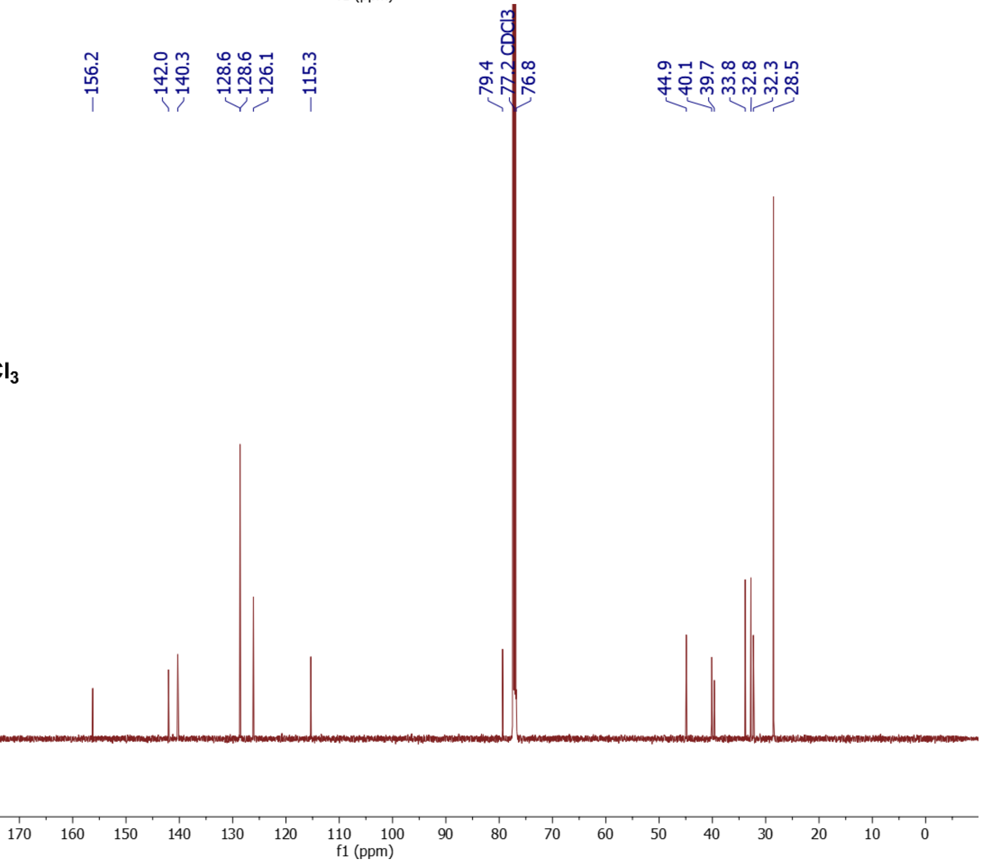

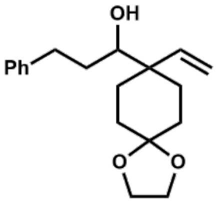

3m

^{1}H NMR, 600 MHz, $CDCl_3$

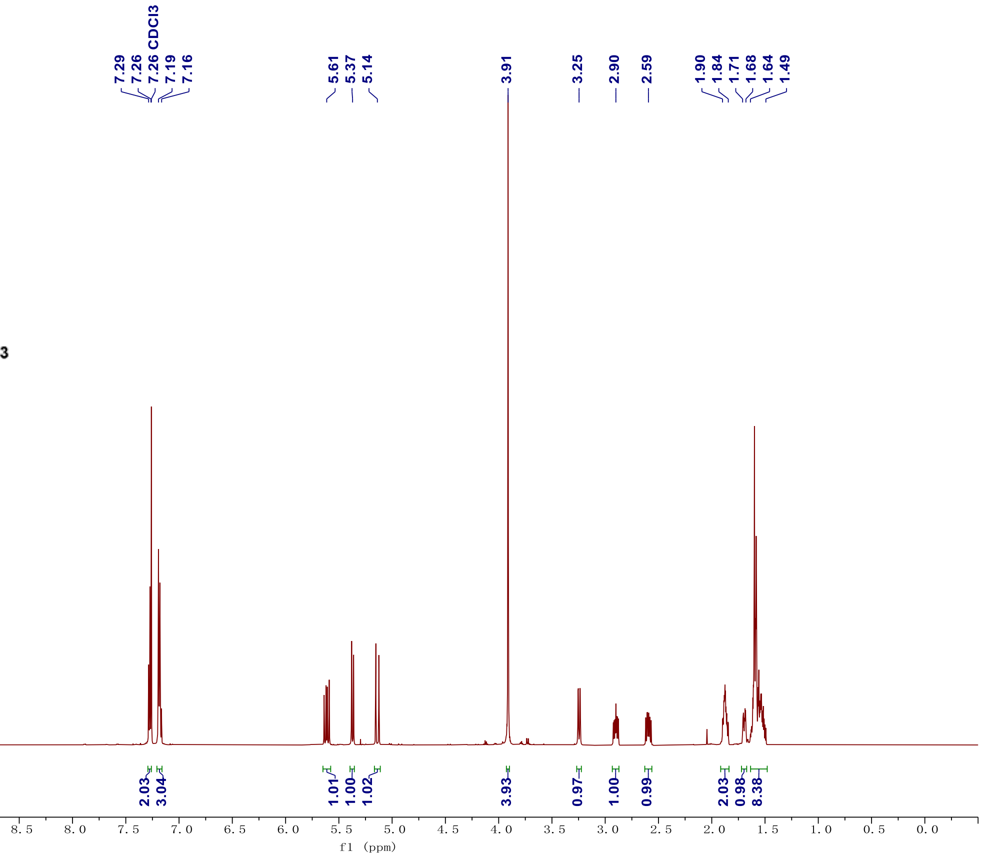

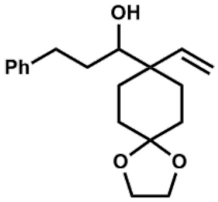

3m

^{13}C NMR, 151 MHz, $CDCl_3$

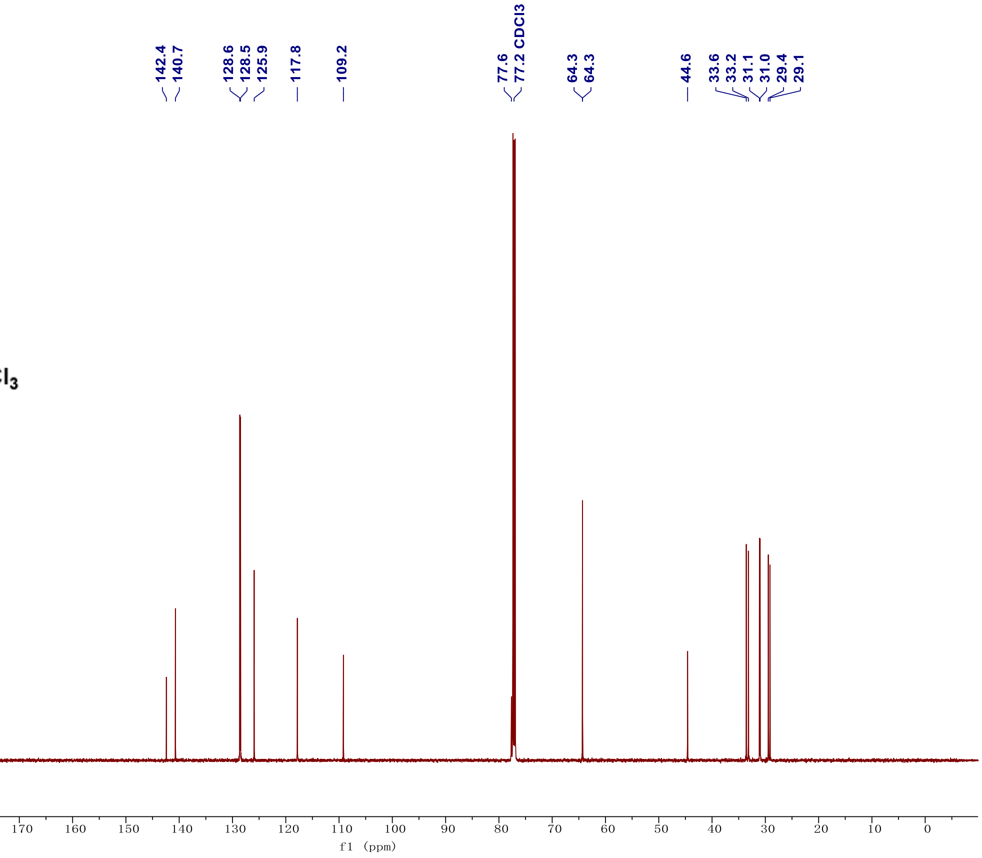

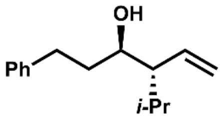

3n

^{1}H NMR, 600 MHz, $CDCl_3$

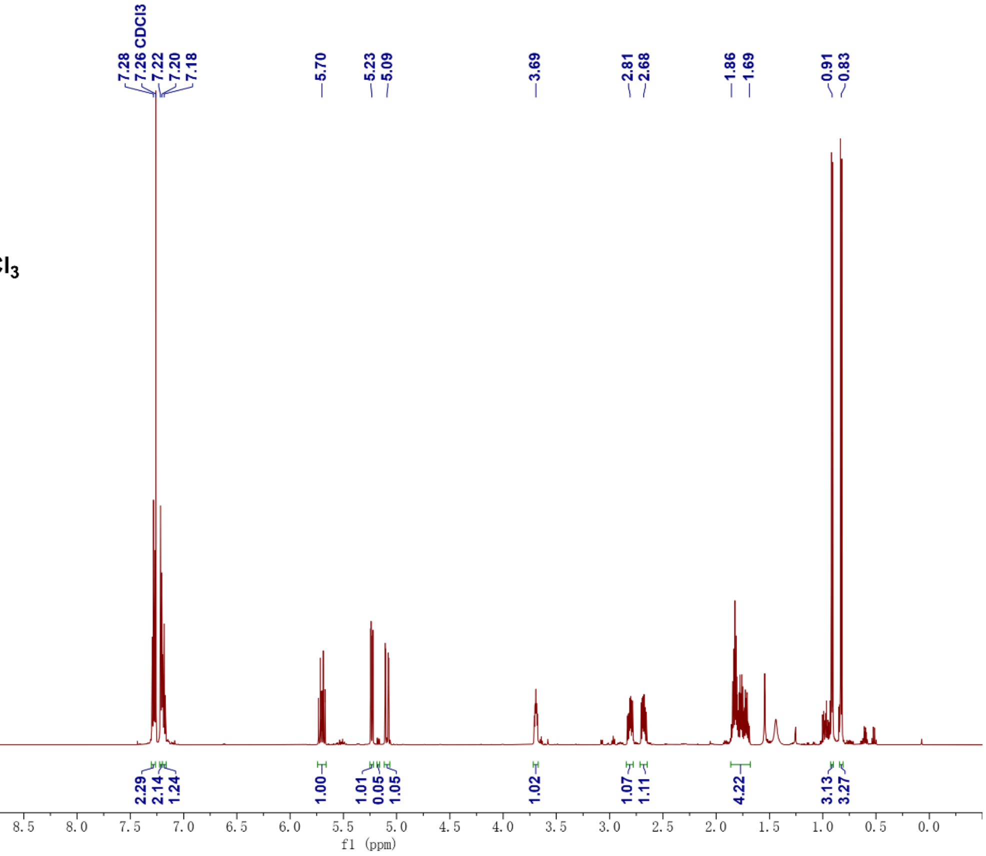

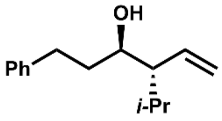

3n

^{13}C NMR, 151 MHz, $CDCl_3$

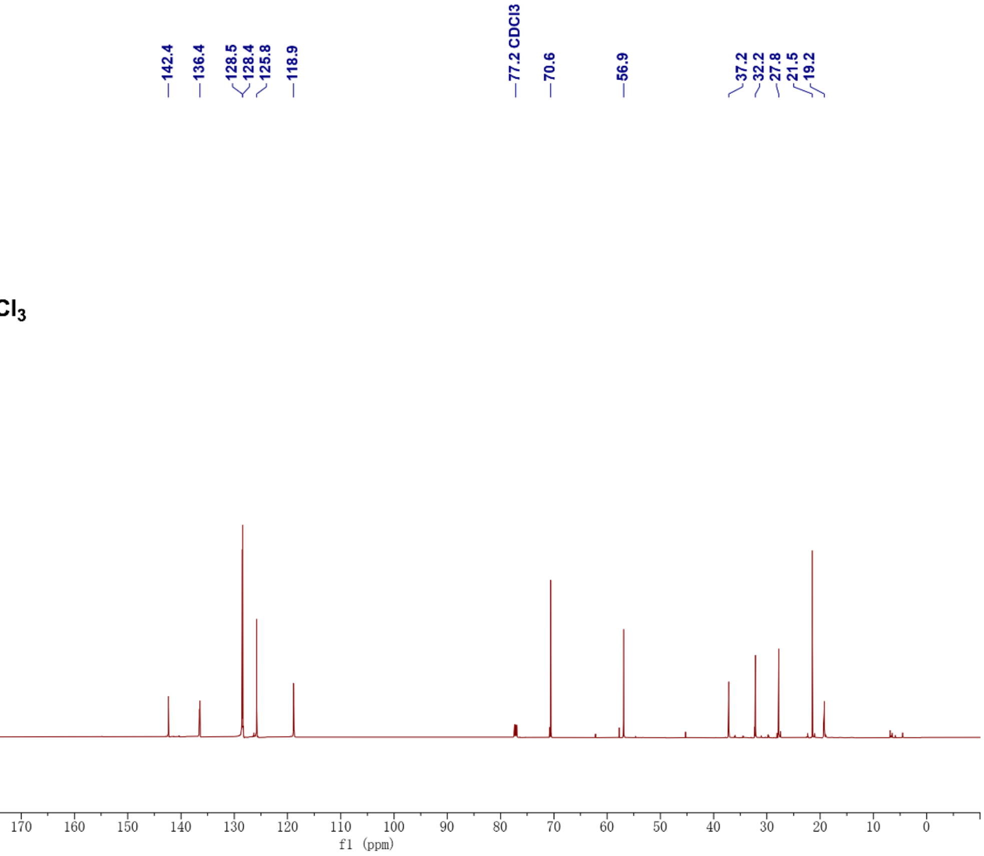

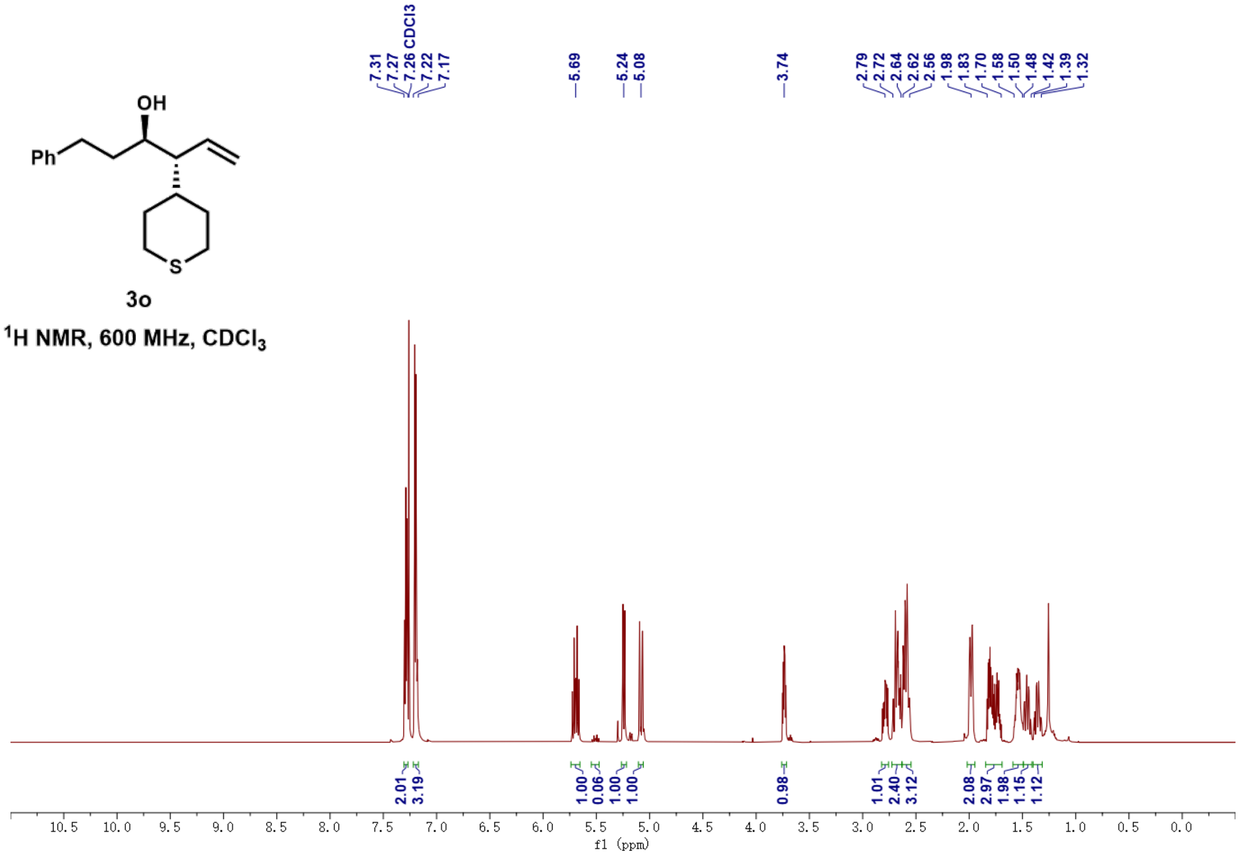

OH
Ph
S
3o
¹H NMR, 600 MHz, CDCl₃
7.31
7.27
7.26 CDCl3
7.22
7.17
5.69
5.24
5.08
3.74
2.79
2.72
2.64
2.62
2.56
1.98
1.83
1.70
1.58
1.50
1.48
1.42
1.39
1.32
2.01
3.19
1.00
0.06
1.00
1.00
0.98
1.01
2.40
3.12
2.08
2.97
1.98
1.15
1.12
f1 (ppm)

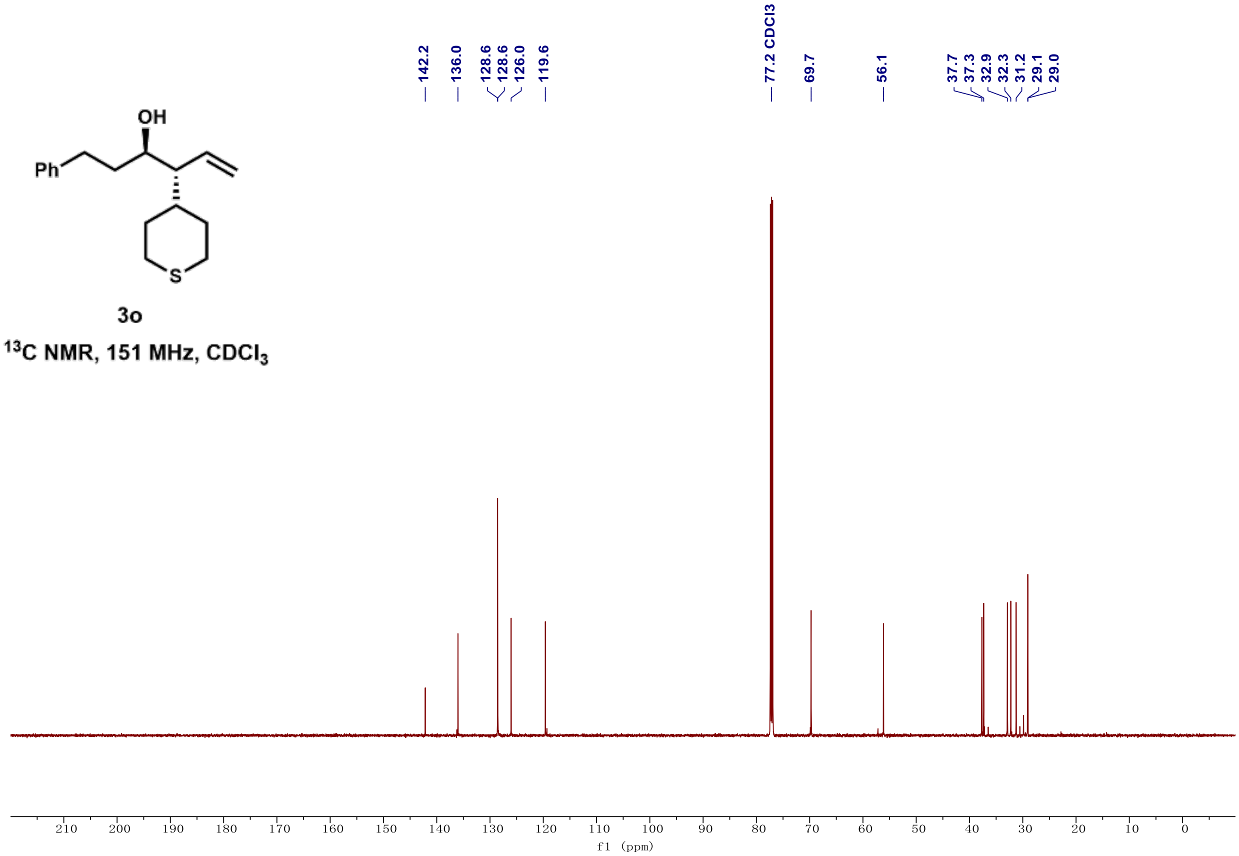

OH
Ph
S
3o
¹³C NMR, 151 MHz, CDCl₃
142.2
136.0
128.6
128.6
126.0
119.6
77.2 CDCl3
69.7
56.1
37.7
37.3
32.9
32.3
31.2
29.1
29.0
f1 (ppm)

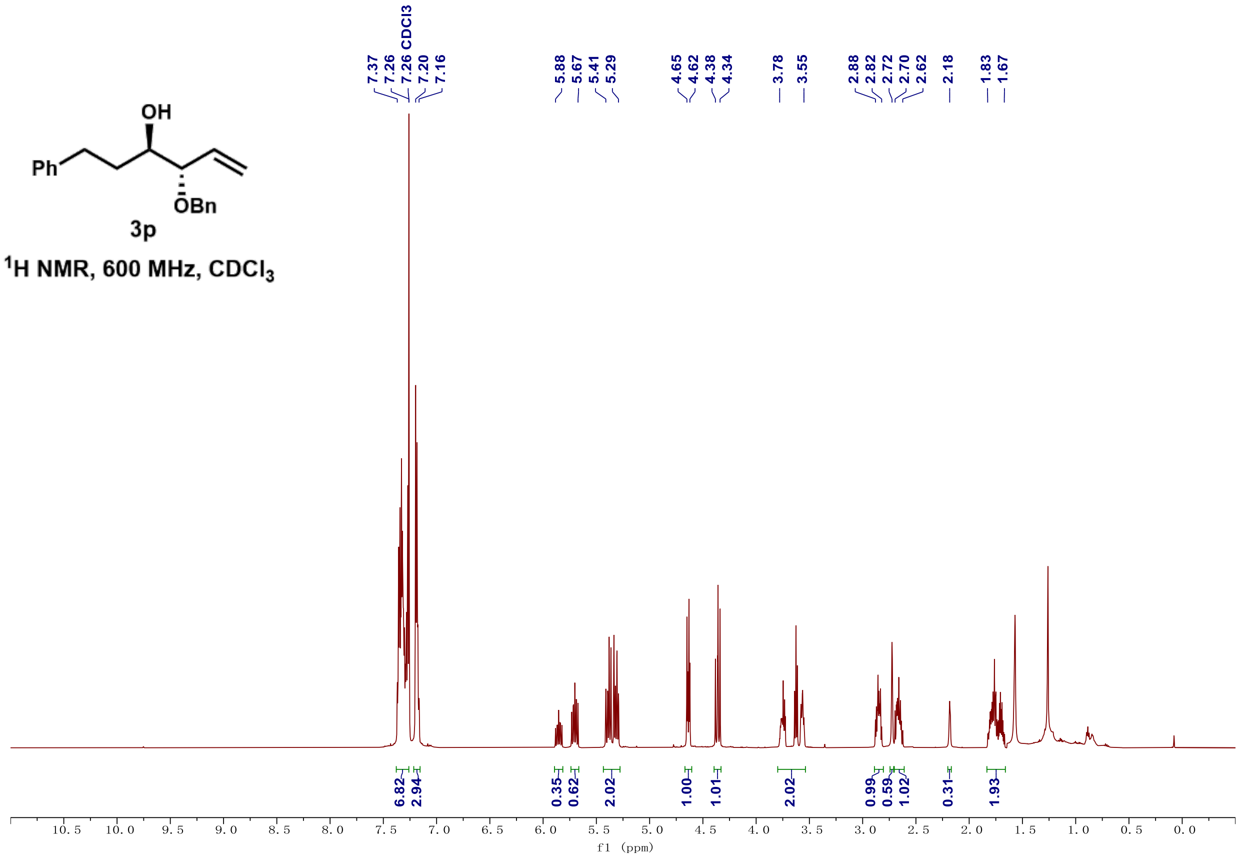
OH
Ph
OBn
3p
1H NMR, 600 MHz, CDCl3
7.37
7.26
7.26 CDCl3
7.20
7.16
5.88
5.67
5.41
5.29
4.65
4.62
4.38
4.34
3.78
3.55
2.88
2.82
2.72
2.70
2.62
2.18
1.83
1.67
6.82
2.94
0.35
0.62
2.02
1.00
1.01
2.02
0.99
0.59
1.02
0.31
1.93
f1 (ppm)

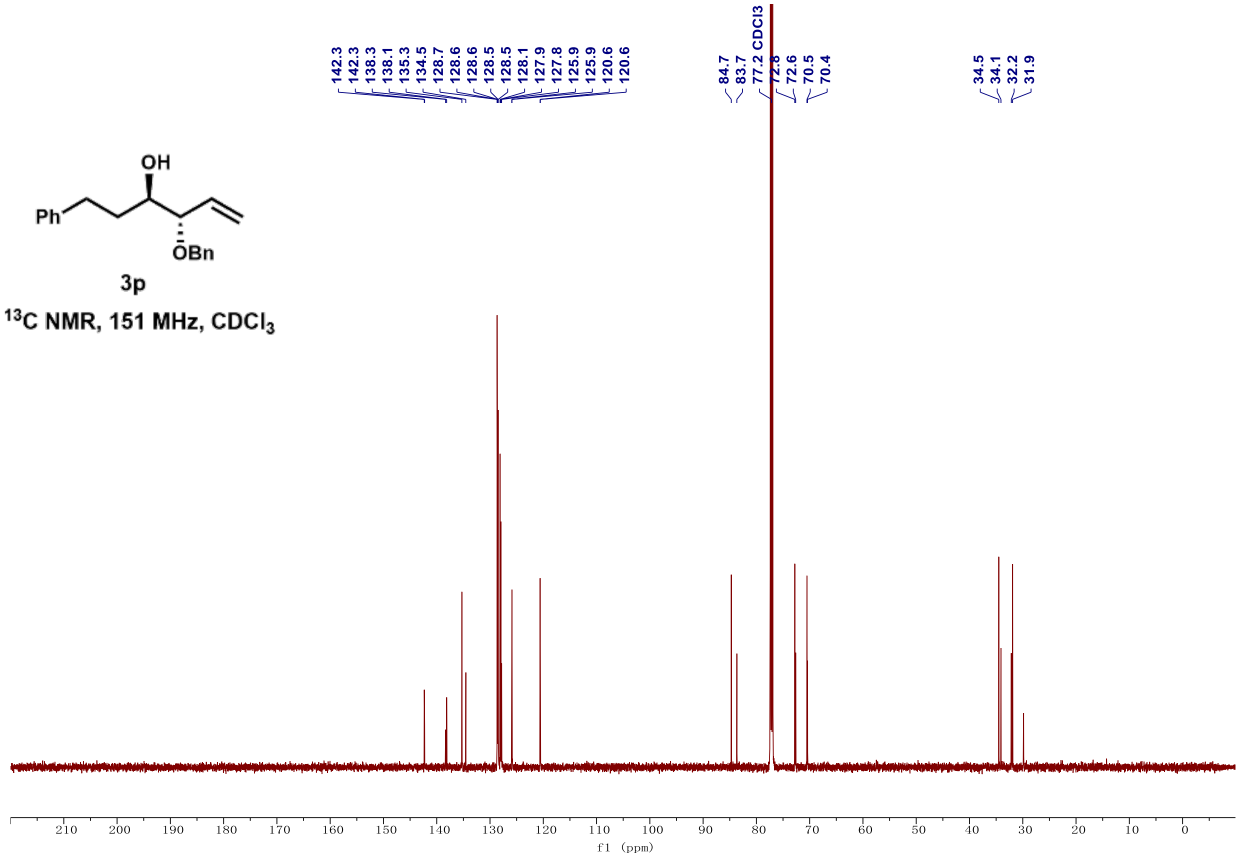
OH
Ph
OBn
3p
13C NMR, 151 MHz, CDCl3
142.3
142.3
138.3
138.1
135.3
134.5
128.7
128.6
128.6
128.5
128.5
128.1
127.9
127.8
125.9
125.9
120.6
120.6
84.7
83.7
77.2 CDCl3
72.8
72.6
70.5
70.4
34.5
34.1
32.2
31.9
f1 (ppm)

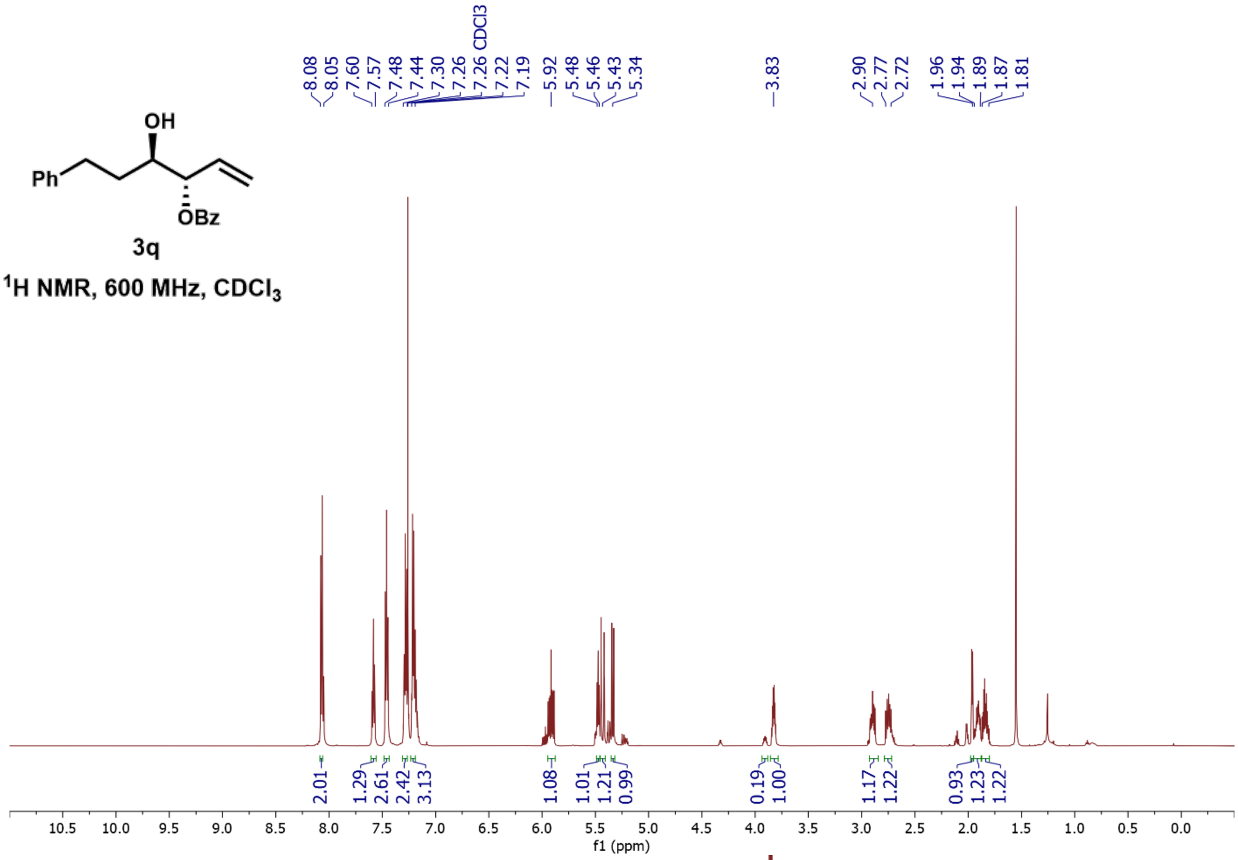

3q

^{1}H NMR, 600 MHz, $CDCl_3$

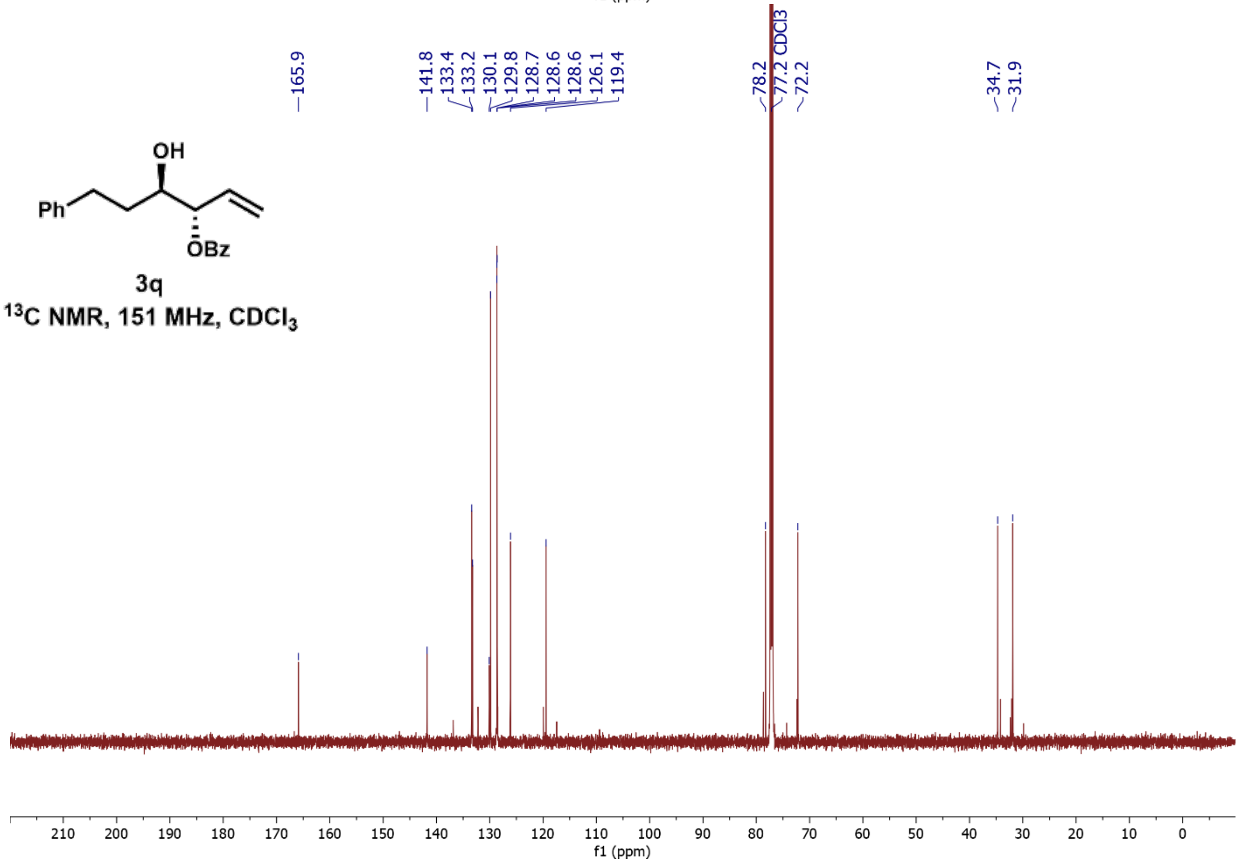

3q

^{13}C NMR, 151 MHz, $CDCl_3$

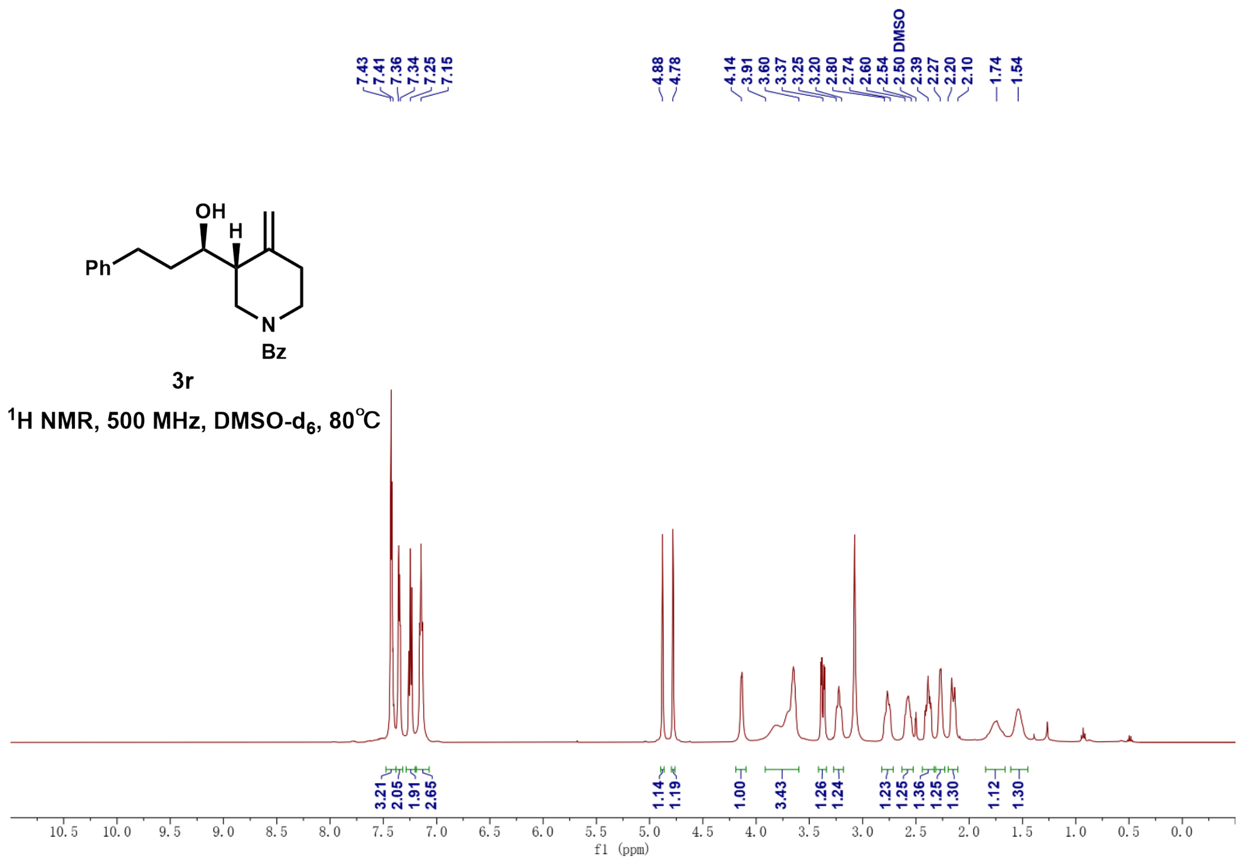
7.43 7.41 7.36 7.34 7.25 7.15
4.88 4.78
4.14 3.91 3.60 3.37 3.25 3.20 2.80 2.74 2.60 2.54 2.50 DMSO 2.39 2.27 2.20 2.10
1.74 1.54
OH
H
Ph
N
Bz
3r
¹H NMR, 500 MHz, DMSO-d6, 80°C
3.21 2.05 1.91 2.65
1.14 1.19
1.00 3.43 1.26 1.24 1.23 1.25 1.36 1.25 1.30 1.12 1.30
f1 (ppm)

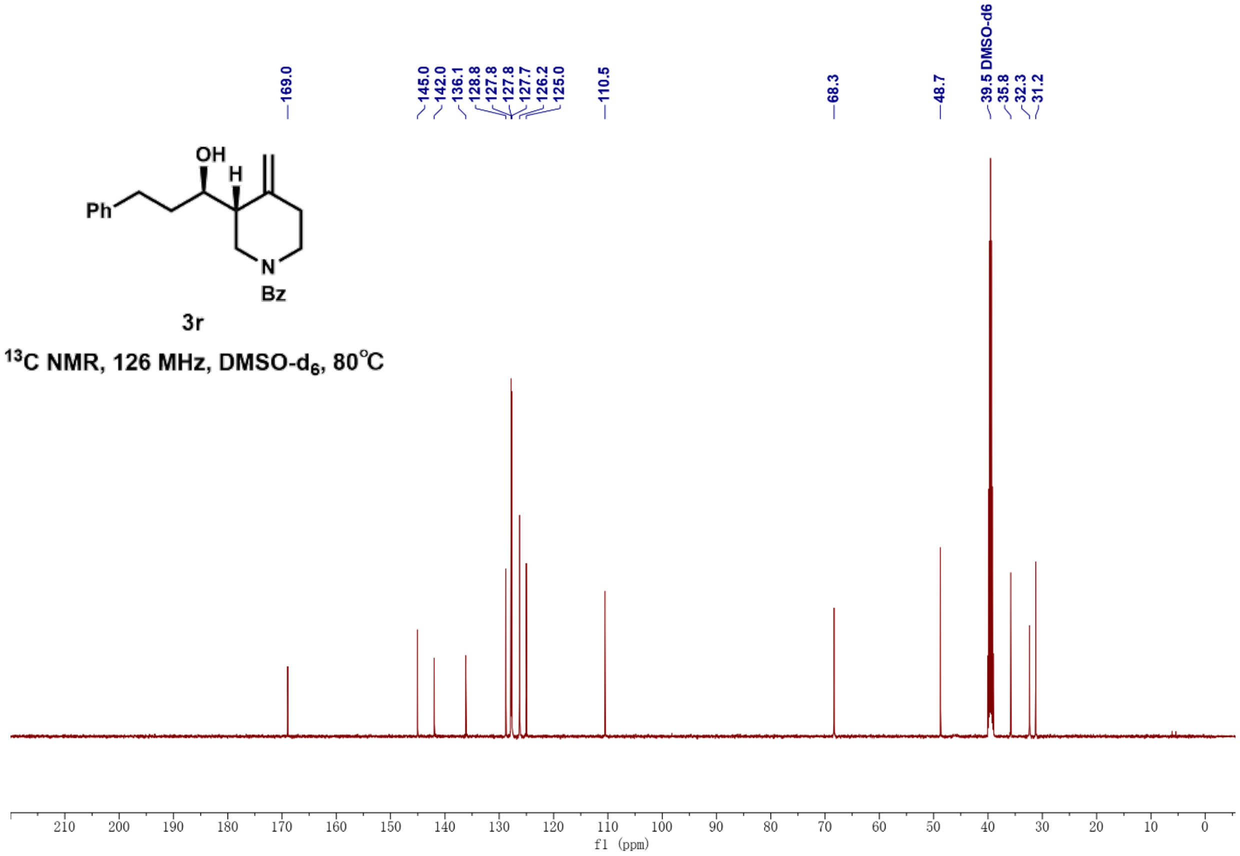
169.0
145.0 142.0 136.1 128.8 127.8 127.8 127.7 126.2 125.0
110.5
68.3
48.7
39.5 DMSO-d6 35.8 32.3 31.2
OH
H
Ph
N
Bz
3r
¹³C NMR, 126 MHz, DMSO-d6, 80°C
f1 (ppm)

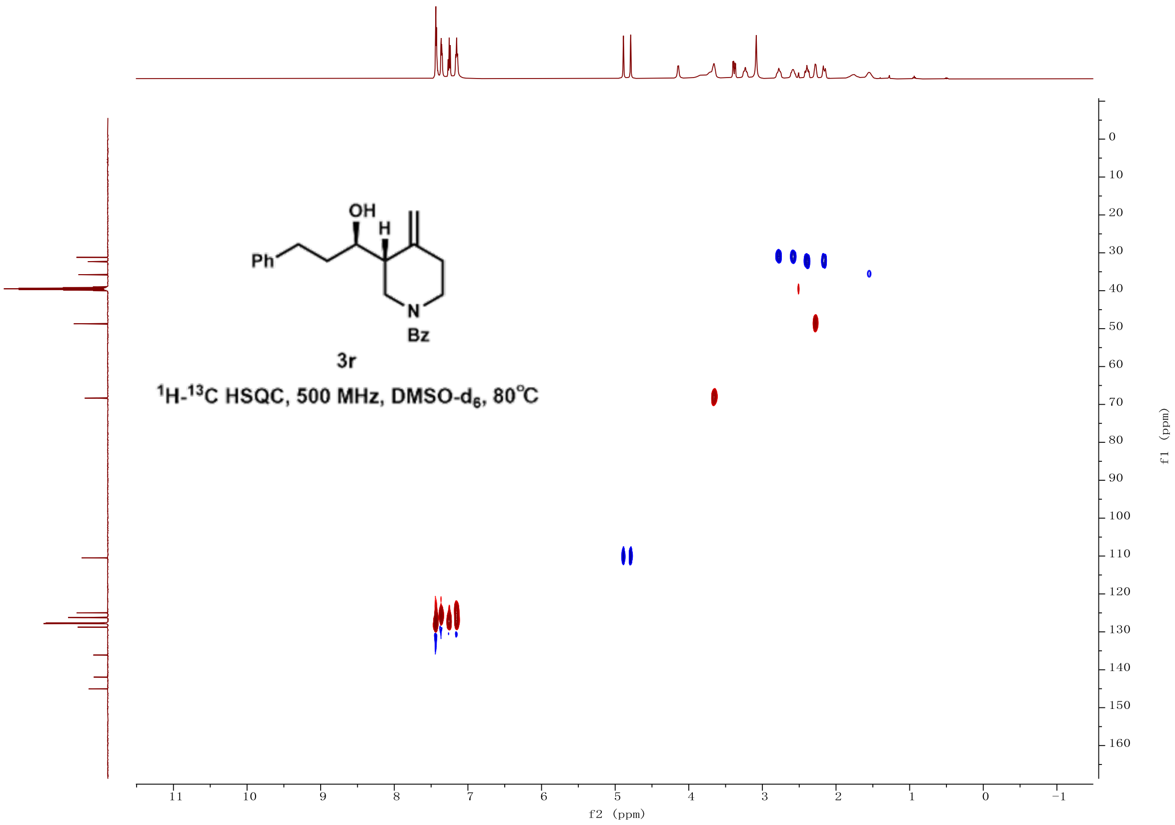
OH
H
Ph
N
Bz
3r
¹H-¹³C HSQC, 500 MHz, DMSO-d6, 80°C
f2 (ppm)
f1 (ppm)

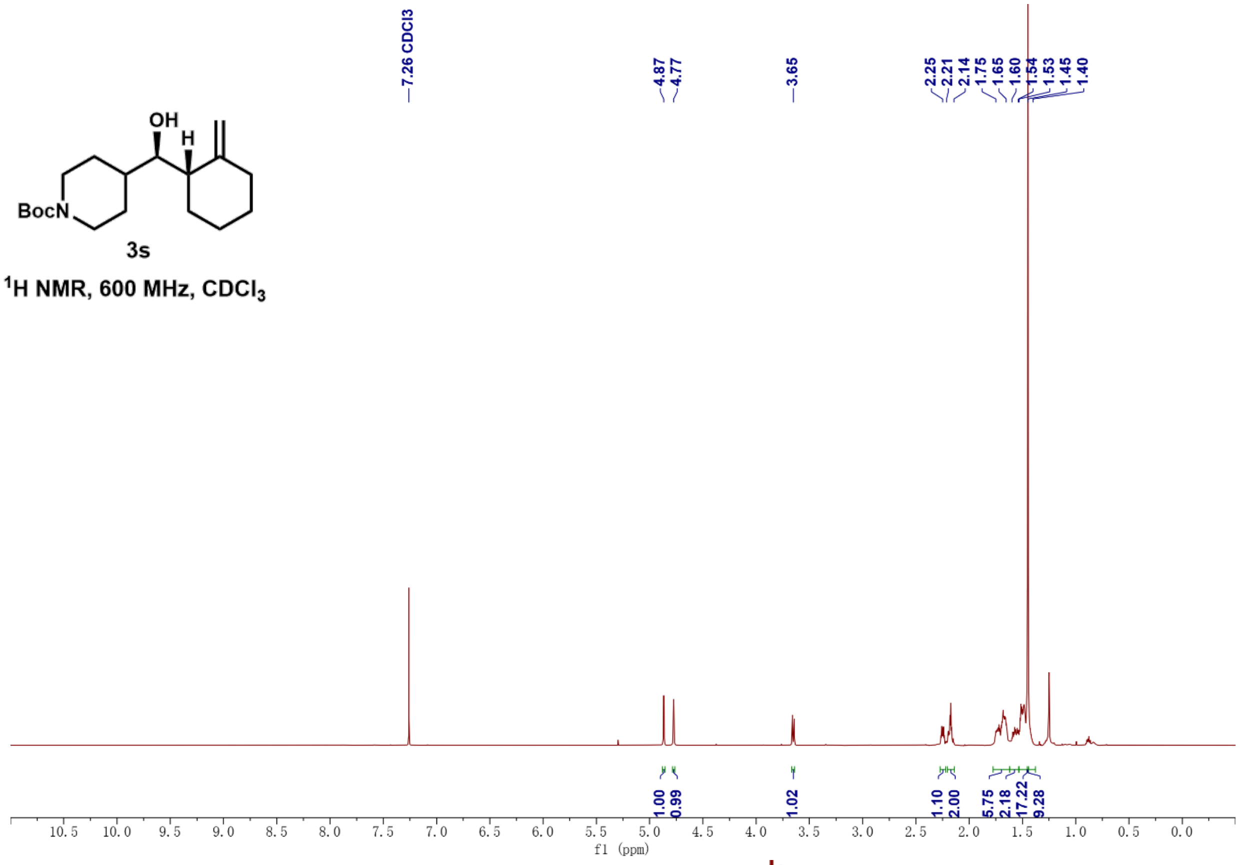

3s
^{1}H NMR, 600 MHz, CDCl$_3$
7.26 CDCl3
4.87
4.77
3.65
2.25
2.21
2.14
1.75
1.65
1.60
1.54
1.53
1.45
1.40
1.00
0.99
1.02
1.10
2.00
5.75
2.18
17.22
9.28
f1 (ppm)

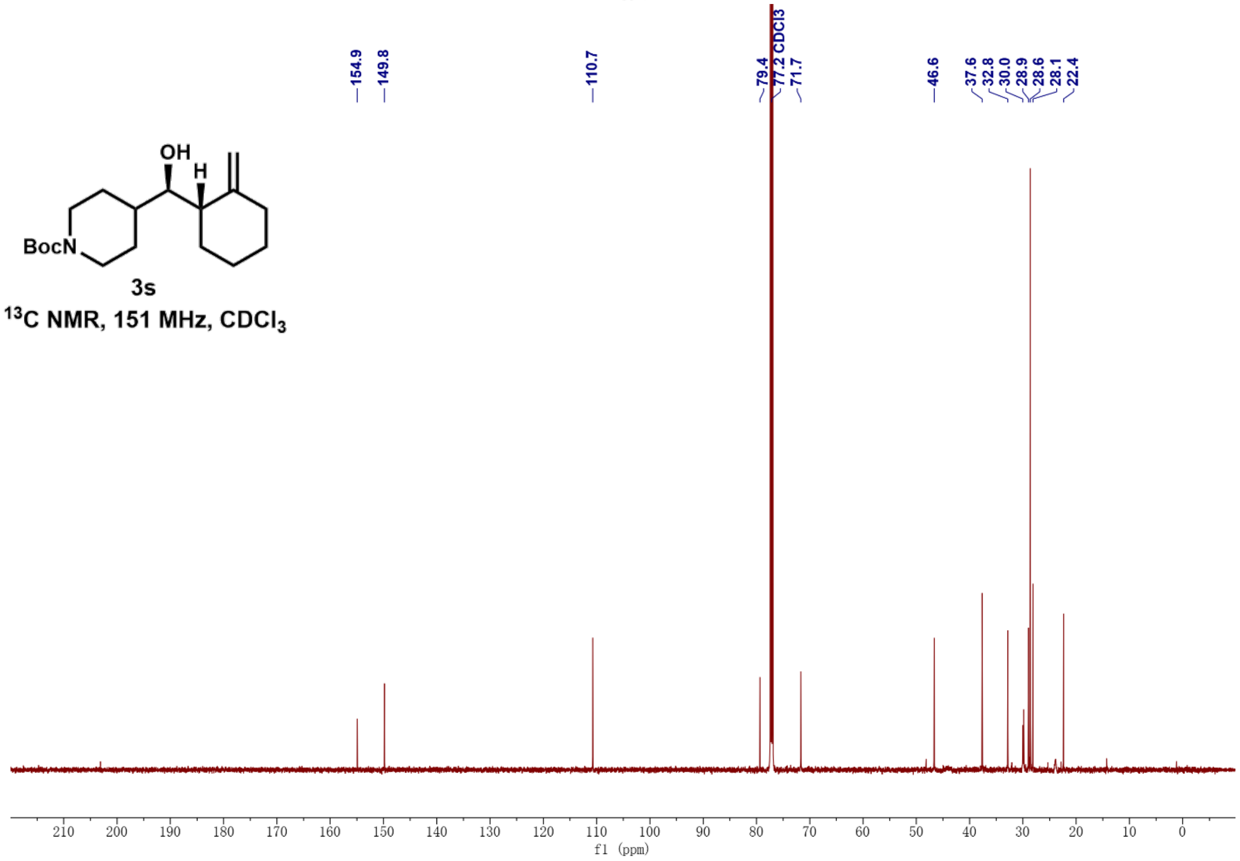

3s
^{13}C NMR, 151 MHz, CDCl$_3$
154.9
149.8
110.7
79.4
77.2 CDCl3
71.7
46.6
37.6
32.8
30.0
28.9
28.6
28.1
22.4
f1 (ppm)

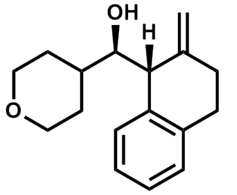

3t

^{1}H NMR, 600 MHz, $CDCl_3$

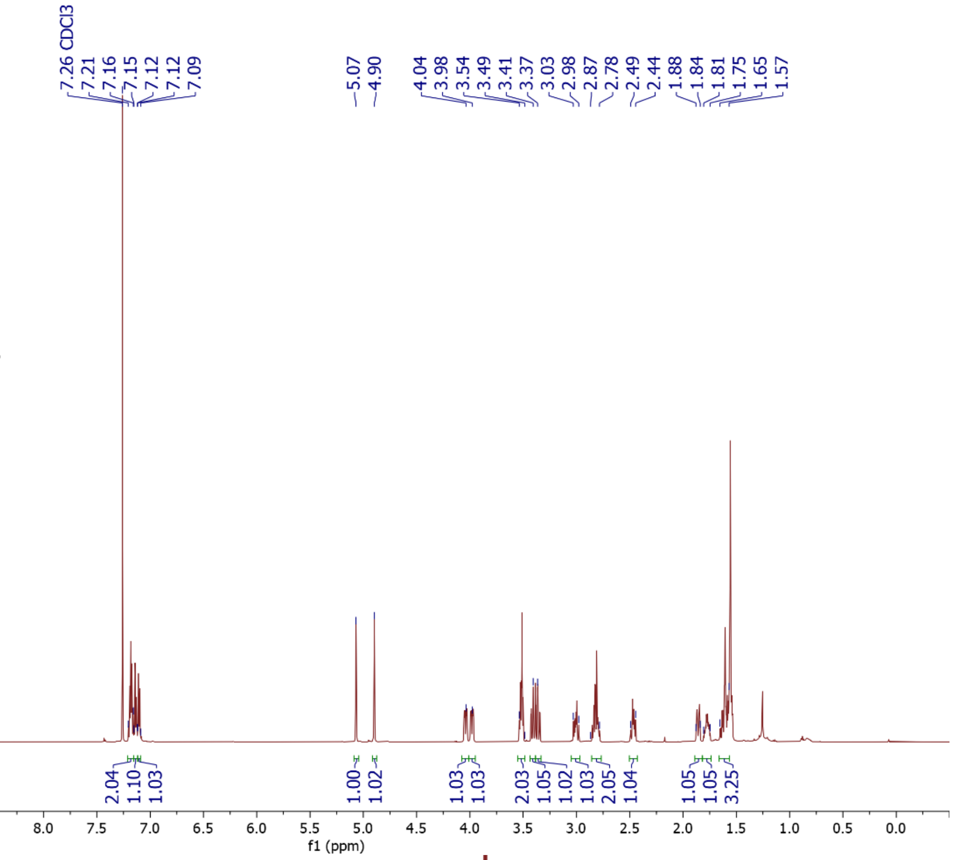

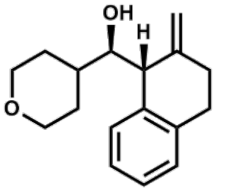

3t

^{13}C NMR, 151 MHz, $CDCl_3$

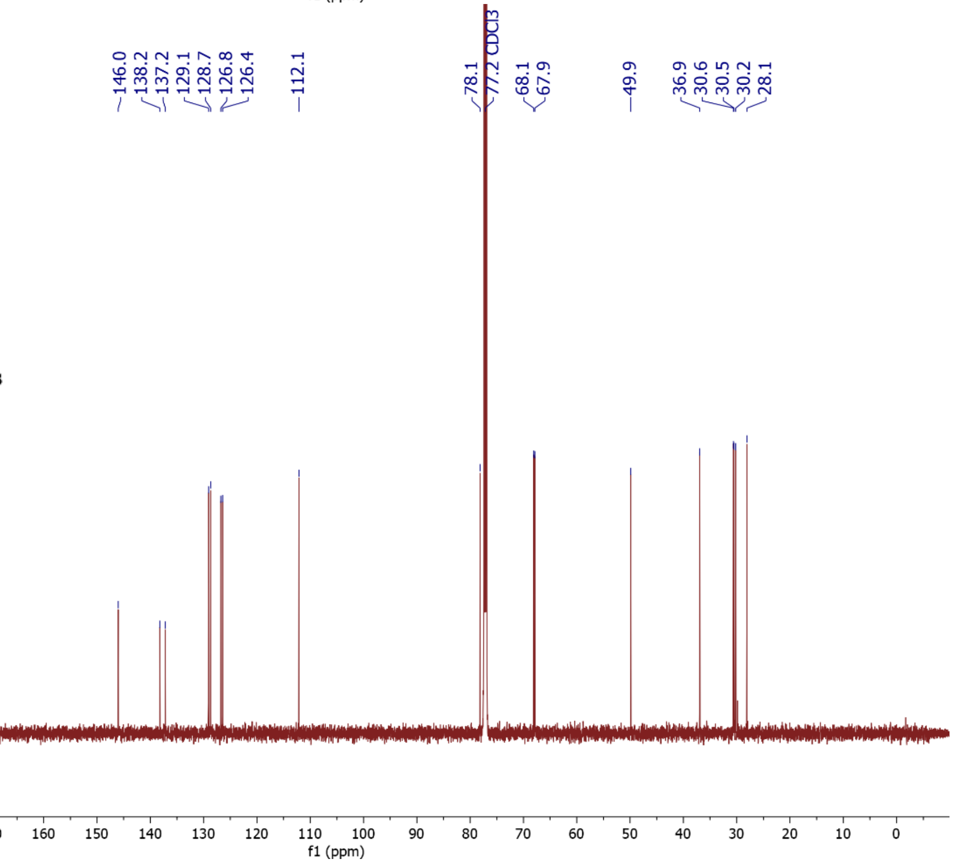

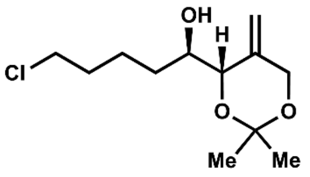

3u

^{1}H NMR, 600 MHz, C_6D_6

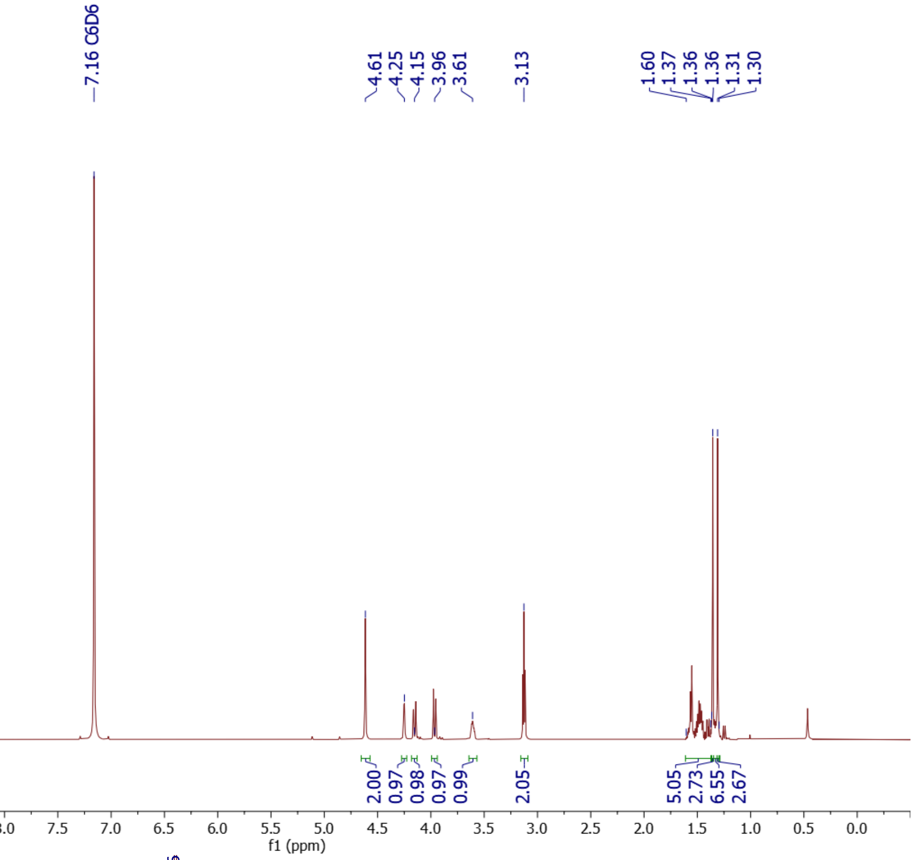

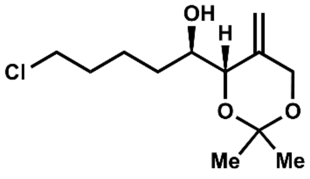

3u

^{13}C NMR, 151 MHz, C_6D_6

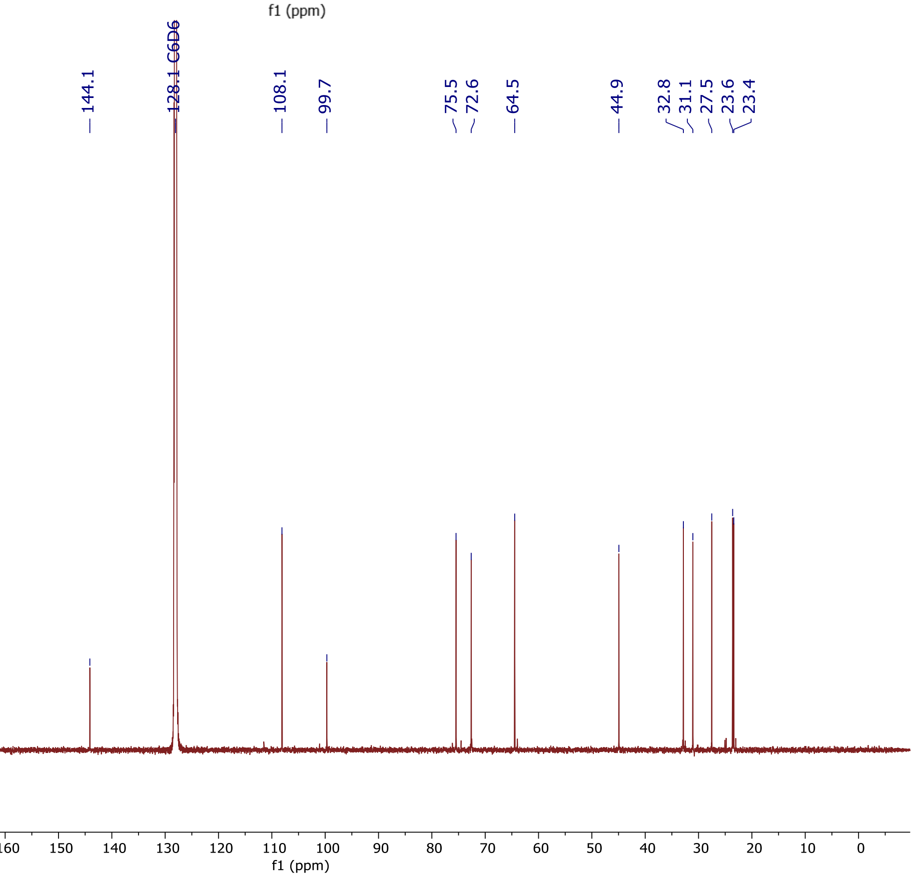

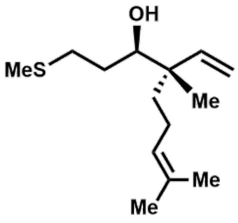

3v

^{1}H NMR, 600 MHz, $CDCl_3$

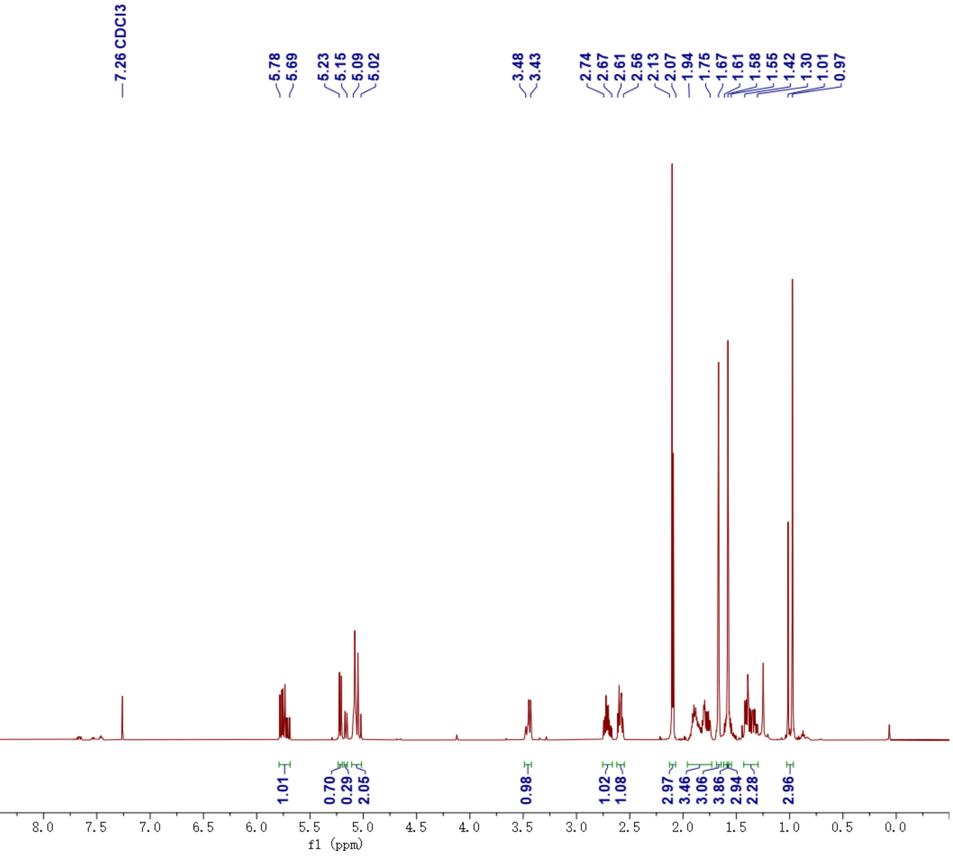

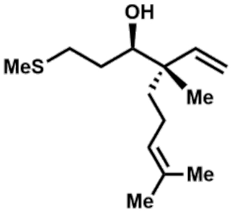

3v

^{13}C NMR, 151 MHz, $CDCl_3$

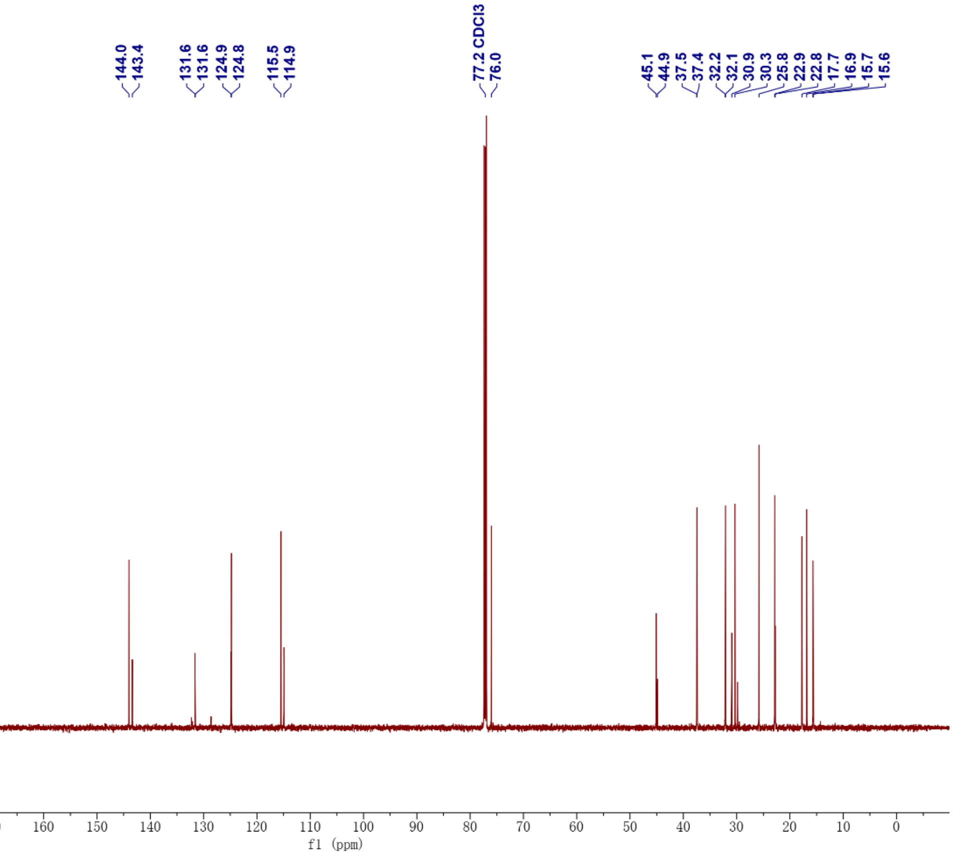

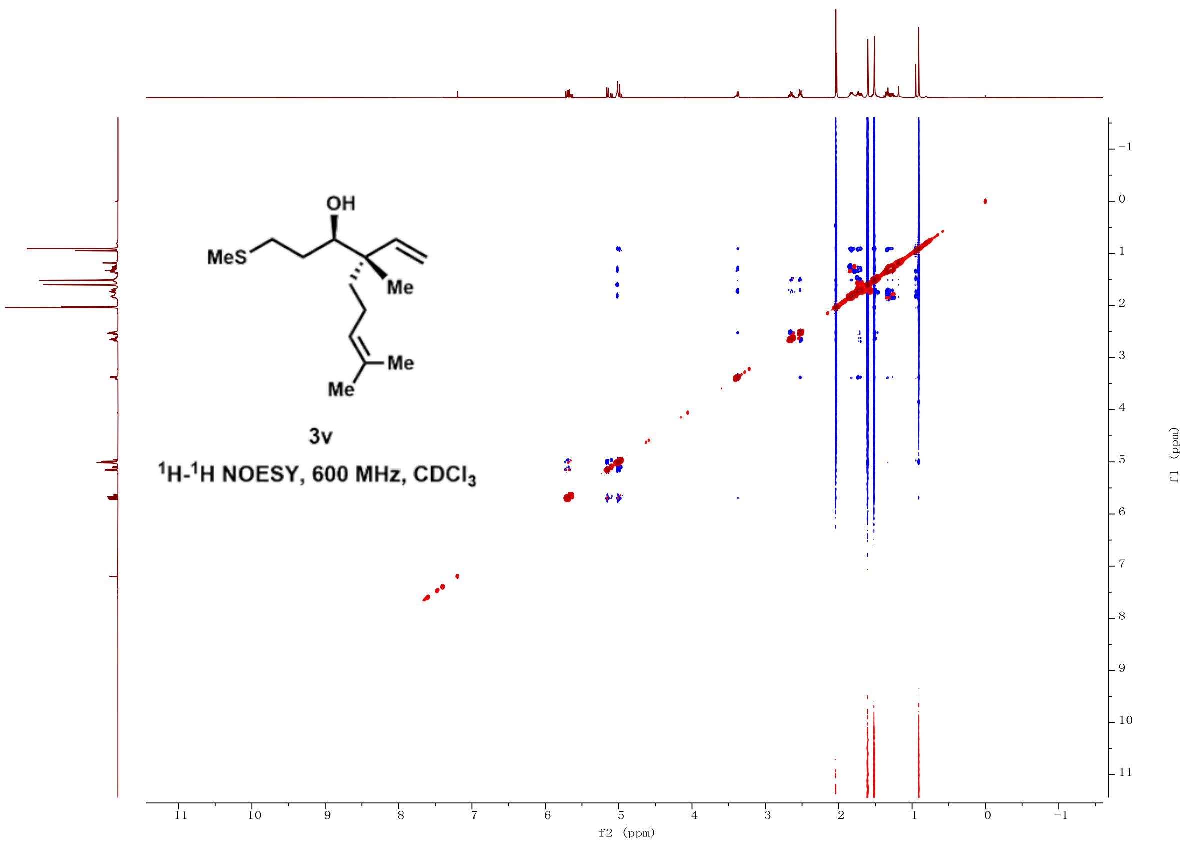
OH
MeS
Me
Me
Me
3v
1H-1H NOESY, 600 MHz, CDCl3
f2 (ppm)
f1 (ppm)

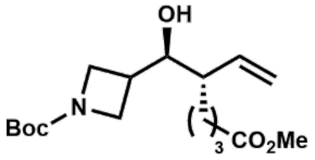

3w

^{1}H NMR, 600 MHz, $CDCl_3$

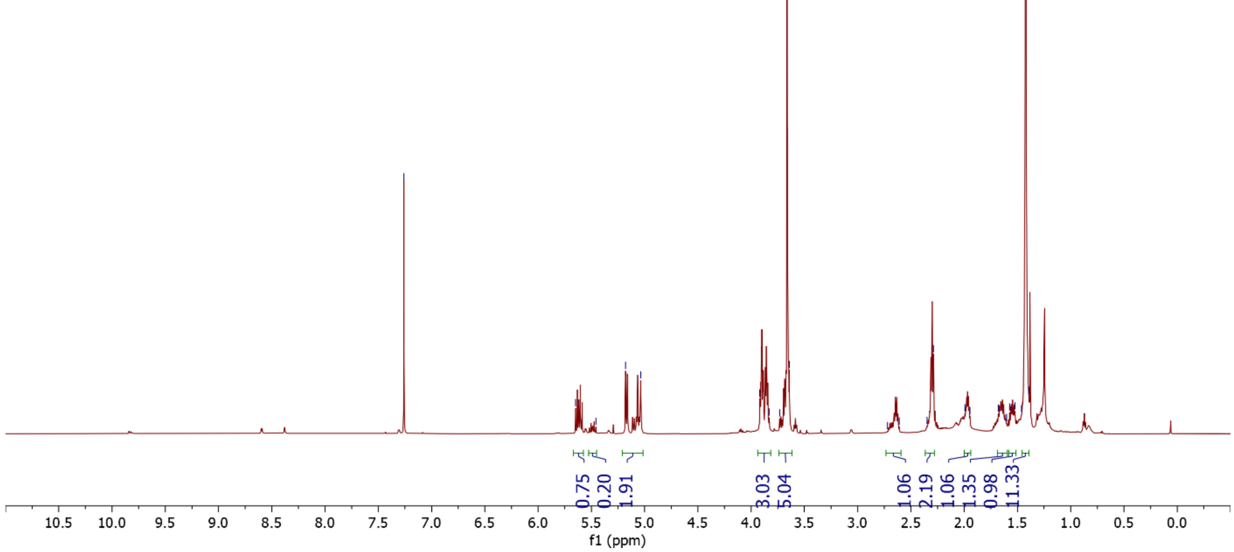

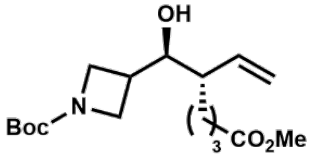

3w

^{13}C NMR, 151 MHz, $CDCl_3$

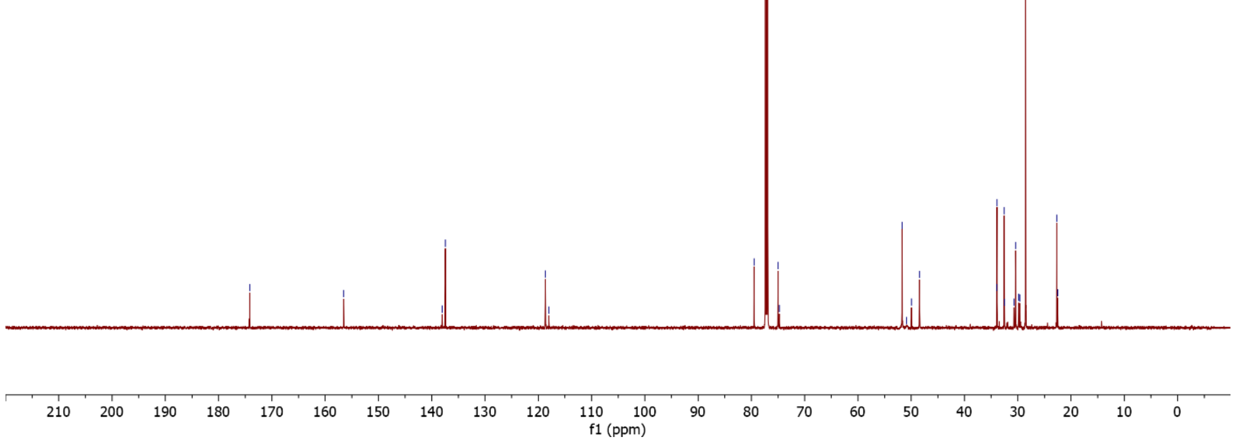

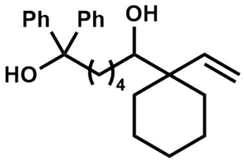

3x

^{1}H NMR, 600 MHz, $CDCl_3$

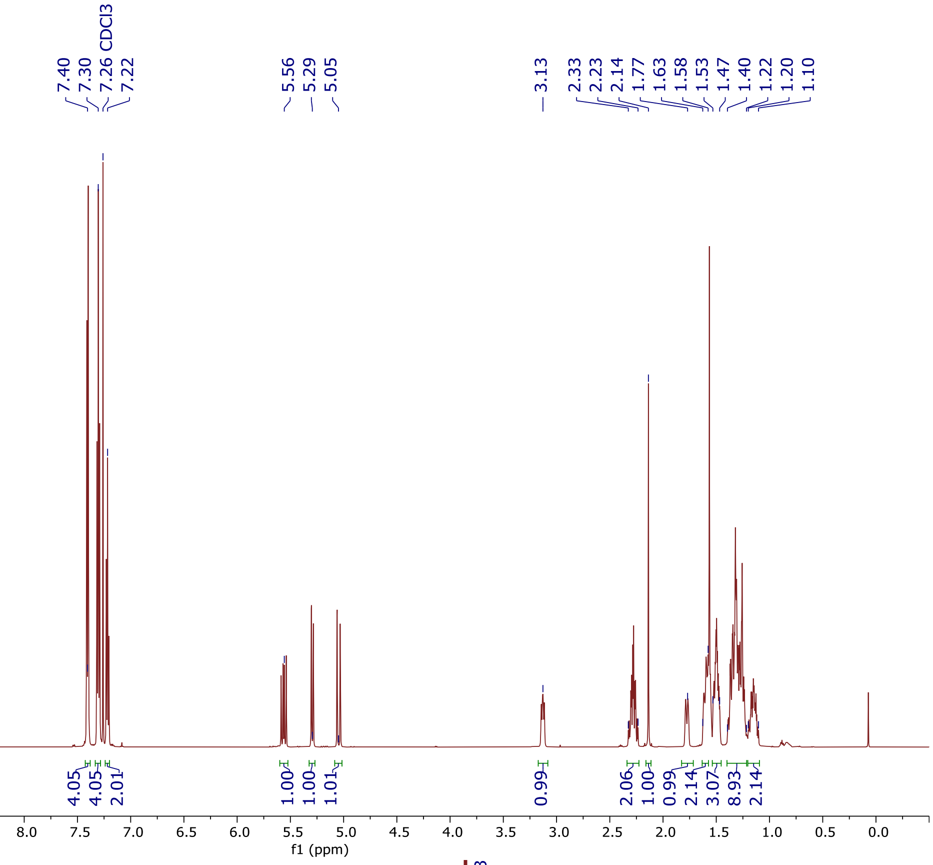

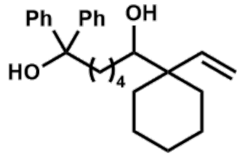

3x

^{13}C NMR, 151 MHz, $CDCl_3$

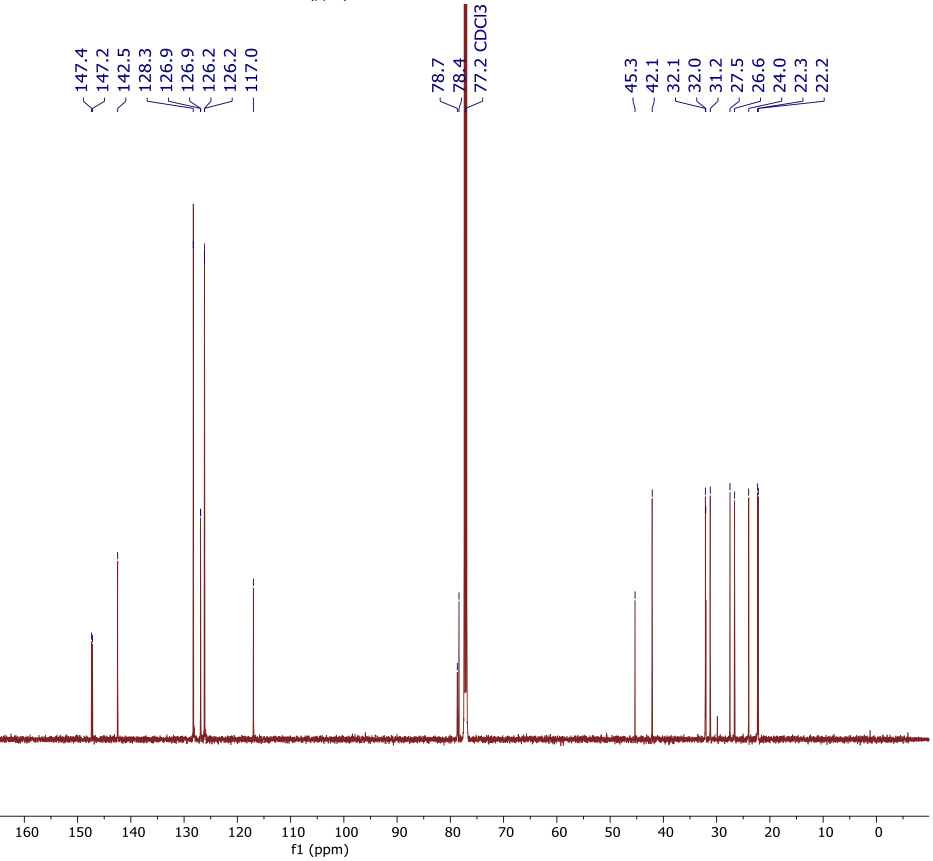

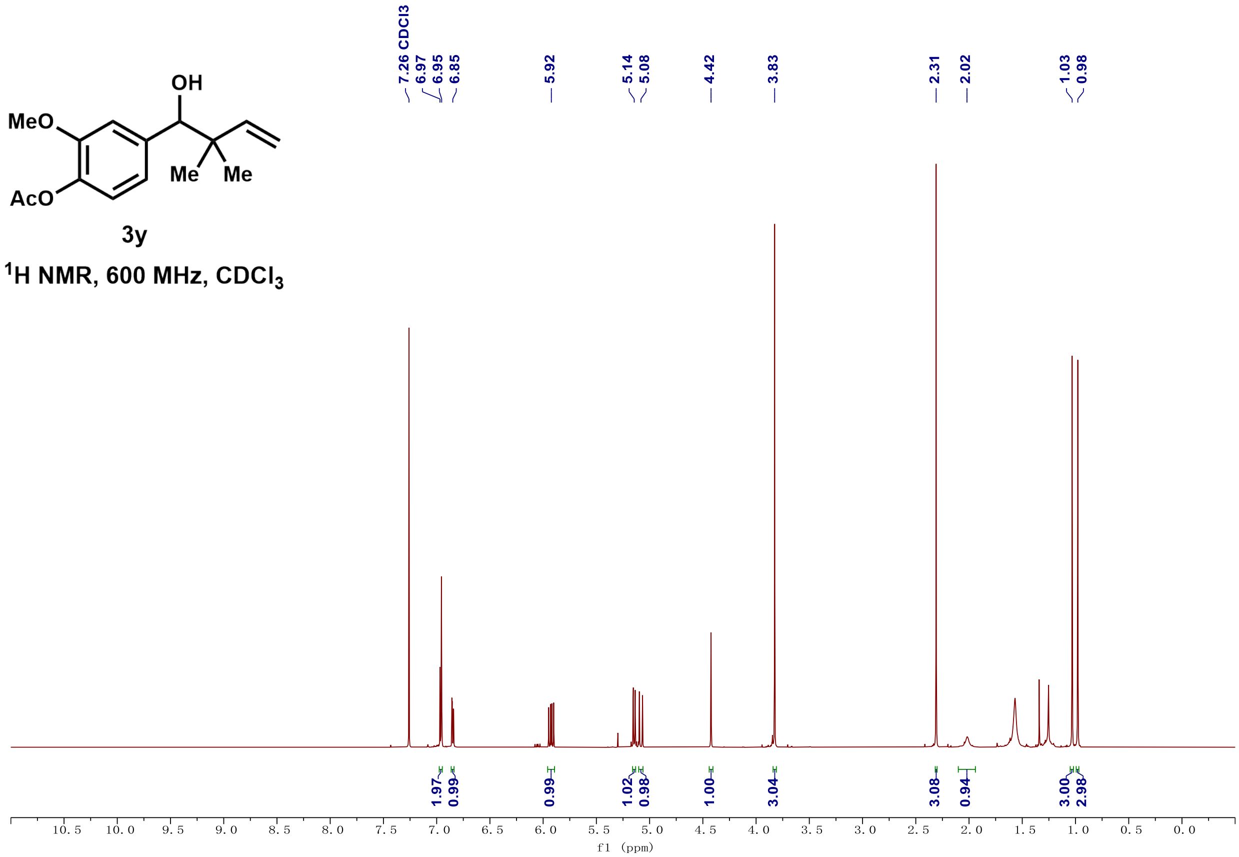
OH
MeO
AcO
Me
Me
3y
^{1}H NMR, 600 MHz, $CDCl_3$
7.26 CDCl3
6.97
6.95
6.85
5.92
5.14
5.08
4.42
3.83
2.31
2.02
1.03
0.98
1.97
0.99
0.99
1.02
0.98
1.00
3.04
3.08
0.94
3.00
2.98
10.5 10.0 9.5 9.0 8.5 8.0 7.5 7.0 6.5 6.0 5.5 5.0 4.5 4.0 3.5 3.0 2.5 2.0 1.5 1.0 0.5 0.0
f1 (ppm)

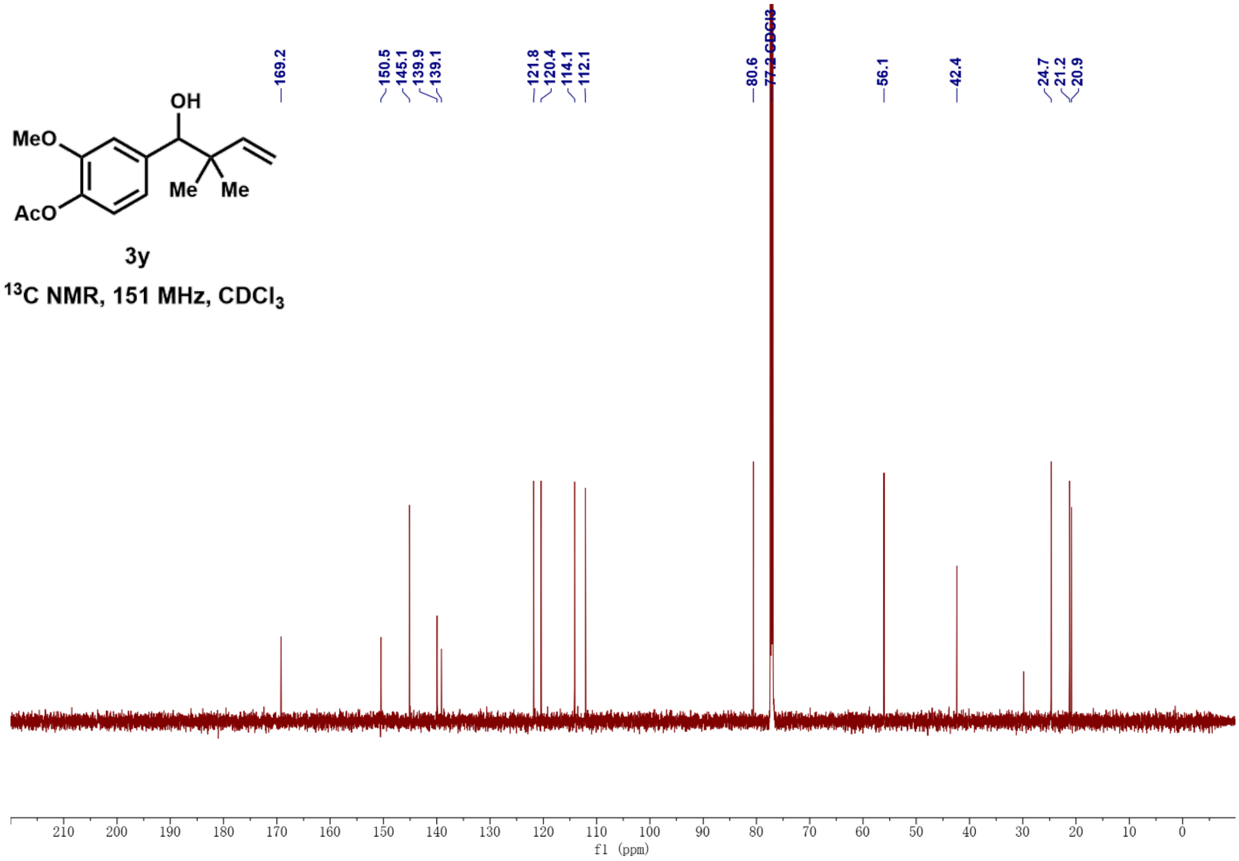
OH
MeO
AcO
Me
Me
3y
^{13}C NMR, 151 MHz, $CDCl_3$
169.2
150.5
145.1
139.9
139.1
121.8
120.4
114.1
112.1
80.6
77.2 CDCl3
56.1
42.4
24.7
21.2
20.9
210 200 190 180 170 160 150 140 130 120 110 100 90 80 70 60 50 40 30 20 10 0
f1 (ppm)

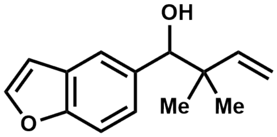

3z

^{1}H NMR, 600 MHz, $CDCl_3$

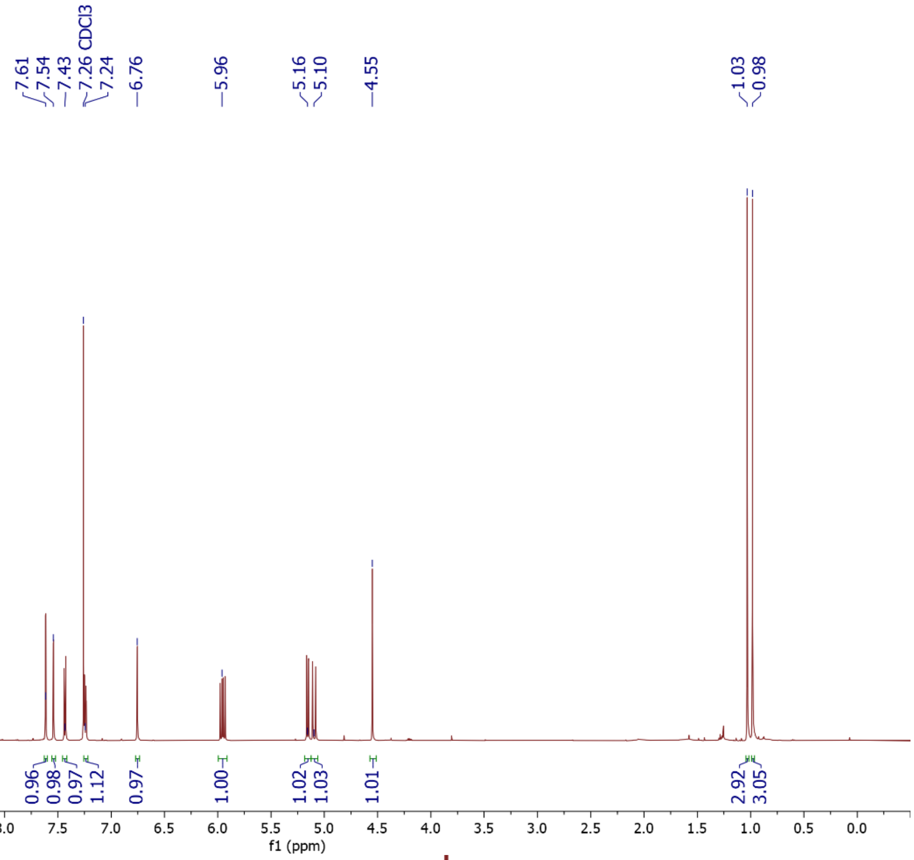

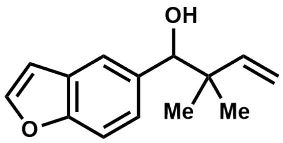

3z

^{13}C NMR, 151 MHz, $CDCl_3$

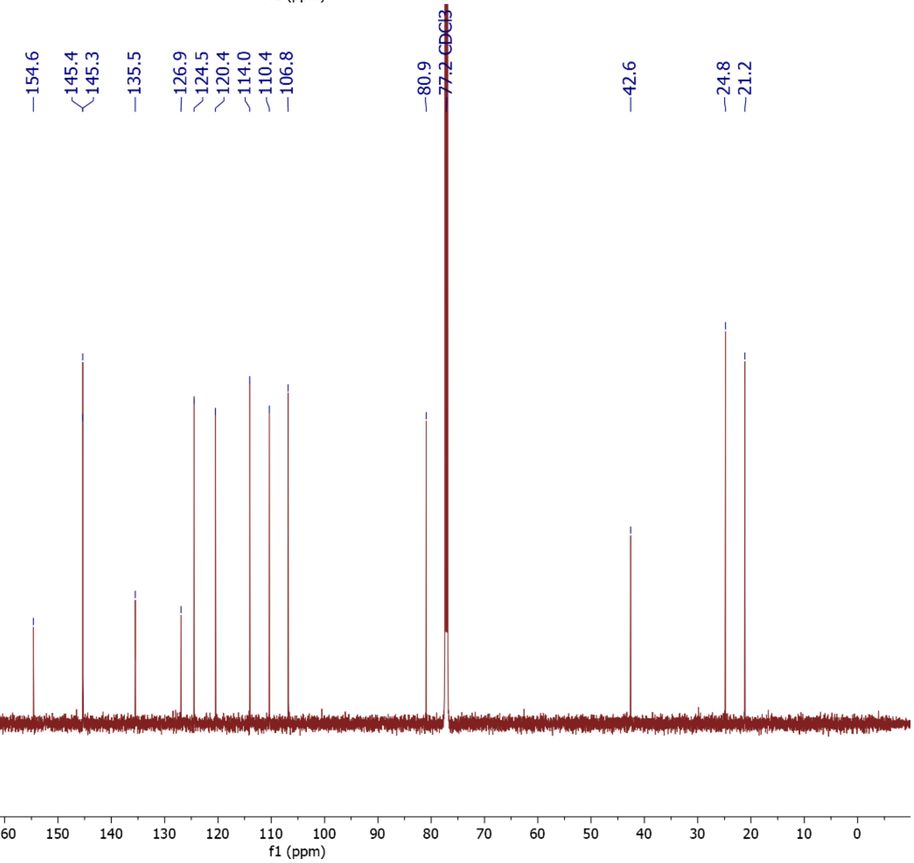

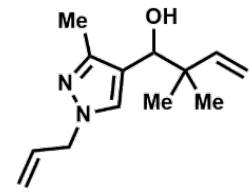

3aa

^1H NMR, 600 MHz, $CDCl_3$

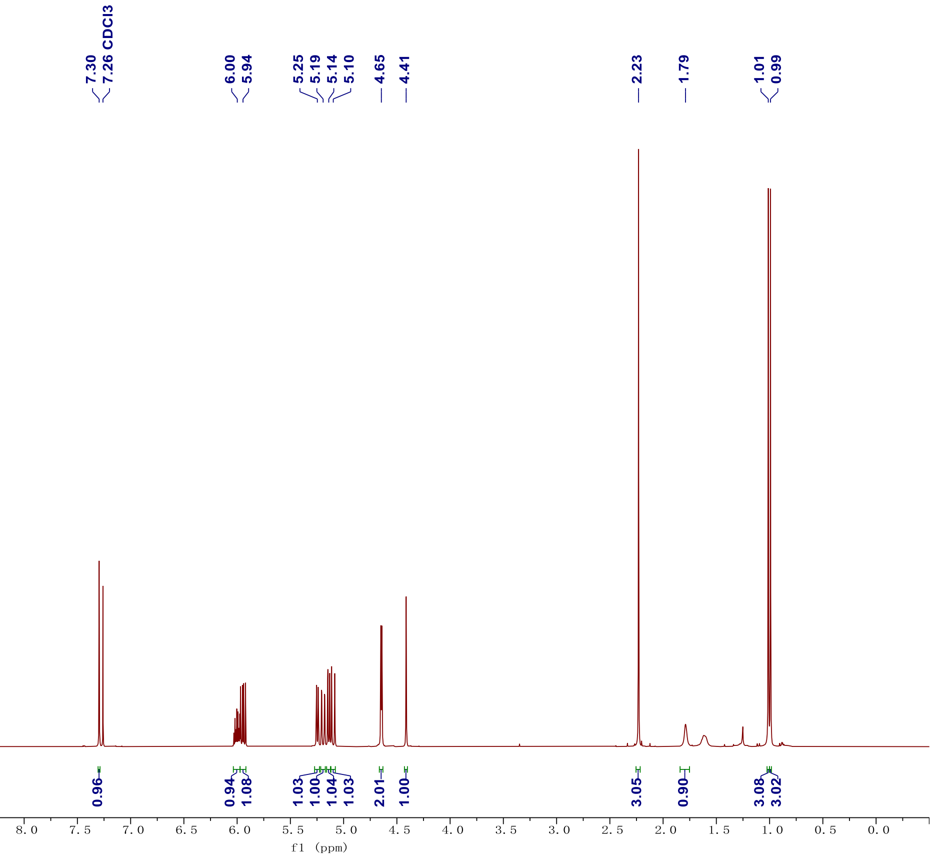

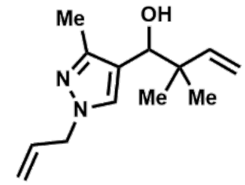

3aa

^{13}C NMR, 151 MHz, $CDCl_3$

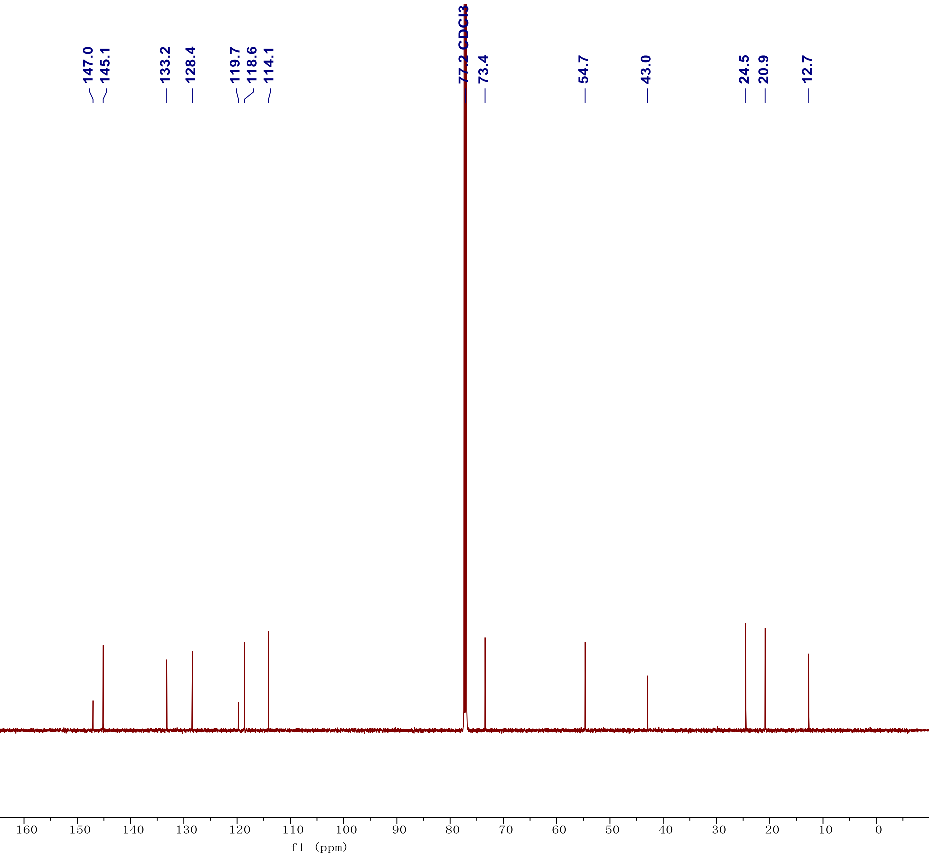

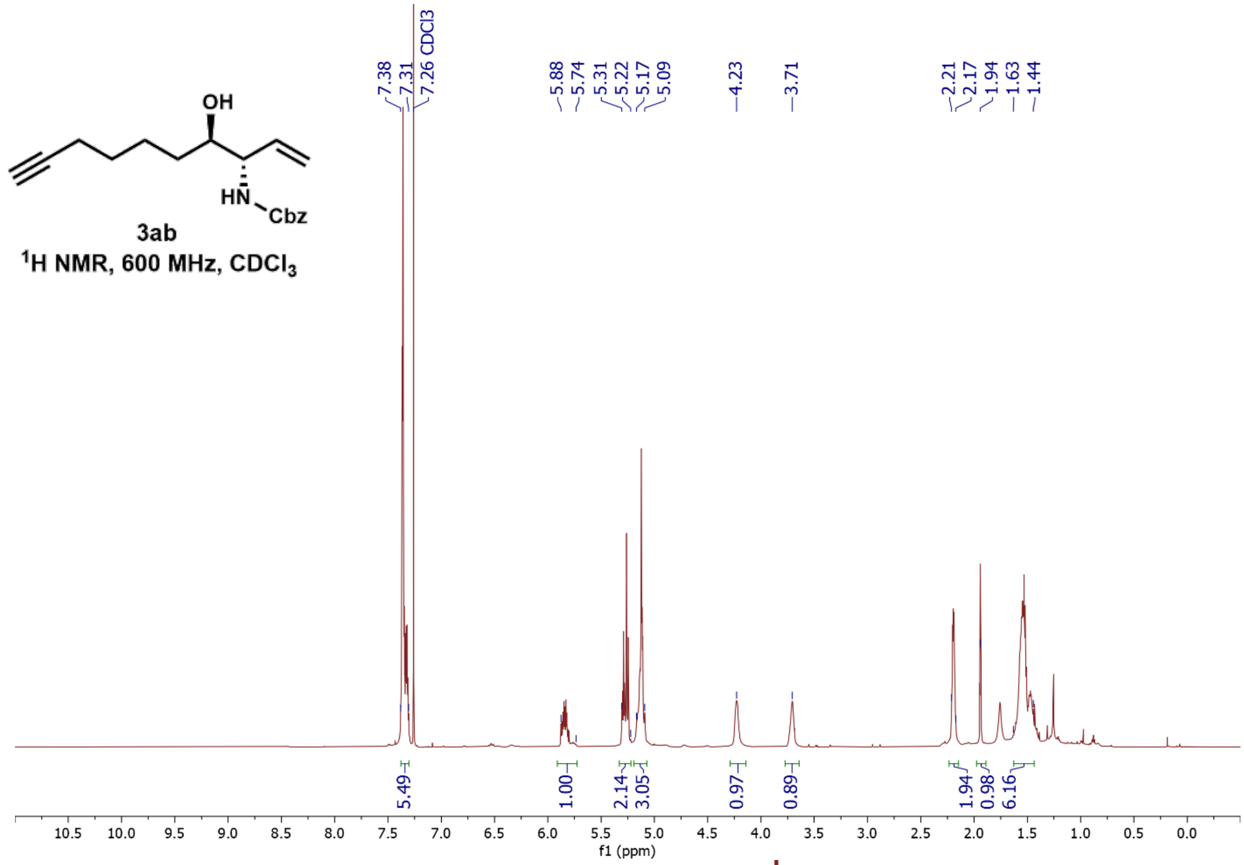
OH
HN
Cbz
3ab
1H NMR, 600 MHz, CDCl3
7.38
7.31
7.26 CDCl3
5.88
5.74
5.31
5.22
5.17
5.09
4.23
3.71
2.21
2.17
1.94
1.63
1.44
5.49
1.00
2.14
3.05
0.97
0.89
1.94
0.98
6.16
f1 (ppm)

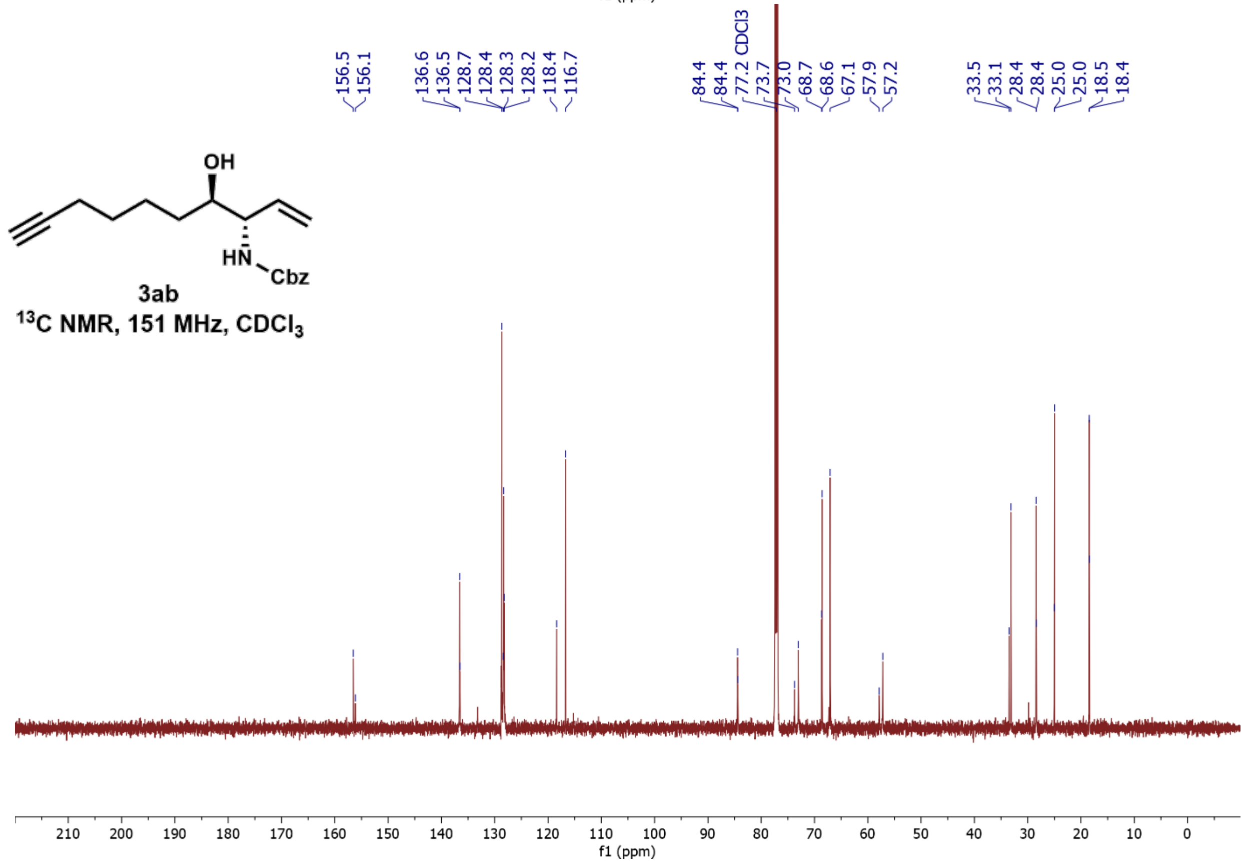
OH
HN
Cbz
3ab
13C NMR, 151 MHz, CDCl3
156.5
156.1
136.6
136.5
128.7
128.4
128.3
128.2
118.4
116.7
84.4
84.4
77.2 CDCl3
73.7
73.0
68.7
68.6
67.1
57.9
57.2
33.5
33.1
28.4
28.4
25.0
25.0
18.5
18.4
f1 (ppm)

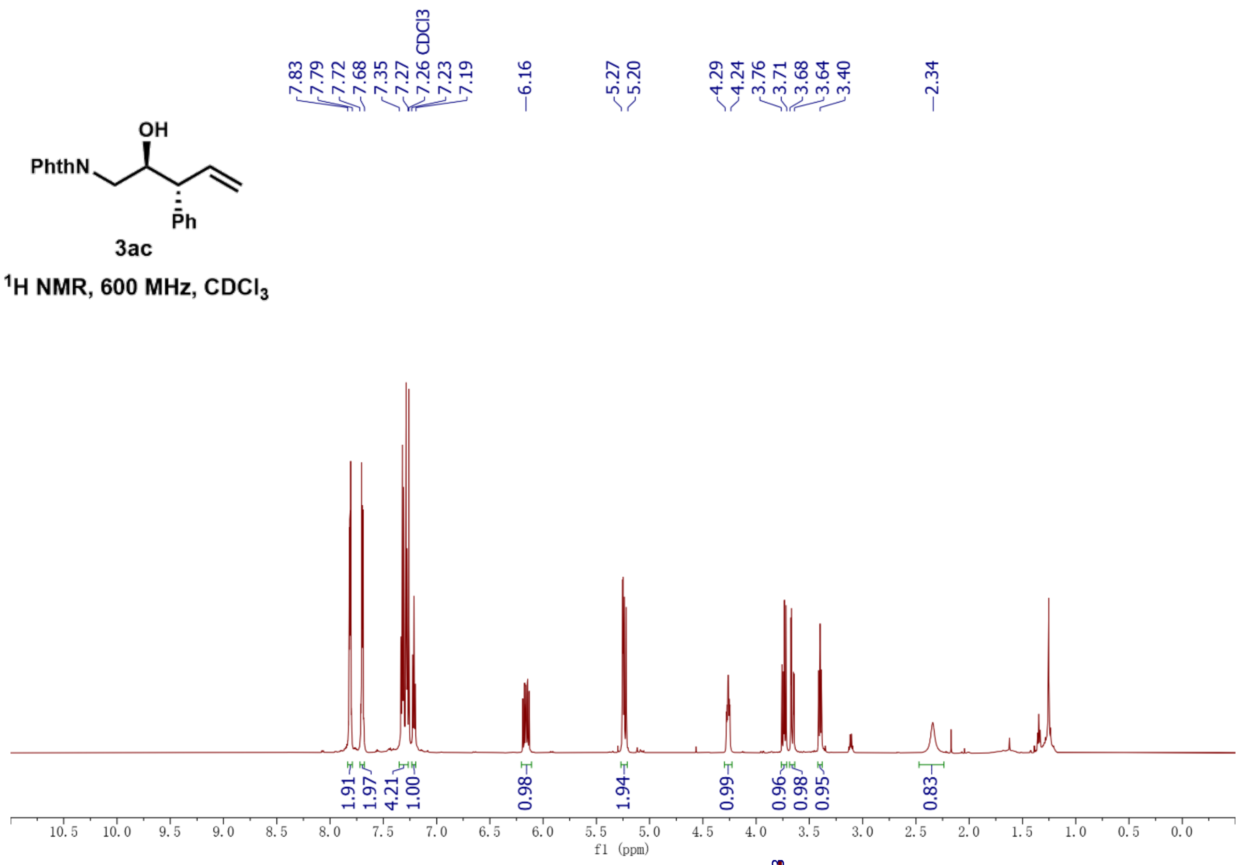
PhthN
OH
Ph
3ac
^{1}H NMR, 600 MHz, CDCl$_3$
7.83
7.79
7.72
7.68
7.35
7.27
7.26 CDCl3
7.23
7.19
6.16
5.27
5.20
4.29
4.24
3.76
3.71
3.68
3.64
3.40
2.34
1.91
1.97
4.21
1.00
0.98
1.94
0.99
0.96
0.98
0.95
0.83
10.5 10.0 9.5 9.0 8.5 8.0 7.5 7.0 6.5 6.0 5.5 5.0 4.5 4.0 3.5 3.0 2.5 2.0 1.5 1.0 0.5 0.0
f1 (ppm)

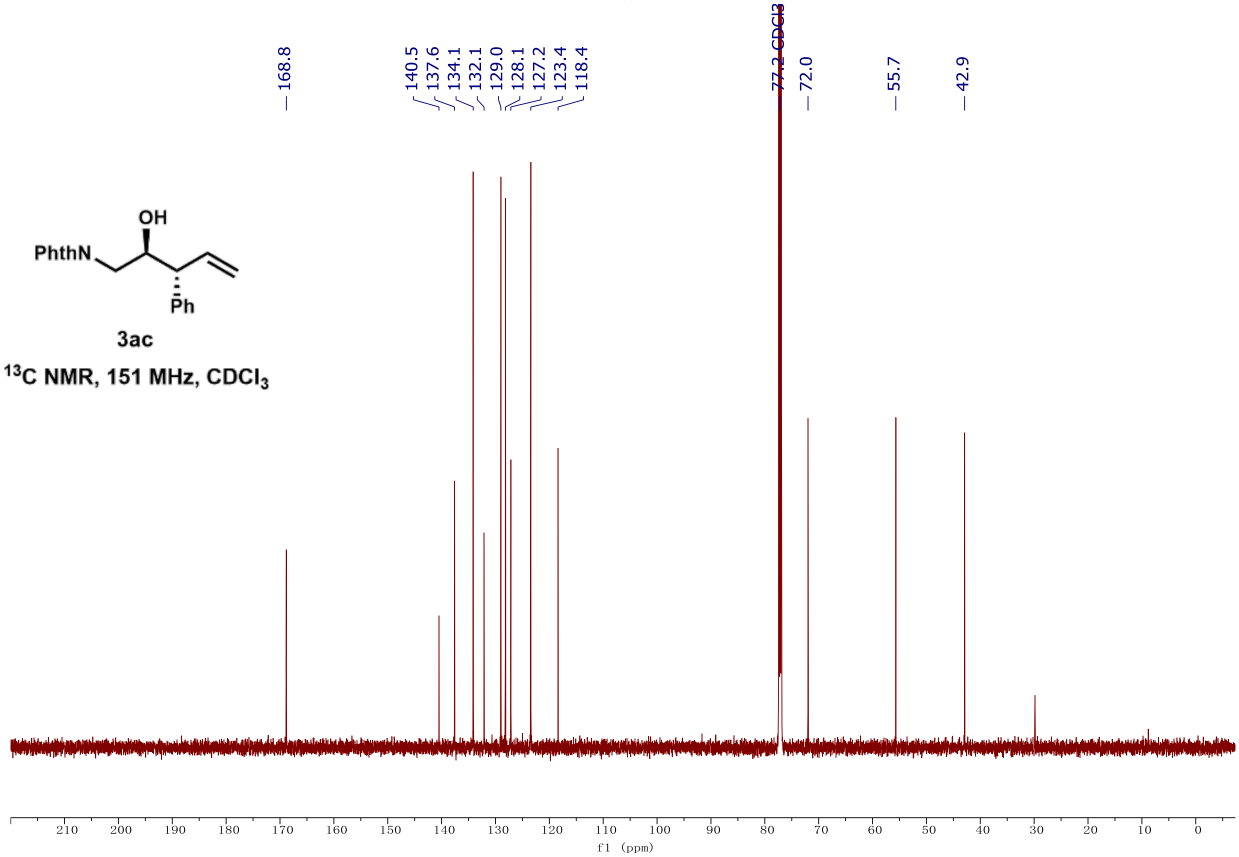
PhthN
OH
Ph
3ac
^{13}C NMR, 151 MHz, CDCl$_3$
168.8
140.5
137.6
134.1
132.1
129.0
128.1
127.2
123.4
118.4
77.2 CDCl3
72.0
55.7
42.9
210 200 190 180 170 160 150 140 130 120 110 100 90 80 70 60 50 40 30 20 10 0
f1 (ppm)

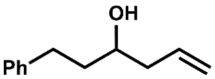

3ad

^{1}H NMR, 600 MHz, $CDCl_3$

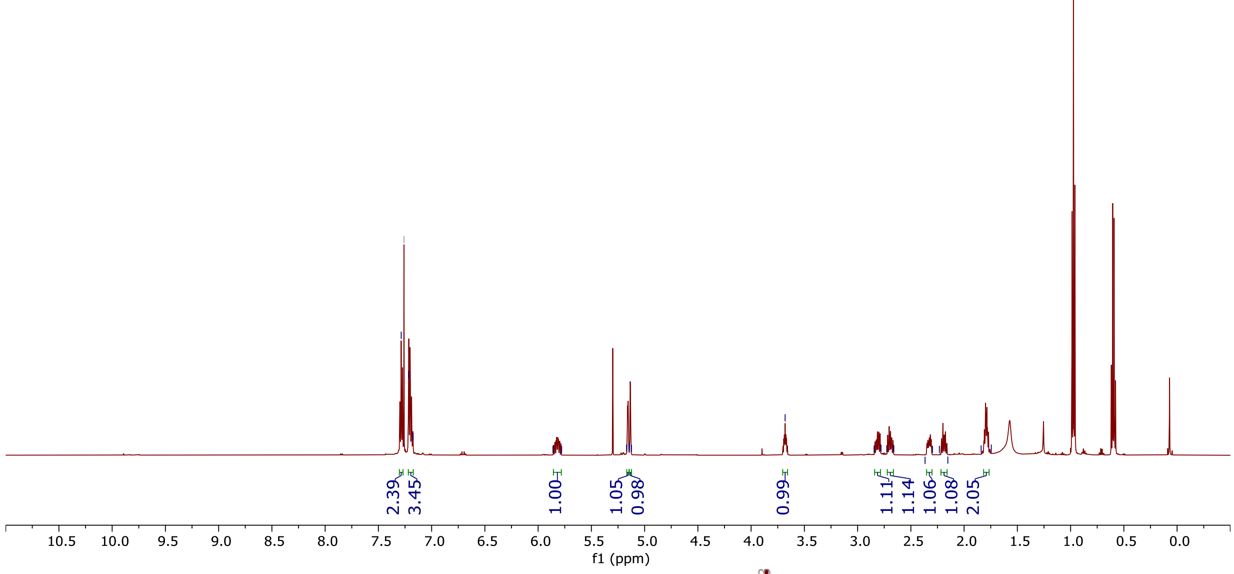

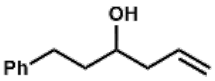

3ad

^{13}C NMR, 151 MHz, $CDCl_3$

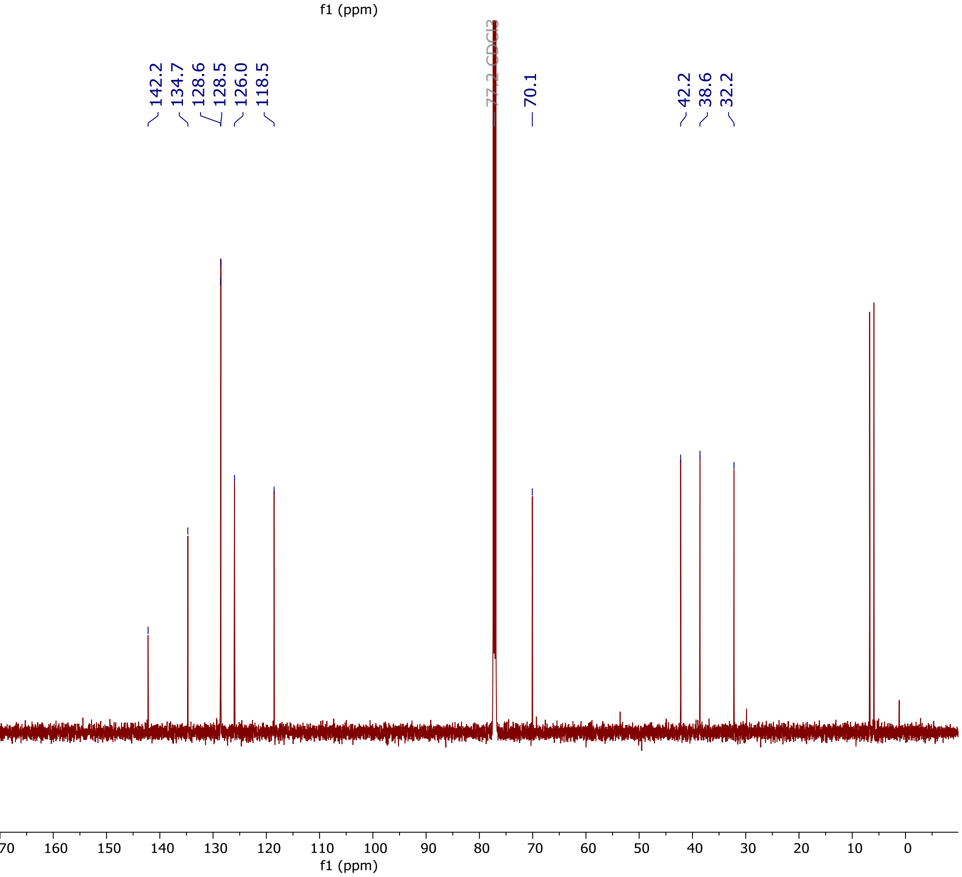

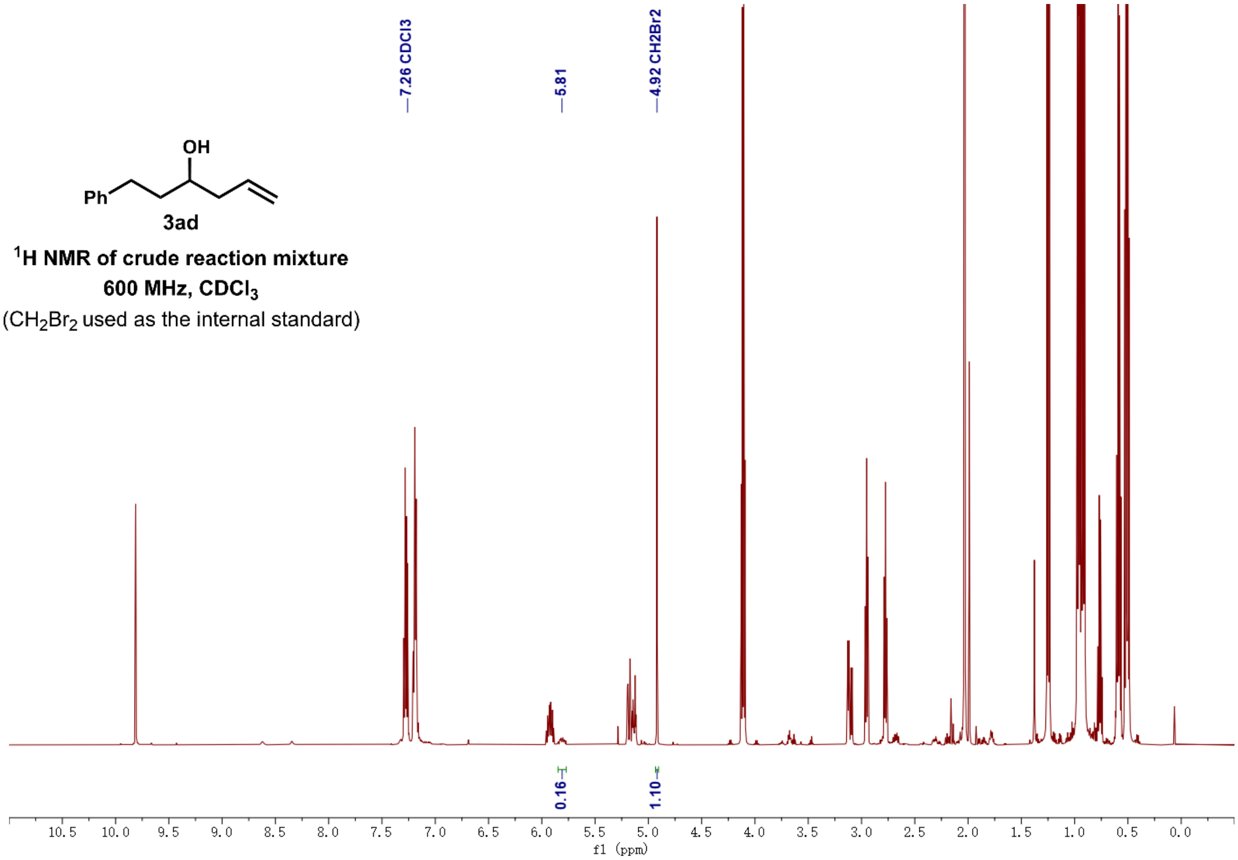

3ad

^{1}H NMR of crude reaction mixture

600 MHz, $CDCl_3$

(CH_2Br_2 used as the internal standard)

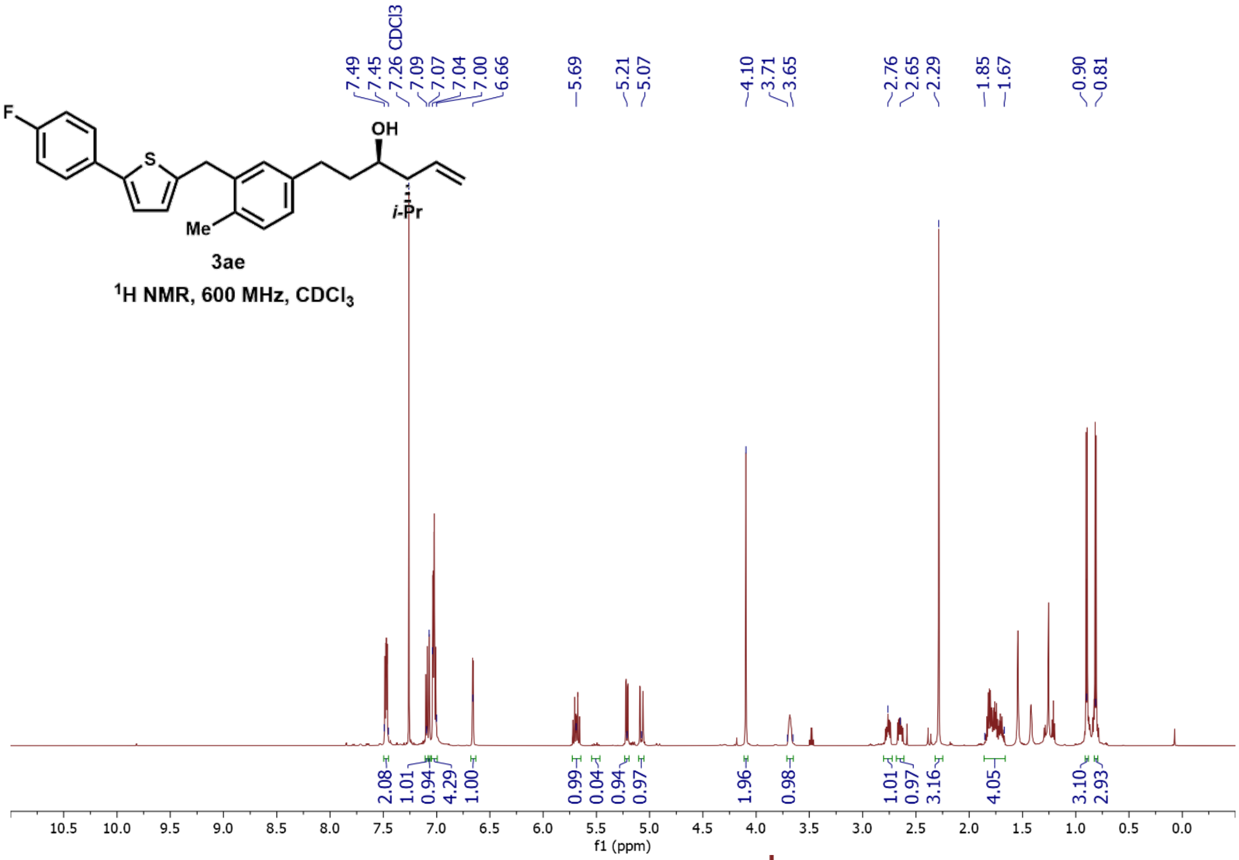

3ae

^{1}H NMR, 600 MHz, $CDCl_3$

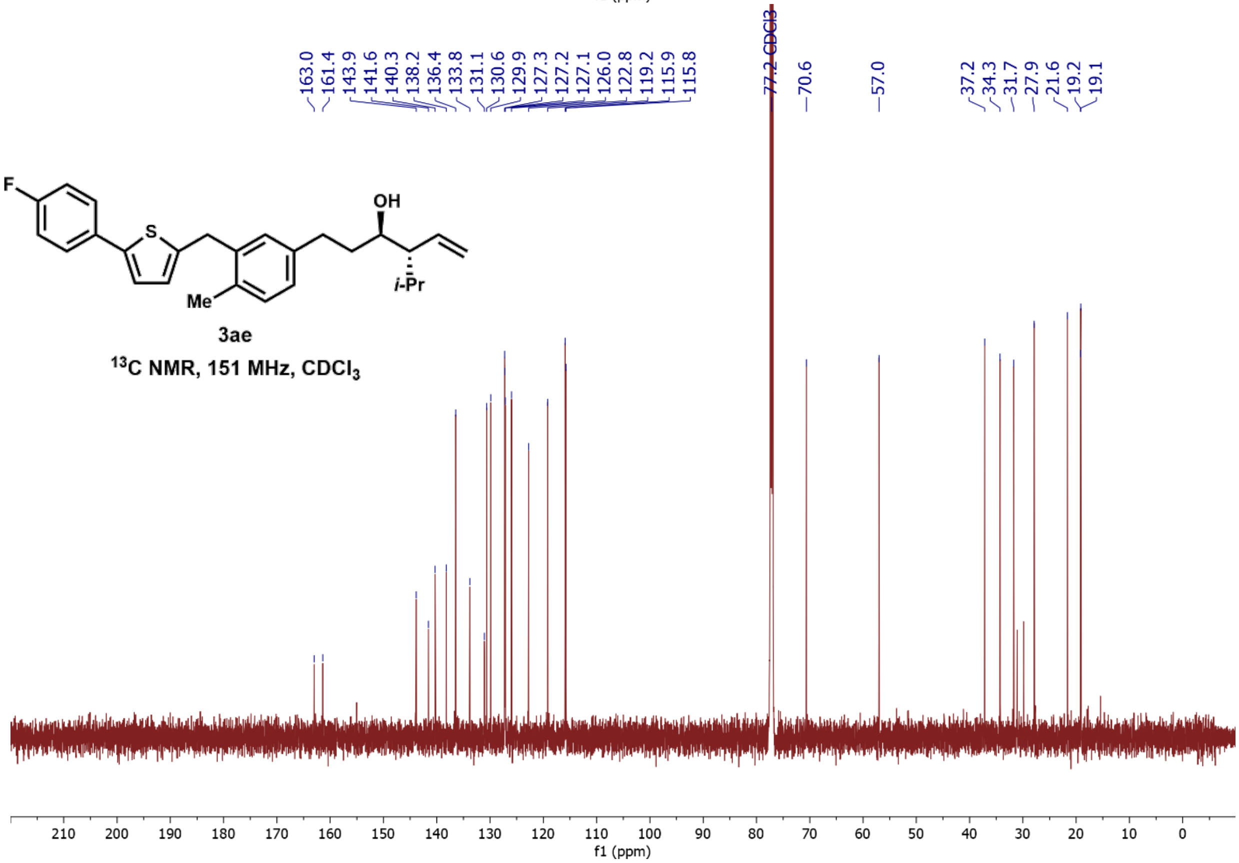

3ae

^{13}C NMR, 151 MHz, $CDCl_3$

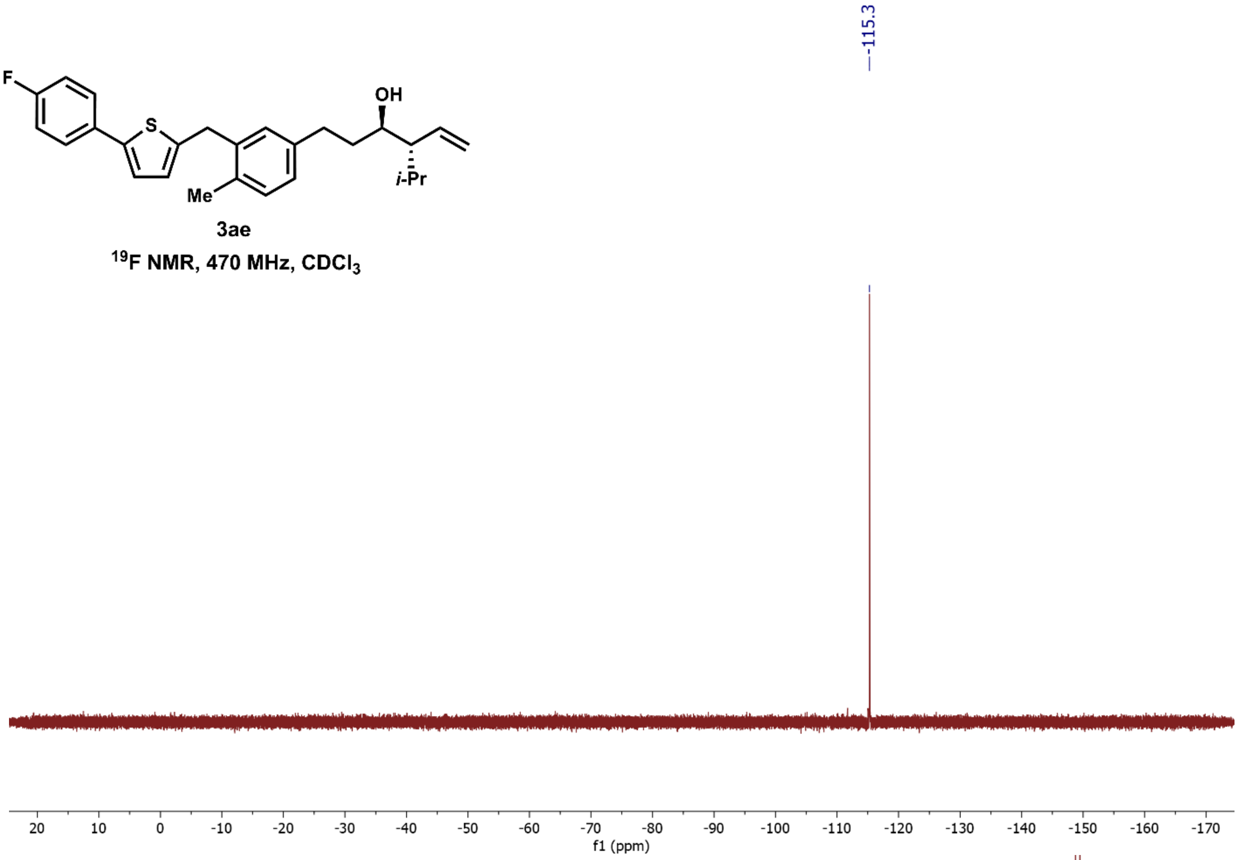

3ae

^{19}F NMR, 470 MHz, CDCl$_3$

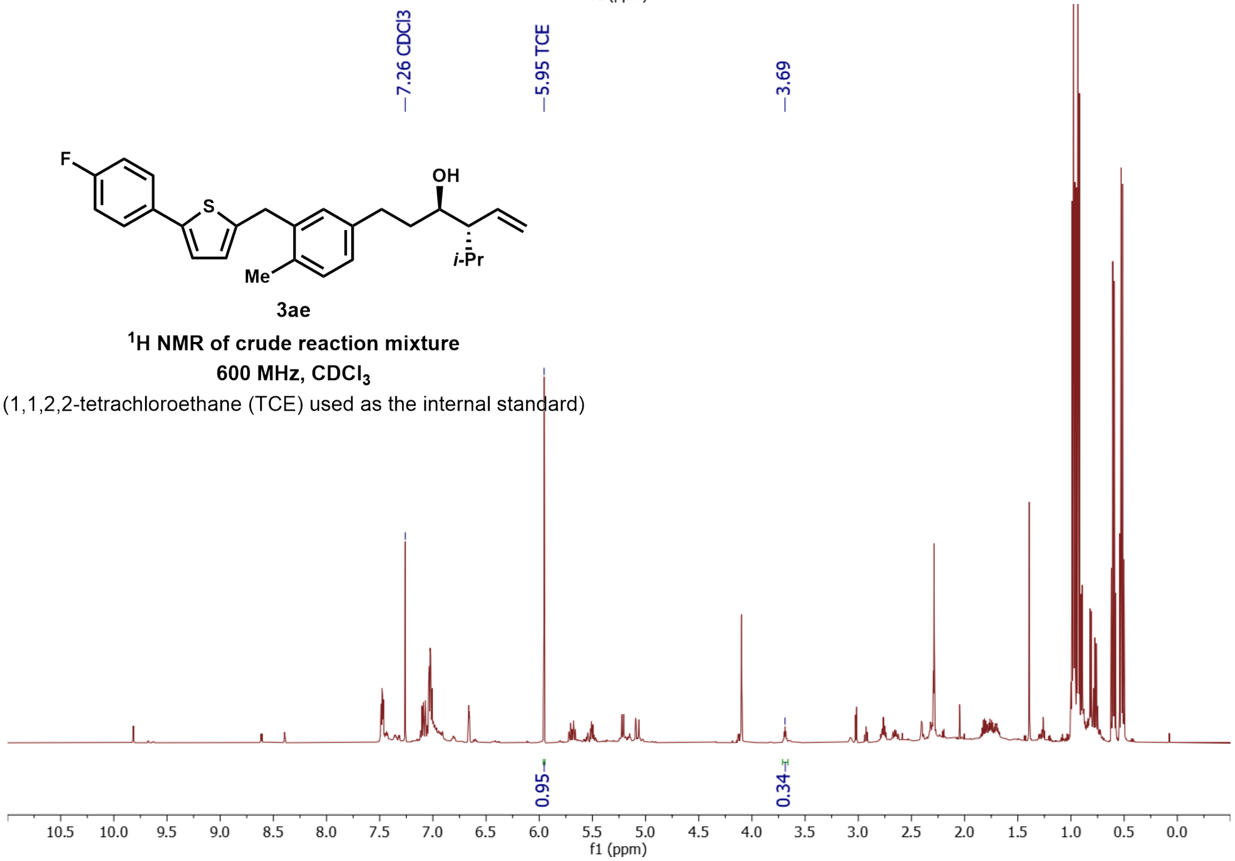

3ae

^{1}H NMR of crude reaction mixture

600 MHz, CDCl$_3$

(1,1,2,2-tetrachloroethane (TCE) used as the internal standard)

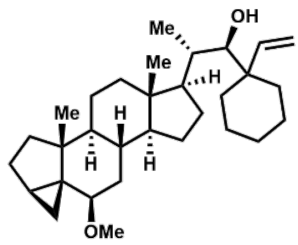

3af

^{1}H NMR, 600 MHz, $CDCl_3$

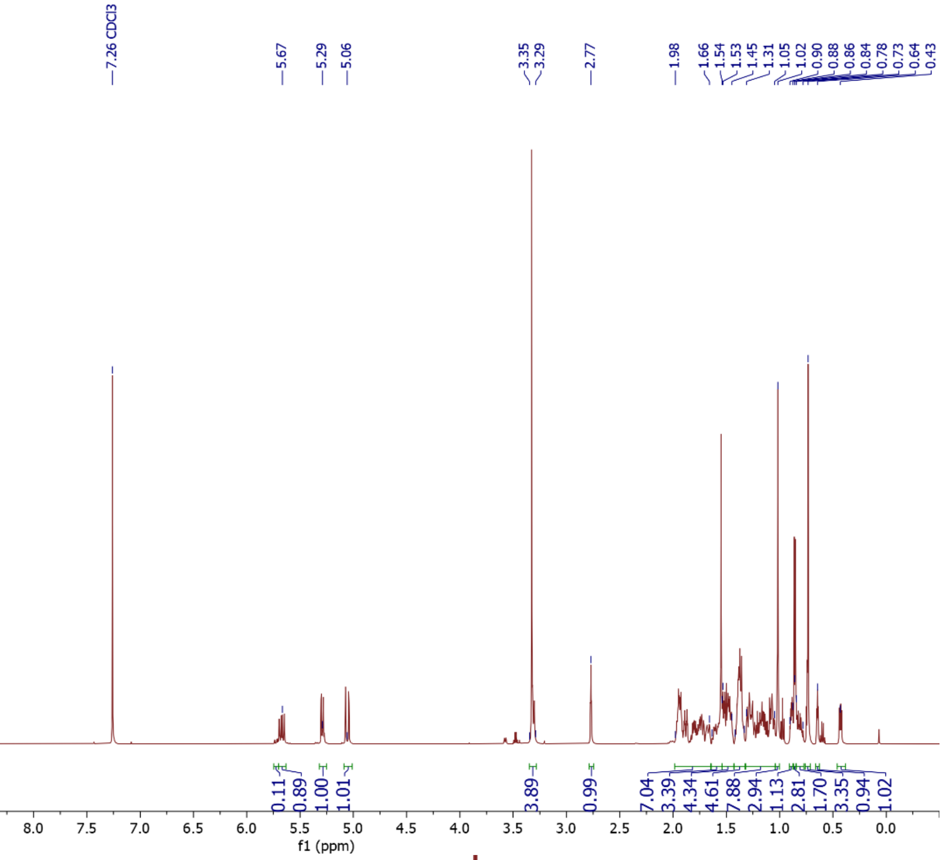

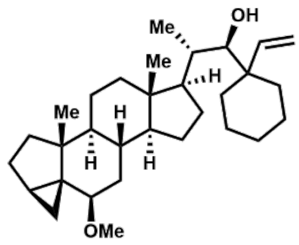

3af

^{13}C NMR, 151 MHz, $CDCl_3$

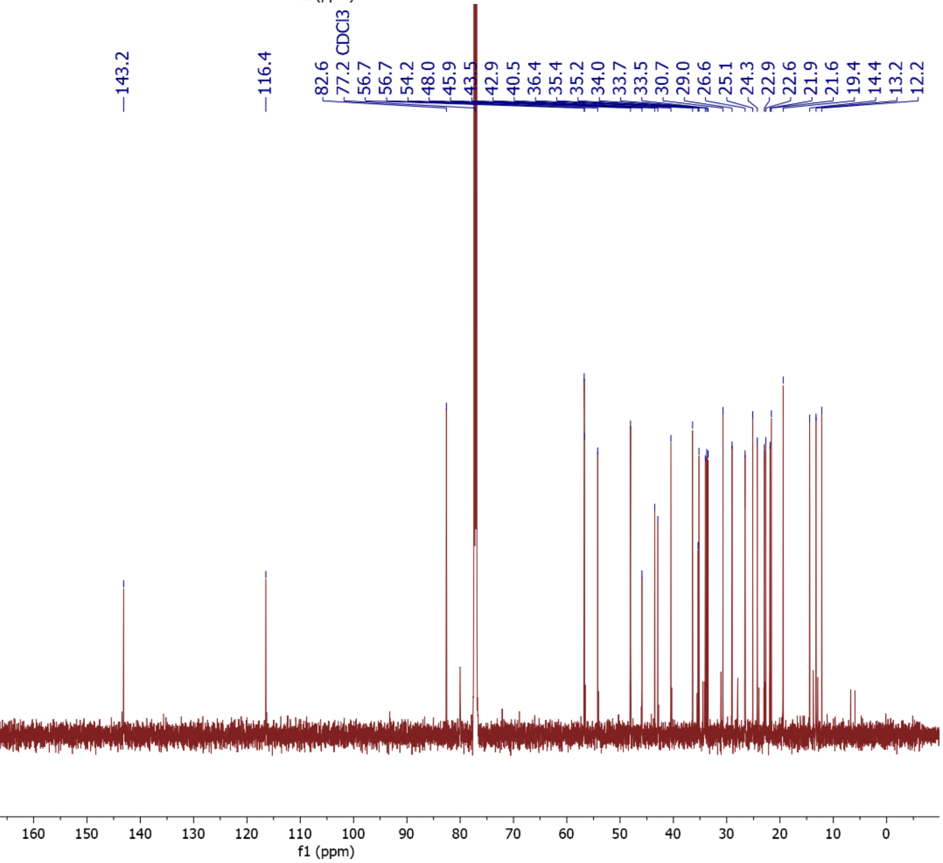

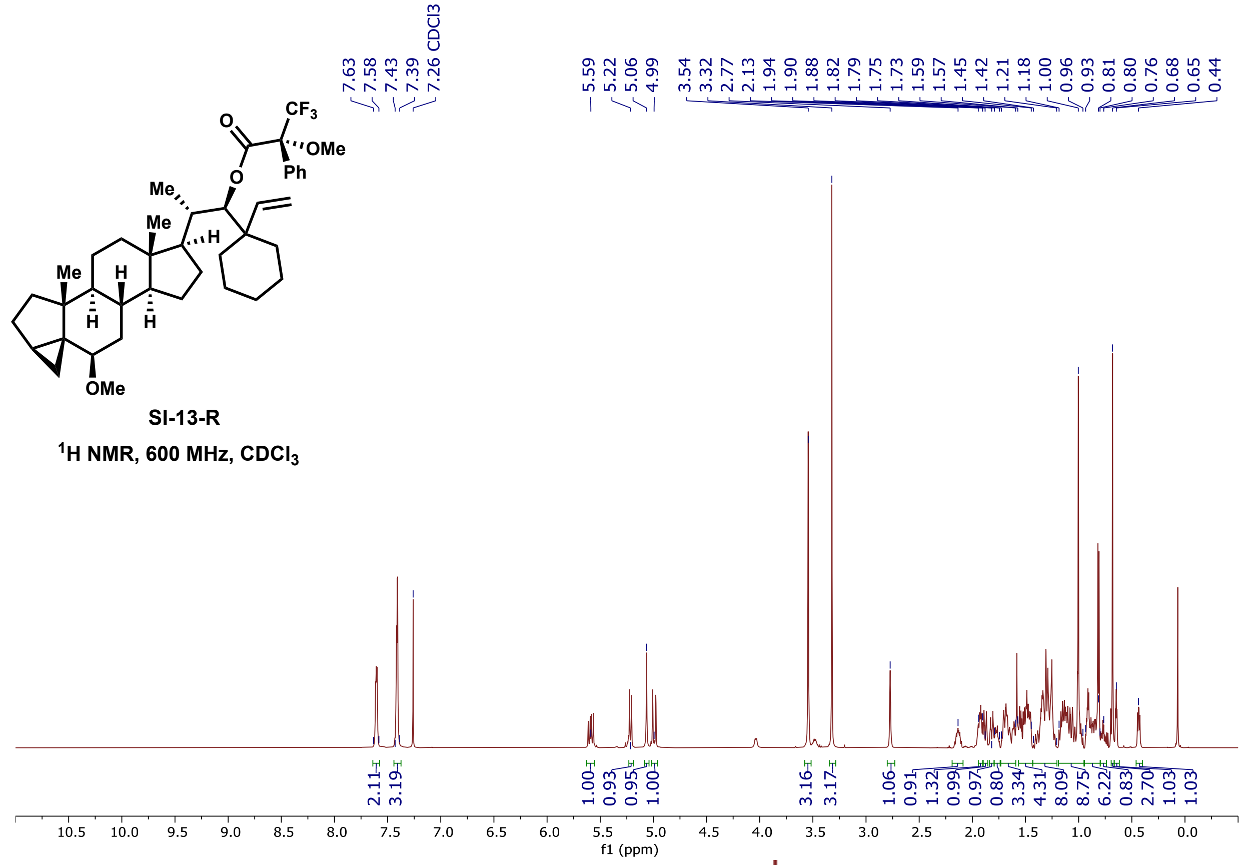

SI-13-R

^{1}H NMR, 600 MHz, $CDCl_3$

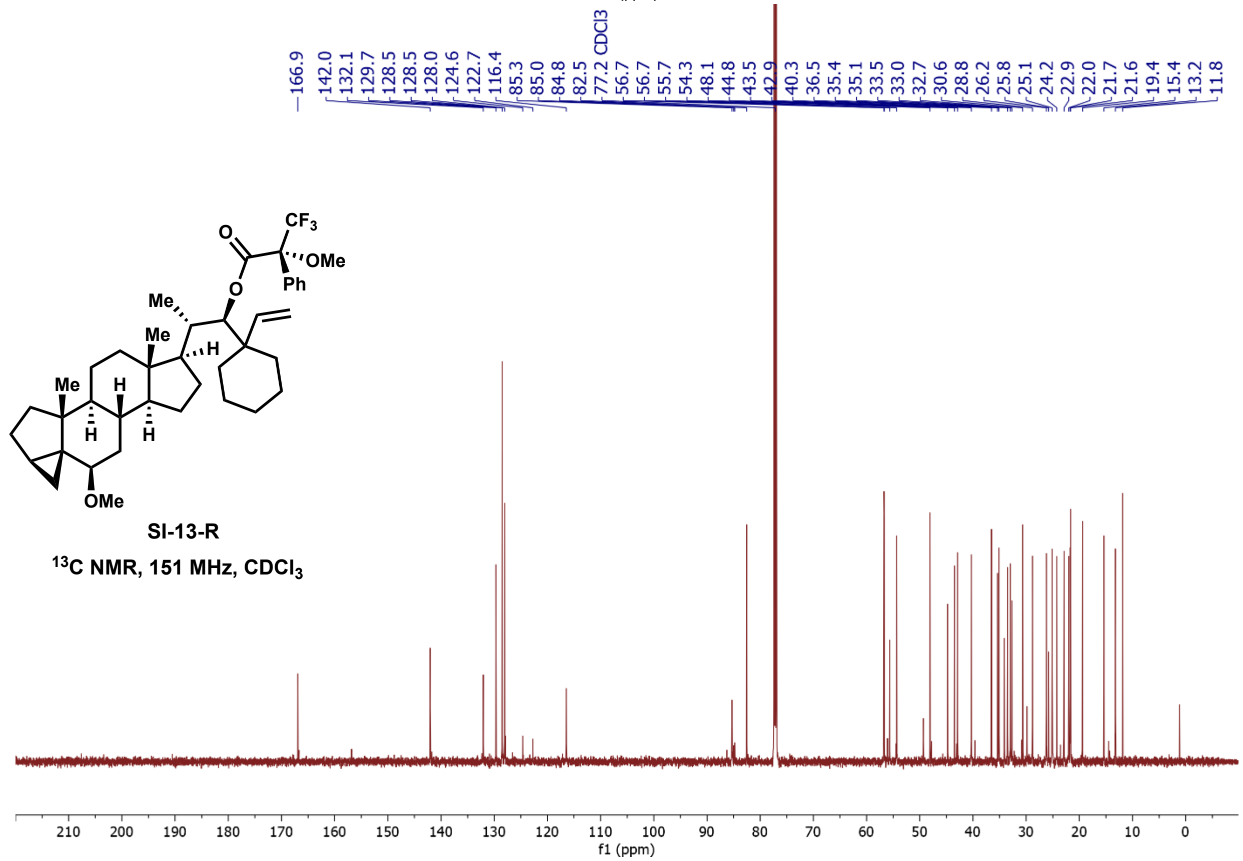

SI-13-R

^{13}C NMR, 151 MHz, $CDCl_3$

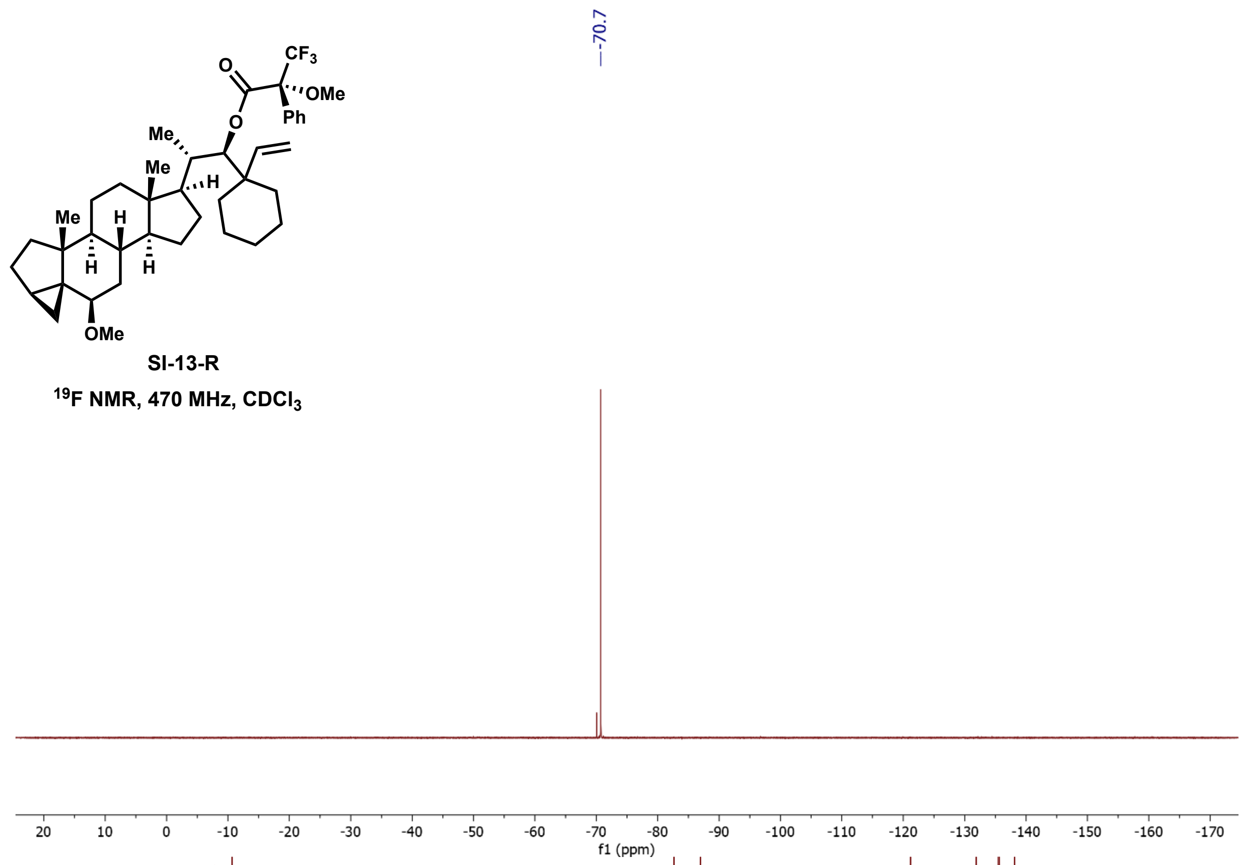

SI-13-R

^{19}F NMR, 470 MHz, $CDCl_3$

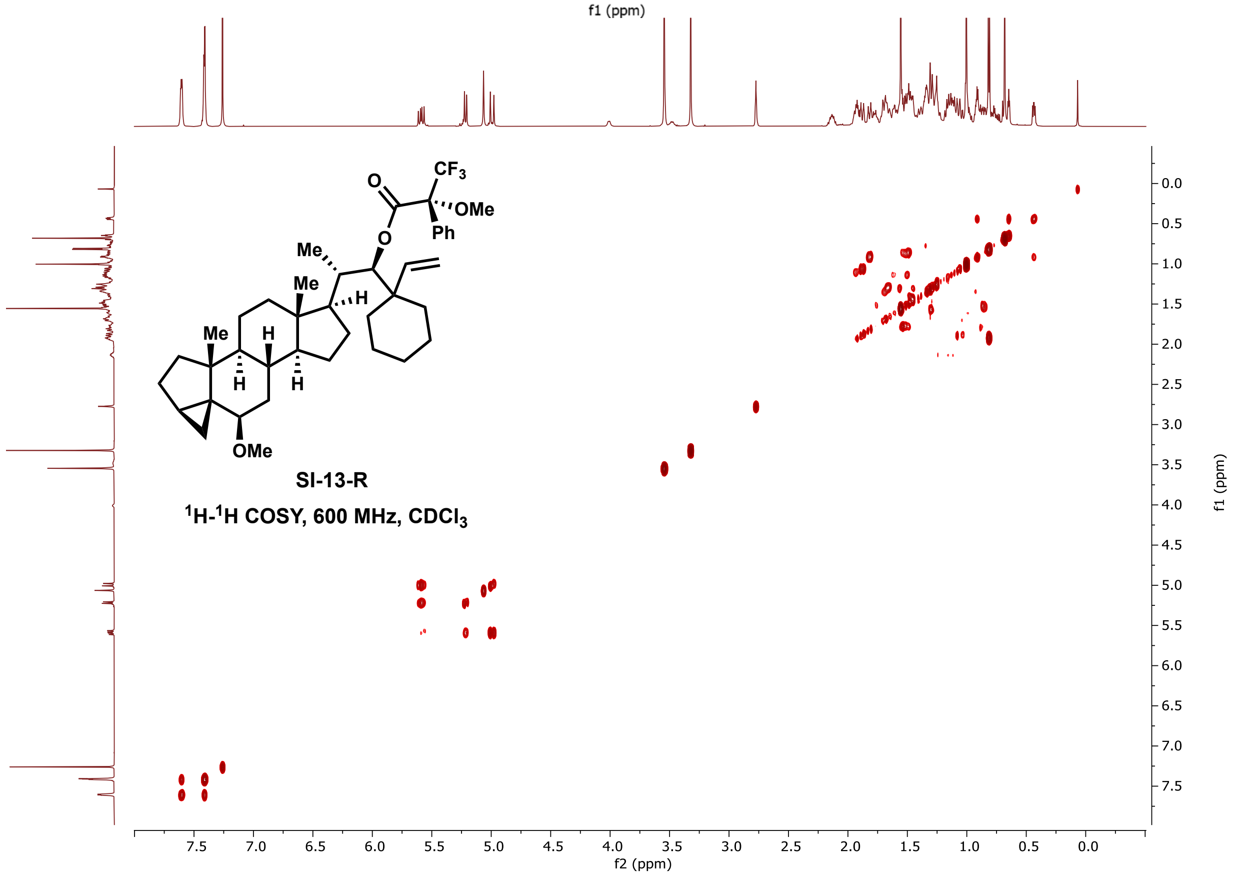

SI-13-R

^{1}H-^{1}H COSY, 600 MHz, $CDCl_3$

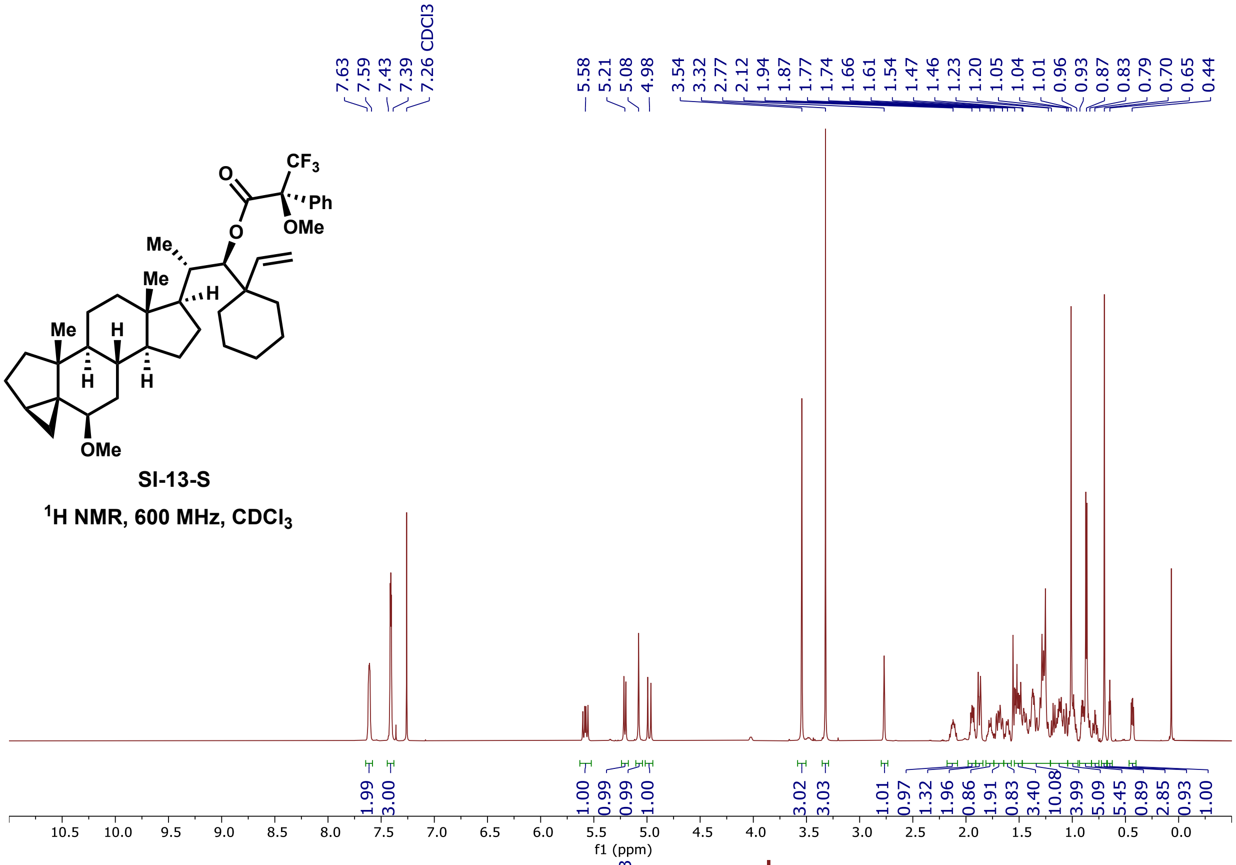

SI-13-S

^{1}H NMR, 600 MHz, $CDCl_3$

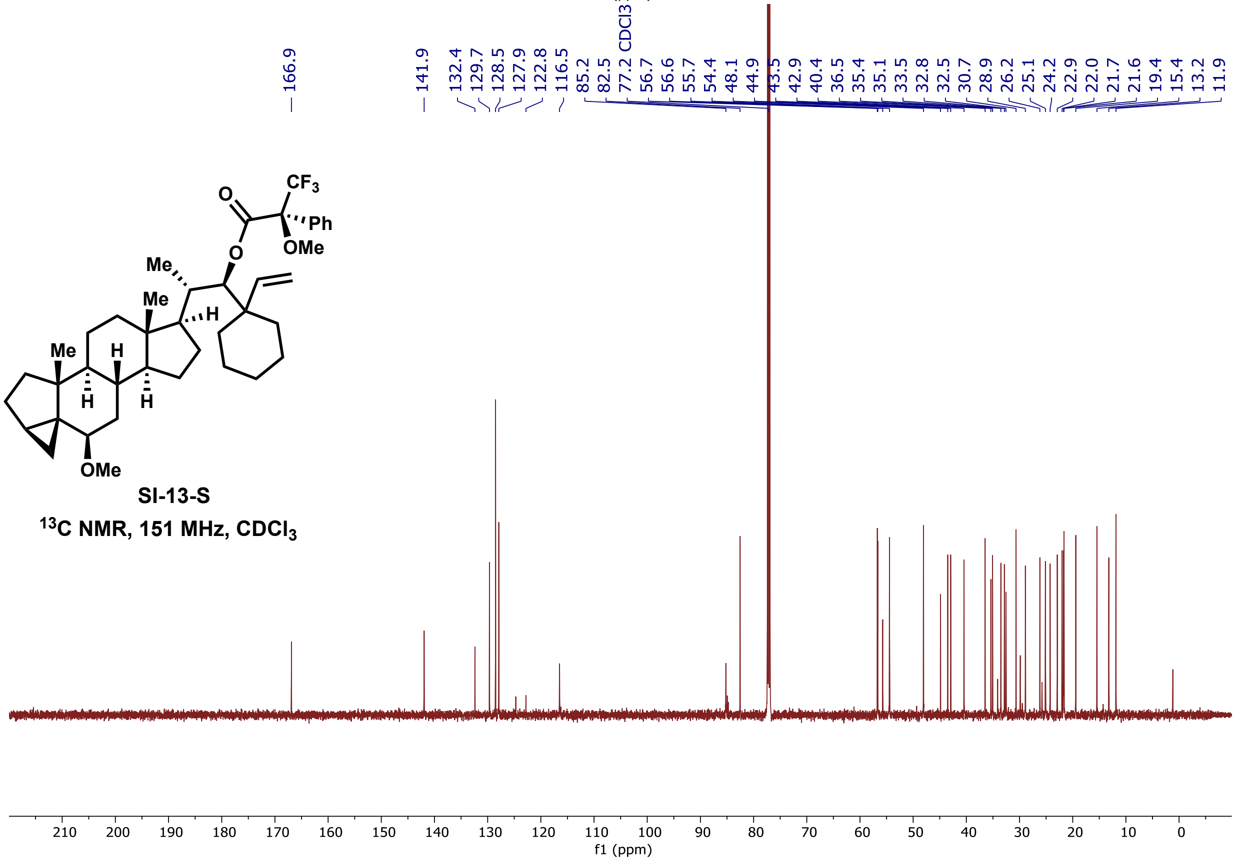

SI-13-S

^{13}C NMR, 151 MHz, $CDCl_3$

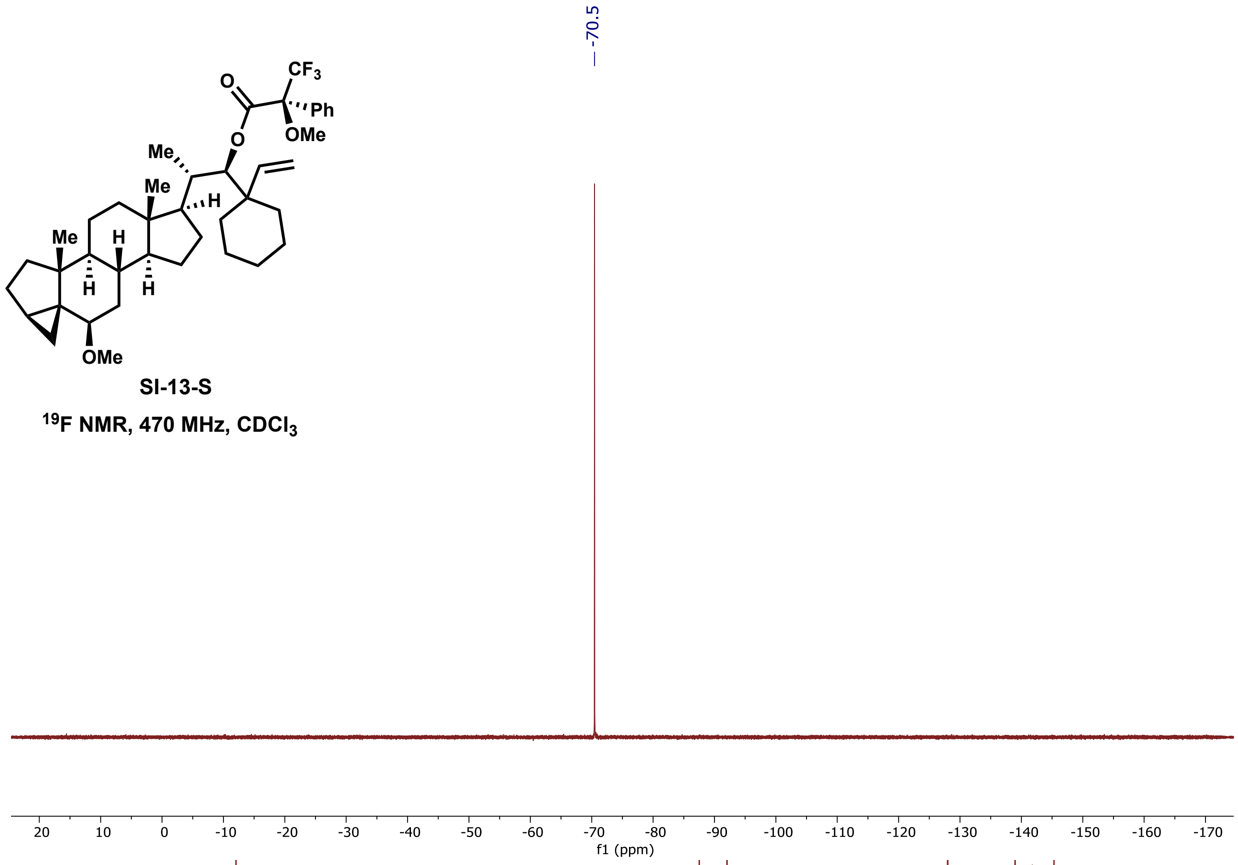
SI-13-S
^{19}F NMR, 470 MHz, CDCl$_3$
-70.5
f1 (ppm)

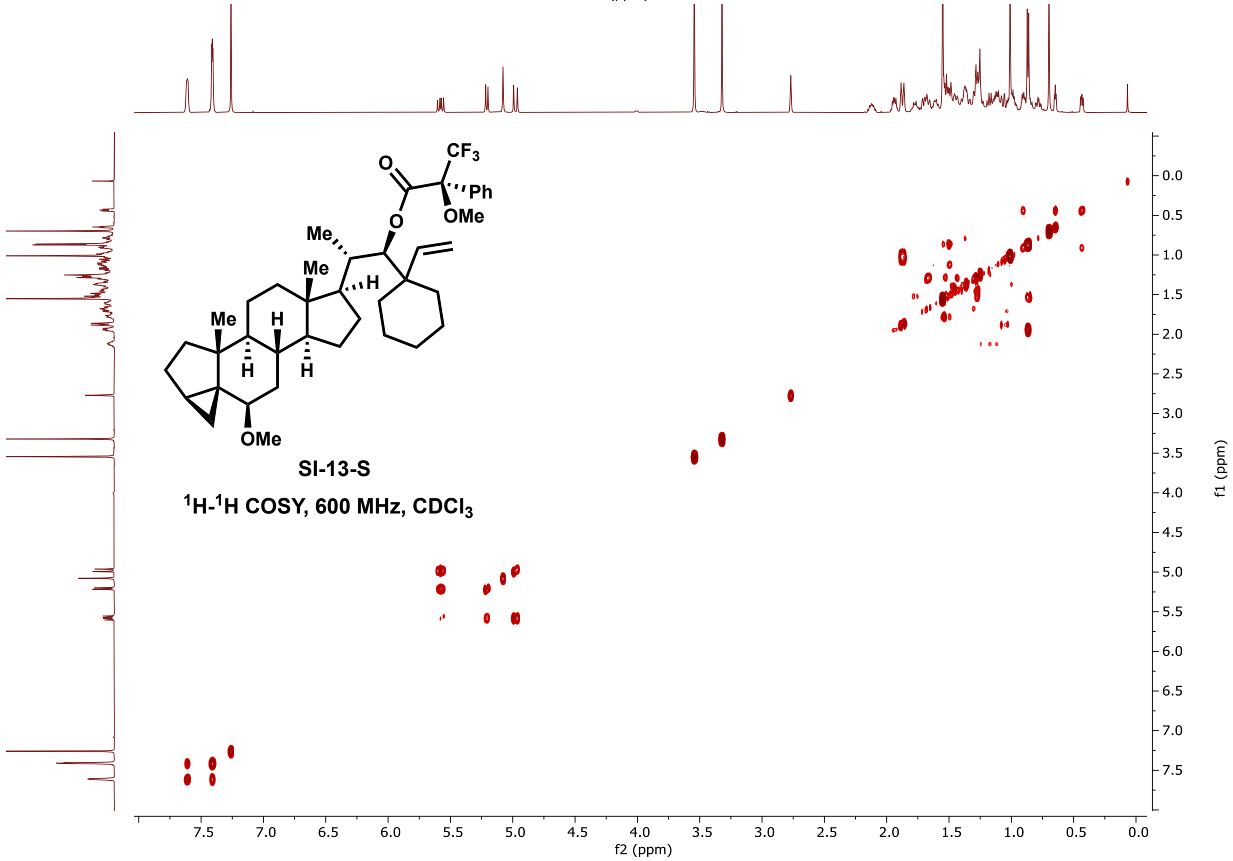
SI-13-S
^{1}H-^{1}H COSY, 600 MHz, CDCl$_3$
f2 (ppm)
f1 (ppm)

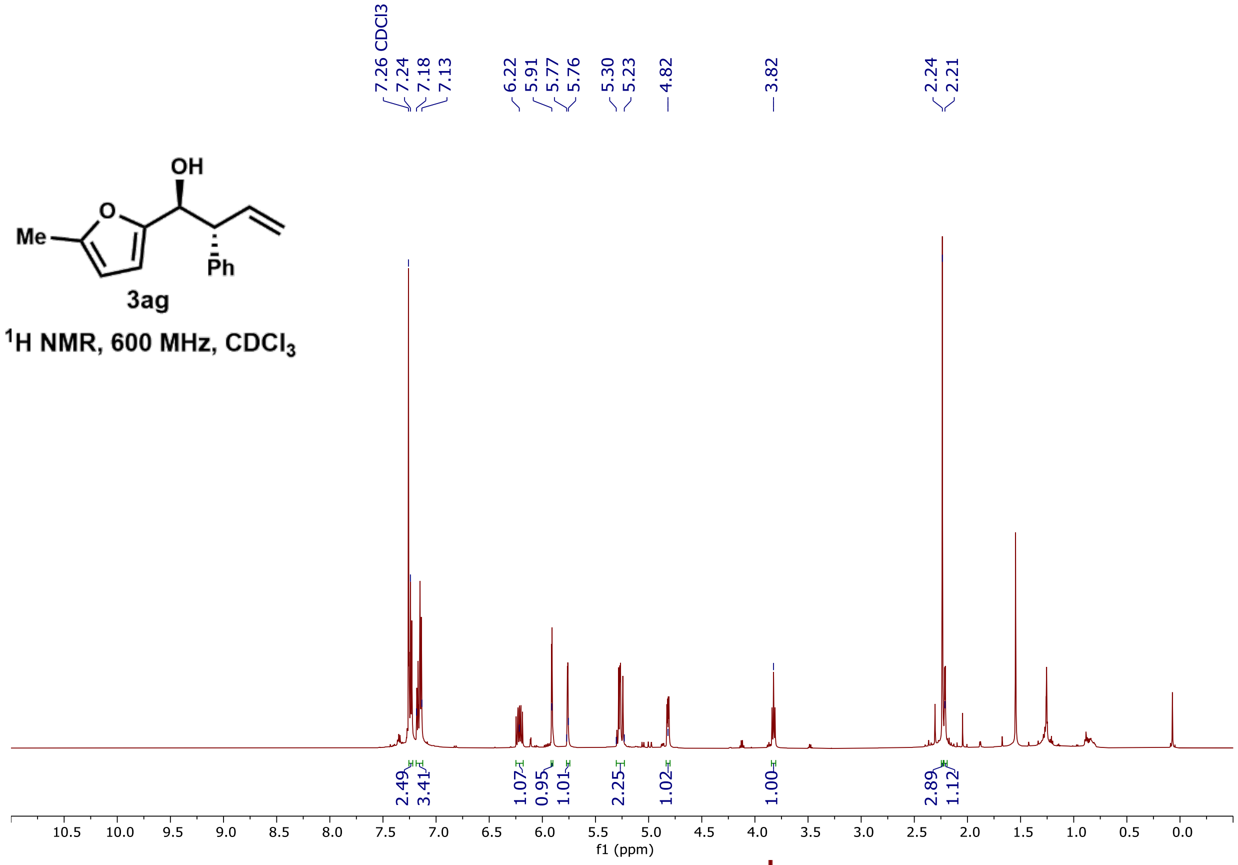
7.26 CDCl3
7.24
7.18
7.13
6.22
5.91
5.77
5.76
5.30
5.23
4.82
3.82
2.24
2.21
OH
O
Me
Ph
3ag
^{1}H NMR, 600 MHz, $CDCl_3$
2.49
3.41
1.07
0.95
1.01
2.25
1.02
1.00
2.89
1.12
10.5 10.0 9.5 9.0 8.5 8.0 7.5 7.0 6.5 6.0 5.5 5.0 4.5 4.0 3.5 3.0 2.5 2.0 1.5 1.0 0.5 0.0
f1 (ppm)

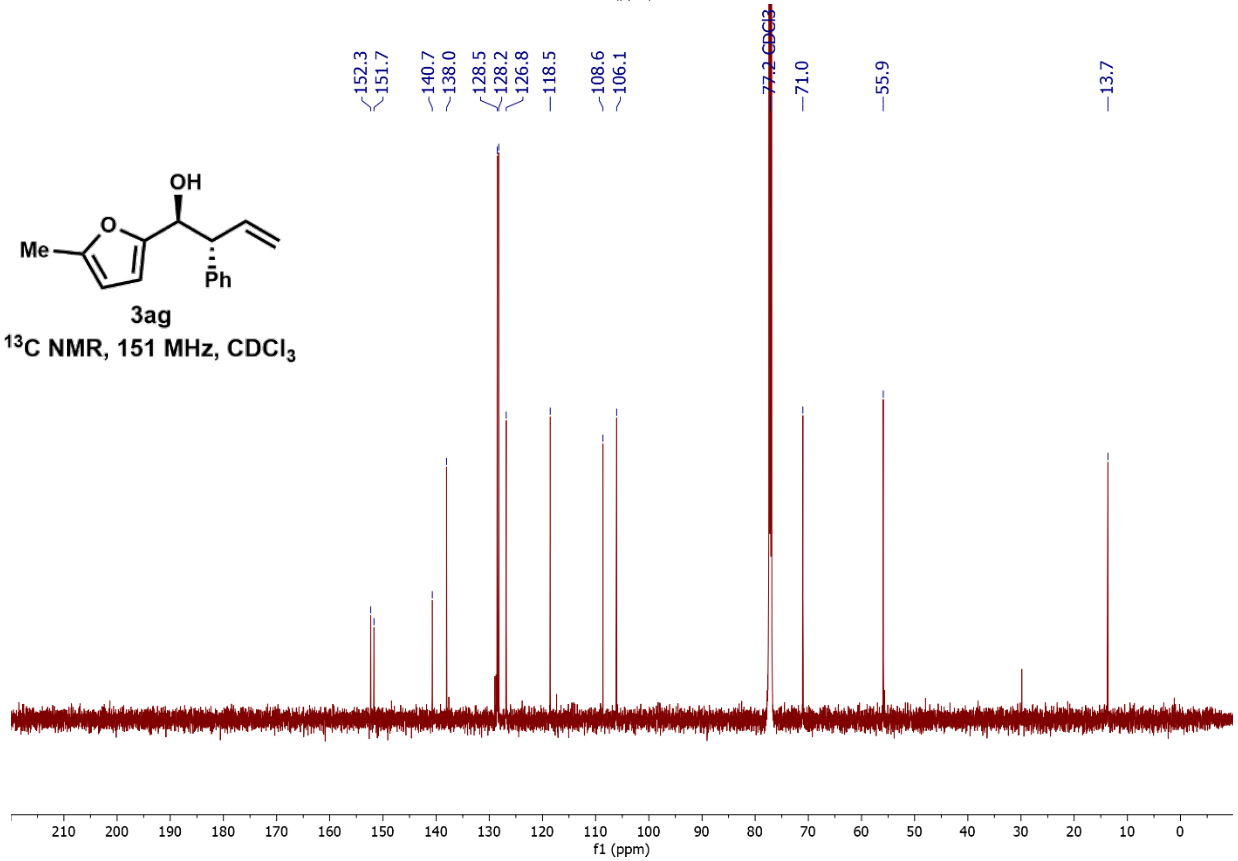
152.3
151.7
140.7
138.0
128.5
128.2
126.8
118.5
108.6
106.1
77.2 CDCl3
71.0
55.9
13.7
OH
O
Me
Ph
3ag
^{13}C NMR, 151 MHz, $CDCl_3$
210 200 190 180 170 160 150 140 130 120 110 100 90 80 70 60 50 40 30 20 10 0
f1 (ppm)

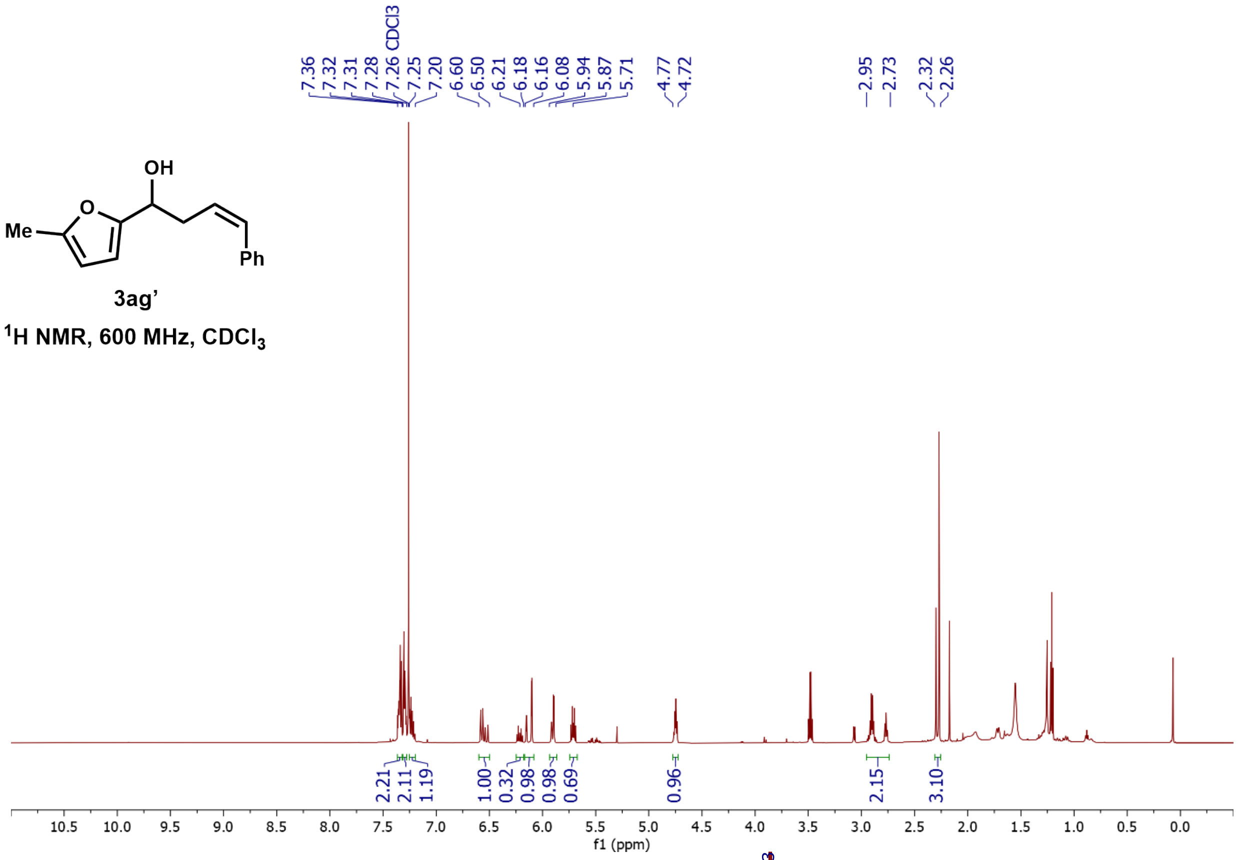

3ag'

^{1}H NMR, 600 MHz, $CDCl_3$

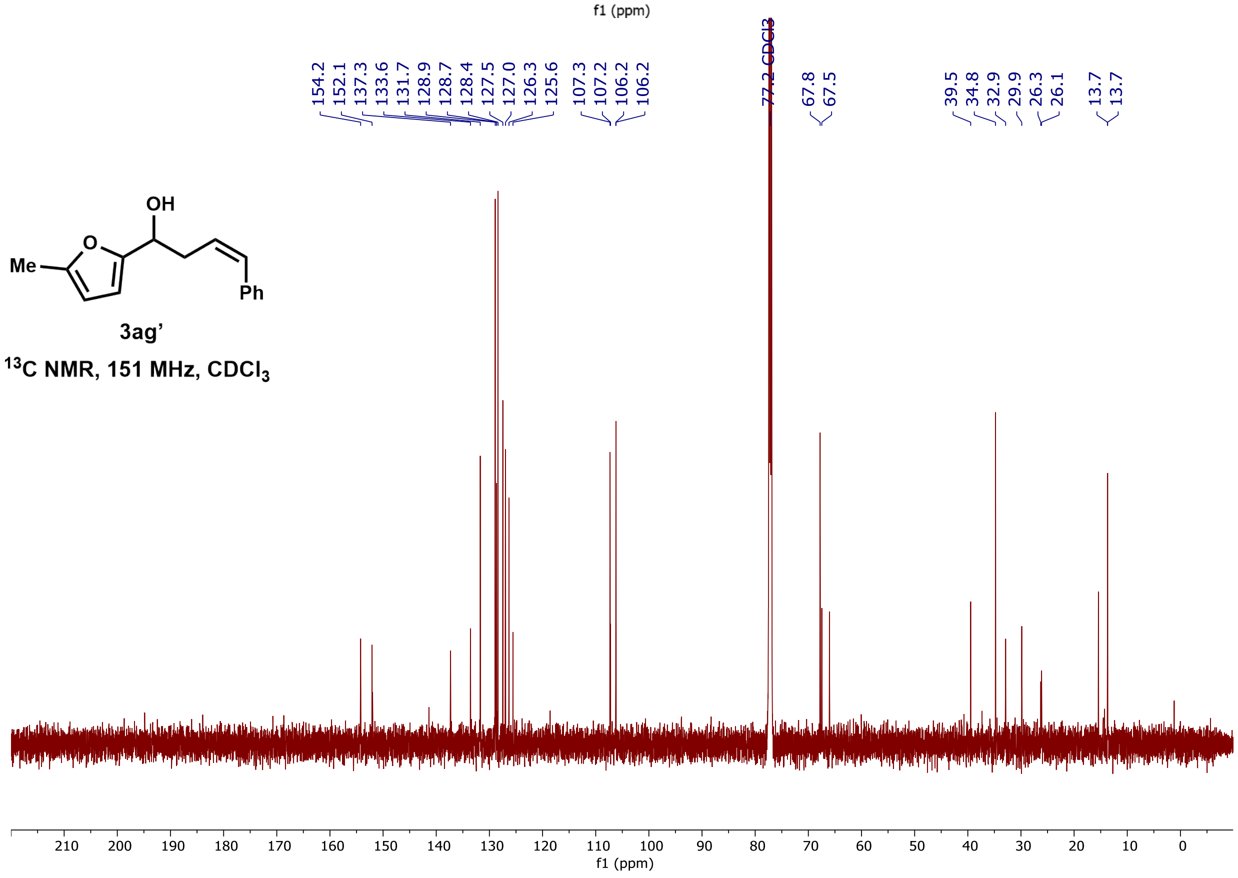

3ag'

^{13}C NMR, 151 MHz, $CDCl_3$

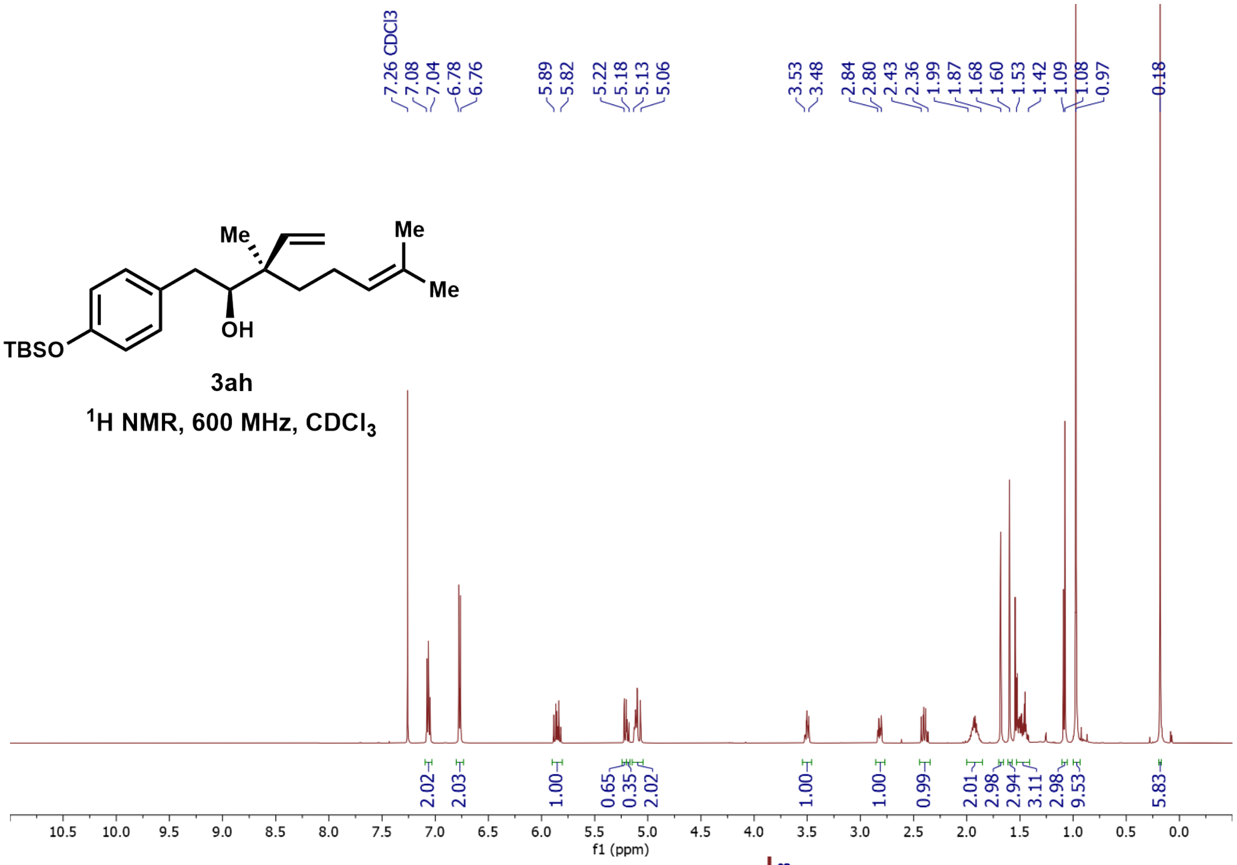

3ah

^{1}H NMR, 600 MHz, $CDCl_3$

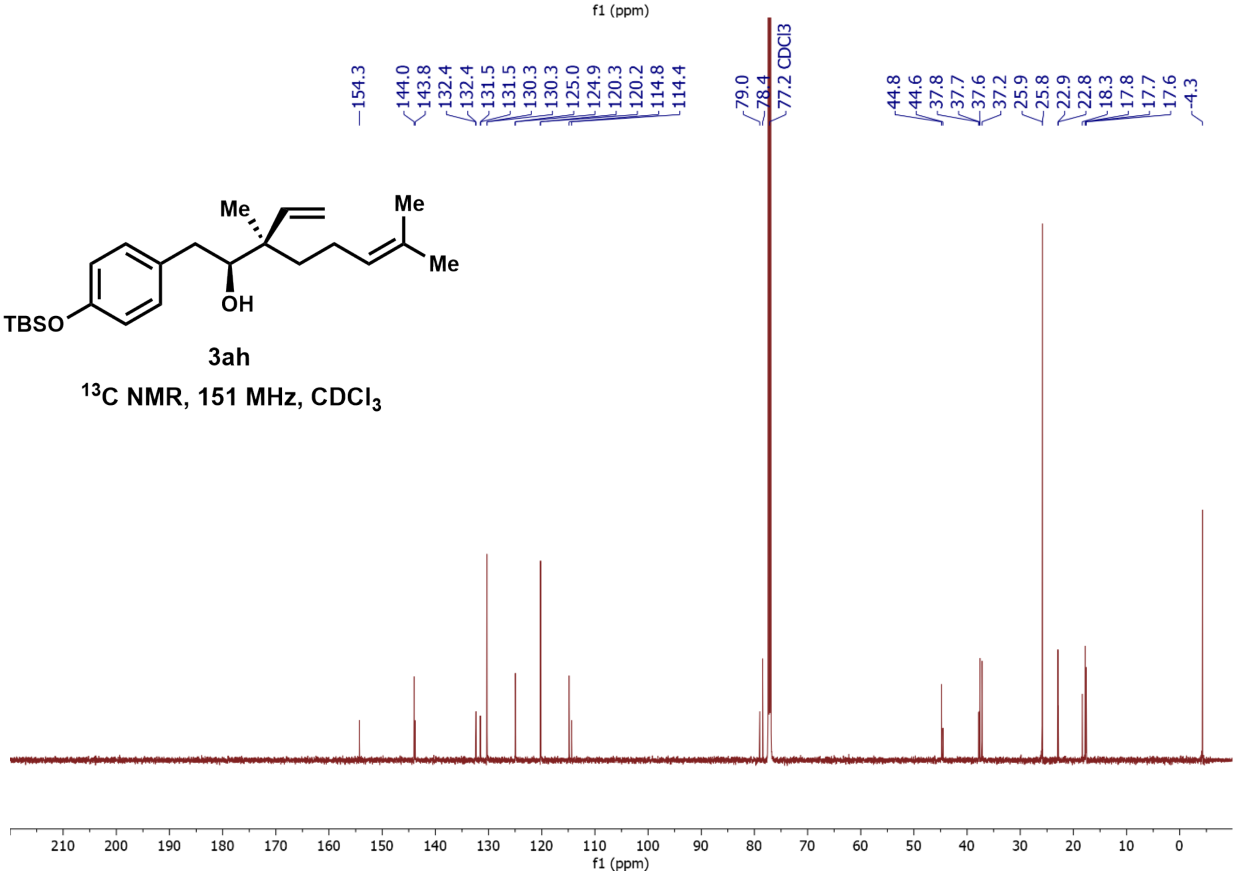

3ah

^{13}C NMR, 151 MHz, $CDCl_3$

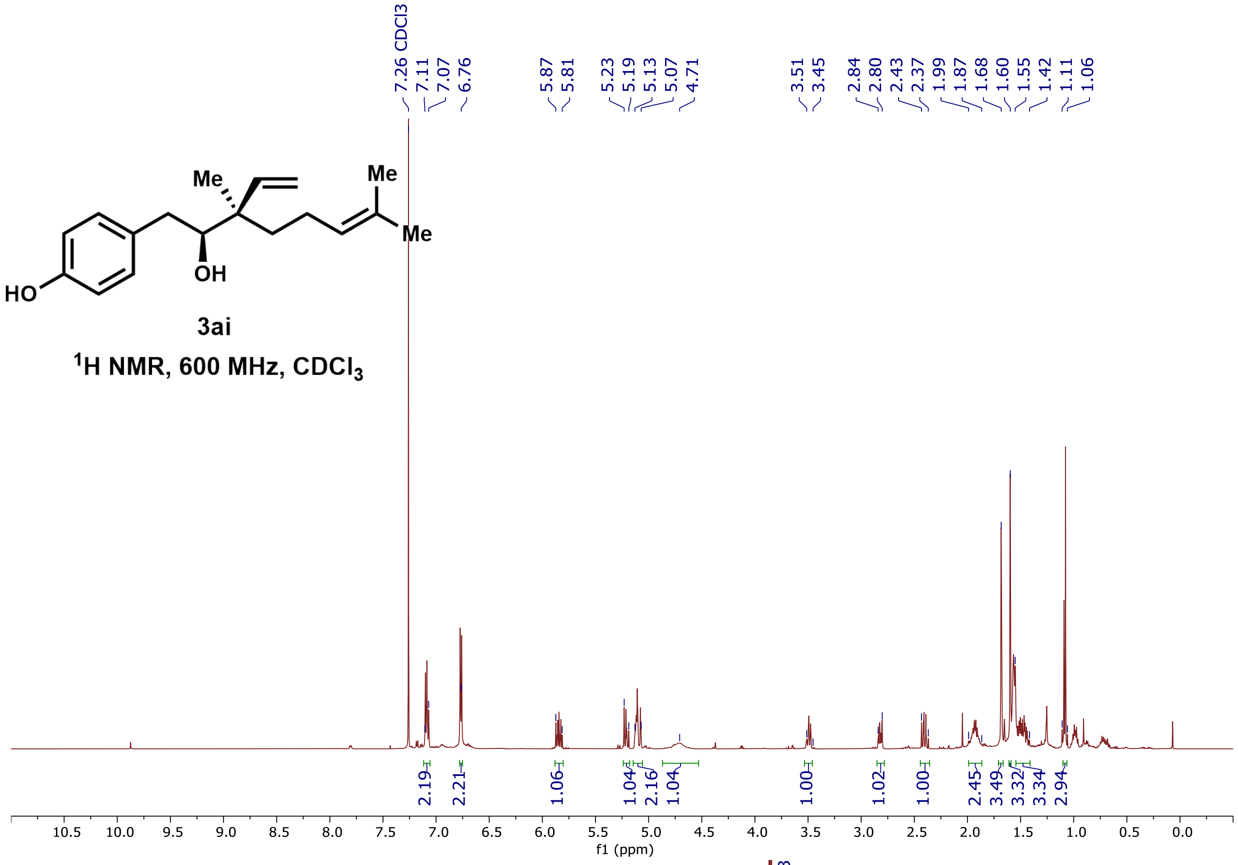

3ai

^{1}H NMR, 600 MHz, CDCl$_3$

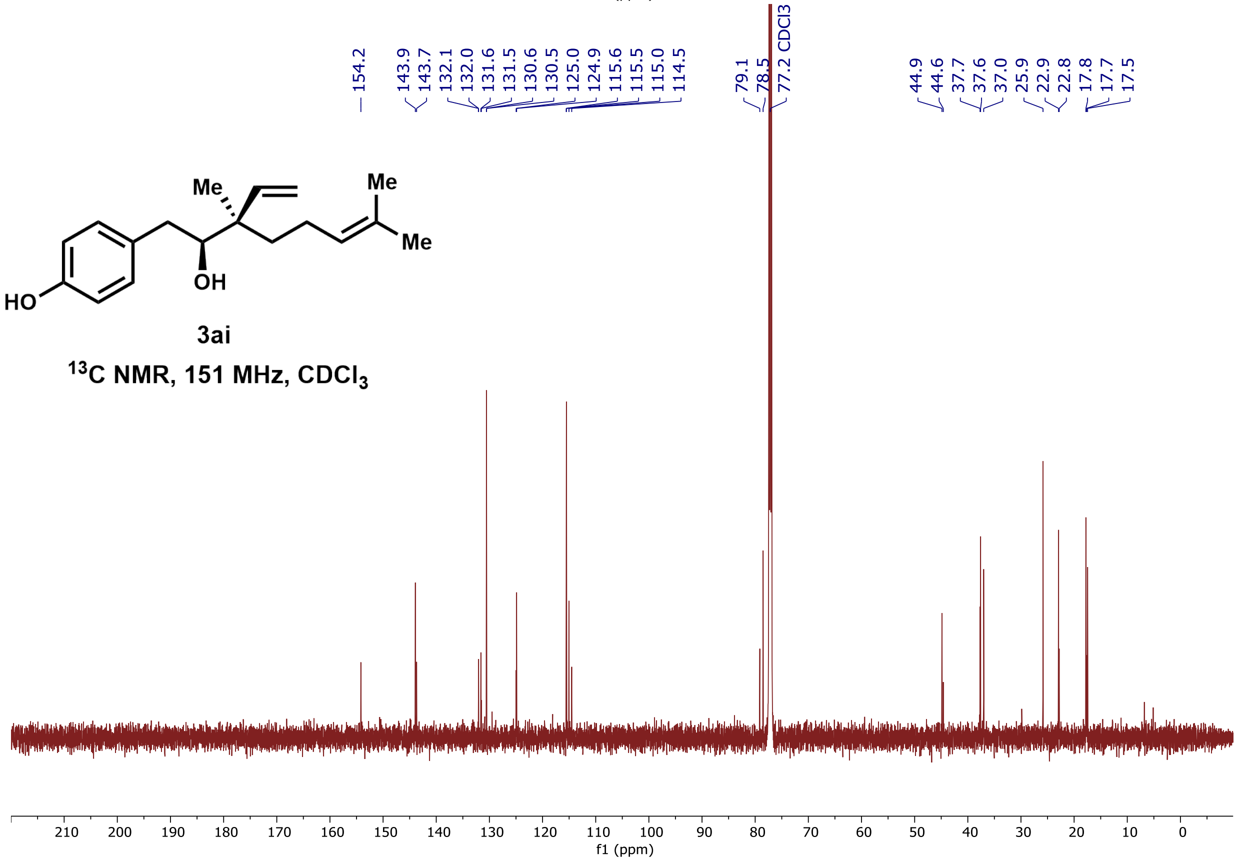

3ai

^{13}C NMR, 151 MHz, CDCl$_3$

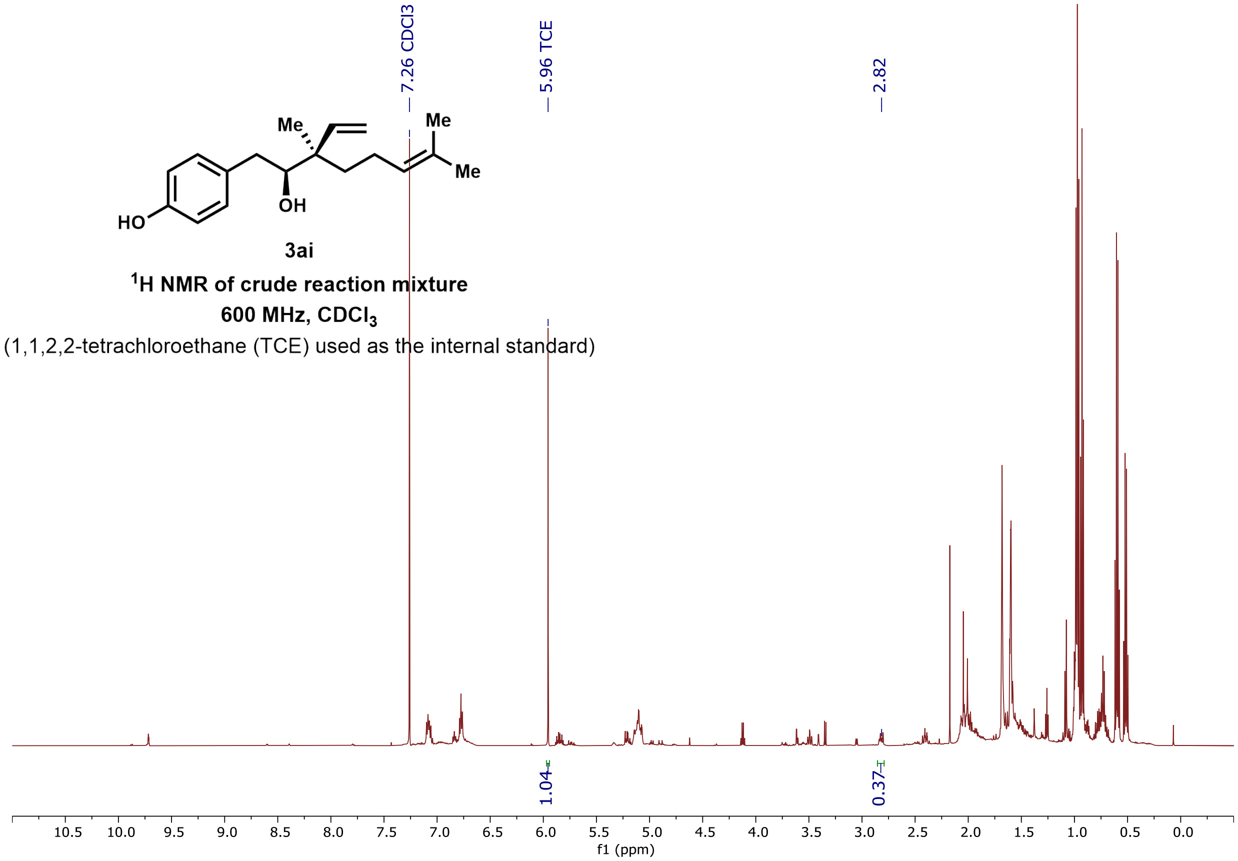
Me
Me
Me
HO
OH
3ai
1H NMR of crude reaction mixture
600 MHz, CDCl3
(1,1,2,2-tetrachloroethane (TCE) used as the internal standard)
7.26 CDCl3
5.96 TCE
2.82
1.04
0.37
10.5 10.0 9.5 9.0 8.5 8.0 7.5 7.0 6.5 6.0 5.5 5.0 4.5 4.0 3.5 3.0 2.5 2.0 1.5 1.0 0.5 0.0
f1 (ppm)

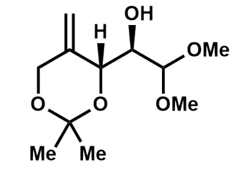

3aj

^{1}H NMR, 600 MHz, $CDCl_3$

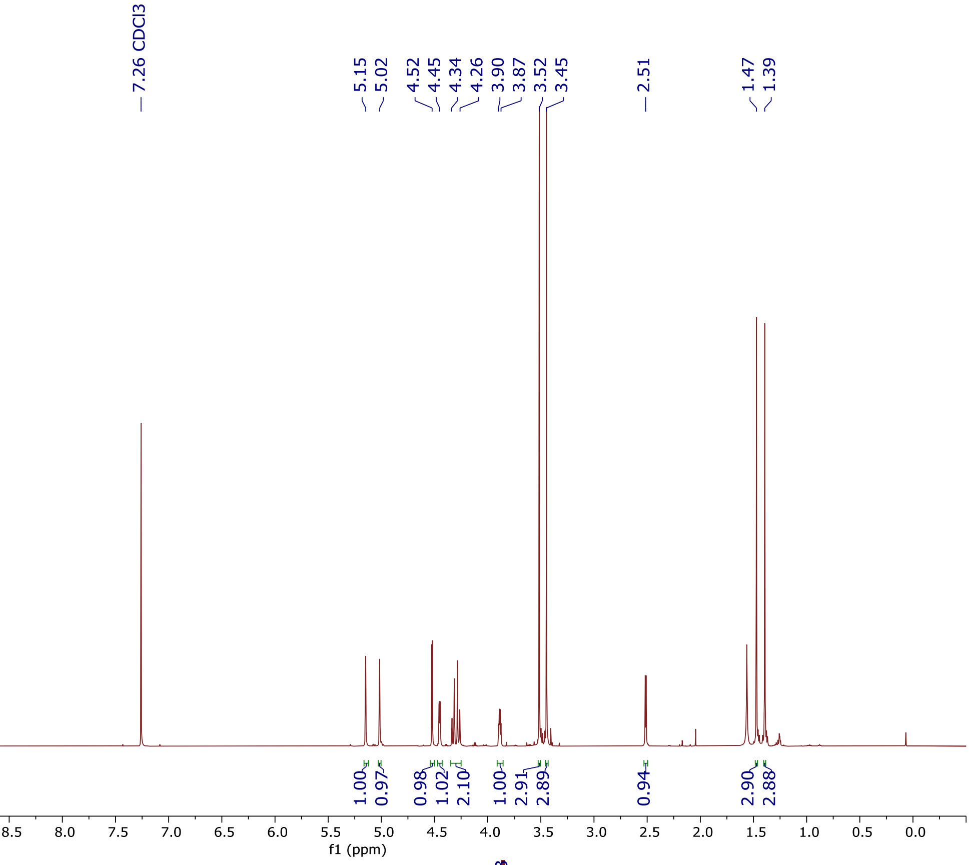

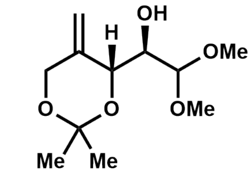

3aj

^{13}C NMR, 151 MHz, $CDCl_3$

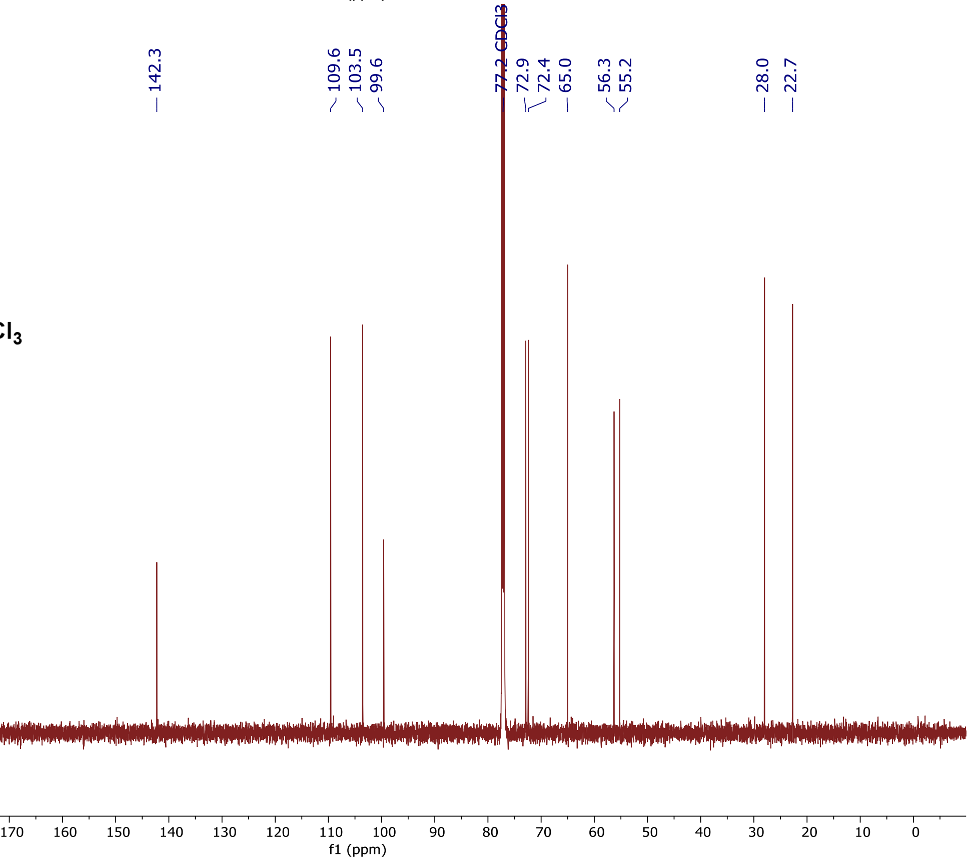

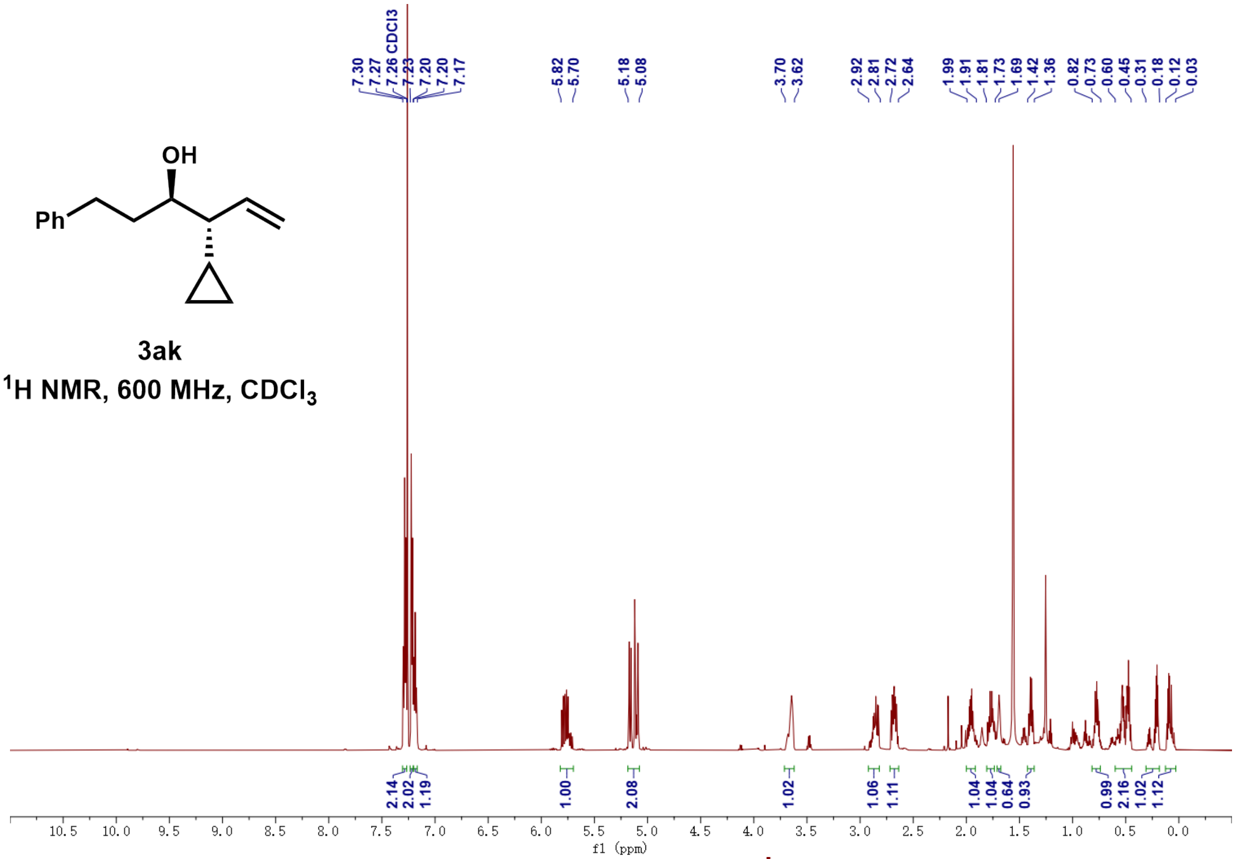

3ak

^{1}H NMR, 600 MHz, $CDCl_3$

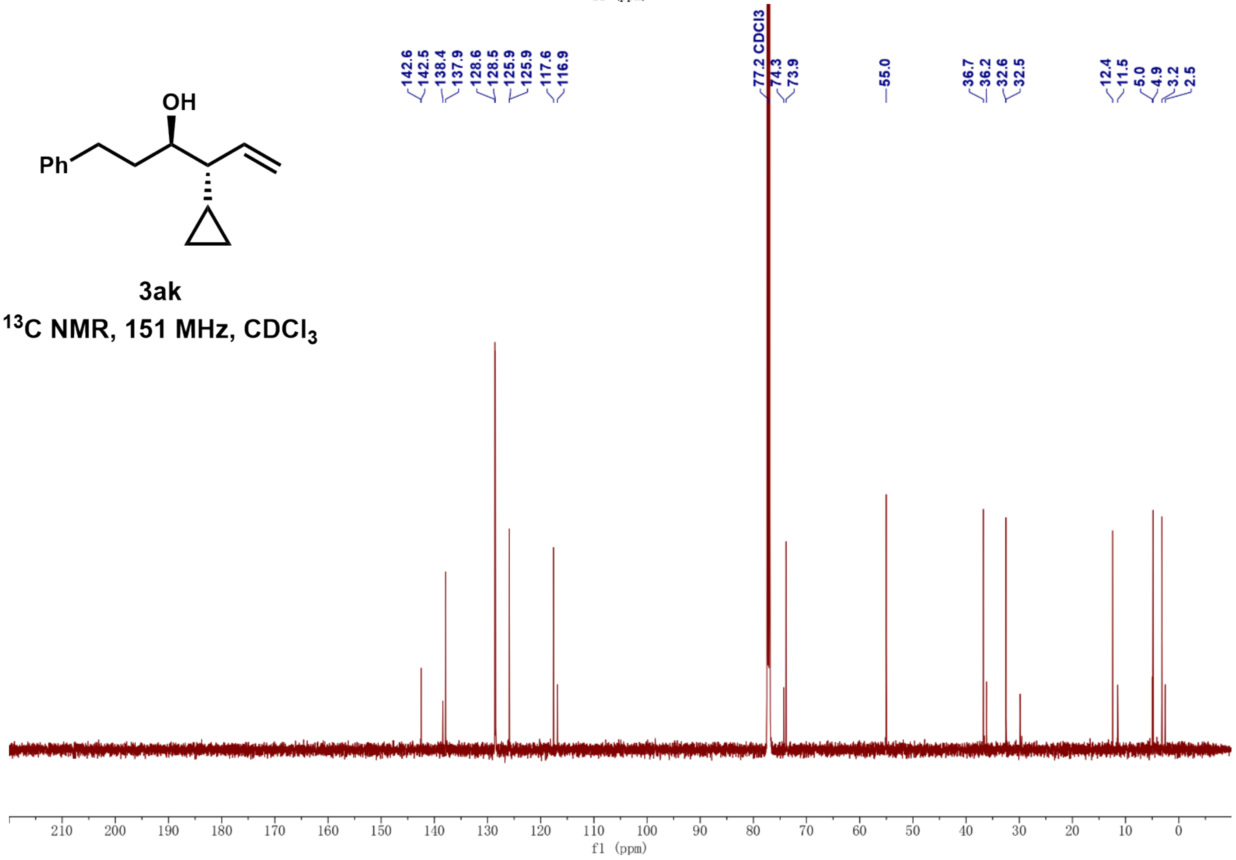

3ak

^{13}C NMR, 151 MHz, $CDCl_3$

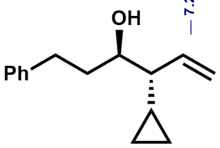

3ak

^{1}H NMR of crude reaction mixture

600 MHz, $CDCl_3$

(1,1,2,2-tetrachloroethane (TCE) used as the internal standard)

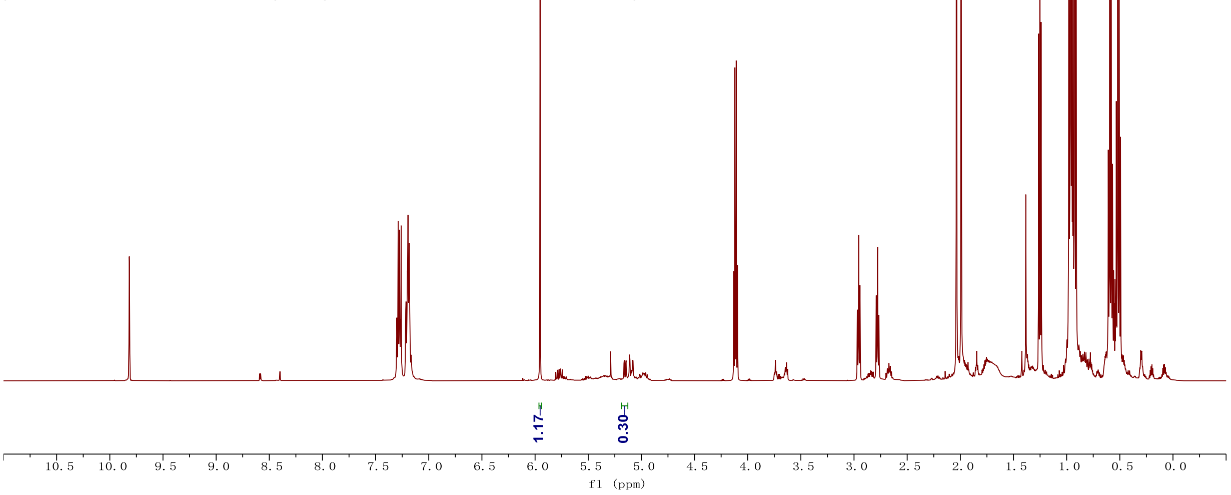

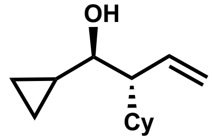

SI-15

^{1}H NMR, 600 MHz, $CDCl_3$

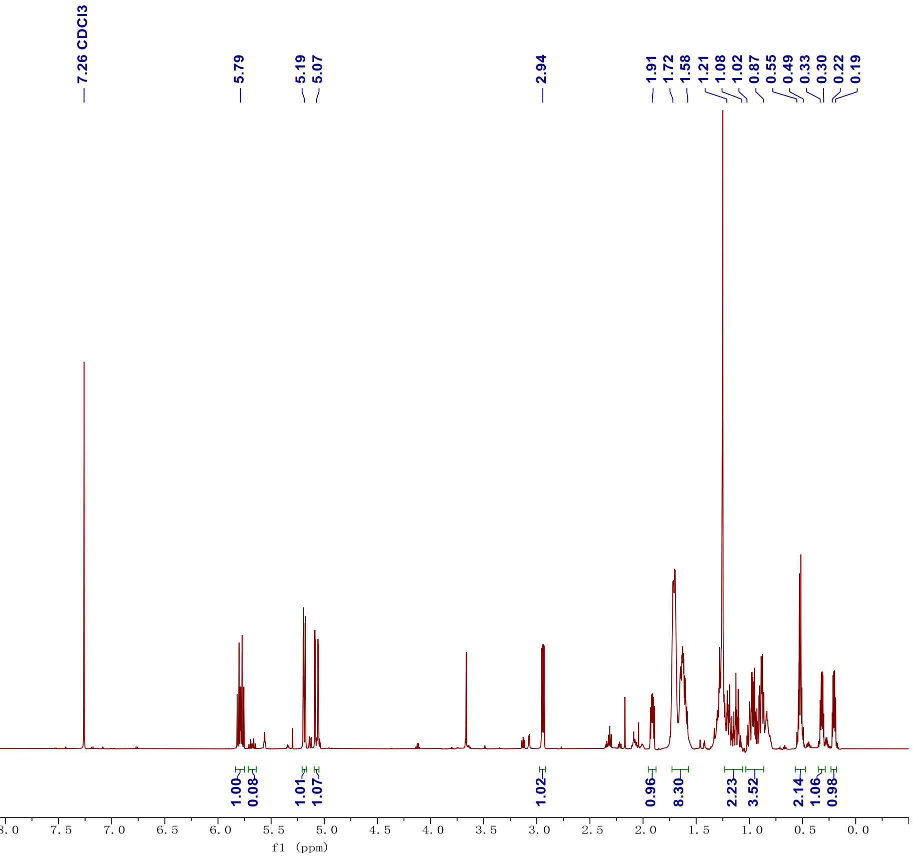

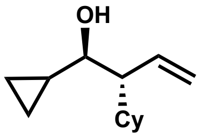

SI-15

^{13}C NMR, 151 MHz, $CDCl_3$

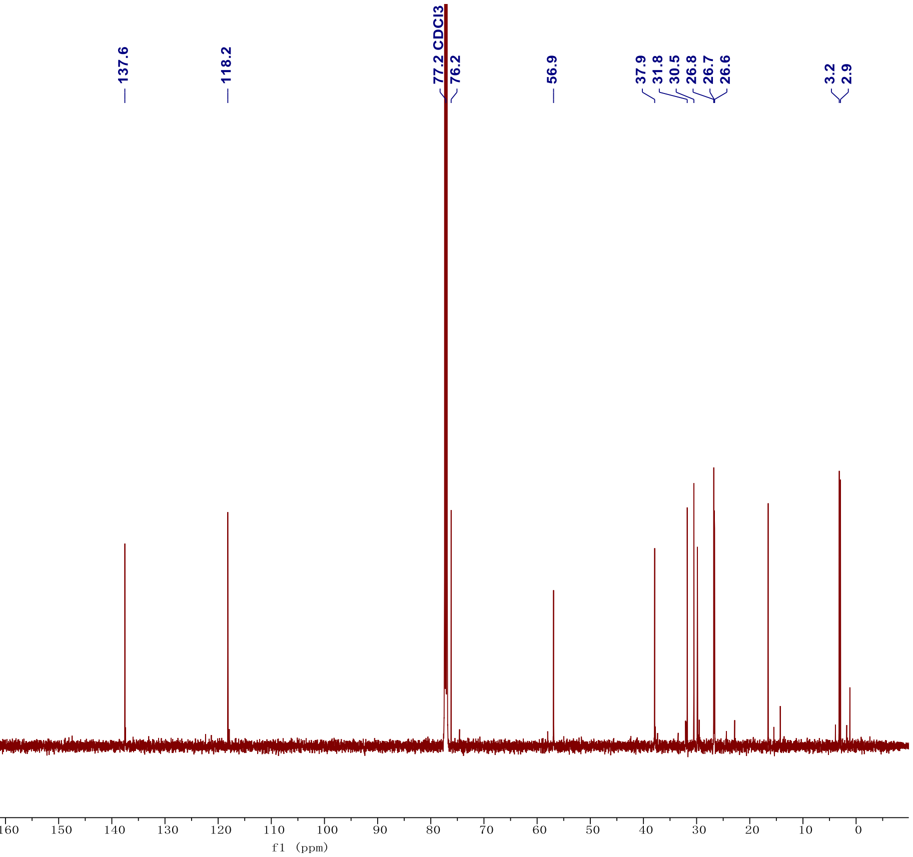

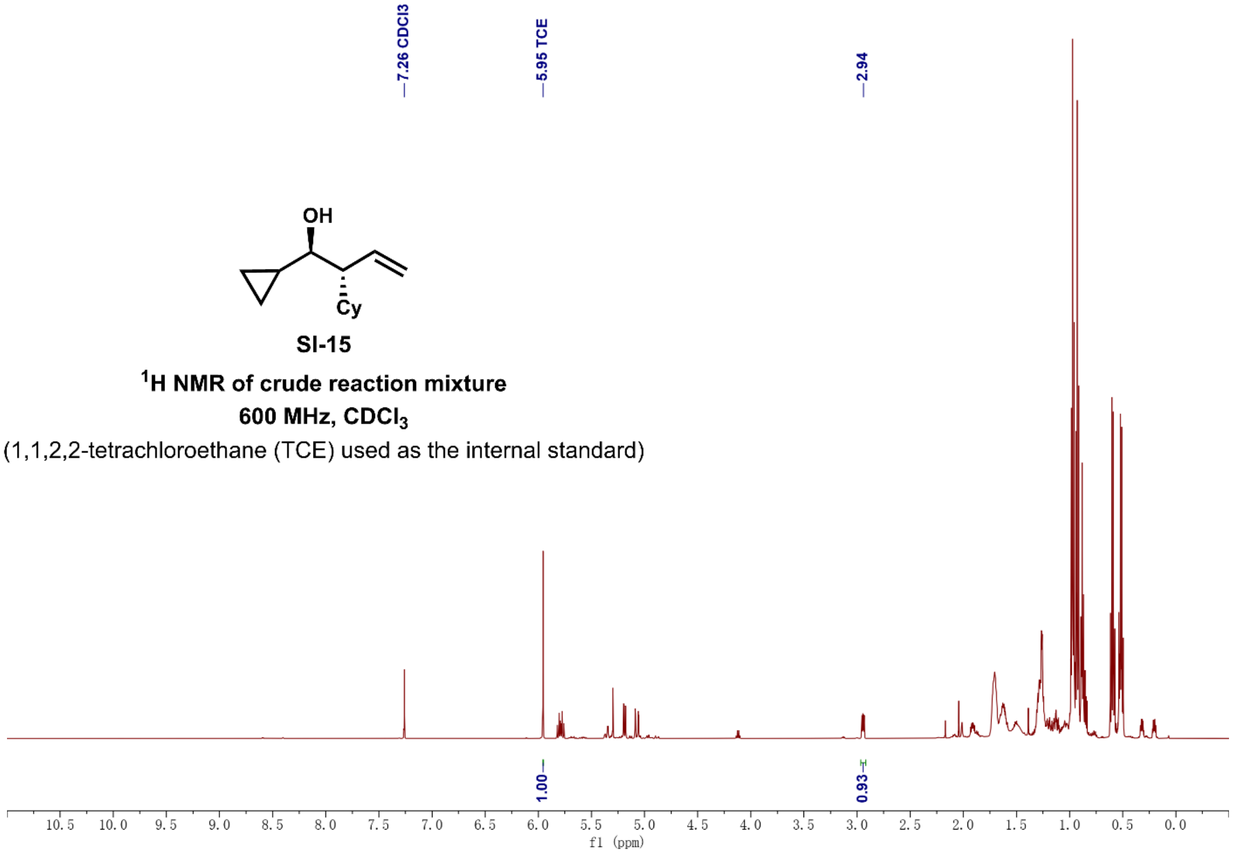

SI-15

^{1}H NMR of crude reaction mixture

600 MHz, $CDCl_3$

(1,1,2,2-tetrachloroethane (TCE) used as the internal standard)

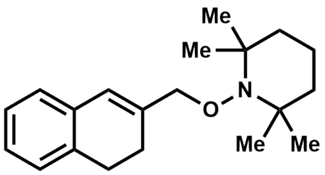

7

^{1}H NMR, 600 MHz, $CDCl_3$

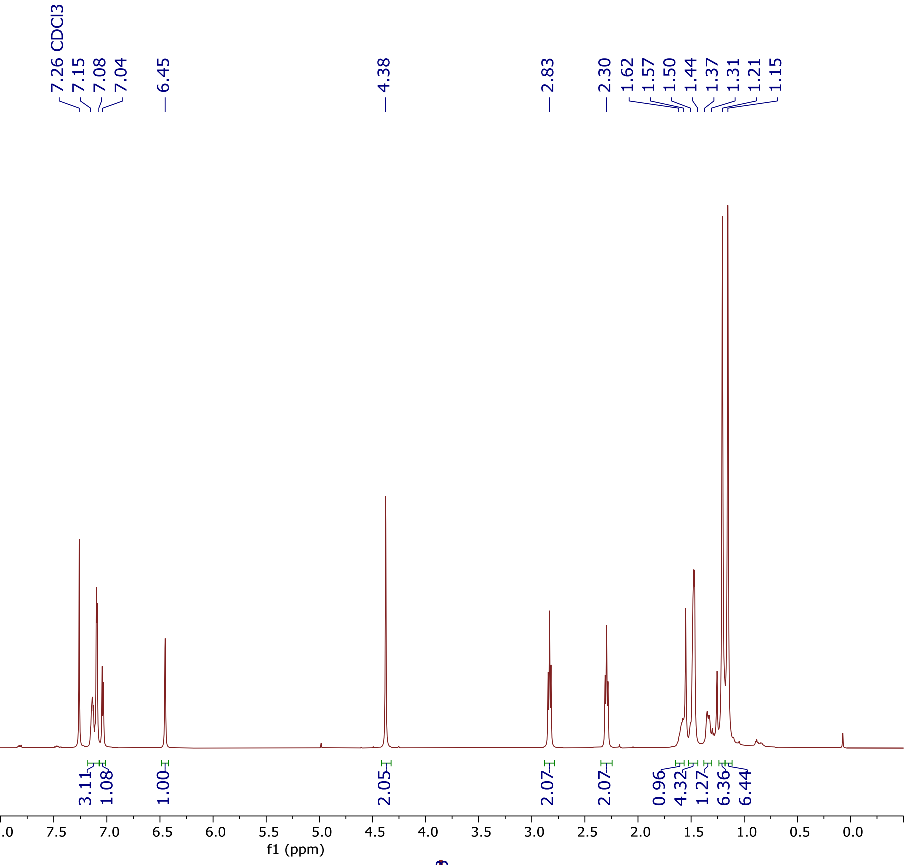

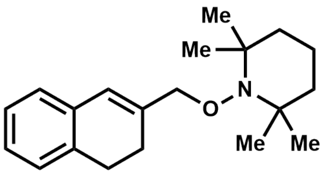

7

^{13}C NMR, 151 MHz, $CDCl_3$

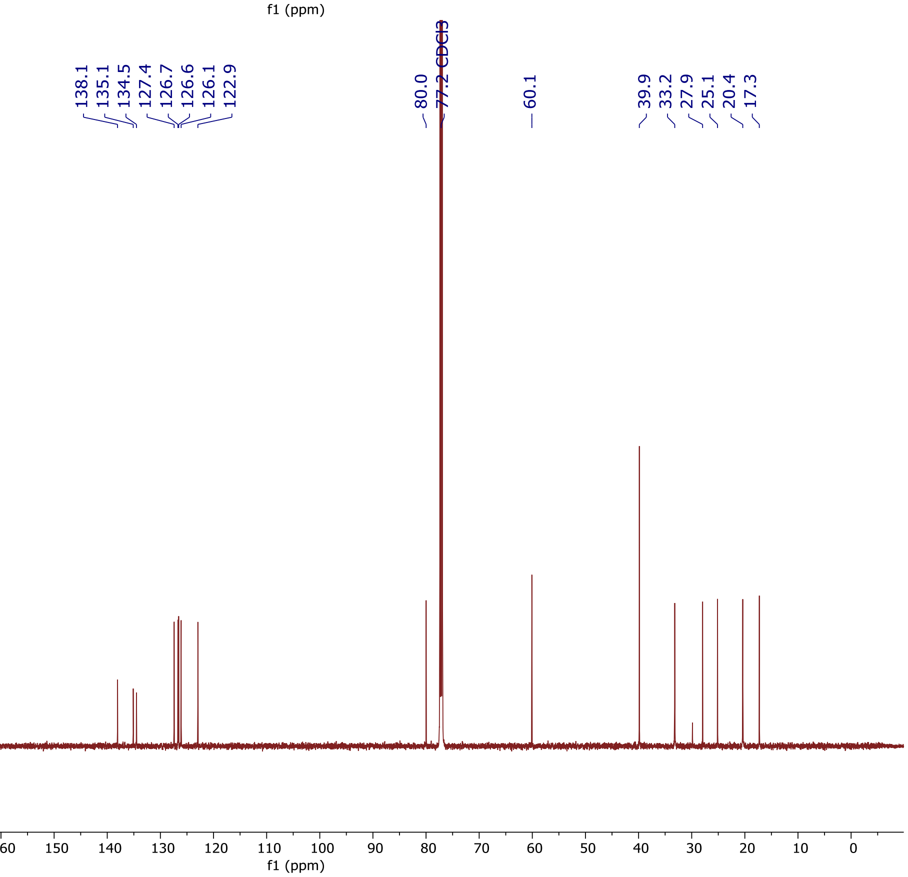

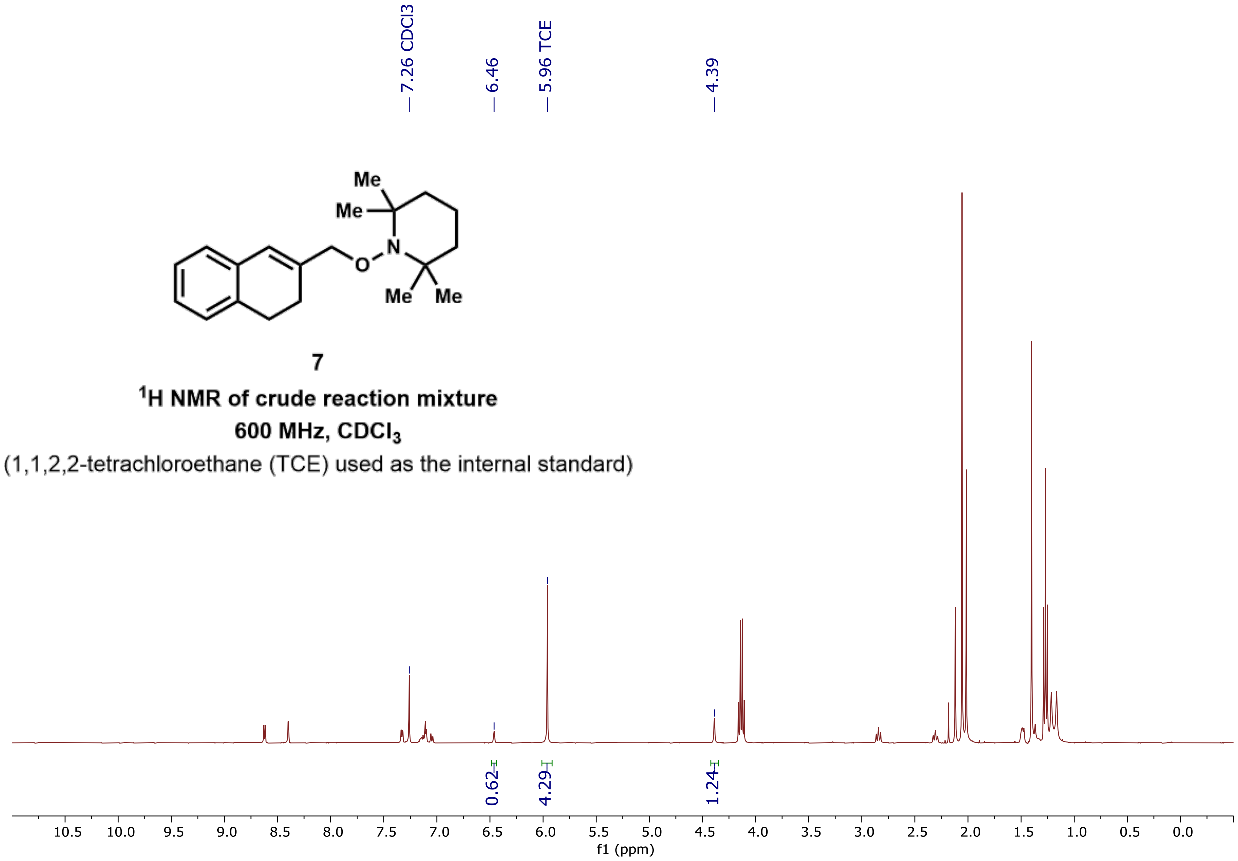

7

^{1}H NMR of crude reaction mixture

600 MHz, $CDCl_3$

(1,1,2,2-tetrachloroethane (TCE) used as the internal standard)

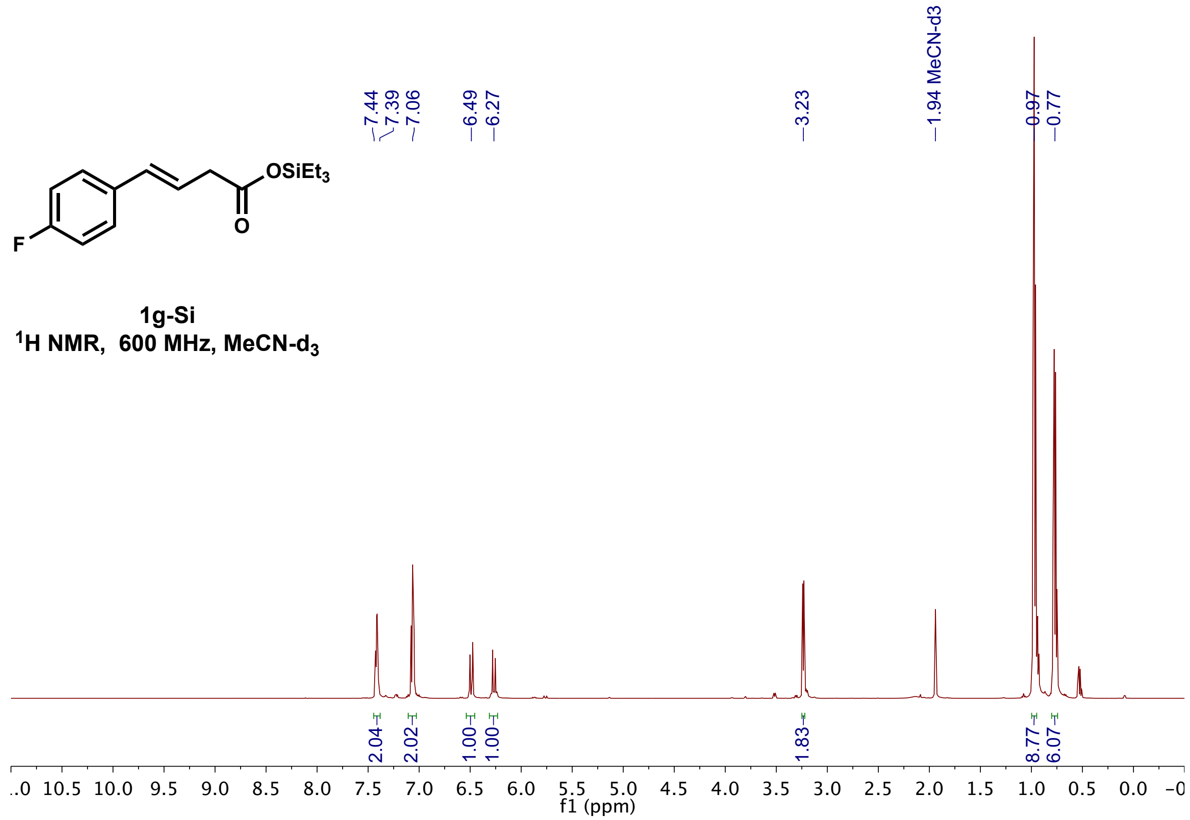
OSiEt3
O
F
1g-Si
1H NMR, 600 MHz, MeCN-d3
7.44
7.39
7.06
6.49
6.27
3.23
1.94 MeCN-d3
0.97
0.77
2.04
2.02
1.00
1.00
1.83
8.77
6.07
f1 (ppm)

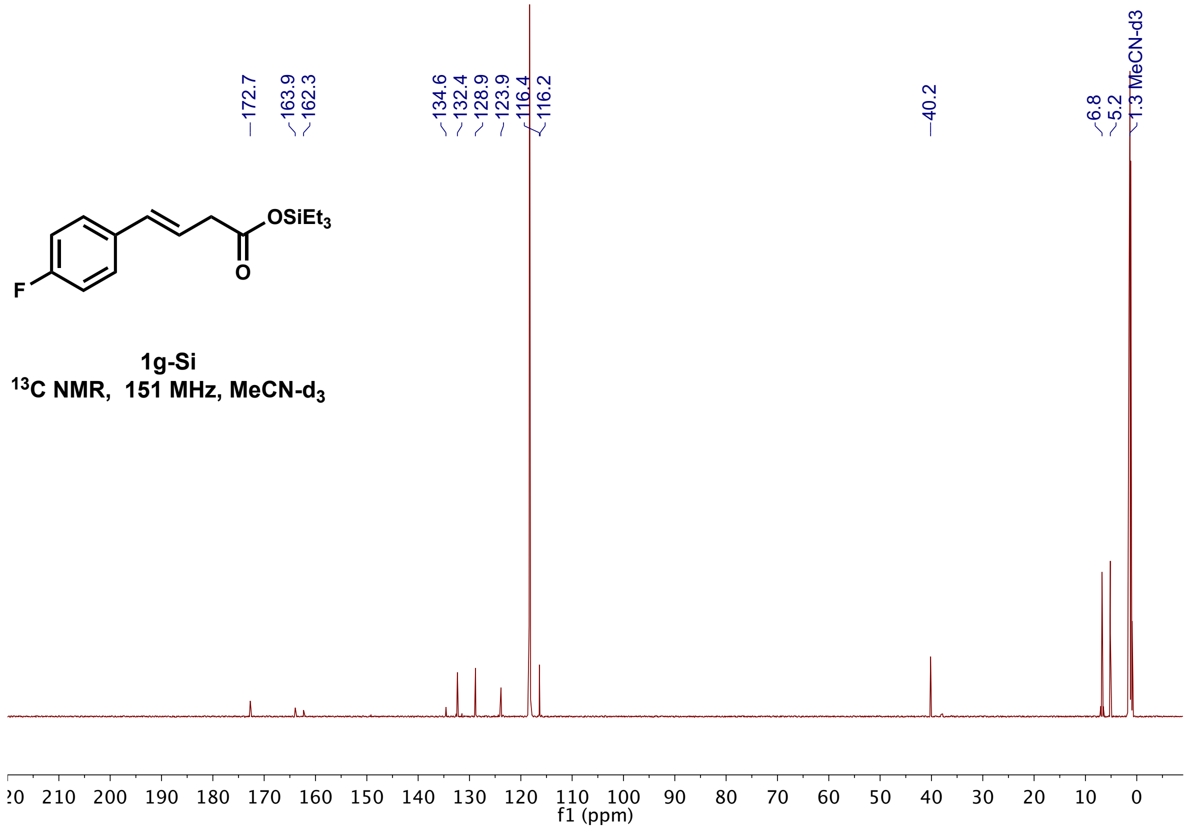
OSiEt3
O
F
1g-Si
13C NMR, 151 MHz, MeCN-d3
172.7
163.9
162.3
134.6
132.4
128.9
123.9
116.4
116.2
40.2
6.8
5.2
1.3 MeCN-d3
f1 (ppm)

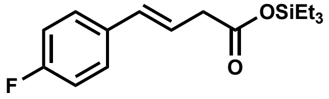

1g-Si

^{19}F NMR, 471 MHz, MeCN-d$_3$

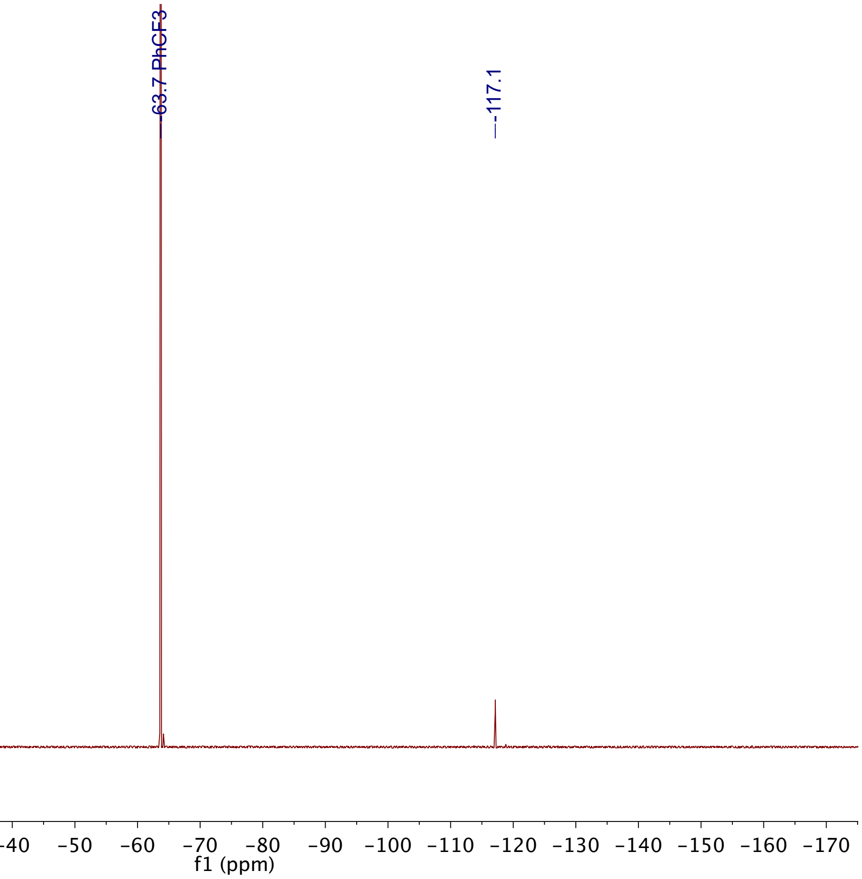

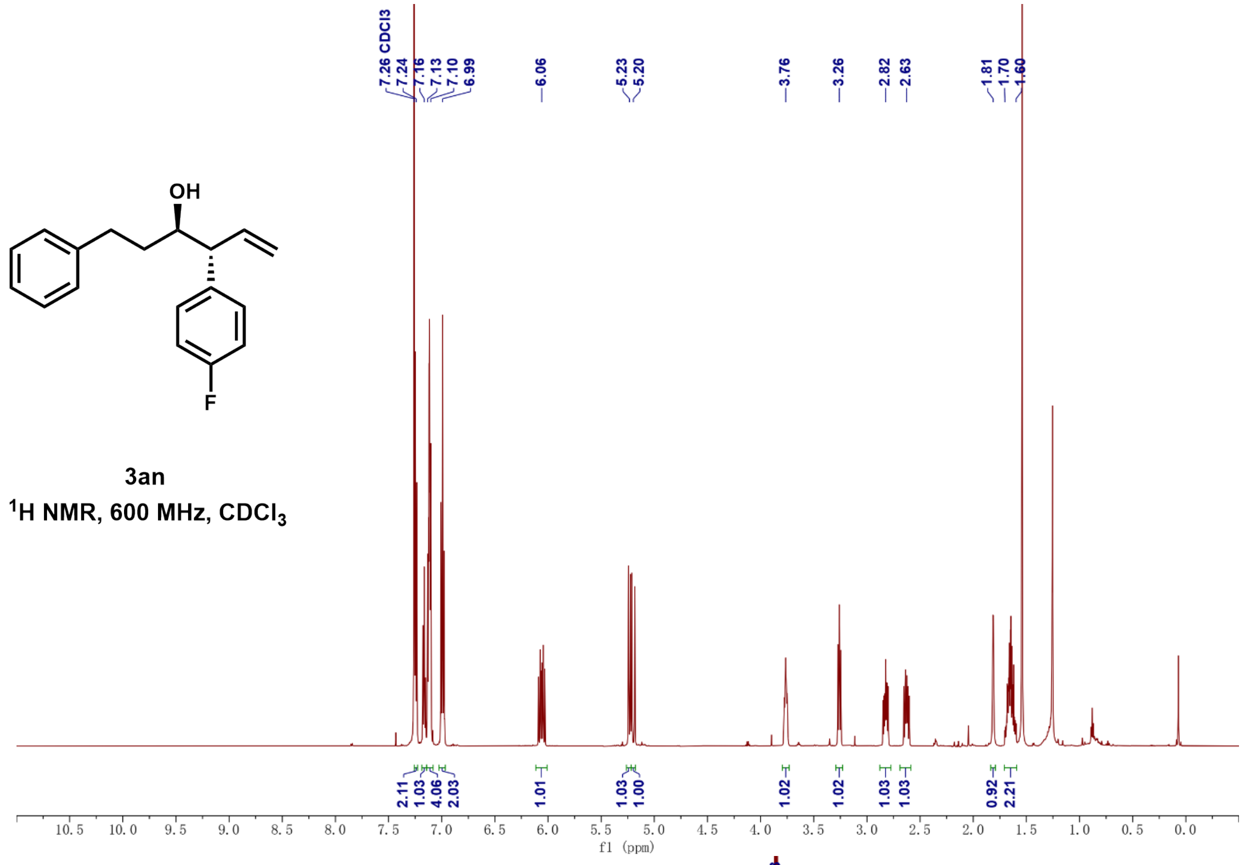

3an

^{1}H NMR, 600 MHz, $CDCl_3$

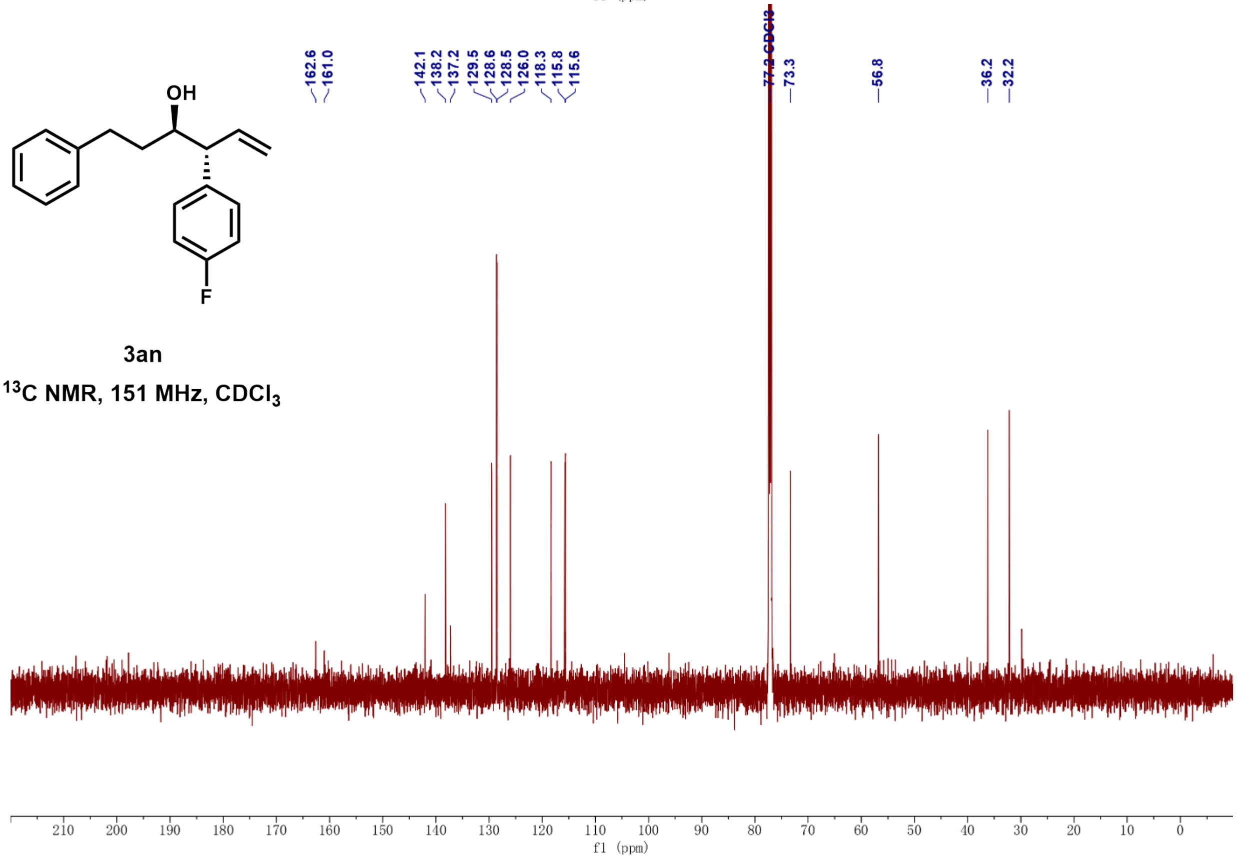

3an

^{13}C NMR, 151 MHz, $CDCl_3$

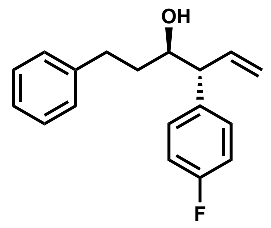

3an

^{19}F NMR, 470 MHz, MeCN-d_3

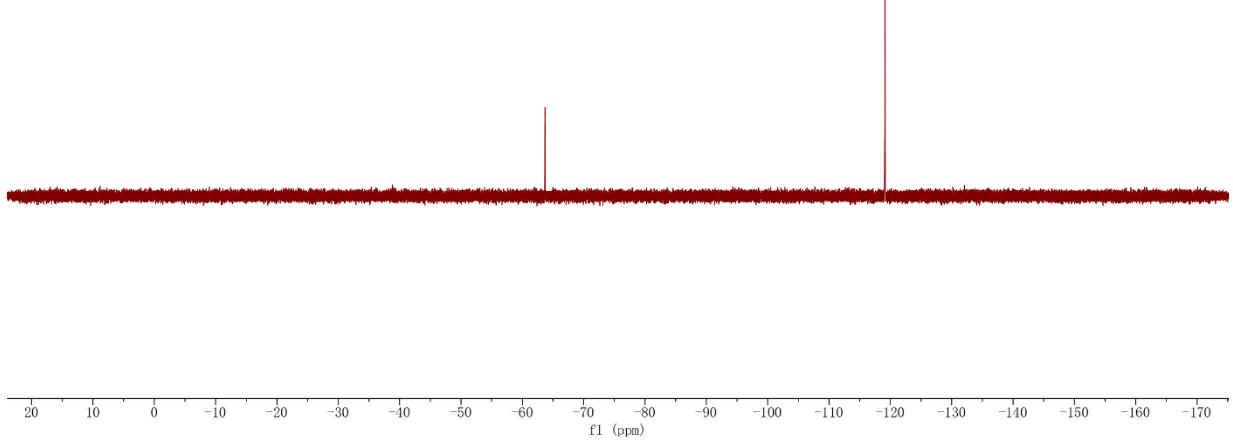

8. References

1. CYLview20; Legault, C. Y., Université de Sherbrooke, **2020** (http://www.cylview.org)

2. Zhang, S.-J.; Hu, W.-X. Method for Regio- and Stereoselective Synthesis of (E)-β,γ-Unsaturated Acids from Aldehydes Under Solvent-Free Conditions. *Synth. Commun.* **2010**, *40* (20), 3093−3100. DOI: 10.1080/00397910903370659.

3. Zhang, H.-J.; Schuppe, A. W.; Pan, S.-T.; Chen, J.-X.; Wang, B.-R.; Newhouse, T. R.; Yin, L. Copper-Catalyzed Vinylogous Aerobic Oxidation of Unsaturated Compounds with Air. *J. Am. Chem. Soc.* **2018**, *140* (15), 5300−5310. DOI: 10.1021/jacs.8b01886.

4. Itaya, T.; Shimizu, S.; Nakagawa, S.; Morisue, M. Syntheses of Optically Active, Protected and Unprotected Vinylglycines. *Chem. Pharm. Bull.* **1994**, *42* (9), 1927−1930. DOI: 10.1248/cpb.42.1927.

5. Fu, M.-C.; Shang, R.; Cheng, W.-M.; Fu, Y. Efficient Pd-Catalyzed Regio- and Stereoselective Carboxylation of Allylic Alcohols with Formic Acid. *Chem. Eur. J.* **2017**, *23* (37), 8818–8822. DOI: 10.1002/chem.201701971.

6. Rode, K.; Palomba, M.; Ortgies, S.; Rieger, R.; Breder, A. Aerobic Allylation of Alcohols with Non-Activated Alkenes Enabled by Light-Driven Selenium-π-Acid Catalysis. *Synthesis* **2018**, *50* (19), 3875−3885. DOI: 10.1055/s-0037-1609938.

7. Singh, R. P.; Das, J.; Yousufuddin, M.; Gout, D.; Lovely, C. J. Tandem Oxidative Dearomatizing Spirocyclizations of Propargyl Guanidines and Ureas. *Org. Lett.* **2017**, *19* (15), 4110−4113. DOI: 10.1021/acs.orglett.7b01898.

8. Sunazuka, T.; Tabata, N.; Nagamitsu, T.; Tomoda, H.; Ōmura, S.; Smith, A. B. Asymmetric synthesis of the anticoccidial antibiotic diolmycin A1. Determination of absolute stereochemistry. *Tetrahedron Lett.* **1993**, *34* (42), 6659−6660. DOI: 10.1016/S0040-4039(00)61668-4.

9. Rosenbaum, N.; Schmidt, L.; Mohr, F.; Fuhr, O.; Nieger, M.; Bräse, S. Formal Semisynthesis of Demethylgorgosterol Utilizing a Stereoselective Intermolecular Cyclopropanation Reaction. *Eur. J. Org. Chem.* **2021**, *10*, 1568−1574. DOI: 10.1002/ejoc.202100035.

10. Andersen, P.; Jens, J. Configurational Correlations of cis-Bis(2,2'-bipyridine) and of cis-Bis(1,10-phenanthroline) Complexes of Trivalent Metals by Means of X-Ray Powder Photographs. *Acta. Chem. Scand.* **1971**, *25*, 3255−3260. DOI: 10.3891/acta.chem.scand.25-3255.

11. Strenger, I.; Rosu, T.; Negoiu, M. **R**efinement of the crystal structure of cw-bis(2,2'-bipyridyl)- dichlorocobalt(III) chloride dihydrate, $[C_{20}H_{16}N_4CoCl_2]Cl \cdot 2H_2O$. *Z. fur Krist. – New Cryst. Struct.* **2000**, *215*, 489−490. DOI: 10.1515/ncrs-2000-0415.

12. Lahuerta, P.; Latorre, J.; Martínez-Máñez, R.; García-Granda, S.; Gómez-Beltrán, F. Structure of bis(2,2'-bipyridine)dichlororhodium(III) chloride dihydrate. *Acta. Cryst.* **1991**, *47*, 519−522. DOI: 10.1107/S010827019000960X.

13. Doistau, B.; Collet, G.; Bolomey, E. A.; Sadat-Noorbakhsh, V.; Besnard, C.; Piguet, C. Heteroleptic Ter–Bidentate Cr(III) Complexes as Tunable Optical Sensitizers. *Inorg. Chem.* **2018**, *57* (22), 14362–14373. DOI: 10.1021/acs.inorgchem.8b02530.

14. Otto, S.; Grabolle, M.; Förster, C.; Kreitner, C.; Resch-Gener, U.; Heinze, K. $[Cr(ddpd)_2]^{3+}$: A Molecular, Water-Soluble, Highly NIR-Emissive Ruby Analogue. *Angew. Chem. Int. Ed.* **2015**, *54* (39), 11572−11576. DOI: 10.1002/anie.201504894.

15. Namba, K.; Kishi, Y. Catalytic Ni/Cr-Mediated Macrocyclization without Use of High-Dilution Techniques. *J. Am. Chem. Soc.* **2005**, *127* (44), 15382−15383. DOI: 10.1021/ja055966v.

16. van Gemmeren, M.; Börjesson, M.; Tortajada, A.; Sun, S. -Z.; Okura, K.; Martin, R. Switchable Site-Selective Catalytic Carboxylation of Allylic Alcohols with CO_2. *Angew. Chem. Int. Ed.* **2017**, *56* (23), 6558−6562. DOI: 10.1002/anie.201702857.

17. Sundberg, R. J.; Holcombe, F. O. Synthesis and stereochemical characterization of derivatives of cis- and trans-3-ethyl-4-piperidineacetic acid. *J. Org. Chem.* **1969**, *34* (11), 3273−3279. DOI: 10.1021/jo01263a012.

18. Van der Veen, R. H.; Cerfontain, H. Temperature-dependent alkylation of .gamma.-phenyl .beta.,.gamma.-unsaturated acid and ester systems in hexamethylphosphoric triamide-tetrahydrofuran solutions using lithium diisopropylamide. *J. Org. Chem.* **1985**, *50* (3), 342−346. DOI: 10.1021/jo00203a011.

19. Pinosa, E.; Bassan, E.; Cetin, S.; Villa, M.; Potenti, S.; Calogero, F.; Gualandi, A.; Fermi, A.; Ceroni, P.; Cozzi, P. G. Light-Induced Access to Carbazole-1,3-dicarbonitrile: A Thermally Activated Delayed Fluorescent (TADF) Photocatalyst for Cobalt-Mediated Allylations. *J. Org. Chem.* **2023**, *88* (10), 6390−6400. DOI: 10.1021/acs.joc.2c01825.

20. Hoye, T. R.; Jeffrey, C. S.; Shao, F. Mosher ester analysis for the determination of absolute configuration of stereogenic (chiral) carbinol carbons. *Nat. Protoc.* **2007**, *2*, 2451−2458. DOI: 10.1038/nprot.2007.354.

21. Huang, M. -Y.; Chen, L.; Li, R.; Jia, X.; Hong, R. Synthesis of (±)-Bakuchiol via a Pot-Economy Approach. *Chin. J. Chem.* **2014**, *32* (8), 715–720. DOI: 10.1002/cjoc.201400160.

22. Grondal, C.; Enders, D. A Direct Organocatalytic Entry to Selectively Protected Aldopentoses and Derivatives. *Adv. Synth. Catal.* **2007**, *349*, 694–702. DOI: 10.1002/adsc.200600573.

23. Sada, M.; Komagawa, S.; Uchiyama, M.; Kobata, M.; Mizuno, T.; Utimoto, K.; Oshima, K.; Matsubara, S. Reaction Pathway of Methylenation of Carbonyl Compounds with Bis(iodozincio)methane. *J. Am. Chem. Soc*. **2010**, *132* (49), 17452–17458. DOI: 10.1021/ja104439w.

24. Candish, L.; Freitag, M.; Gensch, T.; Glorius, F. Mild, visible light-mediated decarboxylation of aryl carboxylic acids to access aryl radicals. *Chem. Sci.* **2017**, *8*, 3618–3622. DOI: 10.1039/C6SC05533H.

25. Demas, J. N.; Bowman, W. D.; Zalewski, E. F.; Velapoldi, R. A. Determination of the quantum yield of the ferrioxalate actinometer with electrically calibrated radiometers. *J. Phys. Chem.* **1981**, *85*, 2766–2771. DOI: 10.1021/j150619a015.

26. Zeng, X.; Zhang, F.-H.; Lai, R.; Lin, X.; Wang, Z. Cr-Catalyzed Allylic C(Sp3)–H Addition to Aldehydes Enabled by Photoinduced Ligand-to-Metal Charge Transfer. *Sci. China Chem.* **2024**. *67*, 1589–1595. DOI: 10.1007/s11426-023-1911-x.

27. Scarborough, C. C.; Sproules, S.; Weyhermüller, T.; DeBeer, S.; Wieghardt, K. Electronic and Molecular Structures of the Members of the Electron Transfer Series [Cr(Tbpy)3]n (n = 3+, 2+, 1+, 0): An X-Ray Absorption Spectroscopic and Density Functional Theoretical Study. *Inorg. Chem.* **2011**, *50* (24), 12446–12462. DOI: 10.1021/ic201123x.

28. Denmark, S. E.; Edwards, M. G. On the Mechanism of the Selenolactonization Reaction with Selenenyl Halides. *J. Org. Chem.* **2006**, *71* (19), 7293–7306. DOI: 10.1021/jo0610457.

29. Dolomanov, O. V.; Bourhis, L. J.; Gildea, R. J.; Howard, J. A. K.; Puschmann, H. OLEX2: a complete structure solution, refinement, and analysis program. *J. Appl. Cryst.* **2009**, *42*, 339–341. DOI: 10.1107/S0021889808042726.

30. Sheldrick, G. M. A short history of *SHELX*. *Acta Crystallogr. Sect. A.* **2008**, *64*, 112–122. DOI: 10.1107/S0108767307043930.

31. Guzei, I. A. An idealized molecular geometry library for refinement of poorly behaved molecular fragments with constraints. *J. Appl. Cryst.* **2014**, *47*, 806–809. DOI: 10.1107/S1600576714004427.
